# Google Health Trends performance reflecting dengue incidence for the Brazilian states

**DOI:** 10.1186/s12879-020-04957-0

**Published:** 2020-03-26

**Authors:** Daniel Romero-Alvarez, Nidhi Parikh, Dave Osthus, Kaitlyn Martinez, Nicholas Generous, Sara del Valle, Carrie A. Manore

**Affiliations:** 1 Department of Ecology & Evolutionary Biology and Biodiversity Institute, University of Kansas, Lawrence, Kansas USA; 2 Information Systems and Modeling (A-1), Los Alamos National Laboratory, Los Alamos, NM USA; 3 Statistical Sciences (CCS-6), Los Alamos National Laboratory, Los Alamos, NM USA; 4 Applied Math and Statistics, Colorado School of Mines, Golden, CO USA; 5 National Security & Defense Program Office (GS-NSD), Los Alamos National Laboratory, Los Alamos, NM USA

**Keywords:** Google health trends, Digital epidemiology, Brazil, Volatility, Epidemiology, Internet data streams, Internet penetration

## Abstract

**Background:**

Dengue fever is a mosquito-borne infection transmitted by *Aedes aegypti* and mainly found in tropical and subtropical regions worldwide. Since its re-introduction in 1986, Brazil has become a hotspot for dengue and has experienced yearly epidemics. As a notifiable infectious disease, Brazil uses a passive epidemiological surveillance system to collect and report cases; however, dengue burden is underestimated. Thus, Internet data streams may complement surveillance activities by providing real-time information in the face of reporting lags.

**Methods:**

We analyzed 19 terms related to dengue using Google Health Trends (GHT), a free-Internet data-source, and compared it with weekly dengue incidence between 2011 to 2016. We correlated GHT data with dengue incidence at the national and state-level for Brazil while using the adjusted R squared statistic as primary outcome measure (0/1). We used survey data on Internet access and variables from the official census of 2010 to identify where GHT could be useful in tracking dengue dynamics. Finally, we used a standardized volatility index on dengue incidence and developed models with different variables with the same objective.

**Results:**

From the 19 terms explored with GHT, only seven were able to consistently track dengue. From the 27 states, only 12 reported an adjusted R squared higher than 0.8; these states were distributed mainly in the Northeast, Southeast, and South of Brazil. The usefulness of GHT was explained by the logarithm of the number of Internet users in the last 3 months, the total population per state, and the standardized volatility index.

**Conclusions:**

The potential contribution of GHT in complementing traditional established surveillance strategies should be analyzed in the context of geographical resolutions smaller than countries. For Brazil, GHT implementation should be analyzed in a case-by-case basis. State variables including total population, Internet usage in the last 3 months, and the standardized volatility index could serve as indicators determining when GHT could complement dengue state level surveillance in other countries.

## Background

Dengue fever is transmitted by the homonymous arthropod-borne virus (i.e., arbovirus) from the family *Flaviviridae* [1]. There are four dengue virus (DENV) serotypes with a potential fifth serotype described [2], all of them distributed in tropical and subtropical regions worldwide [3, 4] with emerging cases in northern latitudes [5, 6]. The pathogen is mainly transmitted by *Aedes aegypti* and *Ae. albopictus* mosquitoes [1, 7, 8]. Dengue disease symptoms range from asymptomatic, mild fever, rash, and joint pain (i.e., dengue with and without warning signs), to life threatening syndromes involving hemorrhagic fever and shock (i.e., severe dengue) [1]; severe clinical presentations are related with immunological cross-reactivity between dengue serotypes [9, 10]. As a viral infection, treatment is based mainly on support measures during the acute and critical phase of the infection [1, 11]. Although there has been progress on the development of vaccines, more research is needed before they are used as an effective public health mechanism for control [1, 12, 13].

The burden of dengue fever is high; around half of the world population is estimated to be at risk of infection [14] and every year, ~ 100 million symptomatic cases are detected [14, 15]. This poses a significant burden to the health systems in at least 128 countries worldwide [15] as well as economic impacts [16] that likely will expand to new regions in the future [3, 8, 17]. In Brazil, dengue was re-introduced in 1986 in the state of Roraima [18, 19] and quickly spread to the rest of the country [13]. In 2018, the total number of cases reported in Brazil was 265,934 [20] and so far 1,439,471 cases have been reported in 2019 through August [21].

As a notifiable infectious disease, any case of dengue detected in the Brazilian public health system must be reported to the corresponding health authorities [22]. Case notification relies on a passive surveillance framework in which disease reporting builds on patients seeking medical attention [23]. However, cases are often missed by the official reporting system because of non-severe presentations, lack of accessibility to health care infrastructure, misdiagnosis, or even misreporting. Thus, reported case counts are assumed to be an underestimation of the true disease burden [22]. Moreover, availability of health data based on traditional public health surveillance is usually constrained by time, bureaucracy, and staffing, with a lag of 2 weeks for the best systems [24], partial notifications in high burden settings [25], or even complete lack of reporting due to political instability as recently evidenced [26]. Timely disease reporting is critical for preparedness and executable real-time interventions to curb outbreaks [27].

As a consequence, the exploitation of Internet data as a source to characterize epidemiological patterns for communicable and non-communicable diseases has been promoted since the mid-90’s under the concept of digital epidemiology [28–31]. These efforts have focused on leveraging freely available information from Twitter, Google, Wikipedia, among others, to follow traces of disease patterns in the population [32–34]. Following the pioneering work of Eysenbach G. on using web-based search queries to track influenza [35] and other efforts that used Google-derived data for influenza in the United States [36, 37] and dengue in different countries [38, 39], Google developed Google Flu Trends (GFT) in 2009 and Google Dengue Trends (GDT) in 2011, as specific disease surveillance tools for digital epidemiology. However, a close examination of the predicting power of these algorithms, specifically GFT, showed signs of over and under prediction and low performance [40–43] cautioning against the broader implementation and applicability of these tools. As a consequence, both GFT and GDT were shut down in 2015 [44]. Nevertheless, two portals remained open to harvest search queries from Google, Google Trends (GT, https://trends.google.com/trends/?geo=US), and Google Health Trends (GHT). GT was released in 2006 as a free and publicly available source, whereas GHT, although free, it requires access through an application private interface (API, https://www.google.org/flutrends/about/). Many researchers have continued using Google-derived data to assess epidemiological patterns and inform epidemiological models for different pathogens with encouraging or conflicting results [45–51].

For Brazil, digital tools to quantify dengue reporting have been previously explored, in fact Chan et al. (2011) inspired the creation of GDT in the first place, showing a strong correlation with dengue cases in Brazil, among other countries [38]. Recently, Marques-Toledo et al. (2017) found that Twitter was useful in characterizing dengue incidence for different Brazilian cities [52], and the authors further compared their results against GT and Wikipedia query logs at the country level finding close agreement among several models [52]. Moreover, Yang et al. (2017) recently used an autoregressive model with Google search queries as exogenous variables (ARGO) to predict dengue cases in Brazil and showed good model performance at the country level [53]. Neither of these studies examined the ability of Google-derived data to characterize dengue incidence at the state level, and in fact, only few studies have examined Google-based algorithms at smaller political administrative levels [39, 50, 54]. To address this gap, we explore the ability of GHT to characterize weekly dengue cases from 2011 to 2016 in Brazil. For this goal, we used 19 dengue-related search terms for all 27 Brazilian states and quantified how Internet penetration data, demographic variables, and a standardized volatility index could determine a-priori where GHT might be a reliable tool.

## Methods

### Dengue incidence data

We obtained weekly dengue case counts for Brazil and all its 27 states from January 1st, 2011 to July 31st 2016. Data was given by the Brazilian Ministry of Health as a weekly aggregated data sheet with cases identified as counts without any identity information (e.g., names, gender, age, etc) [55]. This data encompasses the number of confirmed and suspected dengue cases reported by the official surveillance system, which follows specific guidelines of mandatory disease notification [22]. Incidence rates were calculated as the number of cases per week divided by the total population per state according to the official Brazilian population census of 2010 [56, 57]; for our analysis we used incidence instead of case counts to allow comparisons of dengue burden between Brazilian states [57].

### Google health trends data

The private API of GHT provides Internet search query data starting in 2004. Queries are sampled from the overall Google dataset in the form of a relative proportion, dividing the number of searches for a specified term over a particular time interval (i.e., days, weeks, months, or years) by the total number of term searches in that time, and multiplied by a predefined constant [58]. Thus, it differs from GT which provides a ranked score from 0 to 100 based on the highest frequency of searches in a particular period of time [58]. We obtained weekly GHT data for the same timeframe of dengue cases using 19 disease and mosquito vector related terms in Portuguese and English, including: “aedes”, “*Aedes aegypti*”, “aedes egípcio”, “aegypti”, dengue”, “dengue é vírus”, “dengue fever”, “dengue hemorrhagic fever”, “dengue sintomas”, “dengue vírus”, “DENV”, “DHF” (i.e., dengue hemorrhagic fever), “egípcio”, “mosquito”, “mosquito dengue”, “mosquitoes”, “novo vírus da dengue”, “sintomas da dengue”, and “vírus da dengue”. We downloaded the data on June 26th, 2017 and gathered information for the 27 Brazilian states and for the whole country.

### Statistical analysis

We fitted a linear regression model using GHT search terms as predictors of dengue incidence at the state level and recorded the adjusted R squared statistic as the primary outcome measure (0/1). Then, we fitted a multiple linear regression model using all the terms retrieving information by state (i.e., all terms model). Due to the potential overlap from conceptually related terms (e.g., “aedes” and “*Aedes aegypti*”), we also calculated Pearson’s correlation among terms and developed multiple linear regression models with those with a correlation less than 0.7 (i.e., uncorrelated terms model). Finally, we fitted models using four terms: “dengue”, “dengue sintomas”, “aedes”, and “mosquito”, which although correlated, have the potential to capture the full spectrum of searches considering the information they provide related to the disease and the mosquito vector (i.e., four terms model). We addressed the statistical differences between models with full vs. reduced number of terms, and between full and individual terms per state using pair-wise analysis of variance (ANOVA).

Given the heterogeneous Internet access throughout Brazil, we analyzed the role that accessibility could play in explaining our ability to track dengue incidence via GHT. Since 2005, Brazil has monitored the accessibility of their population to information and communication technologies [59, 60] through the “Survey for Internet Access, Television and Mobile Phone Possession for Personal Use (Acesso à Internet e a Televisão e Posse de Telefone Móvel Celular para Uso Pessoal, Portuguese)” [61]. The survey is part of the National Household Sample Survey (Pesquisa Nacional por Amostra de Domicílios (PNAD), Portuguese) conducted by the Brazilian Institute for Geography and Statistics (Instituto Brasileiro de Geografia e Estatística (IBGE), Portuguese [61]). We analyzed data from the 2015 survey, which included a sample of 356,904 individuals and 151,189 households distributed across the country and was subsequently extrapolated to a total of 177,657 million people and 68,037 million households [61, 62]. From all the available variables provided in this survey (~ 150), we used: (1) the total number of people above 10 years using Internet in the last 3 months, (2) the number of households that have used Internet in the last 3 months, (3) the number of people with mobile phones, and (4) the number of households with computers, as working predictors. In addition to these four variables, we used their logarithmic transformation considering their positive (i.e., right) skewed distribution, for a total of eight variables.

We explored all the available demographic variables from the 2010 official census provided by the IBGE [56]. The census includes information at the municipality level for multiple socio-economic factors including education, sanitation, income, etc., with a total of 237 potential predictors. We aggregated the information to the state level and examined them together with their logarithmic transformations as explained above for a total of 474 variables. Because they represent information with different magnitudes in the form of total counts, percentages, and rates, we normalized all the variables before the analysis. We examined each variable individually with a pair-wise univariate linear regression using the R squared for GHT against dengue cases as a dependent variable; we selected those variables with an adjusted R squared higher than 0.6, and performed a Pearson’s correlation among those selected to keep those with a score below 0.8. We then performed a multiple linear regression using the same dependent variable. We compared the ability of the selected variables to quantify GHT usefulness in Brazil using a principal component analysis (PCA) with the 474 predictors and selecting those components recovering more than 90% of the variance. The Pearson’s correlation statistic was also implemented in order to assess the relatedness between Internet and demographic variables.

Finally, to determine if variability in the signal of dengue incidence influences GHT accuracy, we computed a standardized volatility index for the dengue incidence data in each state. First, we normalized the dengue incidence time series, subtracting the mean and dividing it by its standard deviation. For this normalized time series, we averaged the absolute difference between each point in the data for each state—incidence per week in this case—to obtain a value representing the stability of dengue incidence (i.e., stable vs. unstable). All analyses were performed in R programming language [63] using standard packages for statistical analysis.

## Results

**Fig. 1:**
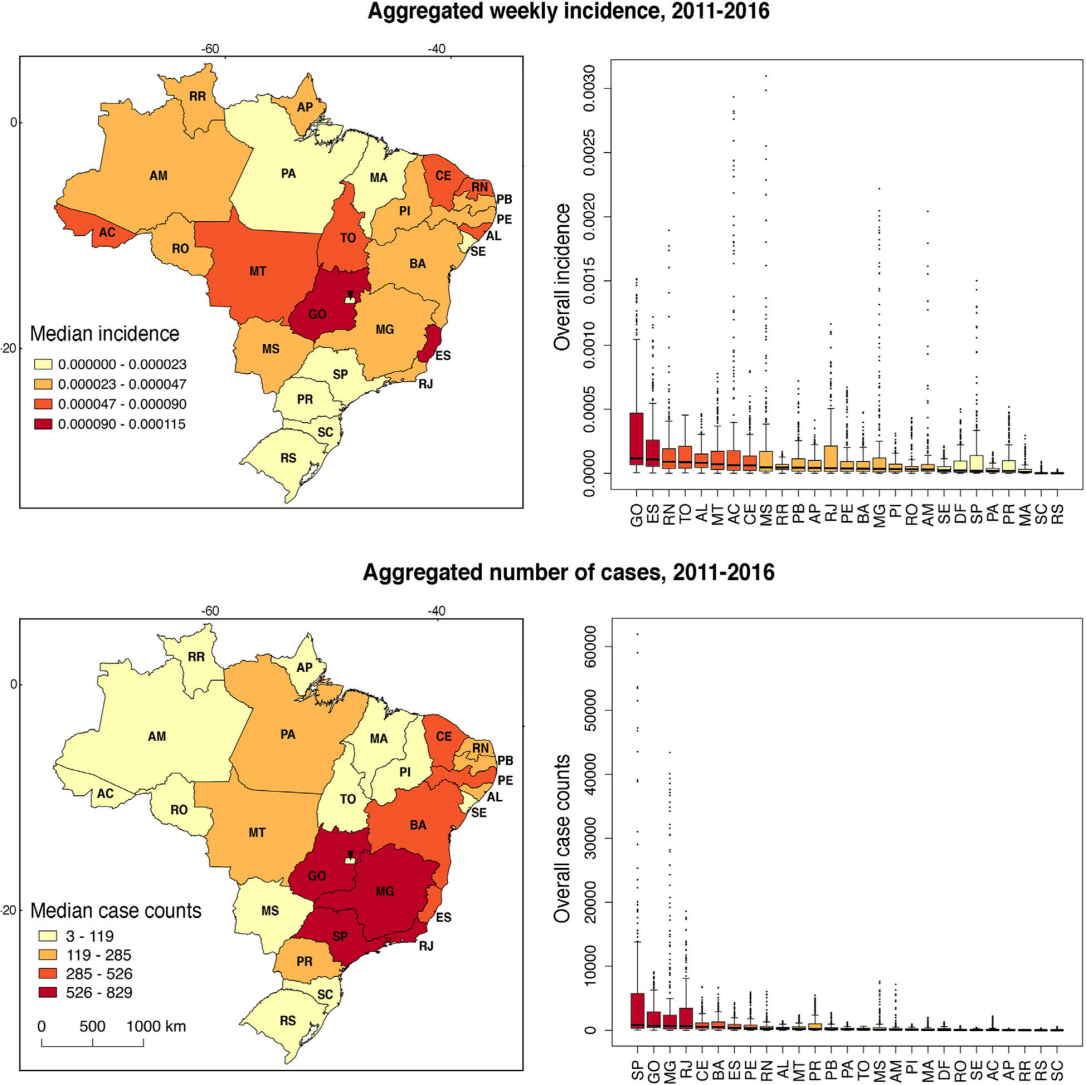
Dengue weekly incidence and case counts at the state level in Brazil aggregated across 2011–2016. States with low (yellow) and high (red) weekly incidence (top panel) and case counts (bottom panel) are depicted according to the median for the whole study period from 2011 to 2016. Boxplots (right panel) represent the variability of each state during the same time period. Maps were created with shape files from https://www.naturalearthdata.com/. Labels are the official abbreviations of Brazilian states: AC: Acré, AL: Alagoas, AP: Amapá, AM: Amazonas, BA: Bahia, CE: Ceará, DF (arrow): Distrito Federal, ES: Espírito Santo, GO: Goiás, MA: Maranhão, MT: Mato Grosso, MS: Mato Grosso do Sul, MG: Minas Gerais, PA: Pará, PB: Paraiba, PR: Paraná, PE: Pernambuco, PI: Piauí, RJ: Rio de Janeiro, RN: Rio Grande do Norte, RS: Rio Grande do Sul, RO: Rondônia, RR: Roraima, SC: Santa Catarina, SP: São Paulo, SE: Sergipe, TO: Tocantins

The Brazilian states with the overall highest yearly median number of dengue case counts from 2011 to 2016 are: São Paulo (829; min = 52; max = 61,944), Goiás (693; min = 29; max = 9094), Minas Gerais (687; min = 13; max = 43,424), and Rio de Janeiro (635; min = 12; max = 18,602), followed by Ceará (526; min = 5; max = 6754), Bahia (507; min = 12; max = 6654), Espírito Santo (382; min = 11; max = 4279), and Pernambuco (326; min = 19; max = 5881; Fig. 1 and Additional file 1: cases/incidences). States such as Rio Grande do Sul and Santa Catarina had a median of 4 and 3 cases, with a minimum of 0 cases and a maximum of 324 and 561 respectively, during the whole study period (Fig. 1 and Additional file 1). Conversely, when considering the weekly dengue incidence, there were two states with the overall highest yearly median values: Goiás (1.154x10e-4; min = 4.83x10e-6; max = 1.515x10e-3) and Espírito Santo (1.087x10e-4; min = 3.129x10e-6; max = 1.217x10e-3), followed by Rio Grande do Norte (8.996 × 10e-5; min = 0; max = 1.893x10e-3), Tocantins (8.638x10e-5; min = 3.614x10e-6; max = 4.539x10e-4), Alagoas (8.236x10e-5; min = 1.602x10e-6; max = 4.637x10e-4), Mato Grosso (7.034x10e-5; min = 6.59x10e-7; max = 7.762x10e-4), Acré (6.271x10e-5; min = 0; max = 2.931x10e-3), and Ceará (6.223x10e-5; min = 5.915x10e-7; max = 7.991x10e-4; Fig. 1); Santa Catarina and Rio Grande do Sul remained the states with the lowest median of weekly dengue incidence in the whole study period (4.801x10e-7 and 3.740x10e-7, respectively; Fig. 1 and Additional file 1).

From the 19 GHT terms, seven were useful in recovering information for all the Brazilian states, namely: “aedes”, “dengue”, “mosquito”, “aegypti”, “*Aedes aegypti*”, “sintomas da dengue”, and “dengue sintomas”. From the remaining 12 terms, five never provided information and therefore were discarded in further analysis (Table 1). Six terms tracked dengue for some but not all the states; for example, the word “mosquitoes” were valuable for Distrito Federal, Minas Gerais, and Paraná, but unimportant for Acré, Mato Grosso, or Santa Catarina. Two terms were informative in only one case: “dengue hemorrhagic fever” for São Paulo, and “DENV” for Brazil (Table 1).

**Table 1:** Availability of Google Health Trends to track dengue trends in Brazil and its states by term

	Terms	Number of terms	States
Informative terms	“aedes”, “dengue”, “mosquito”, “aegypti”, “*Aedes aegypti*”, “sintomas da dengue”, “dengue sintomas”	7	Brazil and all states
Uninformative terms	“aedes egípcio”, “egípcio”, “vírus da dengue”, “novo vírus da dengue”, “dengue é ‘virus”	5	Brazil and all states
Ambiguous terms	“dengue fever”	6	Brazil and BA, CE, ES, GO, MG, PB, PR, RJ, RS, SP (10 states)
	“dengue hemorrhagic fever”		SP (1 state)
	“DENV”		Brazil
	“DHF”		Brazil and BA, GO, MT, MG, PR, PR, PE, RJ, RS, SP (10 states)
	“dengue vírus”		Brazil and AM, BA, CE, DF, ES, GO, MA, MS, MG, PA, PB, PR, PE, RJ, RN, RS, RO, SC, SP (19 states)
	“mosquitoes”		Brazil and DF, MG, PR, RJ, RS, SP (6 states)

From the 19 terms explored, only seven were able to consistently track dengue incidence from Google Health Trends in Brazil (i.e., national level) and its individual states. Abbreviations of Brazilian states: AC: Acré, AL: Alagoas, AP: Amapá, AM: Amazonas, BA: Bahia, CE: Ceará, DF: Distrito Federal, ES: Espírito Santo, GO: Goiás, MA: Maranhão, MT: Mato Grosso, MS: Mato Grosso do Sul, MG: Minas Gerais, PA: Pará, PB: Paraiba, PR: Paraná, PE: Pernambuco, PI: Piauí, RJ: Rio de Janeiro, RN: Rio Grande do Norte, RS: Rio Grande do Sul, RO: Rondônia, RR: Roraima, SC: Santa Catarina, SP: São Paulo, SE: Sergipe, TO: Tocantins

**Fig. 2:**
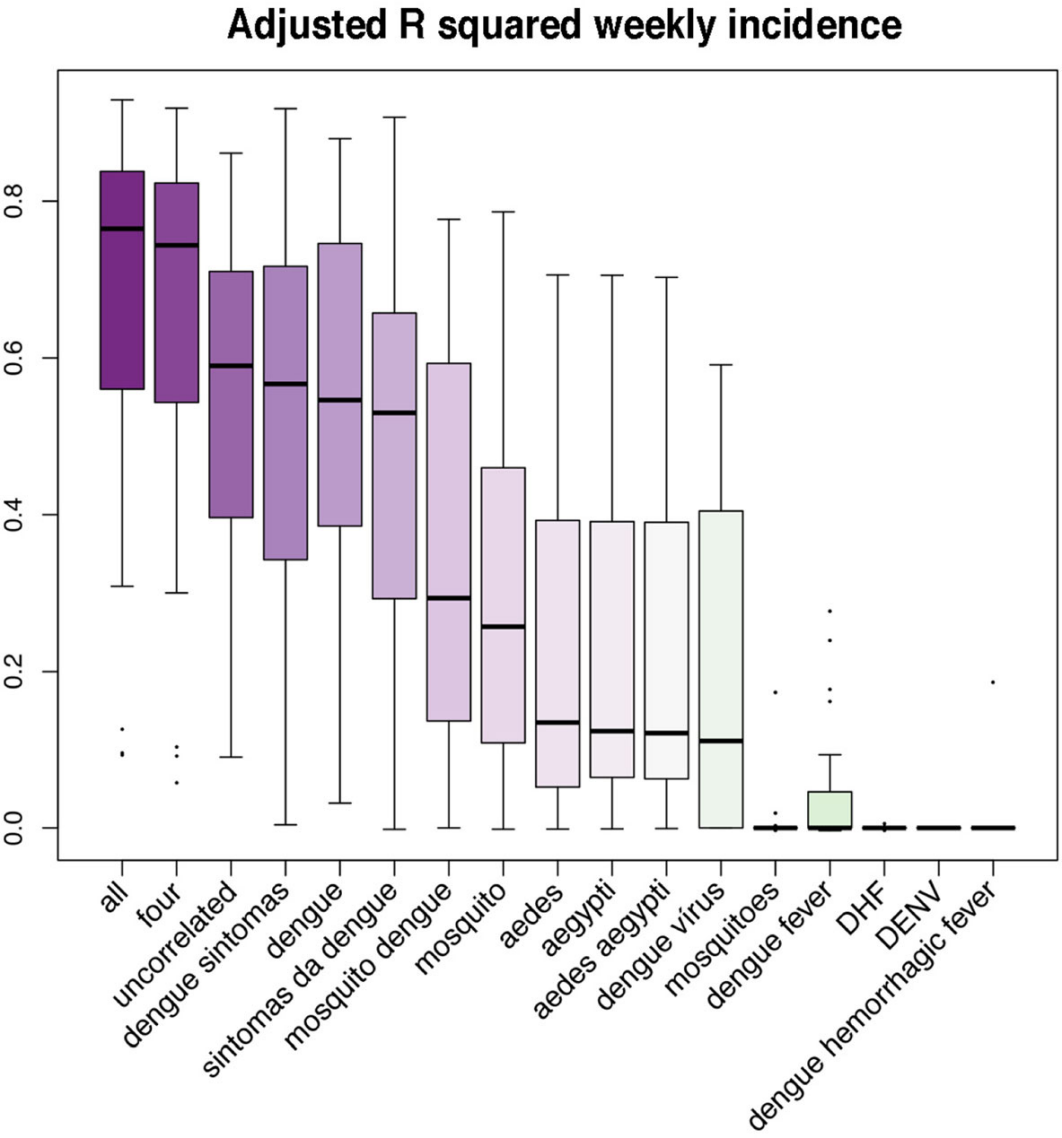
Adjusted R squared according to Google Health Trends search terms. All available terms per state and combined terms (i.e., all, four, and uncorrelated terms) were assessed in their ability to track weekly dengue incidences during 2011–2016. Combined terms showed the median highest adjusted R squared values (purple)

As expected, models developed with all the available terms per state fitted better to the weekly incidence dengue data than models developed with only four terms, uncorrelated terms, or with any of the individual terms when measuring the adjusted R squared statistic (Fig. 2, Table 2, and Additional file 2: adjusted R squared for individual terms). Individually, the most informative terms among those conceptually related with the disease included “dengue sintomas”, “dengue”, and “sintomas da dengue” (Fig. 2). Among the terms related with the vectors, “mosquito dengue” and “mosquito” were the most informative (Fig. 2). Correlated and uncorrelated terms for each state are shown in Additional file 3. We used a pair-wise ANOVA between models developed with all terms vs. models developed with reduced combination of terms (i.e., four terms and uncorrelated terms), and the individual terms for each state. From 328 comparisons—different number of terms were available for different states (Tables 1 and 2)—only in ten comparisons a reduced model was statistically comparable (i.e., not different, *F* statistic with a *p* > 0.05) to the model with all the terms, namely: the models based on four and uncorrelated terms and the model with the word “dengue” for Amapá, models developed with four terms for Distrito Federal, Maranhão, Pará, Rio Grande do Norte, Santa Catarina, and Sergipe, and models developed with uncorrelated terms for Roraima. Thus, for the subsequent analysis we used the adjusted R squared statistic from the models built using all the available terms in each state.

**Table 2:** Adjusted R squared for multiple linear models using all, uncorrelated, and four available terms

State	Adjusted R squared – All terms model (n)	Adjusted R squared – Uncorrelated terms model (n)	Adjusted R squared – Four terms model (***n*** = 4)
Acré (AC)	0.126 (7)	0.110 (6)	0.092
Alagoas (AL)	0.481 (8)	0.418 (3)	0.470
Amapá (AP)	0.096 (7)	0.099 (6)	0.103
Amazonas (AM)	**0.847 (9)**	0.590 (2)	**0.821**
Bahia (BA)	0.647 (11)	0.603 (3)	0.624
Ceará (CE)	**0.839 (10)**	0.746 (3)	**0.812**
Distrito Federal (DF)	**0.829 (10)**	0.433 (2)	**0.830**
Espírito Santo (ES)	0.725 (10)	0.537 (3)	0.688
Goiás (GO)	0.785 (11)	0.660 (3)	0.768
Maranhão (MA)	**0.859 (9)**	0.615 (2)	**0.856**
Mato Grosso (MT)	0.573 (9)	0.453 (3)	0.559
Mato Grosso do Sul (MS)	0.713 (9)	0.583 (3)	0.694
Minas Gerais (MG)	**0.923 (12)**	0.718 (4)	**0.919**
Pará (PA)	0.600 (9)	0.277 (2)	0.596
Paraiba (PB)	**0.837 (10)**	0.683 (3)	**0.832**
Paraná (PR)	**0.845 (12)**	0.741 (4)	**0.821**
Pernambuco (PE)	**0.819 (10)**	0.714 (2)	**0.808**
Piauí (PI)	0.553 (8)	0.377 (3)	0.542
Rio de Janeiro (RJ)	0.765 (12)	0.704 (5)	0.744
Rio Grande do Norte (RN)	**0.890 (9)**	0.706 (2)	**0.891**
Rio Grande do Sul (RS)	**0.804 (12)**	0.724 (4)	0.779
Rondônia (RO)	0.568 (9)	0.415 (4)	0.544
Roraima (RR)	0.093 (7)	0.091 (6)	0.058
Santa Catarina (SC)	**0.826 (9)**	0.788 (2)	**0.825**
São Paulo (SP)	**0.930 (13)**	**0.861 (6)**	**0.919**
Sergipe (SE)	0.308 (8)	0.216 (3)	0.300
Tocantins (TO)	0.415 (8)	0.342 (4)	0.401
Brazil	**0.888 (14)**	**0.852 (7)**	**0.850**

Different numbers of terms (n) were available depending on the state (e.g., São Paulo vs. Acré) and were correlated considering a state-by-state basis (e.g., Amapá vs. Maranhnão; Additional file 3: terms per state). Four terms model was developed with “dengue”, “dengue sintomas”, “aedes”, and “mosquito”. Bold represent adjusted R squared values above 0.8. Data for Brazil is shown in the last row

**Fig. 3:**
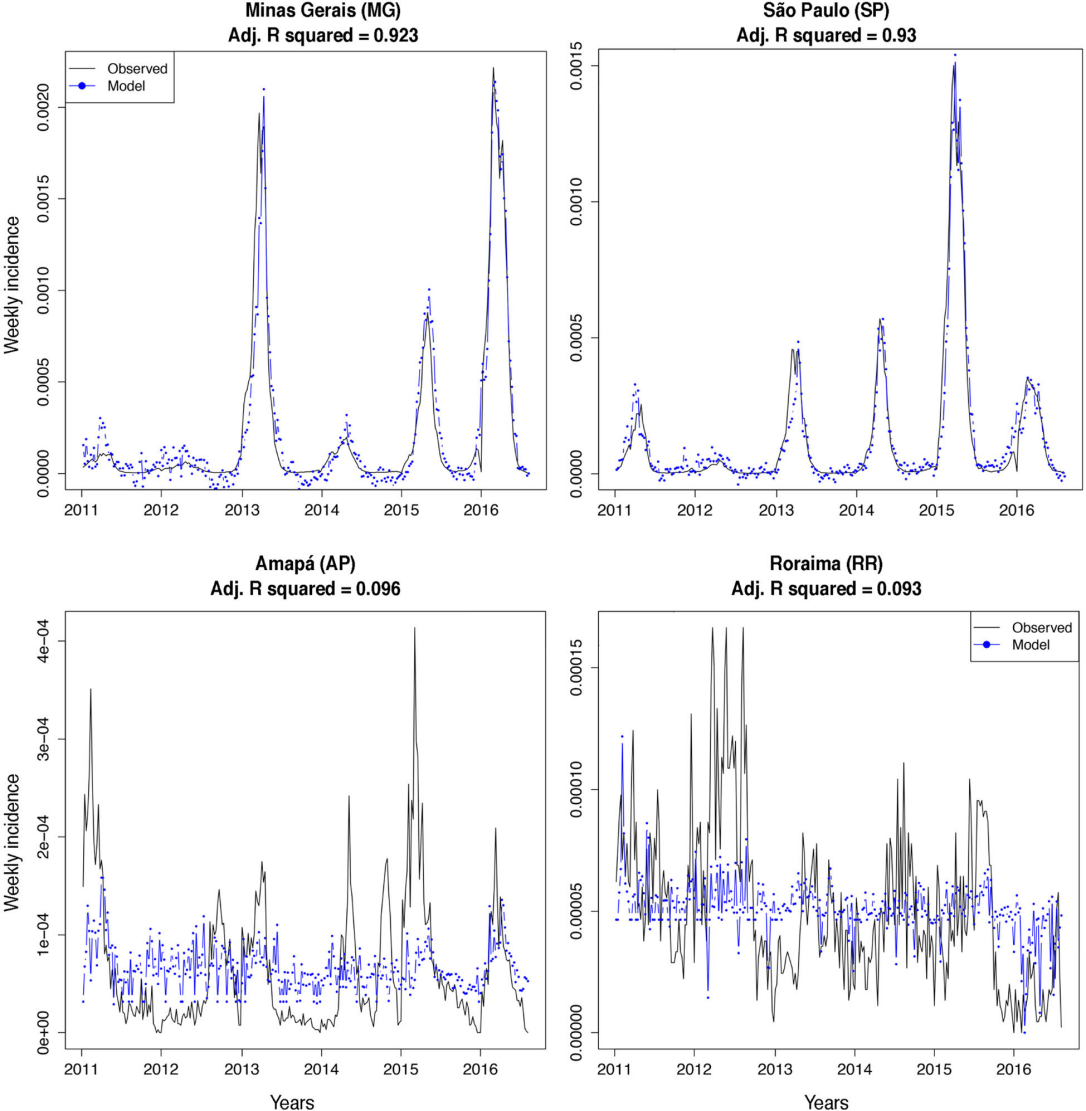
Google Health Trends and weekly dengue incidence in four Brazilian states, 2011–2016. Models built with all the terms available per state were useful for tracking dengue incidence during the study period for some (top panel) but not all (bottom panel) Brazilian states. The states that are not well predicted have noisier signals and lack strong seasonal dengue case counts, which may account for poor correlation with Google Health Trends in these regions. Lower access to Internet may also be a factor (Fig. 4)

As demonstrated previously, GHT fit the aggregated country-level dengue incidence well (All terms adjusted R squared = 0.888, Table 2 and Additional file 4: all plots for Brazil). For Distrito Federal, GHT data was only available starting November 24th, 2013; thus, for this case we performed all the analysis starting that date (Additional file 5: all plots for the 27 states). When analyzing each state separately, GHT was useful in some states but uninformative in others (Fig. 3). The highest adjusted R squared was for Minas Gerais (0.923) and São Paulo (0.930), while the worst fit was for Amapá (0.096) and Roraima (0.093; Fig. 3 and Table 2). Overall, by using all the terms combined, GHT was able to track weekly dengue incidences for 12 states with an adjusted R squared higher than 0.8, namely: Amazonas, Ceará, Distrito Federal, Maranhão, Minas Gerais, Paraiba, Pernambuco, Paraná, Rio Grande do Norte, Rio Grande do Sul, Santa Catarina, and São Paulo (Table 2). Considering an adjusted R squared value of 0.7, we can include four more states in this list: Espírito Santo, Goiás, Mato Grosso do Sul, and Rio de Janeiro, for a total of 16 states were GHT might be implemented for tracking dengue dynamics (Table 2).

**Fig. 4:**
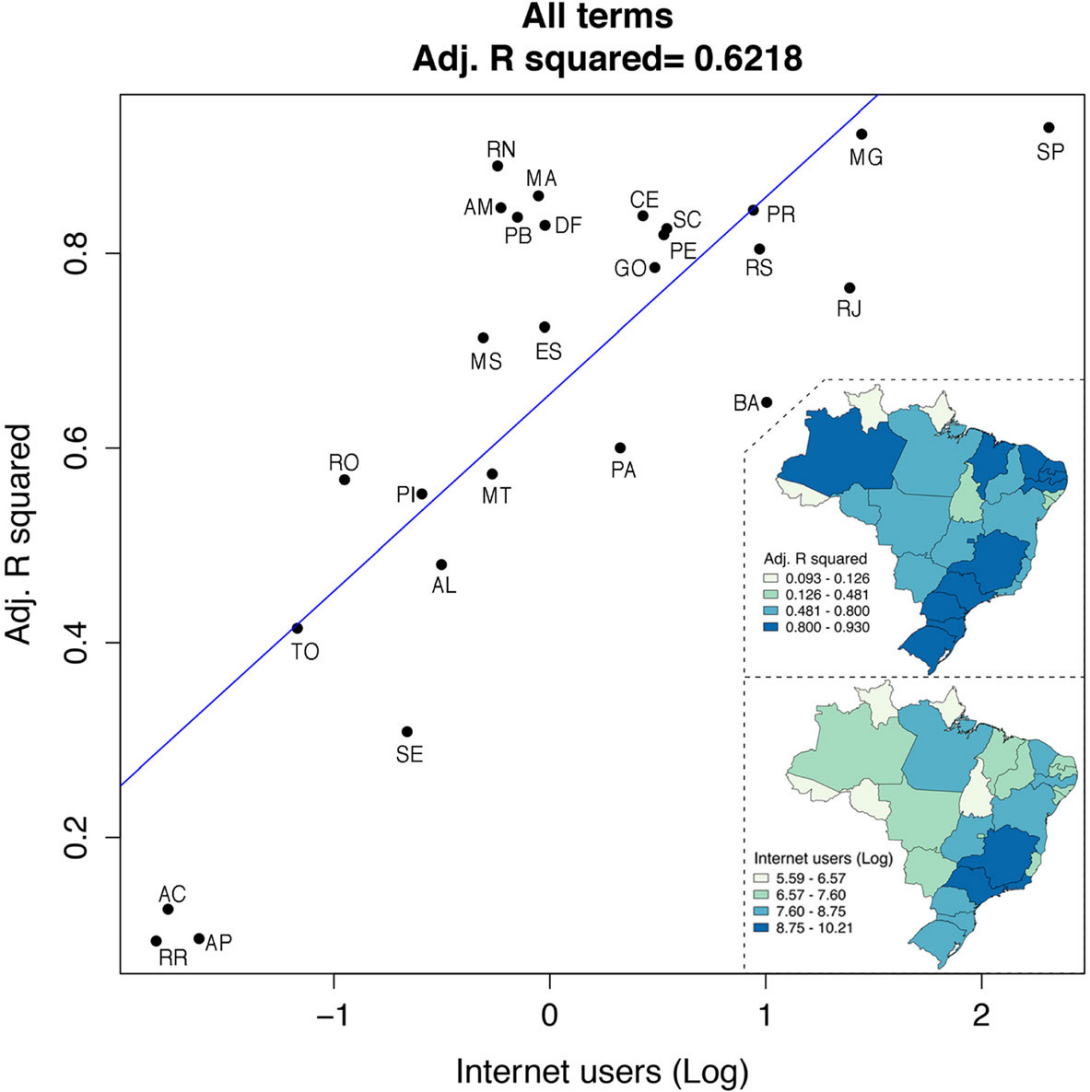
Logarithm of Internet users and Google Health Trends adjusted R squared. Maps (right panels) depict the adjusted R squared statistic per state when assessing Google Health Trends with their corresponding dengue incidence (linear regression plot). Twelve of 27 states showed values above 0.8 (top, dark blue). The logarithm of Internet users shows that the majority of the Brazilian states with high numbers of Internet users were concentrated at the southeast of Brazil (bottom, dark blue), but the remaining states show limited Internet penetration (pale green). Some states showed low number of Internet users but high GHT data fit (e.g., Amazonas, Maranhão, Paraiba, Distrito Federal). Maps were created with shape files from https://www.naturalearthdata.com/. Abbreviations of Brazilian states: AC: Acré, AL: Alagoas, AP: Amapá, AM: Amazonas, BA: Bahia, CE: Ceará, DF: Distrito Federal, ES: Espírito Santo, GO: Goiás, MA: Maranhão, MT: Mato Grosso, MS: Mato Grosso do Sul, MG: Minas Gerais, PA: Pará, PB: Paraiba, PR: Paraná, PE: Pernambuco, PI: Piauí, RJ: Rio de Janeiro, RN: Rio Grande do Norte, RS: Rio Grande do Sul, RO: Rondônia, RR: Roraima, SC: Santa Catarina, SP: São Paulo, SE: Sergipe, TO: Tocantins

All the Internet data variables were highly correlated with each other (Additional file 6: correlation plot). The logarithm of the number of Internet users per state partially explains when GHT will be able to track dengue incidence (all terms adjusted R squared = 0.621, Fig. 4). For instance, in the case of São Paulo and Minas Gerais, with the highest number of Internet users [61], the adjusted R squared for GHT and dengue incidence was high (Fig. 4, log scale); on the other hand, states such as Acré, Amapá, or Roraima with low numbers of Internet users [61] had a lower R squared value (Fig. 4, log scale). States such as Rio Grande do Norte, Amazonas, Paraiba, and Distrito Federal, had high GHT fit and a low number of Internet users, and viceversa for Rio de Janeiro and Bahia (Fig. 4).

**Fig. 5:**
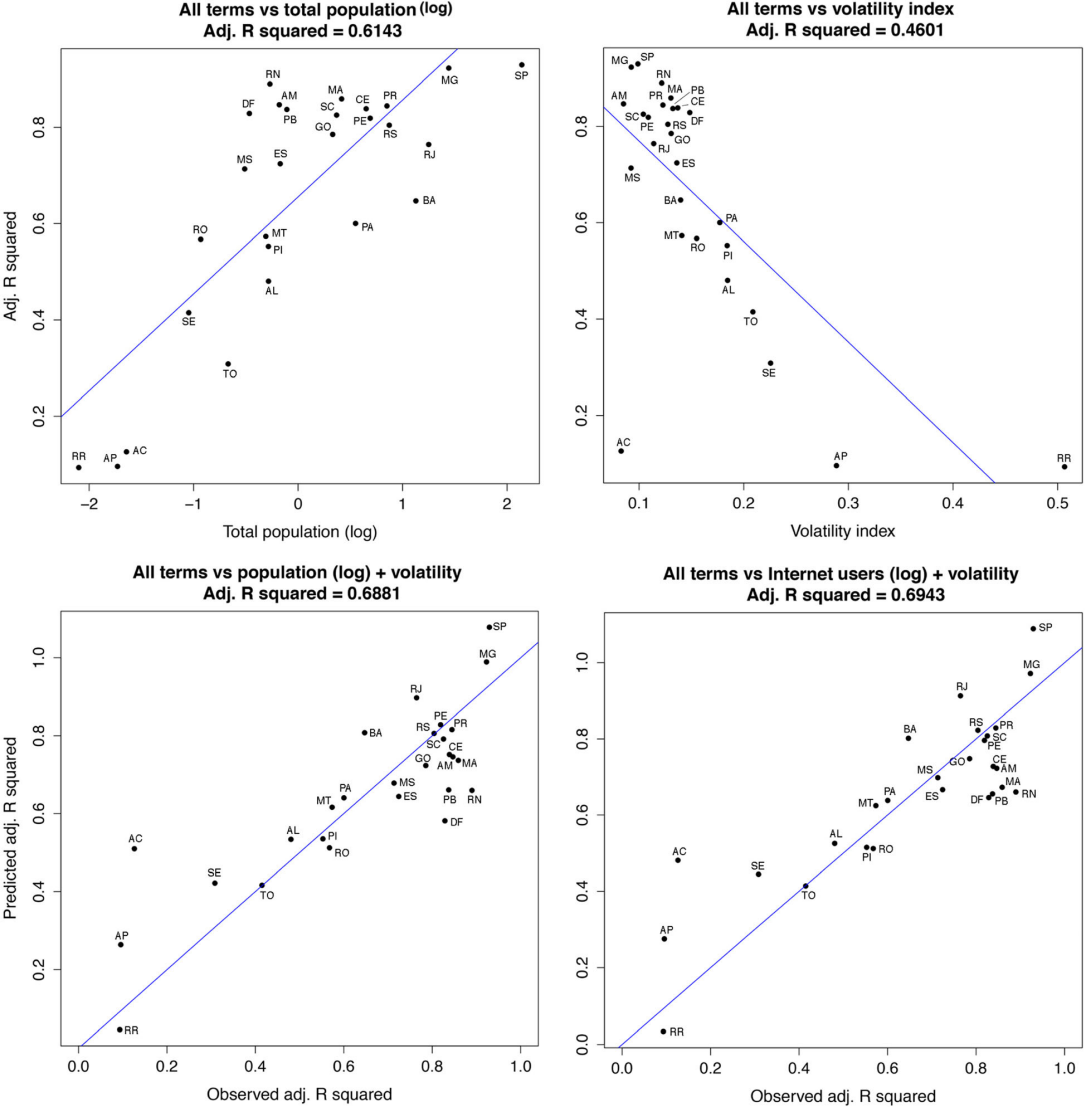
Total population and volatility index as predictors of Google Health Trends adjusted R squared. From 474 predictors, variables related with population where the most informative anticipating GHT behavior (top-left). The volatility index was useful detecting GHT accuracy although imprecise in some states such as Acré (top-right). Models considering the standardized volatility index and either the total population (bottom-left) or the number of Internet users (bottom-right) were the most informative anticipating GHT accuracy. Abbreviations of Brazilian states: AC: Acré, AL: Alagoas, AP: Amapá, AM: Amazonas, BA: Bahia, CE: Ceará, DF: Distrito Federal, ES: Espírito Santo, GO: Goiás, MA: Maranhão, MT: Mato Grosso, MS: Mato Grosso do Sul, MG: Minas Gerais, PA: Pará, PB: Paraiba, PR: Paraná, PE: Pernambuco, PI: Piauí, RJ: Rio de Janeiro, RN: Rio Grande do Norte, RS: Rio Grande do Sul, RO: Rondônia, RR: Roraima, SC: Santa Catarina, SP: São Paulo, SE: Sergipe, TO: Tocantins

From the 474 census demographic predictors, only 49 had an adjusted R squared higher than 0.6, all of which corresponded to variables related to the logarithmic transformation of the state population (see Additional file 7: 49 demographic variables). The 49 variables were highly correlated with each other (minimum *r* = 0.946); thus, a model including only the logarithm of the total population per state (Fig. 5 top-left panel) was similar to the one using the logarithm of the total number of Internet users (Fig. 4—adjusted R squared = 0.6218—vs. Fig. 5 top-left panel—adjusted R squared = 0.6143), a consequence of the high correlation between the predictors involved: population and number of Internet users (*r* = 0.98). Within the PCA framework, the first six PCs recovered 91% of the variance and a model developed with these components yielded similar results as the ones obtained using either the logarithm of the number of Internet users or the logarithm of total population per state (Adjusted R squared = 0.654; Additional file 8: PCA results).

States with the highest standardized volatility index, or high variability in reported dengue incidence, had less informative adjusted R squared when fitting GHT to incidence. States with smoother signals of dengue incidence, and therefore low volatility, had a better fit of GHT to incidence, resulting in adjusted R squared values above 0.8 (Fig. 4 and Fig. 5). However, even with a stable dengue incidence signal, GHT was not able to track dengue in Acré (Fig. 5 top-right panel). A model using the logarithm of the number of Internet users, the volatility index, and their interaction as predictors of GHT had the highest adjusted R squared among all the variables explored (= 0.694, Fig. 5 bottom-right panel). The same model with the logarithm of population instead of Internet users showed a similar adjusted R squared (= 0.688, Fig. 5 bottom-left panel). These models were comparable to those using the individual terms of the logarithm of total population, the number of Internet users, or the selected PCs (Figs. 4, 5, and Additional file 8). Eliminating Acré, the state with low volatility but low GHT accuracy (i.e., outlier), we saw an improvement in the models using the standardized volatility index plus its interactions with Internet users or total population (All terms vs. volatility index model, adjusted R squared = 0.717; all terms vs. Internet users (log) + standardized volatility index, adjusted R squared = 0.793; and all terms vs. population + standardized volatility index adjusted R squared = 0.809).

## Discussion

Digital surveillance systems have been shown to be useful for predicting country-wide dengue incidence in several countries [38, 53, 64]. Here, we evaluate the usefulness of GHT in tracking dengue incidence both at the country and the state level in Brazil, by evaluating GHT correlation with weekly dengue incidence data over 6 years. We have shown that the performance of GHT varies across states (Fig. 3, Table 2, and additional file 3); however, it is highly correlated with dengue incidence (adjusted R squared > 0.8) in 12 Brazilian states that are geographically dispersed. Moreover, we showed that proxies of Internet penetration such as the number of Internet users in the last 3 months only partially explain the usefulness of GHT (Fig. 4). In fact, the logarithm of the total population, from among 474 potential demographic predictors, allowed us to build a model that quantified GHT usefulness with similar accuracy to that of Internet penetration data (Fig. 5). The standardized volatility index in combination with the number of Internet users or the total population provided the highest accuracy when predicting GHT usefulness (Fig. 5).

Harvesting epidemiological information from Internet-data streams remains an active area of research for health purposes [31, 46, 49]. Despite its caveats [40], it has the potential to improve and complement traditional disease surveillance methods. In particular, they may be useful in timely outbreak detection and in settings where health surveillance is underdeveloped [31, 65]. However, before implementation, tools such as GHT should be explored at spatial resolutions smaller than countries [39, 50, 54]. Ours is one of the few studies addressing this gap showing that GHT usefulness will be heavily impacted by the political boundaries at which we wish to predict [52, 54]. In our case, GHT had an adjusted R squared score higher than 0.8 in 12 states, distributed among the five macro-regions of Brazil. The majority of states where GHT successfully track dengue incidence were located in the Southeast—Minas Gerais and São Paulo—, South—Paraná, Santa Catarina, Rio Grande do Sul—, and Northeast of the country—Ceará, Maranhão, Paraiba, Pernambuco, Rio Grande do Norte—, with the latter concentrating states with higher incidences (Figs. 1 and 4, Table 2). In general, these three macro-regions are considered the most economically developed of Brazil [66]. The Southeast and the South macro-regions have, in general, lower dengue incidence despite having the majority of cases (Fig. 1), highlighting the importance of normalizing measures to allow disease burden comparisons [57]. We calculated incidence rates based on the total population of each state to compare dengue burden, showing that high number of dengue cases in the Southeast are explained by the presence of high-density populations. Among the Southeast, Rio de Janeiro state might benefit from integrating GHT surveillance into dengue modeling and prediction efforts, considering an adjusted R squared of 0.765 (Fig. 1 and Fig. 4, Table 2). At the Northeast, Ceará and Rio Grande do Norte showed higher dengue incidence (= 6.223x10e-5 and 8.996x10e-5, respectively) with a high GHT fit to incidence data (adjusted R squared = 0.839 and 0.890, respectively) followed by Paraiba and Pernambuco (adjusted R squared = 0.837 and 0.819, respectively; Figs. 1 and 4, Table 2).

The remaining states where GHT was able to track dengue incidence were found in the Central-West—Distrito Federal—, and the North—Amazonas—macro-regions, with one state each (Fig. 1 and Fig. 4, Table 2). Both macro-regions represent the fourth and fifth economies of Brazil, respectively [66]. GHT for the Amazonas state could complement traditional clinical surveillance approaches (adjusted R squared = 0.847) considering its large area extent, moderate dengue incidence aggregated across all years (= 3.014x10e-5), and geographical location (Western portion of the North macro-region and far from the developed Brazilian Southeast; Fig. 1 and Additional file 1). The North macro-region also included the states of Acré, Amapá, Roraima, and Tocantins where GHT showed a poor behavior due to the low number of Internet users (Table 2 and Fig. 4). Both the North and Central-West areas include states with high dengue incidence consisting of Acré, Goiás, Mato Grosso, and Tocantins (Fig. 1, Additional file 1); among them, only Goiás showed a high GHT fit (adjusted R squared = 0.785); meanwhile the other states showed adjusted R squared values lower than 0.5 (Table 2 and Fig. 4).

Among the North and Northeast Brazilian macro-regions, Maranhão, Pará, and Sergipe have the lower dengue incidence (Fig. 1). For these states, only Maranhão showed a good GHT fit (adjusted R squared = 0.859). The state of Alagoas showed a moderate dengue incidence aggregated across all years (=8.236x10e-5; Fig. 1 and Additional file 1) but poor GHT behavior (adjusted R squared = 0.481) despite being surrounded by states with good GHT performance (e.g., adjusted R squared Pernambuco = 0.819 and Bahia = 0.647; Figs. 1 and 4, Table 2). We expected Alagoas to have similar dynamics as the rest of its surrounding states due to similar environmental and epidemiological trends, all limited by the Atlantic Ocean with a comparable area extent (Fig. 1). However, variability in local surveillance and mosquito control strategies might account for the differences [13, 22].

We suggest that multiple search terms should be explored when using GHT. In our case, only 7 from the initial 19 terms, retrieved information consistently in all the Brazilian states within our study period (Tables 1 and 2, Additional file 3), from the other terms, information was recovered only in specific instances (e.g., “dengue virus”, “dengue fever” for Bahia but not for Acré; Table 1). Models developed with all terms were statistically different from models developed with any of the subset term schemes (i.e., uncorrelated terms, four terms, individual terms) with the exception of ten comparisons. From them, Distrito Federal, Maranhão, Rio Grande do Norte, and Santa Catarina were states in which GHT adjusted R squared was higher than 0.8 (Fig. 4 and Table 2) and the four term model was not statistically different from the full term model (*p* > 0.05 in an ANOVA pair-wise comparison). Still, this was not the case for any of the other comparisons (i.e., 318), and more importantly, not for any of the terms individually. We recommend gathering GHT data using multiple terms in order to track dengue incidence dynamics at the state level in Brazil, potentially, this approach would be useful in other countries. While the approach for determining search terms vary, we posit that they should include both keywords (e.g., “dengue”) and conceptual words (e.g., “dengue sintomas”) [35] and should be selected according to the official and common languages of the country studied, Portuguese and English in our case; for instance, in Singapore searches using English terms were superior than searches using the Malay or Tami local languages [39].

Intuitively, the ability to determine if Internet data is going to be useful should depend on access to information and communication technologies. In this study, we found that the number of Internet users acts as a moderate predictor for GHT performance (Fig. 4). Similarly, Internet penetration has been demonstrated as a dubious variable for data derived from Google search engines [53, 54], and here, we showed that at least for states with both high and low Internet access (e.g., São Paulo, Minas Gerais vs. Acré, Roraima, Fig. 4), this variable can be regarded as a good predictor. However, for states such as Amazonas, Distrito Federal, Paraiba, and Rio Grande do Norte, only relying on the number of Internet users would have been an equivocal signal of GHT usefulness; thus, our data support a case-by-case investigation of Internet data.

Variables related to the total population per state were informative in determining GHT performance (Additional file 7: demographic variables) and these predictors were highly correlated with the total number of Internet users (*r* = 0.98). As Internet penetration increases worldwide [67], the total population per studied political unit, might be a reliable predictor to inform whether or not dengue surveillance based on digital epidemiology tools can complement traditional surveillance. However, other demographic predictors could also serve as indicators of GHT performance. We explored this possibility using various variable selection methods including Pearson’s correlation on the full 474 variables, a stepwise (i.e., forward and backward) multiple linear regression, and an elastic net regression with L1 normalization (i.e., Least Absolute Shrinkage and Selection Operator or Lasso) using leave-one-out cross validation fashion (Additional file 9: variable selection). Although a combination of five demographic variables allowed us to develop an improved prediction than total population alone (adjusted R squared = 0.670), how those variables may translate to other countries is not immediately clear (e.g., percentage of population that lives in urban households with garbage collecting services; Additional files 9 and 10: other demographic variables). In addition, models developed using multivariate linear regressions with different variable combinations never outperformed the model developed using PCA (Additional files 8 and 9), or those involving population and the standardized volatility index (Fig. 5, bottom-left panel).

The volatility index discussed here might also aid on addressing when to use GHT predictions for dengue (Fig. 5). In the context of detailed Internet access data, a model considering volatility and Internet users might be a straightforward exploration (Fig. 5). From a practical perspective, due to the potential lack of fine-resolution data on Internet accessibility in other countries, the total number of people plus the described standardized volatility index might be useful indicators of GHT performance. Finally, as has been discussed previously, we also explored if the total number of dengue cases would be a useful predictor to assess GHT performance [31, 54], but this predictor was less useful than any of the others explored (i.e., GHT for all terms vs. logarithm of total number of dengue cases, adjusted R squared = 0.274).

There are some limitations and caveats to our study. For the Distrito Federal state, GHT data was available for half of the study period, thus, we tested the GHT against weekly dengue incidence only for the corresponding timeframe, which involved ~ 3 years. The state of Distrito Federal corresponds to a small geographical region established as the capital of the country in 1960 (Fig. 1); we hypothesize that the lack of data was an artifact of Google’ algorithm missing the corresponding state before November 2013. Although we do not have any specific evidence of this, it is possibly related to what has been called “blue team dynamics” [40], that is, changes on the search engine can affect how and when GHT gathers data. In the same way, GHT retrieves information as a relative proportion of search volumes, as a consequence GHT data for the Brazilian states will not sum up to the same data at the country level and therefore GHT data for the whole country should be evaluated as an independent unit from its states [58]. This explains issues such as those presented in Table 1 where, for example, the term “DENV” was only available for the whole country and not for any particular state. Regardless, GHT’s relative proportion volume represents search patterns in an improved way than the ranked scores from GT. In the majority of studies using the latter, a transformation is needed in order to continue with the analysis [45], this step might add noise to prediction studies with GT.

In the present work, we did not split our data on training-testing datasets, which is a limitation of our approach. However, instead of dealing with prediction ability (i.e., forecasting [39, 49, 53, 54]), we based our evaluation on how GHT reflects dengue incidence in the Brazilian states and where it could inform and complement traditional epidemiological surveillance. Further, we did not include environmental variables known to alter dengue dynamics [54]. To implement GHT as complementary surveillance tool, other sources of uncertainties should be considered, among them, dengue information-seeking behavior might be confounded by the surge of other arbovirus-diseases such as chikungunya or Zika [68], or less well-known pathogens such as Mayaro, Oropouche, or West Nile viruses [69–71], all transmitted by different vectors from the order Diptera, which could trigger local web search behavior. This is especially true because of unspecific initial clinical presentations for those pathogens, media-induced panic or interest [72], and because health campaigns are often aimed towards vector control due to the lack of specific treatments [11]. Moreover, the GHT platform is unstable and could potentially be altered by any update or improvement on Google’s search algorithm itself [40] hindering replicability of these kind of studies [65].

Although GHT and other digital tools might be also capturing information from non-infected individuals, searching for the disease terms with different goals or induced by panic related media, it is likely also capturing a portion of the population that is missing medical care for multiple reasons. As has been shown for dengue surveillance in Brazil, the number of patients that are hospitalized and recorded as true “dengue” cases are more than ~ 50% the number of patients that are actually registered in the official epidemiological surveillance system [22], which is a broad phenomena described also for other countries [73]. Thus, an agreement between suspected dengue cases and GHT would indicate at least the presence of an outbreak.

Despite critics of models based on Google-derived data, several studies are demonstrating the potential usefulness of this approach for epidemiological research and how it can complement other forecasting models [49, 51, 53, 74]. Nonetheless, further studies face another problem: the availability of reliable health data, which is seldom shared in homogeneous user-friendly formats for representative timeframes [75, 76]. Only by assuring a constant supply of sound, consistent, and truly open access health reports, digital epidemiology could exploit the potential of big data considering the massive, but usually inaccessible, information from the public health domain [76].

## Conclusions

Digital epidemiology approaches based on GHT or other tools should be explored beyond country level to consider its actual ability to inform local public health departments. In the case of dengue in Brazil, 12 out of 27 states showed an adjusted R squared higher than 0.8, which suggests the potential ability of GHT to complement classical epidemiological surveillance, even though some states had low incidence during the 6-year study period (i.e., 2011–2016). Models developed with multiple terms were most informative than models using reduced sets or individual terms. Variables such as number of Internet users and total population per state are useful in determining where GHT could complement current surveillance strategies in several Brazilian states. Moreover, both variables benefit from the use of a standardized volatility index for selection of areas of GHT usefulness. The methods proposed here might be applied in other countries to test the ability of GHT to support dengue surveillance. Future studies might also explore the ability of GHT to track dengue using detailed measures of dengue transmission such as the force of infection, only confirmed cases, finer political boundaries (e.g., GHT data is available to particular cities in the world), or different temporal schemes.

## Supplementary information

**Additional file 1.** Total, median, minimum, and maximum numbers of dengue cases and incidence from the period 2011–2016.

**Additional file 2.** Adjusted R squared between Google Health Trends and weekly incidence of dengue for Brazil and all the 27 states for individual search terms.

**Additional file 3.** Available, correlated and uncorrelated terms, and adjusted R squared for multiple linear models with their corresponding correlation plots.

**Additional file 4.** Plots of multiple and simple linear models between Google Health Trends data and weekly dengue incidence for Brazil.

**Additional file 5.** Plots of multiple and simple linear models between Google Health Trends data and weekly dengue incidence for each of the 27 Brazilian states.

**Additional file 6.** Correlation plot of the Internet access variables selected in the study.

**Additional file 7.** Definition of 49 demographic variables showing an adjusted R squared higher than 0.6 on a pair-wise linear regression models.

**Additional file 8.** Results of the principal component analysis of 474 predictors.

**Additional file 9.** Variables selected by applying different variable selection routines and definitions of each of the selected predictors.

**Additional file 10.** Description of variables selected according to different methods.

## Data Availability

Datasets analyzed in the present study are available from the corresponding author on reasonable request.

## Abbreviations

API: Application private interface
DHF: Dengue hemorrhagic fever
DENV: Dengue virus
GDT: Google Dengue Trends
GFT: Google Flu Trends
GHT: Google Health Trends
GT: Google Trends
IBGE: Instituto Brasileiro de Geografia e Estatística (Brazilian Institute for Geography and Statistics, English)
PNAD: Pesquisa Nacional por Amostra de Domicílios (National Household Sample Survey, English)

## Brazilian states

AC: Acré
AL: Alagoas
AP: Amapá
AM: Amazonas
BA: Bahia
CE: Ceará
DF: Distrito Federal
ES: Espírito Santo
GO: Goiás
MA: Maranhão
MT: Mato Grosso
MS: Mato Grosso do Sul
MG: Minas Gerais
PA: Pará
PB: Paraiba
PR: Paraná
PE: Pernambuco
PI: Piauí
RJ: Rio de Janeiro
RN: Rio Grande do Norte
RS: Rio Grande do Sul
RO: Rondônia
RR: Roraima
SC: Santa Catarina
SP: São Paulo
SE: Sergipe
TO: Tocantins
