# Supplementary material for: Google Health Trends performance reflecting dengue incidence for the Brazilian states
Source: BMC Infect Dis. 2020 Mar 26;20:252. doi: 10.1186/s12879-020-04957-0 (PMC7104526; doi:10.1186/s12879-020-04957-0)
Supplement: Supplementary file 1 — Additional file 1. Total, median, minimum, and maximum numbers of dengue cases and incidence from the period 2011–2016. [file 12879_2020_4957_MOESM1_ESM.docx]

**Google Health Trends performance reflecting dengue incidence for the Brazilian states**

**Authors:** Daniel Romero-Alvarez, Nidhi Parikh, Dave Osthus, Kaitlyn Martinez, Nicholas Generous, Sara del Valle, Carrie A. Manore

**Additional file 1. Total, median, minimum, and maximum numbers of dengue cases and incidence for the period 2011–2016.** Minimum and maximum are specifying the number of dengue cases and incidence with the year where the minimum/maximum record was collected in parenthesis. Incidence was calculated as the total number of cases divided by the total population per state according to the official 2010 Brazilian census. Data for the whole country can be found in the last row.

| **State** | **Total number of cases (2011–2016)** | **Median number of cases (2011–2016)** | **Minimum number of cases (year)** | **Maximum number of cases (year)** | **Median incidence (2011–2016)** | **Minimum incidence (year)** | **Maximum incidence (year)** |
| --- | --- | --- | --- | --- | --- | --- | --- |
| Acré (AC) | 60216 | 46 | 0 (2016) | 2150 (2011) | 6.271x10e-5 | 0 (2016) | 2.931x10e-3 (2011) |
| Alagoas (AL) | 101381 | 257 | 5 (2016) | 1447 (2012) | 8.236 x10e-5 | 1.602x10e-6 (2016) | 4.637x10e-4 (2012) |
| Amapá (AP) | 12882 | 28 | 0 (2016) | 277 (2015) | 4.182 x10e-5 | 0 (2011) | 4.137 x10e-4 (2015) |
| Amazonas (AM) | 103576 | 105 | 4 (2011) | 7110 (2011) | 3.014 x10e-5 | 1.148 x10e-4 (2011) | 2.041 x10e-3 (2011) |
| Bahia (BA) | 278193 | 507 | 12 (2016) | 6654 (2016) | 3.617 x10e-5 | 8.561 x10e-7 (2016) | 4.747 x10e-4 (2016) |
| Ceará (CE) | 281387 | 526 | 5 (2016) | 6754 (2012) | 6.223 x10e-5 | 5.915 x10e-7 (2016) | 7.991 x10e-4 (2012) |
| Distrito Federal (DF) | 55093 | 56 | 3 (2016) | 1282 (2016) | 2.179 x10e-5 | 1.167 x10e-6 (2016) | 4.988 x10e-4 (2016) |
| Espírito Santo (ES) | 213107 | 382 | 11 (2016) | 4279 (2013) | 1.087 x10e-5 | 3.129 x10e-6 (2016) | 1.217 x10e-3 (2013) |
| Goiás (GO) | 548555 | 693 | 29 (2016) | 9094 (2013) | 1.154 x10e-4 | 4.830 x10e-6 (2016) | 1.515 x10e-3 (2013) |
| Maranhão (MA) | 51454 | 69 | 0 (2016) | 1943 (2016) | 1.057 x10e-5 | 0 (2016) | 2.955 x10e-4 (2016) |
| Mato Grosso (MT) | 119721 | 213 | 2 (2016) | 2356 (2013) | 7.034 x10e-5 | 6.590 x10e-7 (2016) | 7.762 x10e-4 (2013) |
| Mato Grosso do Sul (MS) | 161659 | 114 | 7 (2016) | 7588 (2013) | 4.675 x10e-5 | 2.858 x10e-6 (2016) | 3.098 x10e-3 (2013) |
| Minas Gerais (MG) | 1257316 | 687 | 13 (2016) | 43424 (2016) | 3.506 x10e-5 | 6.634 x10e-7 (2016) | 2.216 x10e-3 (2016) |
| Pará (PA) | 68213 | 151 | 4 (2016) | 1245 (2011) | 1.992 x10e-5 | 5.276 x10e-7 (2016) | 1.642 x10e-4 (2011) |
| Paraiba (PB) | 97769 | 165 | 0 (2016) | 2706 (2016) | 4.394 x10e-5 | 0 (2016) | 7.184 x10e-4 (2016) |
| Paraná (PR) | 244036 | 188 | 4 (2012) | 5421 (2013) | 1.999 x10e-5 | 3.830 x10e-7 (2012) | 5.190 x10e-4 (2013) |
| Pernambuco (PE) | 239664 | 326 | 19 (2012) | 5881 (2016) | 3.712 x10e-5 | 2.160 x10e-6 (2012) | 6.686 x10e-4 (2016) |
| Piauí (PI) | 47363 | 104 | 0 (2016) | 966 (2012) | 3.335 x10e-5 | 0 (2016) | 3.098 x10e-4 (2012) |
| Rio de Janeiro (RJ) | 710920 | 635 | 12 (2016) | 18602 (2013) | 3.974 x10e-5 | 7.505 x10e-7 (2016) | 1.163 x10e-3 (2013) |
| Rio Grande do Norte (RN) | 159613 | 285 | 0 (2011) | 5998 (2016) | 8.996 x10e-5 | 0 (2011) | 1.893 x10e-3 (2016) |
| Rio Grande do Sul (RS) | 6168 | 4 | 0 (2011) | 324 (2016) | 3.740 x10e-7 | 0 (2011) | 3.030 x10e-5 (2016) |
| Rondônia (RO) | 27310 | 49 | 0 (2016) | 675 (2013) | 3.168 x10e-5 | 0 (2016) | 4.320 x10e-4 (2013) |
| Roraima (RR) | 6719 | 20 | 0 (2016) | 75 (2012) | 4.439 x10e-5 | 0 (2016) | 1.665 x10e-4 (2012) |
| Santa Catarina (SC) | 10523 | 3 | 0 (2011) | 561 (2016) | 4.801 x10e-7 | 0 (2011) | 8.978 x10e-5 (2016) |
| São Paulo (SP) | 1527136 | 829 | 52 (2012) | 61944 (2015) | 2.010 x10e-5 | 1.260 x10e-6 (2012) | 1.501 x10e-3 (2015) |
| Sergipe (SE) | 23793 | 48 | 1 (2012) | 436 (2015) | 2.321 x10e-5 | 4.836 x10e-7 (2012) | 2.108 x10e-4 (2015) |
| Tocantins (TO) | 52264 | 119 | 5 (2016) | 628 (2016) | 8.638 x10e-5 | 3.614 x10e-6 (2016) | 4.539 x10e-4 (2016) |
| Brazil (BR) | 6466031 | 9300 | 465 (2016) | 107041 (2016) | 4.875 x10e-5 | 2.438 x10e-6 (2016) | 5.611 x10e-4 (2016) |
