# Supplementary material for: Google Health Trends performance reflecting dengue incidence for the Brazilian states
Source: BMC Infect Dis. 2020 Mar 26;20:252. doi: 10.1186/s12879-020-04957-0 (PMC7104526; doi:10.1186/s12879-020-04957-0)
Supplement: Supplementary file 2 — Additional file 2. Adjusted R squared between Google Health Trends and weekly incidence of dengue for Brazil and all the 27 states for individual search terms. [file 12879_2020_4957_MOESM2_ESM.docx]

**Google Health Trends performance reflecting dengue incidence for the Brazilian states**

**Authors:** Daniel Romero-Alvarez, Nidhi Parikh, Dave Osthus, Kaitlyn Martinez, Nicholas Generous, Sara del Valle, Carrie A. Manore

**Additional file 2**. Adjusted R squared between Google Health Trends and weekly incidence of dengue for Brazil and all its 27 states considering individual search terms. Brazilian states: AC: Acré, AL: Alagoas, AP: Amapá, AM: Amazonas, BA: Bahia, CE: Ceará, DF (arrow): Distrito Federal, ES: Espírito Santo, GO: Goiás, MA: Maranhão, MT: Mato Grosso, MS: Mato Grosso do Sul, MG: Minas Gerais, PA: Pará, PB: Paraiba, PR: Paraná, PE: Pernambuco, PI: Piauí, RJ: Rio de Janeiro, RN: Rio Grande do Norte, RS: Rio Grande do Sul, RO: Rondônia, RR: Roraima, SC: Santa Catarina, SP: São Paulo, SE: Sergipe, TO: Tocantins.

| **Political level** | **aedes** | **aedes aegypti** | **aegypti** | **dengue** | **dengue fever** | **dengue hemorrhagic fever** | **dengue sintomas** | **dengue virus** |
| --- | --- | --- | --- | --- | --- | --- | --- | --- |
| Brazil | 0.288 | 0.289 | 0.289 | 0.789 | 0.271 | 0.102 | 0.838 | 0.367 |
| AC | -0.002 | 0.002 | 0.000 | 0.081 | 0.000 | 0.000 | 0.023 | 0.000 |
| AL | 0.111 | 0.121 | 0.124 | 0.360 | 0.000 | 0.000 | 0.388 | 0.000 |
| AP | 0.000 | -0.001 | -0.001 | 0.100 | 0.000 | 0.000 | 0.004 | 0.000 |
| AM | 0.010 | 0.001 | 0.003 | 0.646 | 0.000 | 0.000 | 0.562 | 0.027 |
| BA | 0.330 | 0.327 | 0.325 | 0.566 | 0.063 | 0.000 | 0.593 | 0.353 |
| CE | 0.018 | 0.012 | 0.012 | 0.538 | 0.029 | 0.000 | 0.681 | 0.108 |
| DF | 0.455 | 0.453 | 0.456 | 0.740 | 0.000 | 0.000 | 0.792 | 0.418 |
| ES | 0.176 | 0.133 | 0.135 | 0.546 | 0.078 | 0.000 | 0.567 | 0.202 |
| GO | 0.135 | 0.129 | 0.128 | 0.605 | 0.094 | 0.000 | 0.652 | 0.197 |
| MA | 0.703 | 0.702 | 0.705 | 0.831 | 0.000 | 0.000 | 0.496 | 0.462 |
| MT | 0.096 | 0.091 | 0.089 | 0.464 | 0.000 | 0.000 | 0.490 | 0.000 |
| MS | 0.104 | 0.100 | 0.099 | 0.542 | 0.000 | 0.000 | 0.596 | 0.111 |
| MG | 0.551 | 0.555 | 0.556 | 0.880 | 0.239 | 0.000 | 0.918 | 0.591 |
| PA | 0.086 | 0.079 | 0.080 | 0.411 | 0.000 | 0.000 | 0.243 | 0.088 |
| PB | 0.629 | 0.633 | 0.634 | 0.811 | -0.003 | 0.000 | 0.720 | 0.535 |
| PR | 0.326 | 0.326 | 0.326 | 0.698 | 0.161 | 0.000 | 0.713 | 0.359 |
| PE | 0.240 | 0.229 | 0.227 | 0.623 | 0.000 | 0.000 | 0.712 | 0.391 |
| PI | 0.007 | 0.005 | 0.005 | 0.269 | 0.000 | 0.000 | 0.316 | 0.000 |
| RJ | 0.008 | 0.007 | 0.007 | 0.444 | 0.277 | 0.000 | 0.550 | 0.037 |
| RN | 0.706 | 0.685 | 0.690 | 0.869 | 0.000 | 0.000 | 0.754 | 0.425 |
| RS | 0.537 | 0.561 | 0.562 | 0.751 | -0.003 | 0.000 | 0.729 | 0.558 |
| RO | 0.211 | 0.245 | 0.246 | 0.479 | 0.000 | 0.000 | 0.369 | 0.128 |
| RR | 0.007 | 0.047 | 0.050 | 0.032 | 0.000 | 0.000 | 0.015 | 0.000 |
| SC | 0.564 | 0.559 | 0.558 | 0.803 | 0.000 | 0.000 | 0.811 | 0.538 |
| SP | 0.090 | 0.092 | 0.092 | 0.844 | 0.177 | 0.186 | 0.861 | 0.104 |
| SE | 0.126 | 0.113 | 0.122 | 0.288 | 0.000 | 0.000 | 0.151 | 0.000 |
| TO | 0.181 | 0.113 | 0.114 | 0.313 | 0.000 | 0.000 | 0.303 | 0.000 |

| **Political level** | **DENV** | **DHF** | **mosquito** | **mosquito dengue** | **mosquitoes** | **sintomas da dengue** |
| --- | --- | --- | --- | --- | --- | --- |
| Brazil | 0.008 | 0.003 | 0.488 | 0.607 | 0.059 | 0.820 |
| AC | 0.000 | 0.000 | 0.037 | 0.000 | 0.000 | -0.002 |
| AL | 0.000 | 0.000 | 0.169 | 0.146 | 0.000 | 0.307 |
| AP | 0.000 | 0.000 | 0.036 | 0.000 | 0.000 | 0.009 |
| AM | 0.000 | 0.000 | 0.102 | 0.293 | 0.000 | 0.527 |
| BA | 0.000 | 0.001 | 0.412 | 0.427 | 0.000 | 0.550 |
| CE | 0.000 | 0.000 | 0.070 | 0.096 | 0.000 | 0.618 |
| DF | 0.000 | 0.000 | 0.472 | 0.513 | 0.173 | 0.761 |
| ES | 0.000 | 0.000 | 0.219 | 0.267 | 0.000 | 0.512 |
| GO | 0.000 | 0.001 | 0.273 | 0.340 | 0.000 | 0.640 |
| MA | 0.000 | 0.000 | 0.786 | 0.690 | 0.000 | 0.440 |
| MT | 0.000 | 0.000 | 0.155 | 0.134 | 0.000 | 0.449 |
| MS | 0.000 | 0.000 | 0.257 | 0.338 | 0.000 | 0.556 |
| MG | 0.000 | -0.003 | 0.705 | 0.761 | -0.003 | 0.907 |
| PA | 0.000 | 0.000 | 0.115 | 0.180 | 0.000 | 0.278 |
| PB | 0.000 | 0.000 | 0.702 | 0.679 | 0.000 | 0.571 |
| PR | 0.000 | -0.002 | 0.447 | 0.535 | 0.003 | 0.719 |
| PE | 0.000 | -0.003 | 0.389 | 0.372 | 0.000 | 0.675 |
| PI | 0.000 | 0.000 | 0.017 | 0.029 | 0.000 | 0.260 |
| RJ | 0.000 | 0.005 | 0.036 | 0.125 | 0.019 | 0.530 |
| RN | 0.000 | 0.000 | 0.729 | 0.777 | 0.000 | 0.631 |
| RS | 0.000 | -0.003 | 0.574 | 0.651 | -0.003 | 0.710 |
| RO | 0.000 | 0.000 | 0.269 | 0.191 | 0.000 | 0.311 |
| RR | 0.000 | 0.000 | -0.002 | 0.000 | 0.000 | 0.001 |
| SC | 0.000 | 0.000 | 0.364 | 0.733 | 0.000 | 0.788 |
| SP | 0.000 | 0.001 | 0.551 | 0.719 | -0.001 | 0.840 |
| SE | 0.000 | 0.000 | 0.154 | 0.139 | 0.000 | 0.060 |
| TO | 0.000 | 0.000 | 0.168 | 0.150 | 0.000 | 0.184 |
