# Supplementary material for: Google Health Trends performance reflecting dengue incidence for the Brazilian states
Source: BMC Infect Dis. 2020 Mar 26;20:252. doi: 10.1186/s12879-020-04957-0 (PMC7104526; doi:10.1186/s12879-020-04957-0)
Supplement: Supplementary file 3 — Additional file 3. Available, correlated and uncorrelated terms, and adjusted R squared for multiple linear models with their corresponding correlation plots. [file 12879_2020_4957_MOESM3_ESM.docx]

**Google Health Trends performance reflecting dengue incidence for the Brazilian states**

**Authors:** Daniel Romero-Alvarez, Nidhi Parikh, Dave Osthus, Kaitlyn Martinez, Nicholas Generous, Sara del Valle, Carrie A. Manore

**Additional file 3. Available, correlated and uncorrelated terms, and adjusted R squared for multiple linear models.** Due to Google Health Trends (GHT) behavior while retrieving information, different numbers of terms were available depending on each Brazilian state. Uncorrelated terms were selected using a threshold of 0.7 in a Pearson correlation test considering all the available terms per state. Models developed with four terms used the words “dengue”, “dengue sintomas”, “aedes”, and “mosquito”. Brazil is shown in the last row. Correlation plots for the states and their available terms can be found after the table.

| **State** | **Terms with information** | **Adj. R^2^ all terms** | **Correlated terms (n)** | **Uncorrelated terms (n)** | **Adj. R^2^ uncorrelated terms** | **Adj. R^2^ four terms** |
| --- | --- | --- | --- | --- | --- | --- |
| Acré (AC) | 7 | 0.126 | “aedes aegypti” (1) | “aedes”, “aegypti”, “dengue”, “dengue sintomas” “mosquito”, “sintomas da dengue” (6) | 0.11 | 0.092 |
| Alagoas (AL) | 8 | 0.481 | “aedes”, “aegypti”, “aedes aegypti”, “dengue”, “mosquito”, (5) | “mosquito dengue”, “sintomas da dengue”, “dengue sintomas” (3) | 0.418 | 0.47 |
| Amazonas (AM) | 9 | 0.847 | “aedes”,  “aegypti”,  “dengue”,  “dengue sintomas”,  “dengue virus”,  “mosquito”,  “mosquito dengue” (7) | “aedes aegypti”, “sintomas da dengue” (2) | 0.59 | 0.821 |
| Amapá (AP) | 7 | 0.096 | “aedes aegypti” (1) | “aedes”, “aegypti”,  “dengue”,  “dengue sintomas”,  “dengue virus”,  “mosquito”,  “mosquito dengue” (6) | 0.099 | 0.103 |
| Bahia (BA) | 11 | 0.647 | “aedes”,  “aedes aegypti”,  “aegypti”,  “dengue”,  “dengue virus”, “mosquito”,  “mosquito dengue”,  “sintomas da dengue” (8) | “dengue fever”, “dengue sintomas”  “DHF” (3) | 0.603 | 0.624 |
| Ceará (CE) | 10 | 0.839 | “aedes”,  “aedes aegypti”,  “dengue”,  “dengue virus”,  “mosquito”,  “mosquito dengue”,  “sintomas da dengue” (7) | “aegypti”, “dengue fever”, “dengue sintomas” (3) | 0.746 | 0.812 |
| Distrito Federal (DF) | 10 | 0.829 | “aedes”, “aedes aegypti”,  “aegypti”,  “dengue”,  “dengue sintomas”,  “mosquito”,  “mosquito dengue”,  “sintomas da dengue” (8) | “dengue virus”, “mosquitoes” (2) | 0.433 | 0.83 |
| Espírito Santo (ES) | 10 | 0.725 | “aedes”,  “aedes aegypti”,  “aegypti”,  “dengue”,  “dengue sintomas”,  “mosquito”,  “mosquito dengue” (7) | “dengue virus”, “dengue fever”, “sintomas da dengue” (3) | 0.537 | 0.688 |
| Goiás (GO) | 11 | 0.785 | “aedes”,  “aedes aegypti”,  “aegypti”,  “dengue”,  “dengue virus”, “mosquito”,  “mosquito dengue”,  “sintomas da dengue” (8) | “dengue fever”,  “DHF”,  “dengue sintomas” (3) | 0.66 | 0.768 |
| Maranhão (MA) | 9 | 0.859 | “aedes”,  “aedes aegypti”,  “aegypti”,  “dengue”,  “dengue sintomas”,  “mosquito”,  “mosquito dengue” (7) | “dengue virus”, “sintomas da dengue” (2) | 0.615 | 0.856 |
| Minas Gerais (MG) | 12 | 0.923 | “aedes aegypti”,  “aegypti”,  “dengue”,  “dengue sintomas”,  “dengue virus”,  “mosquito”,  “mosquito dengue”,  “sintomas da dengue” (8) | “aedes”, “dengue fever”, “DHF”, “mosquitoes” (4) | 0.718 | 0.919 |
| Mato Grosso do Sul (MS) | 9 | 0.713 | “aedes”,  “aedes aegypti”,  “dengue”,  “dengue sintomas”,  “mosquito”,  “mosquito dengue” (6) | “aegypti”, “dengue virus”, “sintomas da dengue” (3) | 0.583 | 0.694 |
| Mato Grosso (MT) | 9 | 0.573 | “aedes”,  “aegypti”,  “dengue”,  “dengue sintomas”,  “mosquito”,  “mosquito dengue” (6) | “aedes aegypti”, “DHF”, “sintomas da dengue” (3) | 0.453 | 0.559 |
| Pará (PA) | 9 | 0.6 | “aedes”,  “aedes aegypti”,  “aegypti”,  “dengue”,  “dengue sintomas”,  “mosquito”,  “mosquito dengue” (7) | “dengue virus”, “sintomas da dengue” (2) | 0.277 | 0.596 |
| Paraiba (PB) | 10 | 0.837 | “aedes”,  “aedes aegypti”,  “aegypti”,  “dengue”,  “dengue sintomas”,  “mosquito”,  “mosquito dengue” (7) | “dengue fever”, “dengue virus”, “sintomas da dengue” (3) | 0.683 | 0.832 |
| Pernambuco (PE) | 10 | 0.819 | “aedes”,  “aedes aegypti”,  “aegypti”,  “dengue”,  “dengue virus”,  “mosquito”,  “mosquito dengue”,  “sintomas da dengue” (8) | “dengue sintomas”, “DHF” (2) | 0.714 | 0.808 |
| Piauí (PI) | 8 | 0.553 | “aedes”,  “aedes aegypti”,  “aegypti”,  “dengue”,  “mosquito” (5) | “dengue sintomas”, “mosquito dengue”, “sintomas da dengue” (3) | 0.377 | 0.542 |
| Paraná (PR) | 12 | 0.845 | “aedes”,  “aedes aegypti”,  “aegypti”,  “dengue”,  “dengue sintomas”,  “dengue virus”,  “mosquito”,  “mosquito dengue” (8) | “dengue fever”, “DHF”,  “mosquitoes”, “sintomas da dengue” (4) | 0.741 | 0.821 |
| Rio de Janeiro (RJ) | 12 | 0.765 | “aedes”,  “aegypti”,  “dengue”,  “dengue sintomas”,  “dengue virus”,  “mosquito”,  “mosquito dengue” (7) | “aedes aegypti”, “dengue fever”, “DHF”, “mosquitoes”, “sintomas da dengue” (5) | 0.704 | 0.744 |
| Rio Grande do Norte (RN) | 9 | 0.89 | “aedes”,  “aedes aegypti”,  “aegypti”,  “dengue”,  “dengue sintomas”,  “mosquito”,  “mosquito dengue” (7) | “dengue virus”, “sintomas da dengue” (2) | 0.706 | 0.891 |
| Rondônia (RO) | 9 | 0.568 | “aedes”,  “aedes aegypti”,  “aegypti”,  “dengue”,  “mosquito” (5) | “dengue sintomas”, “dengue virus”, “mosquito dengue”, “sintomas da dengue” (4) | 0.415 | 0.544 |
| Rio Grande do Sul (RS) | 12 | 0.804 | “aedes”,  “aedes aegypti”,  “aegypti”,  “dengue”,  “dengue sintomas”,  “dengue virus”,  “mosquito”,  “mosquito dengue” (8) | “dengue fever”, “DHF”, “mosquitoes”, “sintomas da dengue” (4) | 0.724 | 0.779 |
| Roraima (RR) | 7 | 0.093 | “aegypti” (1) | “aedes”,  “aedes aegypti”,  “dengue”,  “dengue sintomas”,  “mosquito”,  “sintomas da dengue” (6) | 0.091 | 0.058 |
| Santa Catarina (SC) | 9 | 0.826 | “aedes”,  “aedes aegypti”,  “aegypti”,  “dengue”,  “dengue sintomas”,  “dengue virus”,  “mosquito dengue” (7) | “mosquito”, “sintomas da dengue” (2) | 0.788 | 0.825 |
| Sergipe (SE) | 8 | 0.308 | “aedes”,  “aedes aegypti”,  “aegypti”,  “dengue”,  “mosquito” (5) | “dengue sintomas”, “mosquito dengue”, “sintomas da dengue” (3) | 0.216 | 0.3 |
| São Paulo (SP) | 13 | 0.93 | “aedes aegypti”,  “aegypti”,  “dengue”,  “dengue sintomas”,  “dengue virus”,  “mosquito”,  “mosquito dengue” (7) | “aedes”,  “dengue fever”,  “dengue hemorrhagic fever”, “DHF”, “mosquitoes”, “sintomas da dengue” (6) | 0.861 | 0.919 |
| Tocantins (TO) | 8 | 0.415 | “aedes”,  “aegypti”,  “dengue”,  “mosquito”,  “mosquito dengue” (5) | “aedes aegypti”,  “dengue sintomas”, “sintomas da dengue” (3) | 0.342 | 0.401 |
| Brazil (BR) | 14 | 0.889 | “aedes”,  “aedes aegypti”,  “dengue”,  “dengue sintomas”,  “dengue virus”,  “mosquito”,  “mosquito dengue” (7) | “aegypti”, “dengue fever”, “dengue hemorrhagic fever”, “DENV”,  “DHF”, “mosquitoes”, “sintomas da dengue” (7) | 0.852 | 0.85 |

**Acré (AC):**

**Alagoas (AL)**

**Amazonas (AM):**

**Amapá (AP)**

**Bahia (BA)**

**Ceará (CE)**

**Distrito Federal (DF)**

**Espírito Santo(ES)**

**Goiás (GO)**

**Maranhão (MA)**

**Minas Gerais (MG)**

**Mato Grosso do Sul (MS)**

**Mato Grosso (MT)**

**Pará (PA)**

**Paraiba (PB)**

**Pernambuco (PE)**

**Piauí (PI)**

**Paraná (PR)**

**Rio de Janeiro (RJ)**

**Rio Grande do Norte (RN)**

**Rondônia (RO)**

**Roraima (RR)**

**Rio Grande do Sul (RS)**

**Santa Catarina (SC)**

**Sergipe (SE)**

**São Paulo (SP)**

**Tocantins (TO)**

**Brazil (BR)**
