# Supplementary material for: Google Health Trends performance reflecting dengue incidence for the Brazilian states
Source: BMC Infect Dis. 2020 Mar 26;20:252. doi: 10.1186/s12879-020-04957-0 (PMC7104526; doi:10.1186/s12879-020-04957-0)
Supplement: Supplementary file 4 — Additional file 4. Plots of multiple and simple linear models between Google Health Trends data and weekly dengue incidence for Brazil. [file 12879_2020_4957_MOESM4_ESM.pdf]

Google Health Trends performance reflecting dengue incidence for the Brazilian states

Daniel Romero-Alvarez, Nidhi Parikh, Dave Osthus, Kaitlyn Martinez, Nicholas Generous, Sara del Valle, Carrie A. Manore

Additional file: Plots of multiple and simple linear models between Google Health Trends data and weekly dengue incidence for Brazil

Brazil (BR)
Dengue incidence vs. Google Health Trends (2011–2016)

Definitions:

- **Combined terms:**
  - **All:** model with all the available terms. n= number of terms
  - **Four:** model with "dengue", "dengue sintomas", "aedes", and "mosquito".
  - **Uncorrelated:** model with non-correlated terms. n = number of uncorrelated terms
- **Individual terms:** Plots developed with one term at a time.

- Terms for all and uncorrelated models can be found in additional file 3.

Combined terms
Adjusted R squared

Brazil

All
(n = 14)

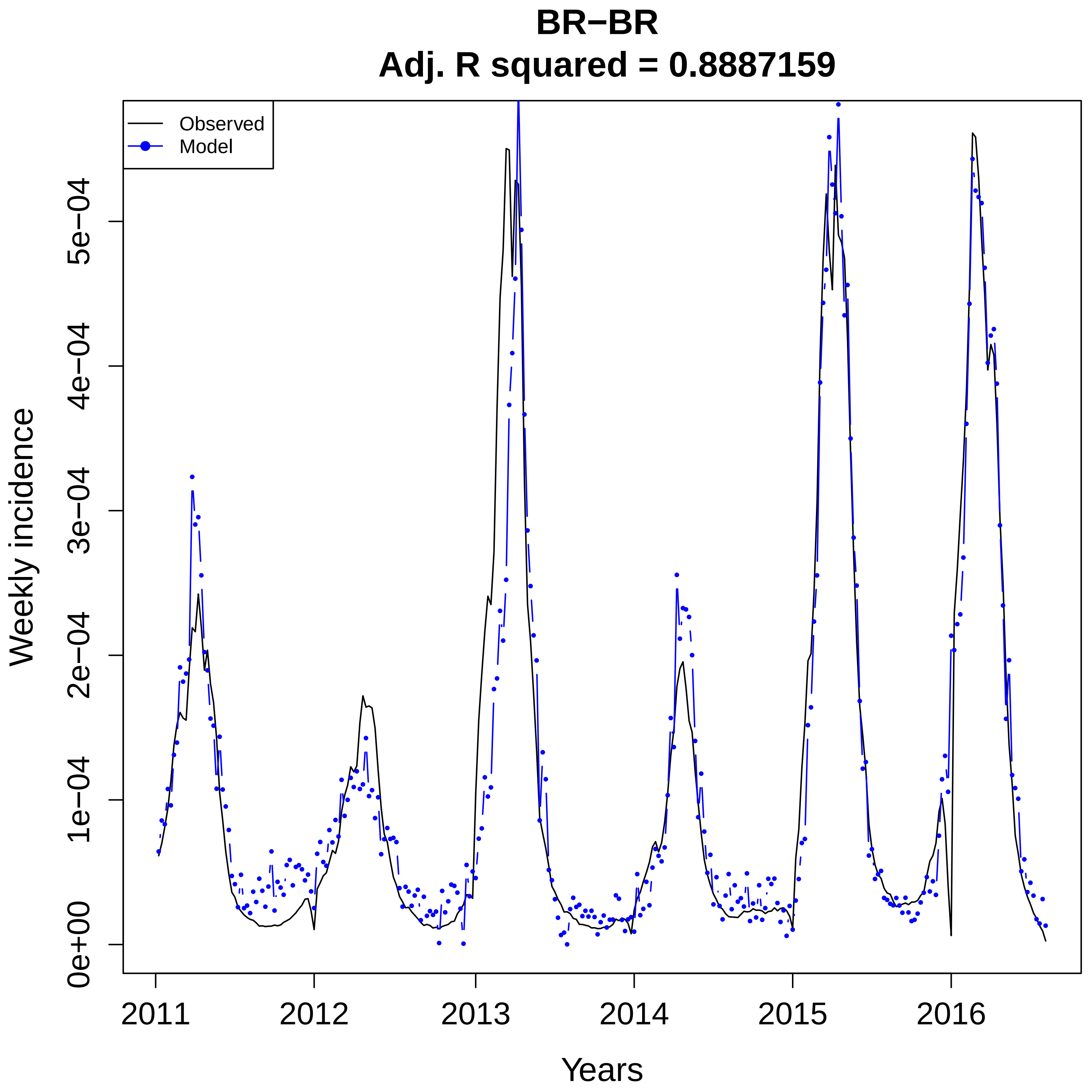
BR−BR
Adj. R squared = 0.8887159
Observed
Model
Weekly incidence
0e+00
1e−04
2e−04
3e−04
4e−04
5e−04
2011
2012
2013
2014
2015
2016
Years

Four

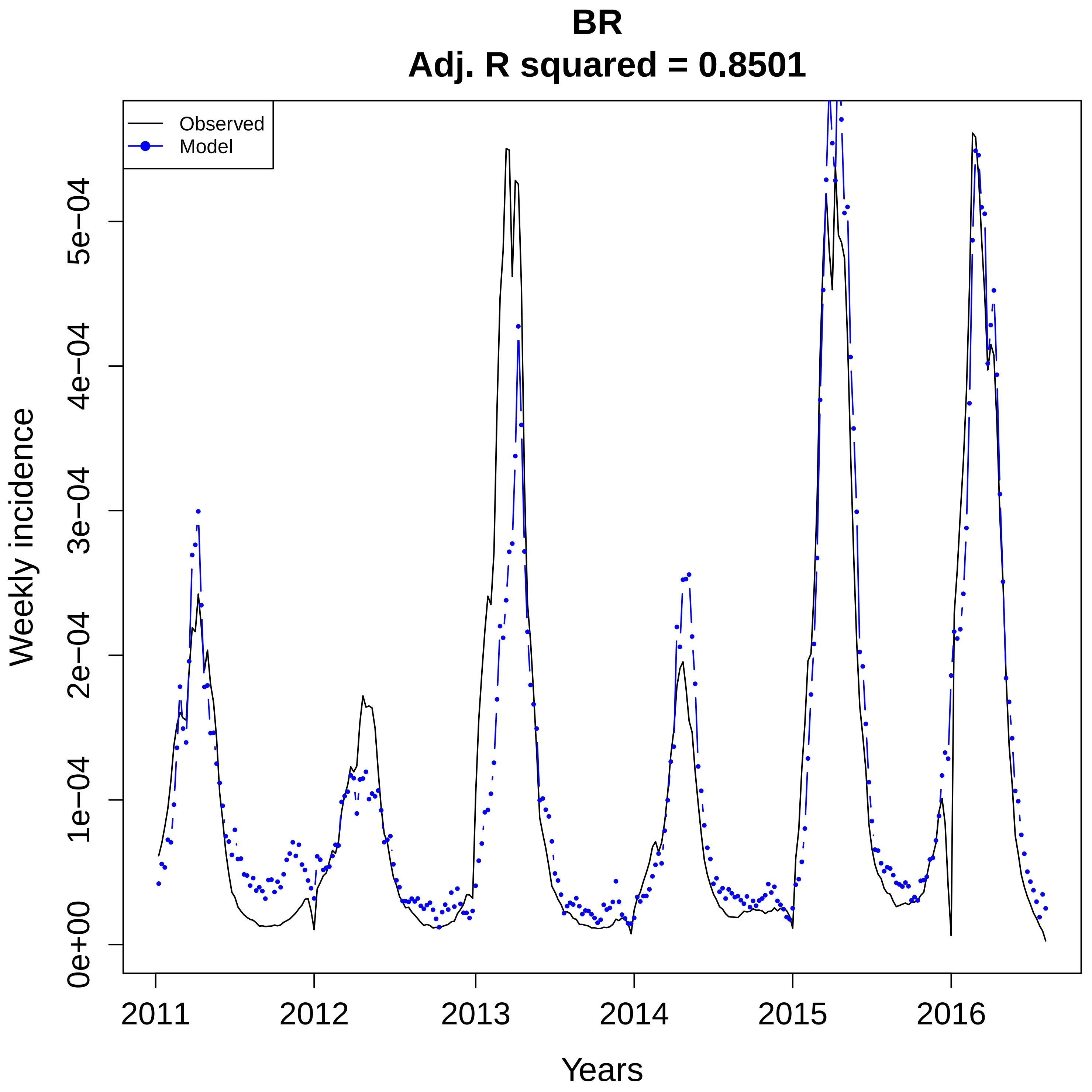

BR
Adj. R squared = 0.8501
Observed
Model
Weekly incidence
0e+00
1e−04
2e−04
3e−04
4e−04
5e−04
2011
2012
2013
2014
2015
2016
Years

Uncorrelated (n = 7)

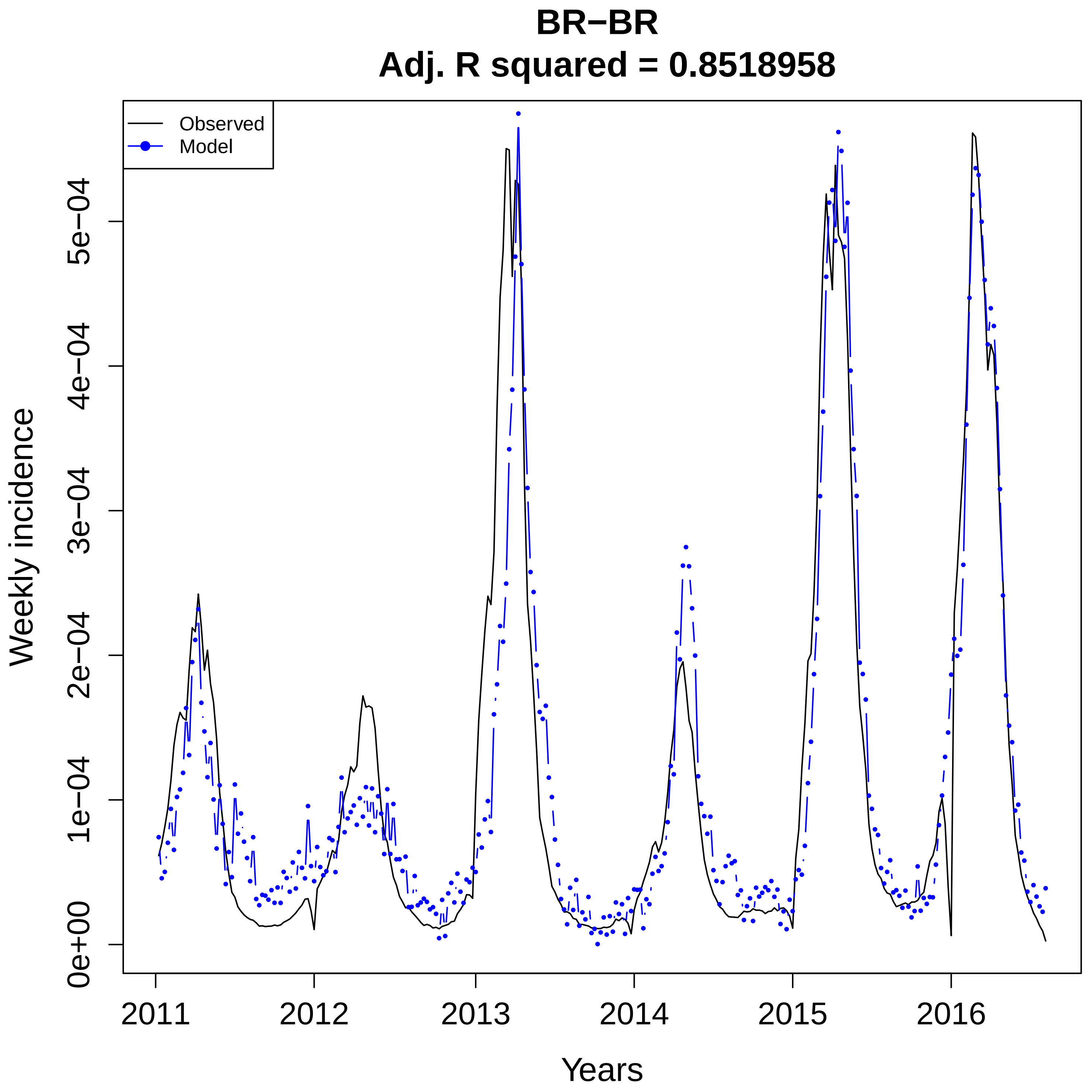

Individual terms
Adjusted R squared

Brazil

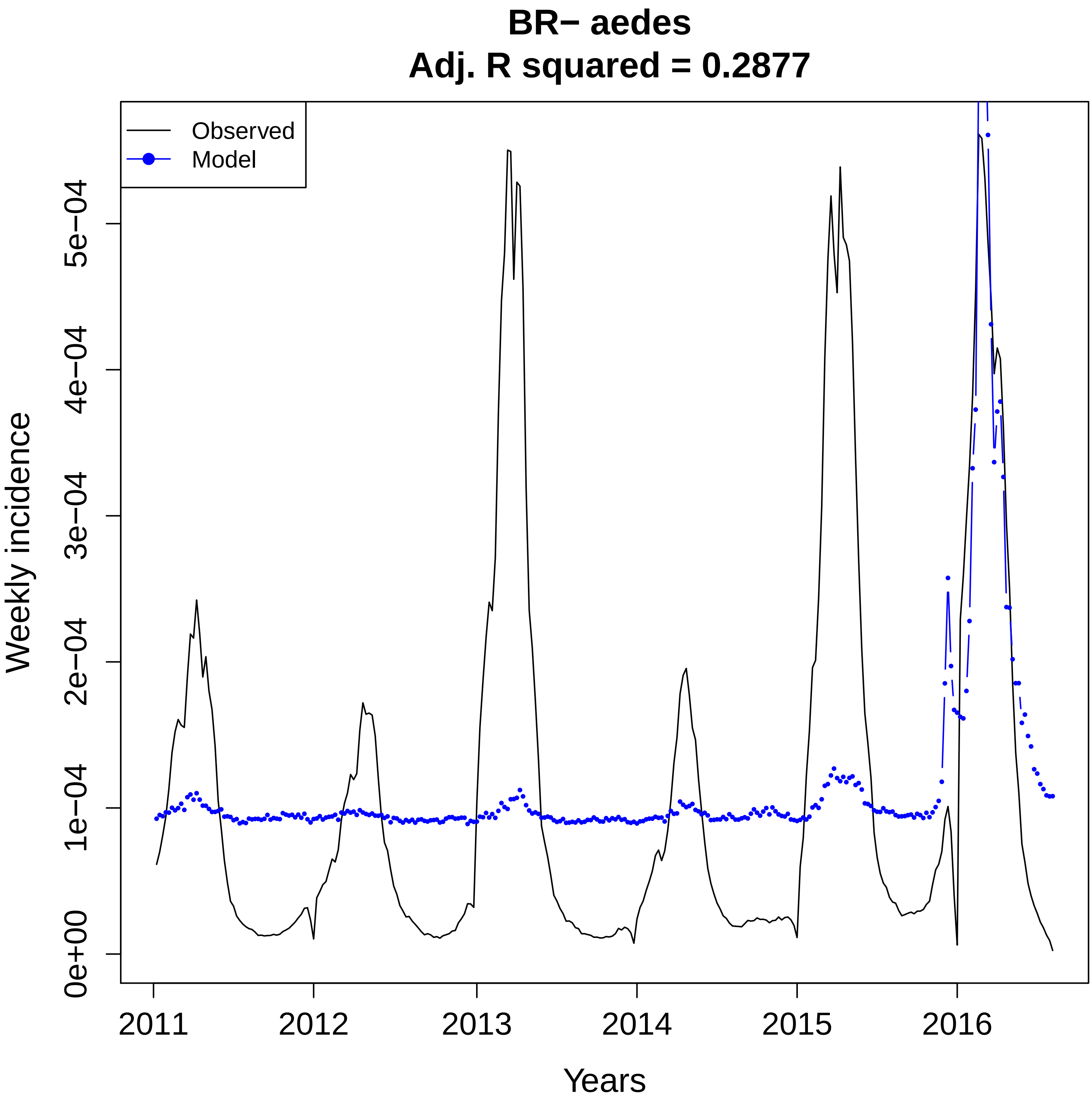
BR− aedes
Adj. R squared = 0.2877
Observed
Model
Weekly incidence
0e+00
1e−04
2e−04
3e−04
4e−04
5e−04
2011
2012
2013
2014
2015
2016
Years

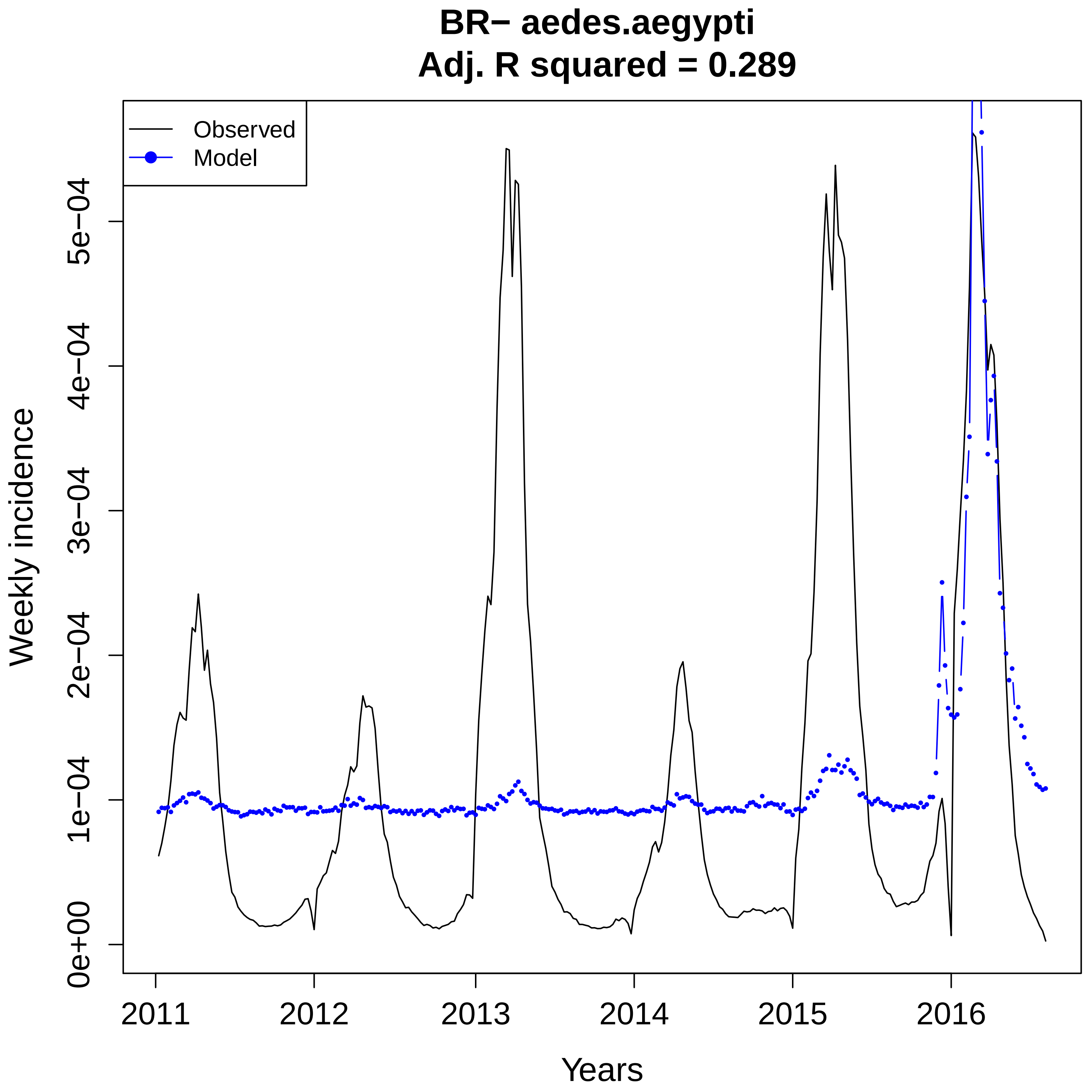
BR− aedes.aegypti
Adj. R squared = 0.289
Observed
Model
Weekly incidence
0e+00
1e−04
2e−04
3e−04
4e−04
5e−04
2011
2012
2013
2014
2015
2016
Years

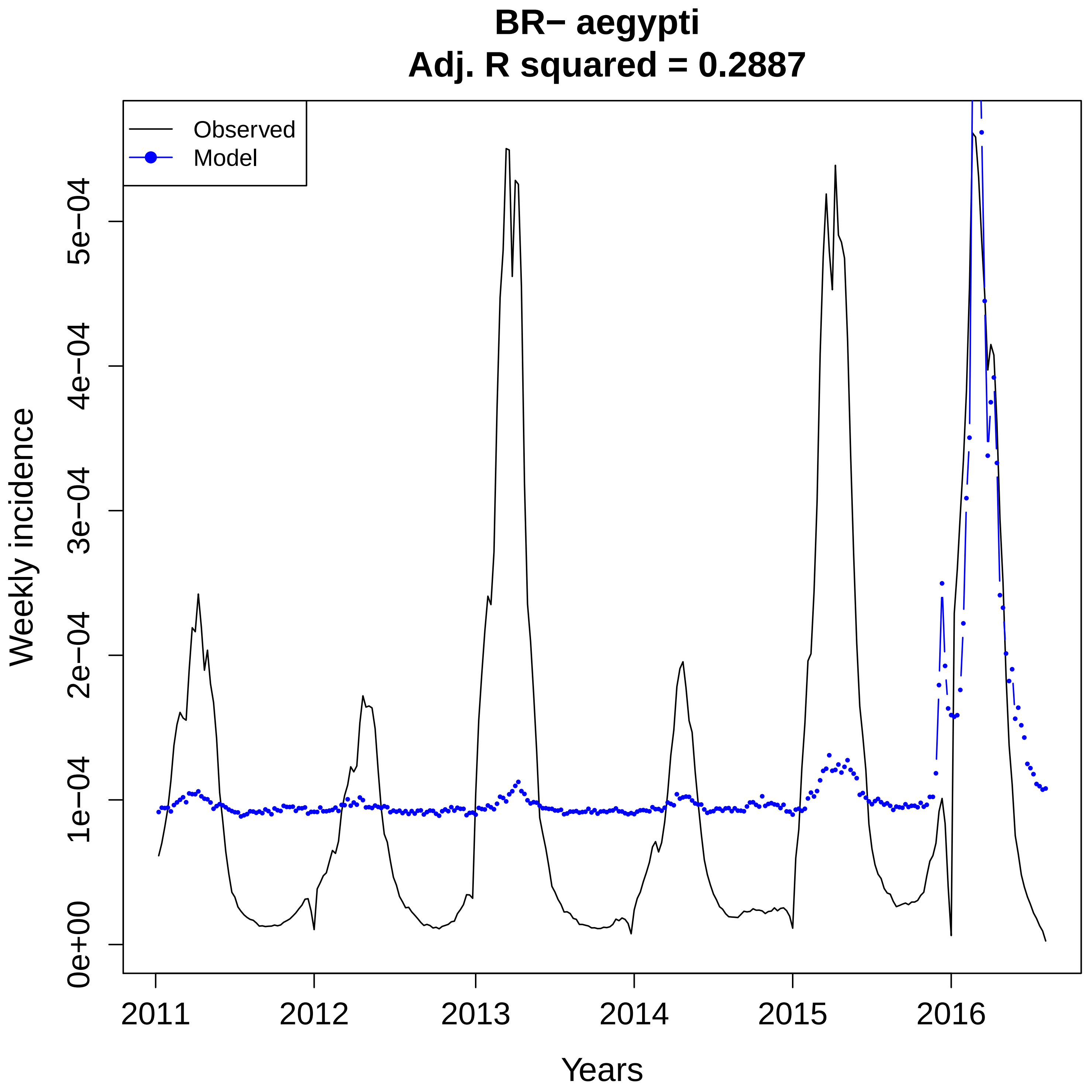
BR− aegypti
Adj. R squared = 0.2887
Observed
Model
Weekly incidence
0e+00
1e−04
2e−04
3e−04
4e−04
5e−04
2011
2012
2013
2014
2015
2016
Years

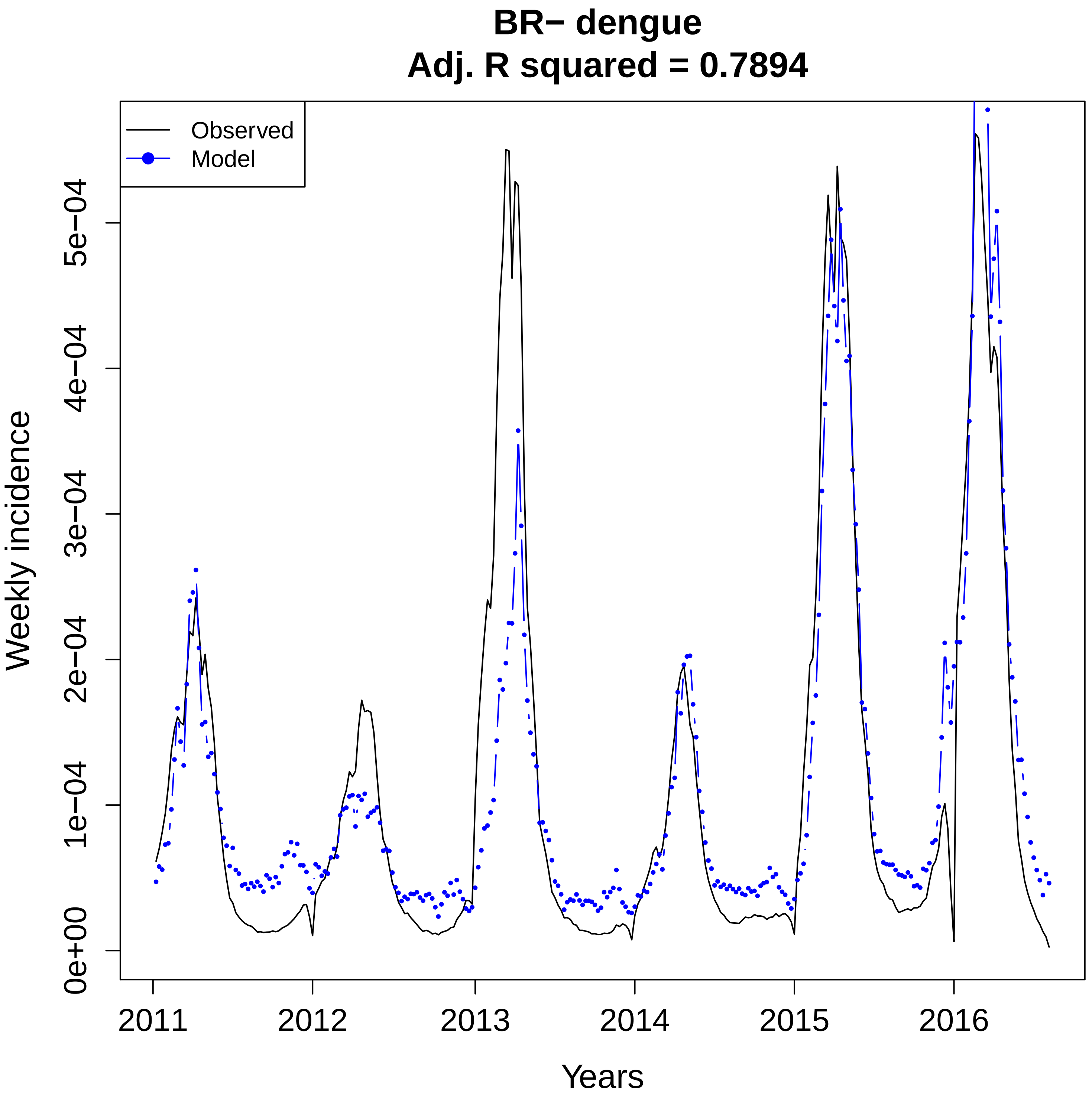
BR– dengue
Adj. R squared = 0.7894
Observed
Model
Weekly incidence
0e+00
1e−04
2e−04
3e−04
4e−04
5e−04
2011
2012
2013
2014
2015
2016
Years

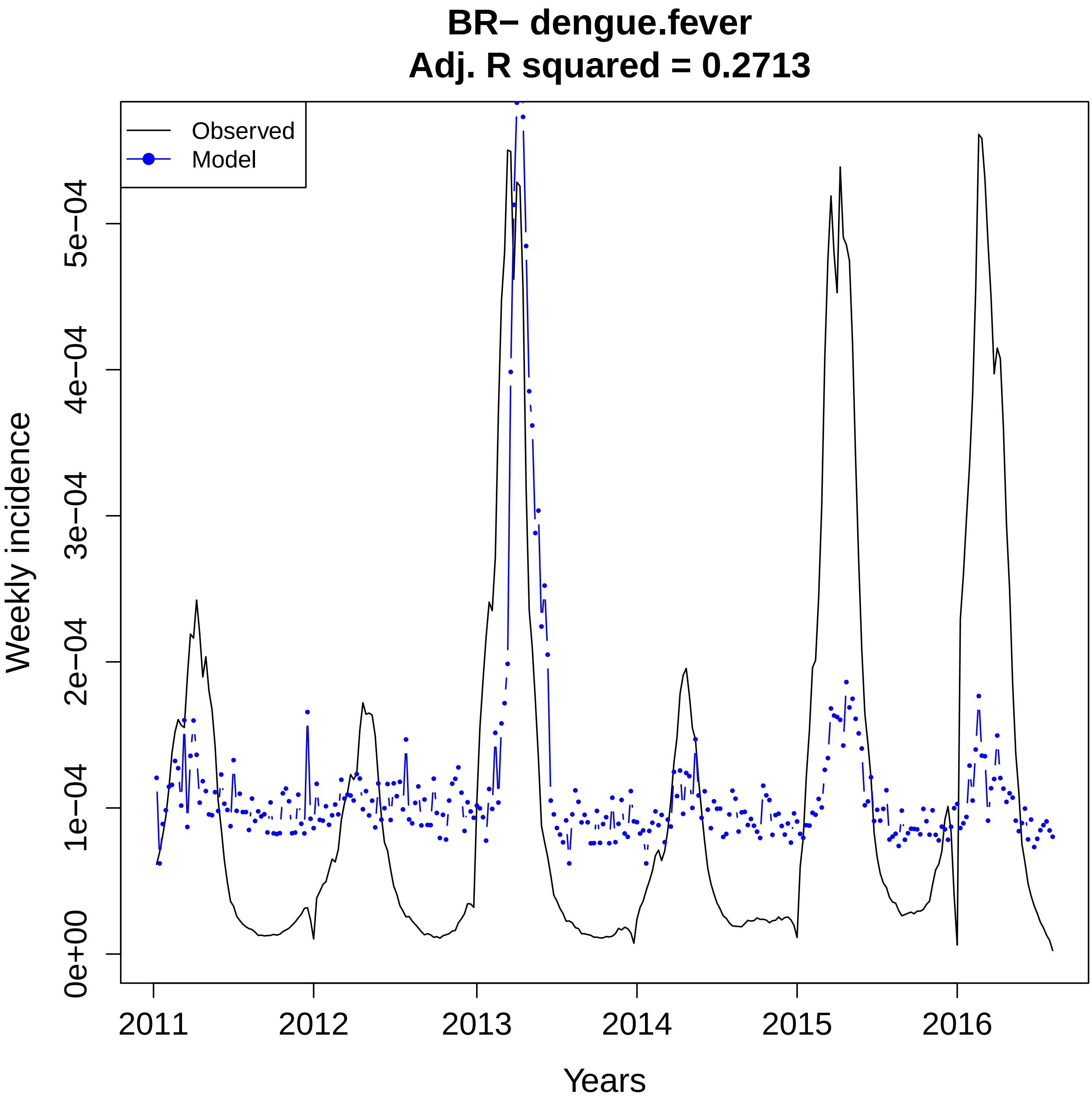
BR− dengue.fever
Adj. R squared = 0.2713
Observed
Model
Weekly incidence
0e+00
1e−04
2e−04
3e−04
4e−04
5e−04
2011
2012
2013
2014
2015
2016
Years

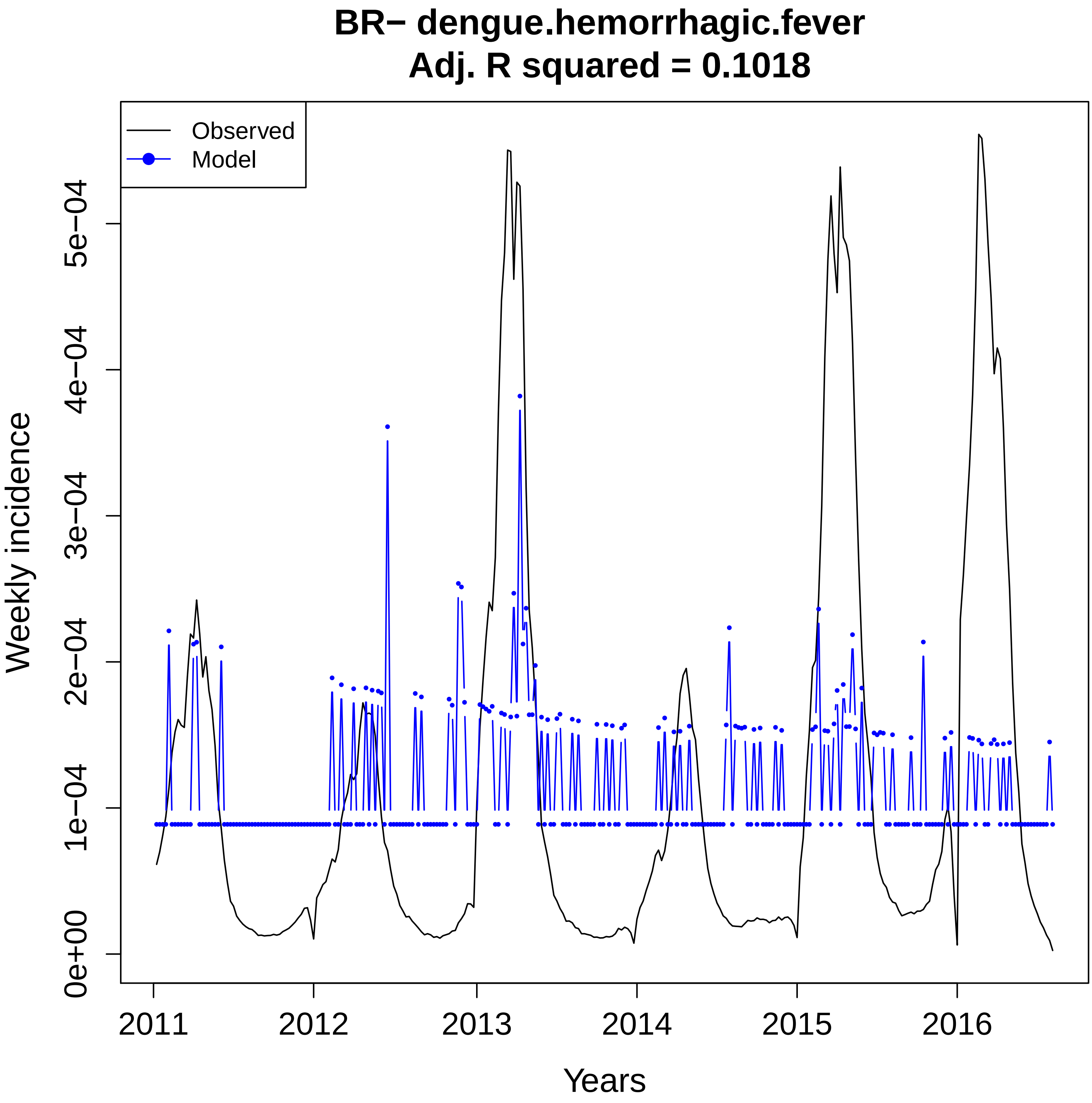
BR− dengue.hemorrhagic.fever
Adj. R squared = 0.1018
Observed
Model
Weekly incidence
0e+00
1e−04
2e−04
3e−04
4e−04
5e−04
2011
2012
2013
2014
2015
2016
Years

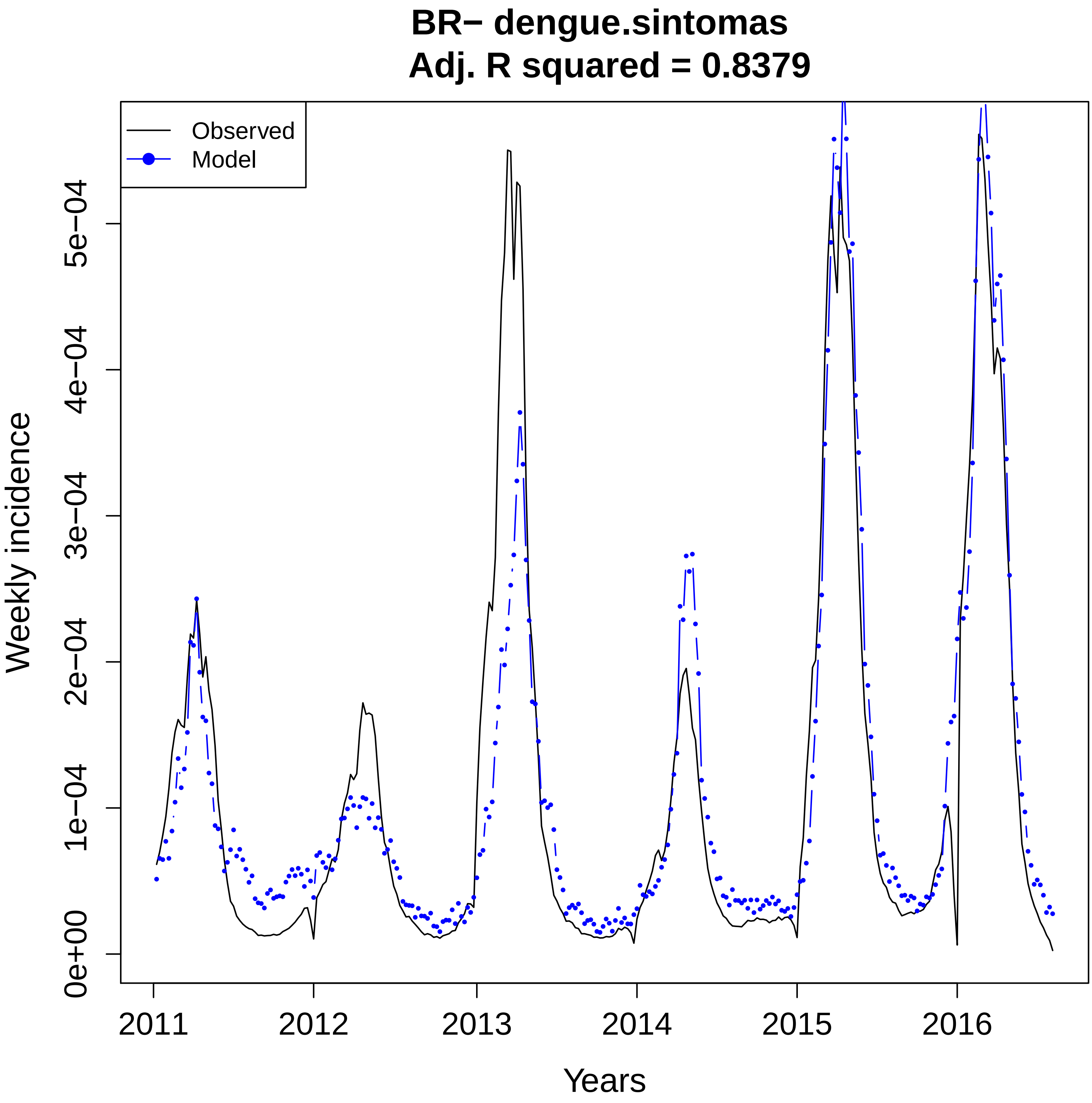
BR− dengue.sintomas
Adj. R squared = 0.8379
Observed
Model
Weekly incidence
0e+00
1e−04
2e−04
3e−04
4e−04
5e−04
2011
2012
2013
2014
2015
2016
Years

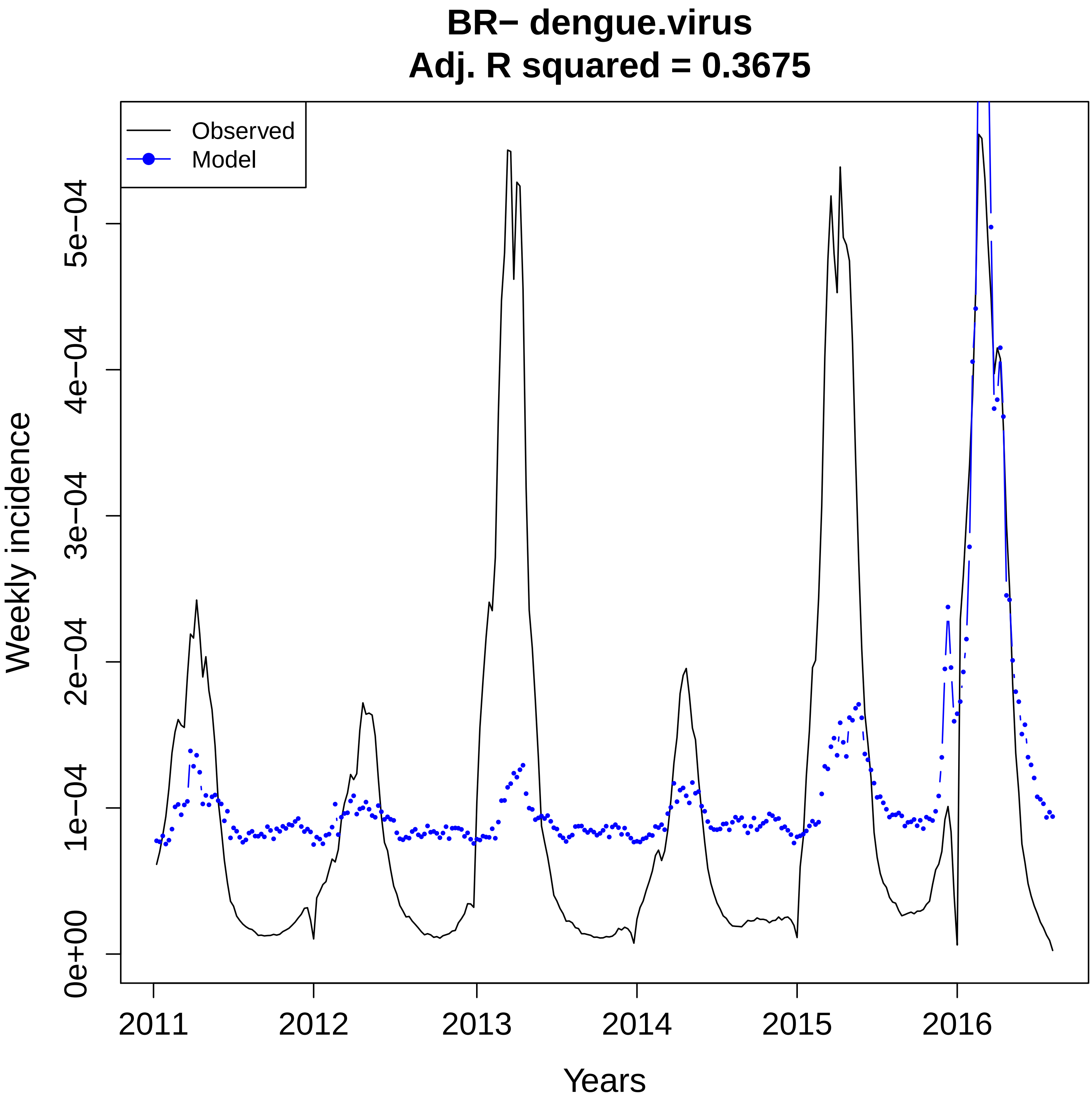

BR− dengue.virus
Adj. R squared = 0.3675
Observed
Model
Weekly incidence
0e+00
1e−04
2e−04
3e−04
4e−04
5e−04
2011
2012
2013
2014
2015
2016
Years

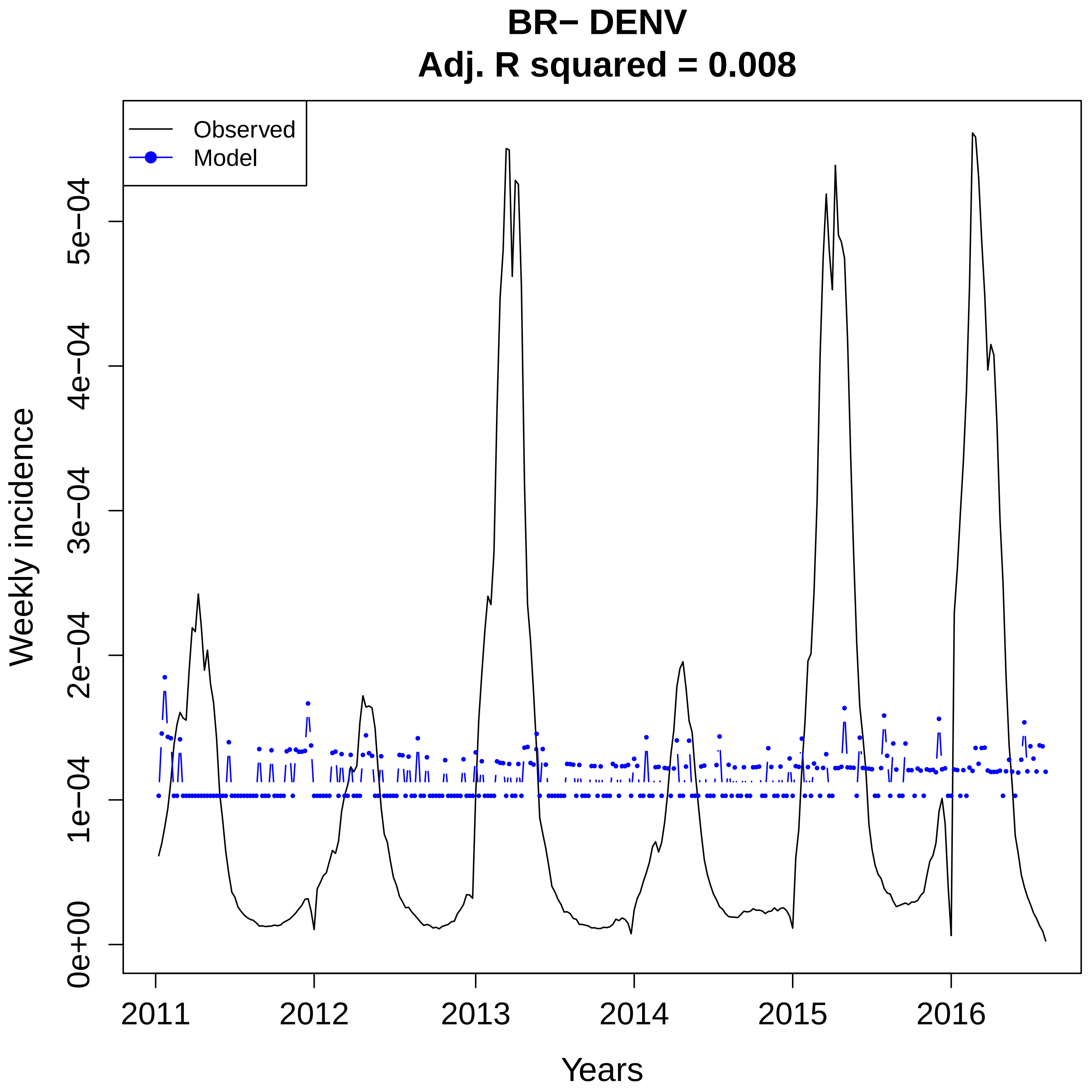
BR− DENV
Adj. R squared = 0.008
Observed
Model
Weekly incidence
0e+00
1e−04
2e−04
3e−04
4e−04
5e−04
2011
2012
2013
2014
2015
2016
Years

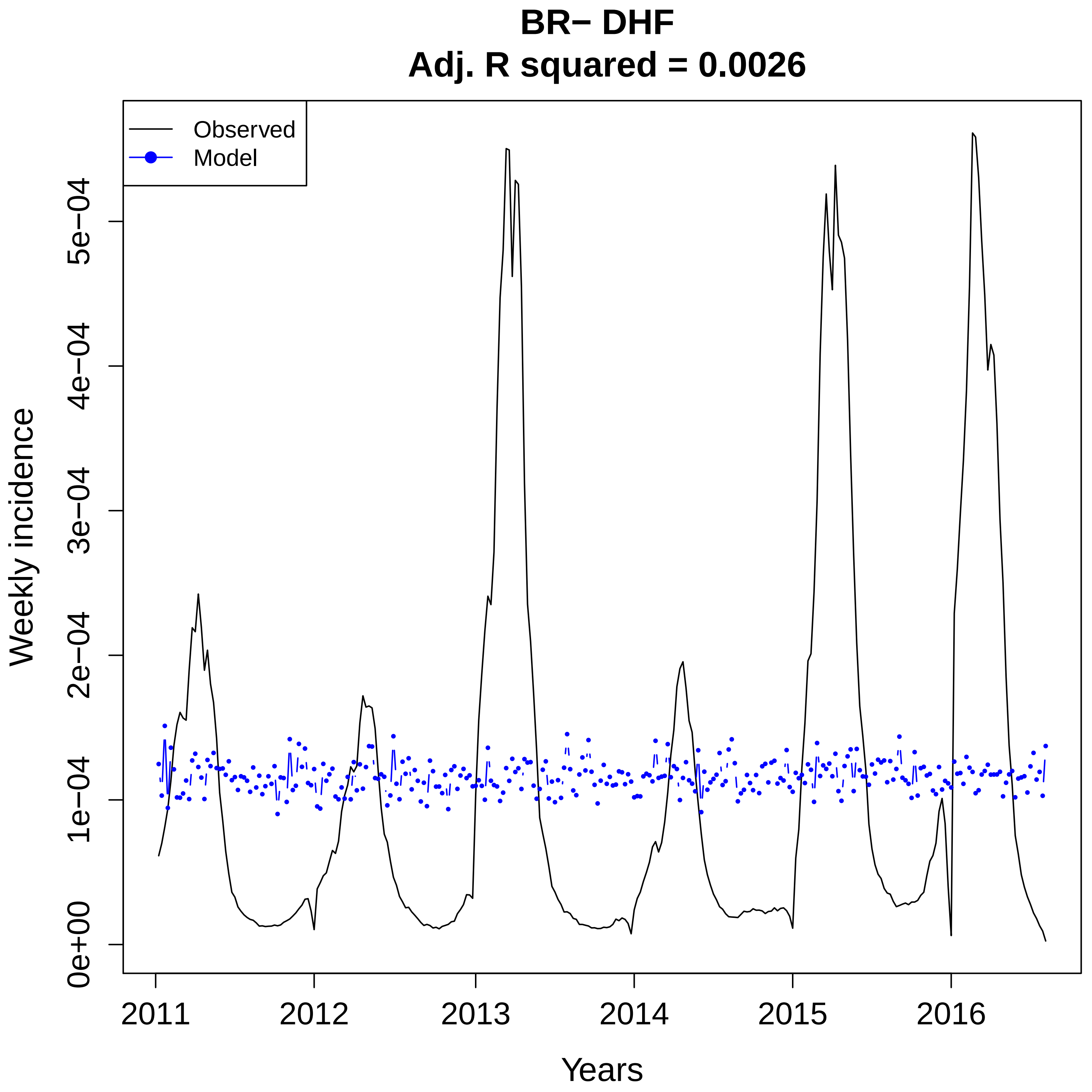

BR− DHF
Adj. R squared = 0.0026
Observed
Model
Weekly incidence
0e+00
1e−04
2e−04
3e−04
4e−04
5e−04
2011
2012
2013
2014
2015
2016
Years

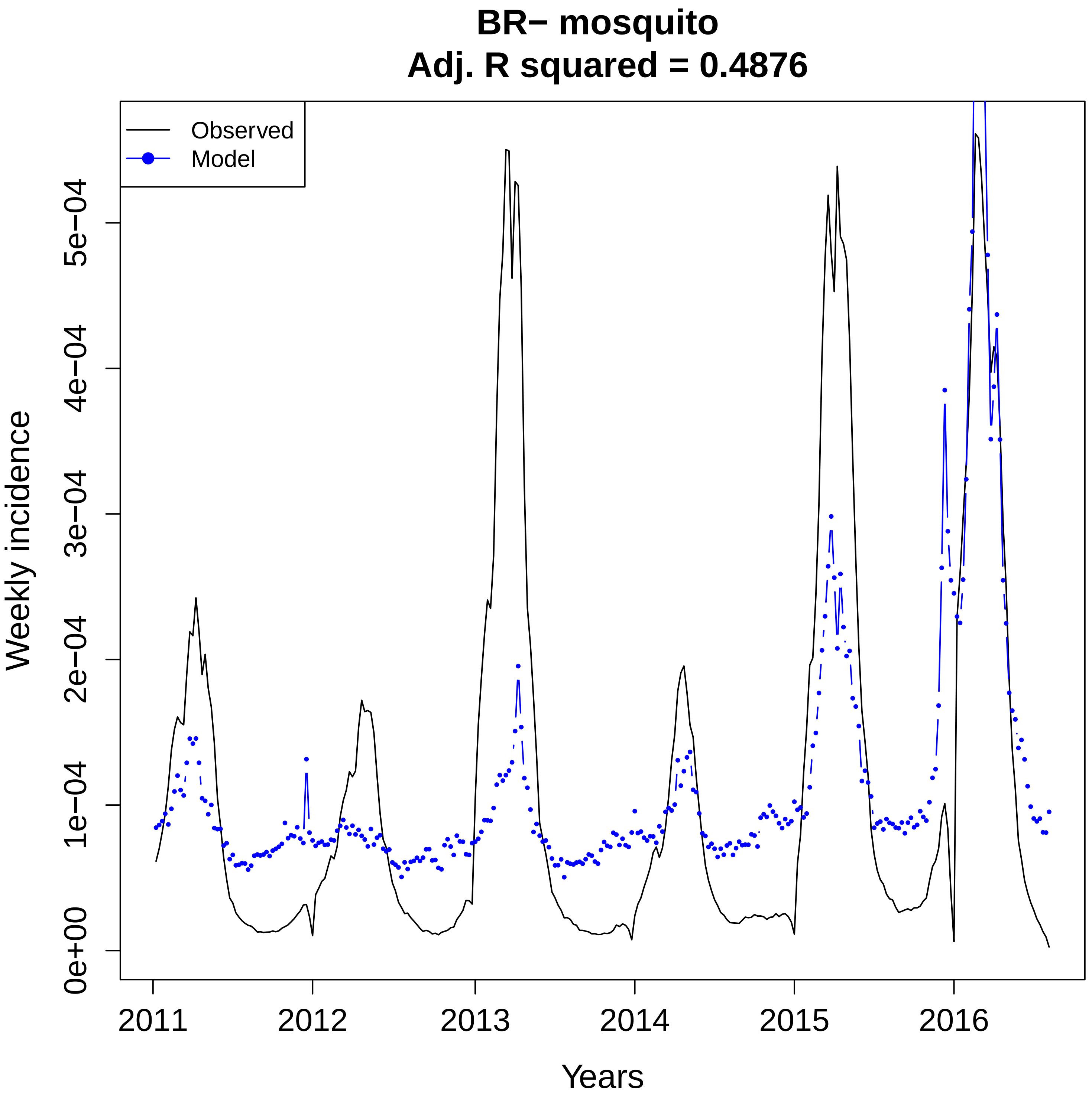

BR− mosquito
Adj. R squared = 0.4876
Observed
Model
Weekly incidence
0e+00
1e−04
2e−04
3e−04
4e−04
5e−04
Years
2011
2012
2013
2014
2015
2016

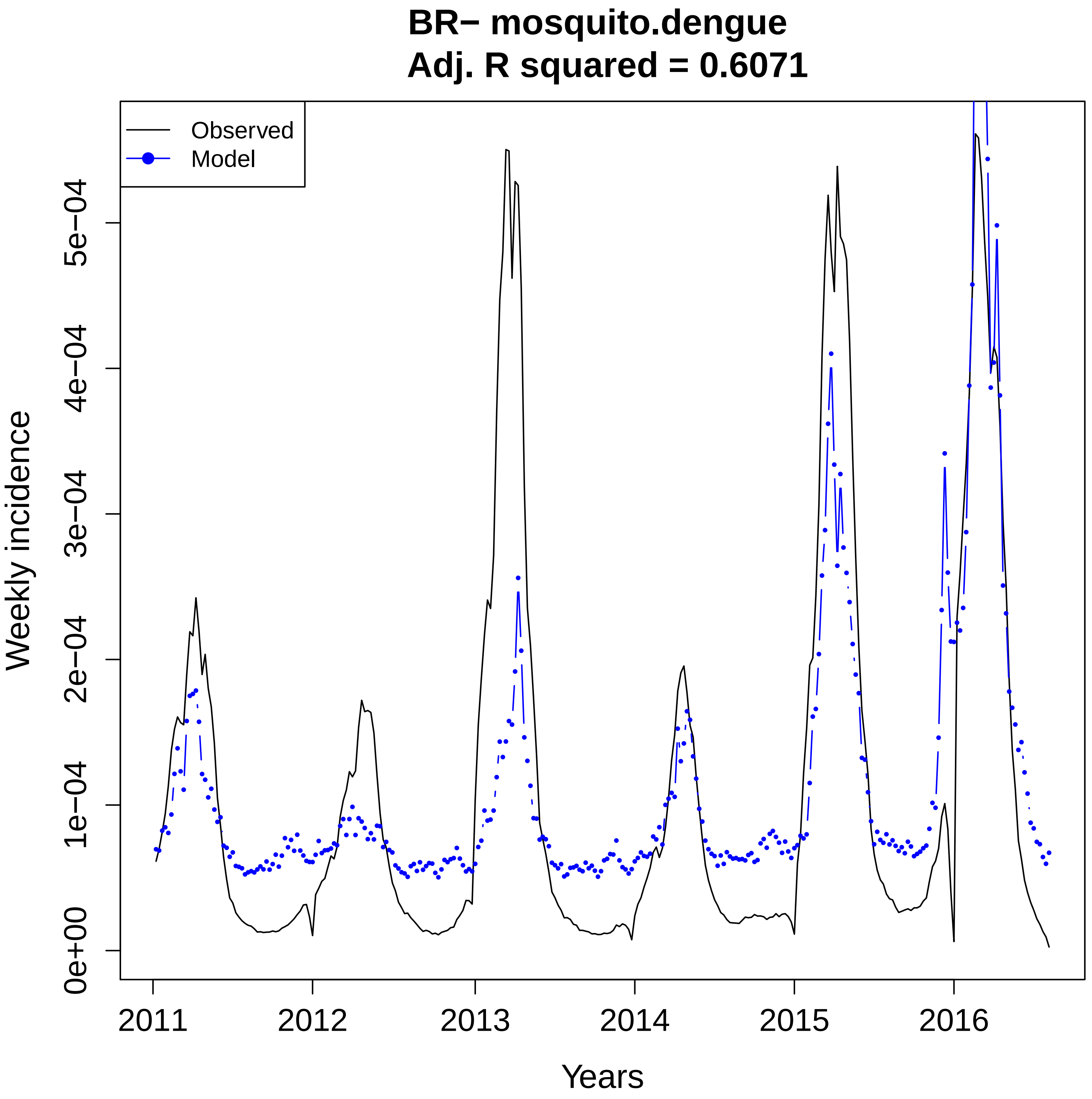
BR− mosquito.dengue
Adj. R squared = 0.6071
Observed
Model
Weekly incidence
0e+00
1e−04
2e−04
3e−04
4e−04
5e−04
2011
2012
2013
2014
2015
2016
Years

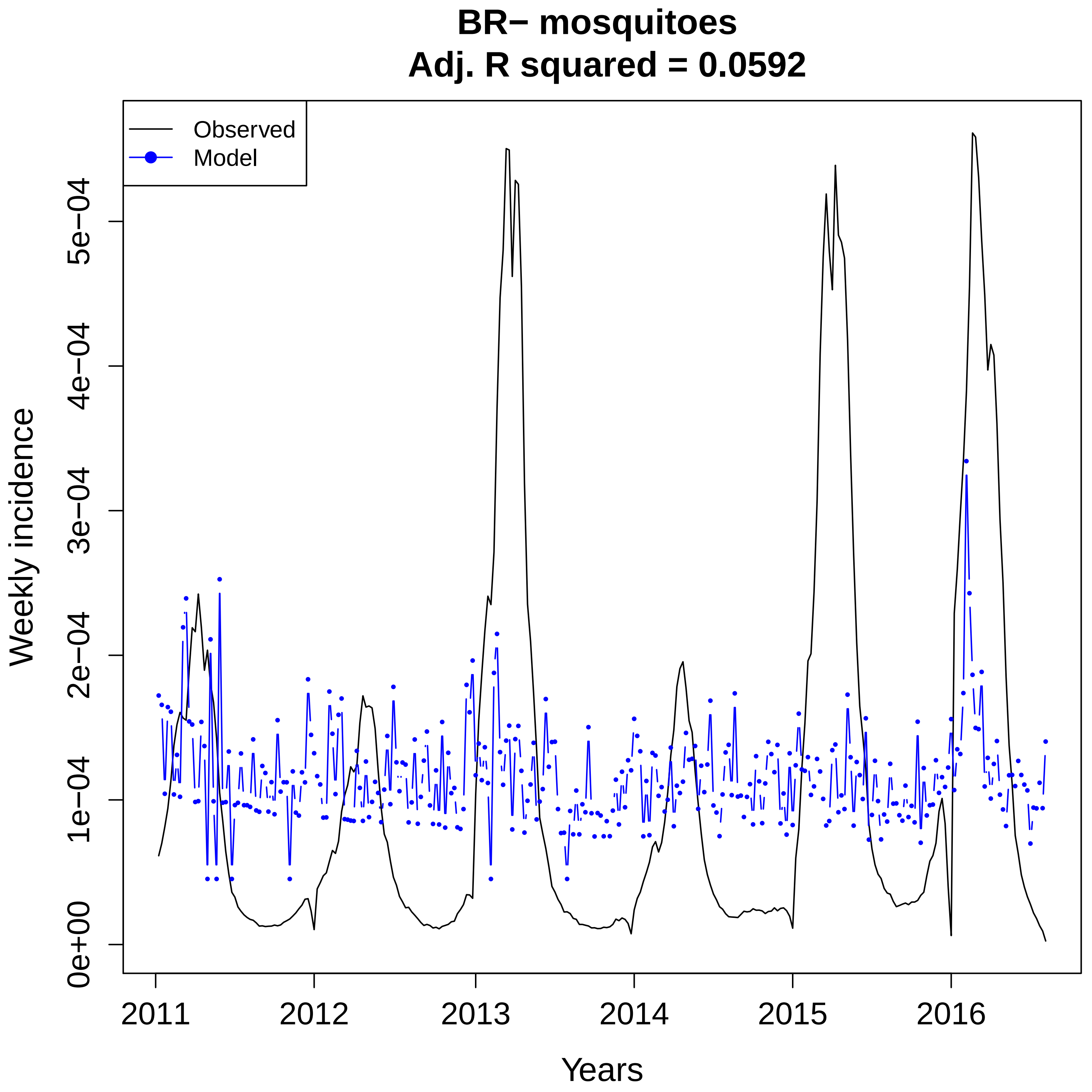
BR− mosquitoes
Adj. R squared = 0.0592
Observed
Model
Weekly incidence
0e+00
1e−04
2e−04
3e−04
4e−04
5e−04
2011
2012
2013
2014
2015
2016
Years

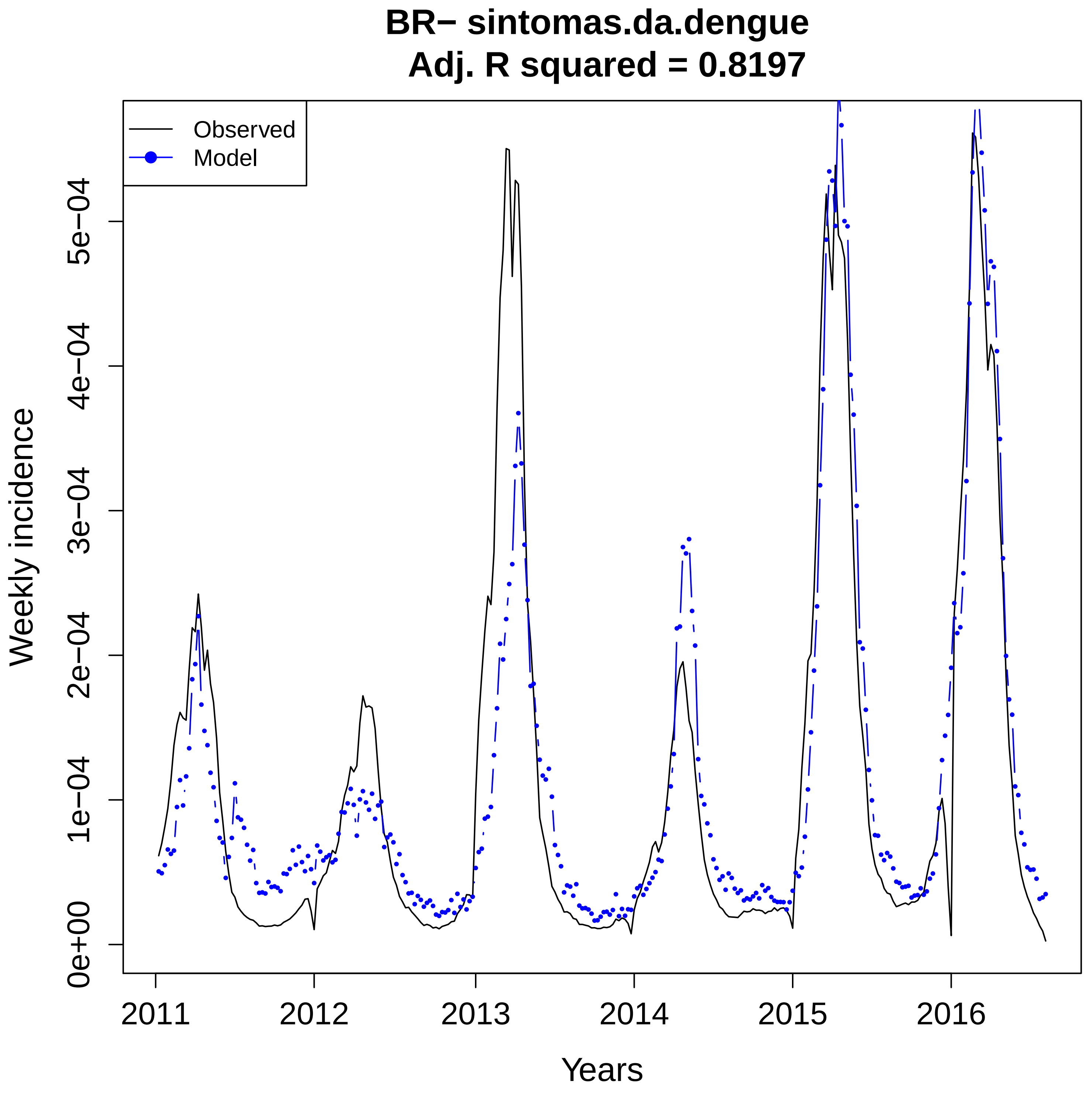
BR− sintomas.da.dengue
Adj. R squared = 0.8197
Observed
Model
Weekly incidence
0e+00
1e−04
2e−04
3e−04
4e−04
5e−04
2011
2012
2013
2014
2015
2016
Years
