# Supplementary material for: Google Health Trends performance reflecting dengue incidence for the Brazilian states
Source: BMC Infect Dis. 2020 Mar 26;20:252. doi: 10.1186/s12879-020-04957-0 (PMC7104526; doi:10.1186/s12879-020-04957-0)
Supplement: Supplementary file 5 — Additional file 5. Plots of multiple and simple linear models between Google Health Trends data and weekly dengue incidence for each of the 27 Brazilian states. [file 12879_2020_4957_MOESM5_ESM.pdf]

Google Health Trends performance reflecting dengue incidence for the Brazilian states

Daniel Romero-Alvarez, Nidhi Parikh, Dave Osthus, Kaitlyn Martinez, Nicholas Generous, Sara del Valle, Carrie A. Manore

Additional file: Plots of multiple and simple linear models between Google Health Trends data and weekly dengue incidence for each of the 27 Brazilian states

Combined and individual terms for all the Brazilian states

Index

- Acré (AC)
- Alagoas (AL)
- Amapá (AP)
- Amazonas (AM)
- Bahia (BA)
- Ceará (CE)
- Distrito Federal (DF)
- Espírito Santo (ES)
- Goiás (GO)
- Maranhão (MA)
- Mato Grosso (MT)
- Mato Grosso do Sul (MS)
- Minas Gerais (MG)
- Pará (PA)
- Paraiba (PB)
- Paraná (PR)
- Pernambuco (PE)
- Piauí (PI)
- Rio de Janeiro (RJ)
- Rio Grande do Norte (RN)
- Rondônia (RO)
- Roraima (RR)
- Santa Catarina (SC)
- São Paulo (SP)
- Sergipe (SE)
- Tocantins (TO)

Acré (AC)

Dengue incidence vs. Google Health Trends (2011–2016)

Definitions:

- **Combined terms:**
  - **All:** model with all the available terms. n= number of terms
  - **Four:** model with "dengue", "dengue sintomas", "aedes", and "mosquito".
  - **Uncorrelated:** model with non-correlated terms. n = number of uncorrelated terms
- **Individual terms:** Plots developed with one term at a time.

- Terms for all and uncorrelated models can be found in additional file 3.

Combined terms
Adjusted R squared

Acré (AC)

All
(n = 7)

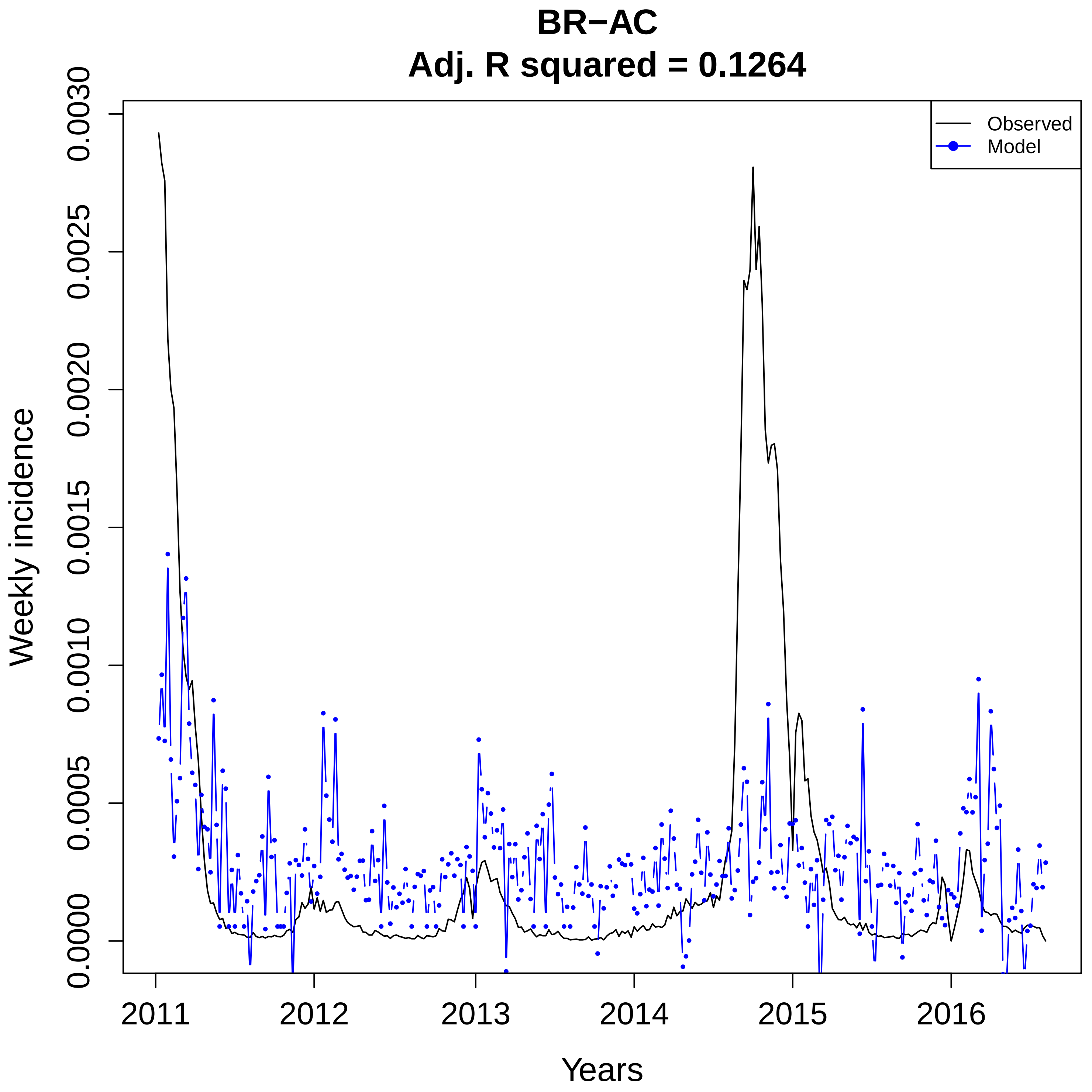
BR−AC
Adj. R squared = 0.1264
Observed
Model
Weekly incidence
0.0000
0.0005
0.0010
0.0015
0.0020
0.0025
0.0030
2011
2012
2013
2014
2015
2016
Years

Four

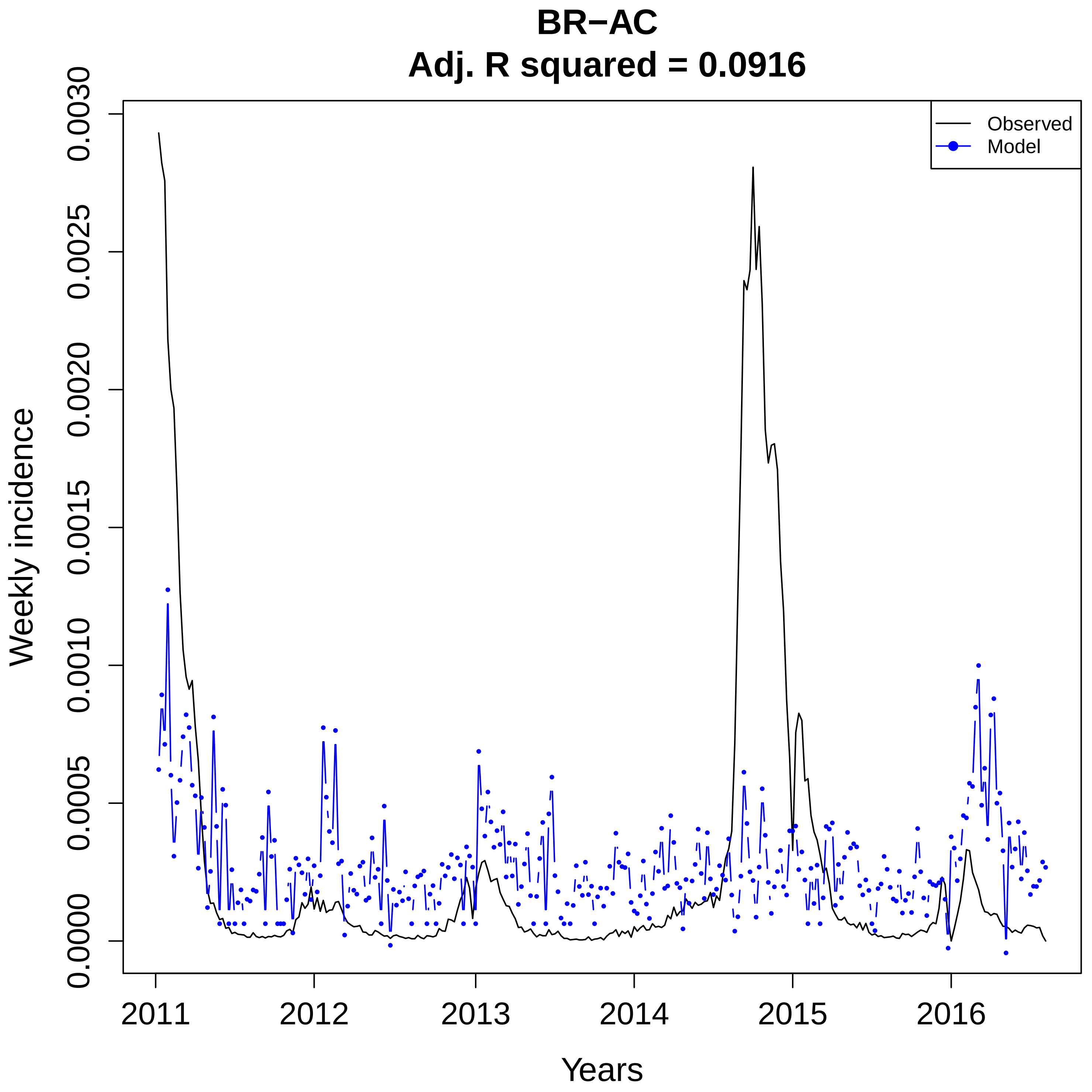
BR−AC
Adj. R squared = 0.0916
Observed
Model
Weekly incidence
0.0000
0.0005
0.0010
0.0015
0.0020
0.0025
0.0030
2011
2012
2013
2014
2015
2016
Years

Uncorrelated
(n = 6)

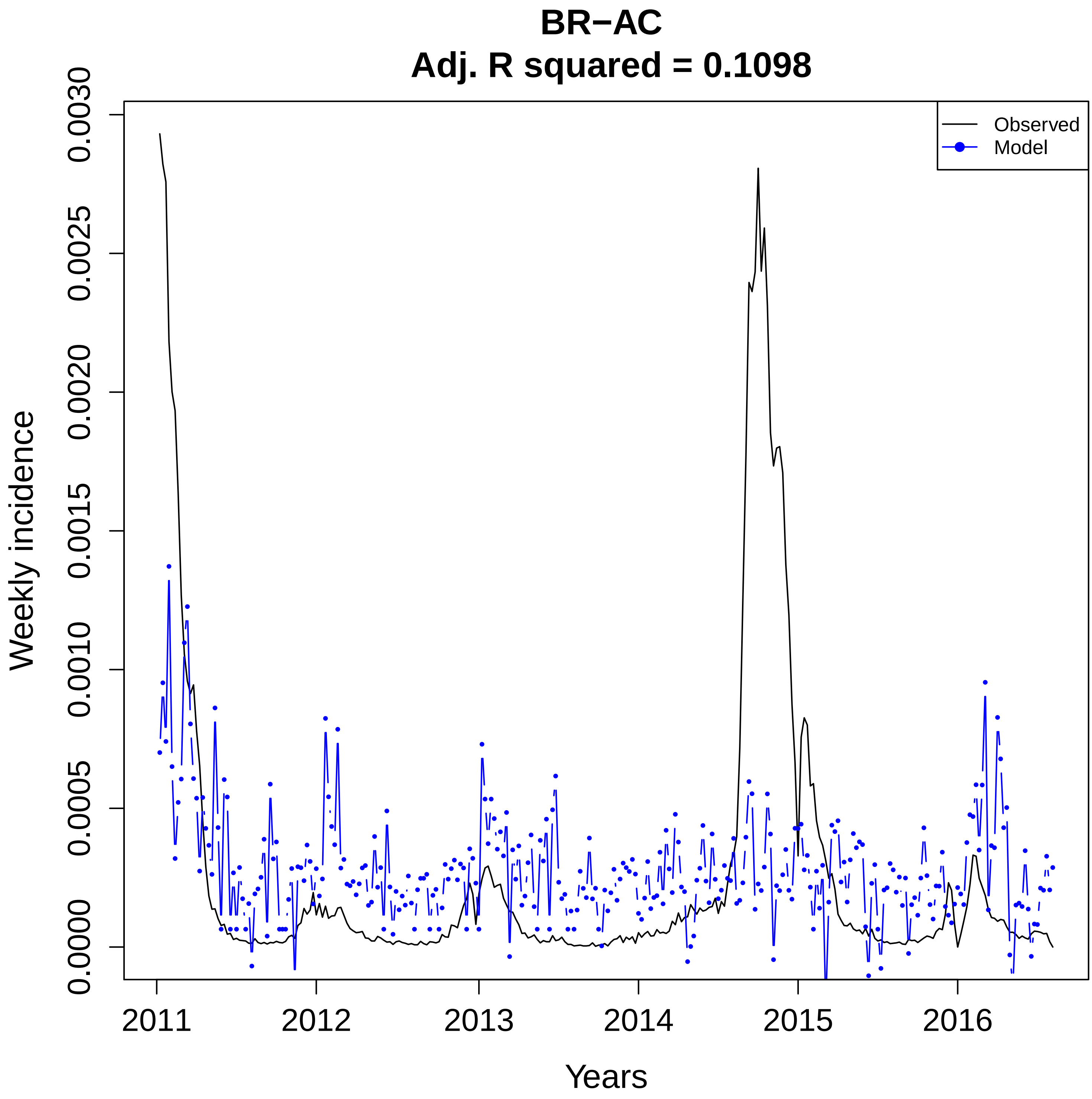

Individual terms
Adjusted R squared

Acré (AC)

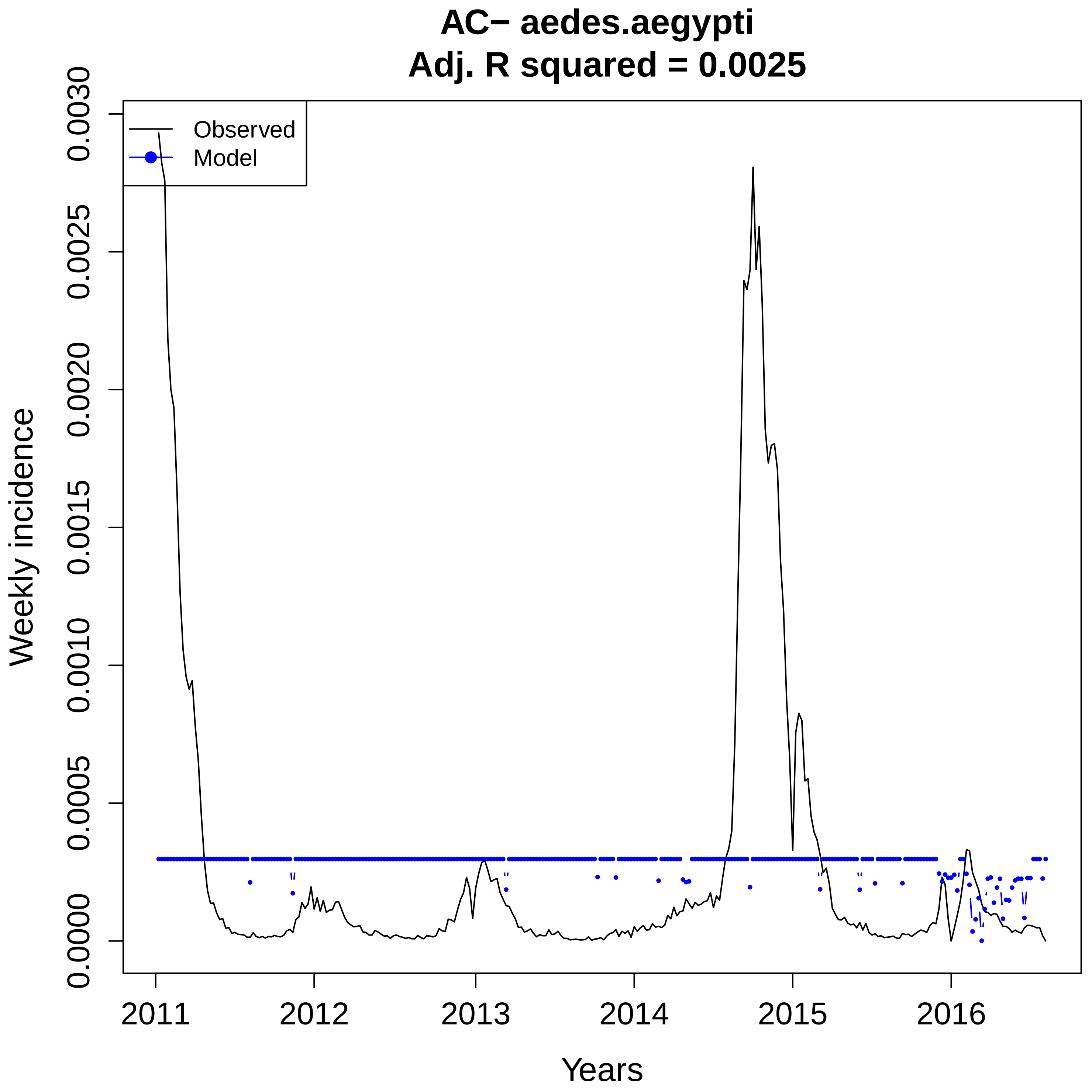
AC− aedes.aegypti
Adj. R squared = 0.0025
Observed
Model
Weekly incidence
0.0000
0.0005
0.0010
0.0015
0.0020
0.0025
0.0030
2011
2012
2013
2014
2015
2016
Years

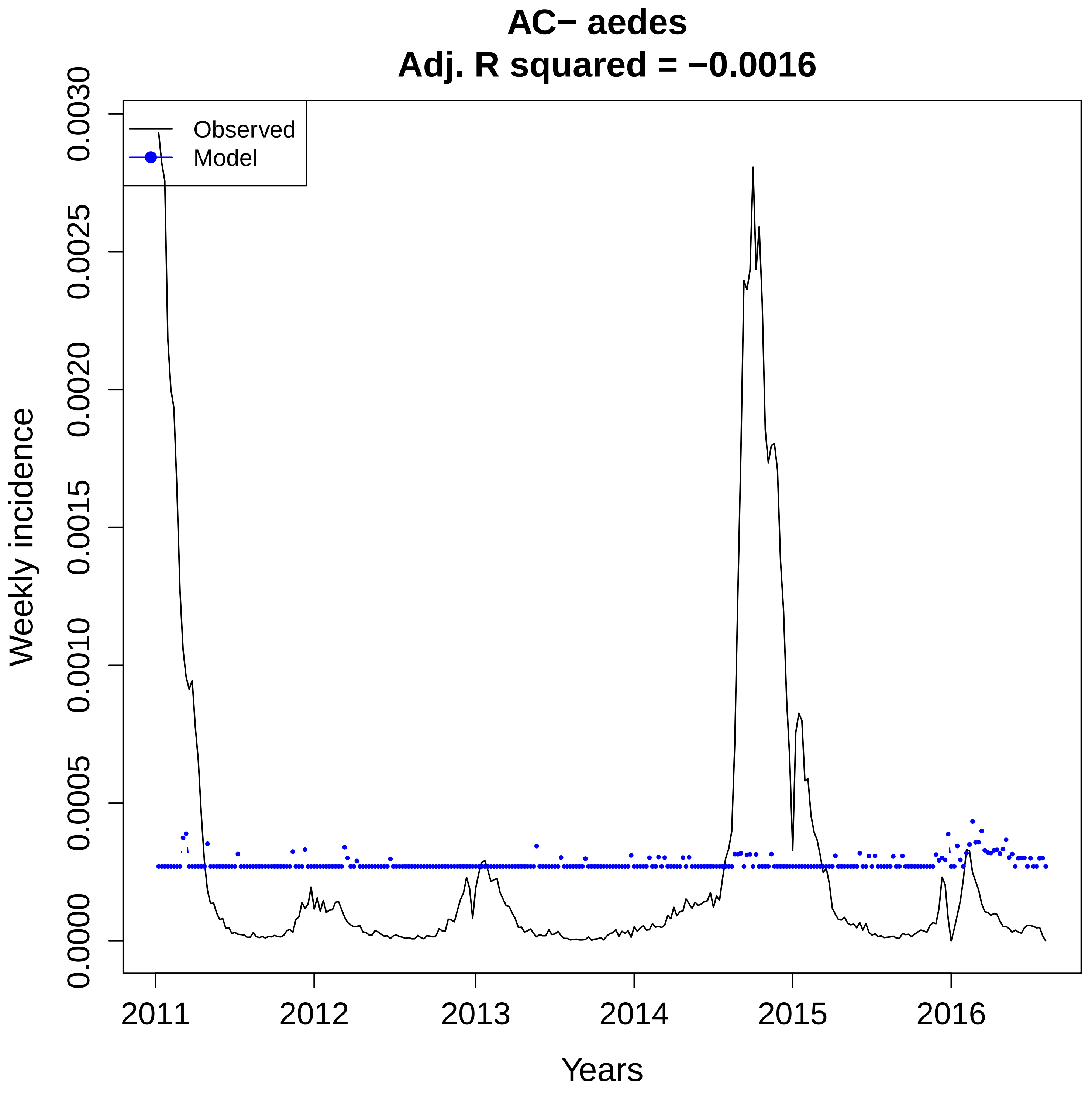
AC− aedes
Adj. R squared = −0.0016
Observed
Model
Weekly incidence
0.0000
0.0005
0.0010
0.0015
0.0020
0.0025
0.0030
2011
2012
2013
2014
2015
2016
Years

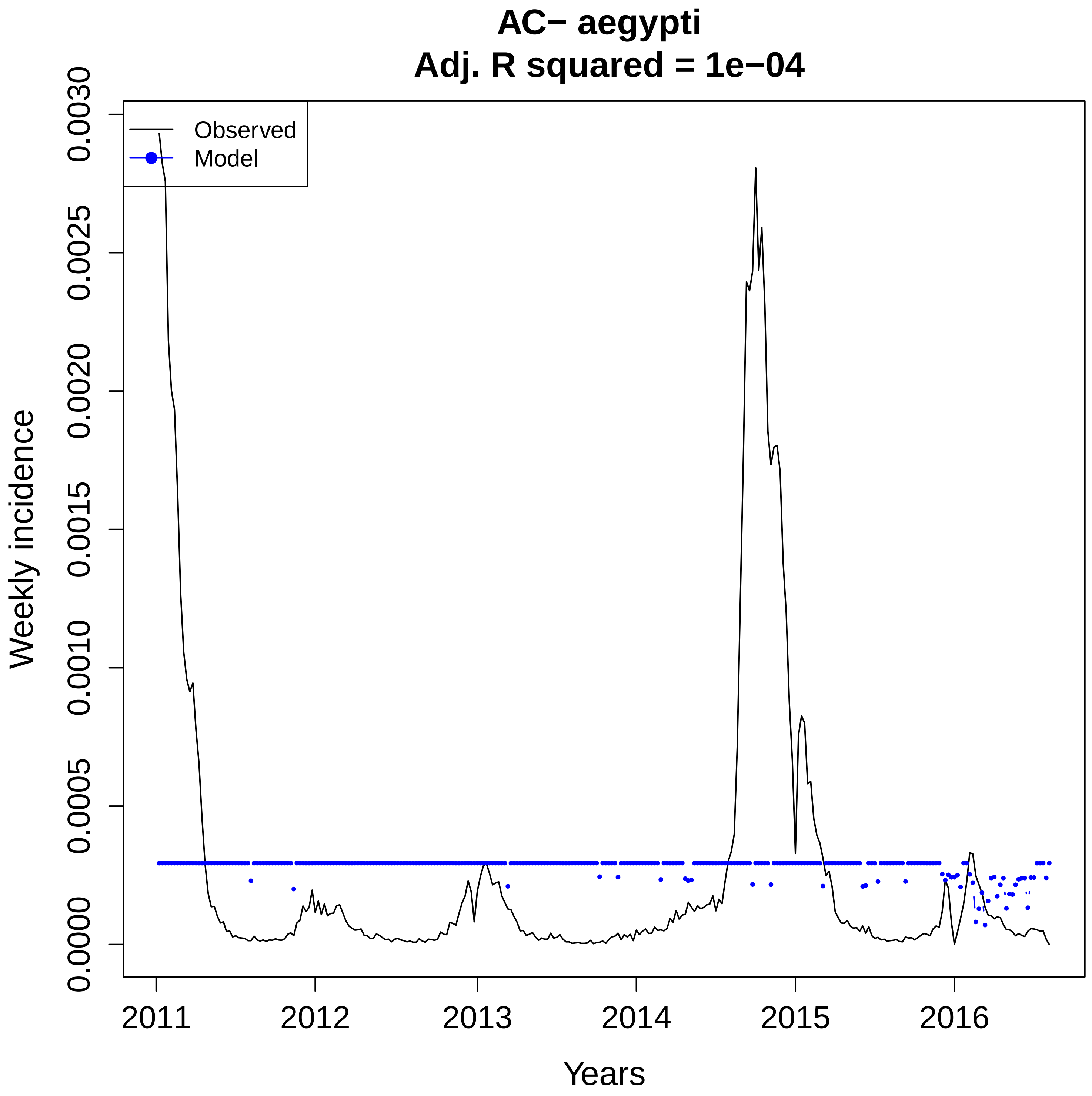

AC− aegypti
Adj. R squared = 1e−04
Observed
Model
Weekly incidence
0.0000
0.0005
0.0010
0.0015
0.0020
0.0025
0.0030
2011
2012
2013
2014
2015
2016
Years

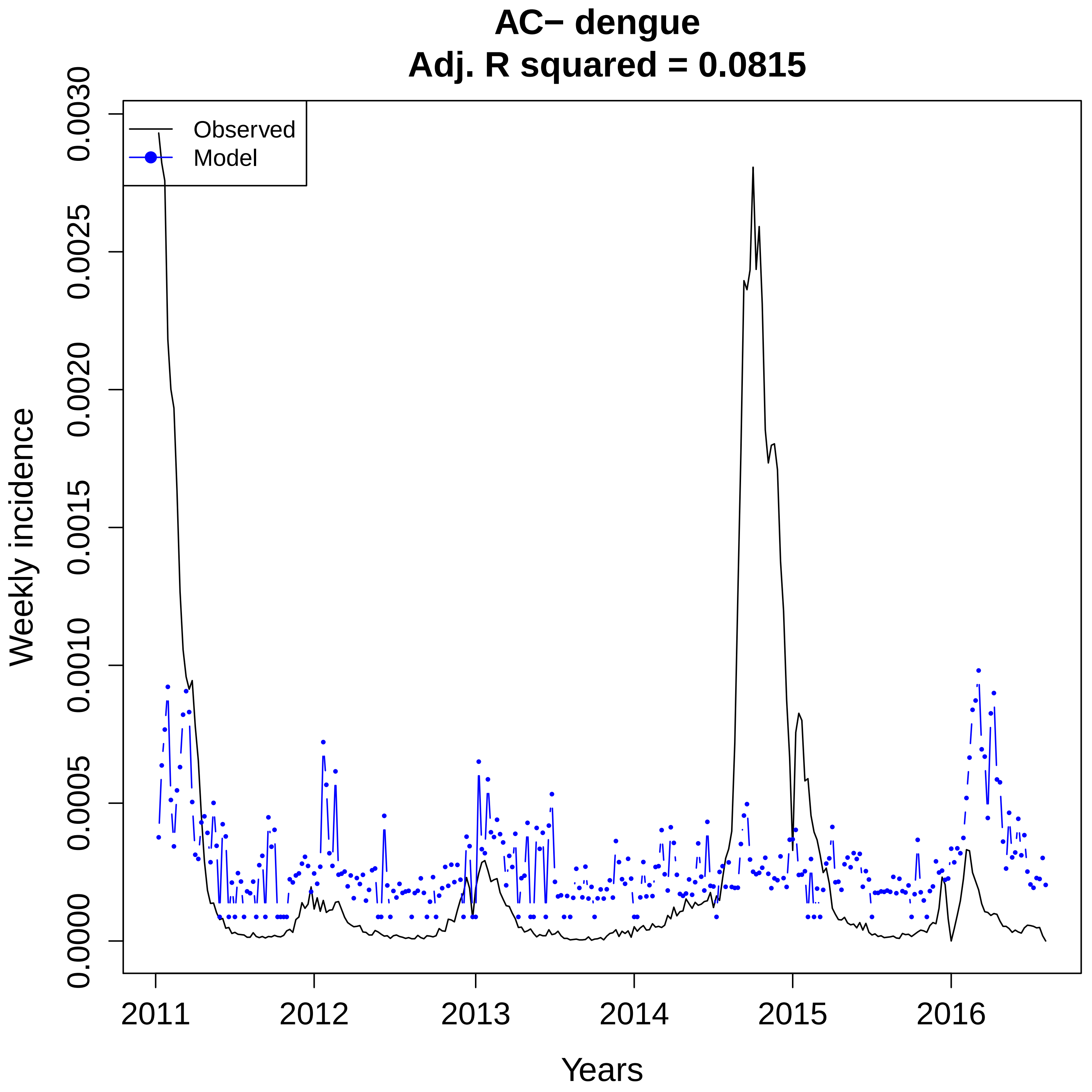
AC− dengue
Adj. R squared = 0.0815
Observed
Model
Weekly incidence
0.0000
0.0005
0.0010
0.0015
0.0020
0.0025
0.0030
2011
2012
2013
2014
2015
2016
Years

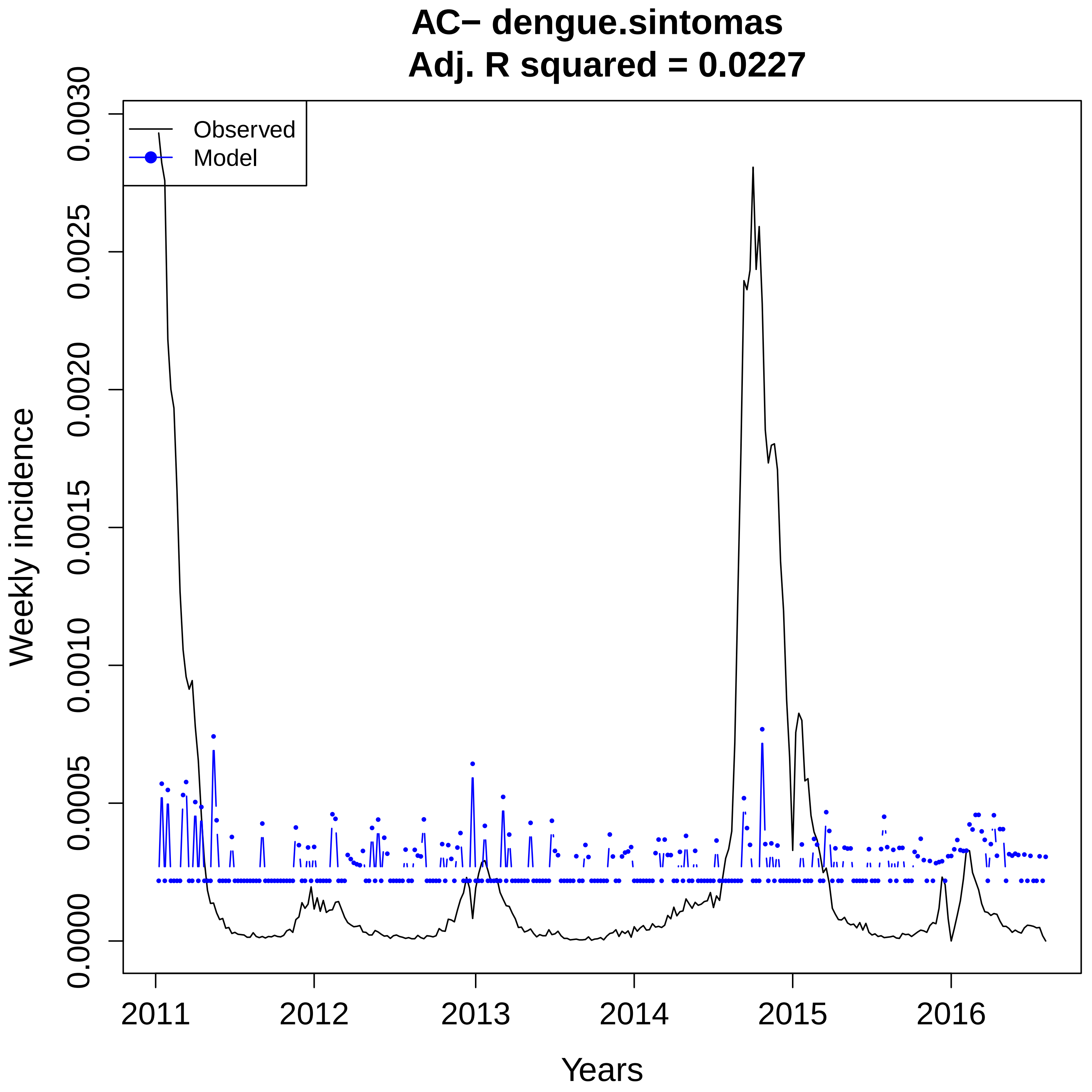
AC− dengue.sintomas
Adj. R squared = 0.0227
Observed
Model
Weekly incidence
0.0000
0.0005
0.0010
0.0015
0.0020
0.0025
0.0030
2011
2012
2013
2014
2015
2016
Years

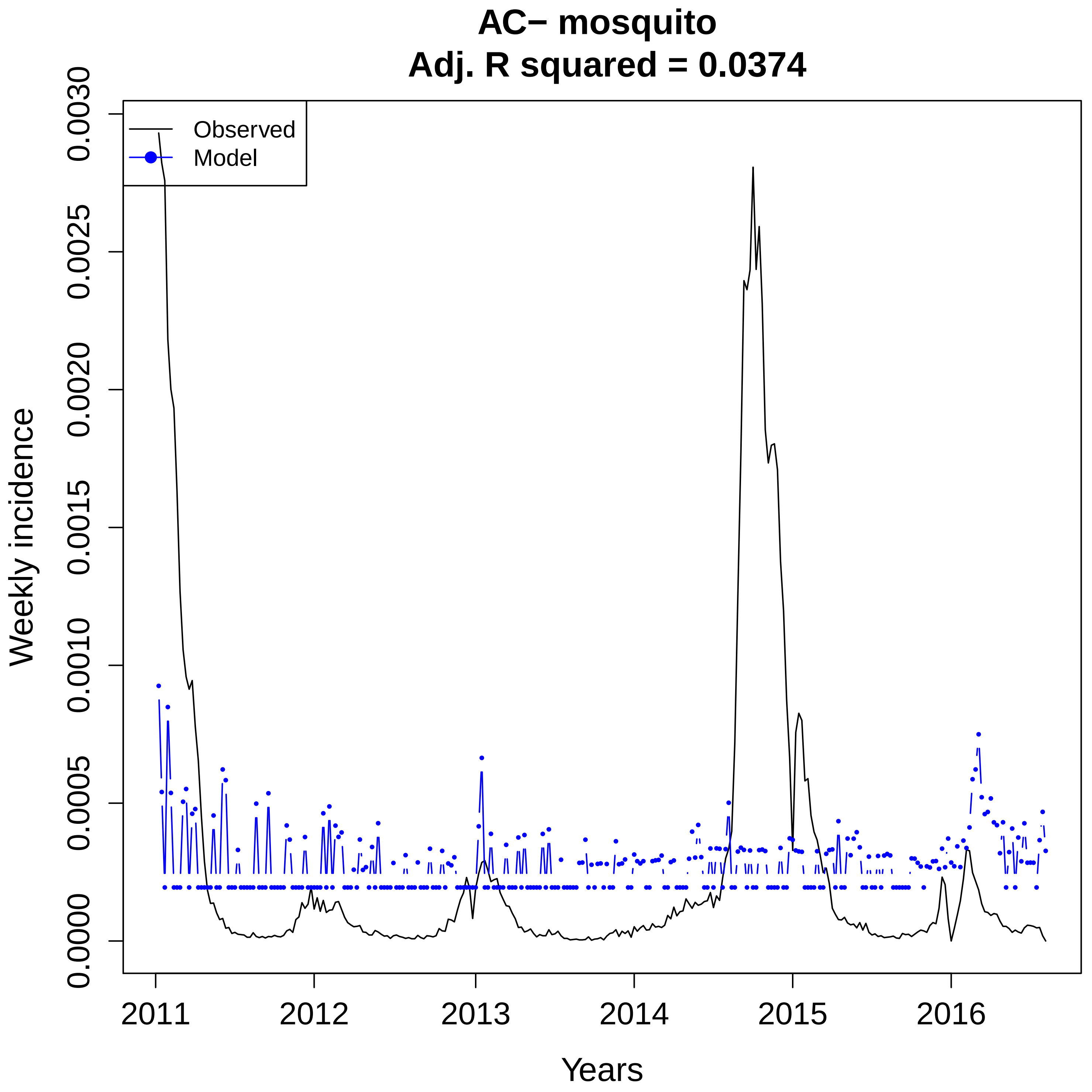

AC− mosquito
Adj. R squared = 0.0374
Observed
Model
Weekly incidence
0.0000
0.0005
0.0010
0.0015
0.0020
0.0025
0.0030
2011
2012
2013
2014
2015
2016
Years

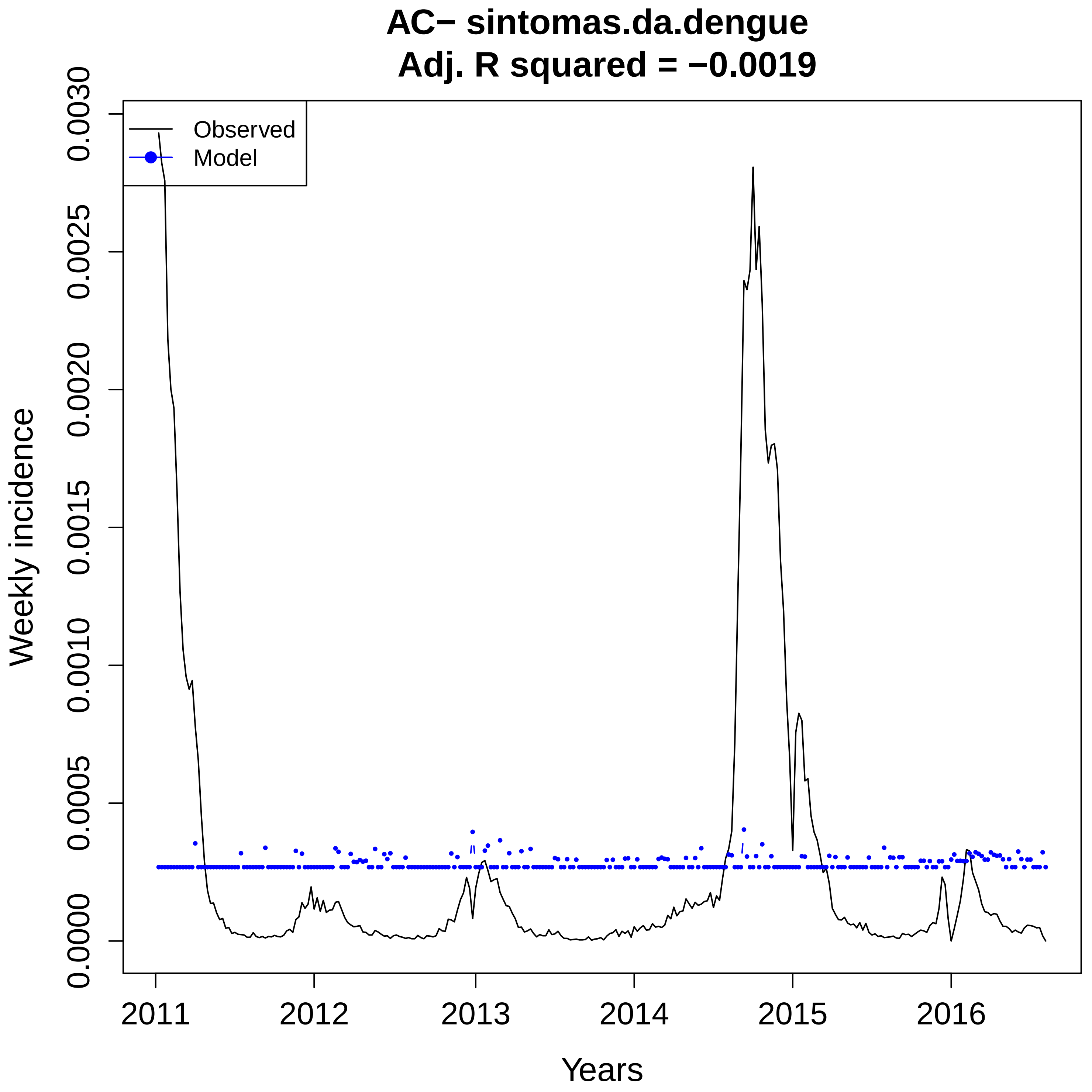
AC− sintomas.da.dengue
Adj. R squared = −0.0019
Observed
Model
Weekly incidence
0.0000
0.0005
0.0010
0.0015
0.0020
0.0025
0.0030
2011
2012
2013
2014
2015
2016
Years

Alagoas (AL)

Dengue incidence vs. Google Health Trends (2011–2016)

Definitions:

- **Combined terms:**
  - **All:** model with all the available terms. n= number of terms
  - **Four:** model with "dengue", "dengue sintomas", "aedes", and "mosquito".
  - **Uncorrelated:** model with non-correlated terms. n = number of uncorrelated terms
- **Individual terms:** Plots developed with one term at a time.

- Terms for all and uncorrelated models can be found in additional file 3.

Combined terms
Adjusted R squared

Alagoas (AL)

All
(n = 8)

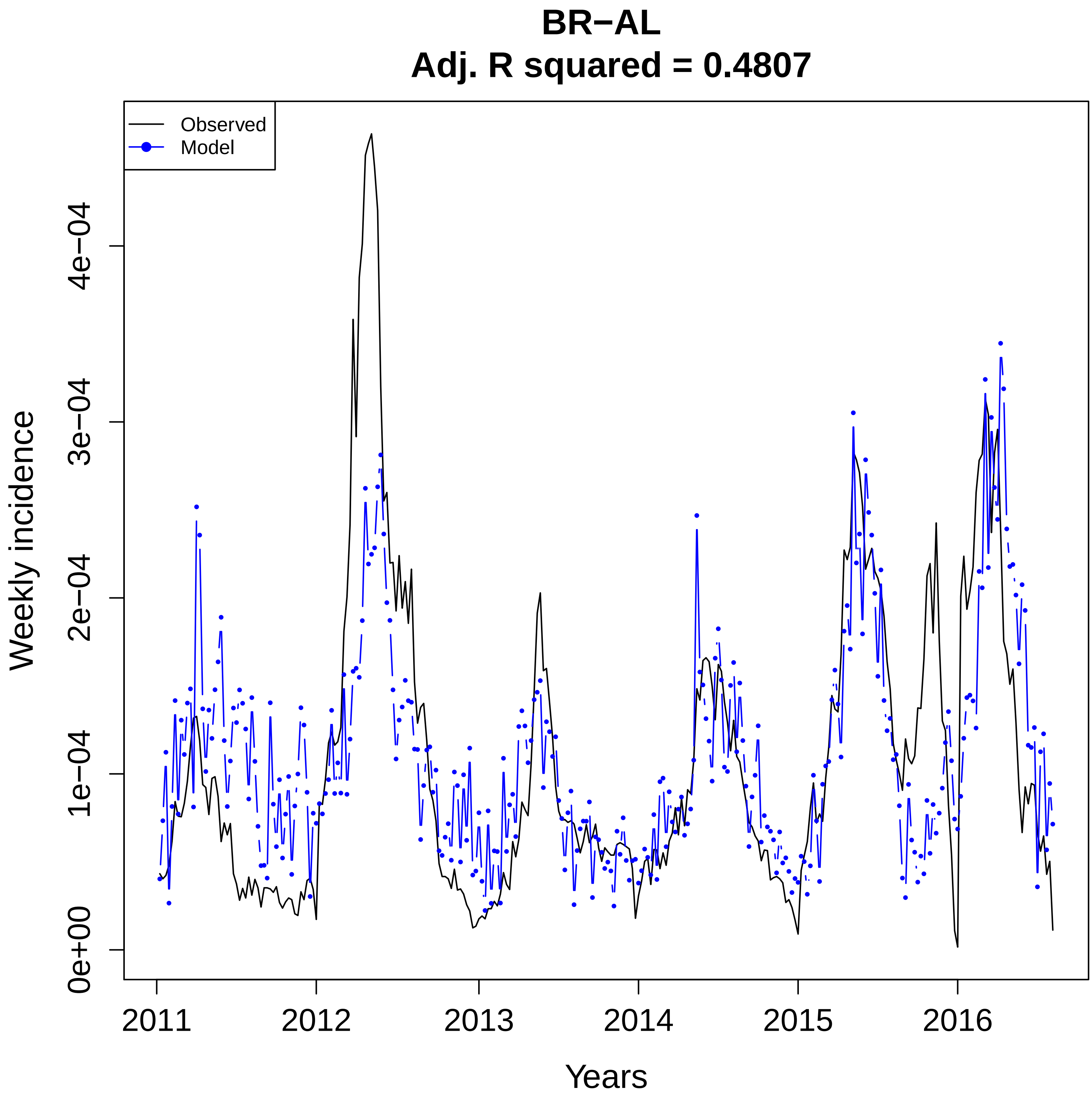
BR−AL
Adj. R squared = 0.4807
Observed
Model
Weekly incidence
0e+00
1e−04
2e−04
3e−04
4e−04
2011
2012
2013
2014
2015
2016
Years

Four

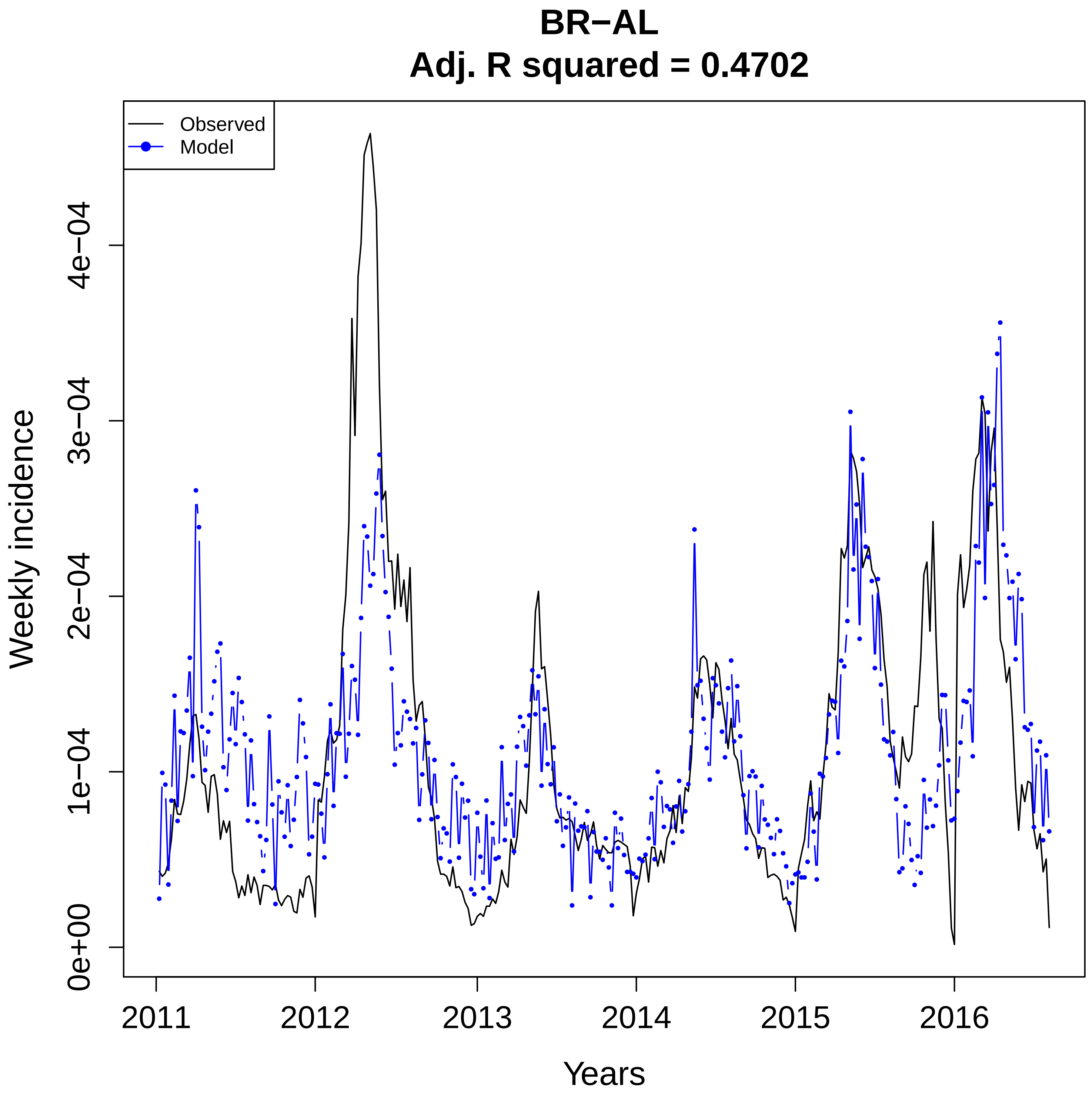

BR−AL
Adj. R squared = 0.4702
Observed
Model
Weekly incidence
0e+00
1e−04
2e−04
3e−04
4e−04
2011
2012
2013
2014
2015
2016
Years

Uncorrelated
(n = 3)

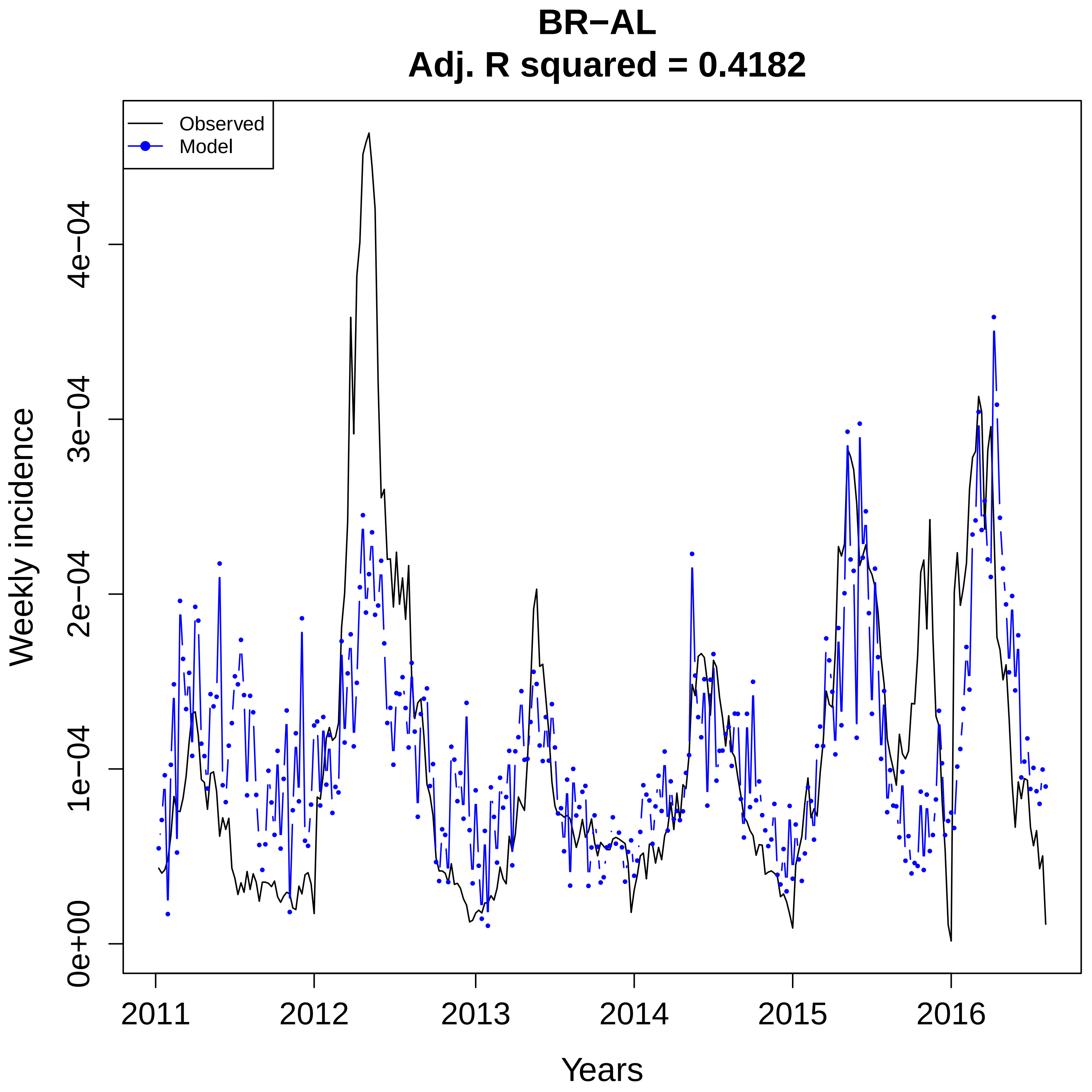

Individual terms
Adjusted R squared

Alagoas (AL)

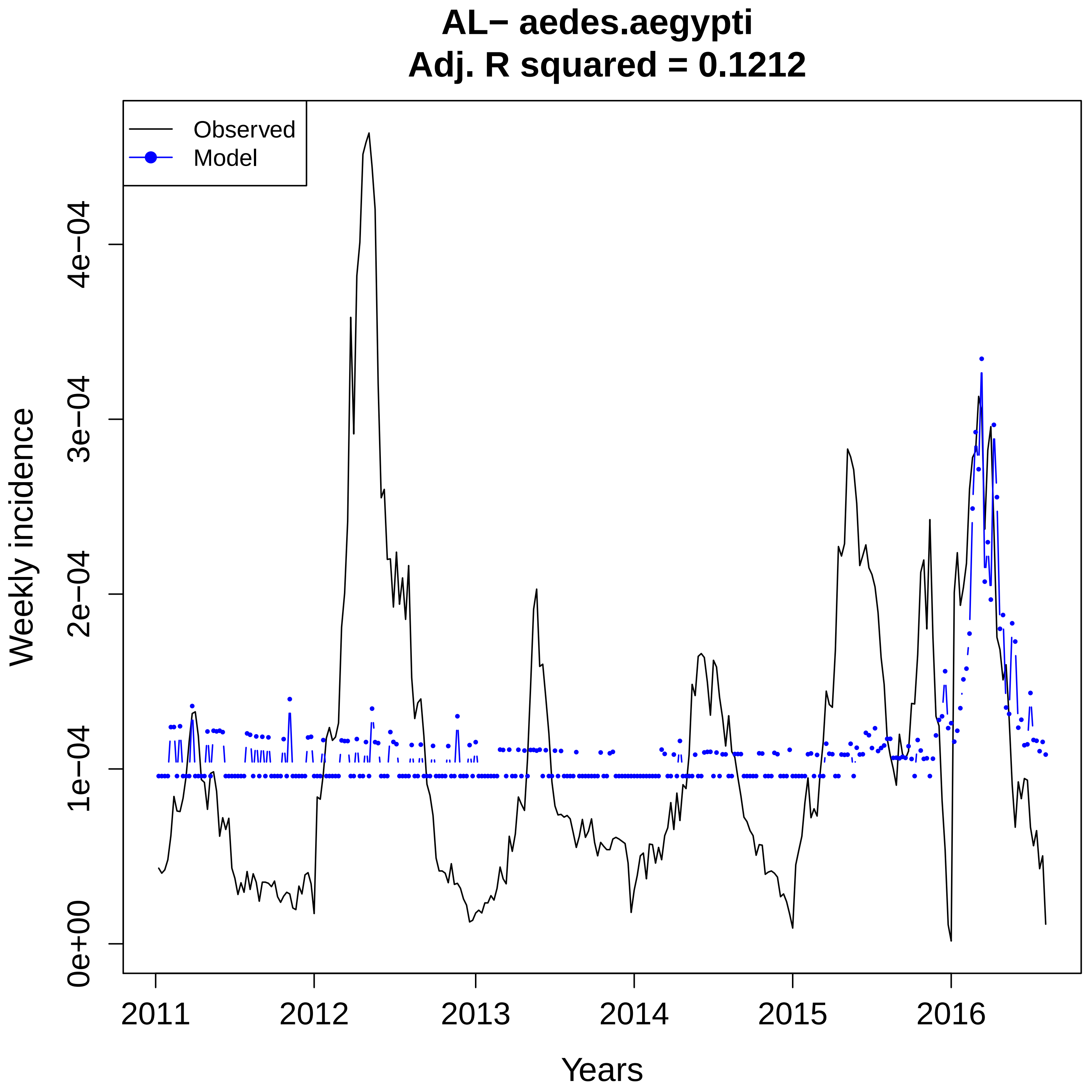
AL− aedes.aegypti
Adj. R squared = 0.1212
Observed
Model
Weekly incidence
0e+00
1e−04
2e−04
3e−04
4e−04
2011
2012
2013
2014
2015
2016
Years

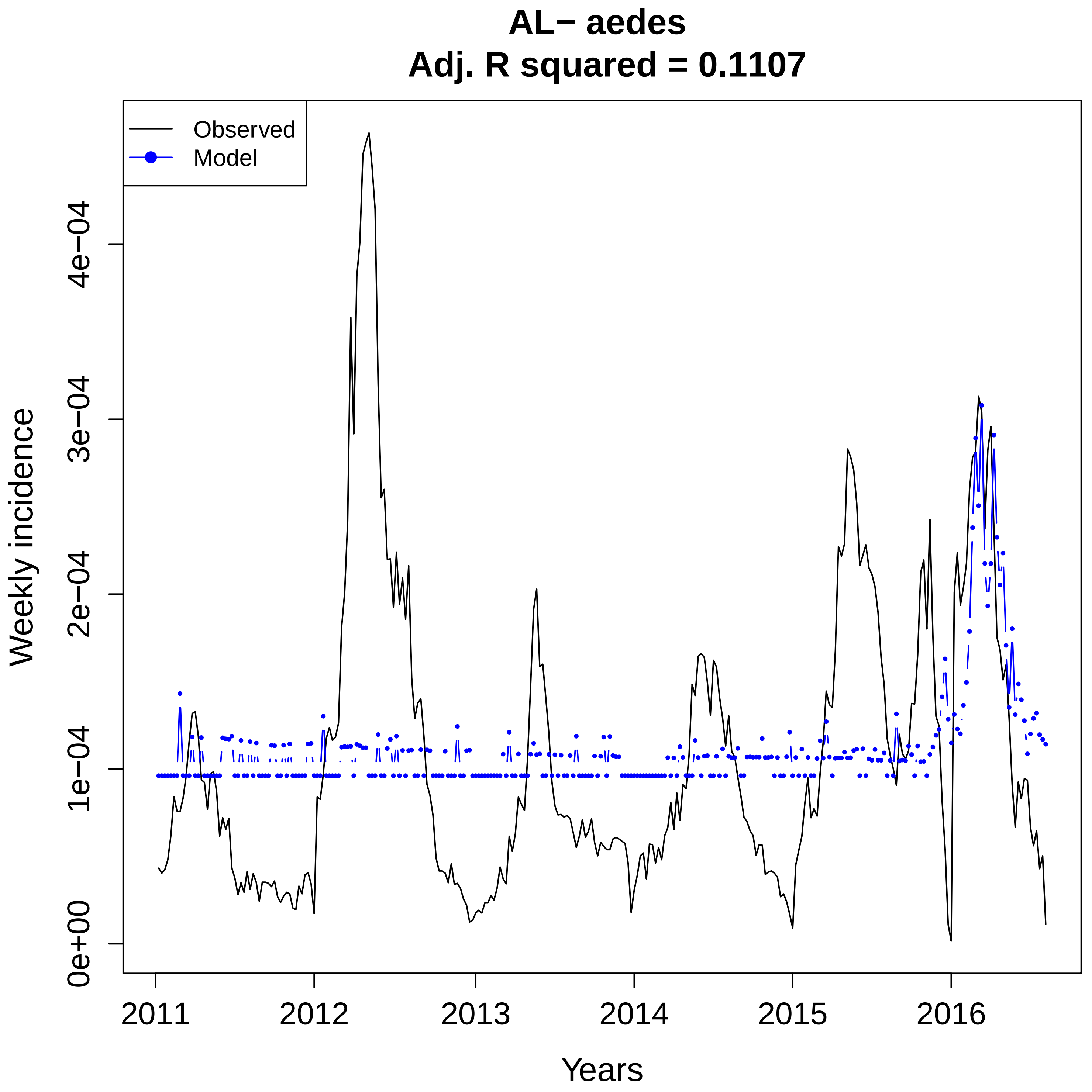

AL− aedes
Adj. R squared = 0.1107
Observed
Model
Weekly incidence
0e+00
1e−04
2e−04
3e−04
4e−04
2011
2012
2013
2014
2015
2016
Years

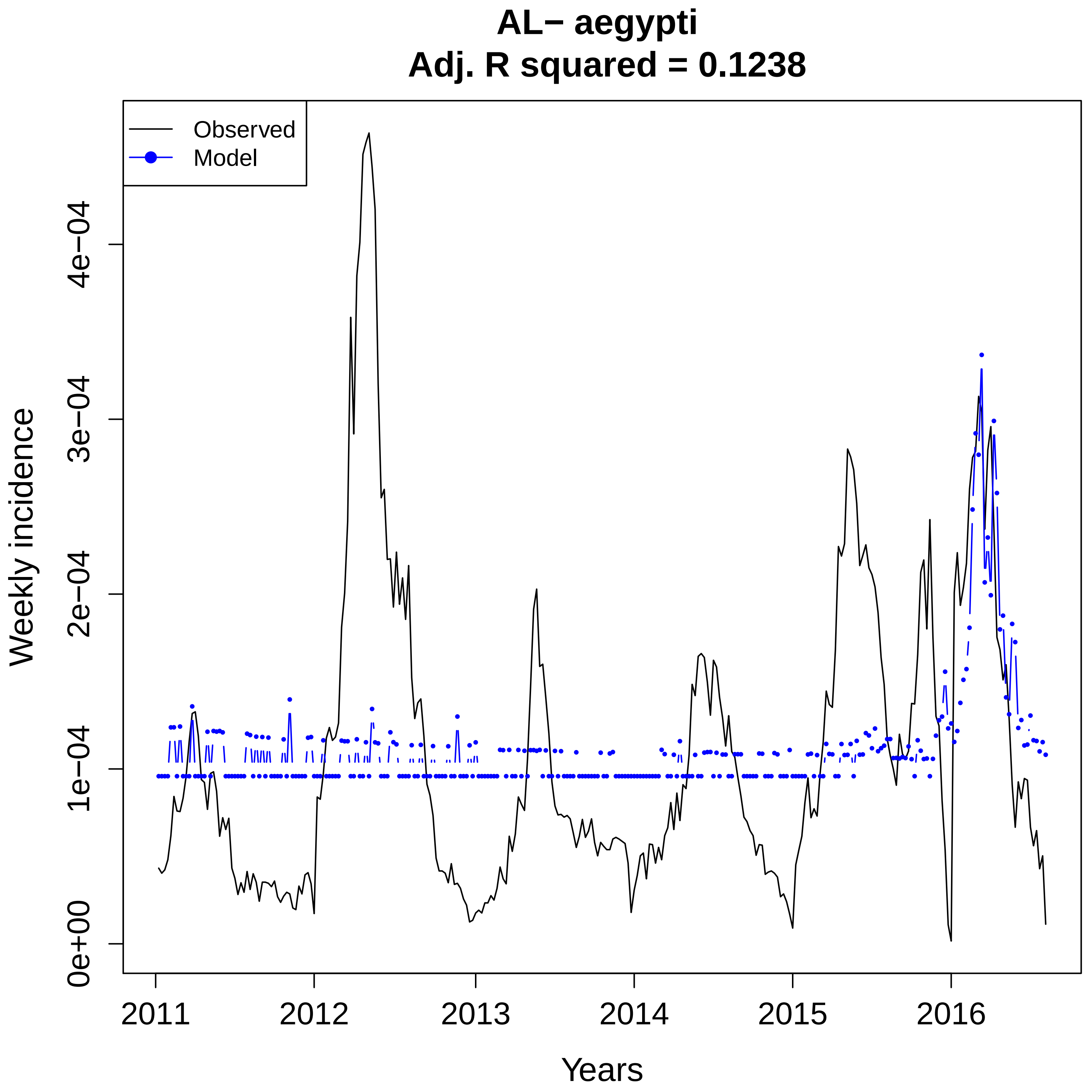
AL− aegypti
Adj. R squared = 0.1238
Observed
Model
Weekly incidence
0e+00
1e−04
2e−04
3e−04
4e−04
2011
2012
2013
2014
2015
2016
Years

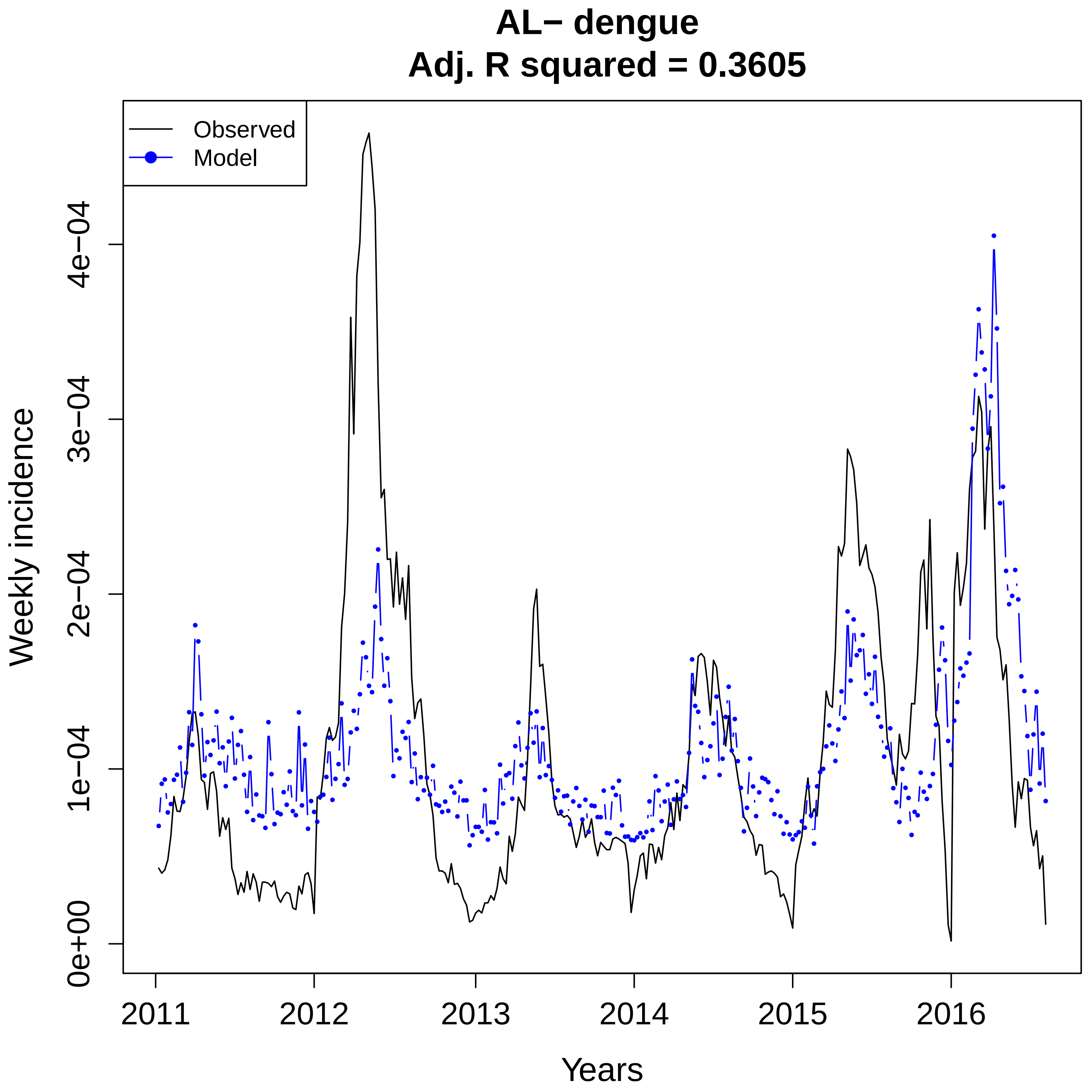
AL− dengue
Adj. R squared = 0.3605
Observed
Model
Weekly incidence
0e+00
1e−04
2e−04
3e−04
4e−04
2011
2012
2013
2014
2015
2016
Years

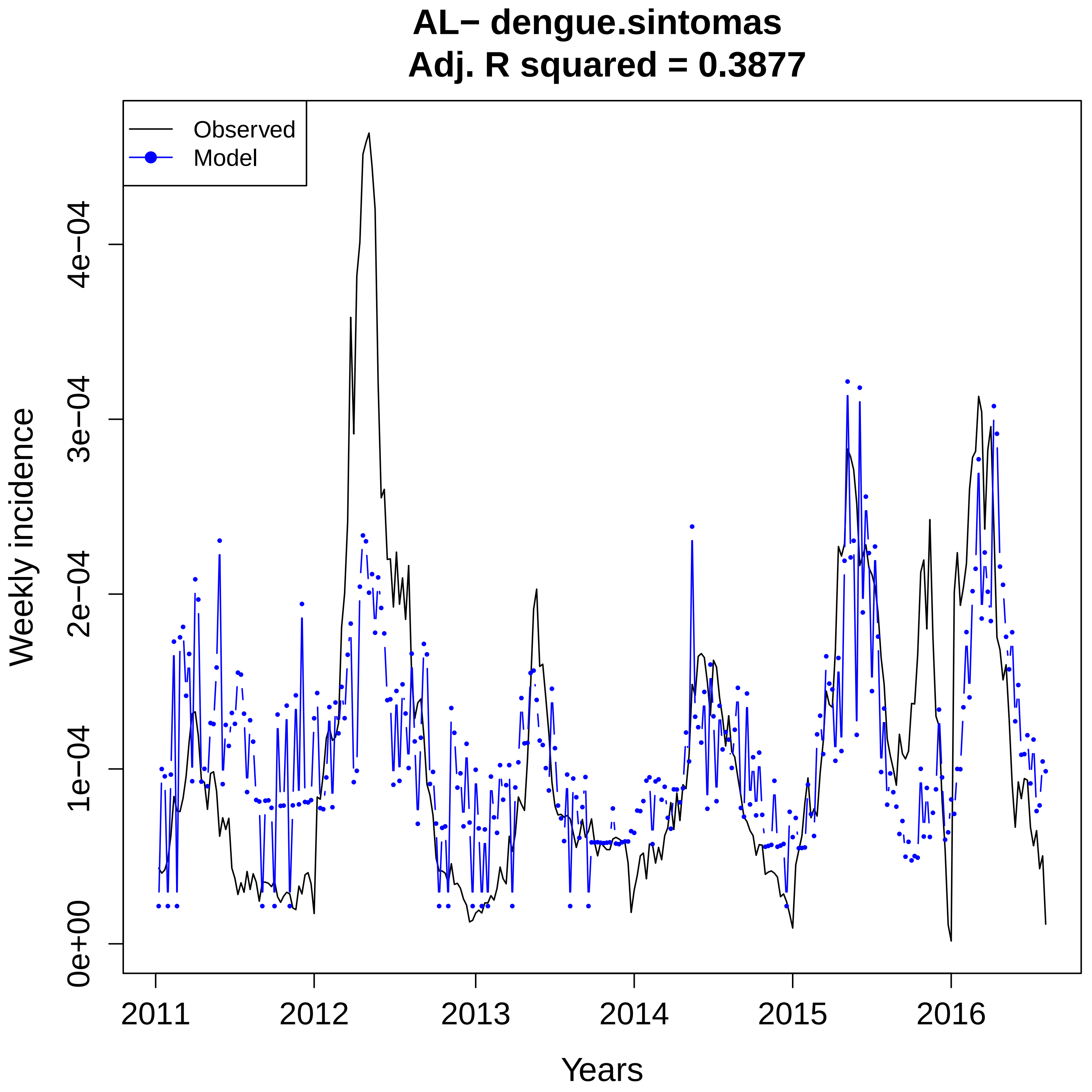
AL− dengue.sintomas
Adj. R squared = 0.3877
Observed
Model
Weekly incidence
0e+00
1e−04
2e−04
3e−04
4e−04
2011
2012
2013
2014
2015
2016
Years

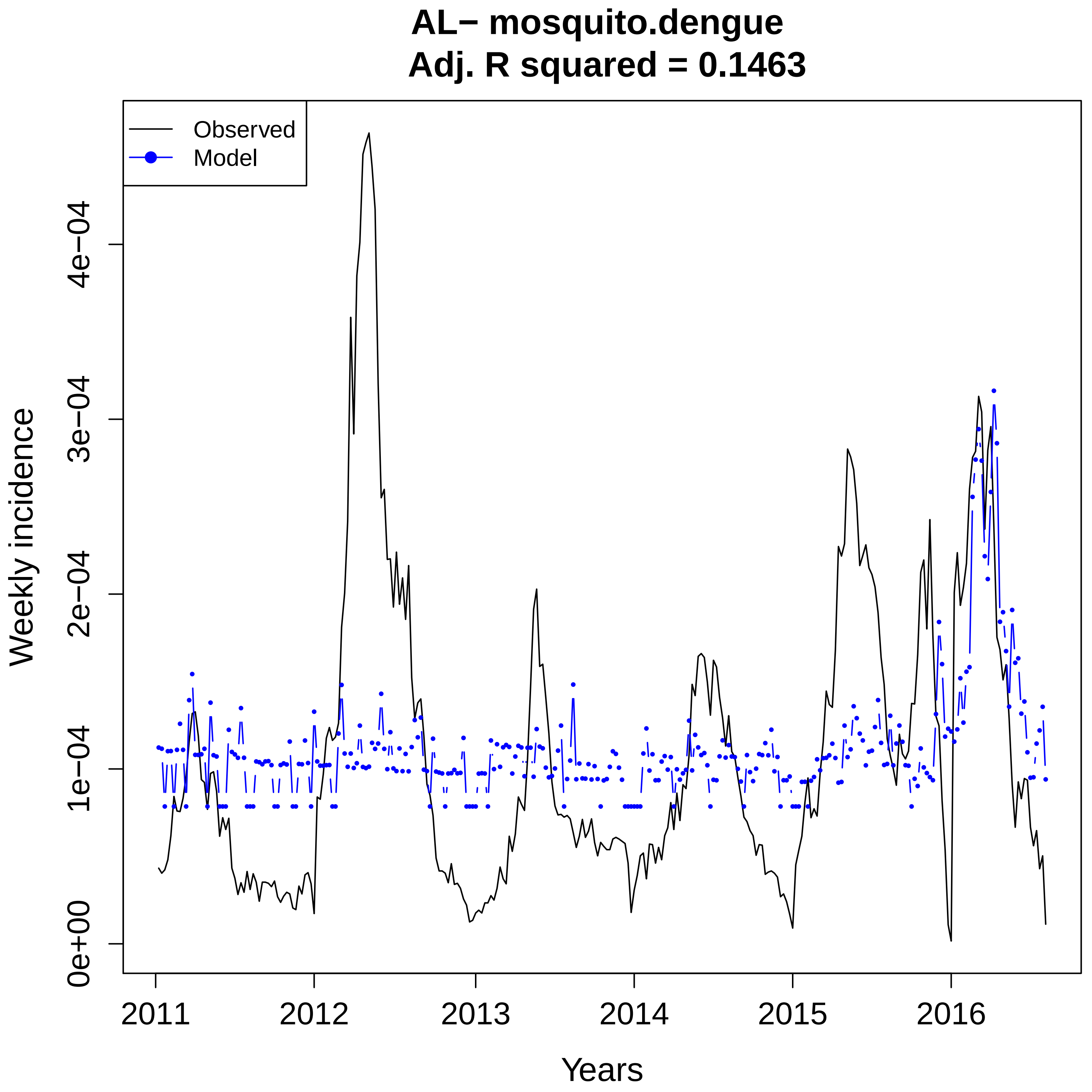
AL− mosquito.dengue
Adj. R squared = 0.1463
Observed
Model
Weekly incidence
0e+00
1e−04
2e−04
3e−04
4e−04
2011
2012
2013
2014
2015
2016
Years

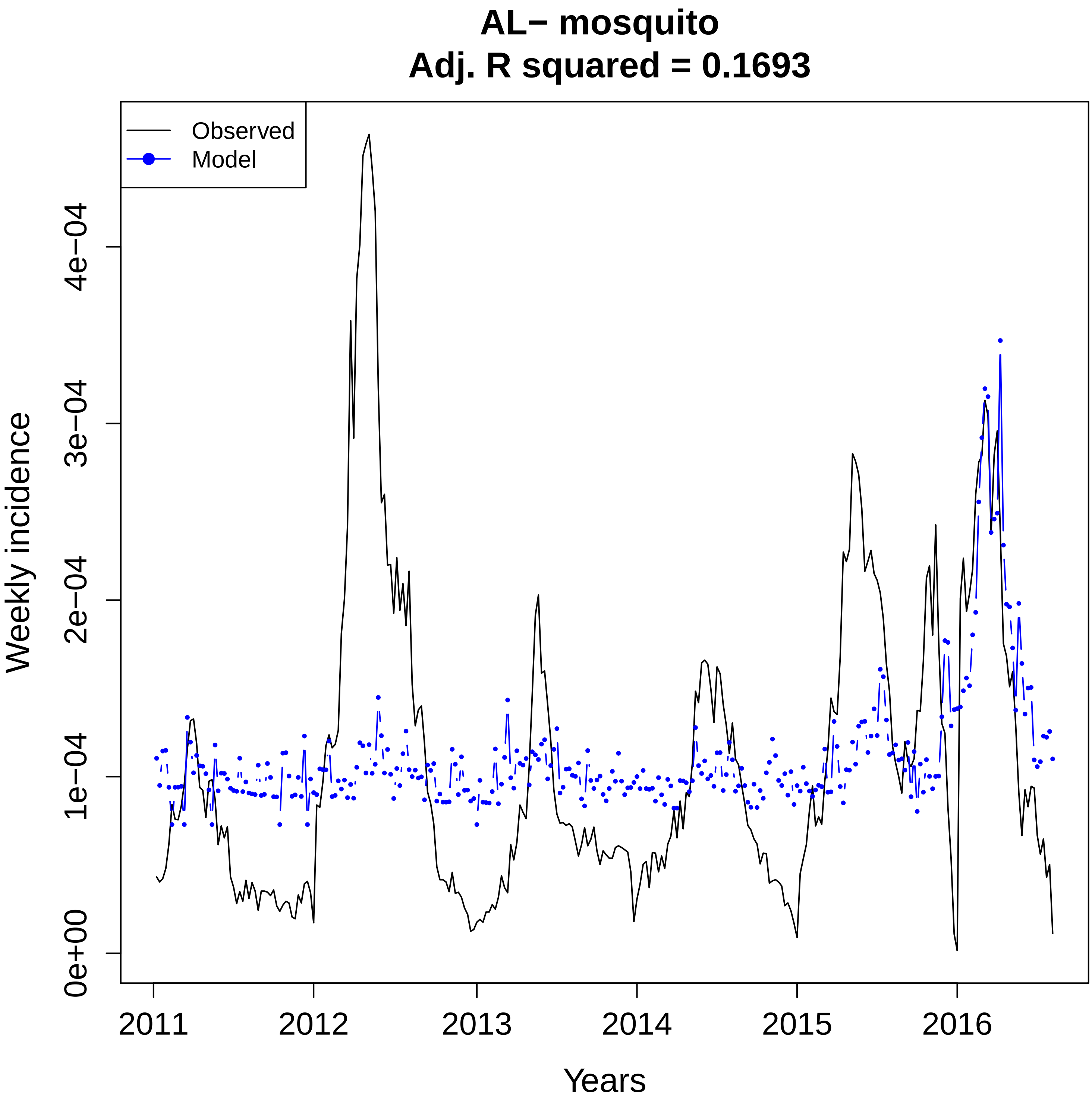
AL− mosquito
Adj. R squared = 0.1693
Observed
Model
Weekly incidence
0e+00
1e−04
2e−04
3e−04
4e−04
2011
2012
2013
2014
2015
2016
Years

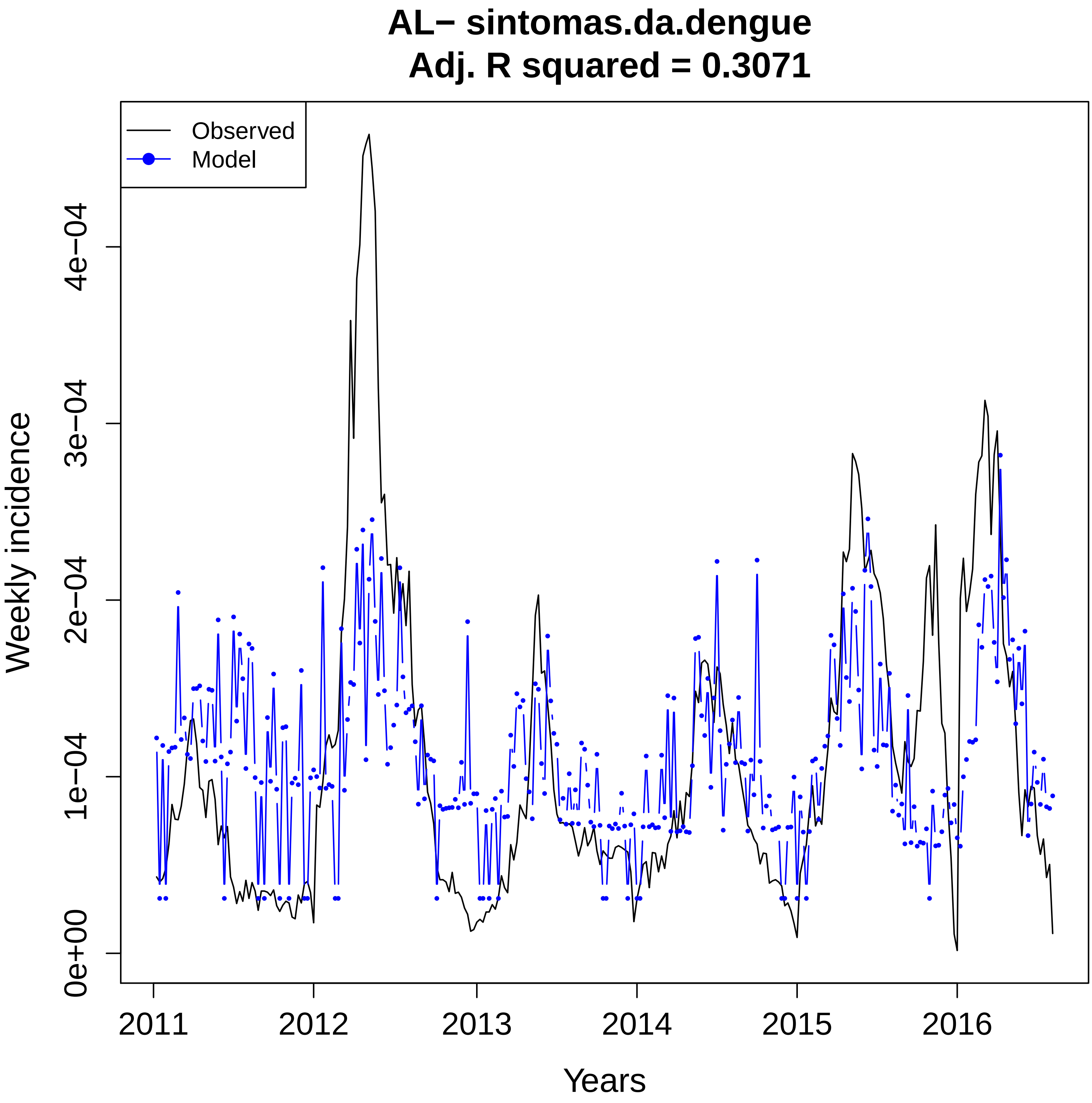

AL− sintomas.da.dengue
Adj. R squared = 0.3071
Observed
Model
Weekly incidence
0e+00
1e−04
2e−04
3e−04
4e−04
2011
2012
2013
2014
2015
2016
Years

Amazonas (AM)
Dengue incidence vs. Google Health Trends (2011–2016)

Definitions:

- **Combined terms:**
  - **All:** model with all the available terms. n= number of terms
  - **Four:** model with "dengue", "dengue sintomas", "aedes", and "mosquito".
  - **Uncorrelated:** model with non-correlated terms. n = number of uncorrelated terms
- **Individual terms:** Plots developed with one term at a time.

- Terms for all and uncorrelated models can be found in additional file 3.

Combined terms
Adjusted R squared

Amazonas (AM)

All
(n = 9)

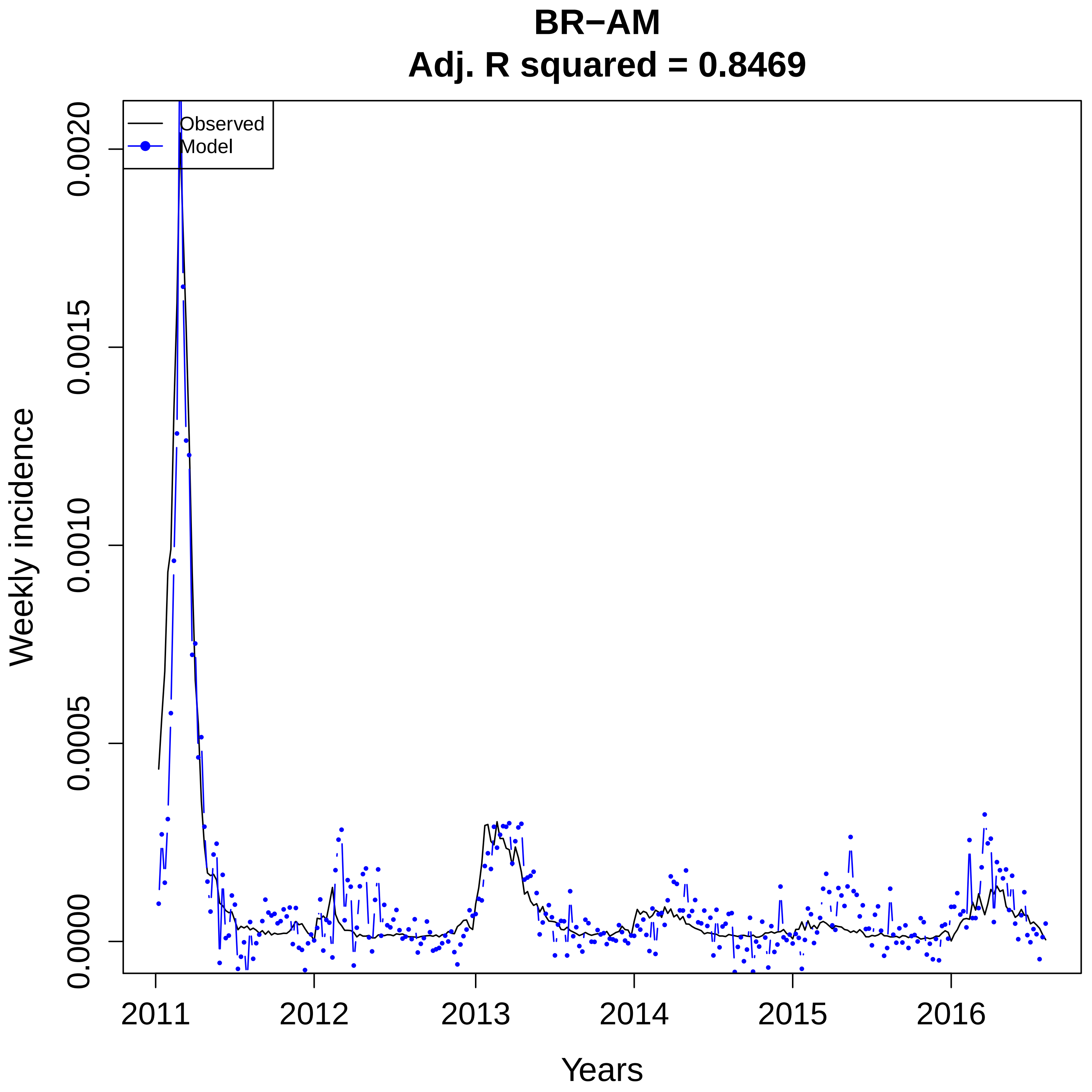
BR−AM
Adj. R squared = 0.8469
Observed
Model
Weekly incidence
0.0000
0.0005
0.0010
0.0015
0.0020
2011
2012
2013
2014
2015
2016
Years

Four

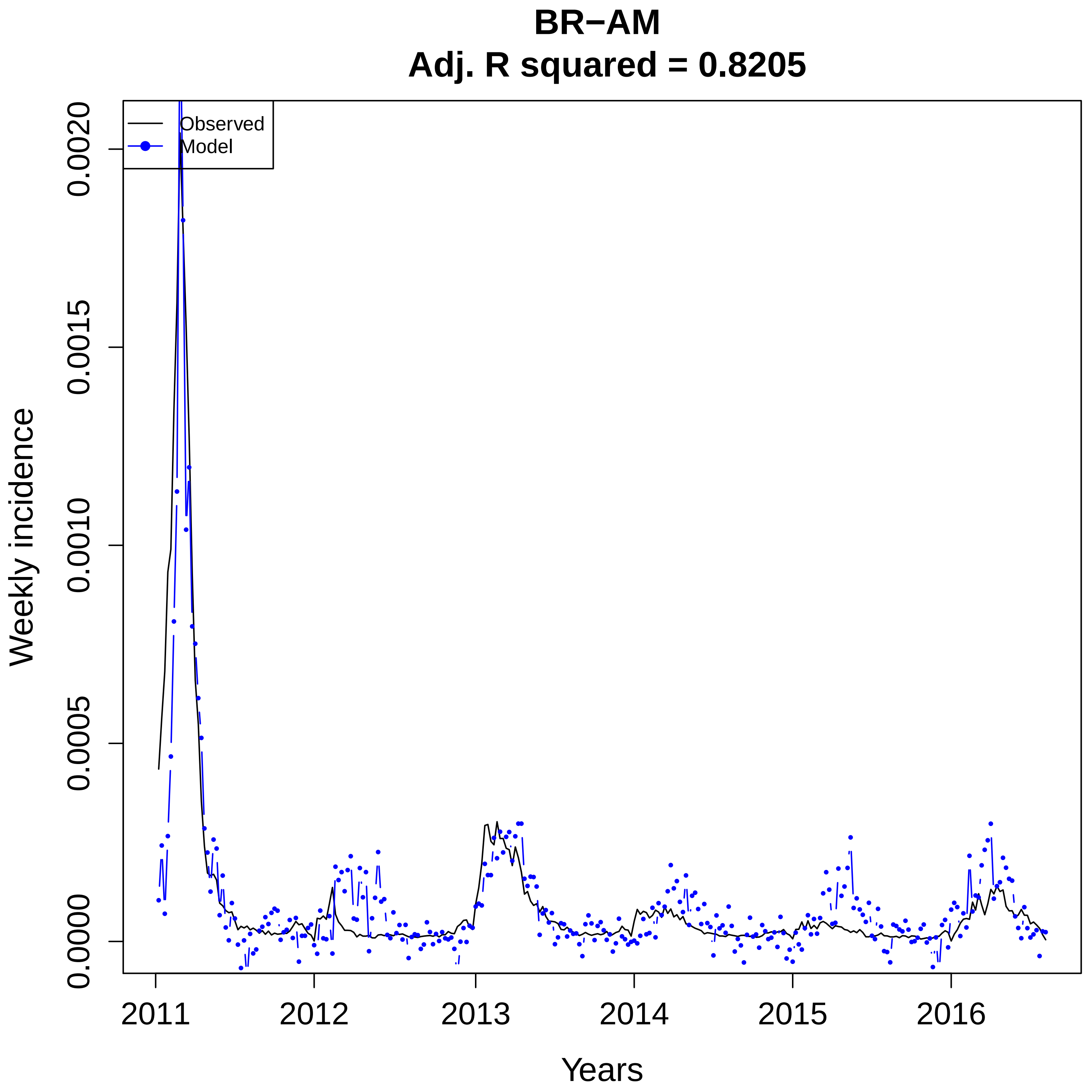

BR−AM
Adj. R squared = 0.8205
Observed
Model
Weekly incidence
0.0000
0.0005
0.0010
0.0015
0.0020
2011
2012
2013
2014
2015
2016
Years

Uncorrelated
(n = 2)

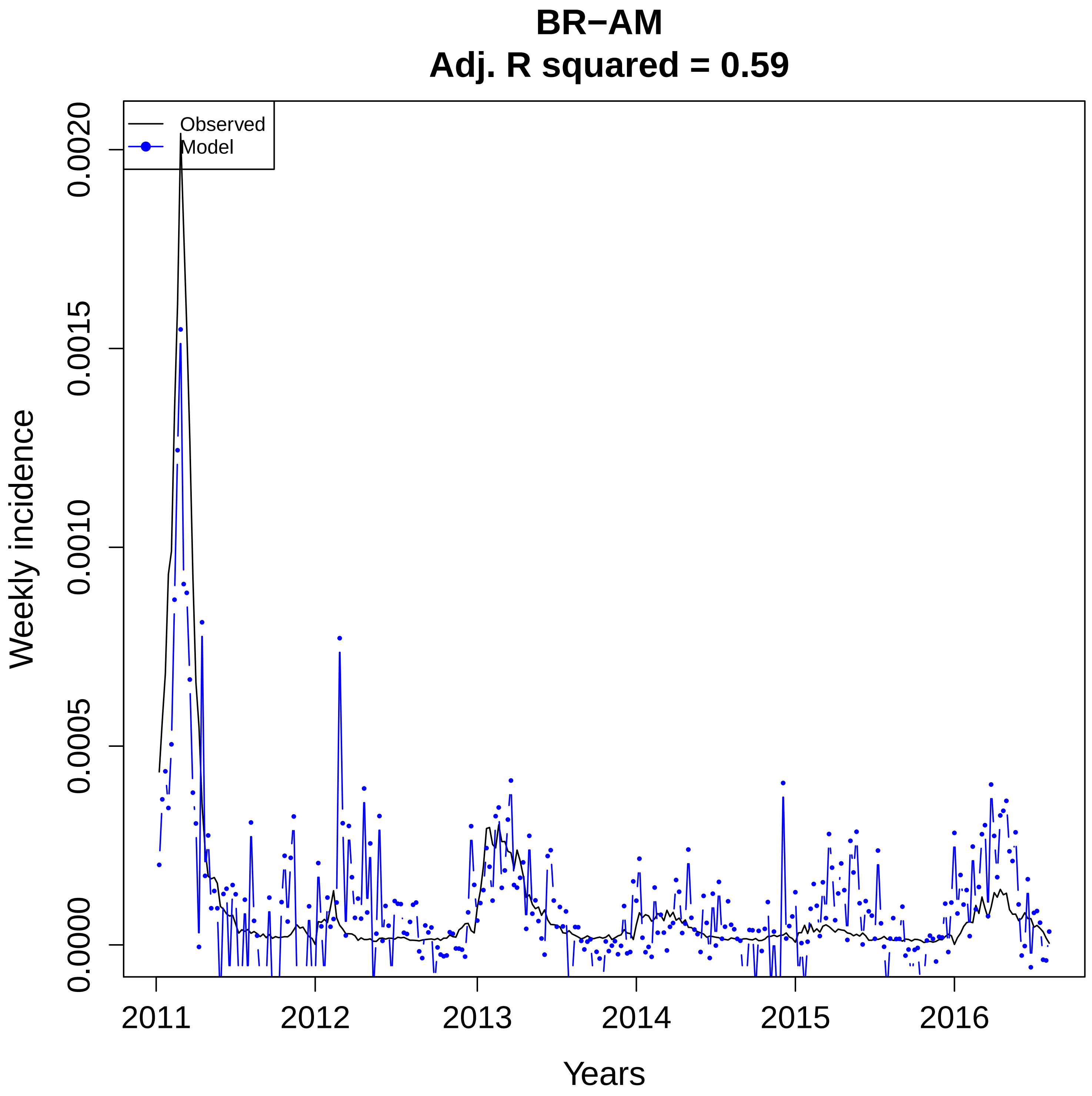

Individual terms
Adjusted R squared

Amazonas (AM)

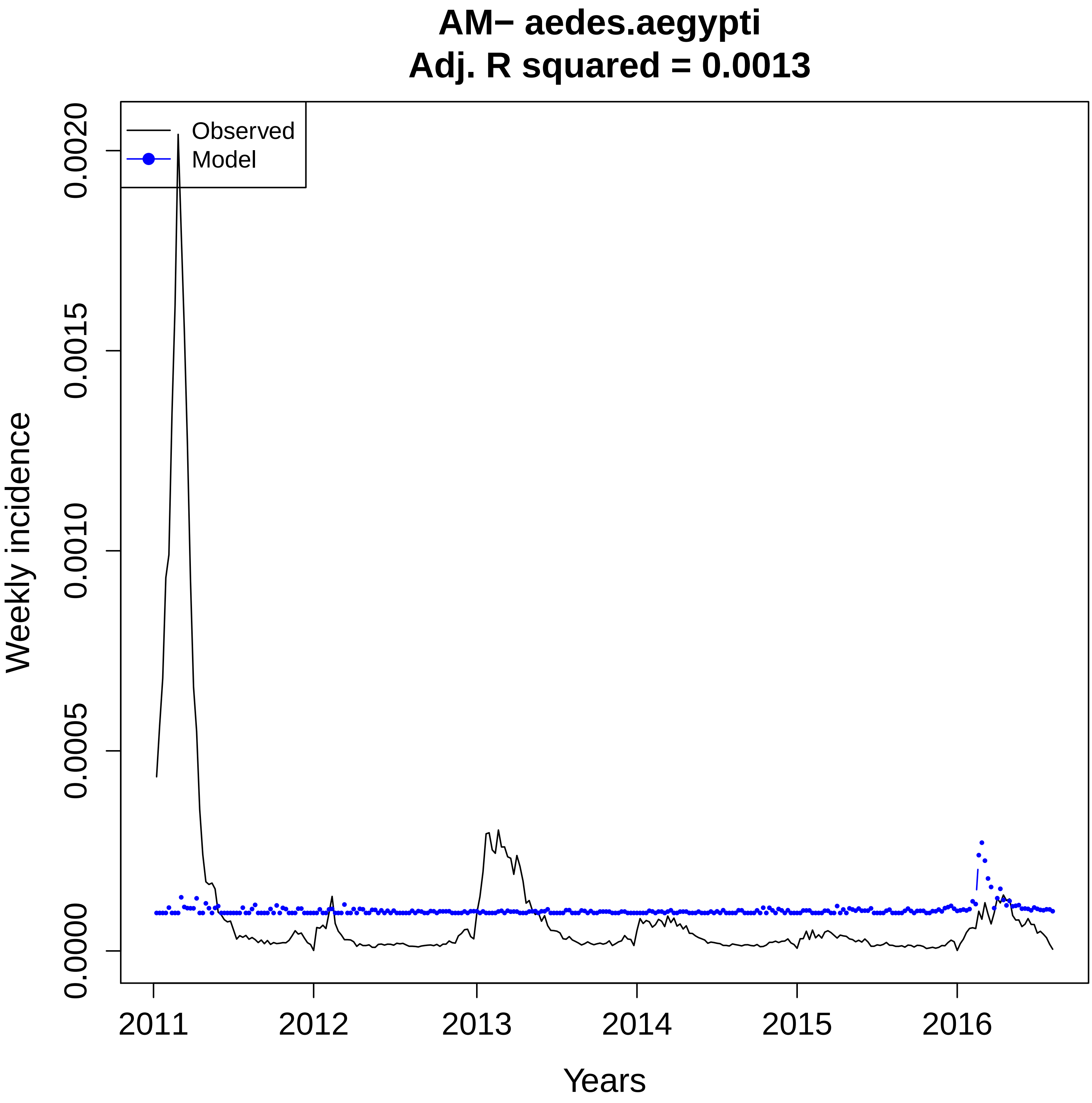
AM− aedes.aegypti
Adj. R squared = 0.0013
Observed
Model
Weekly incidence
0.0000
0.0005
0.0010
0.0015
0.0020
2011
2012
2013
2014
2015
2016
Years

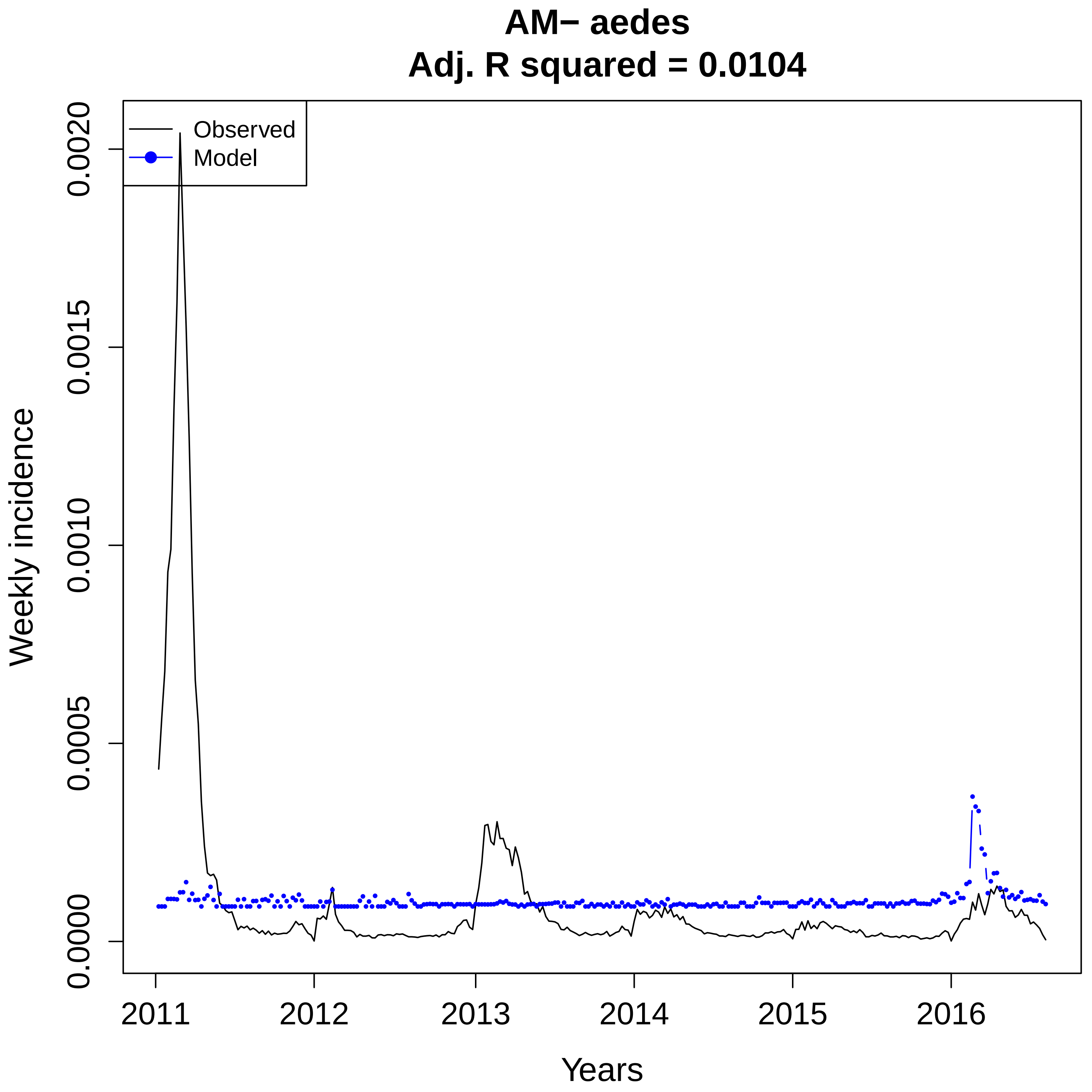
AM− aedes
Adj. R squared = 0.0104
Observed
Model
Weekly incidence
0.0000
0.0005
0.0010
0.0015
0.0020
2011
2012
2013
2014
2015
2016
Years

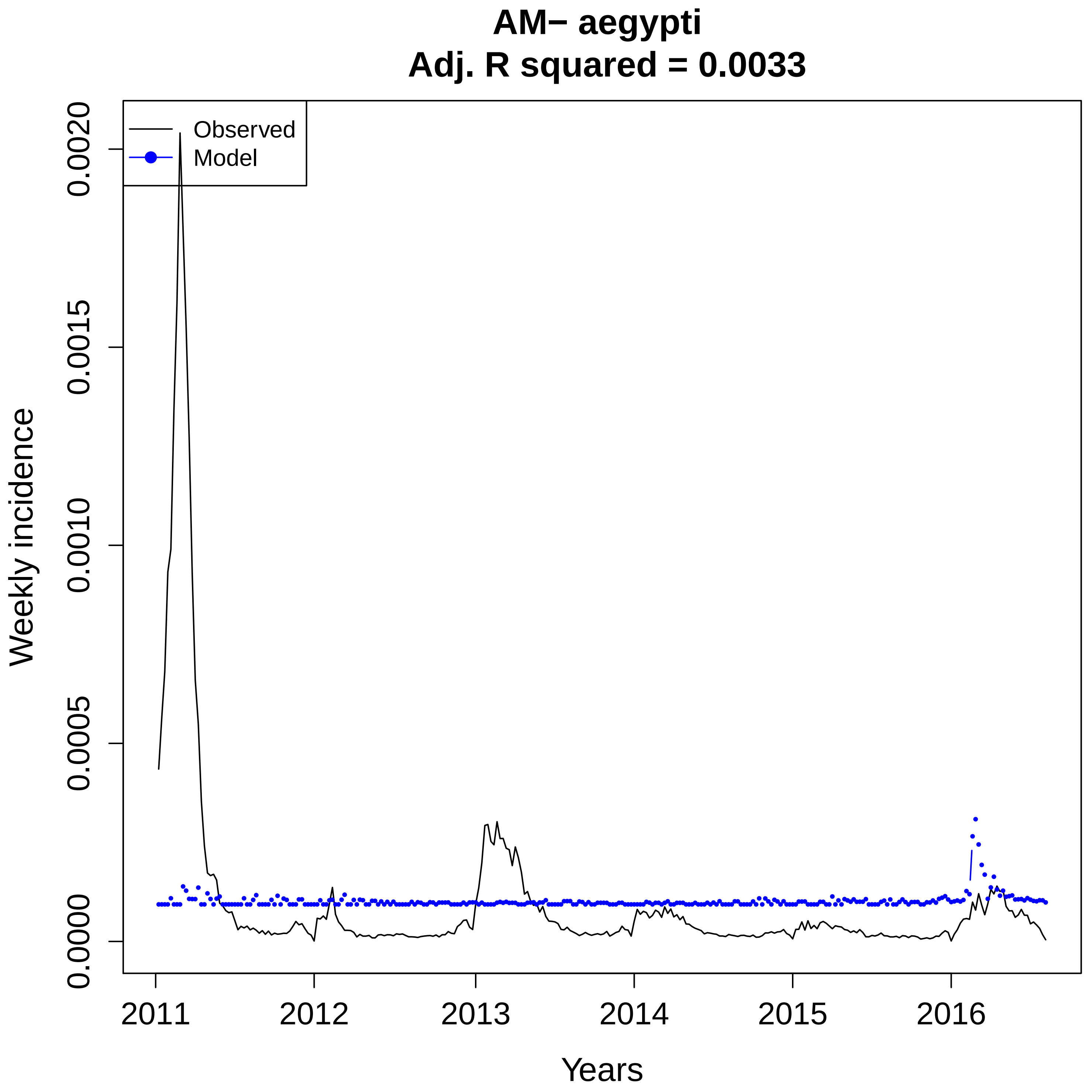

AM− aegypti
Adj. R squared = 0.0033
Observed
Model
Weekly incidence
0.0000
0.0005
0.0010
0.0015
0.0020
2011
2012
2013
2014
2015
2016
Years

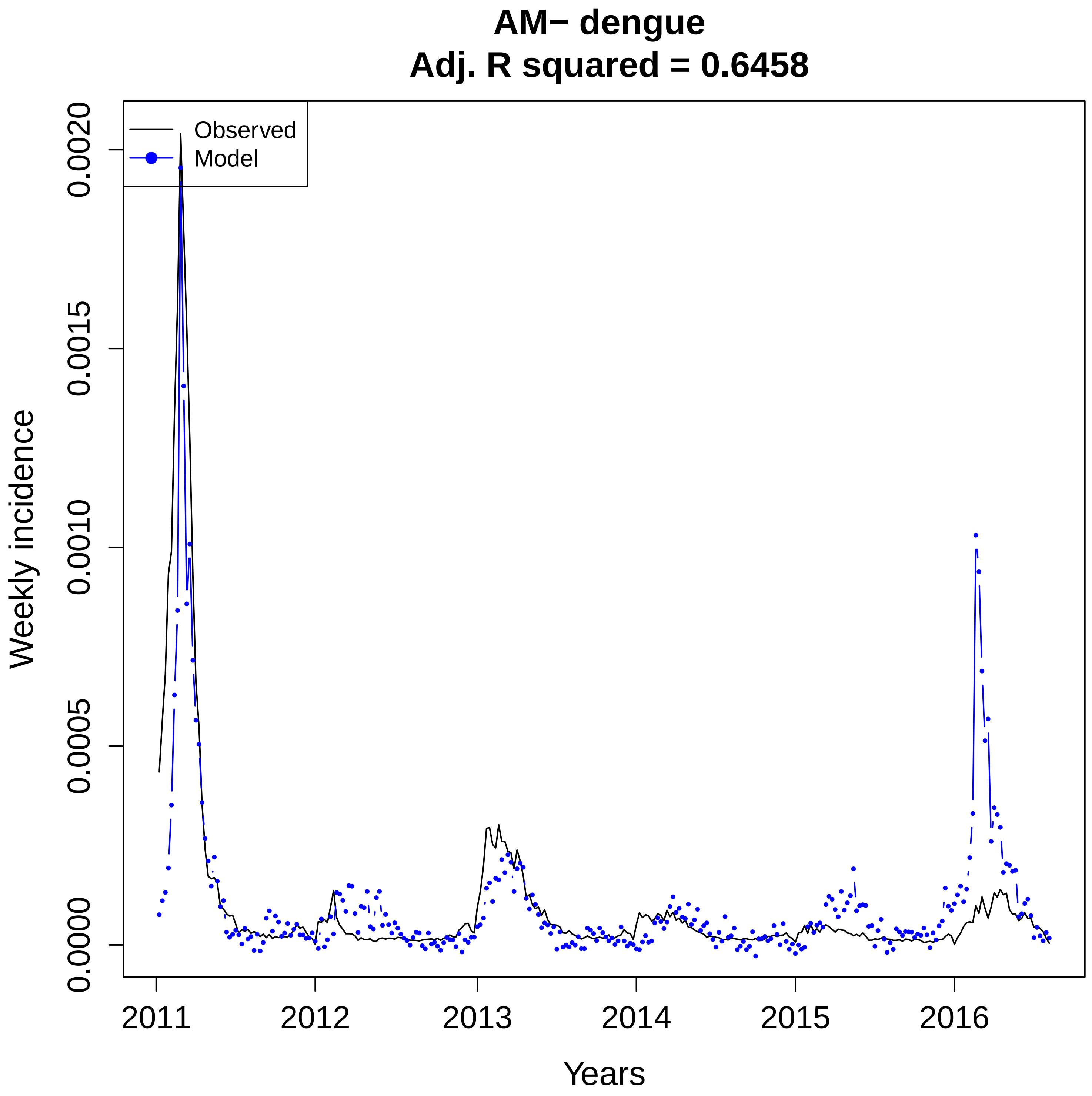

AM− dengue
Adj. R squared = 0.6458
Observed
Model
Weekly incidence
0.0000
0.0005
0.0010
0.0015
0.0020
2011
2012
2013
2014
2015
2016
Years

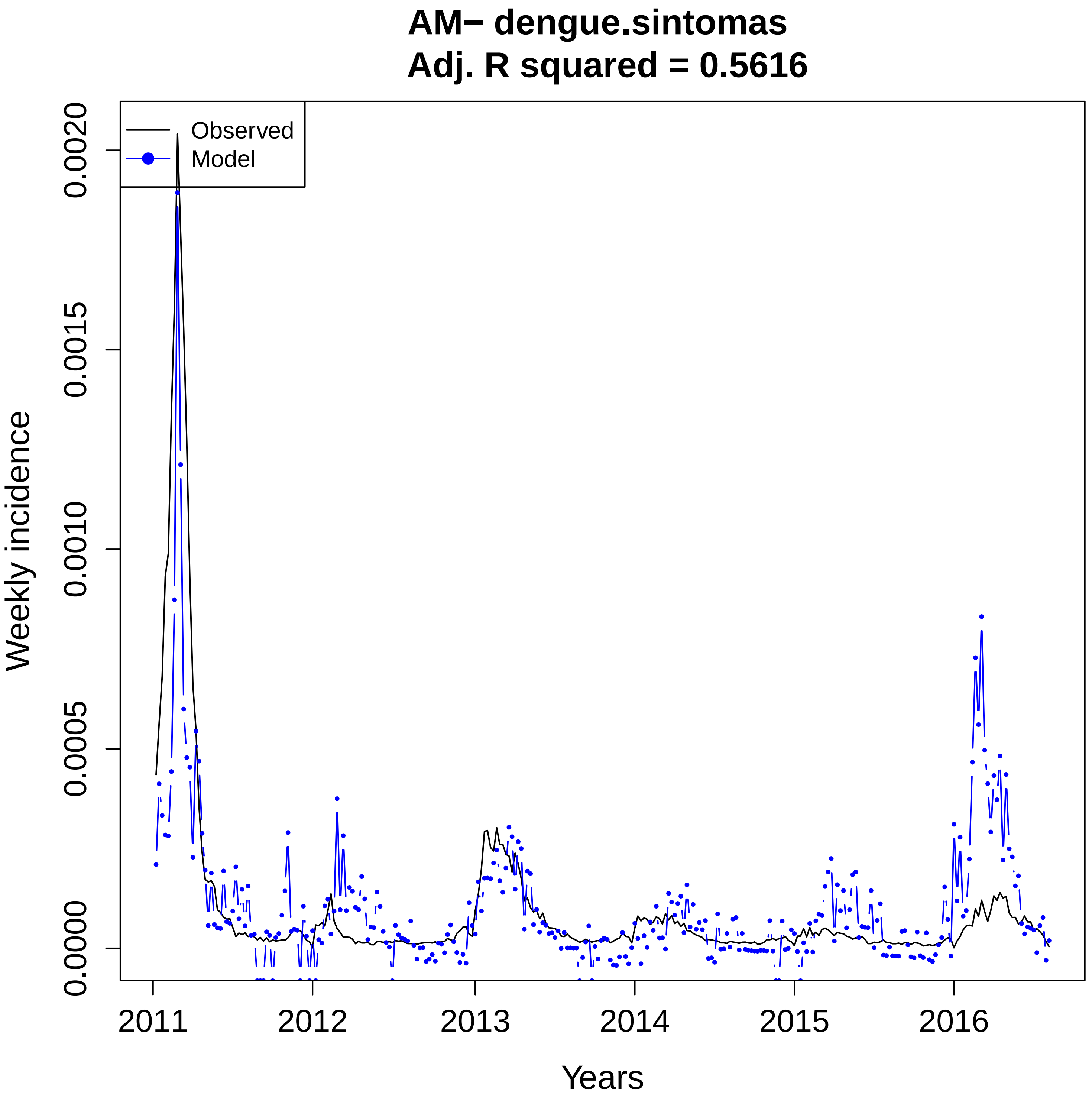

AM− dengue.sintomas
Adj. R squared = 0.5616
Observed
Model
Weekly incidence
0.0000
0.0005
0.0010
0.0015
0.0020
2011
2012
2013
2014
2015
2016
Years

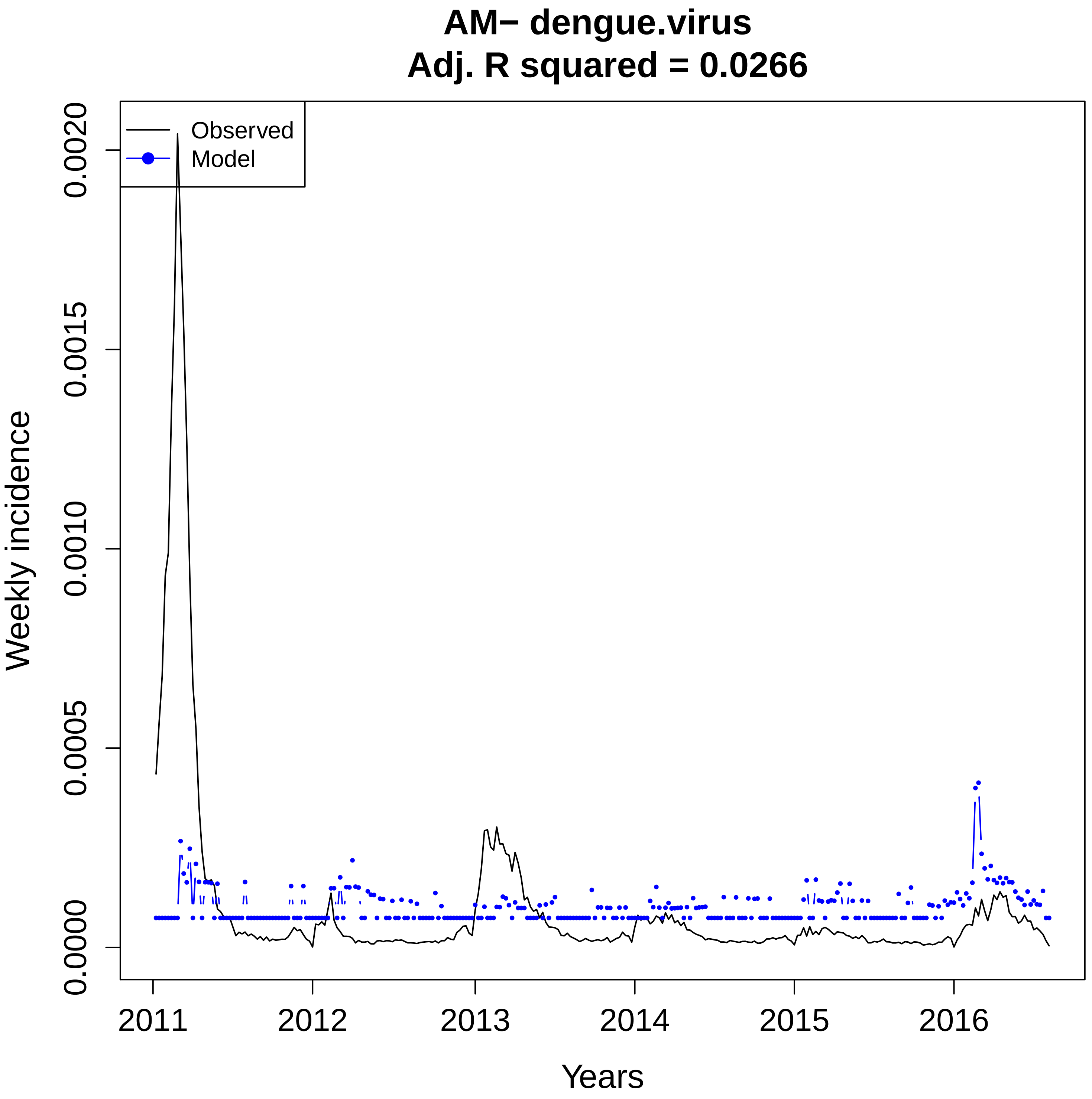
AM− dengue.virus
Adj. R squared = 0.0266
Observed
Model
Weekly incidence
0.0000
0.0005
0.0010
0.0015
0.0020
2011
2012
2013
2014
2015
2016
Years

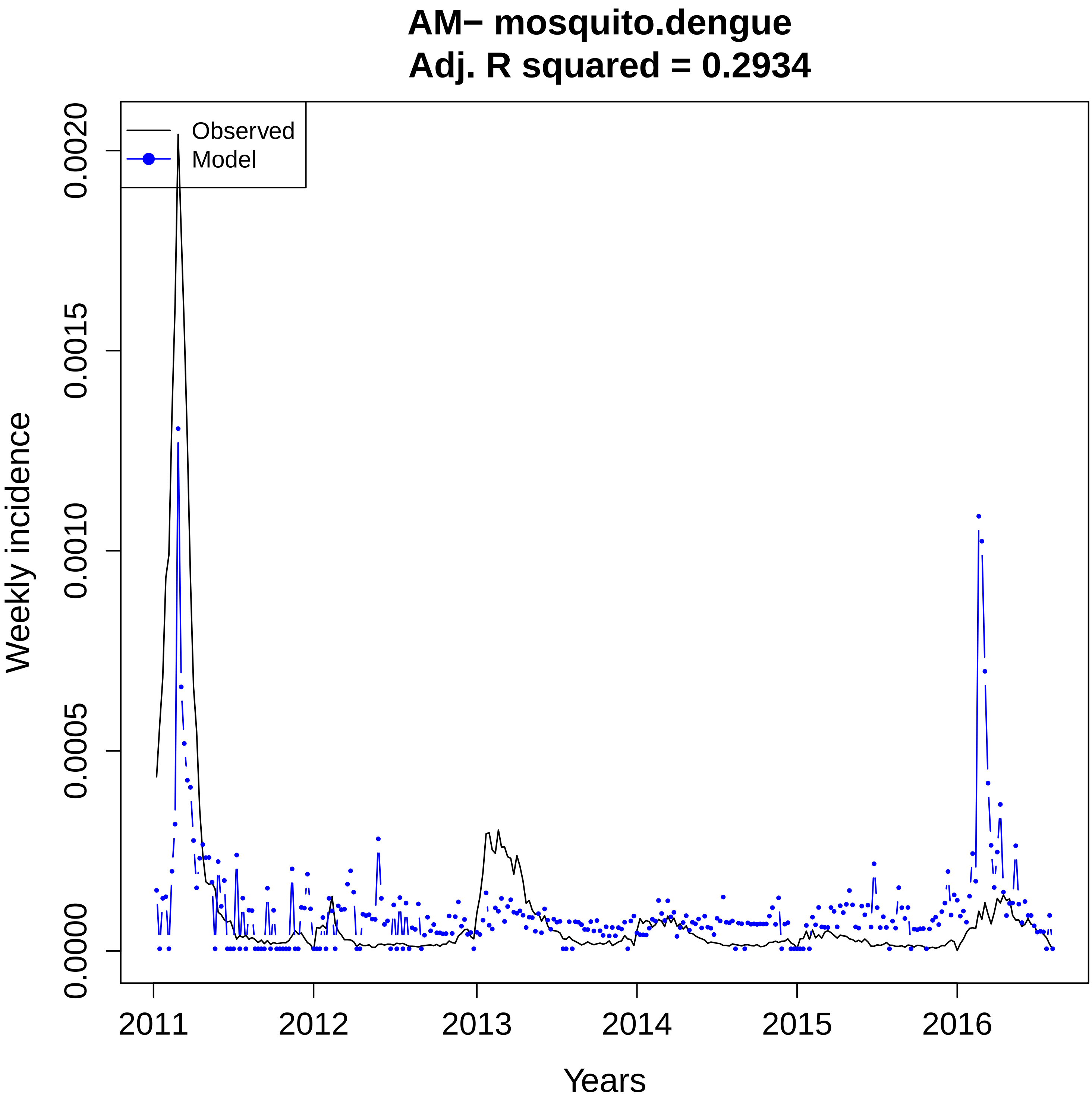

AM− mosquito.dengue
Adj. R squared = 0.2934
Observed
Model
Weekly incidence
0.0000
0.0005
0.0010
0.0015
0.0020
2011
2012
2013
2014
2015
2016
Years

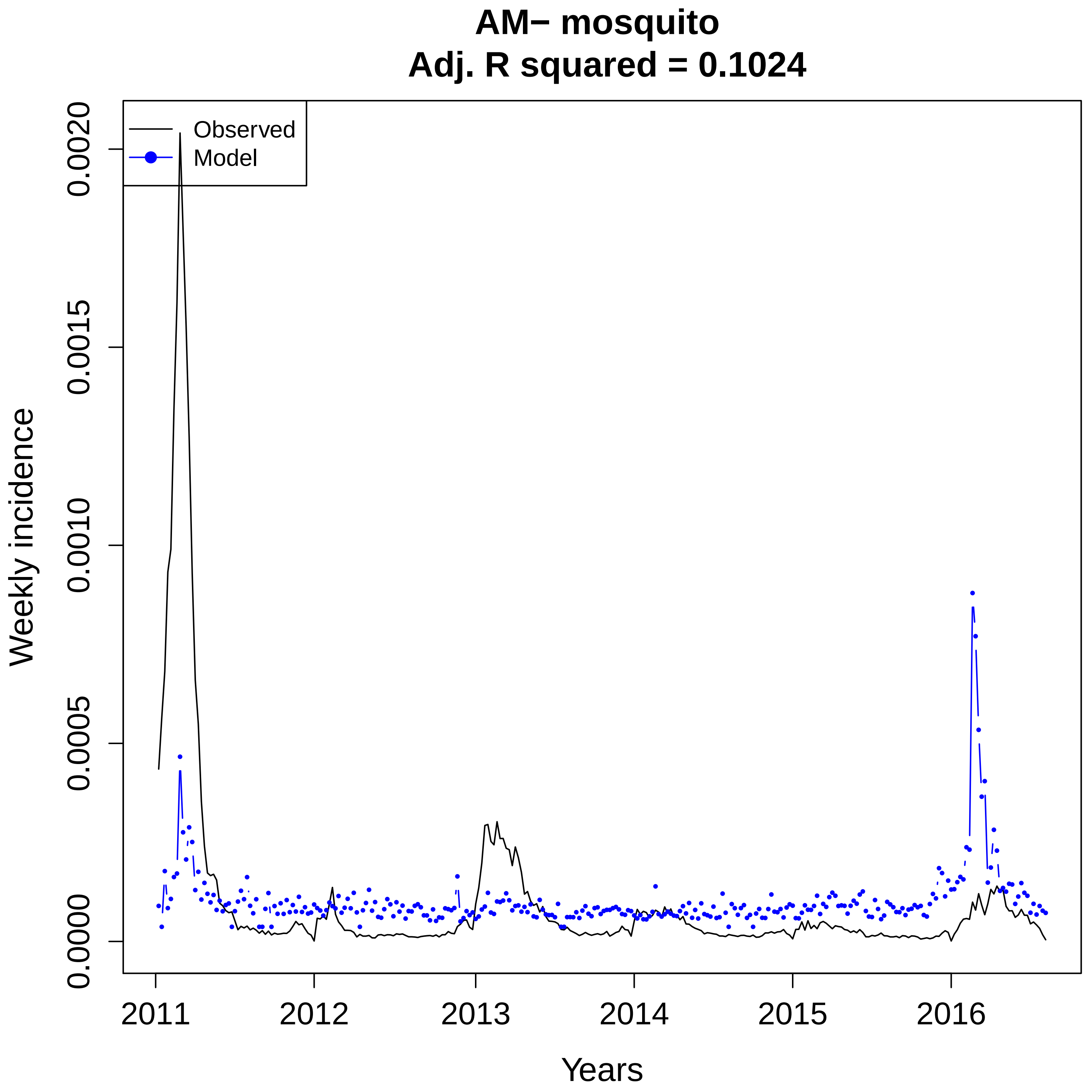
AM− mosquito
Adj. R squared = 0.1024
Observed
Model
Weekly incidence
0.0000
0.0005
0.0010
0.0015
0.0020
2011
2012
2013
2014
2015
2016
Years

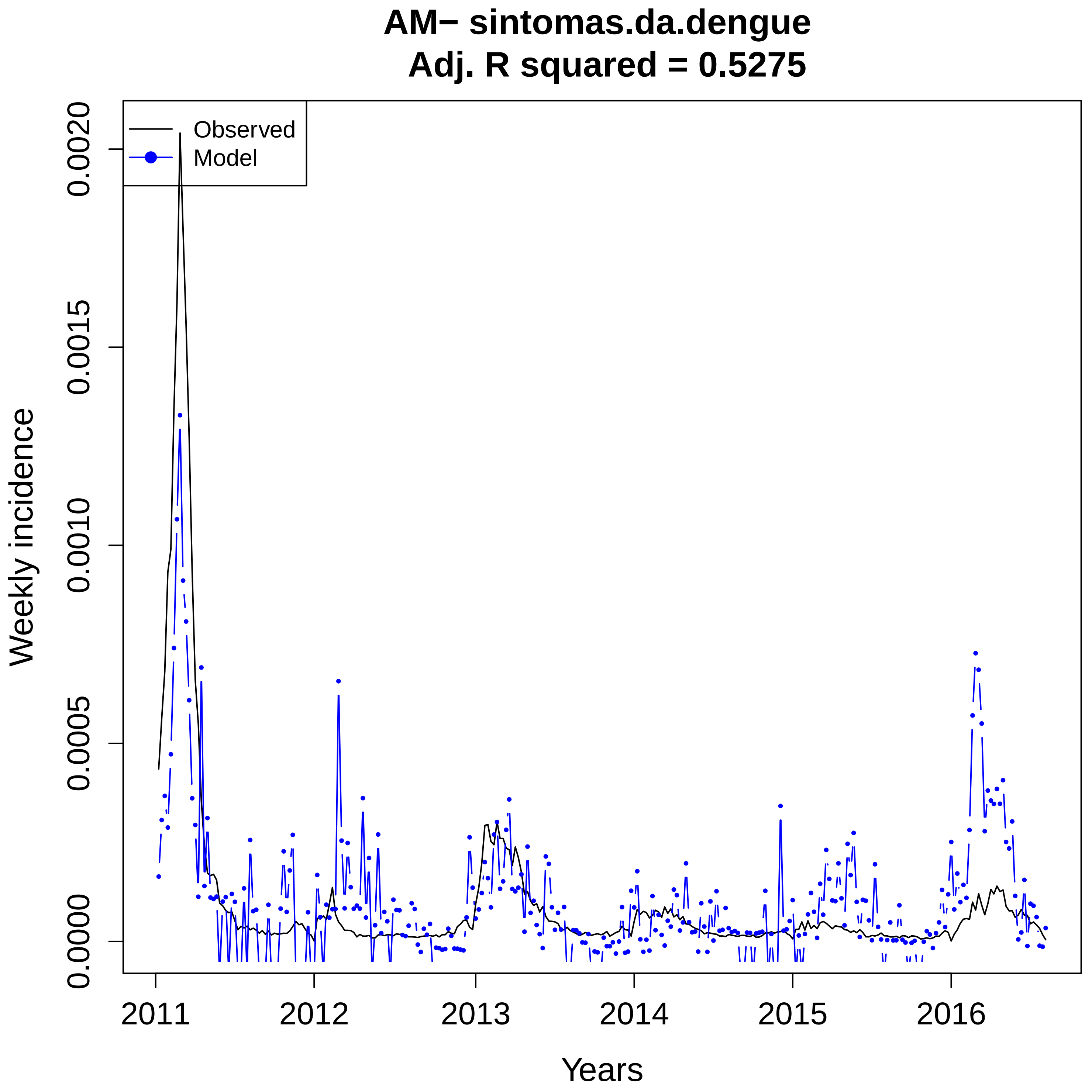
AM− sintomas.da.dengue
Adj. R squared = 0.5275
Observed
Model
Weekly incidence
0.0000
0.0005
0.0010
0.0015
0.0020
2011
2012
2013
2014
2015
2016
Years

Amapá (AP)

Dengue incidence vs. Google Health Trends (2011–2016)

Definitions:

- **Combined terms:**
  - **All:** model with all the available terms. n= number of terms
  - **Four:** model with "dengue", "dengue sintomas", "aedes", and "mosquito".
  - **Uncorrelated:** model with non-correlated terms. n = number of uncorrelated terms
- **Individual terms:** Plots developed with one term at a time.

- Terms for all and uncorrelated models can be found in additional file 3.

Combined terms
Adjusted R squared

Amapá (AP)

All
(n = 7)

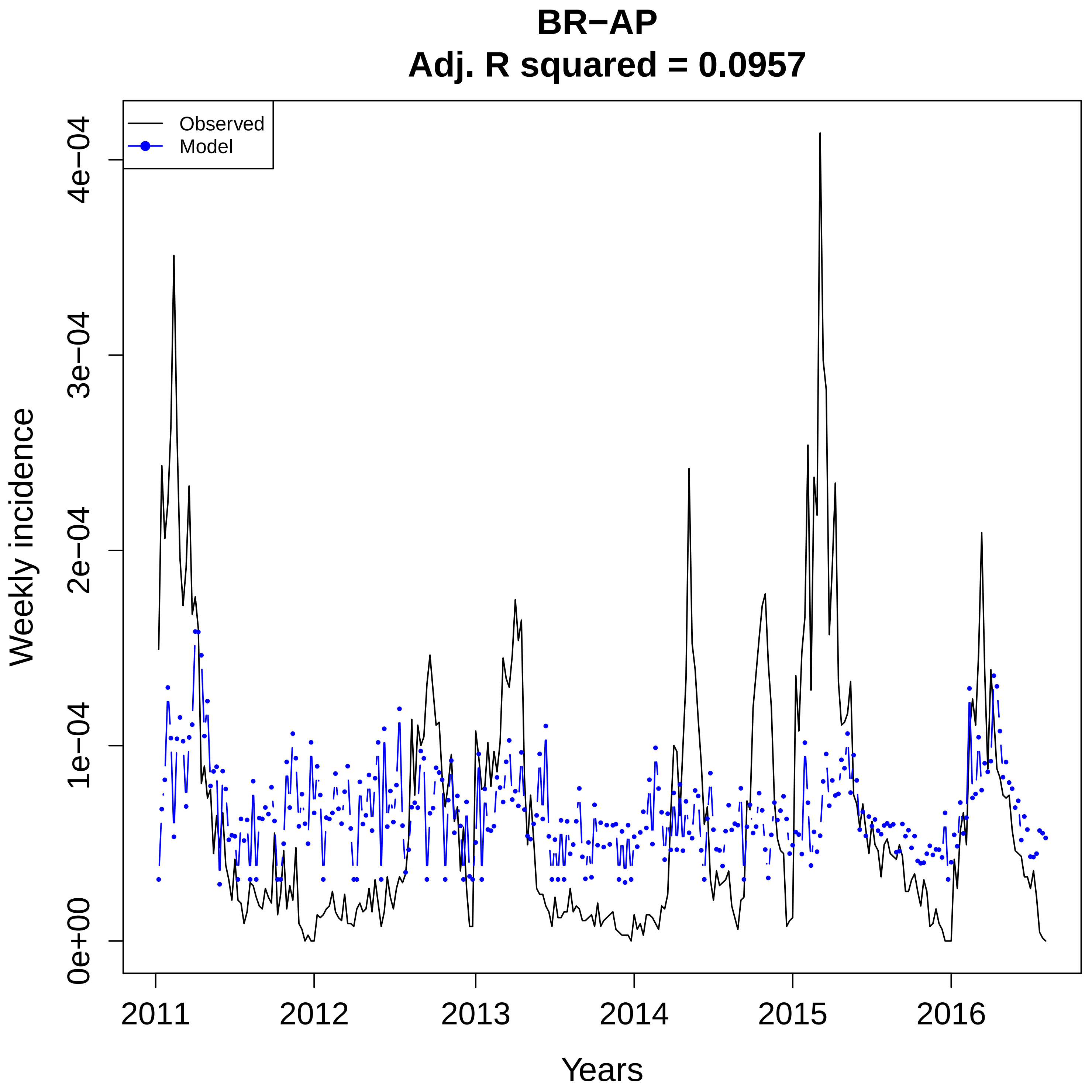
BR−AP
Adj. R squared = 0.0957
Observed
Model
Weekly incidence
0e+00
1e−04
2e−04
3e−04
4e−04
2011
2012
2013
2014
2015
2016
Years

Four

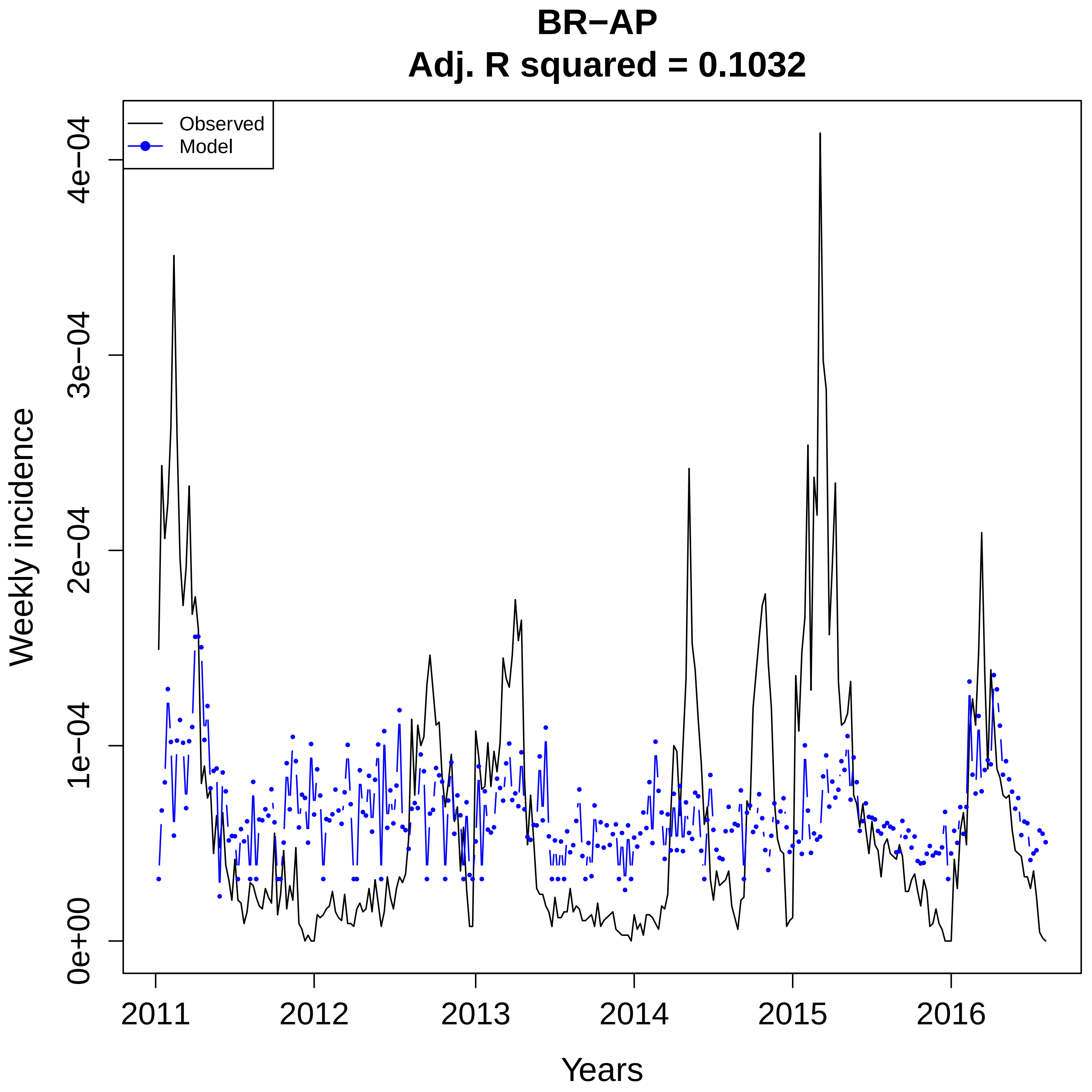

BR−AP
Adj. R squared = 0.1032
Observed
Model
Weekly incidence
0e+00
1e−04
2e−04
3e−04
4e−04
2011
2012
2013
2014
2015
2016
Years

Uncorrelated
(n = 6)

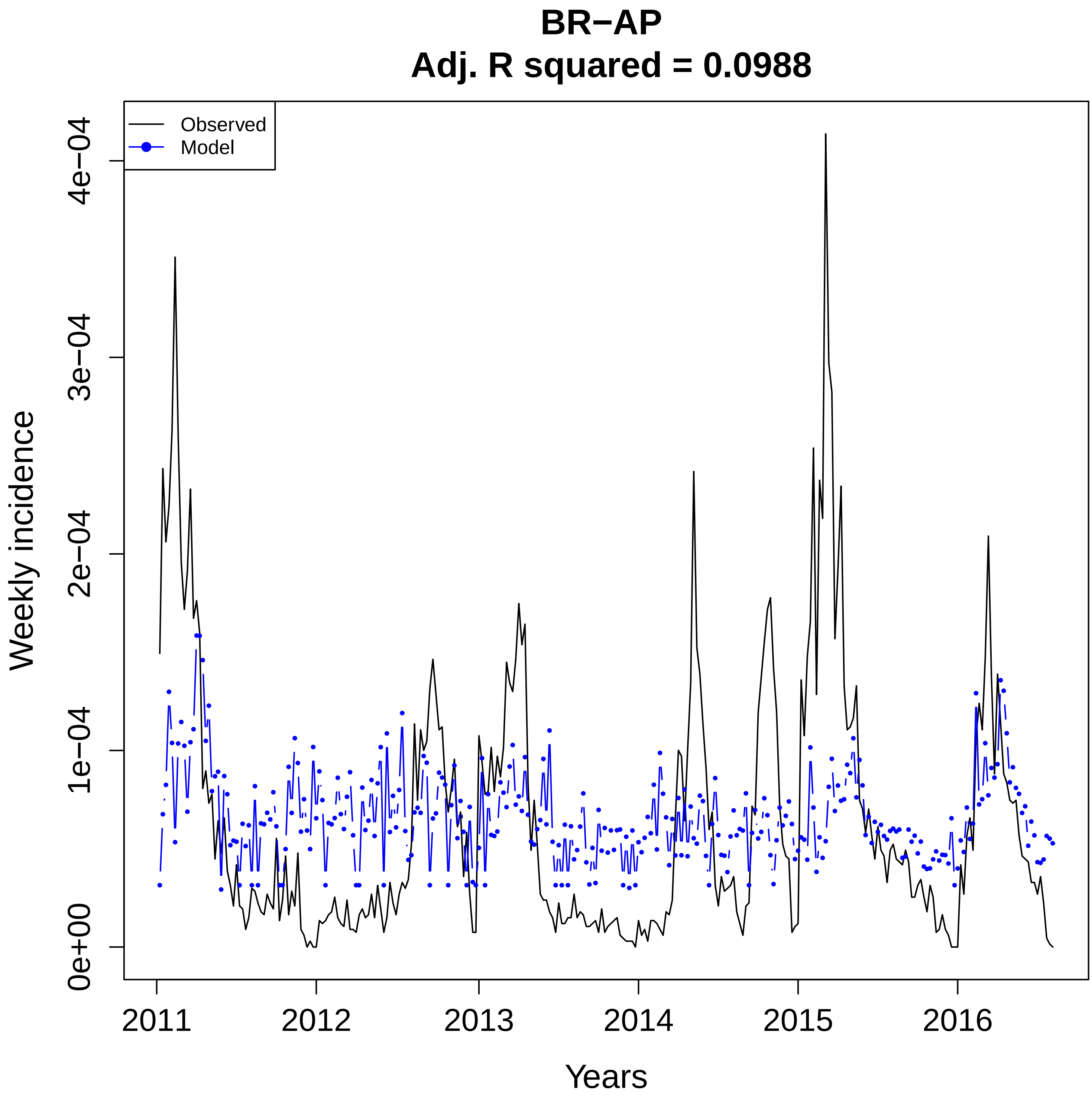

Individual terms
Adjusted R squared

Amapá (AP)

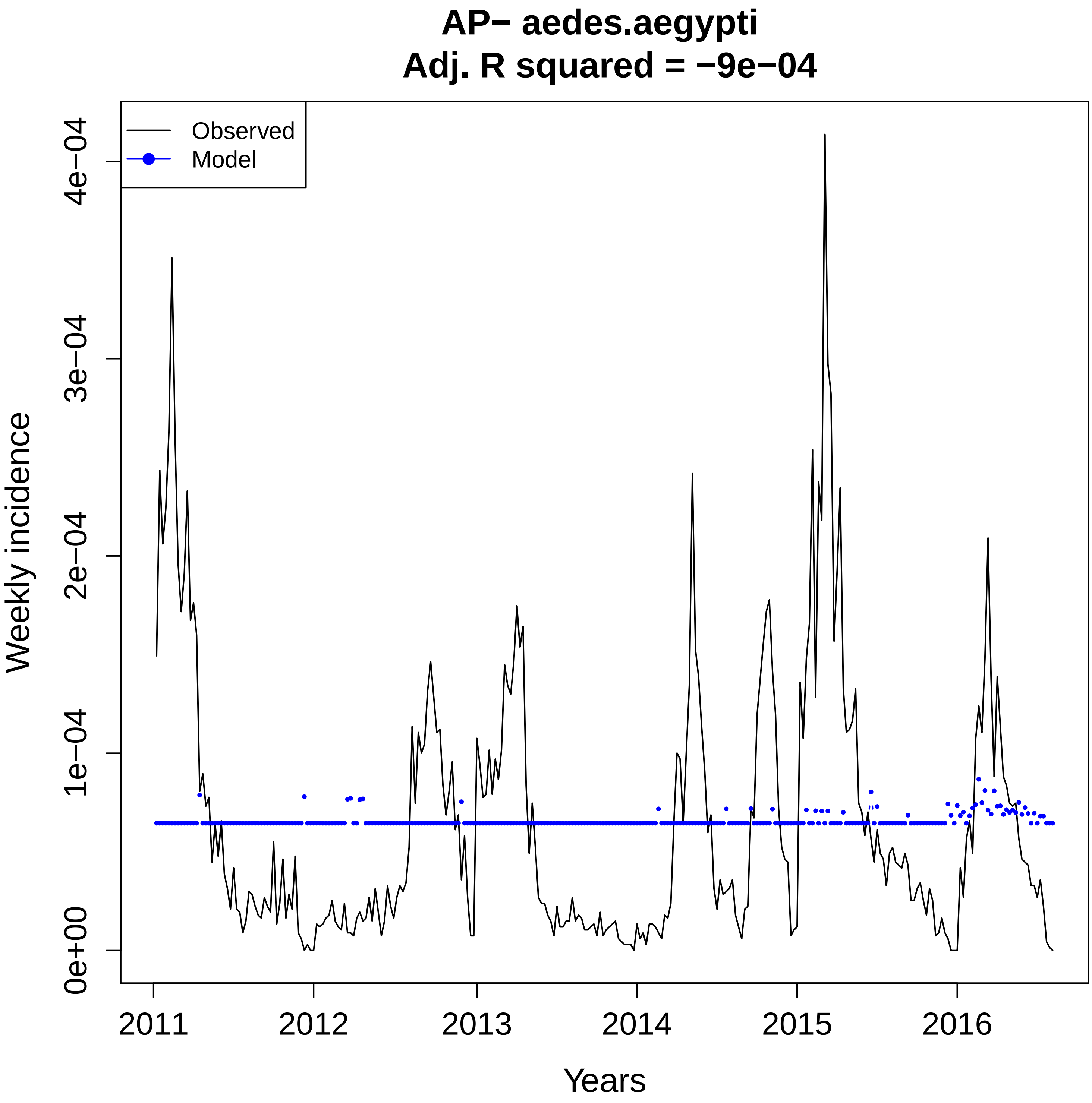

AP− aedes.aegypti
Adj. R squared = −9e−04
Observed
Model
Weekly incidence
0e+00
1e−04
2e−04
3e−04
4e−04
2011
2012
2013
2014
2015
2016
Years

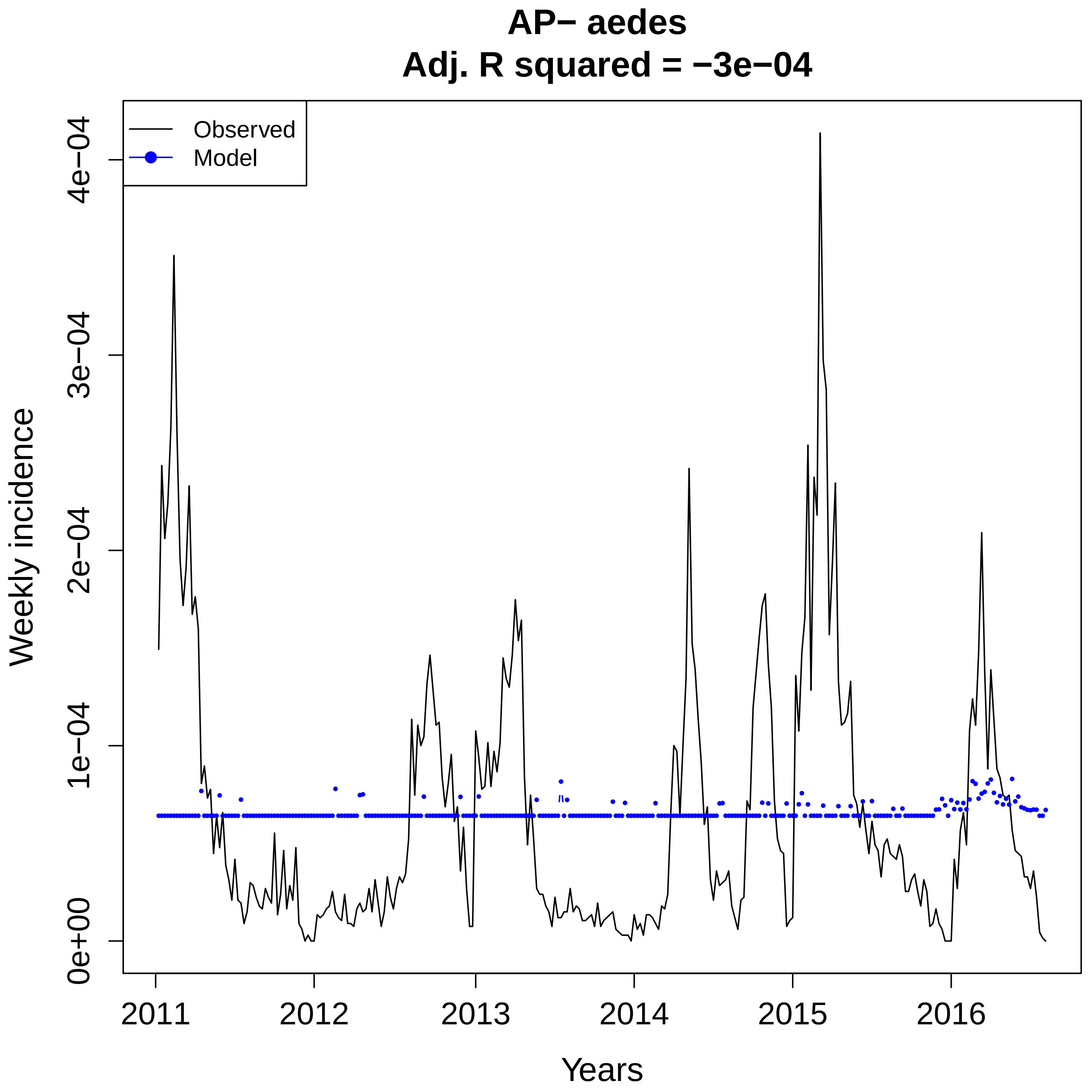

AP− aedes
Adj. R squared = −3e−04
Observed
Model
Weekly incidence
0e+00
1e−04
2e−04
3e−04
4e−04
2011
2012
2013
2014
2015
2016
Years

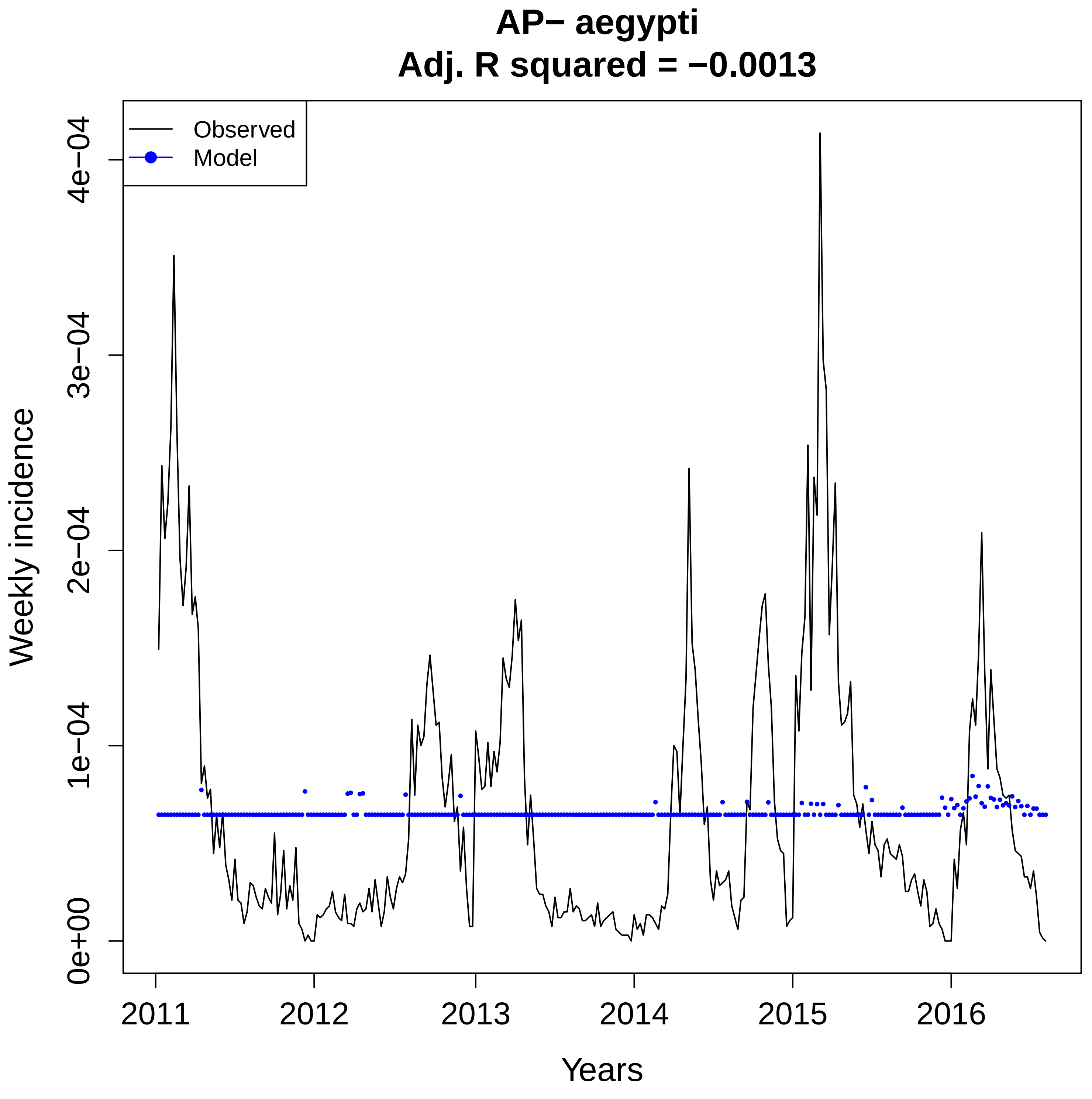
AP− aegypti
Adj. R squared = −0.0013
Observed
Model
Weekly incidence
0e+00
1e−04
2e−04
3e−04
4e−04
2011
2012
2013
2014
2015
2016
Years

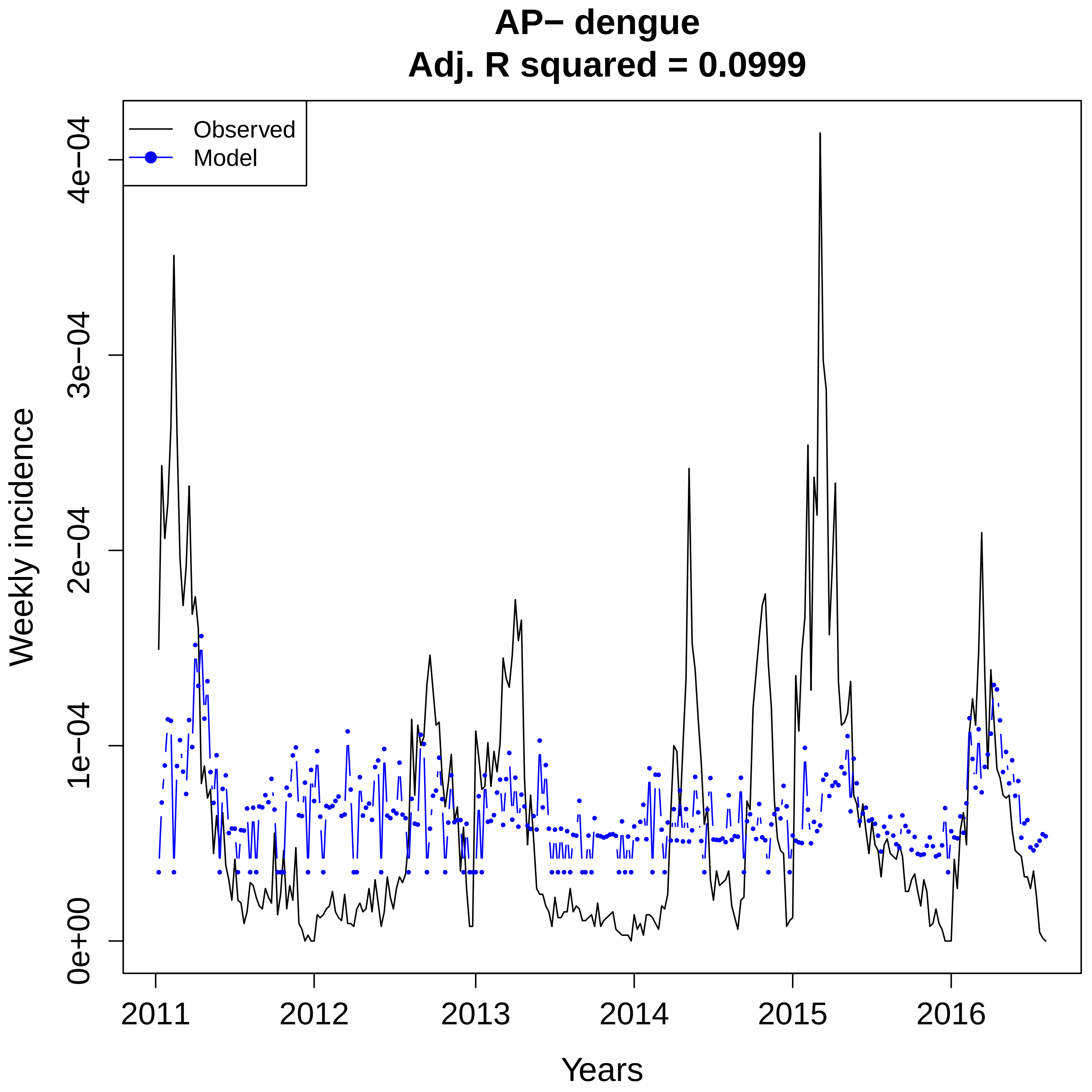
AP− dengue
Adj. R squared = 0.0999
Observed
Model
Weekly incidence
0e+00
1e−04
2e−04
3e−04
4e−04
2011
2012
2013
2014
2015
2016
Years

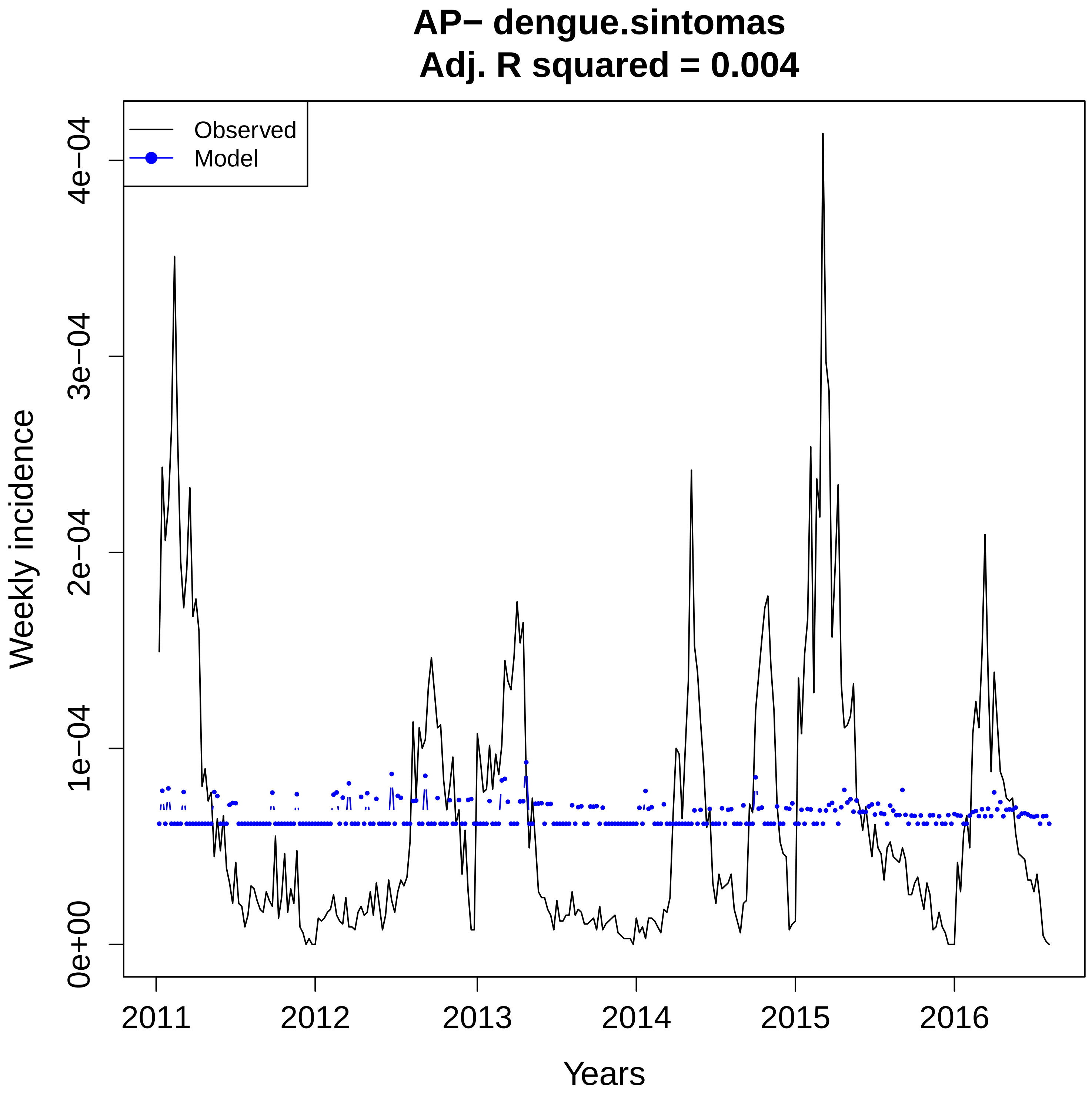
AP− dengue.sintomas
Adj. R squared = 0.004
Observed
Model
Weekly incidence
0e+00
1e−04
2e−04
3e−04
4e−04
2011
2012
2013
2014
2015
2016
Years

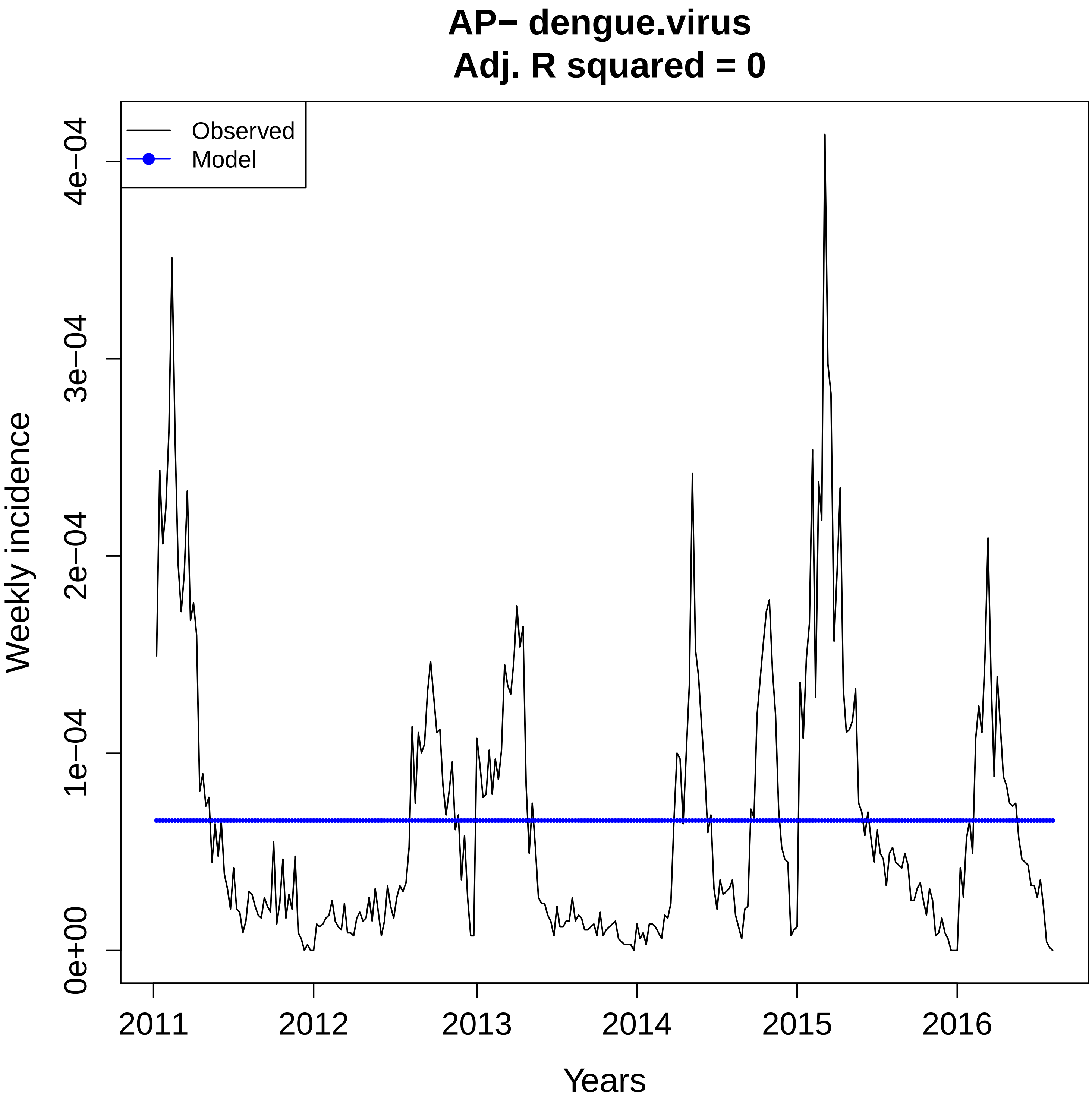

AP− dengue.virus
Adj. R squared = 0
Observed
Model
Weekly incidence
0e+00
1e−04
2e−04
3e−04
4e−04
2011
2012
2013
2014
2015
2016
Years

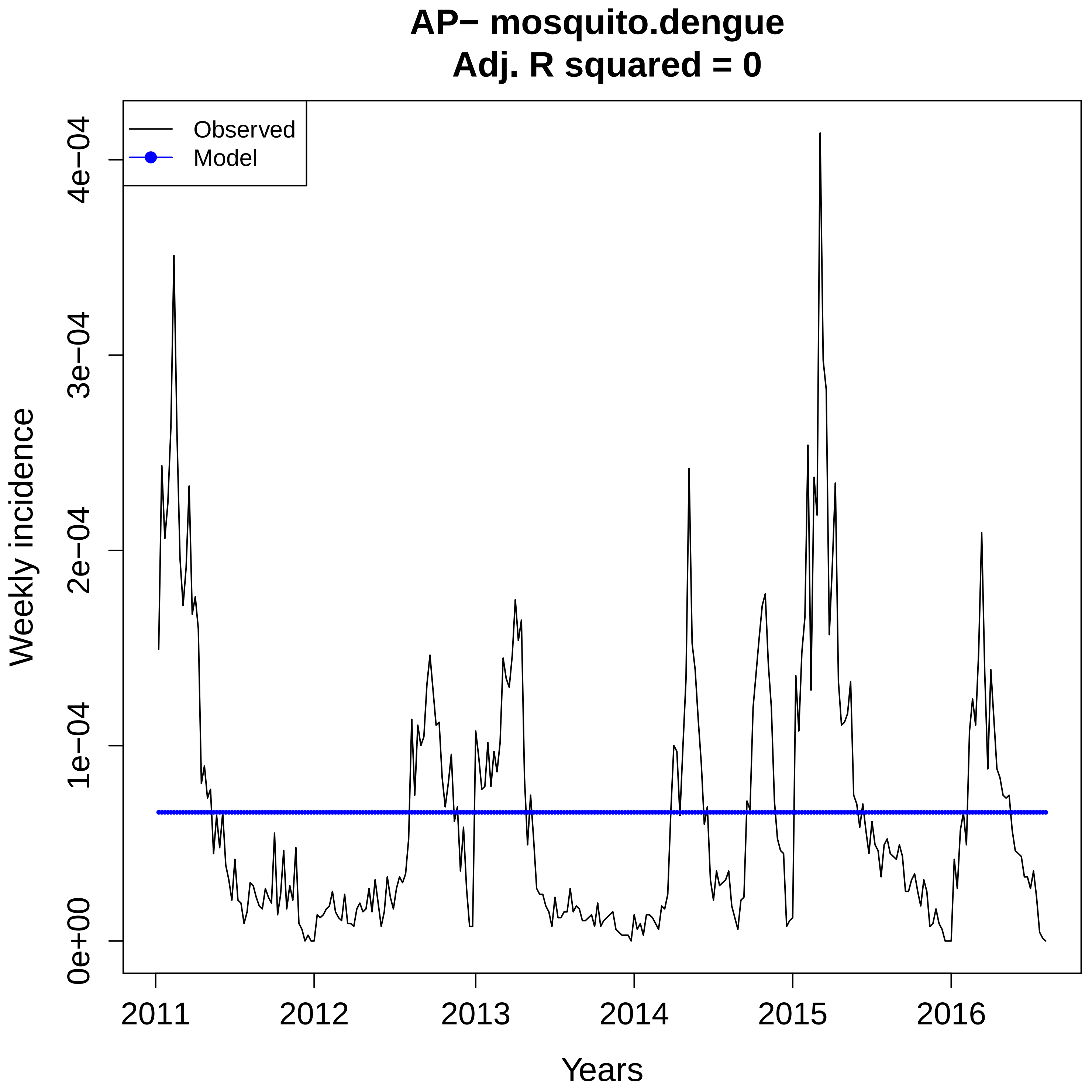
AP− mosquito.dengue
Adj. R squared = 0
Observed
Model
Weekly incidence
0e+00
1e−04
2e−04
3e−04
4e−04
2011
2012
2013
2014
2015
2016
Years

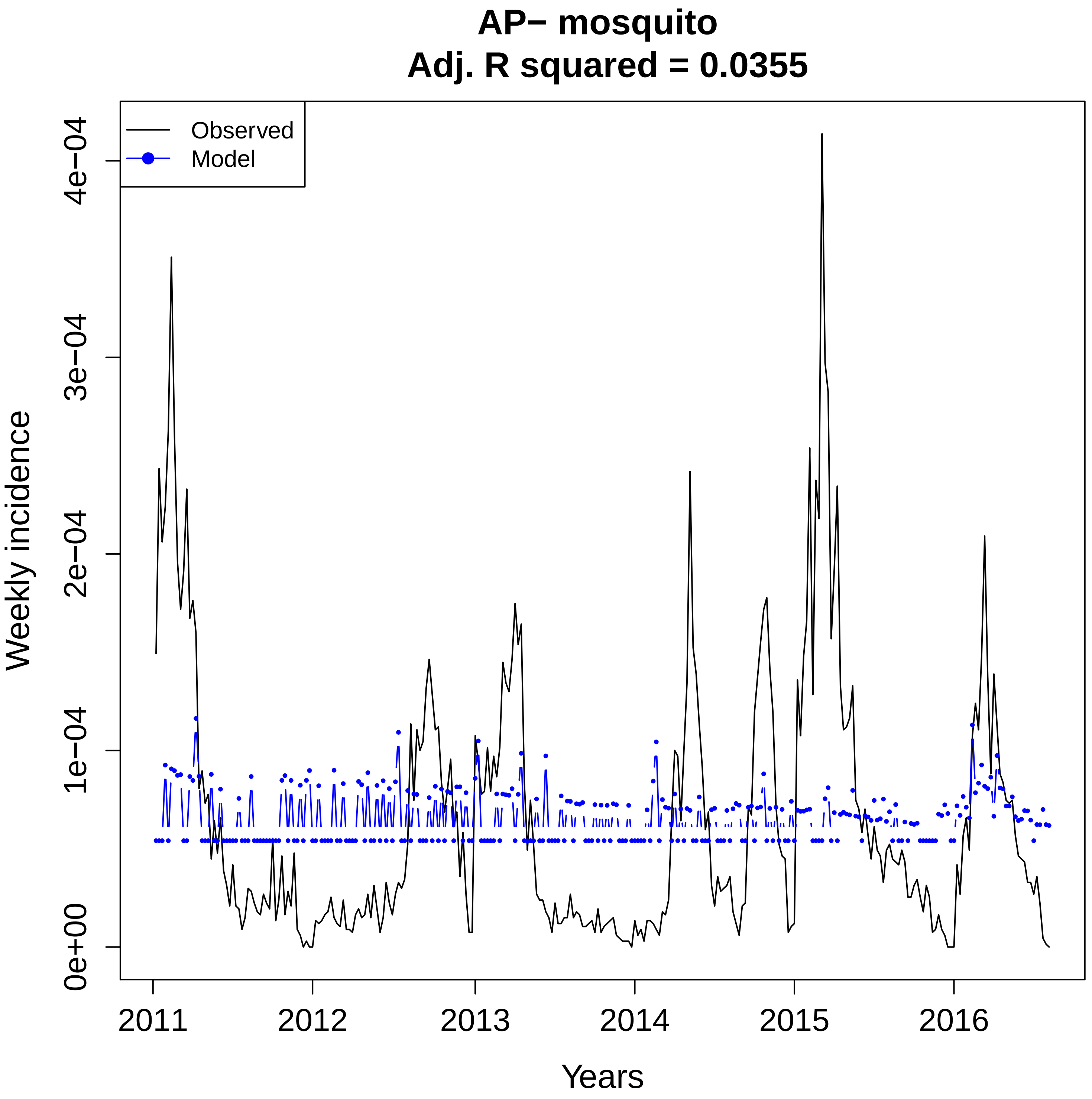

AP− mosquito
Adj. R squared = 0.0355
Observed
Model
Weekly incidence
0e+00
1e−04
2e−04
3e−04
4e−04
2011
2012
2013
2014
2015
2016
Years

Bahia (BA)
Dengue incidence vs. Google Health Trends (2011–2016)

Definitions:

- **Combined terms:**
  - **All:** model with all the available terms. n= number of terms
  - **Four:** model with "dengue", "dengue sintomas", "aedes", and "mosquito".
  - **Uncorrelated:** model with non-correlated terms. n = number of uncorrelated terms
- **Individual terms:** Plots developed with one term at a time.

- Terms for all and uncorrelated models can be found in additional file 3.

Combined terms
Adjusted R squared

Bahia (BA)

All
(n = 11)

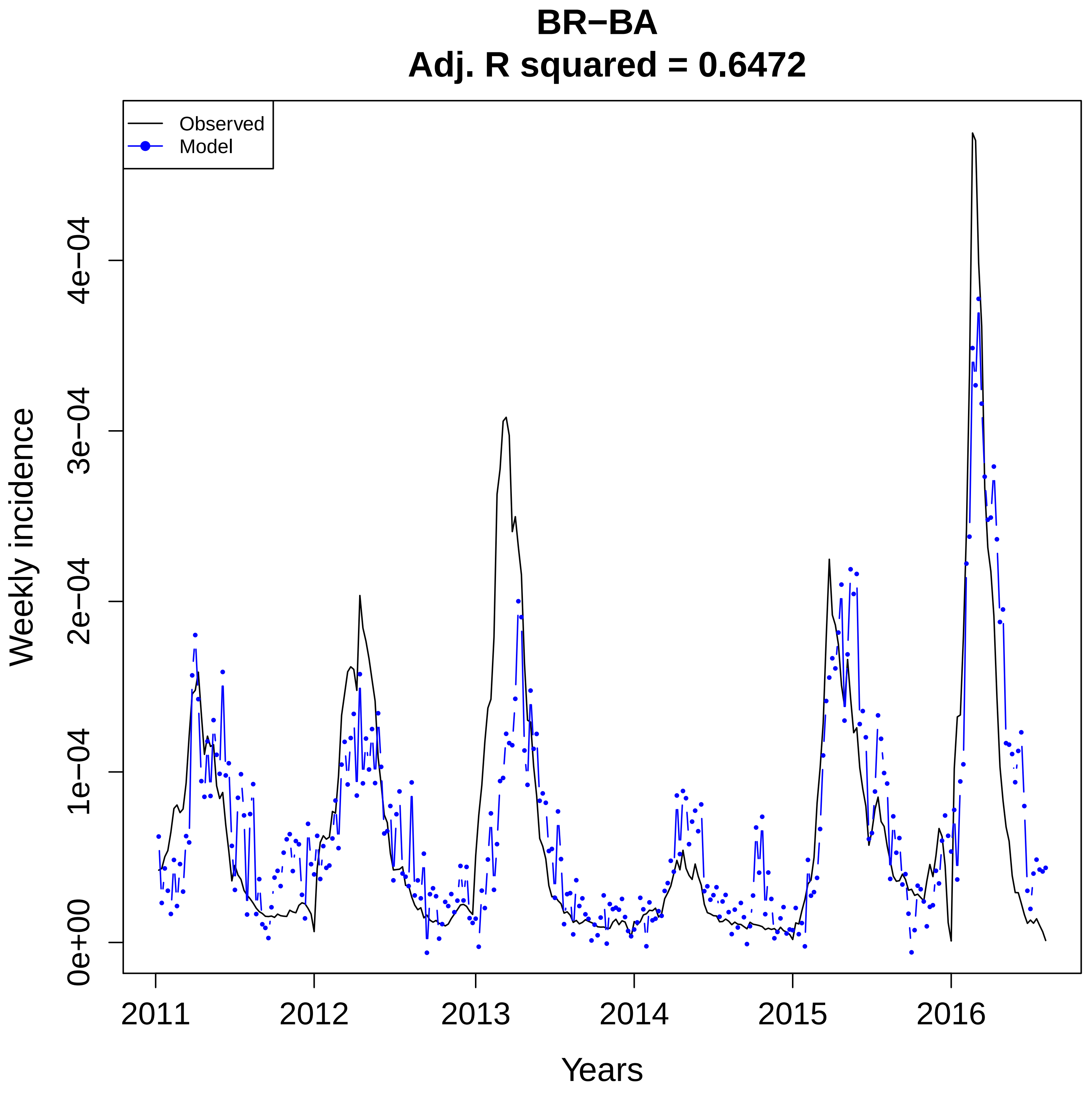
BR−BA
Adj. R squared = 0.6472
Observed
Model
Weekly incidence
0e+00
1e−04
2e−04
3e−04
4e−04
2011
2012
2013
2014
2015
2016
Years

Four

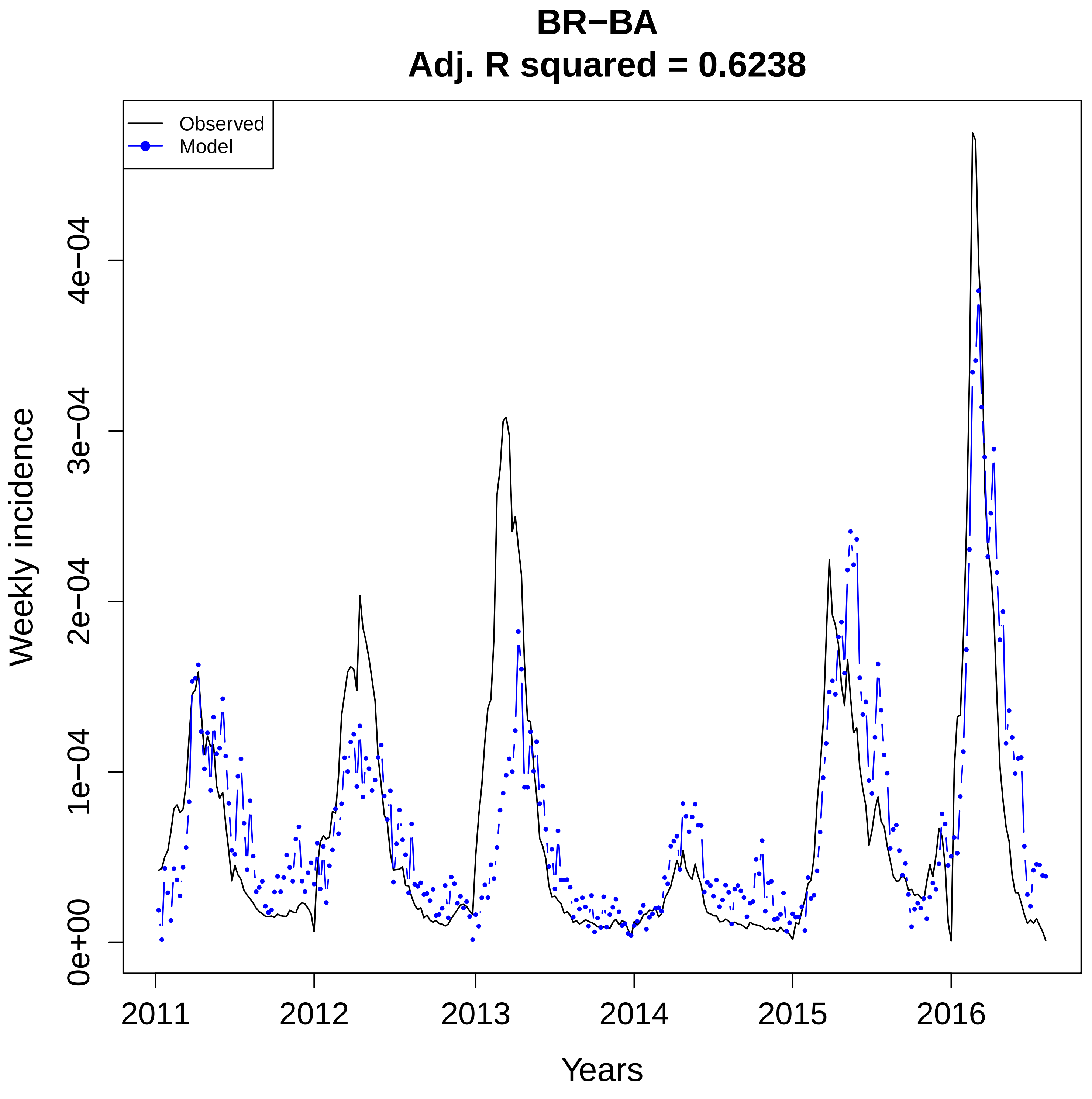

BR−BA
Adj. R squared = 0.6238
Observed
Model
Weekly incidence
0e+00
1e−04
2e−04
3e−04
4e−04
2011
2012
2013
2014
2015
2016
Years

Uncorrelated
(n = 3)

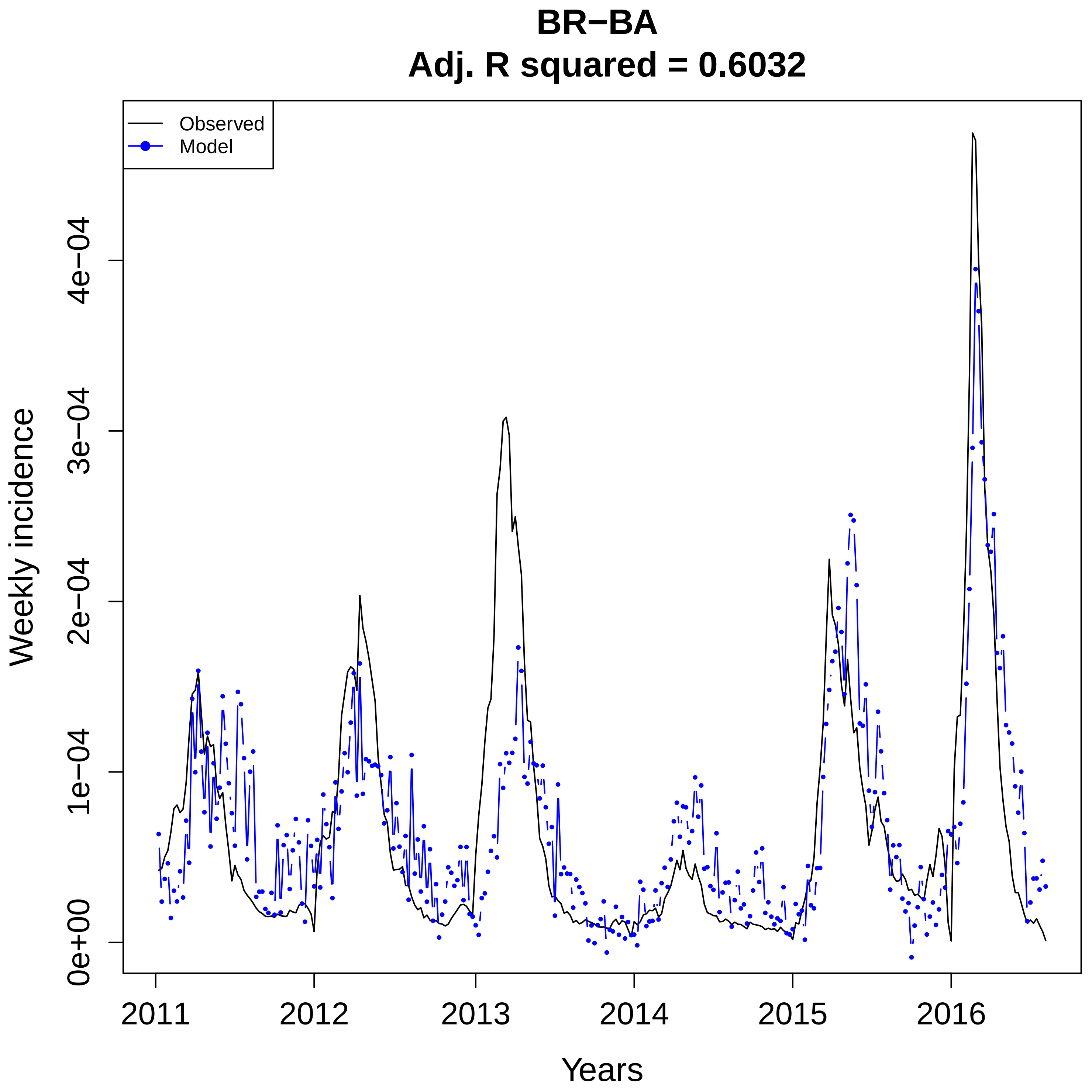

Individual terms
Adjusted R squared

Bahia (BA)

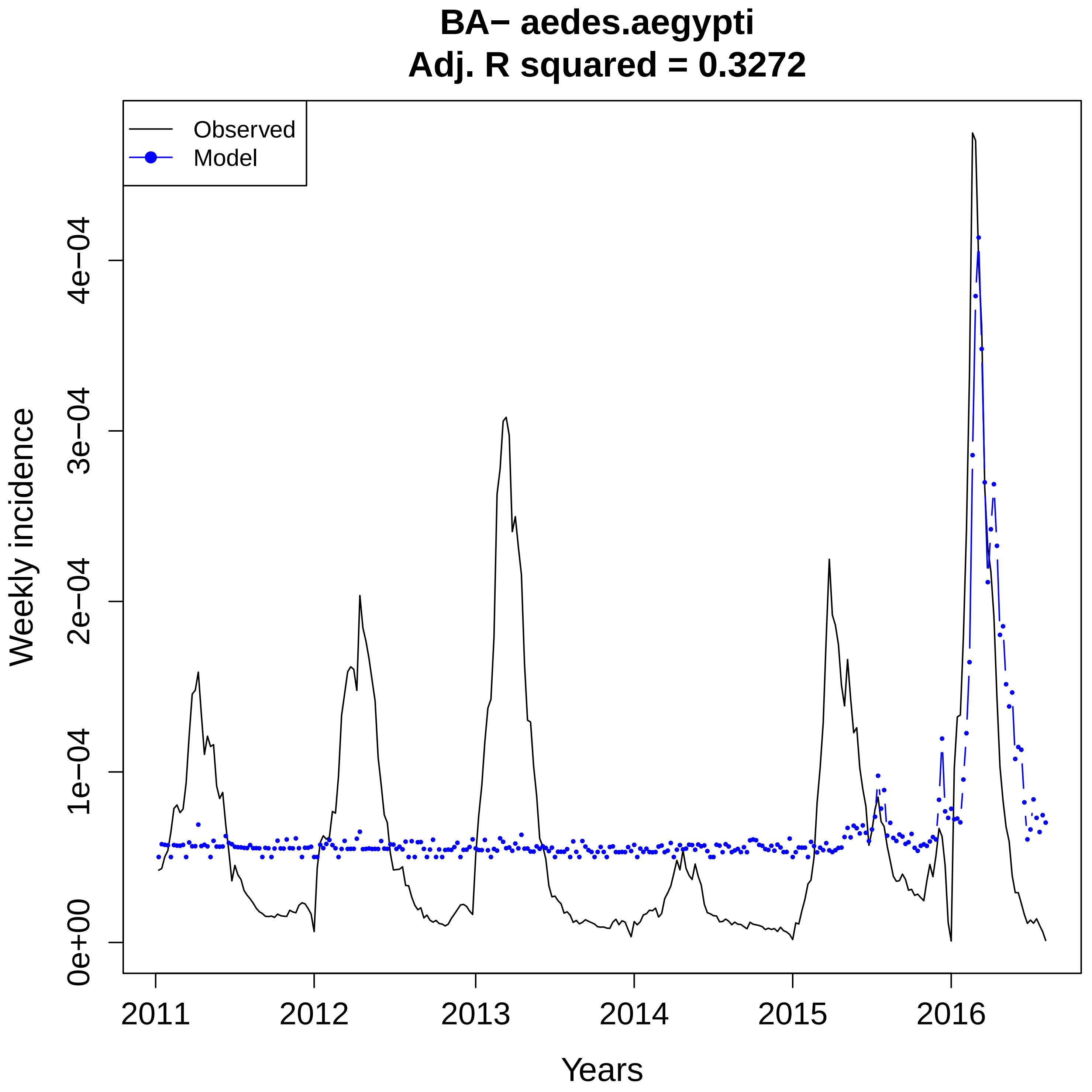
BA− aedes.aegypti
Adj. R squared = 0.3272
Observed
Model
Weekly incidence
0e+00
1e−04
2e−04
3e−04
4e−04
2011
2012
2013
2014
2015
2016
Years

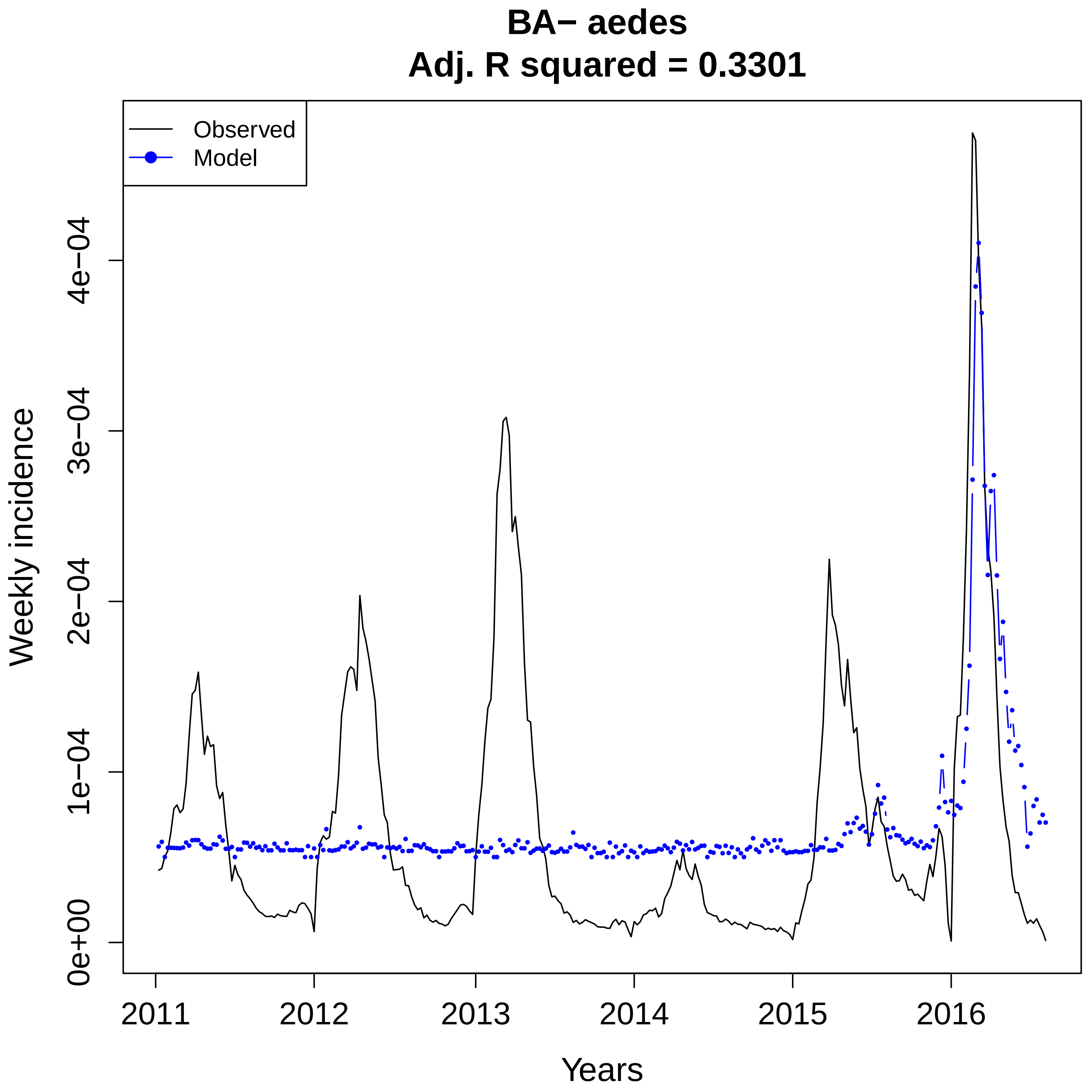

BA− aedes
Adj. R squared = 0.3301
Observed
Model
Weekly incidence
0e+00
1e−04
2e−04
3e−04
4e−04
2011
2012
2013
2014
2015
2016
Years

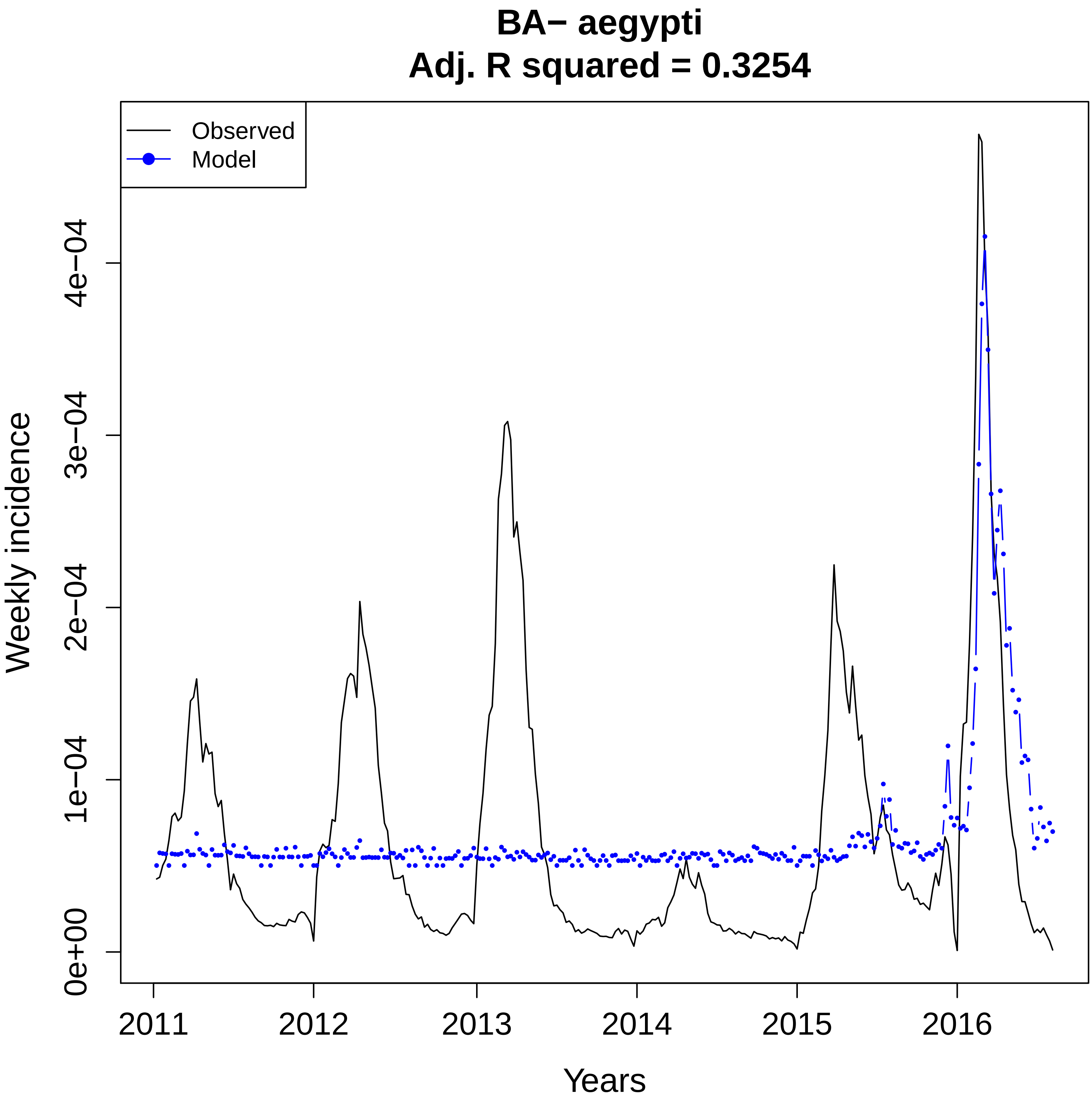
BA− aegypti
Adj. R squared = 0.3254
Observed
Model
Weekly incidence
0e+00
1e−04
2e−04
3e−04
4e−04
2011
2012
2013
2014
2015
2016
Years

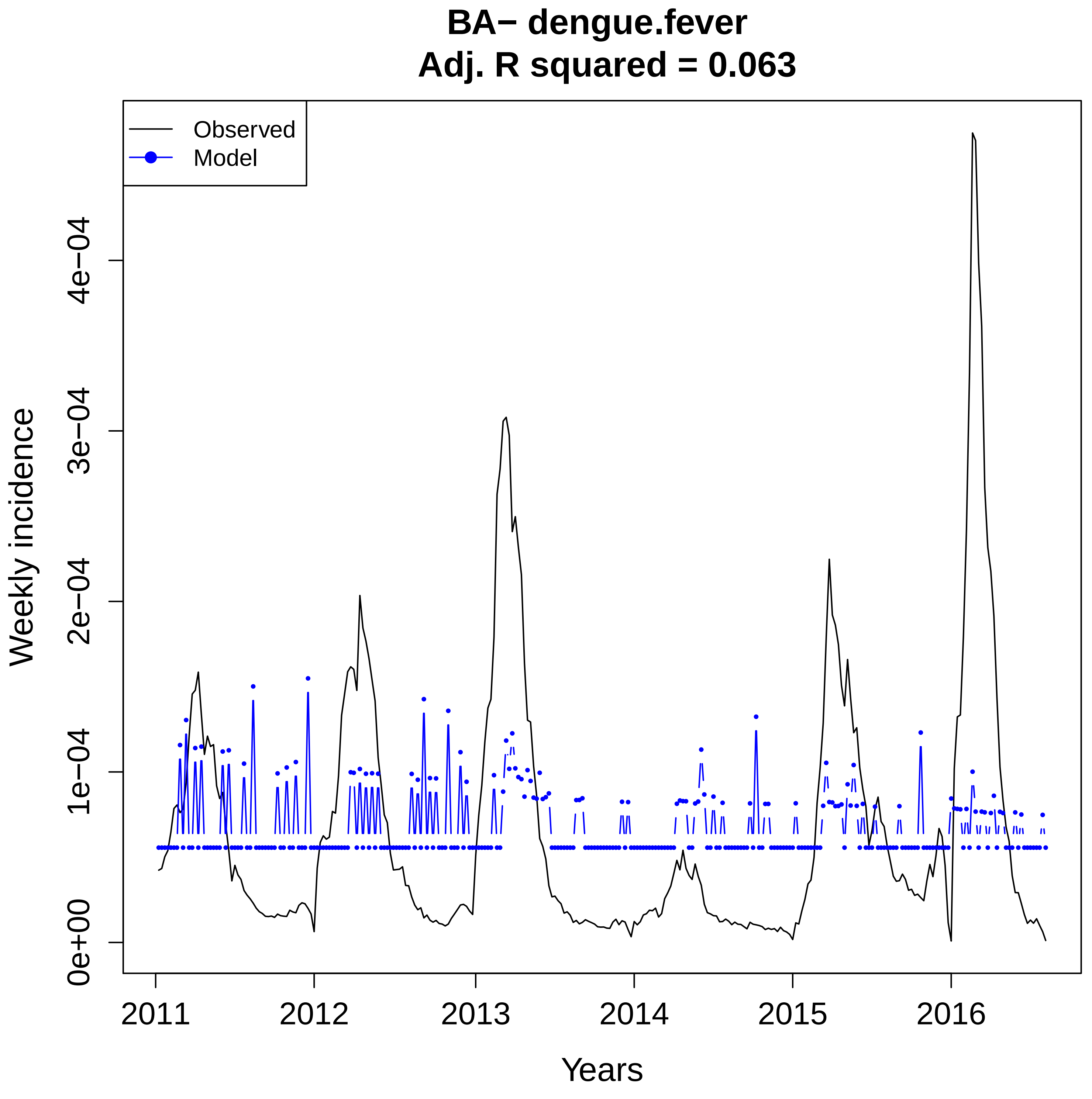

BA− dengue.fever
Adj. R squared = 0.063
Observed
Model
Weekly incidence
0e+00
1e−04
2e−04
3e−04
4e−04
2011
2012
2013
2014
2015
2016
Years

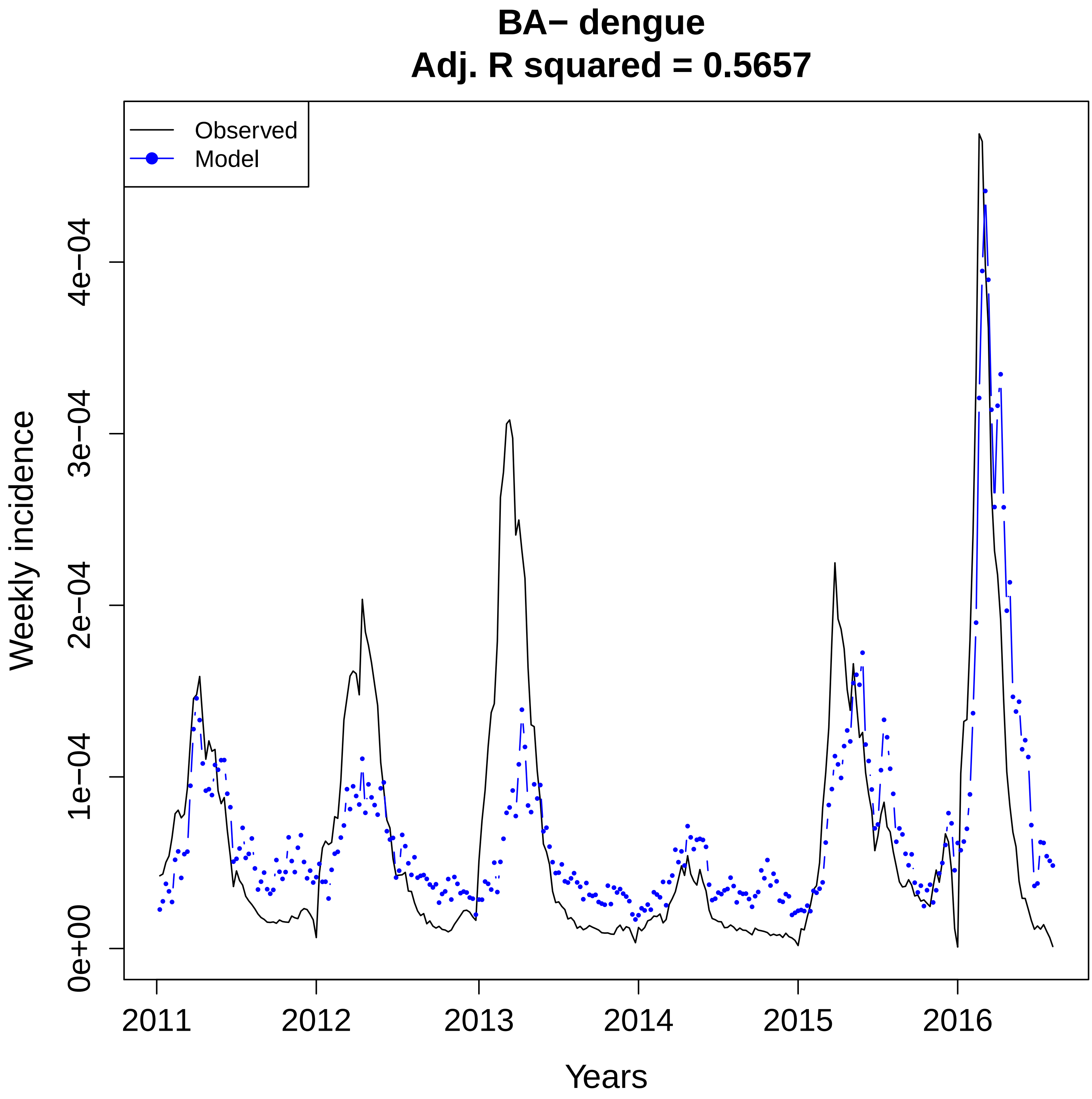

BA− dengue
Adj. R squared = 0.5657
Observed
Model
Weekly incidence
0e+00
1e−04
2e−04
3e−04
4e−04
2011
2012
2013
2014
2015
2016
Years

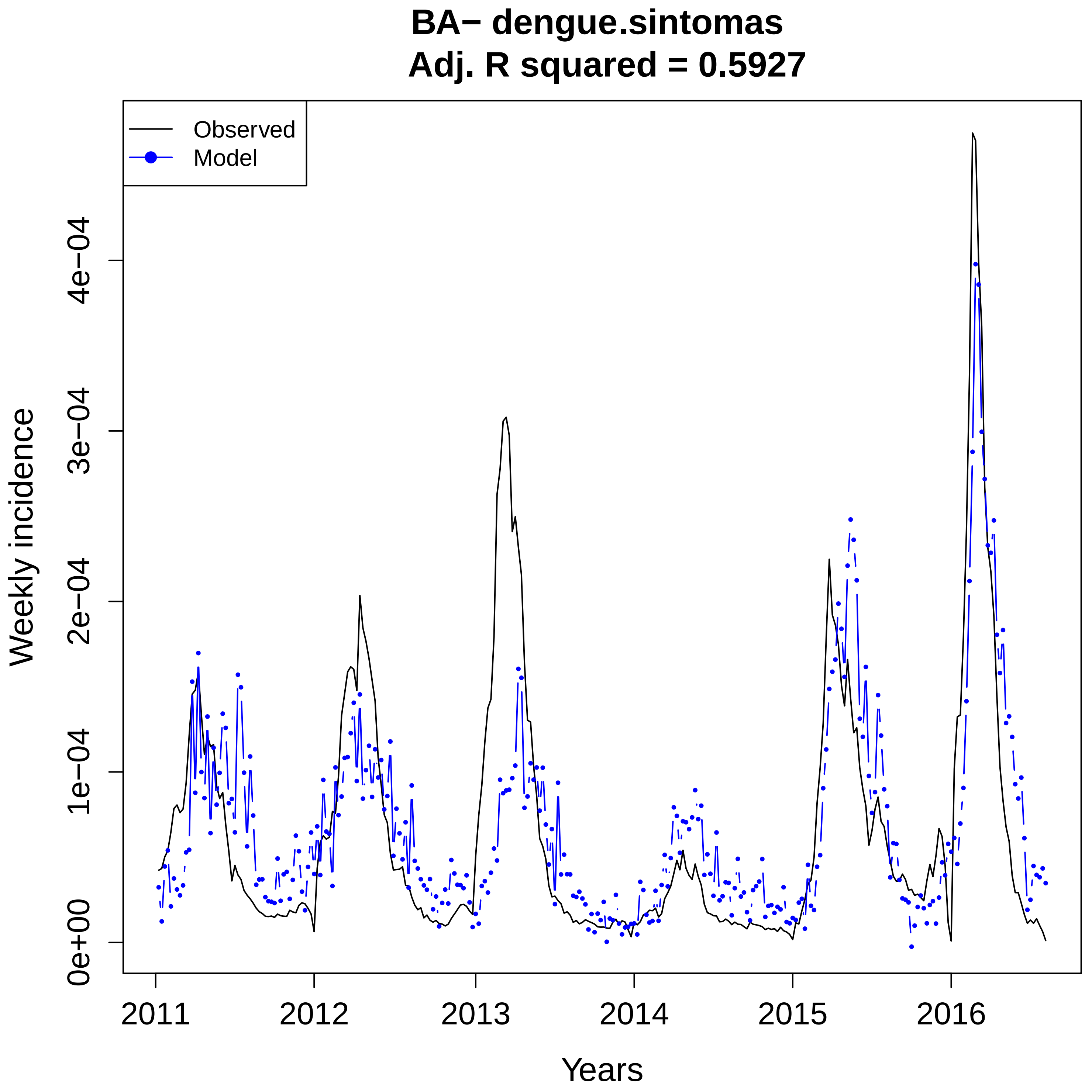

BA− dengue.sintomas
Adj. R squared = 0.5927
Observed
Model
Weekly incidence
0e+00
1e−04
2e−04
3e−04
4e−04
2011
2012
2013
2014
2015
2016
Years

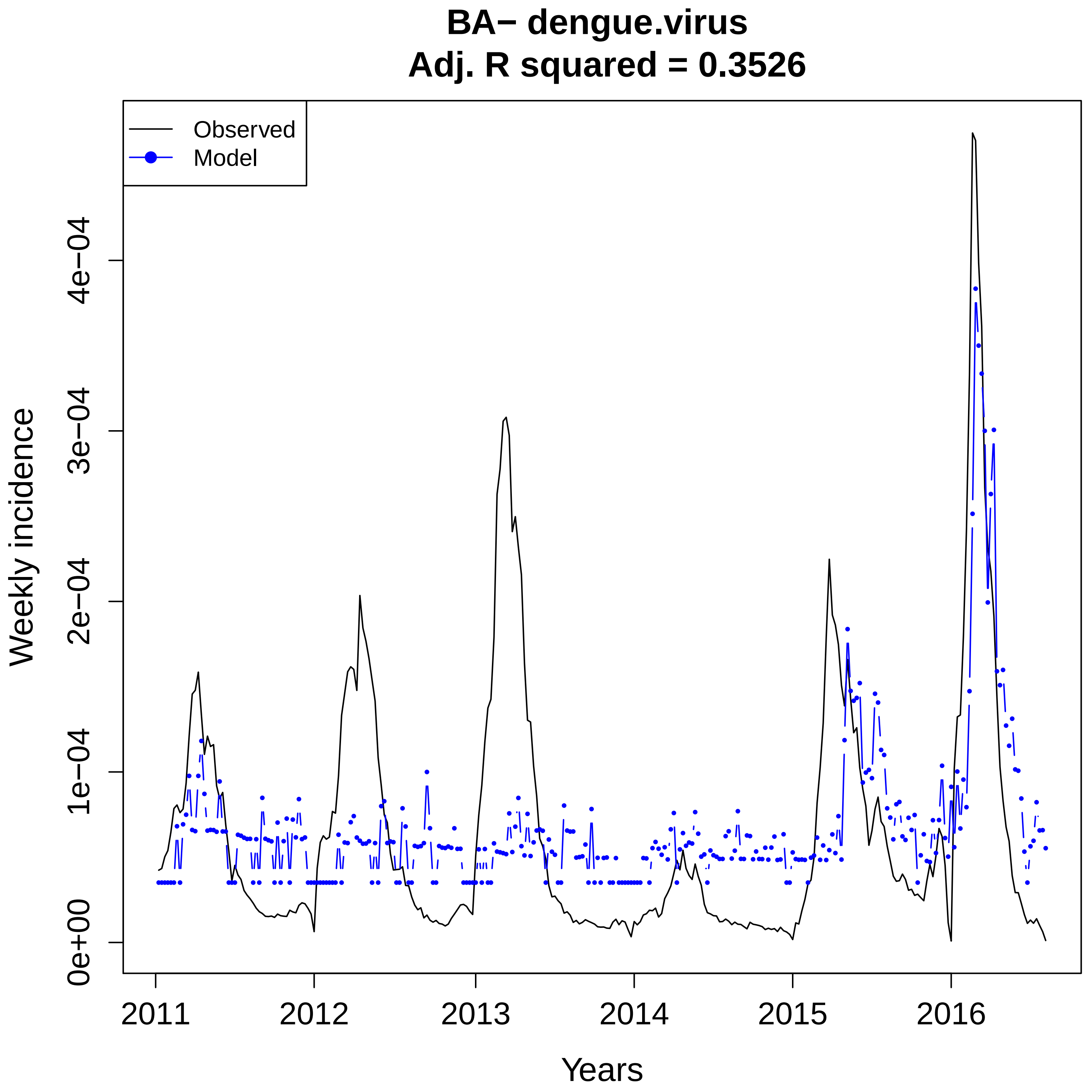

BA− dengue.virus
Adj. R squared = 0.3526
Observed
Model
Weekly incidence
0e+00
1e−04
2e−04
3e−04
4e−04
2011
2012
2013
2014
2015
2016
Years

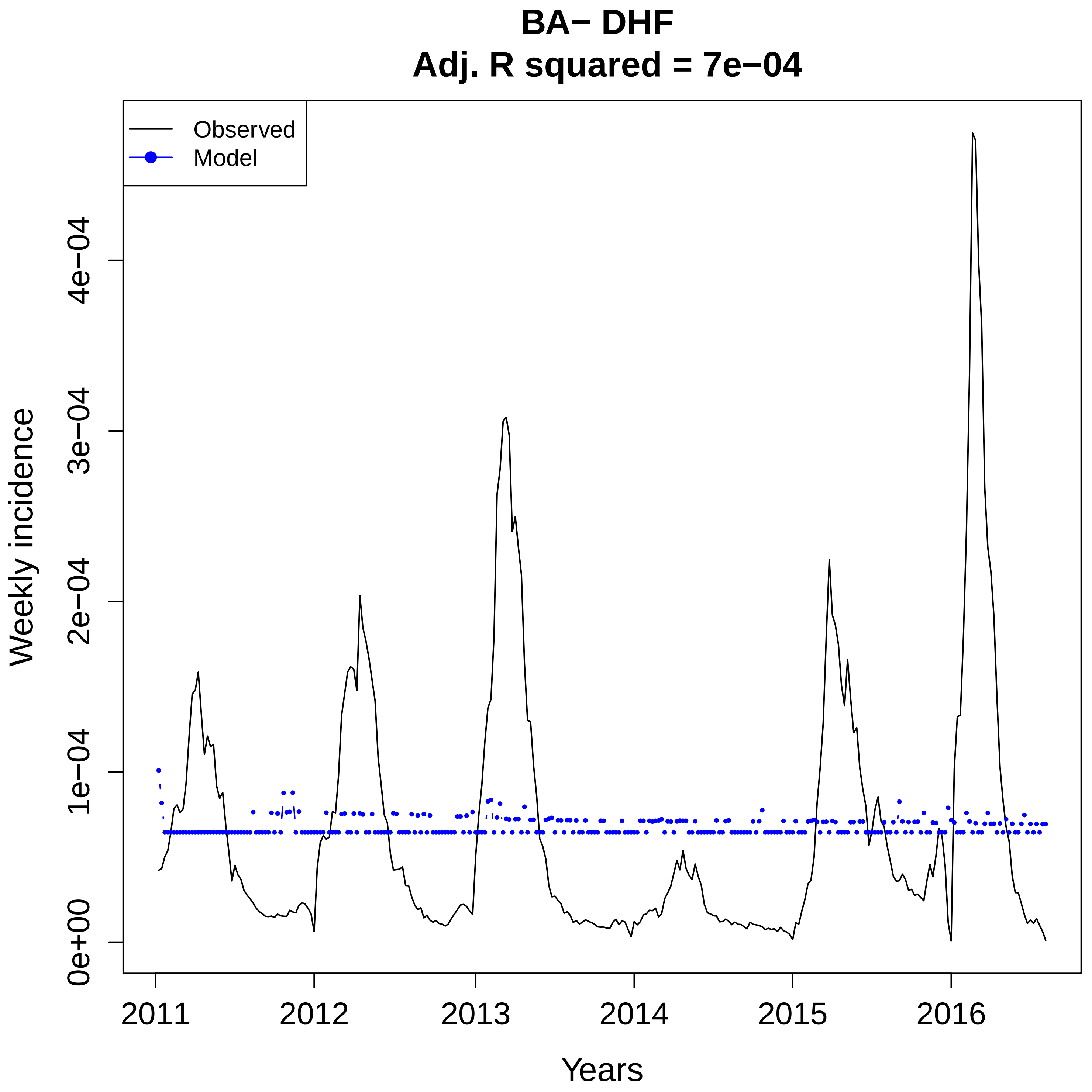
BA− DHF
Adj. R squared = 7e−04
Observed
Model
Weekly incidence
0e+00
1e−04
2e−04
3e−04
4e−04
2011
2012
2013
2014
2015
2016
Years

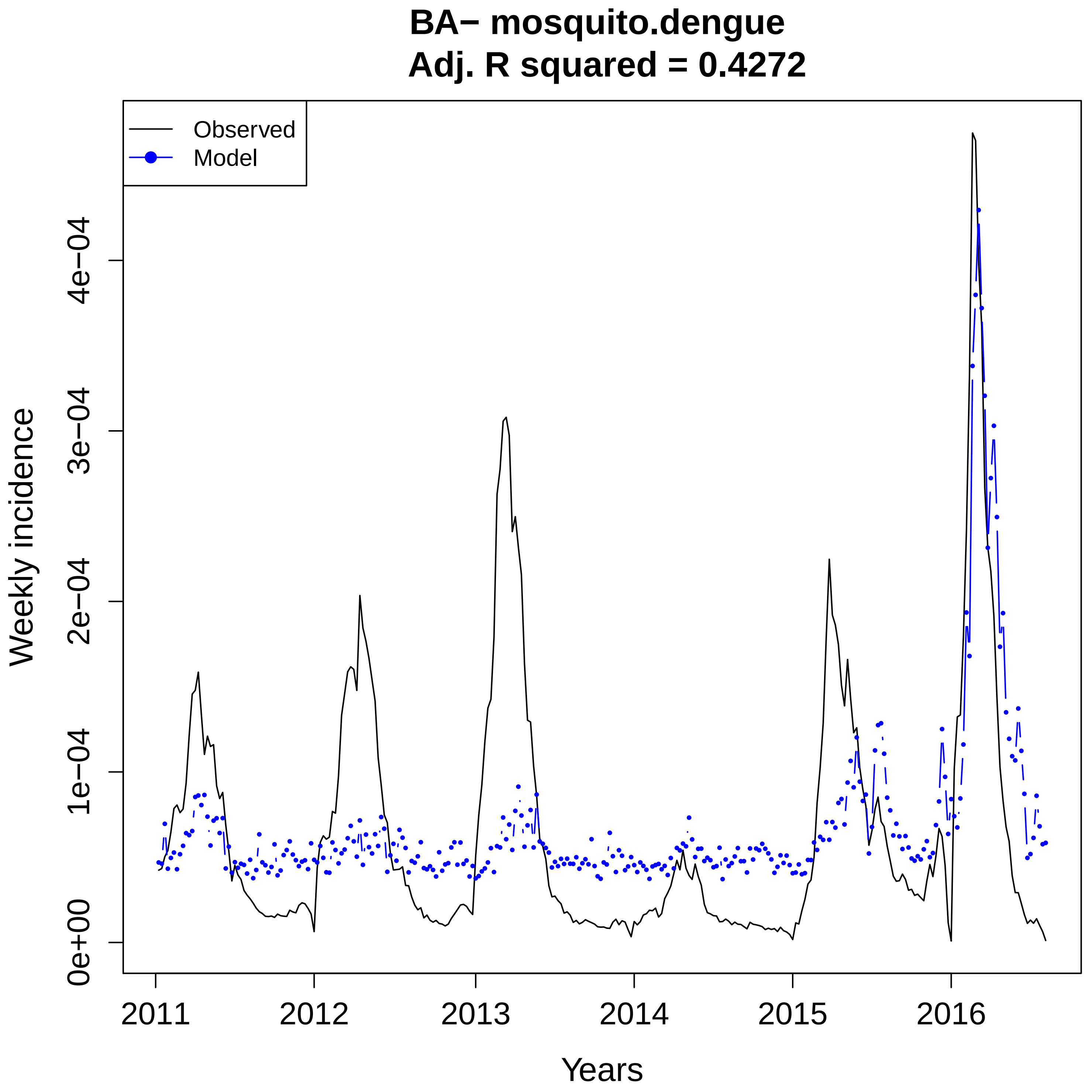
BA− mosquito.dengue
Adj. R squared = 0.4272
Observed
Model
Weekly incidence
0e+00
1e−04
2e−04
3e−04
4e−04
2011
2012
2013
2014
2015
2016
Years

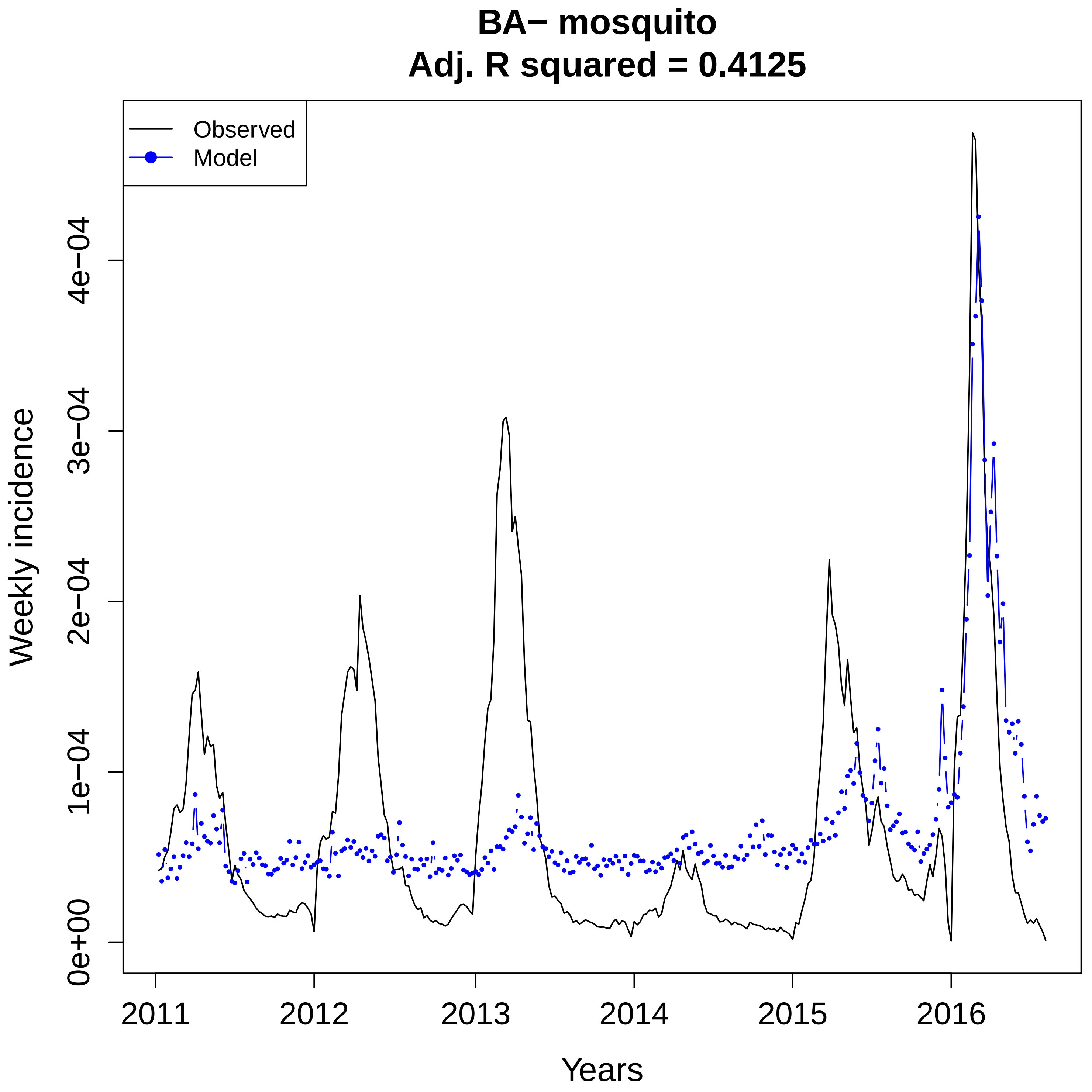
BA− mosquito
Adj. R squared = 0.4125
Observed
Model
Weekly incidence
0e+00
1e−04
2e−04
3e−04
4e−04
2011
2012
2013
2014
2015
2016
Years

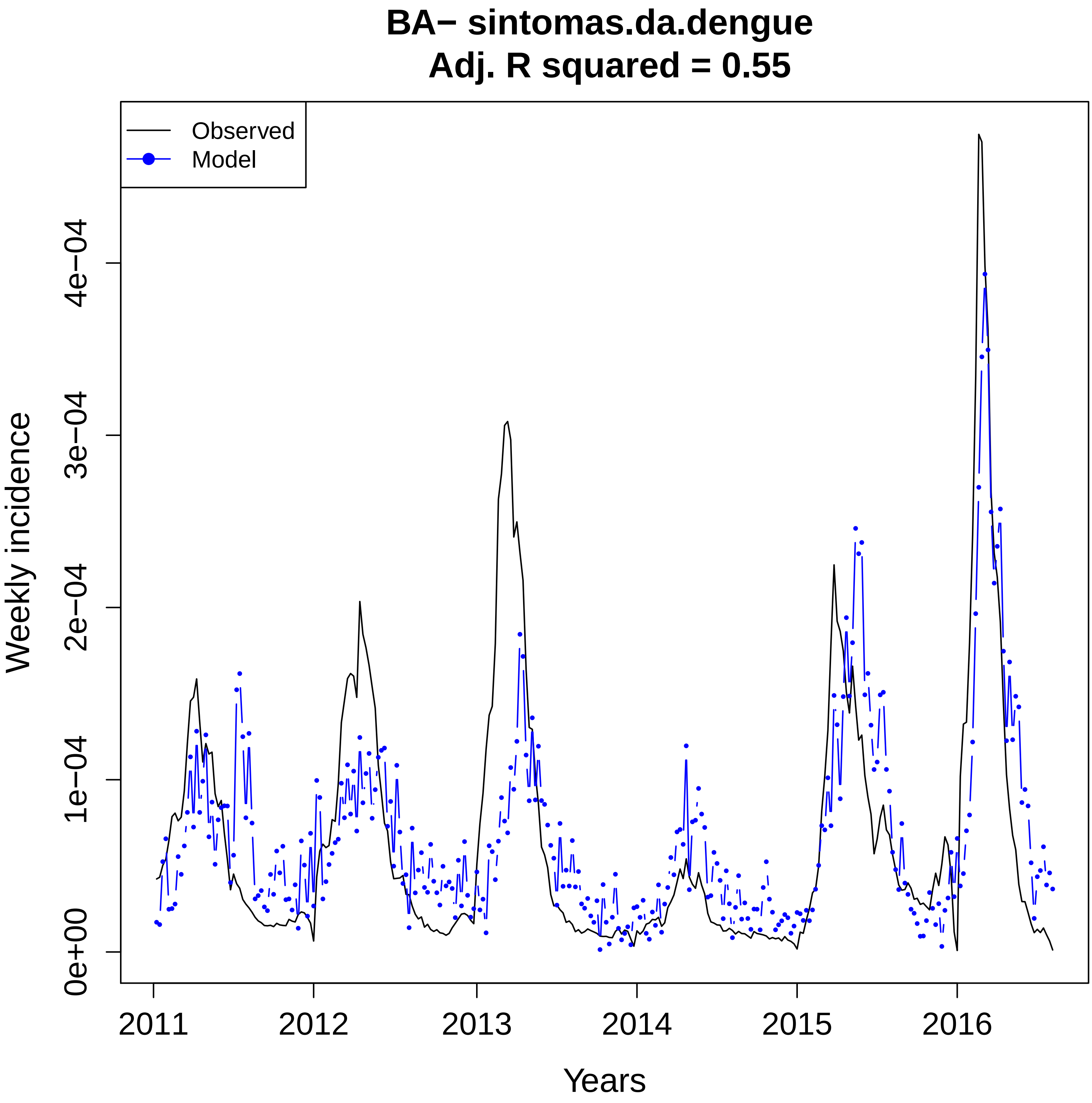

BA− sintomas.da.dengue
Adj. R squared = 0.55
Observed
Model
Weekly incidence
0e+00
1e−04
2e−04
3e−04
4e−04
2011
2012
2013
2014
2015
2016
Years

Ceará (CE)

Dengue incidence vs. Google Health Trends (2011–2016)

Definitions:

- **Combined terms:**
  - **All:** model with all the available terms. n= number of terms
  - **Four:** model with "dengue", "dengue sintomas", "aedes", and "mosquito".
  - **Uncorrelated:** model with non-correlated terms. n = number of uncorrelated terms
- **Individual terms:** Plots developed with one term at a time.

- Terms for all and uncorrelated models can be found in additional file 3.

Combined terms
Adjusted R squared

Ceará (CE)

All
(n = 10)

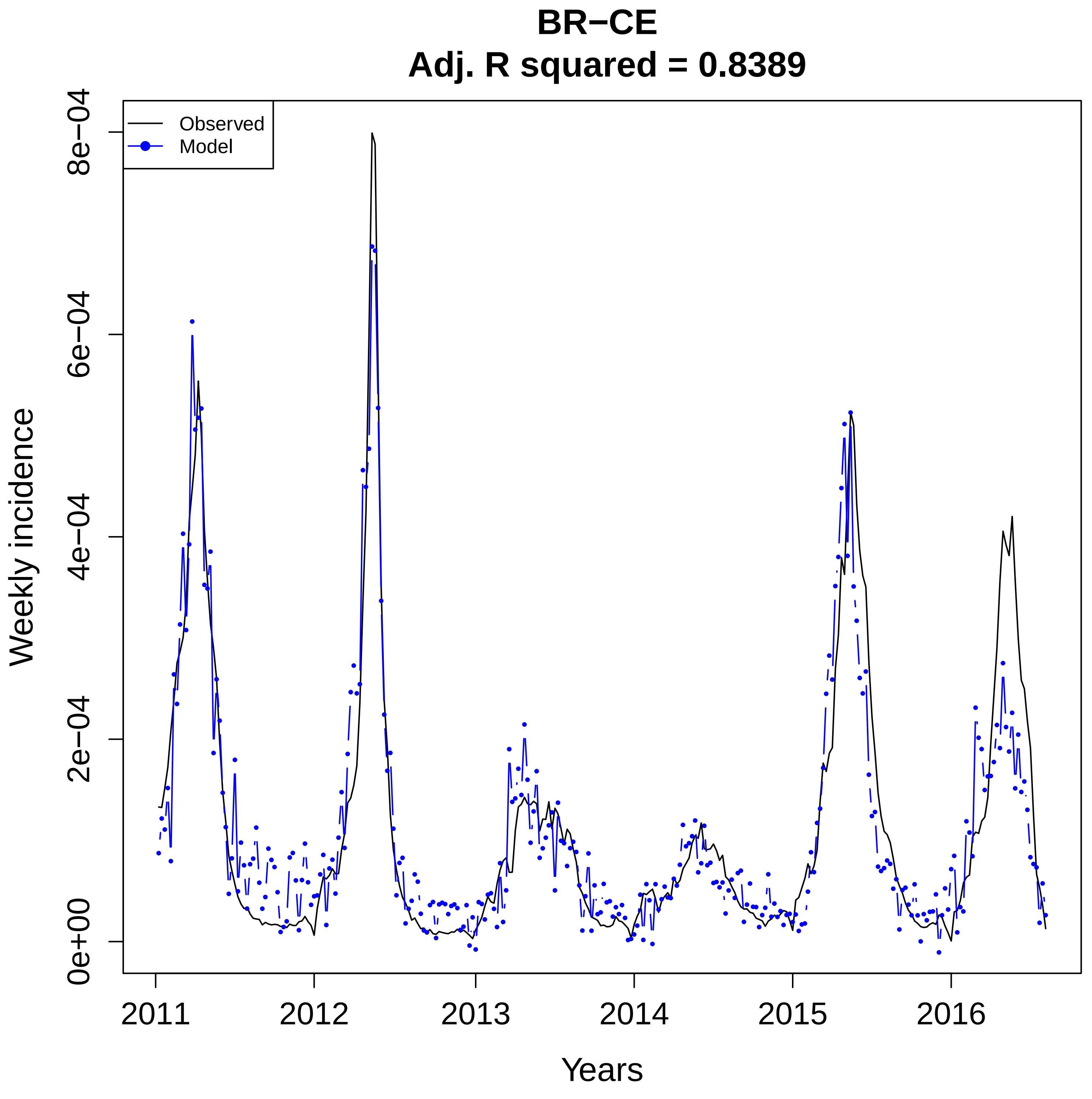

BR−CE
Adj. R squared = 0.8389
Observed
Model
Weekly incidence
0e+00
2e−04
4e−04
6e−04
8e−04
Years
2011
2012
2013
2014
2015
2016

Four

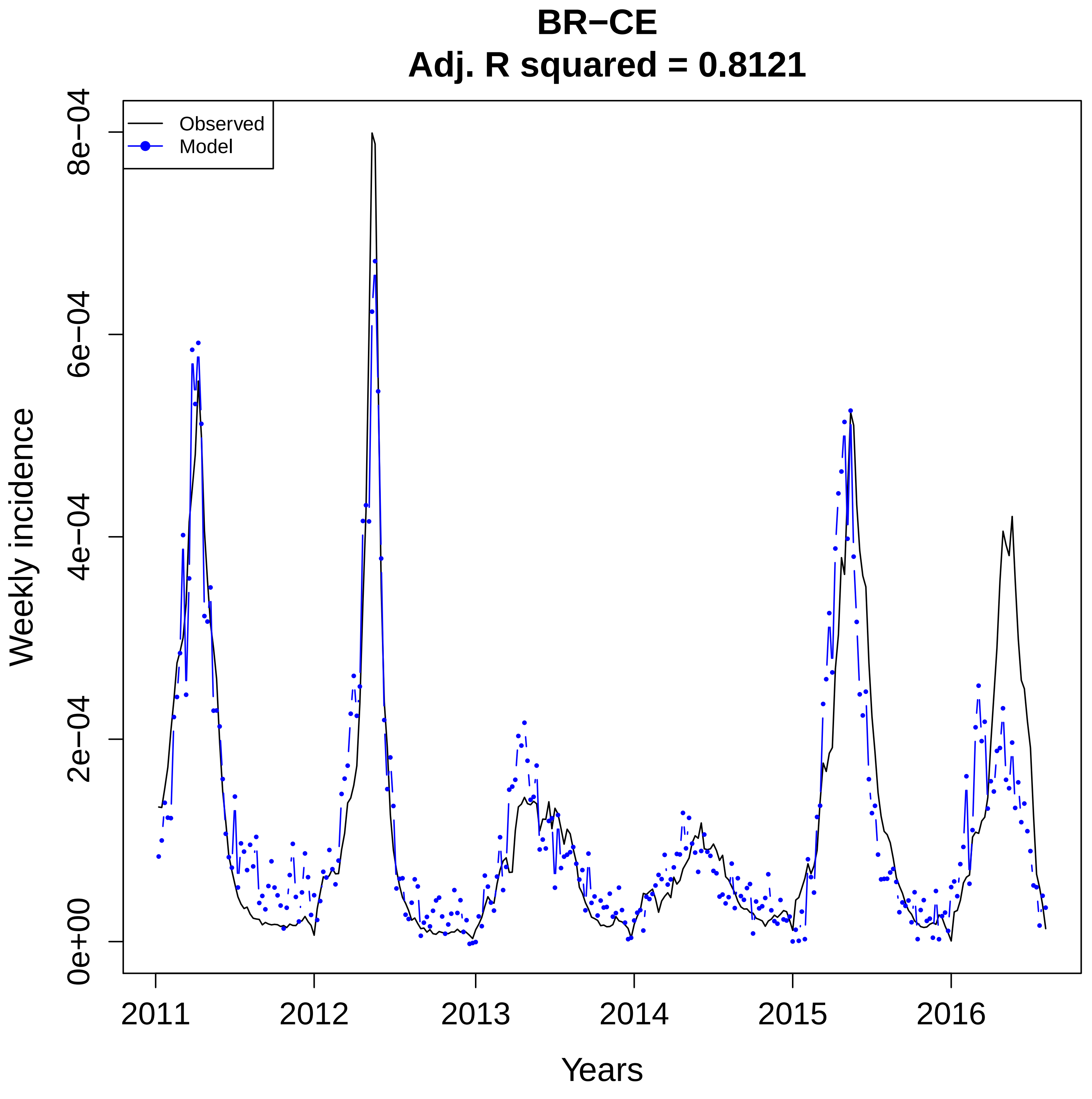

BR−CE
Adj. R squared = 0.8121
Observed
Model
Weekly incidence
0e+00
2e−04
4e−04
6e−04
8e−04
2011
2012
2013
2014
2015
2016
Years

Uncorrelated
(n = 3)

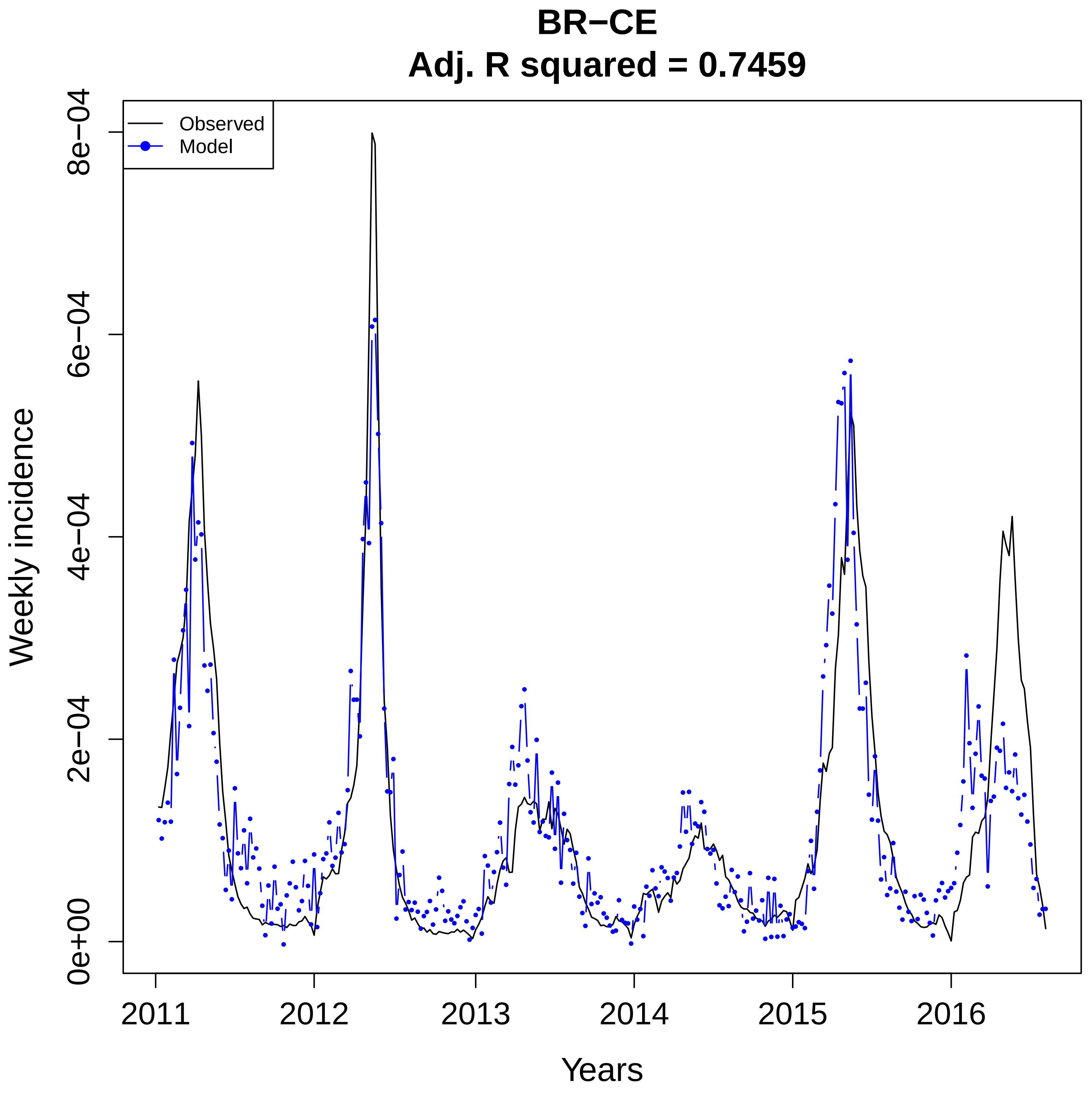

Individual terms
Adjusted R squared

Ceará (CE)

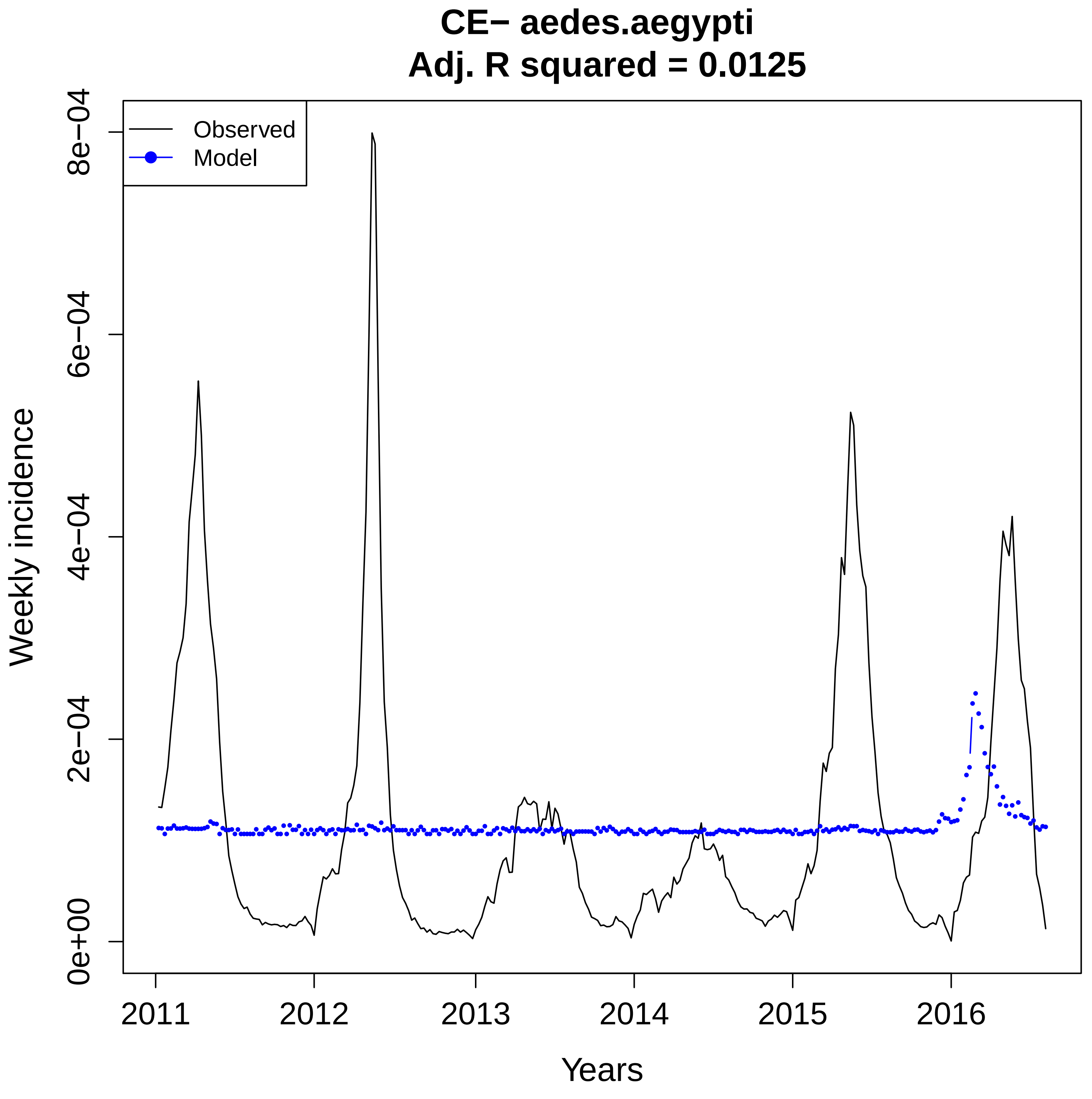
CE− aedes.aegypti
Adj. R squared = 0.0125
Observed
Model
Weekly incidence
0e+00
2e−04
4e−04
6e−04
8e−04
2011
2012
2013
2014
2015
2016
Years

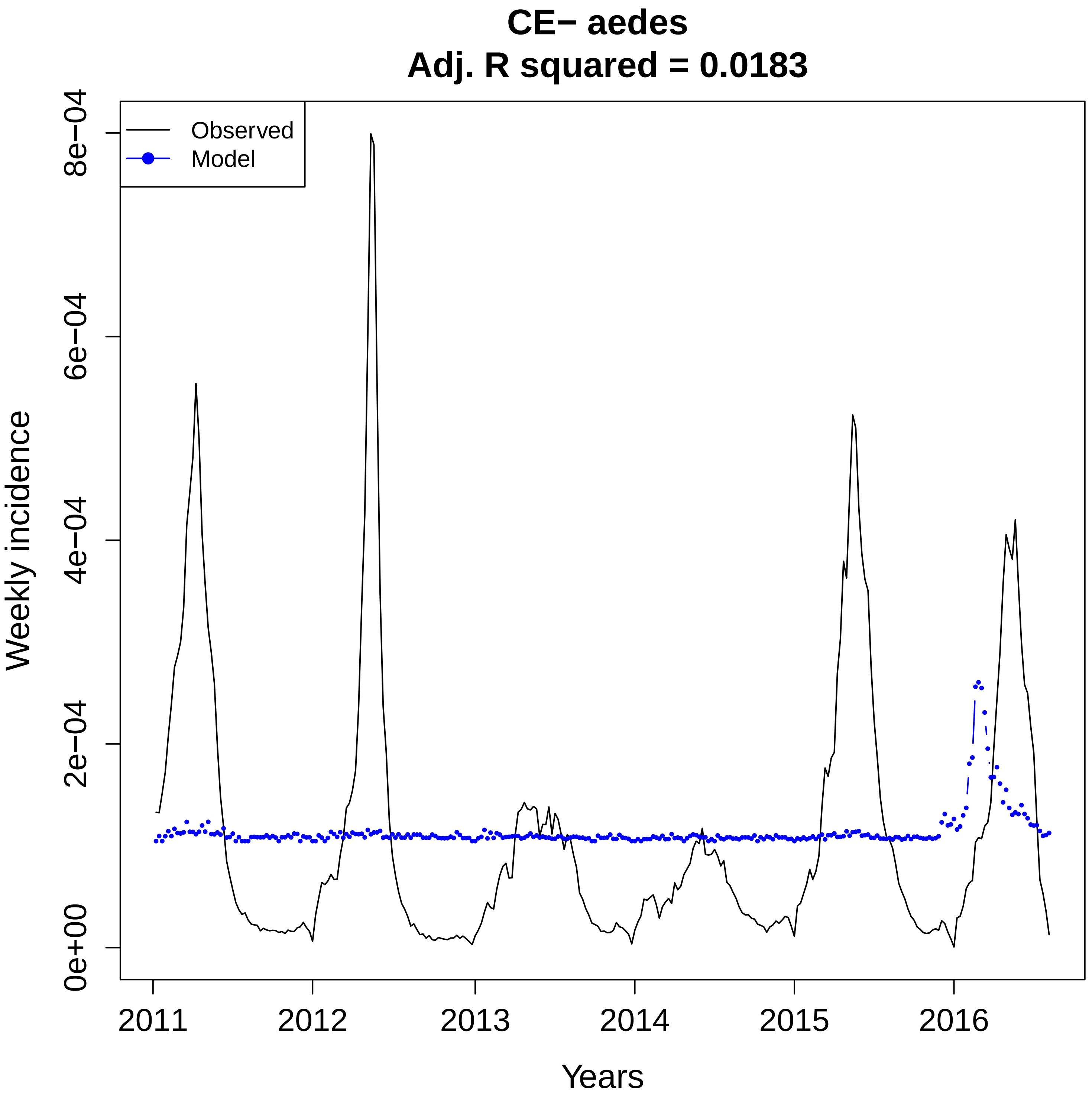

CE− aedes
Adj. R squared = 0.0183
Observed
Model
Weekly incidence
0e+00
2e−04
4e−04
6e−04
8e−04
2011
2012
2013
2014
2015
2016
Years

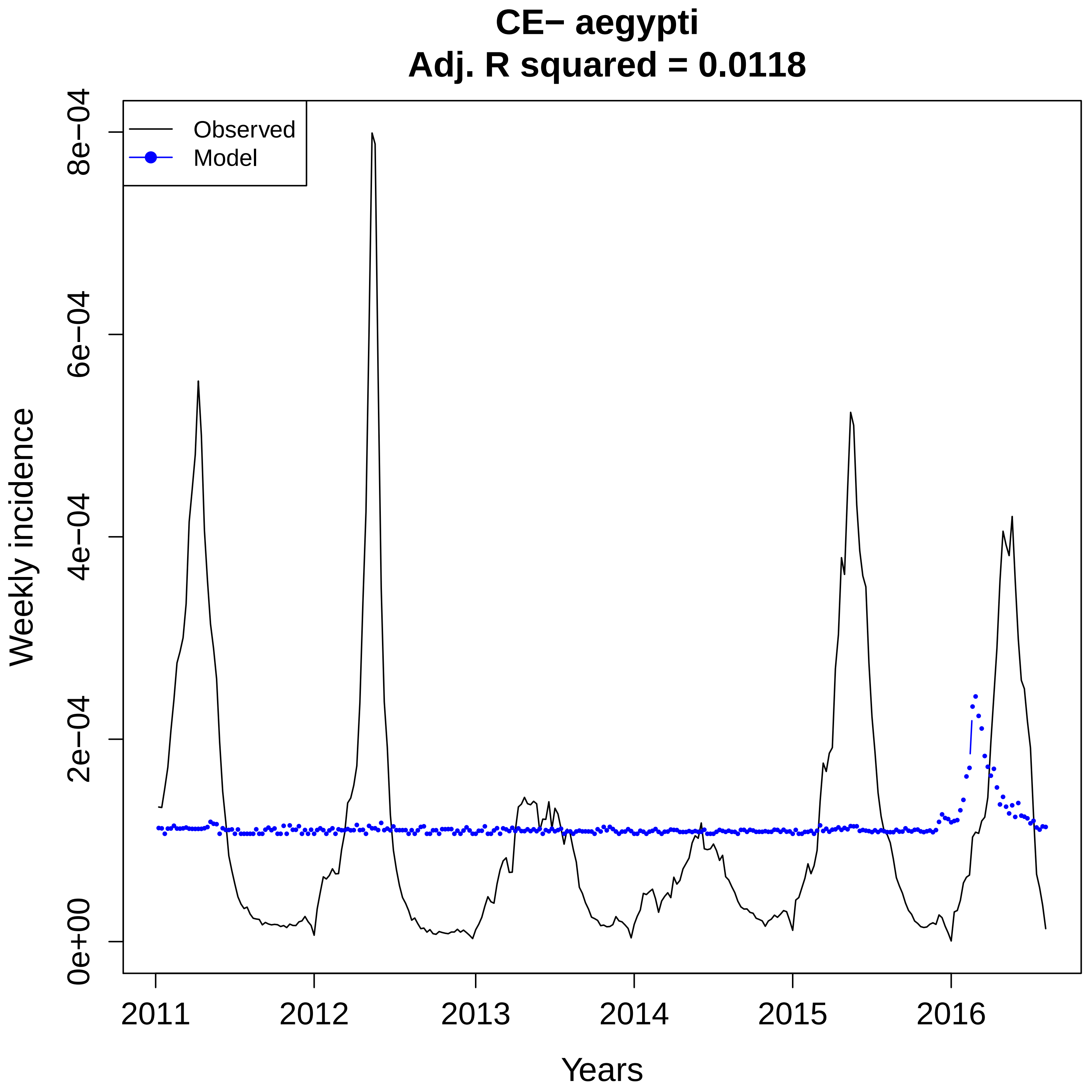
CE− aegypti
Adj. R squared = 0.0118
Observed
Model
Weekly incidence
0e+00
2e−04
4e−04
6e−04
8e−04
2011
2012
2013
2014
2015
2016
Years

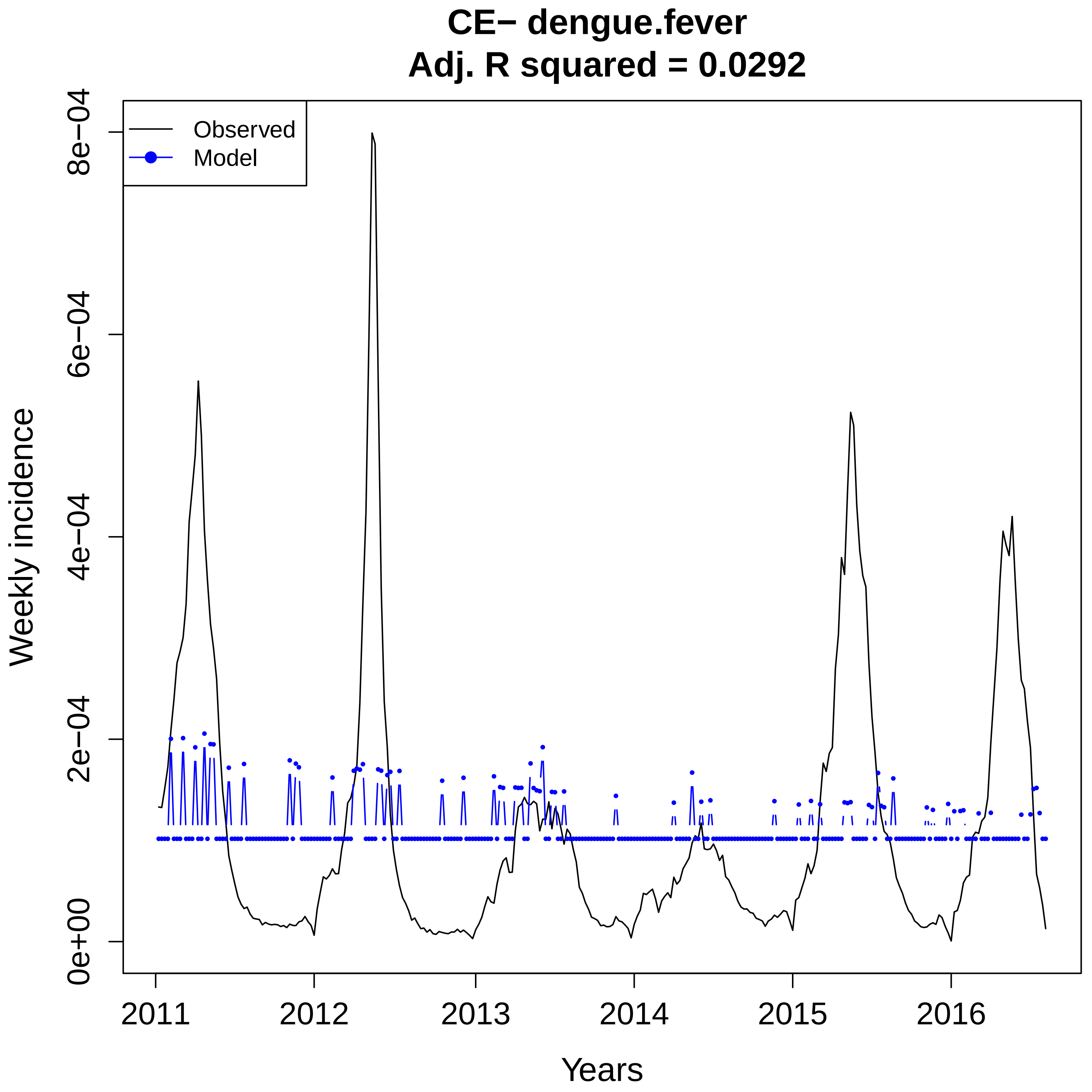
CE− dengue.fever
Adj. R squared = 0.0292
Observed
Model
Weekly incidence
0e+00
2e−04
4e−04
6e−04
8e−04
2011
2012
2013
2014
2015
2016
Years

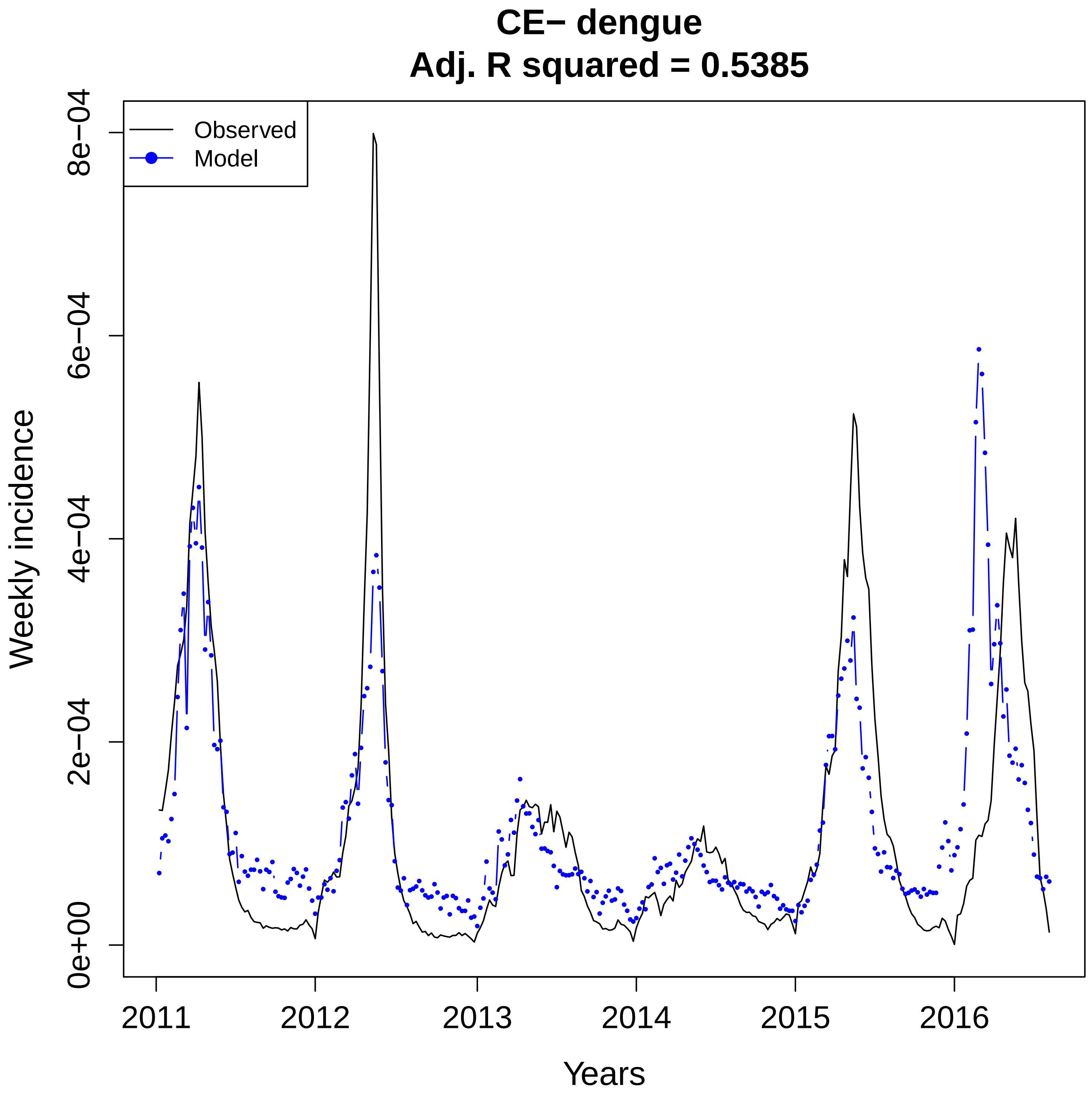
CE− dengue
Adj. R squared = 0.5385
Observed
Model
Weekly incidence
0e+00
2e−04
4e−04
6e−04
8e−04
2011
2012
2013
2014
2015
2016
Years

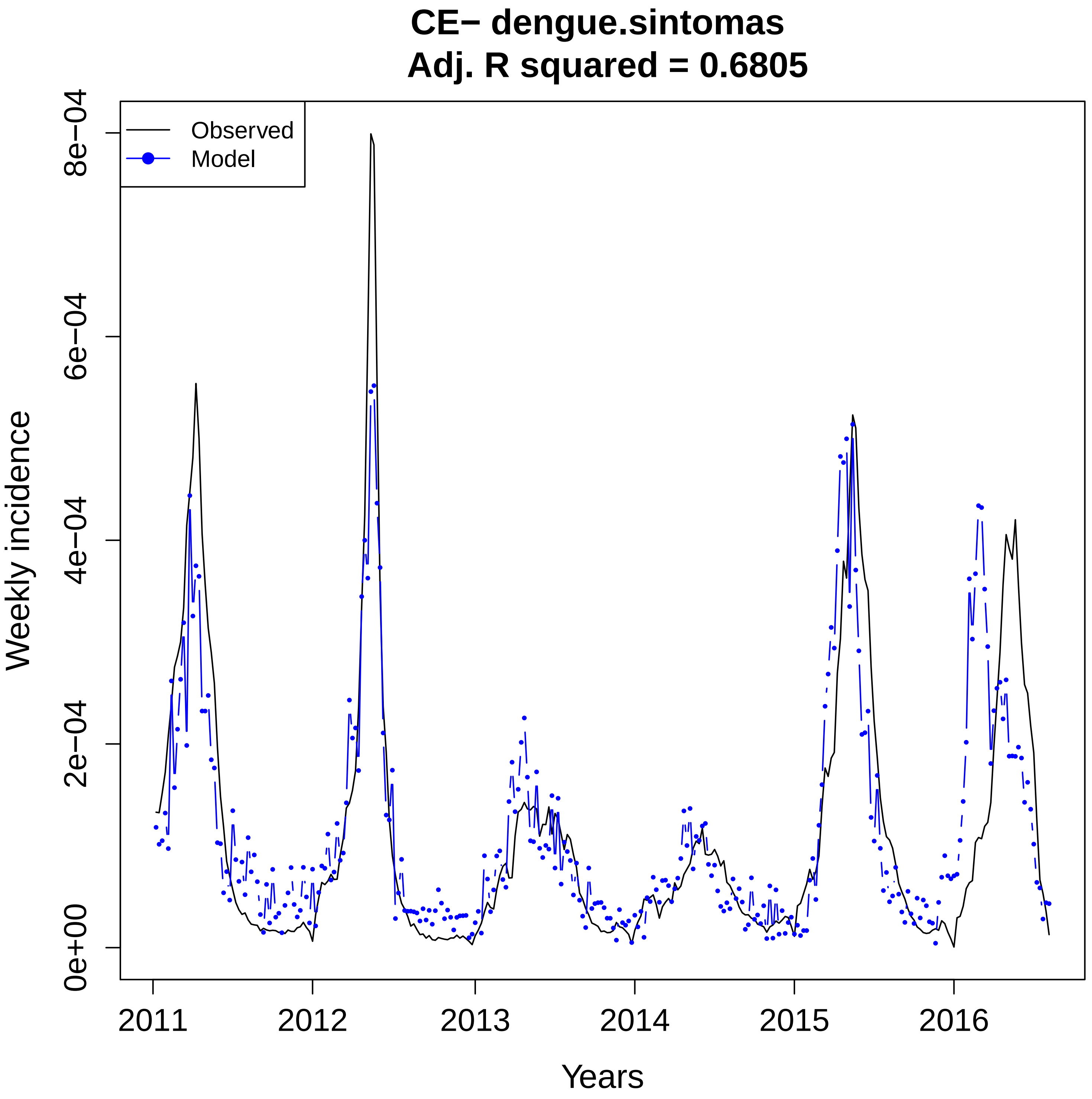

CE− dengue.sintomas
Adj. R squared = 0.6805
Observed
Model
Weekly incidence
0e+00
2e−04
4e−04
6e−04
8e−04
2011
2012
2013
2014
2015
2016
Years

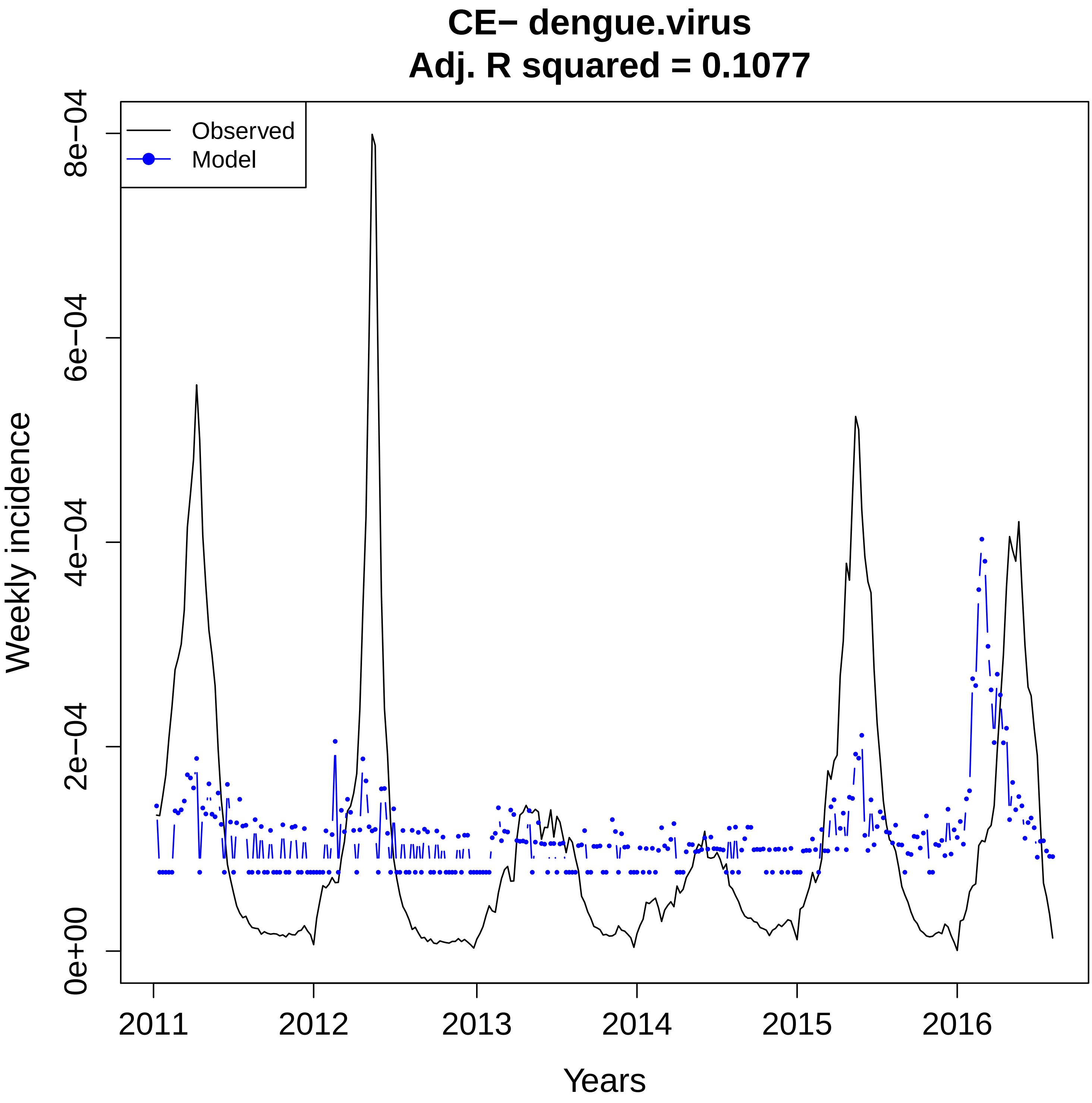
CE− dengue.virus
Adj. R squared = 0.1077
Observed
Model
Weekly incidence
0e+00
2e−04
4e−04
6e−04
8e−04
2011
2012
2013
2014
2015
2016
Years

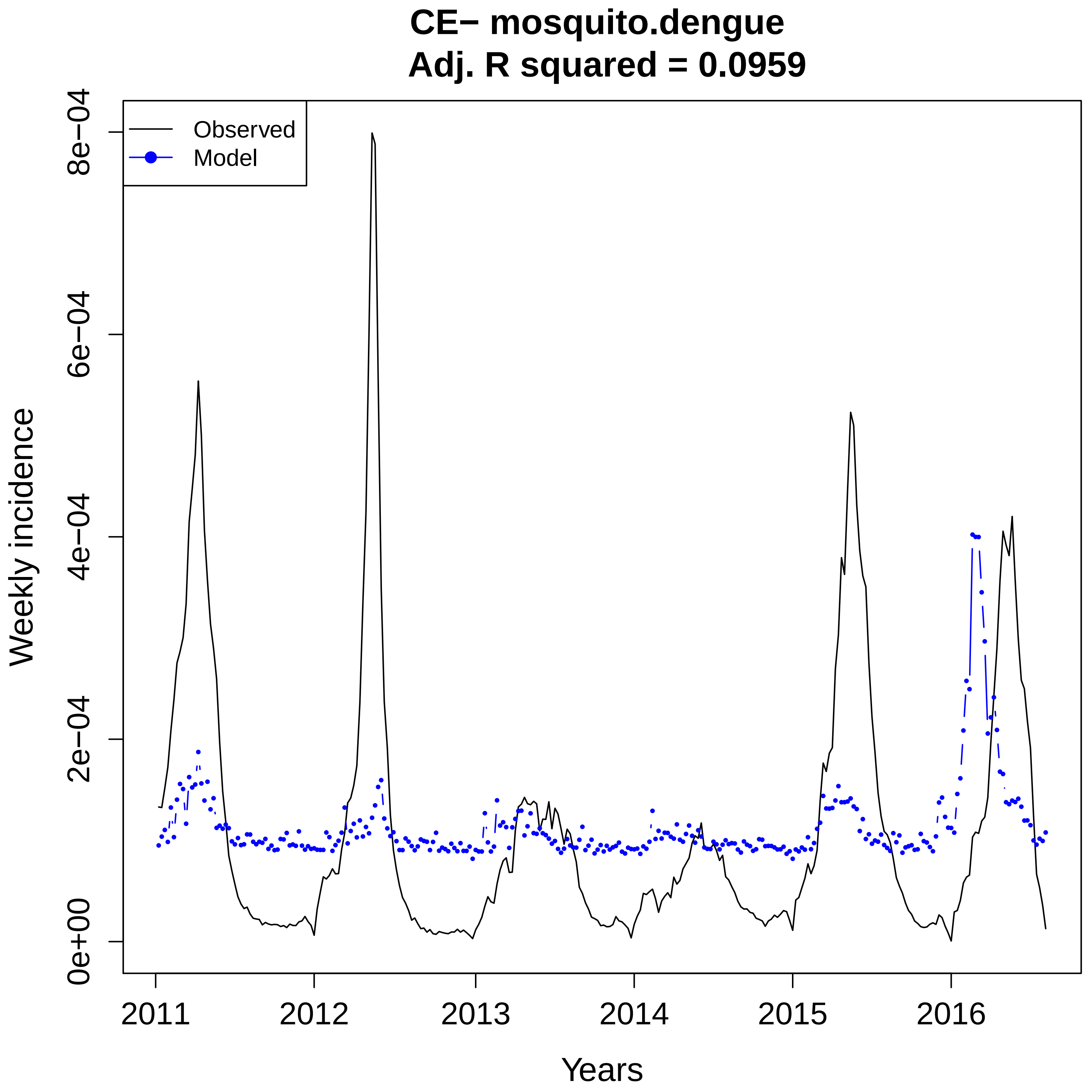
CE− mosquito.dengue
Adj. R squared = 0.0959
Observed
Model
Weekly incidence
0e+00
2e−04
4e−04
6e−04
8e−04
2011
2012
2013
2014
2015
2016
Years

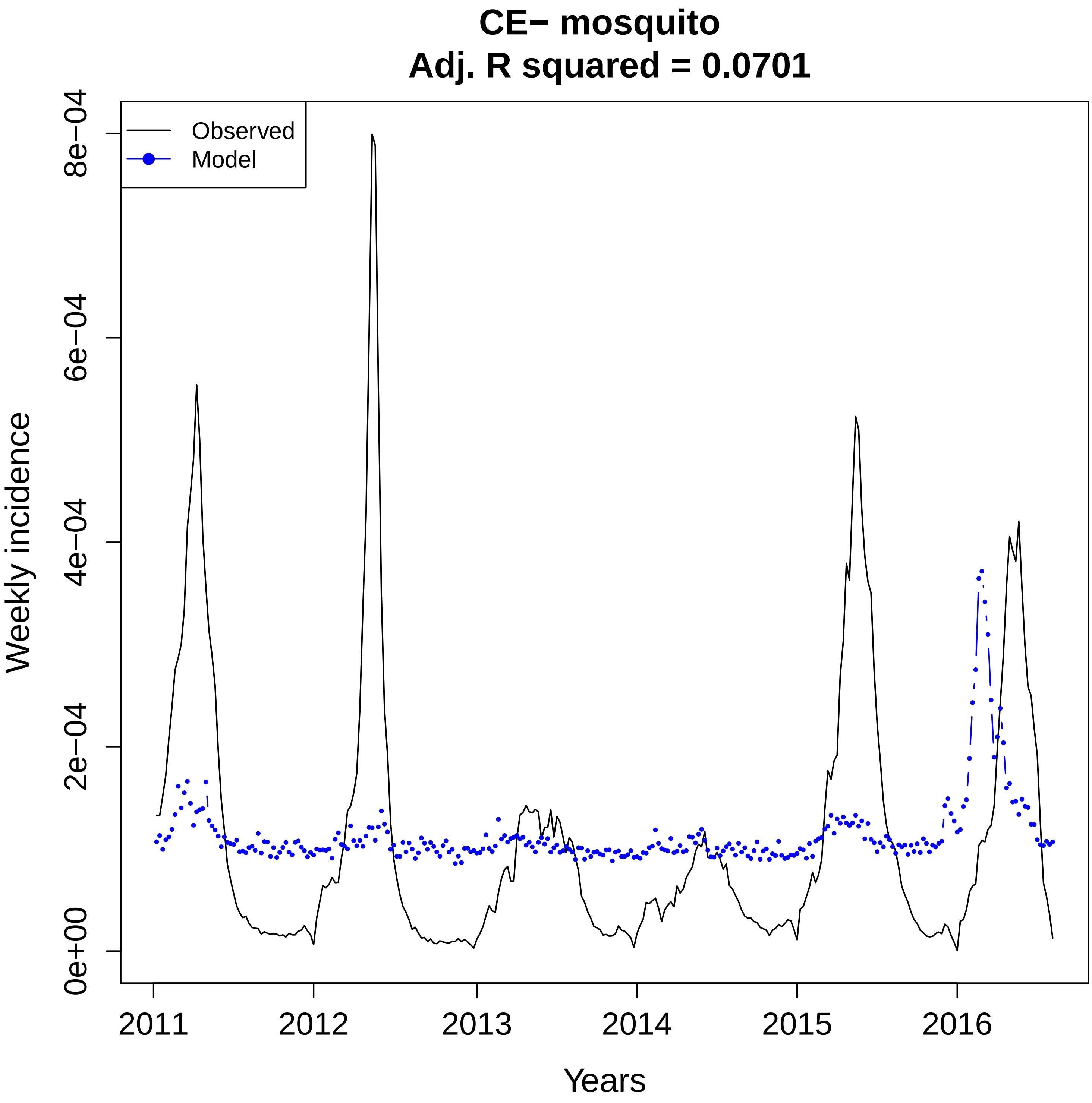

CE− mosquito
Adj. R squared = 0.0701
Observed
Model
Weekly incidence
0e+00
2e−04
4e−04
6e−04
8e−04
2011
2012
2013
2014
2015
2016
Years

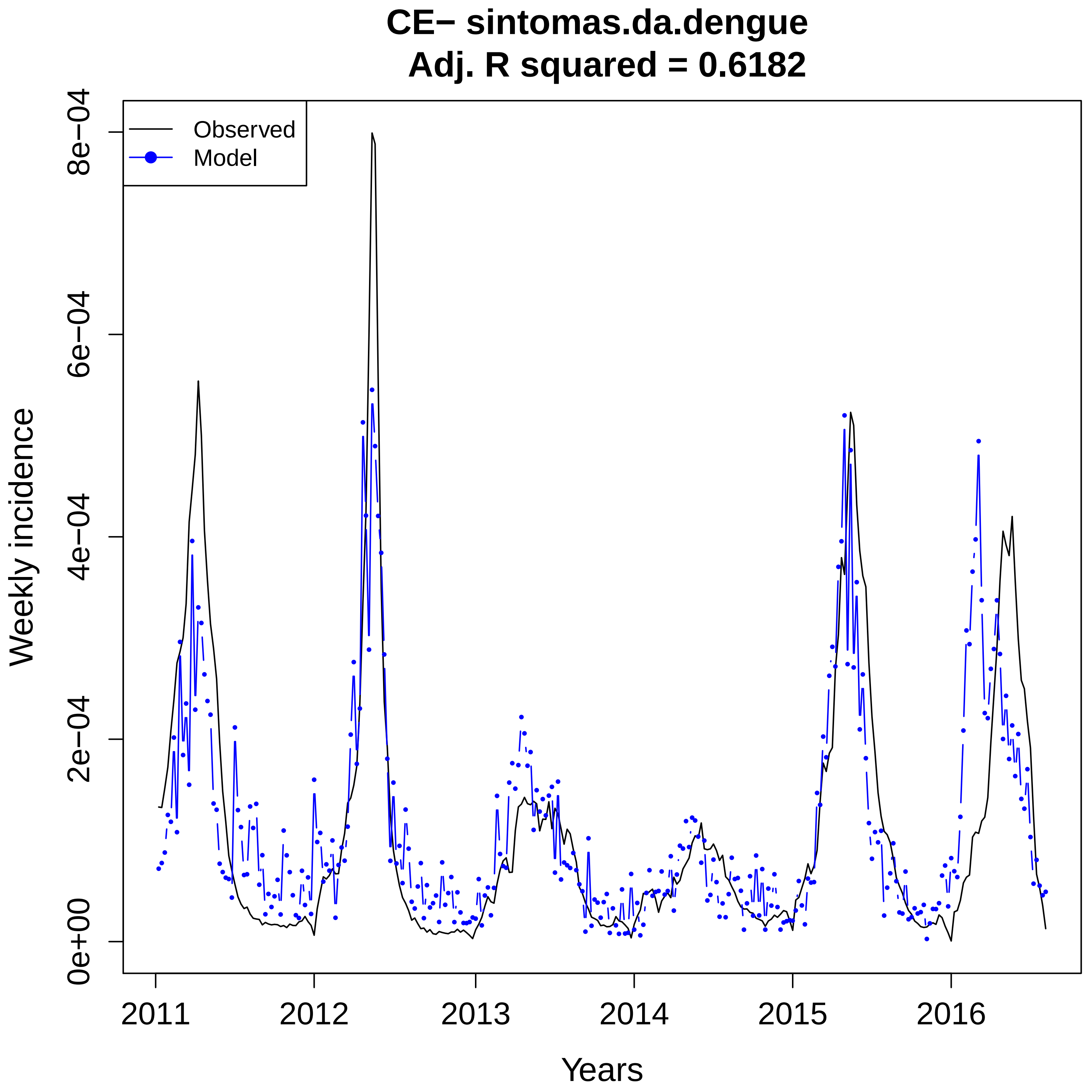

CE− sintomas.da.dengue
Adj. R squared = 0.6182
Observed
Model
Weekly incidence
0e+00
2e−04
4e−04
6e−04
8e−04
2011
2012
2013
2014
2015
2016
Years

Distrito Federal (DF)

Dengue incidence vs. Google Health Trends (2014–2016)

Definitions:

- **Combined terms:**
  - **All:** model with all the available terms. n= number of terms
  - **Four:** model with "dengue", "dengue sintomas", "aedes", and "mosquito".
  - **Uncorrelated:** model with non-correlated terms. n = number of uncorrelated terms
- **Individual terms:** Plots developed with one term at a time.

- Terms for all and uncorrelated models can be found in additional file 3.

Combined terms
Adjusted R Squared

Distrito Federal (DF)

All terms
(n = 10)

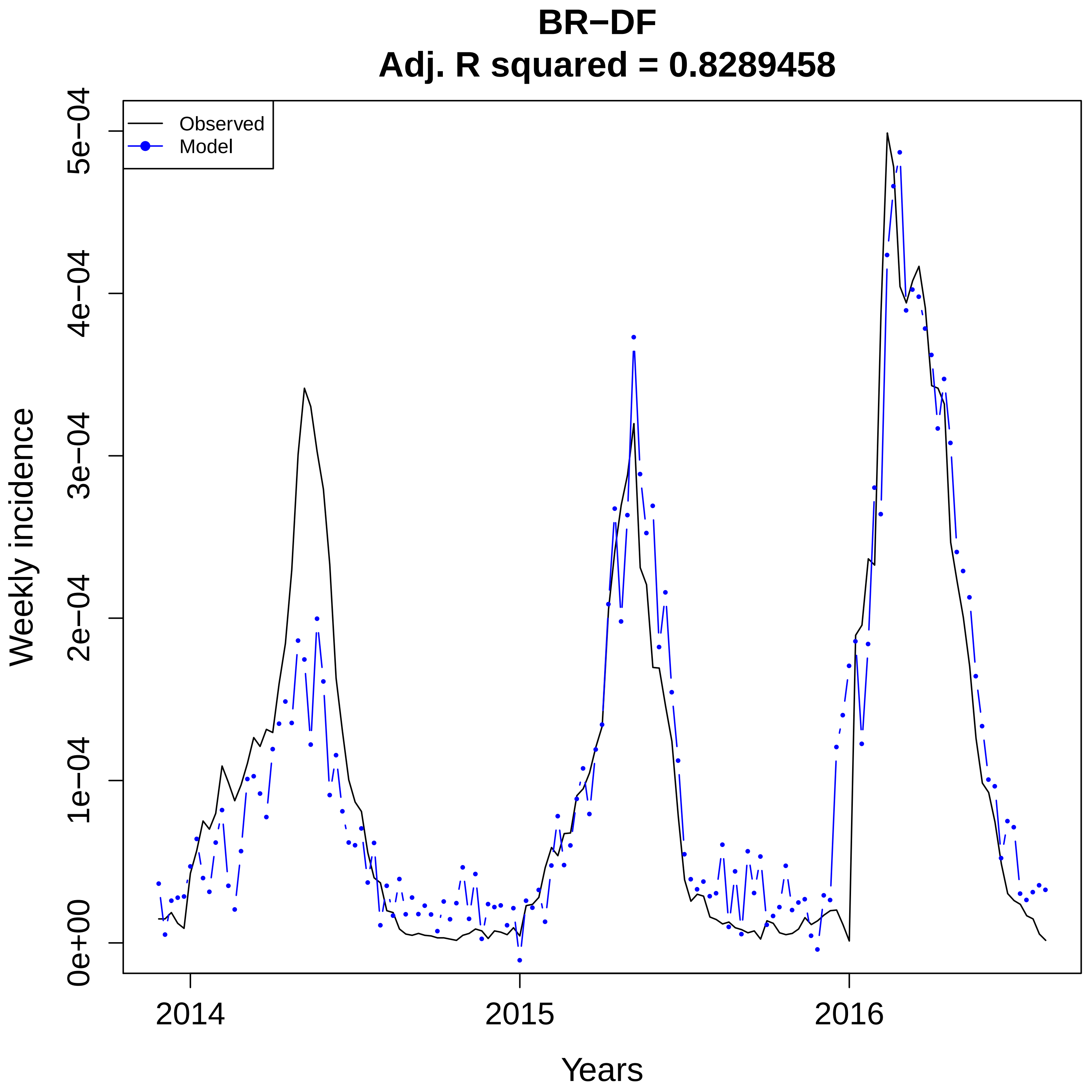

Four terms

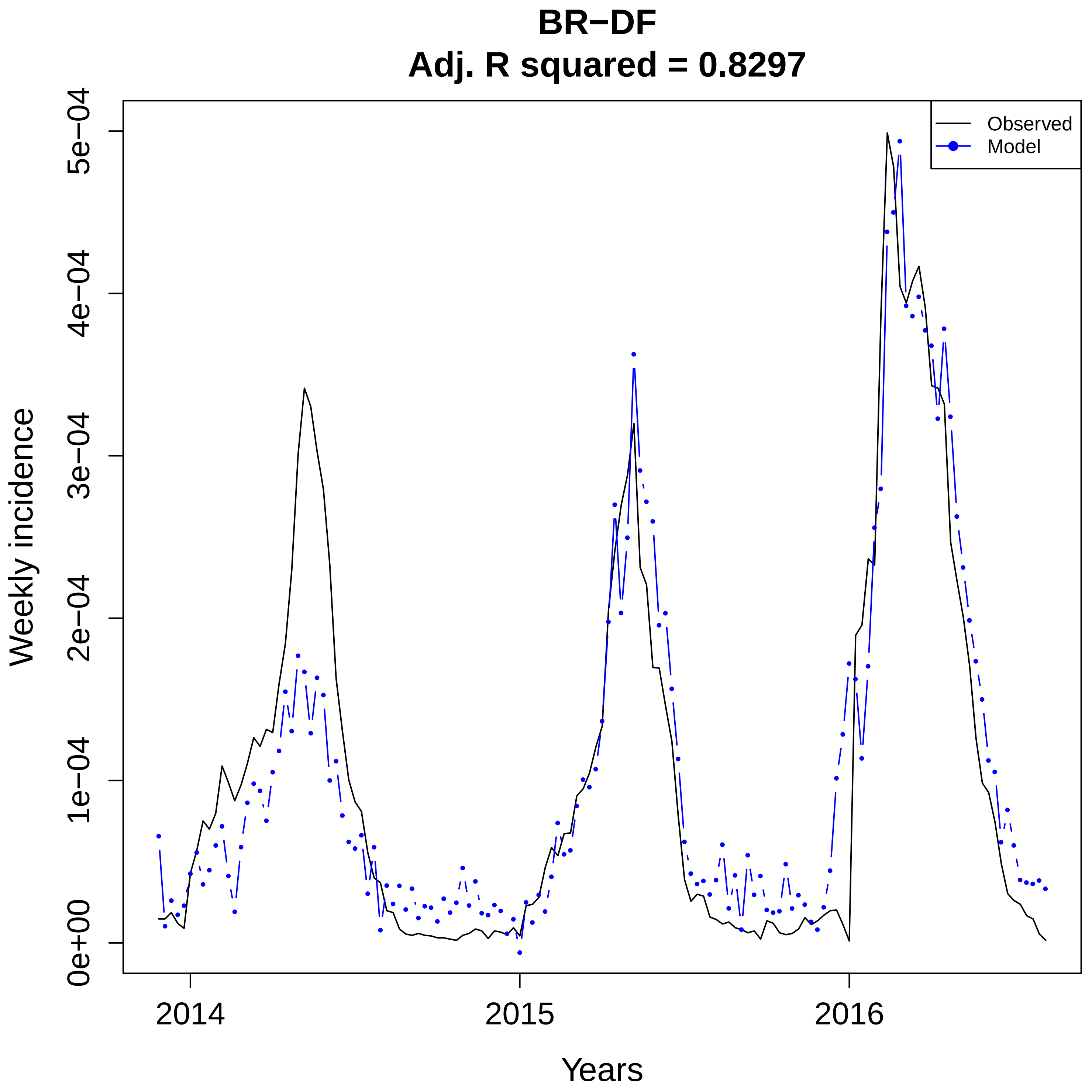
BR−DF
Adj. R squared = 0.8297
Observed
Model
Weekly incidence
0e+00
1e−04
2e−04
3e−04
4e−04
5e−04
2014
2015
2016
Years

Uncorrelated
(n = 2)

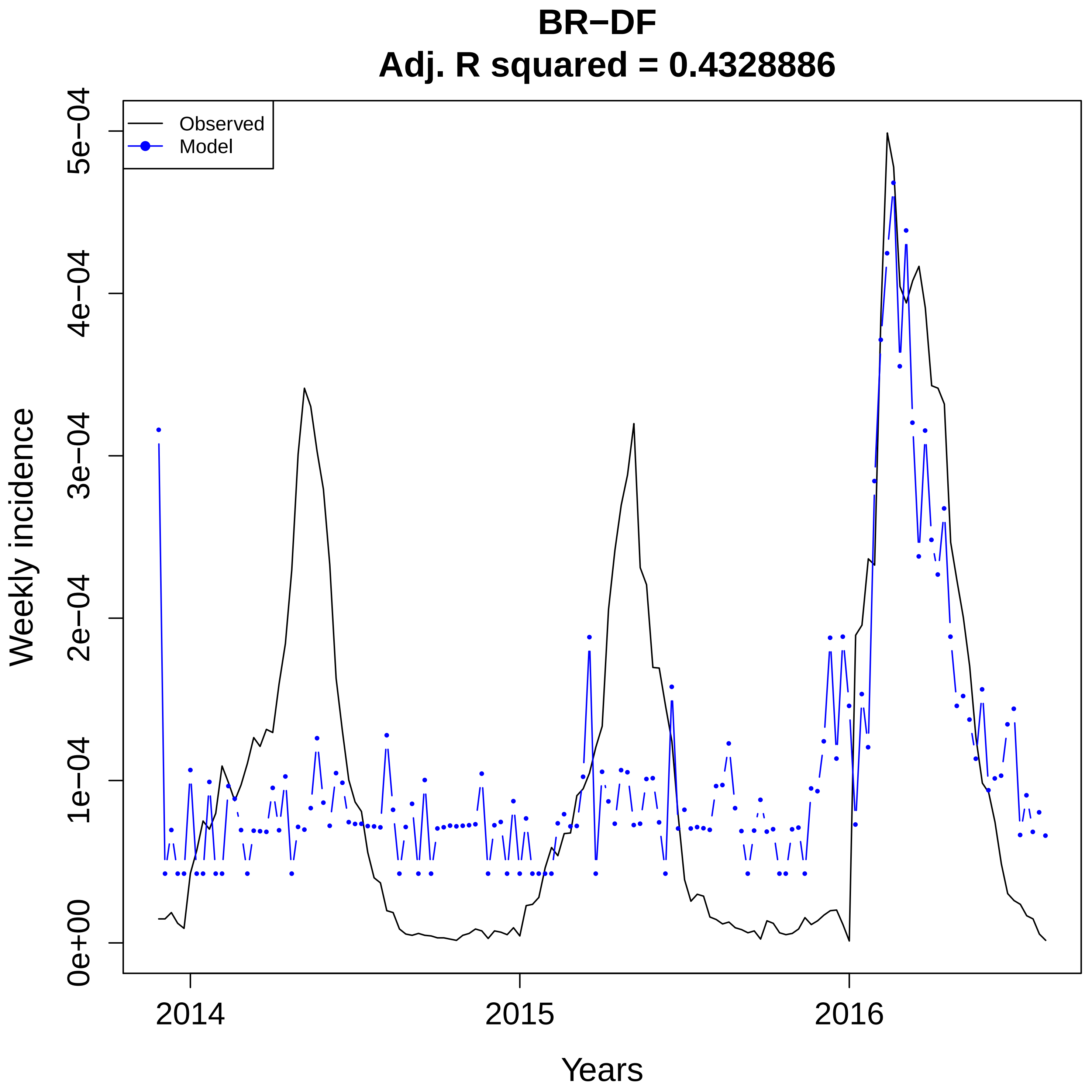

Individual terms
Adjusted R Squared

Distrito Federal (DF)

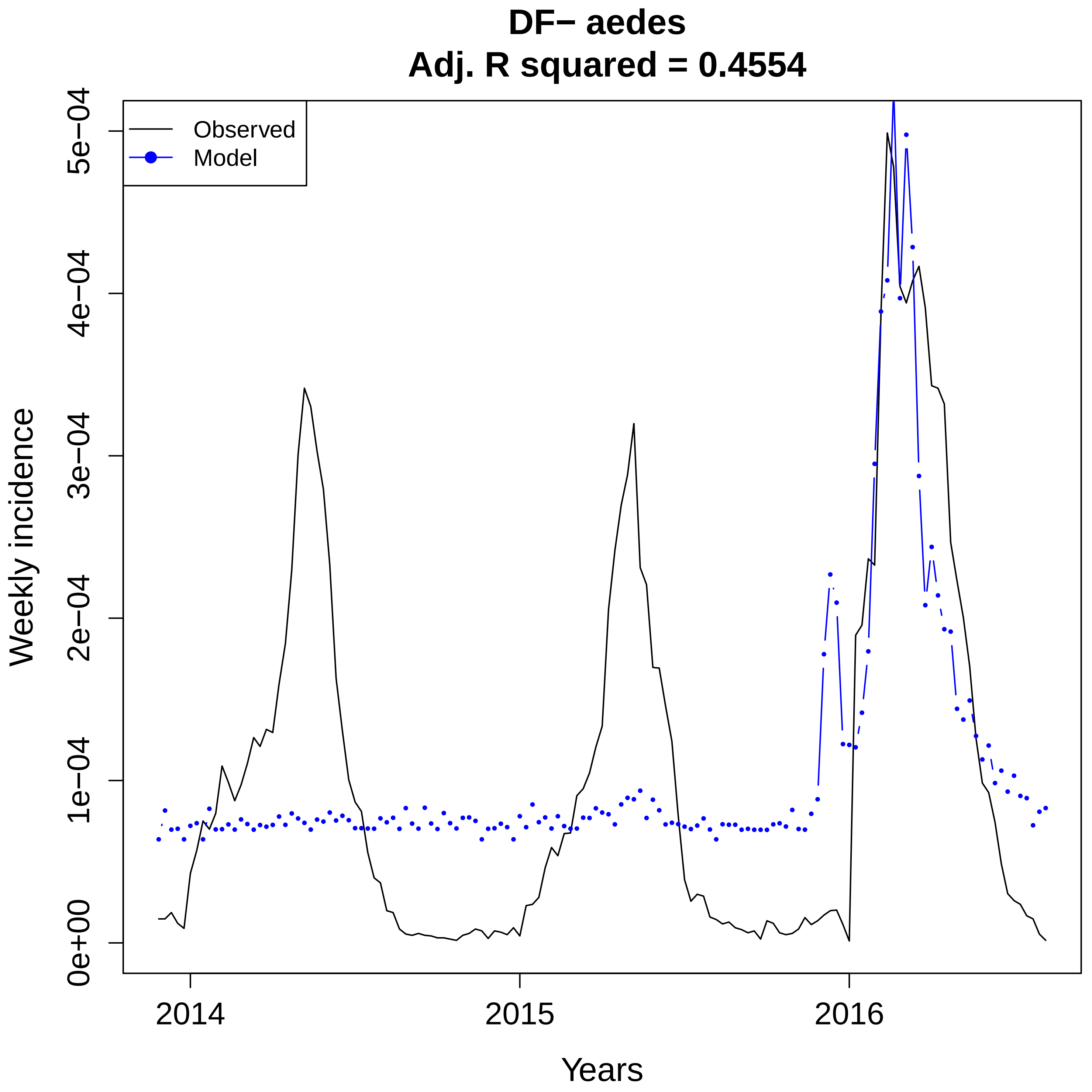
DF− aedes
Adj. R squared = 0.4554
Observed
Model
Weekly incidence
0e+00
1e−04
2e−04
3e−04
4e−04
5e−04
2014
2015
2016
Years

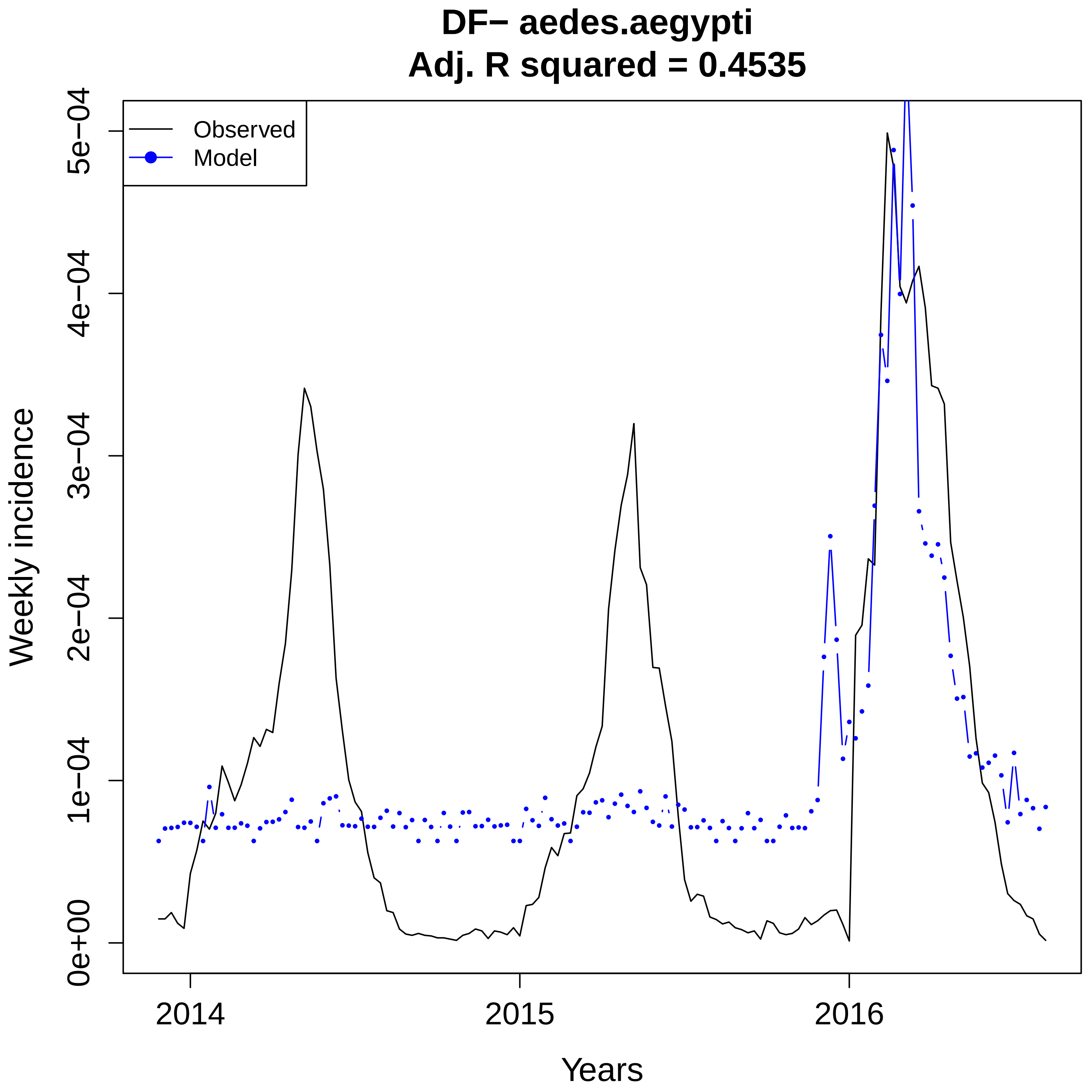
DF− aedes.aegypti
Adj. R squared = 0.4535
Observed
Model
Weekly incidence
0e+00
1e−04
2e−04
3e−04
4e−04
5e−04
2014
2015
2016
Years

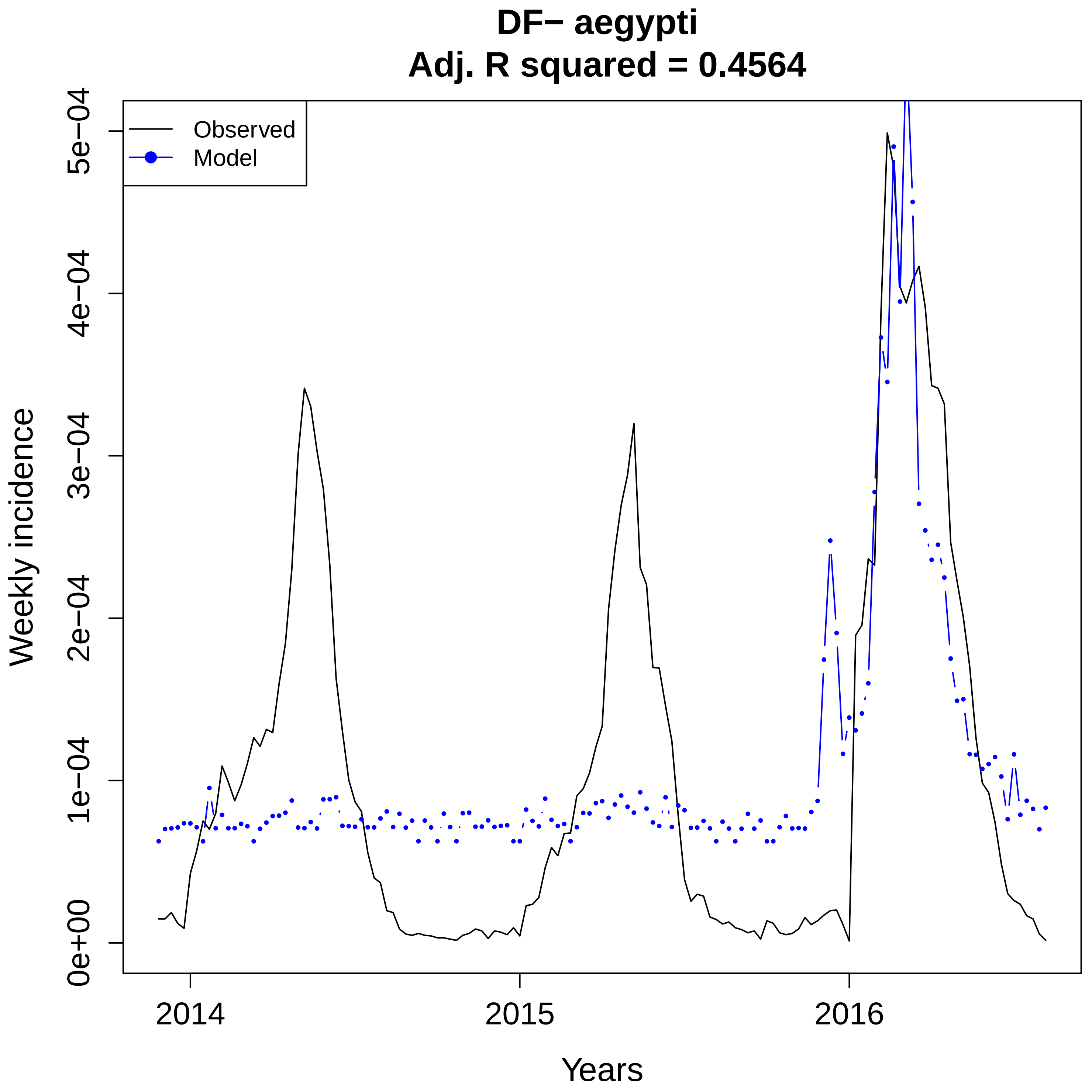

DF− aegypti
Adj. R squared = 0.4564
Observed
Model
Weekly incidence
0e+00
1e−04
2e−04
3e−04
4e−04
5e−04
2014
2015
2016
Years

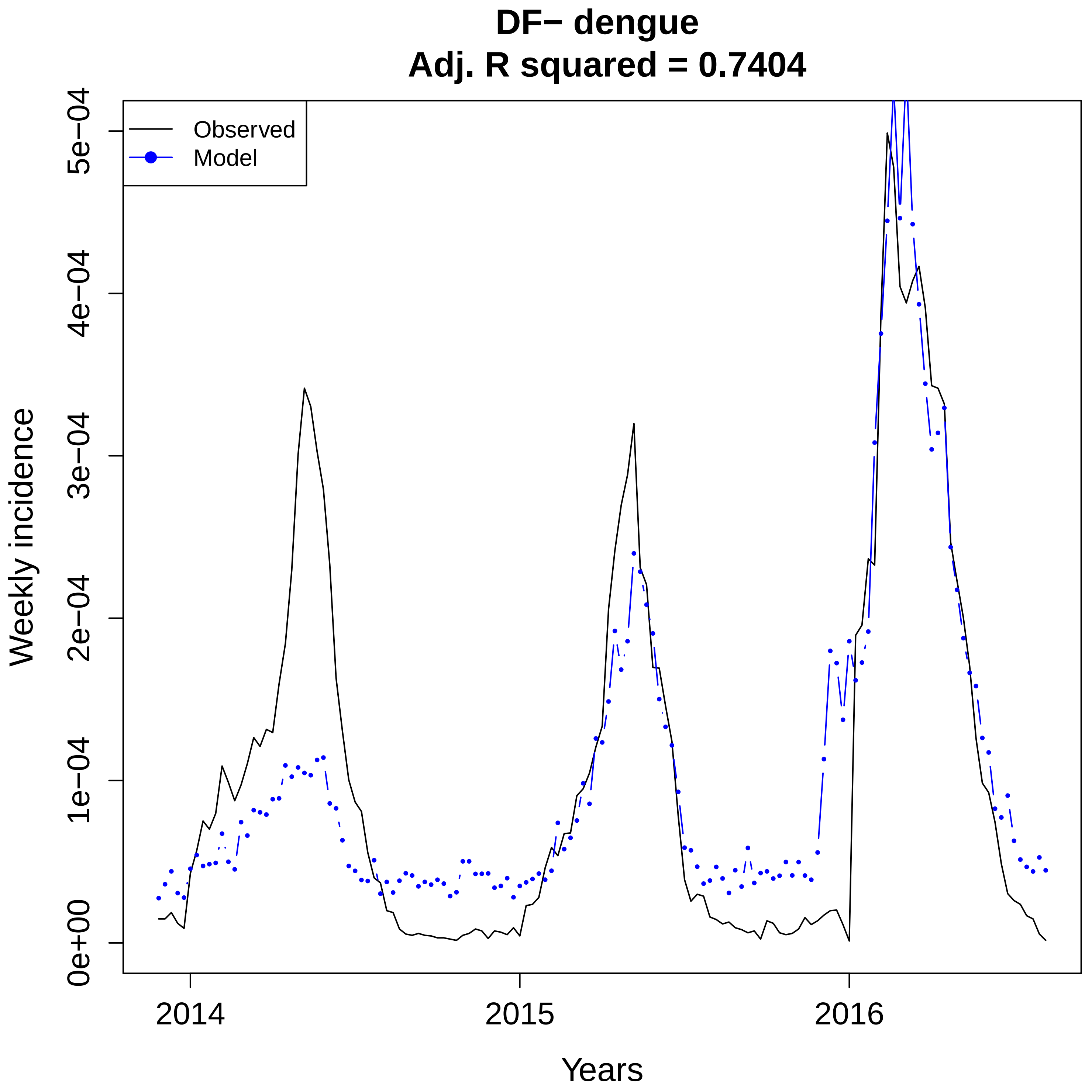

DF− dengue
Adj. R squared = 0.7404
Observed
Model
Weekly incidence
0e+00
1e−04
2e−04
3e−04
4e−04
5e−04
2014
2015
2016
Years

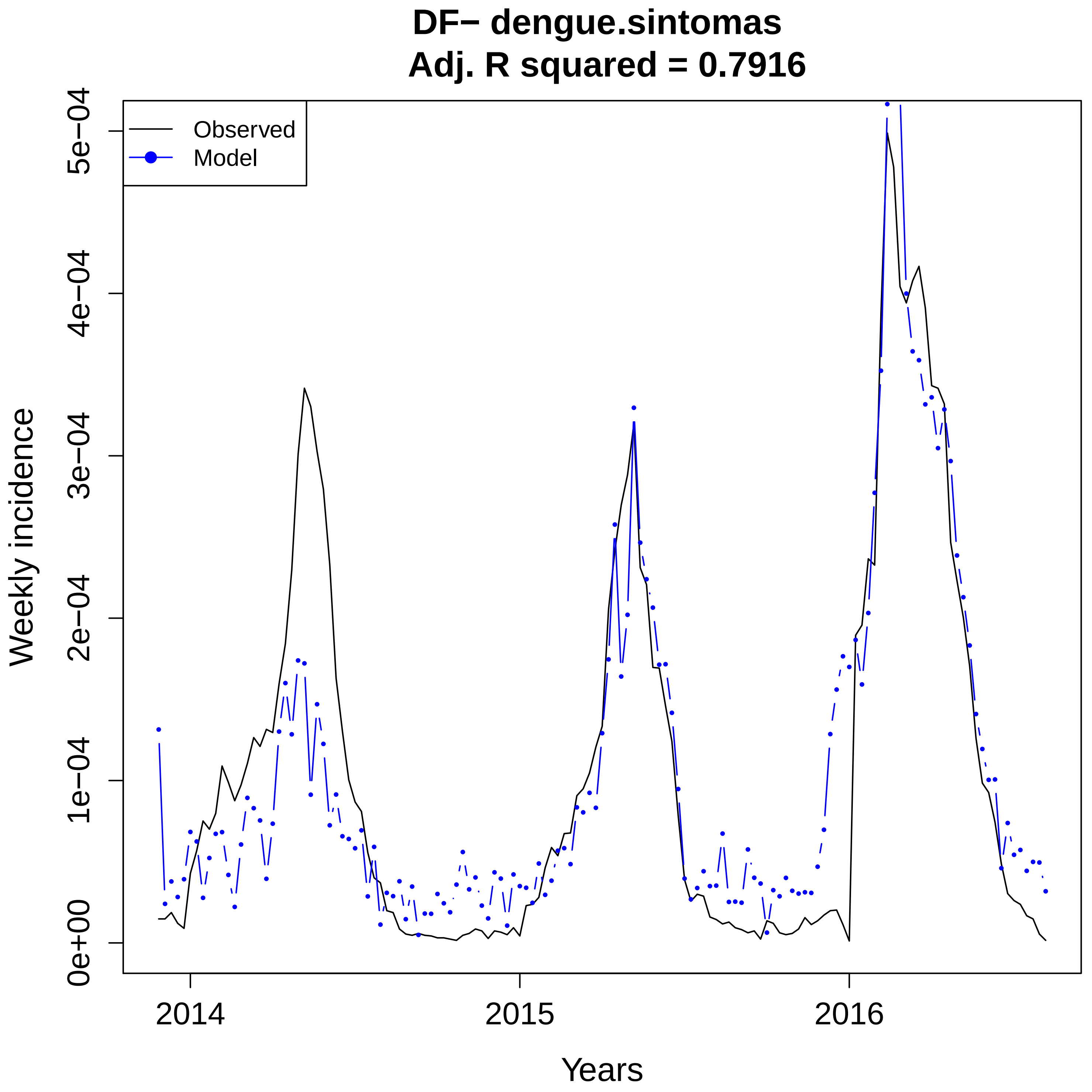
DF− dengue.sintomas
Adj. R squared = 0.7916
Observed
Model
Weekly incidence
0e+00
1e−04
2e−04
3e−04
4e−04
5e−04
2014
2015
2016
Years

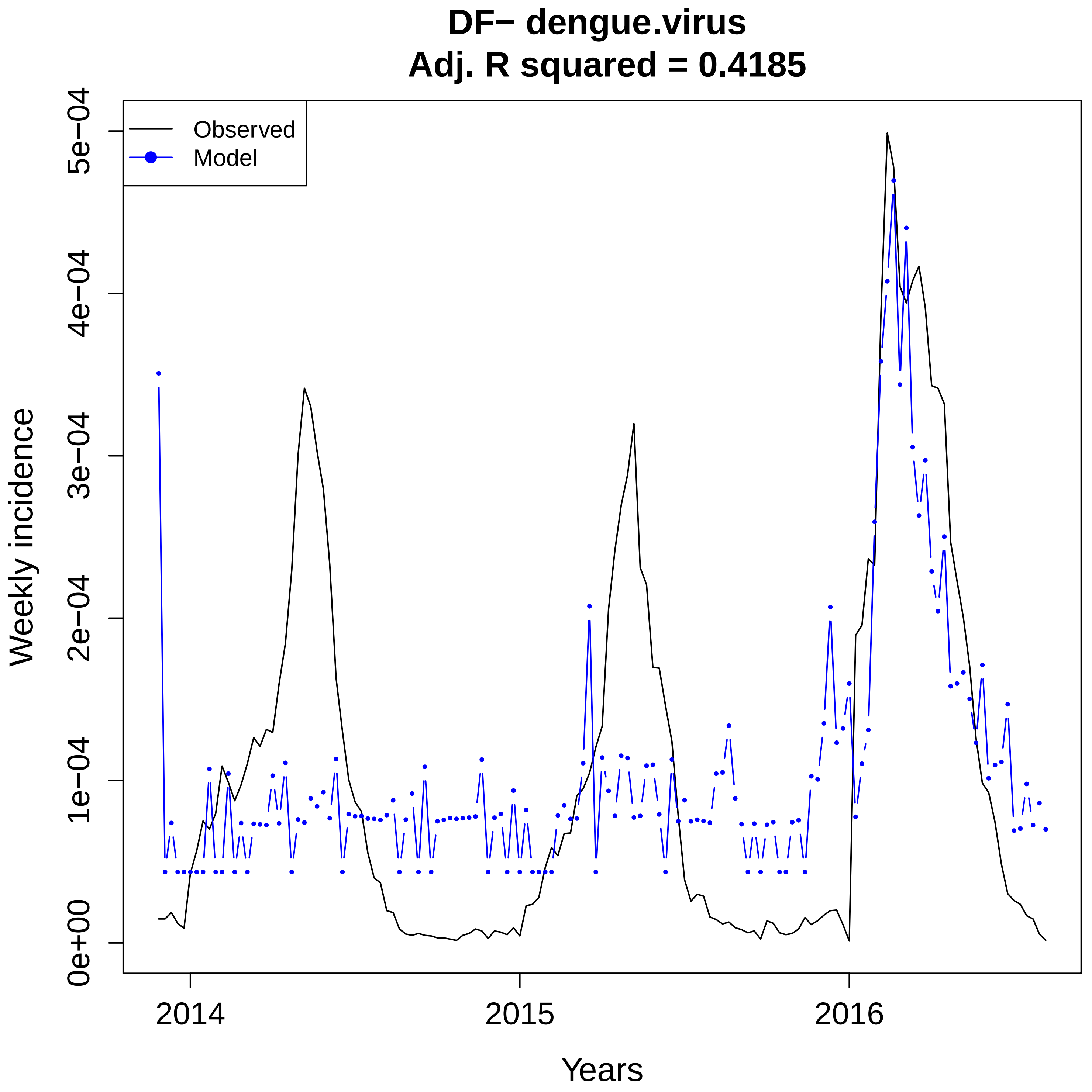
DF− dengue.virus
Adj. R squared = 0.4185
Observed
Model
Weekly incidence
0e+00
1e−04
2e−04
3e−04
4e−04
5e−04
2014
2015
2016
Years

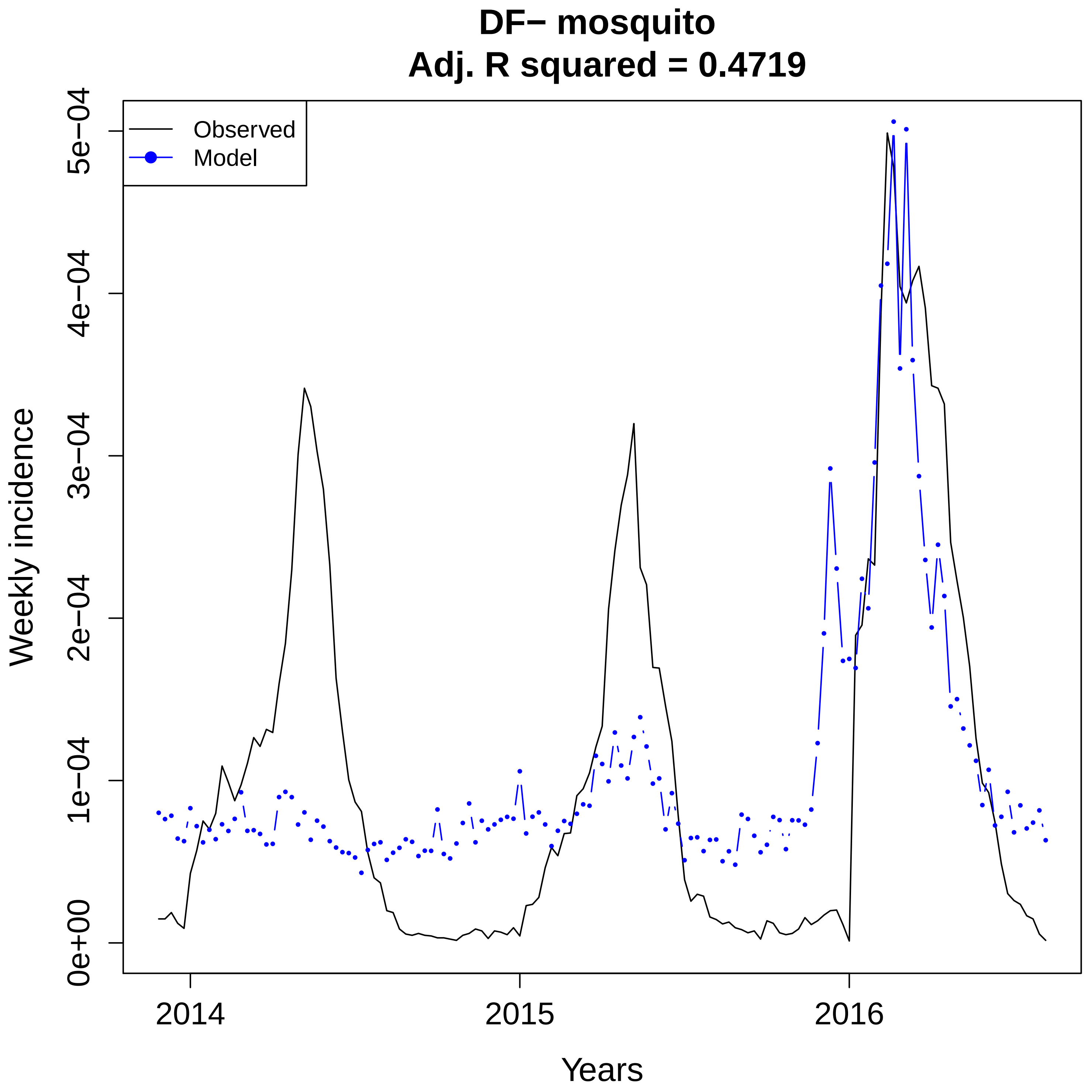

DF− mosquito
Adj. R squared = 0.4719
Observed
Model
Weekly incidence
0e+00
1e−04
2e−04
3e−04
4e−04
5e−04
2014
2015
2016
Years

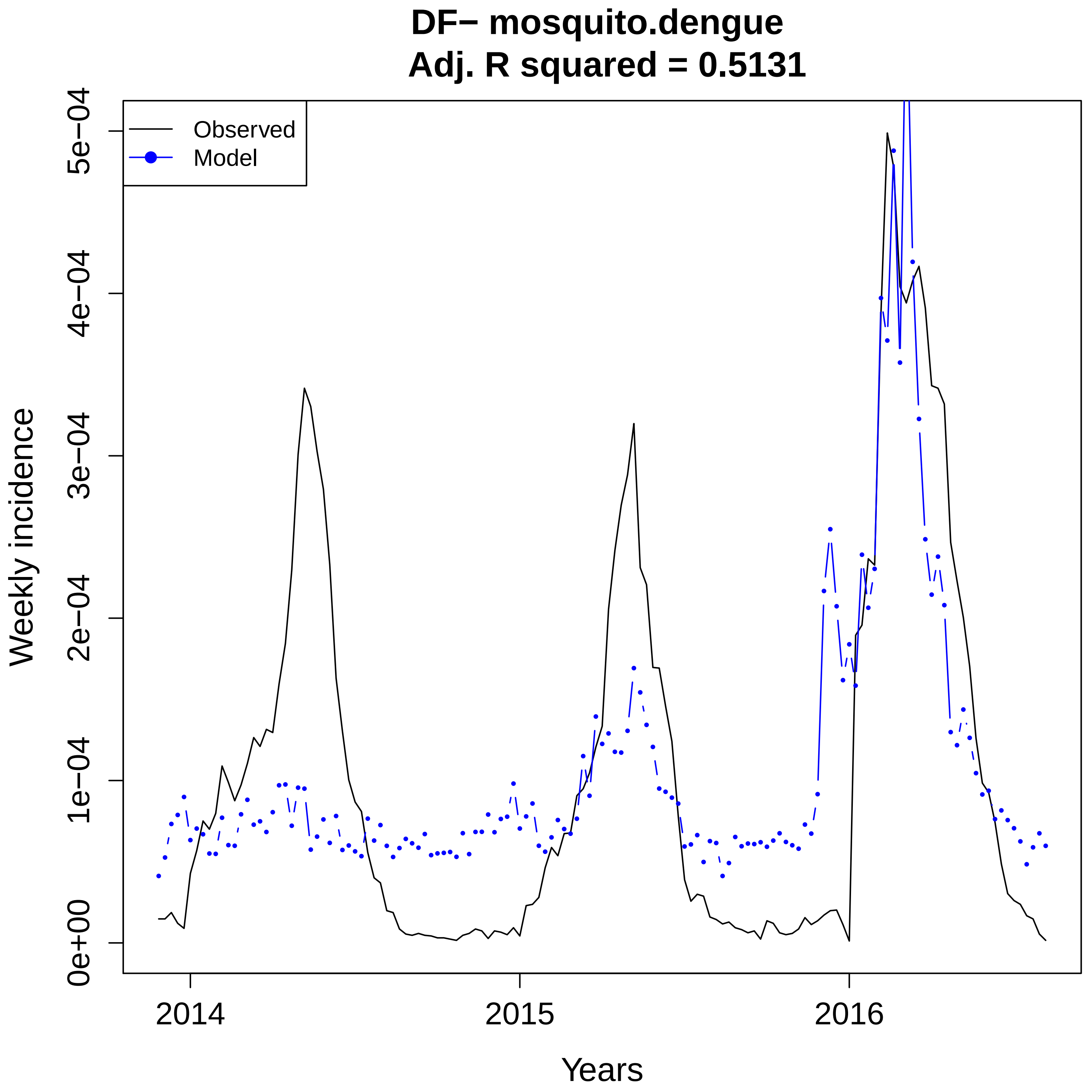

DF− mosquito.dengue
Adj. R squared = 0.5131
Observed
Model
Weekly incidence
0e+00
1e−04
2e−04
3e−04
4e−04
5e−04
2014
2015
2016
Years

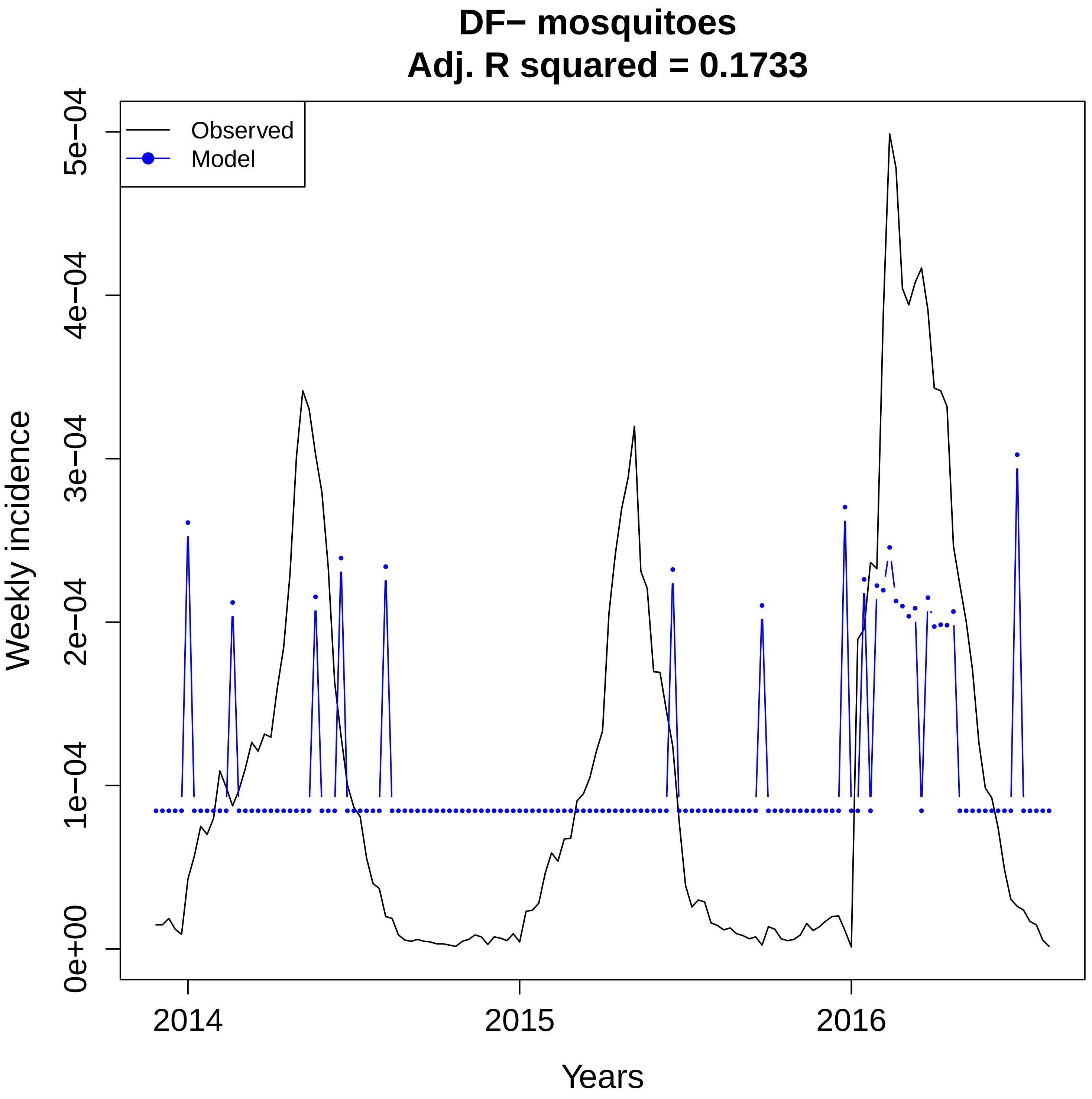

DF− mosquitoes
Adj. R squared = 0.1733
Observed
Model
Weekly incidence
0e+00
1e−04
2e−04
3e−04
4e−04
5e−04
2014
2015
2016
Years

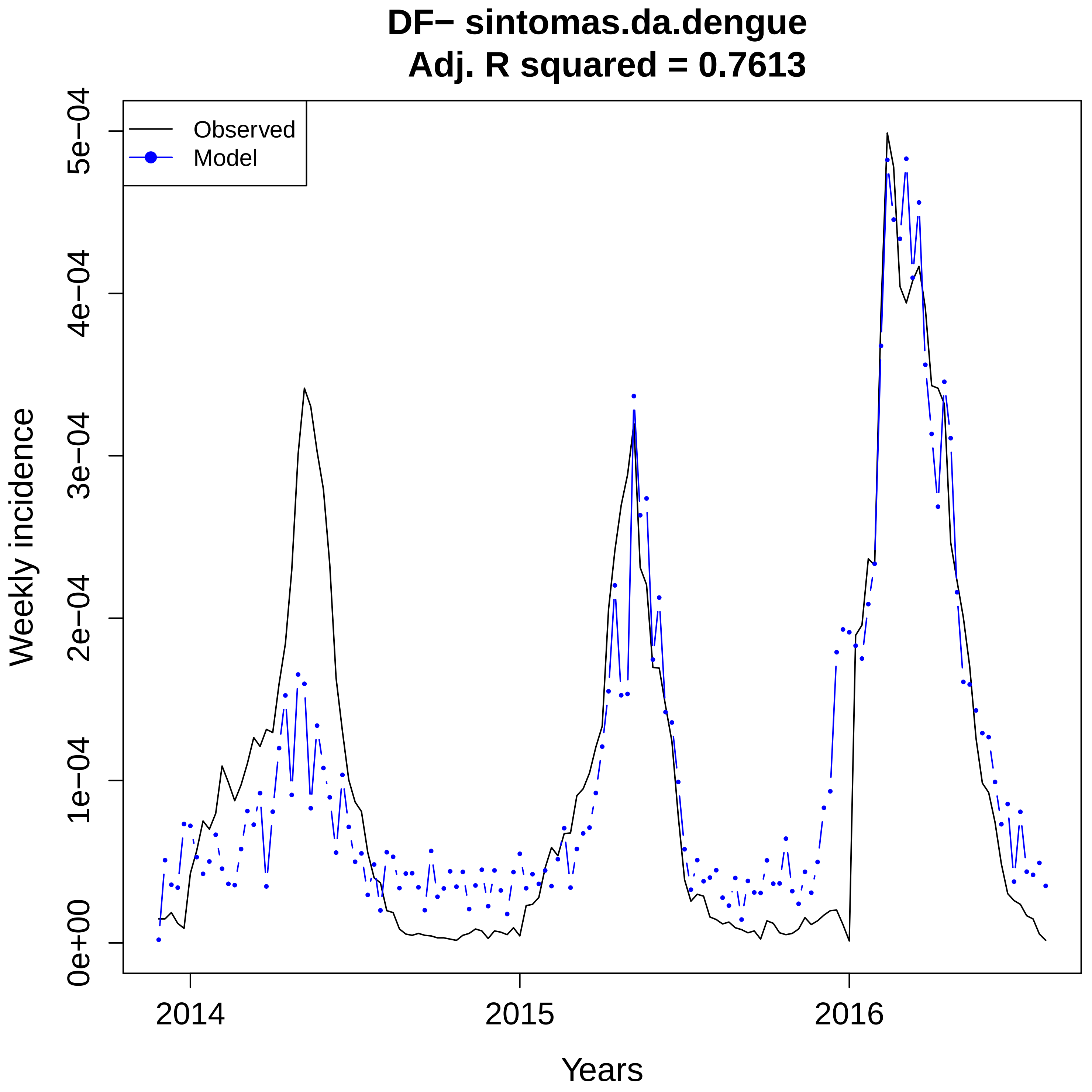
DF− sintomas.da.dengue
Adj. R squared = 0.7613
Observed
Model
Weekly incidence
0e+00
1e−04
2e−04
3e−04
4e−04
5e−04
2014
2015
2016
Years

Espírito Santo (ES)
Dengue incidence vs. Google Health Trends (2011–2016)

Definitions:

- **Combined terms:**
  - **All:** model with all the available terms. n= number of terms
  - **Four:** model with "dengue", "dengue sintomas", "aedes", and "mosquito".
  - **Uncorrelated:** model with non-correlated terms. n = number of uncorrelated terms
- **Individual terms:** Plots developed with one term at a time.

- Terms for all and uncorrelated models can be found in additional file 3.

Combined terms
Adjusted R squared

Espírito Santo (ES)

All
(n = 10)

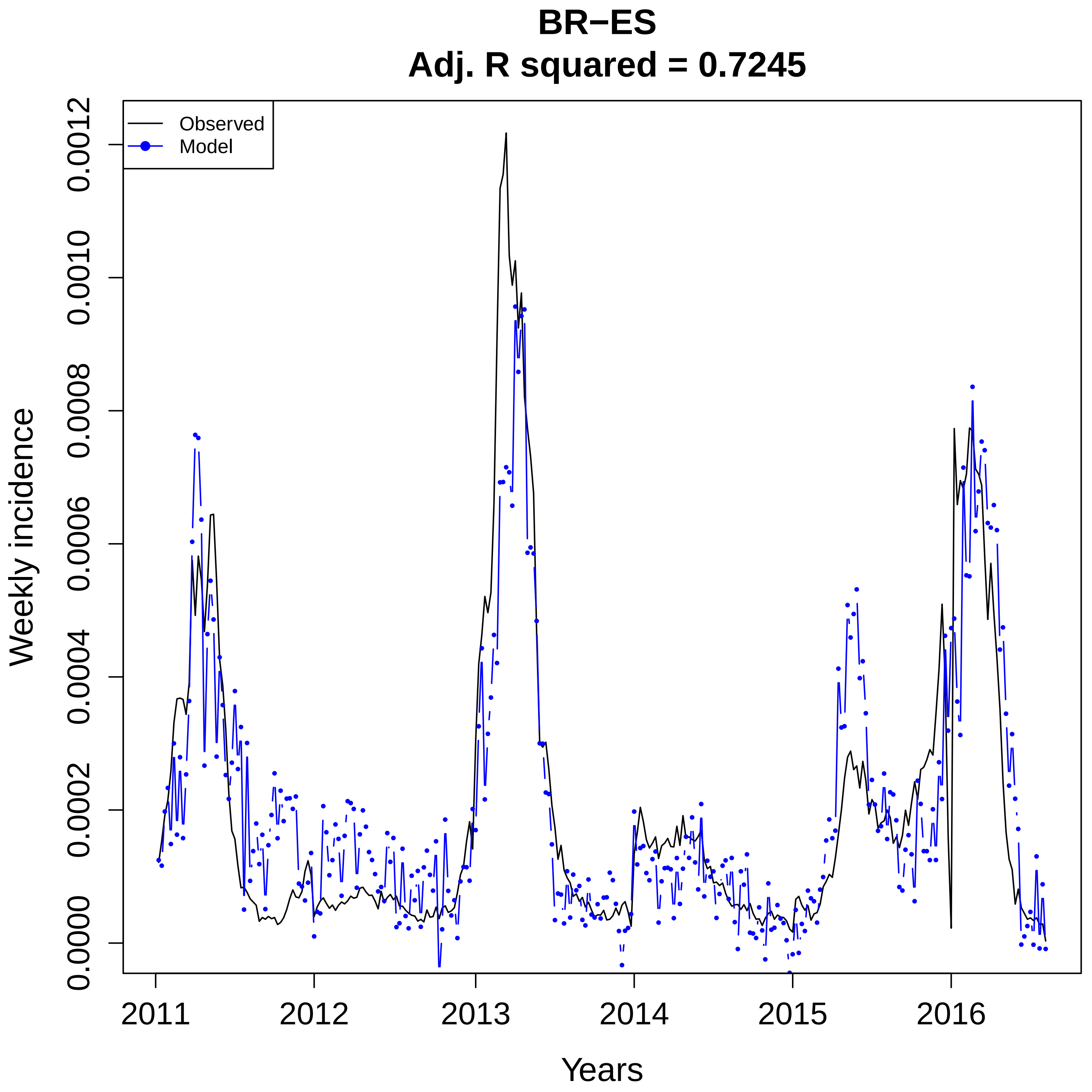

BR−ES
Adj. R squared = 0.7245
Observed
Model
Weekly incidence
0.0000
0.0002
0.0004
0.0006
0.0008
0.0010
0.0012
2011
2012
2013
2014
2015
2016
Years

Four

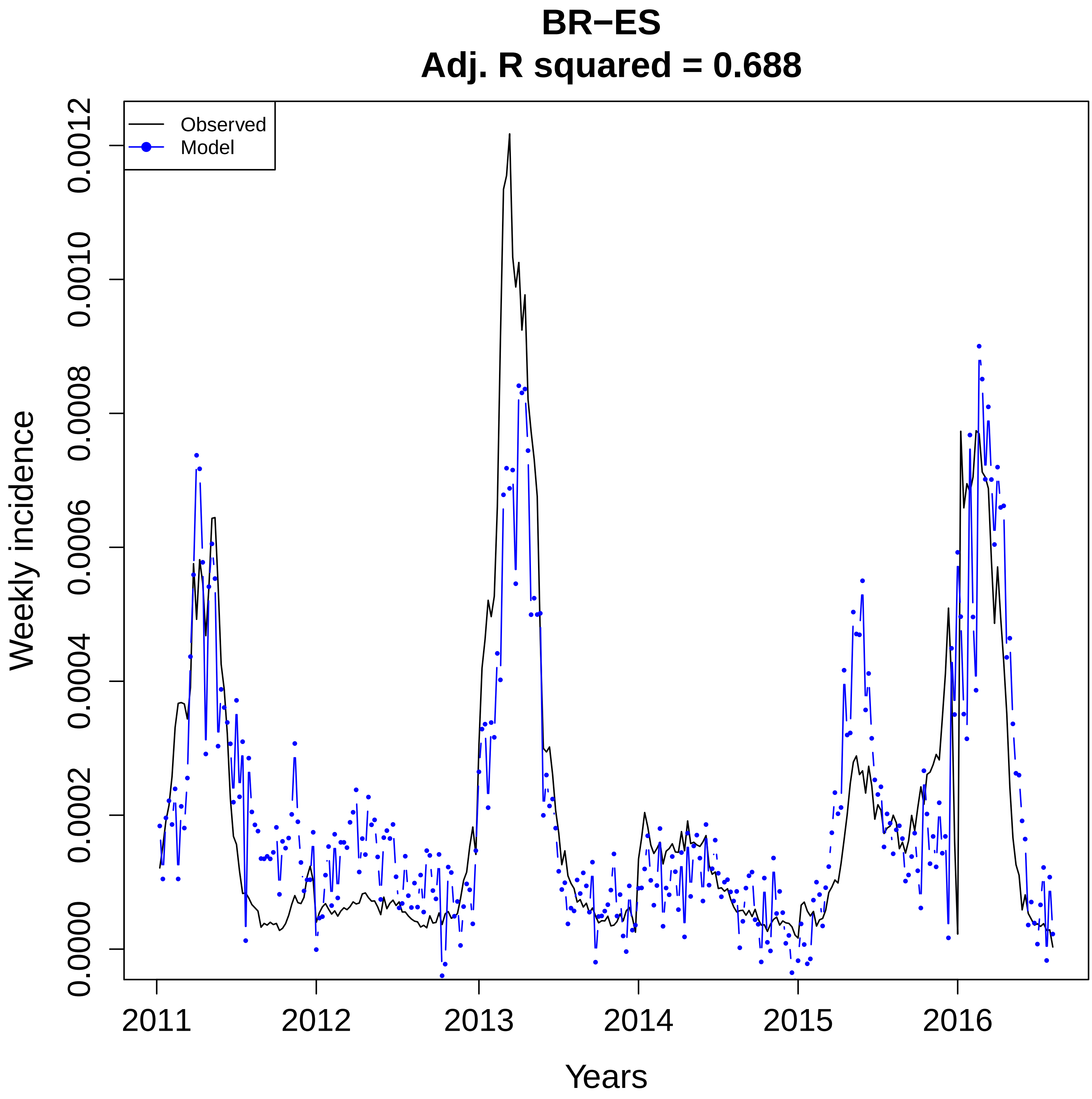

BR−ES
Adj. R squared = 0.688
Observed
Model
Weekly incidence
0.0000
0.0002
0.0004
0.0006
0.0008
0.0010
0.0012
2011
2012
2013
2014
2015
2016
Years

Uncorrelated
(n = 3)

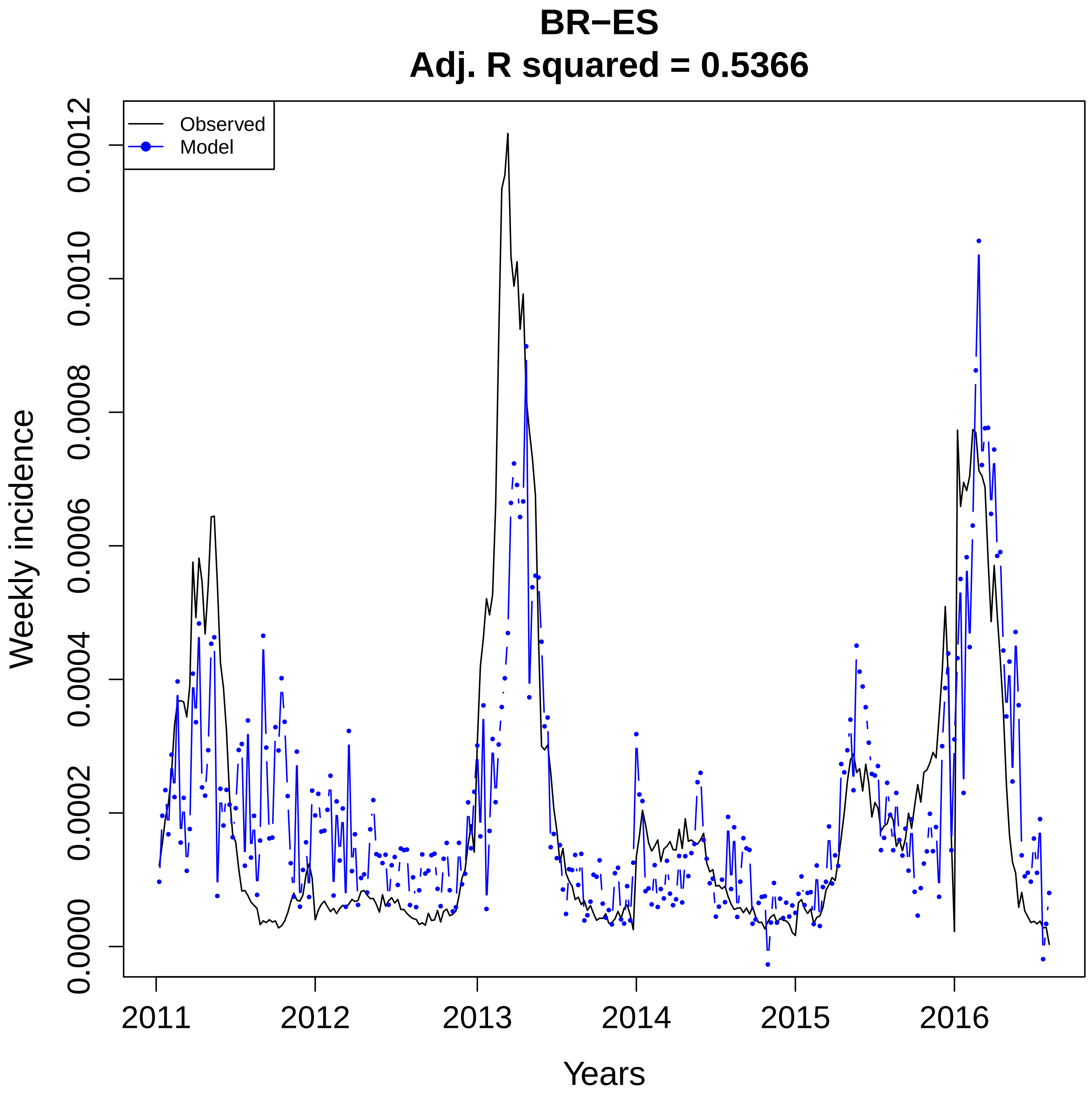

Individual terms
Adjusted R squared

Espírito Santo (ES)

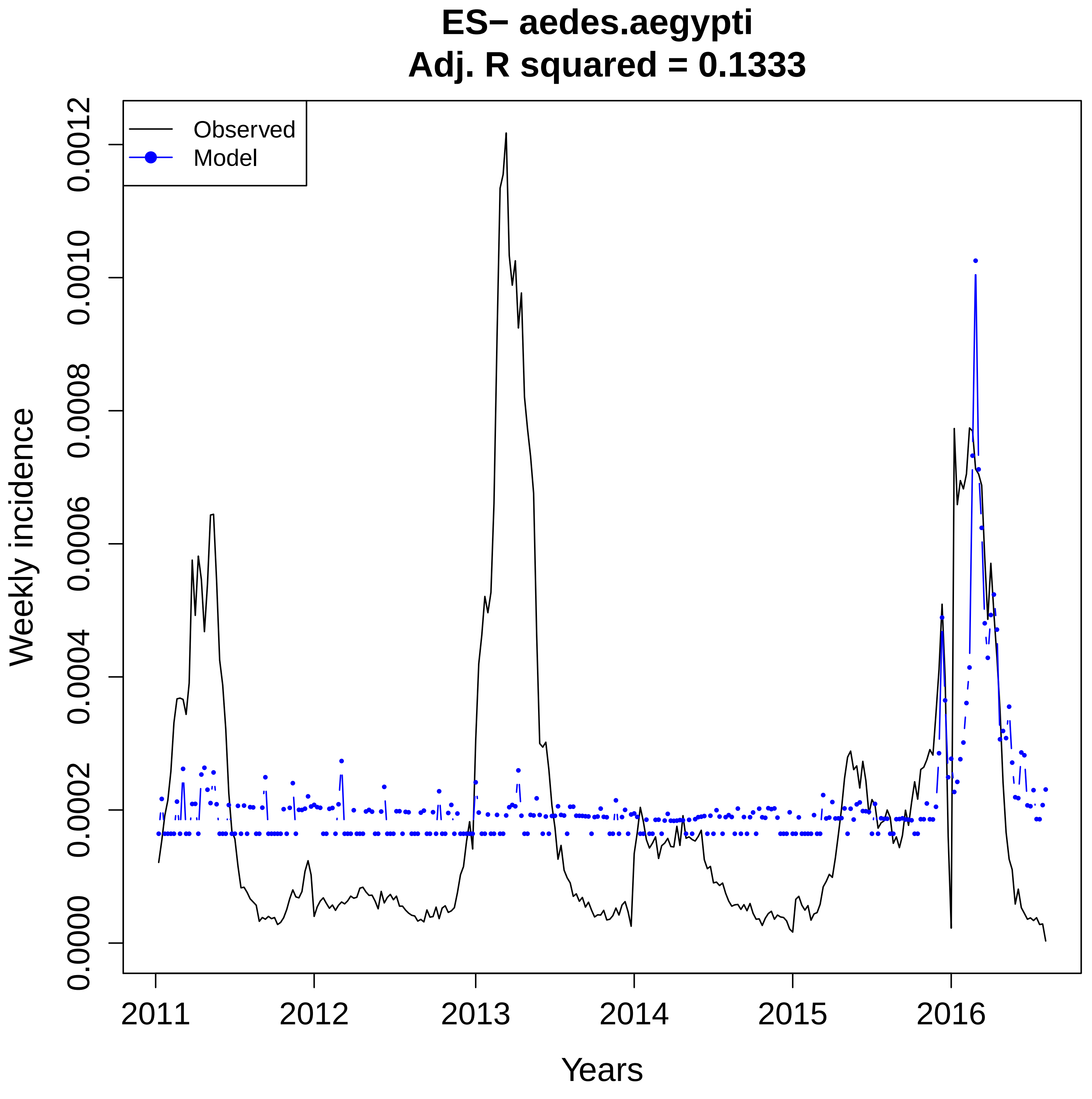
ES− aedes.aegypti
Adj. R squared = 0.1333
Observed
Model
Weekly incidence
0.0000
0.0002
0.0004
0.0006
0.0008
0.0010
0.0012
2011
2012
2013
2014
2015
2016
Years

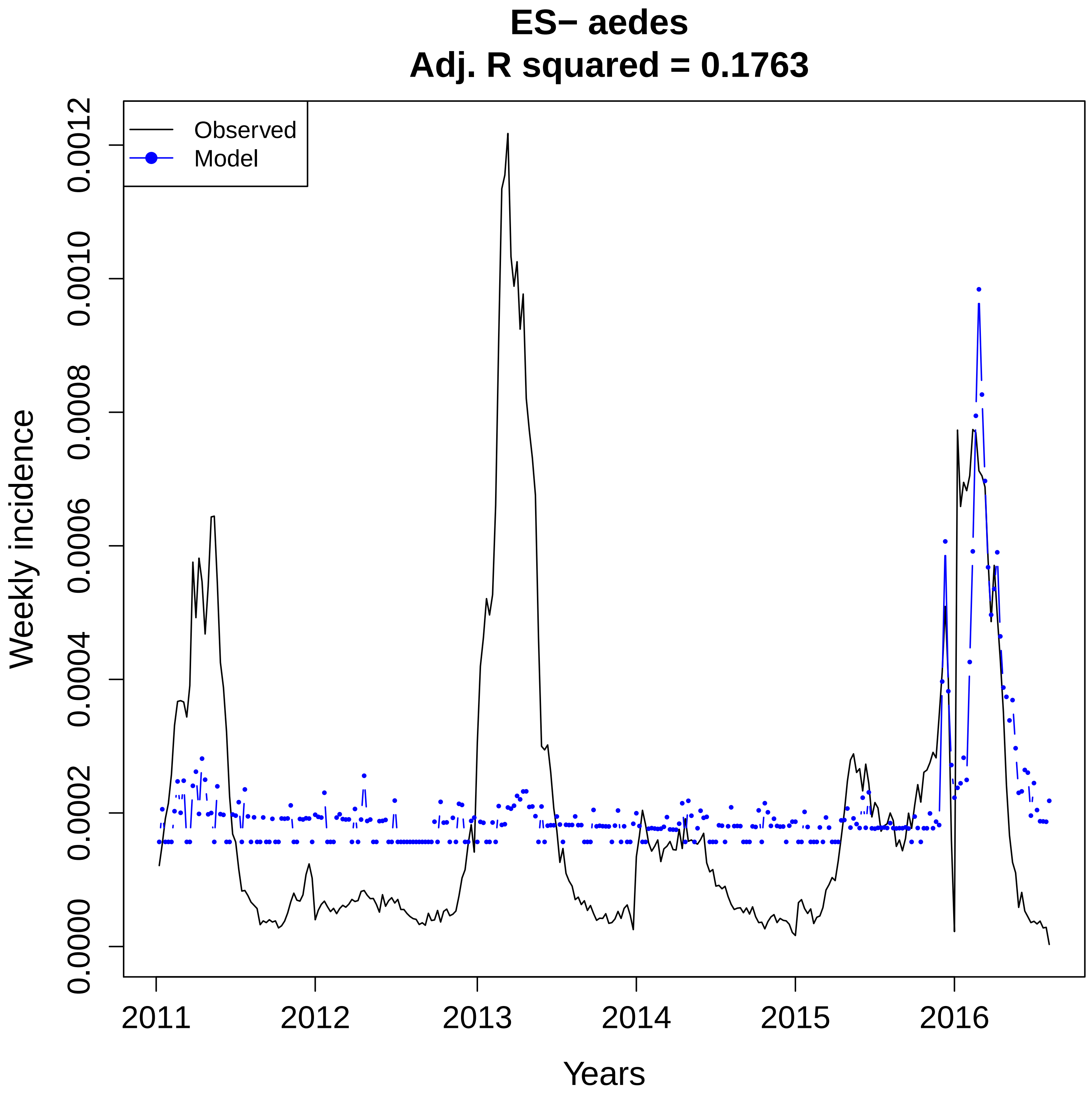

ES− aedes
Adj. R squared = 0.1763
Observed
Model
Weekly incidence
0.0000
0.0002
0.0004
0.0006
0.0008
0.0010
0.0012
2011
2012
2013
2014
2015
2016
Years

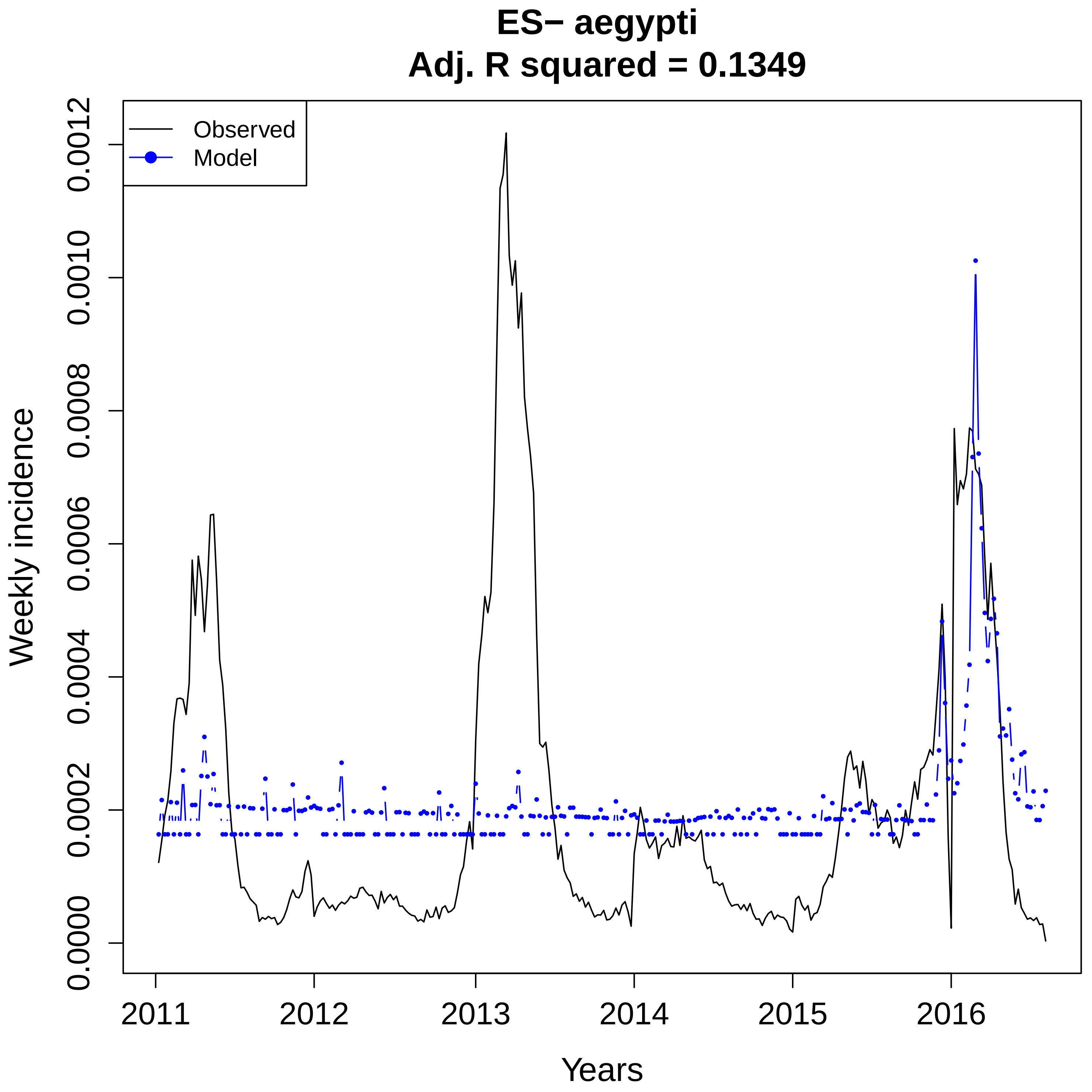
ES− aegypti
Adj. R squared = 0.1349
Observed
Model
Weekly incidence
0.0000
0.0002
0.0004
0.0006
0.0008
0.0010
0.0012
2011
2012
2013
2014
2015
2016
Years

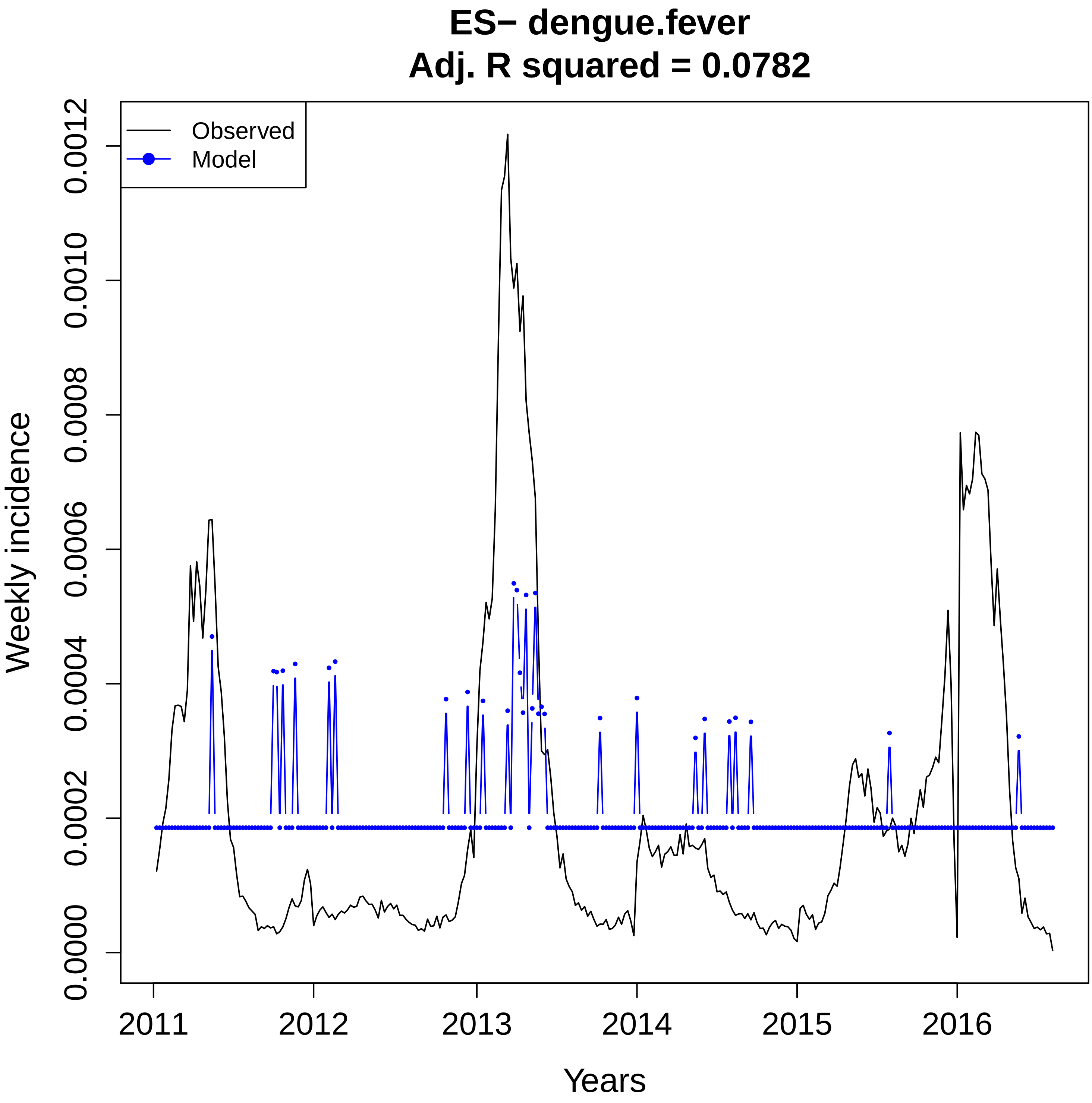
ES− dengue.fever
Adj. R squared = 0.0782
Observed
Model
Weekly incidence
0.0000
0.0002
0.0004
0.0006
0.0008
0.0010
0.0012
2011
2012
2013
2014
2015
2016
Years

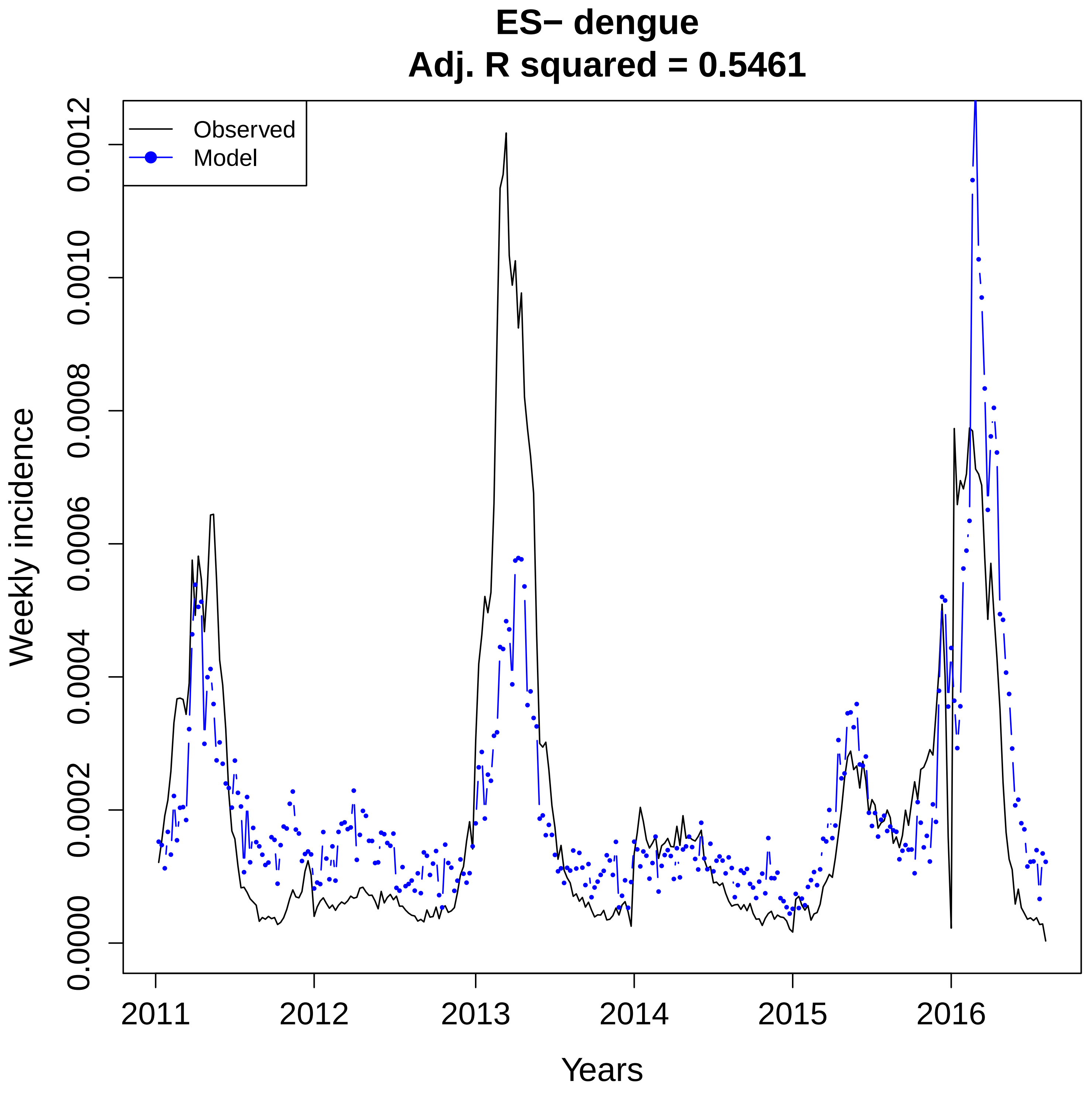
ES− dengue
Adj. R squared = 0.5461
Observed
Model
Weekly incidence
0.0000
0.0002
0.0004
0.0006
0.0008
0.0010
0.0012
2011
2012
2013
2014
2015
2016
Years

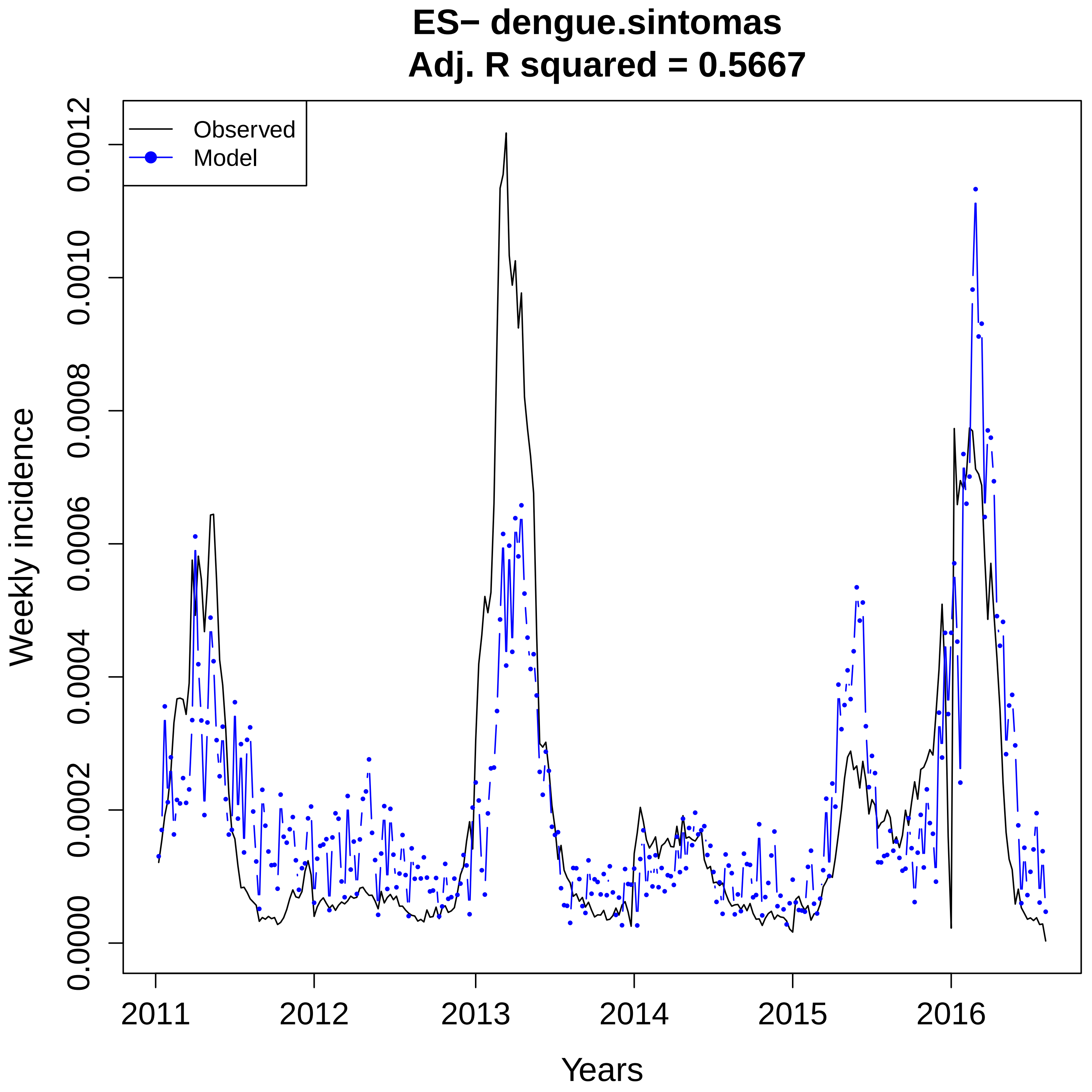
ES− dengue.sintomas
Adj. R squared = 0.5667
Observed
Model
Weekly incidence
0.0000
0.0002
0.0004
0.0006
0.0008
0.0010
0.0012
2011
2012
2013
2014
2015
2016
Years

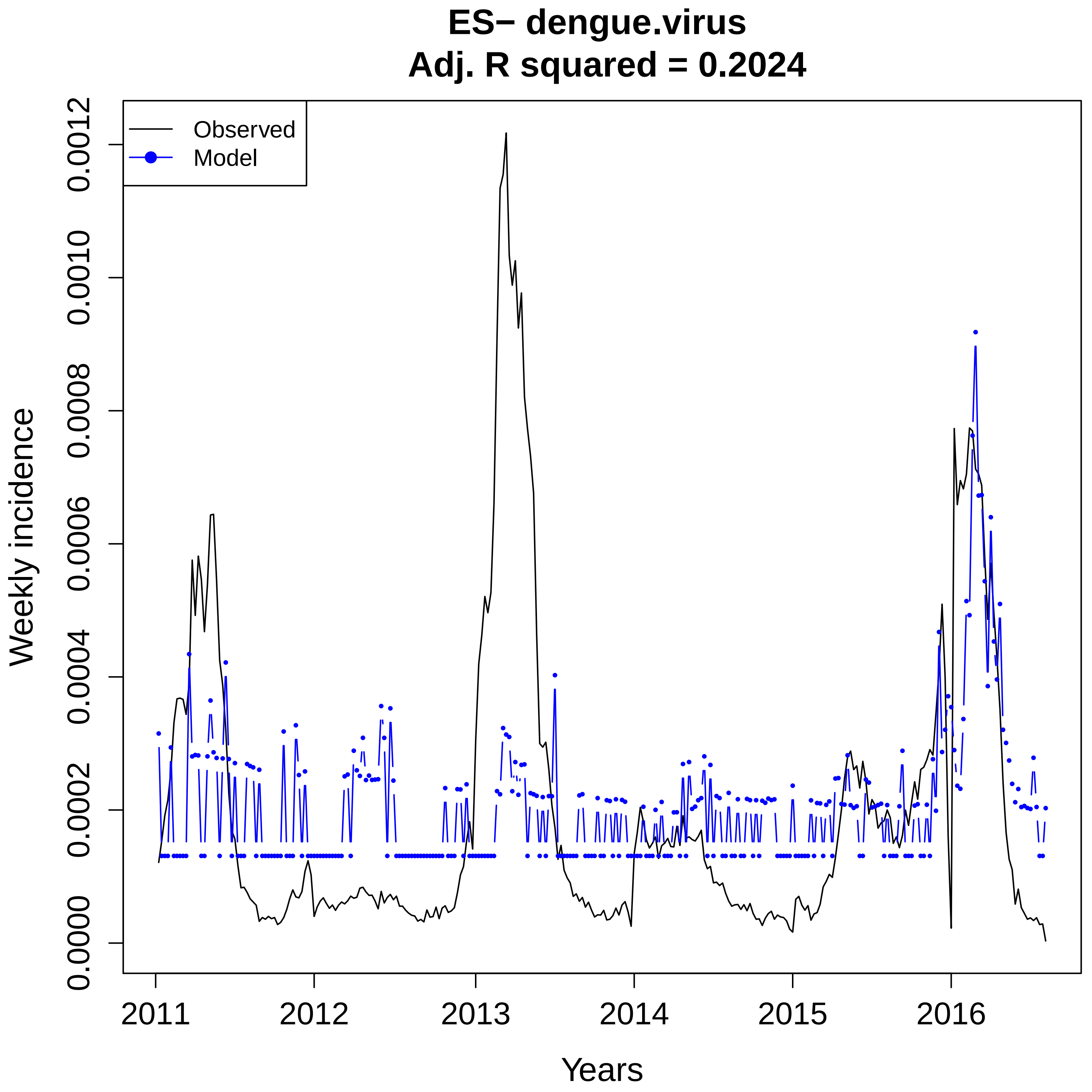
ES− dengue.virus
Adj. R squared = 0.2024
Observed
Model
Weekly incidence
0.0000
0.0002
0.0004
0.0006
0.0008
0.0010
0.0012
2011
2012
2013
2014
2015
2016
Years

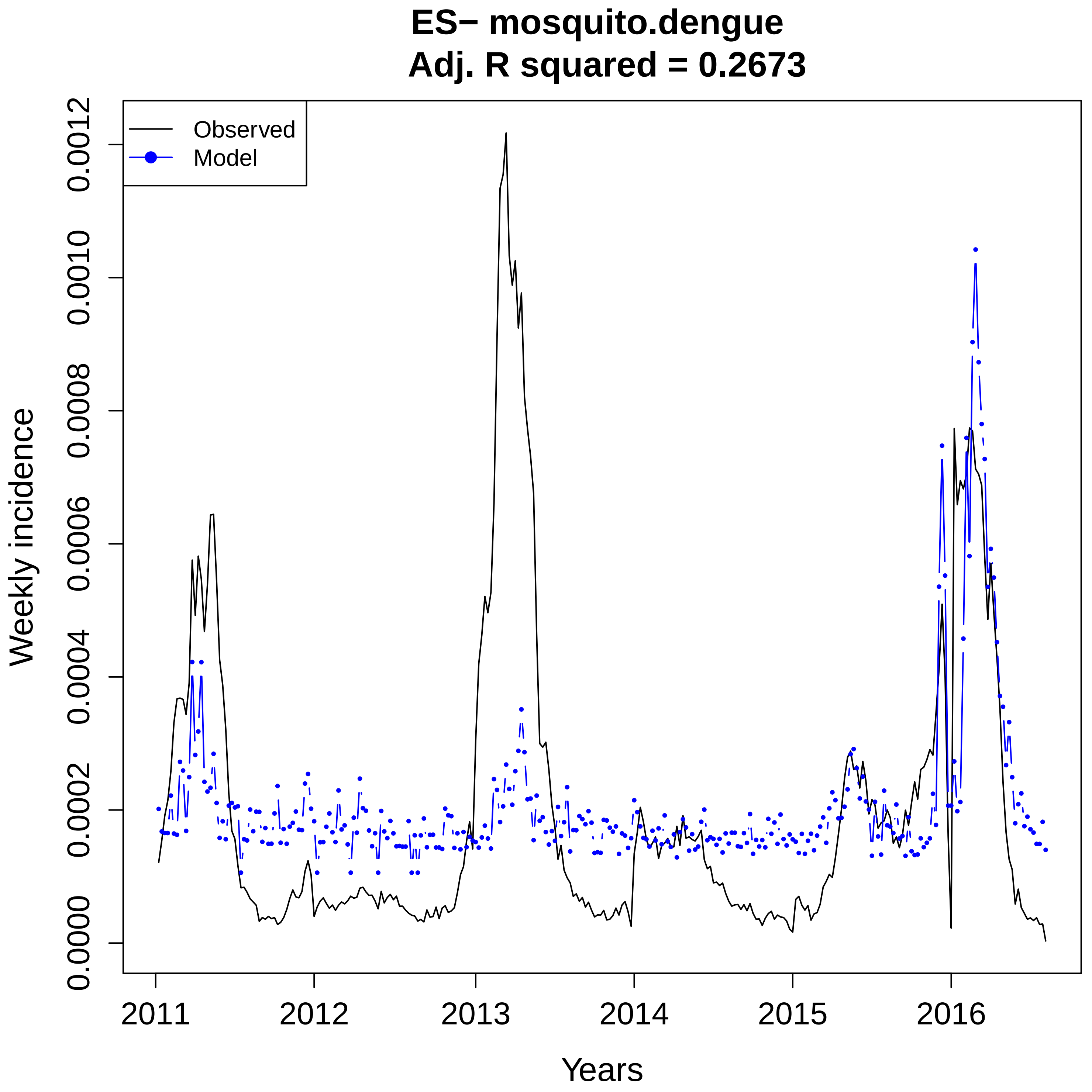
ES− mosquito.dengue
Adj. R squared = 0.2673
Observed
Model
Weekly incidence
0.0000
0.0002
0.0004
0.0006
0.0008
0.0010
0.0012
2011
2012
2013
2014
2015
2016
Years

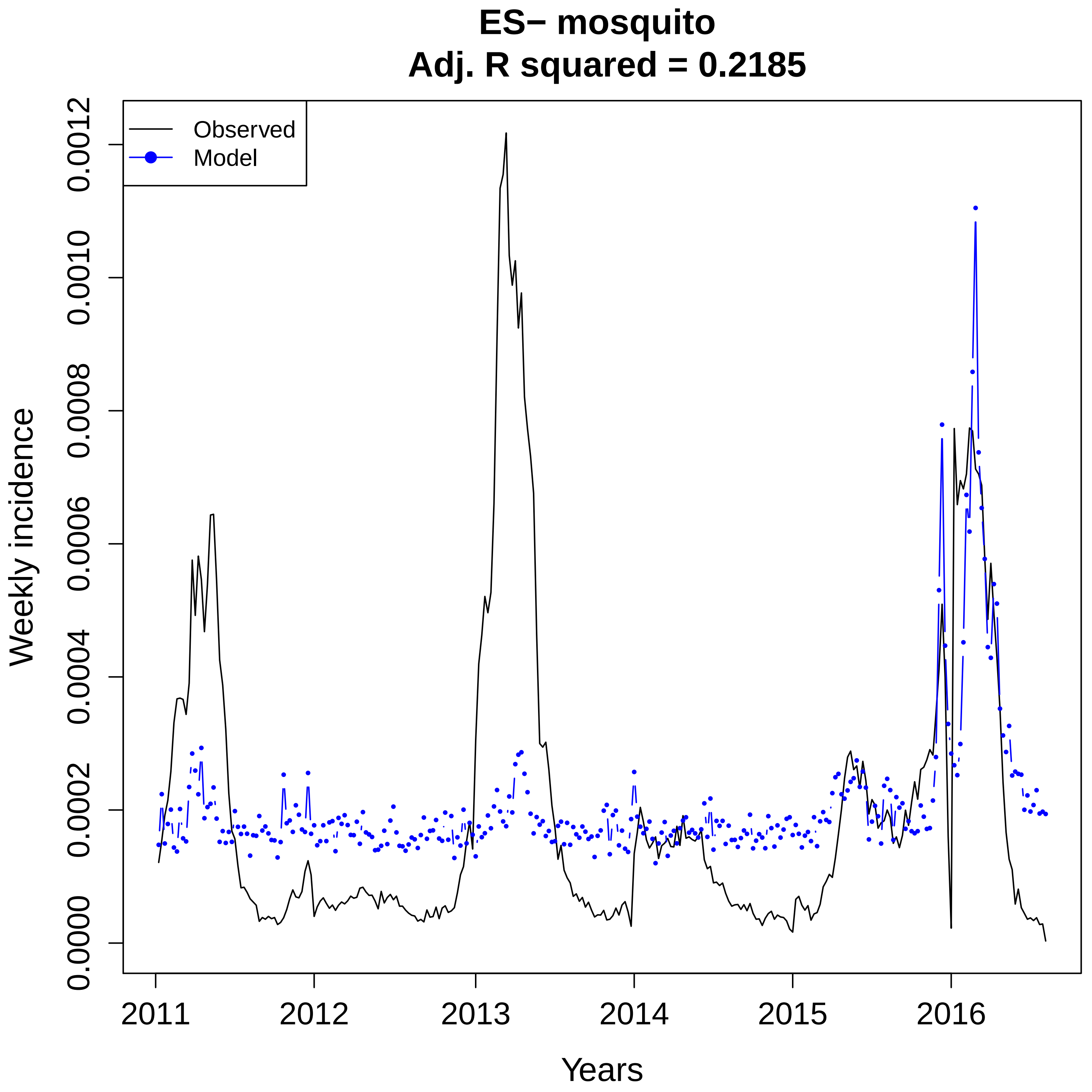
ES− mosquito
Adj. R squared = 0.2185
Observed
Model
Weekly incidence
0.0000
0.0002
0.0004
0.0006
0.0008
0.0010
0.0012
2011
2012
2013
2014
2015
2016
Years

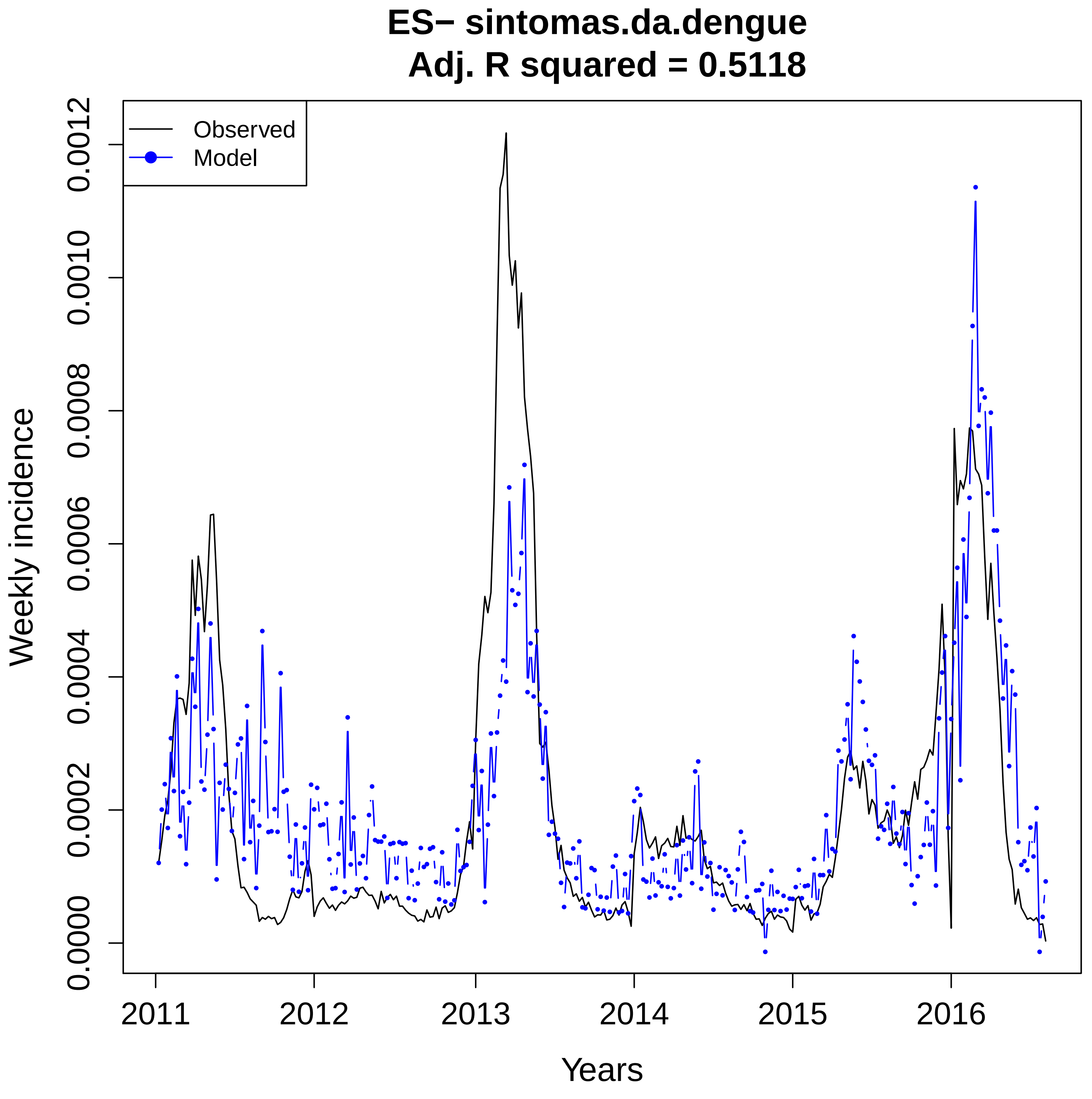

ES− sintomas.da.dengue
Adj. R squared = 0.5118
Observed
Model
Weekly incidence
0.0000
0.0002
0.0004
0.0006
0.0008
0.0010
0.0012
2011
2012
2013
2014
2015
2016
Years

Goiás (GO)

Dengue incidence vs. Google Health Trends (2011–2016)

Definitions:

- **Combined terms:**
    - **All:** model with all the available terms. n= number of terms
    - **Four:** model with "dengue", "dengue sintomas", "aedes", and "mosquito".
    - **Uncorrelated:** model with non-correlated terms. n = number of uncorrelated terms
- **Individual terms:** Plots developed with one term at a time.

- Terms for all and uncorrelated models can be found in additional file 3.

Combined terms
Adjusted R squared

Goiás (GO)

All
(n = 11)

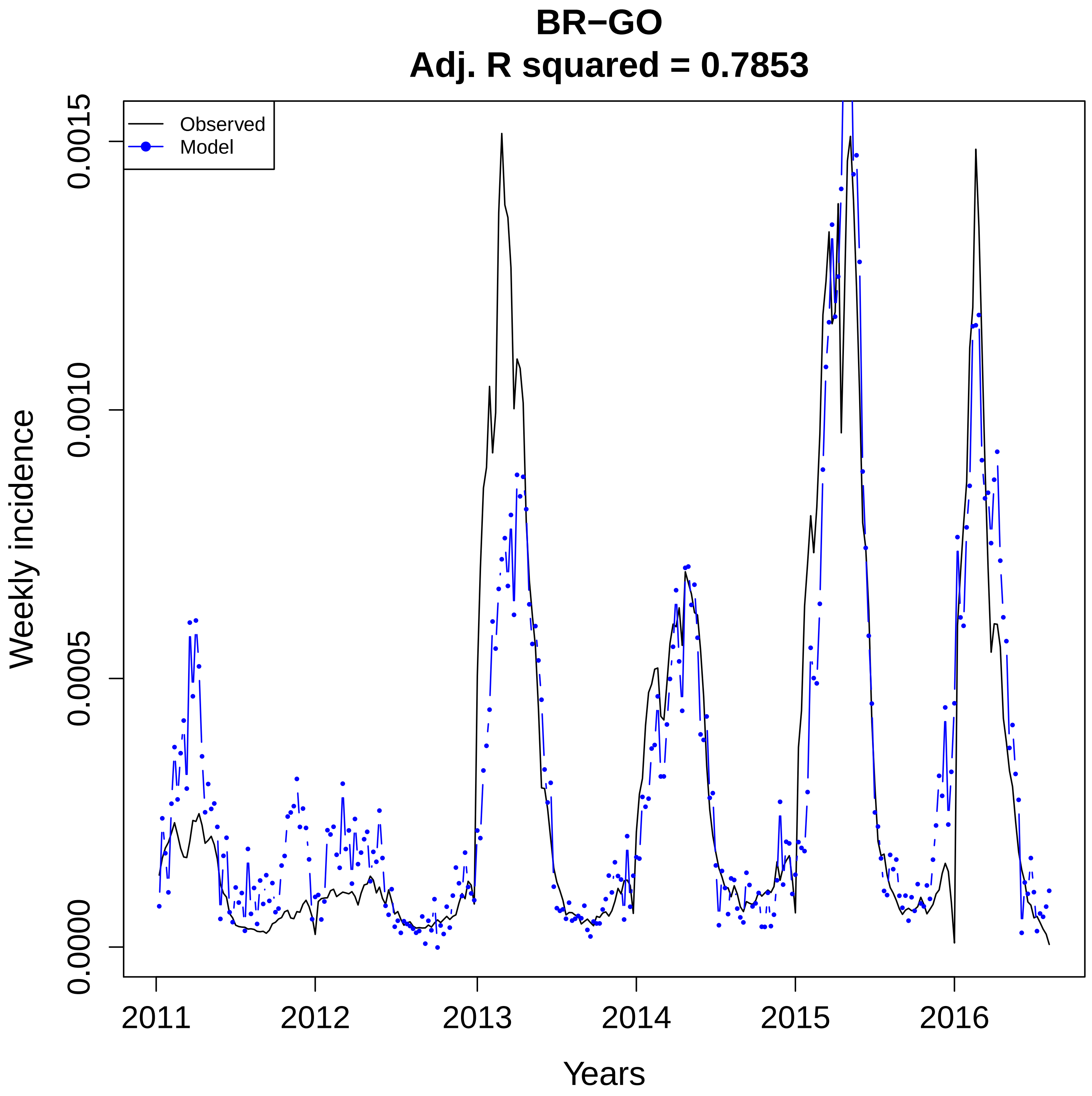

BR−GO
Adj. R squared = 0.7853
Observed
Model
Weekly incidence
0.0000
0.0005
0.0010
0.0015
2011
2012
2013
2014
2015
2016
Years

Four

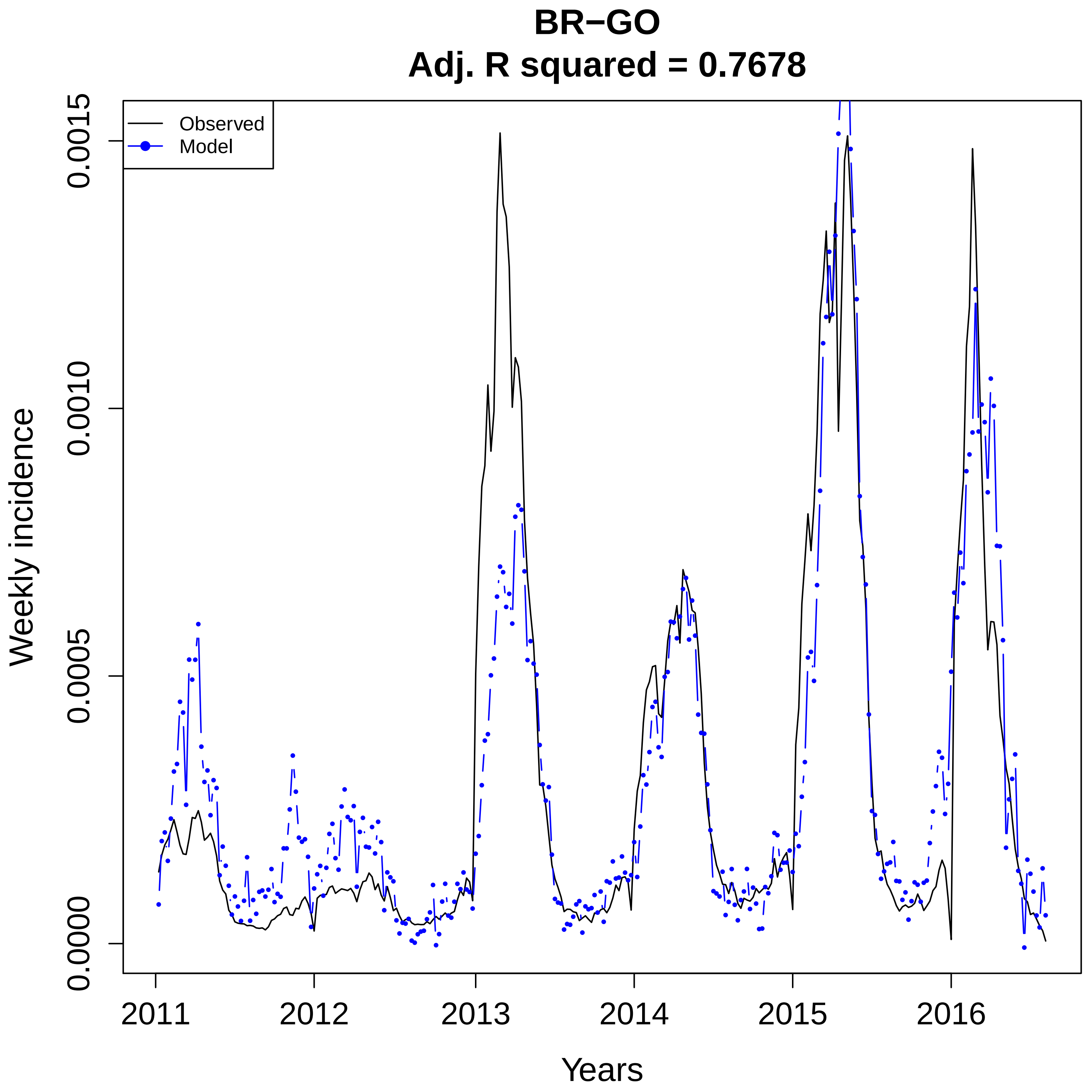

BR−GO
Adj. R squared = 0.7678
Observed
Model
Weekly incidence
0.0000
0.0005
0.0010
0.0015
2011
2012
2013
2014
2015
2016
Years

Uncorrelated
(n = 3)

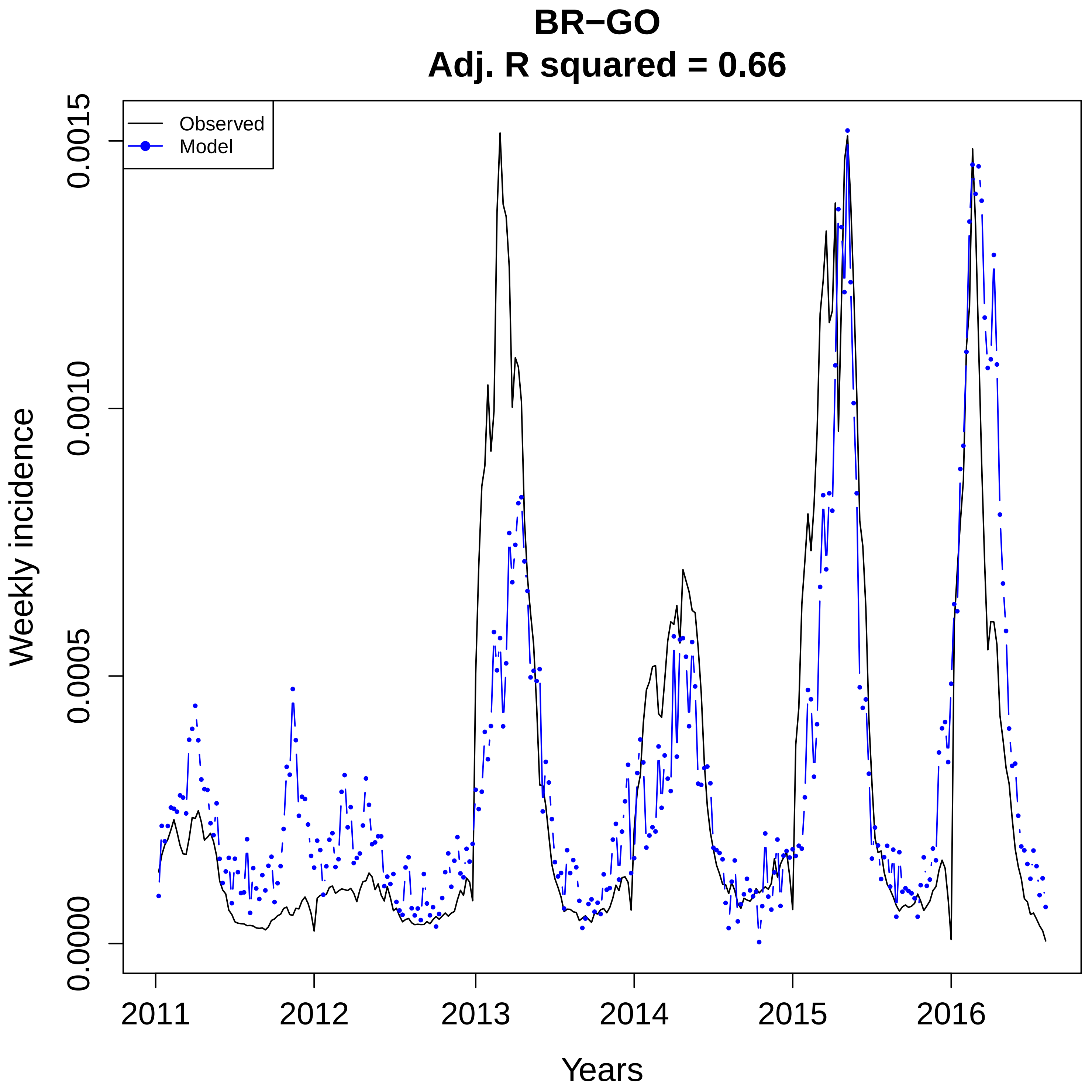

Individual terms
Adjusted R squared

Goiás (GO)

GO− aedes.aegypti
Adj. R squared = 0.129

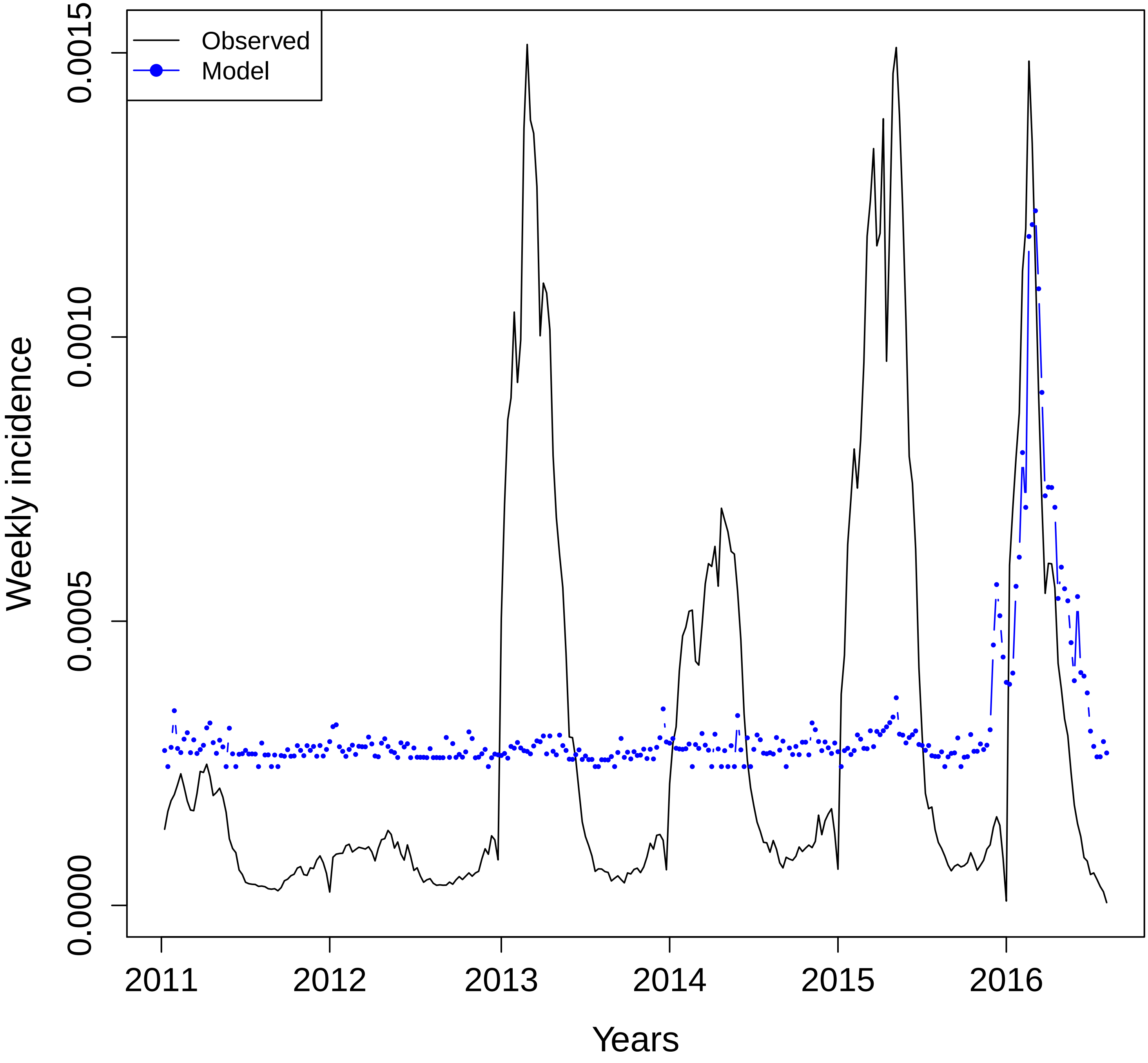

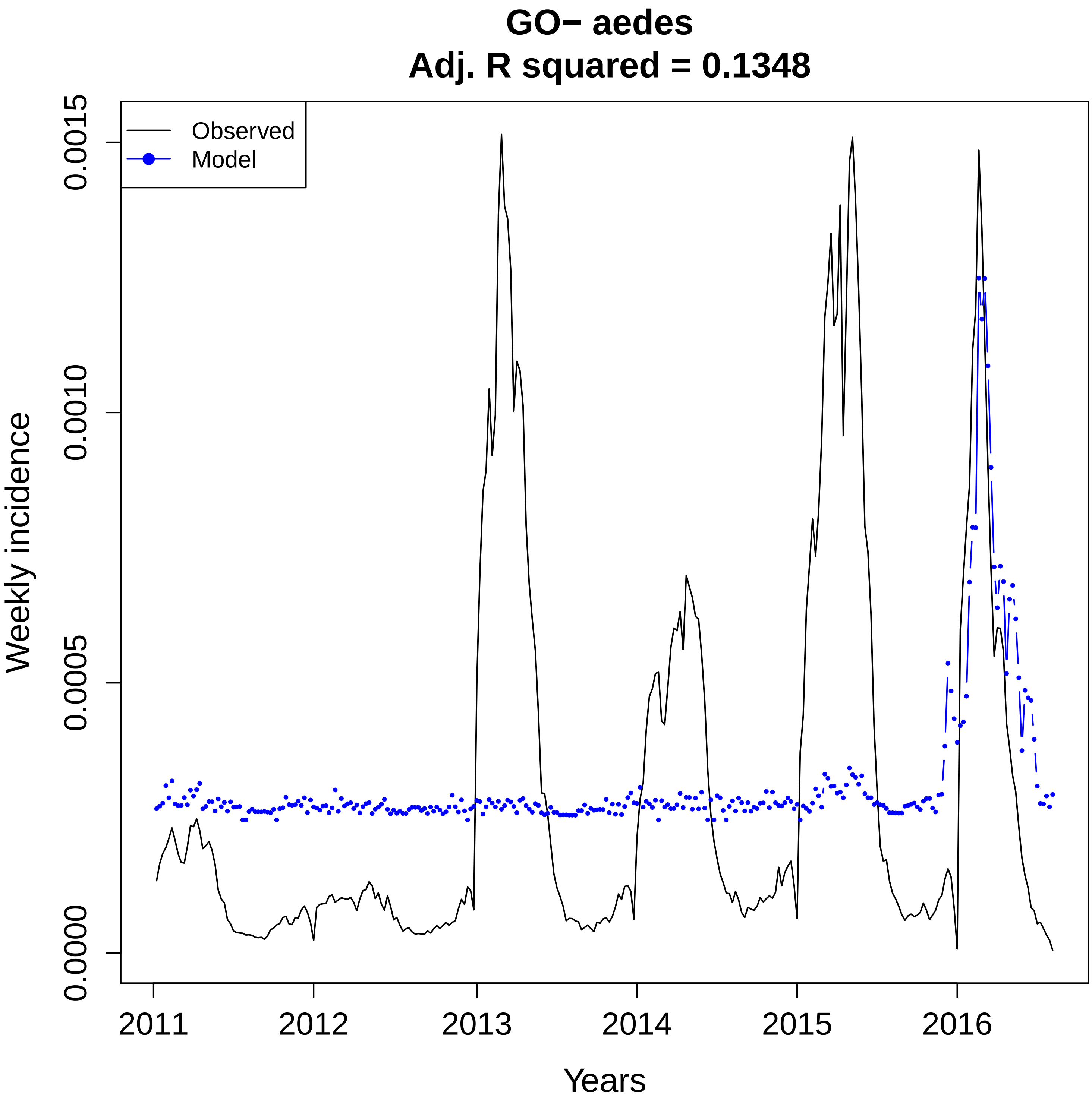
GO− aedes
Adj. R squared = 0.1348
Observed
Model
Weekly incidence
0.0000
0.0005
0.0010
0.0015
2011
2012
2013
2014
2015
2016
Years

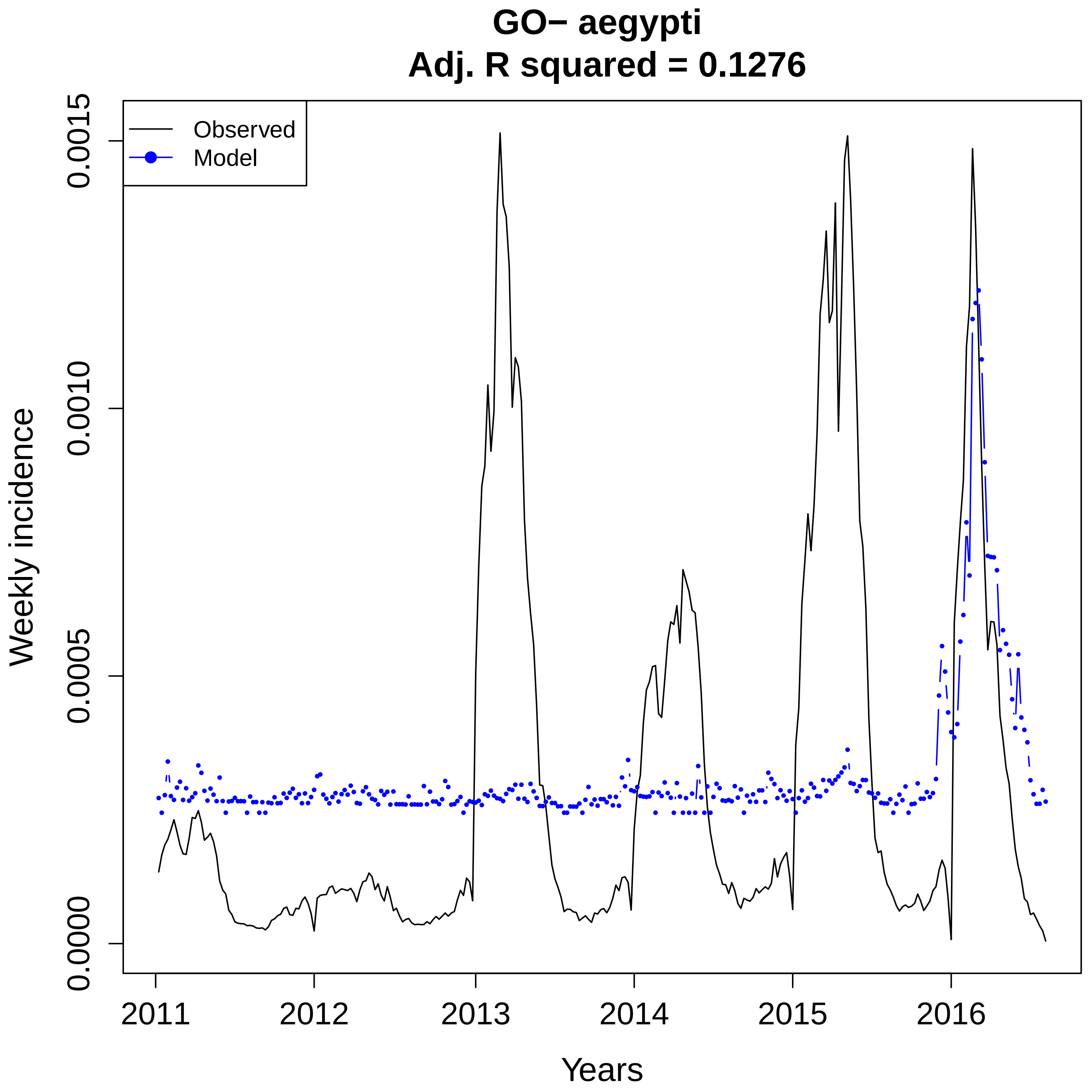
GO− aegypti
Adj. R squared = 0.1276
Observed
Model
Weekly incidence
0.0000
0.0005
0.0010
0.0015
Years
2011
2012
2013
2014
2015
2016

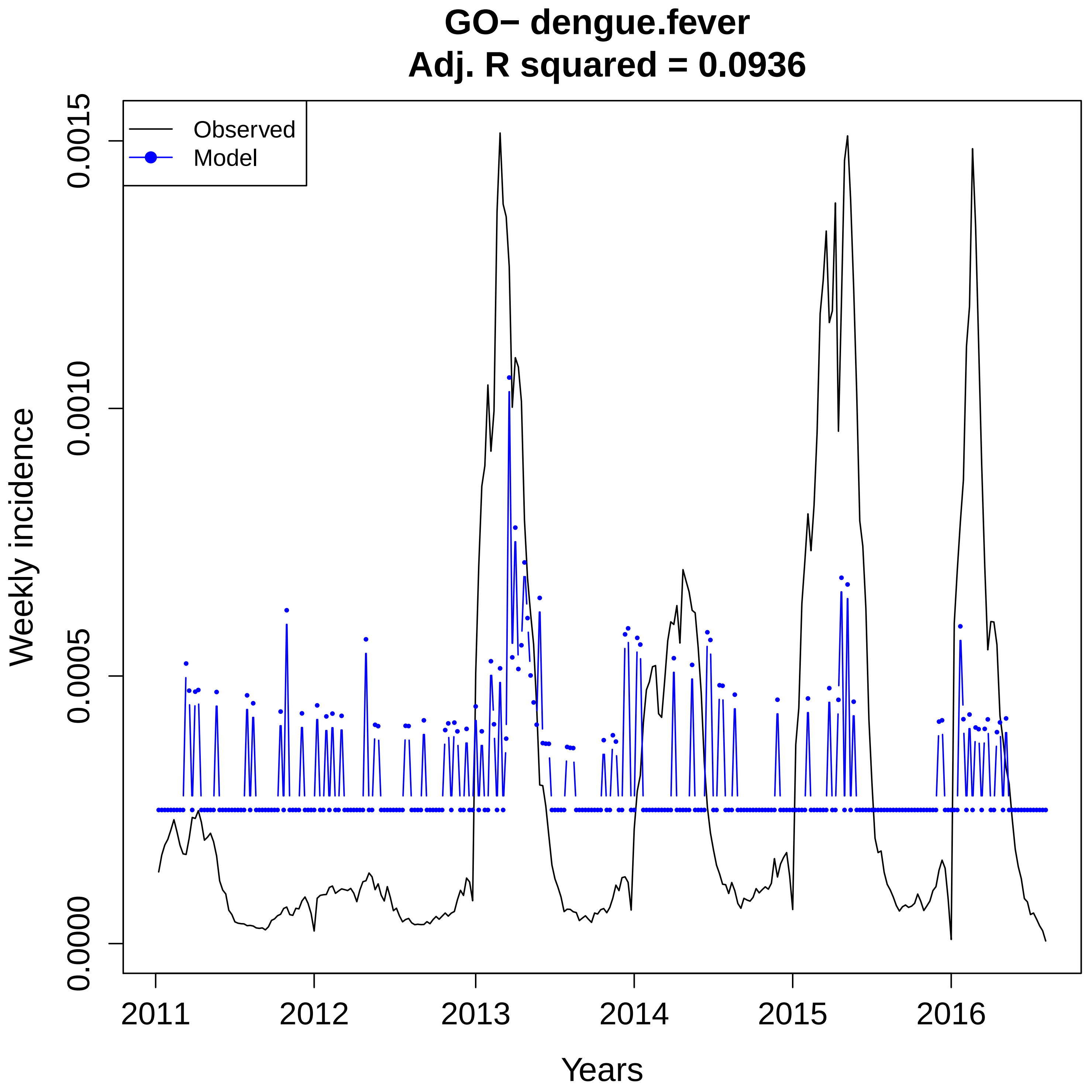

GO− dengue.fever
Adj. R squared = 0.0936
Observed
Model
Weekly incidence
0.0000
0.0005
0.0010
0.0015
2011
2012
2013
2014
2015
2016
Years

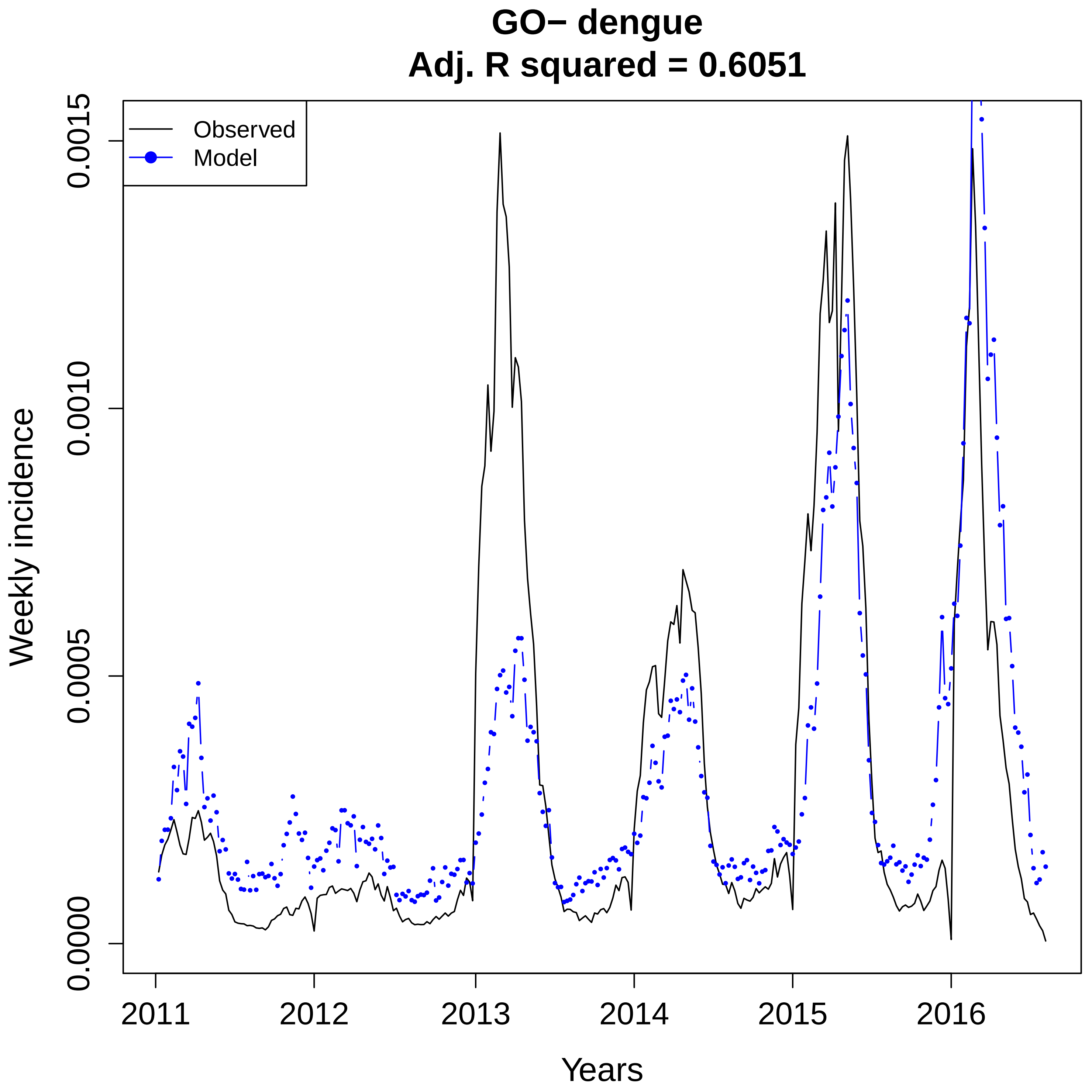

GO− dengue
Adj. R squared = 0.6051
Observed
Model
Weekly incidence
0.0000
0.0005
0.0010
0.0015
2011
2012
2013
2014
2015
2016
Years

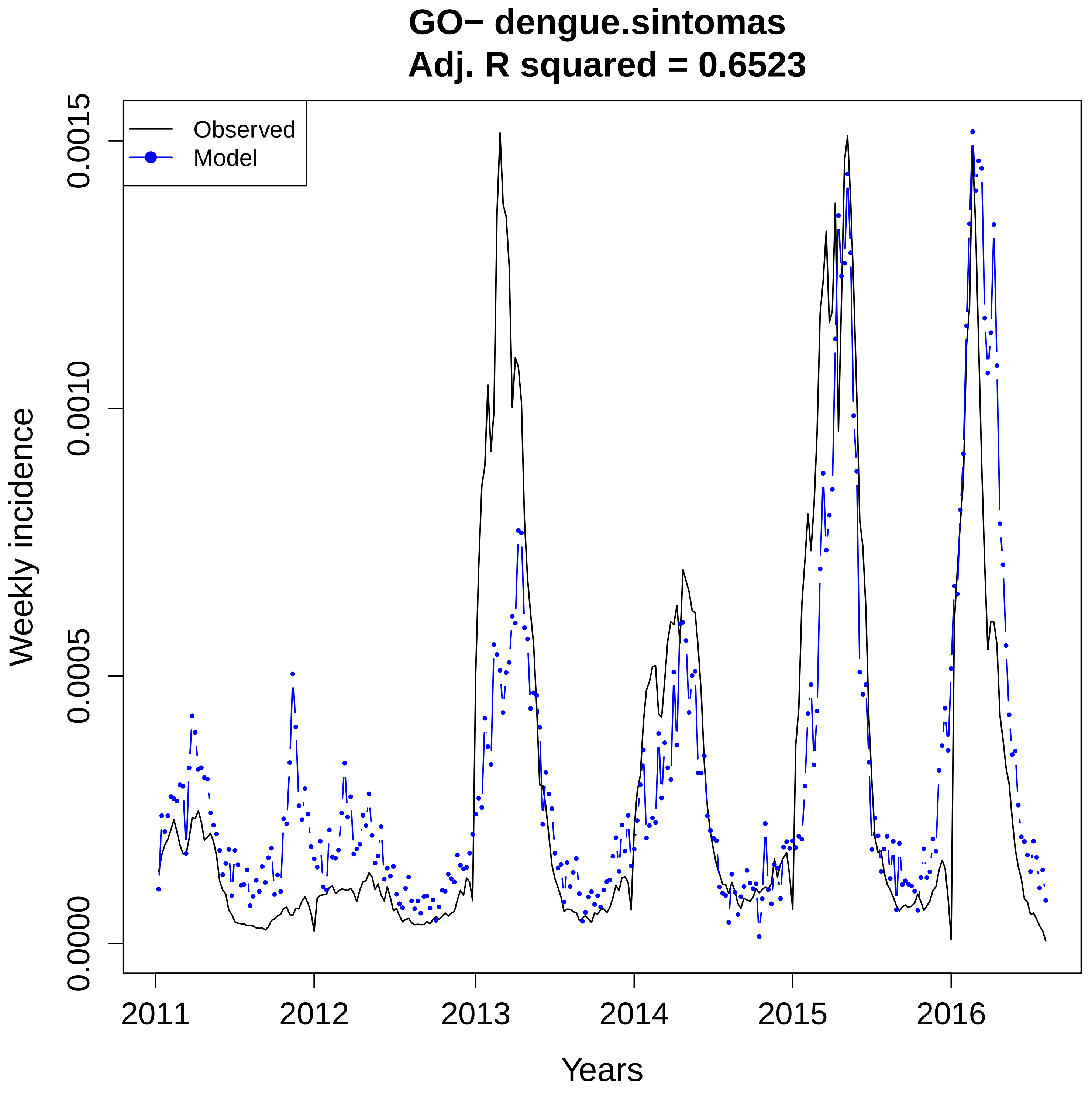
GO− dengue.sintomas
Adj. R squared = 0.6523
Observed
Model
Weekly incidence
0.0000
0.0005
0.0010
0.0015
2011
2012
2013
2014
2015
2016
Years

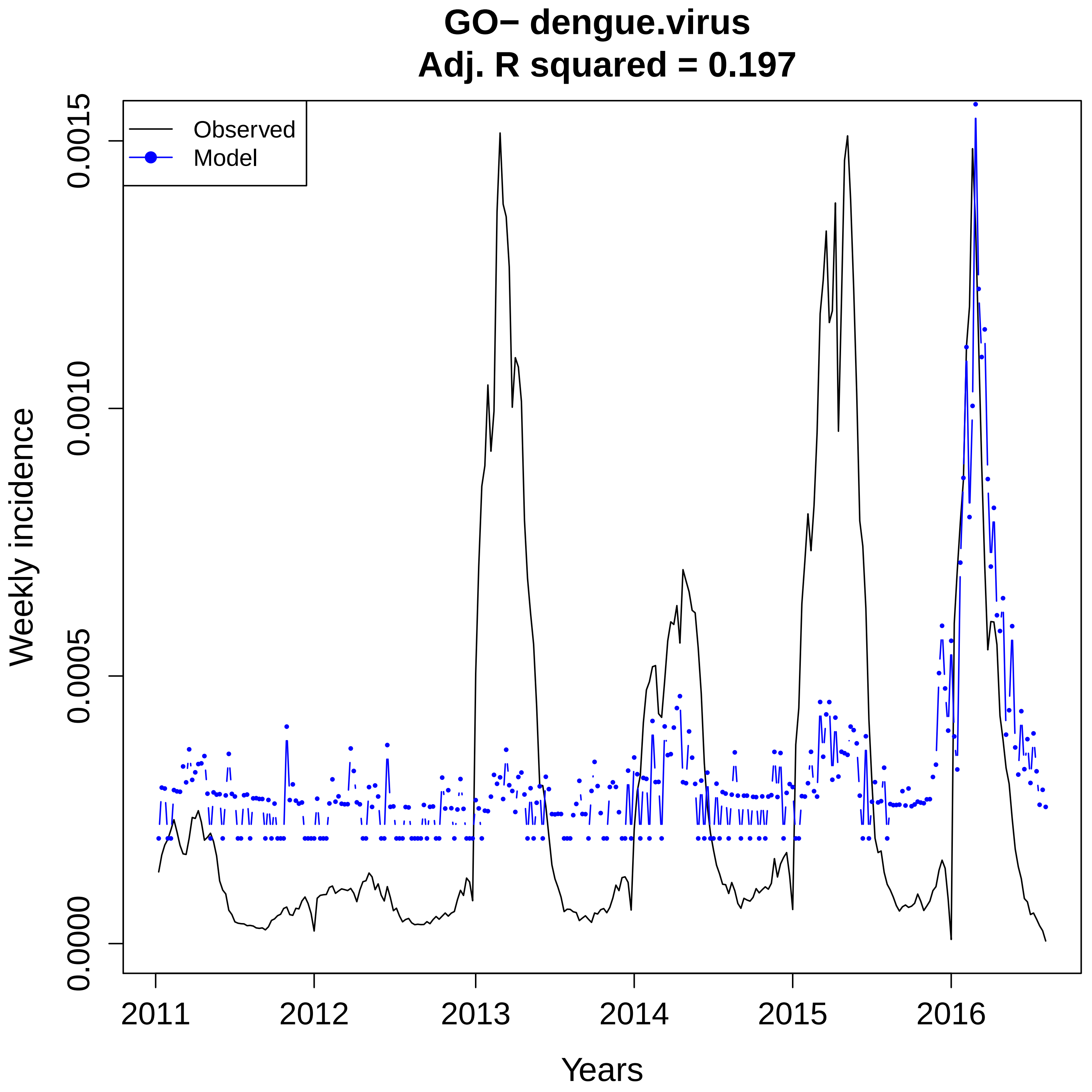
GO− dengue.virus
Adj. R squared = 0.197
Observed
Model
Weekly incidence
0.0000
0.0005
0.0010
0.0015
2011
2012
2013
2014
2015
2016
Years

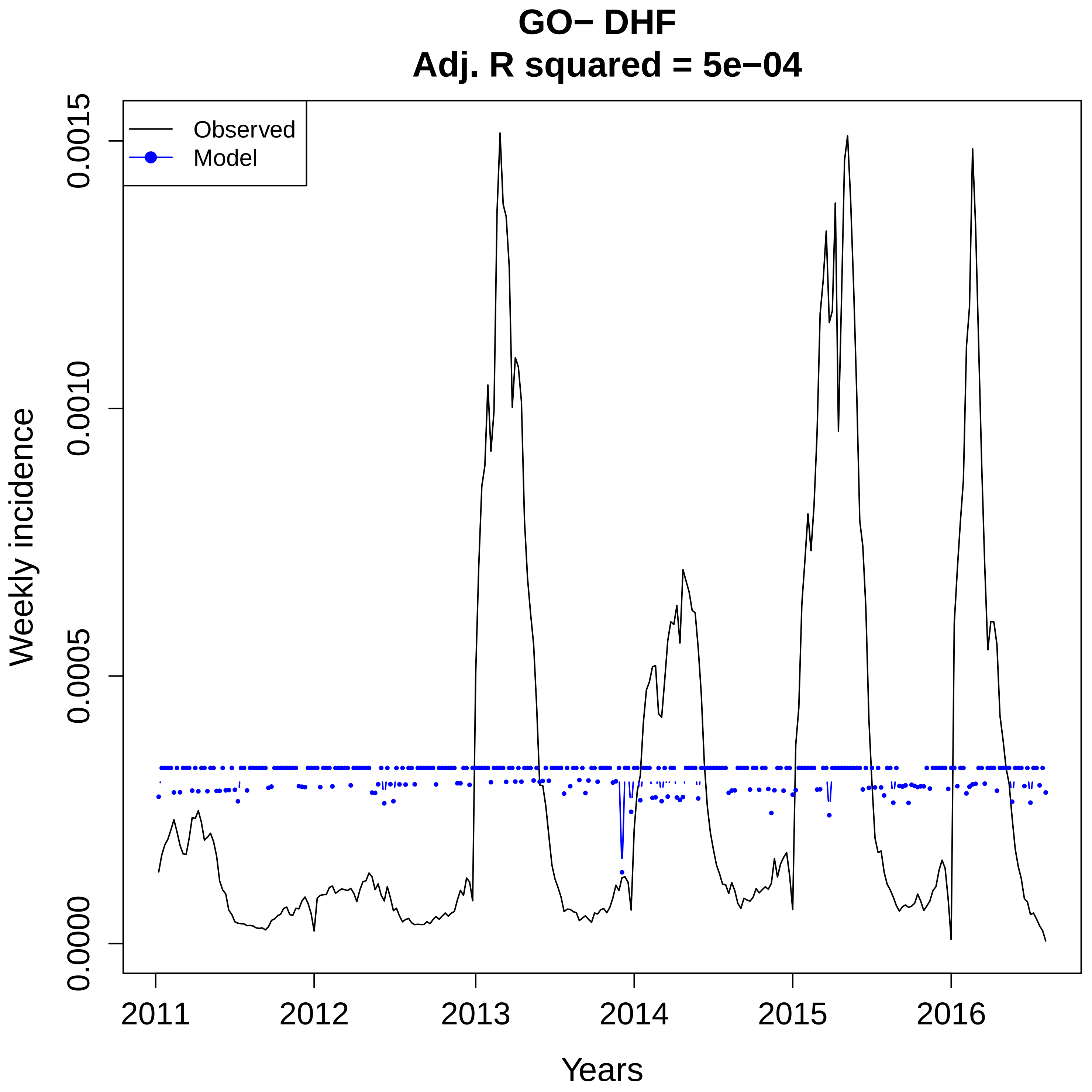
GO− DHF
Adj. R squared = 5e−04
Observed
Model
Weekly incidence
0.0000
0.0005
0.0010
0.0015
Years
2011
2012
2013
2014
2015
2016

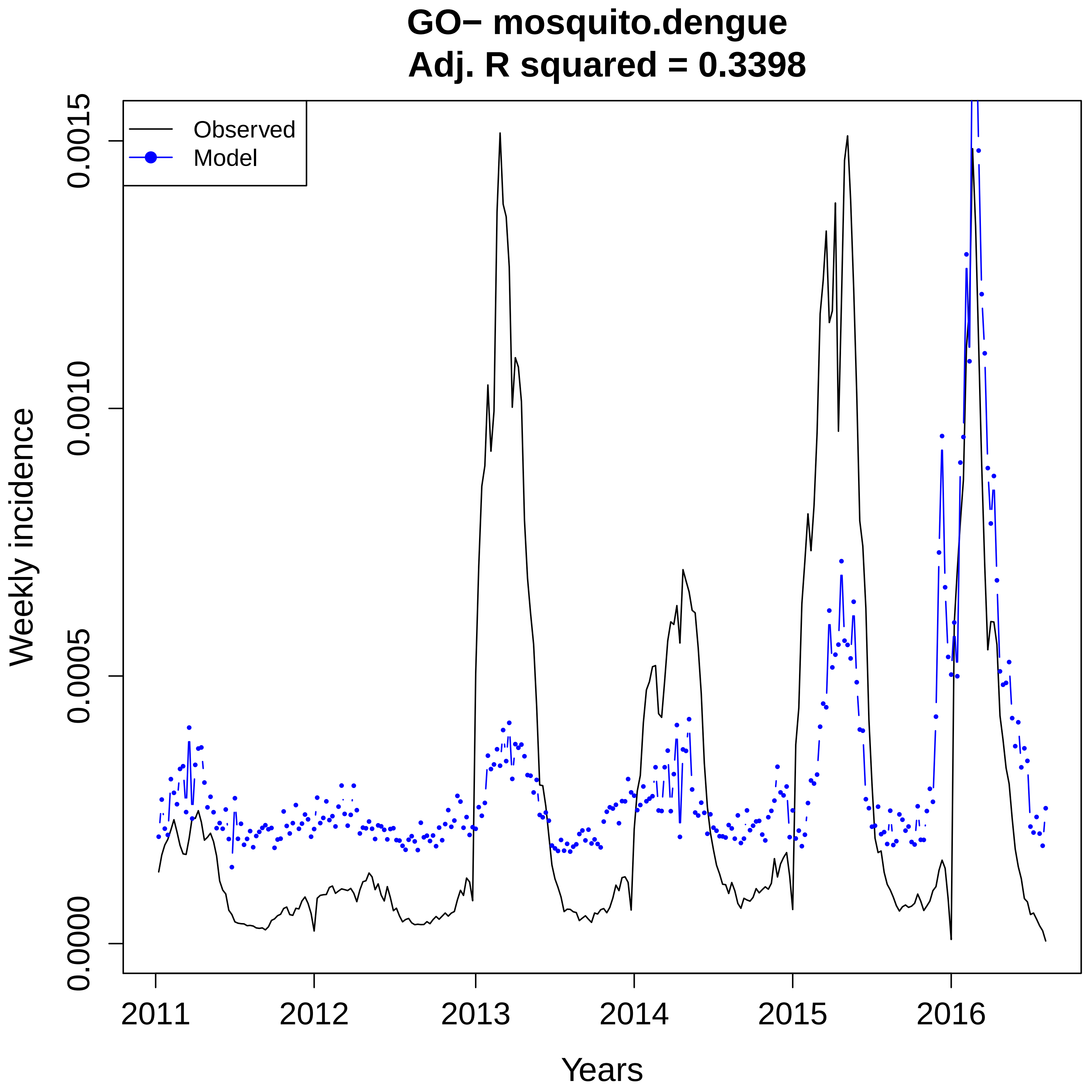
GO− mosquito.dengue
Adj. R squared = 0.3398
Observed
Model
Weekly incidence
0.0000
0.0005
0.0010
0.0015
2011
2012
2013
2014
2015
2016
Years

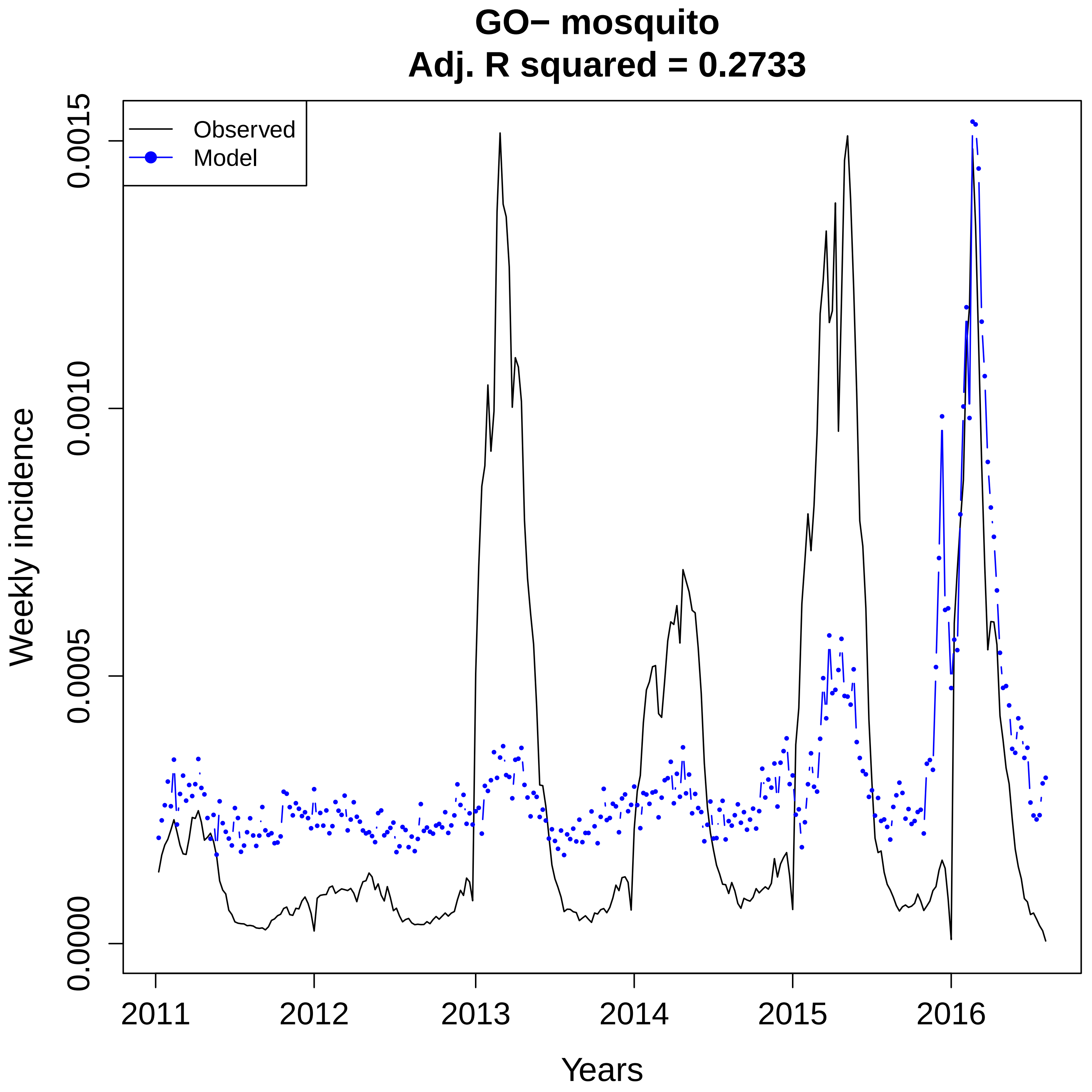

GO− mosquito
Adj. R squared = 0.2733
Observed
Model
Weekly incidence
0.0000
0.0005
0.0010
0.0015
Years
2011
2012
2013
2014
2015
2016

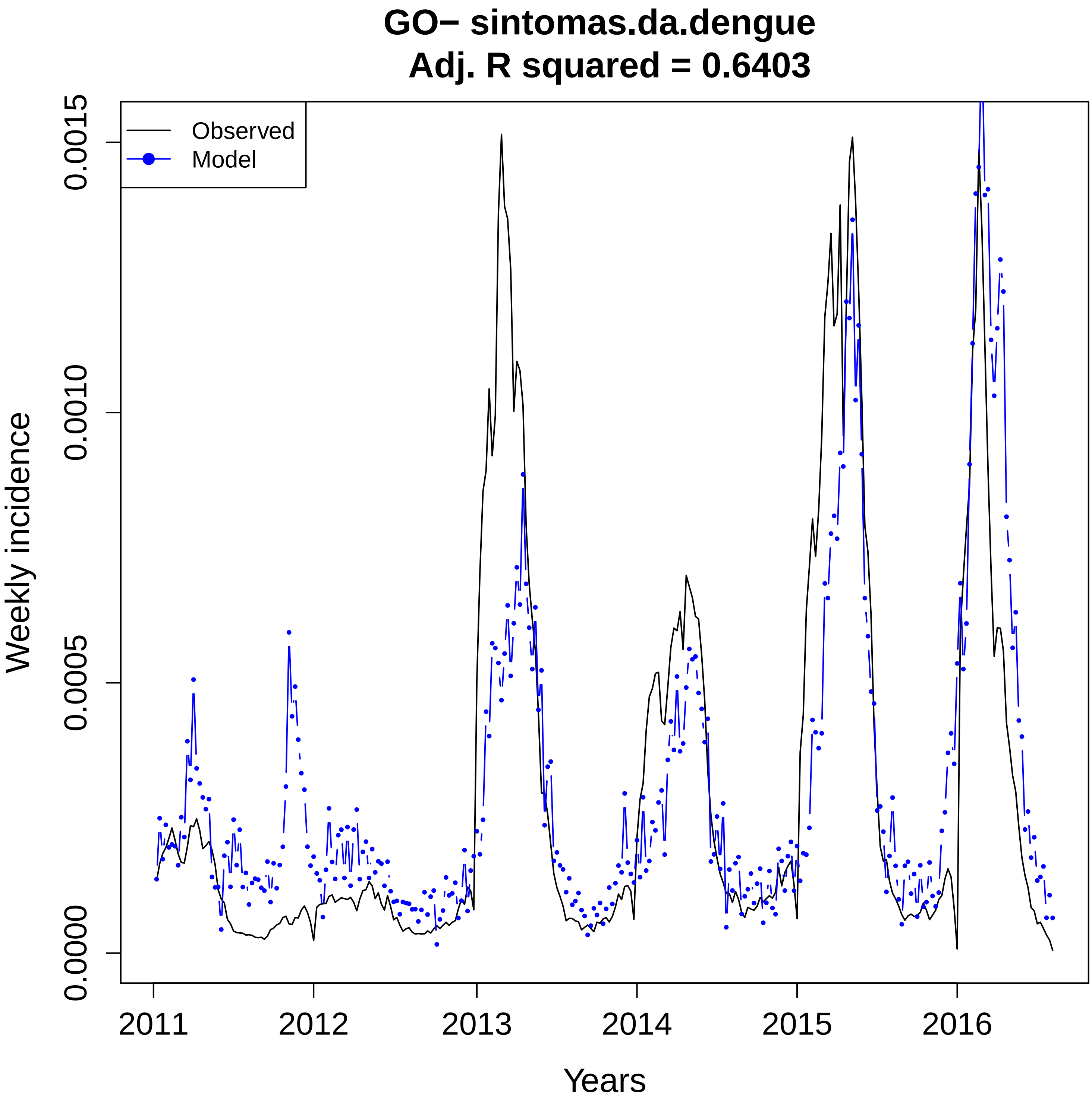
GO− sintomas.da.dengue
Adj. R squared = 0.6403
Observed
Model
Weekly incidence
0.0000
0.0005
0.0010
0.0015
2011
2012
2013
2014
2015
2016
Years

Maranhão (MA)
Dengue incidence vs. Google Health Trends (2011–2016)

Definitions:

- **Combined terms:**
  - **All:** model with all the available terms. n= number of terms
  - **Four:** model with "dengue", "dengue sintomas", "aedes", and "mosquito".
  - **Uncorrelated:** model with non-correlated terms. n = number of uncorrelated terms
- **Individual terms:** Plots developed with one term at a time.

- Terms for all and uncorrelated models can be found in additional file 3.

Combined terms
Adjusted R squared

Maranhão (MA)

All
(n = 9)

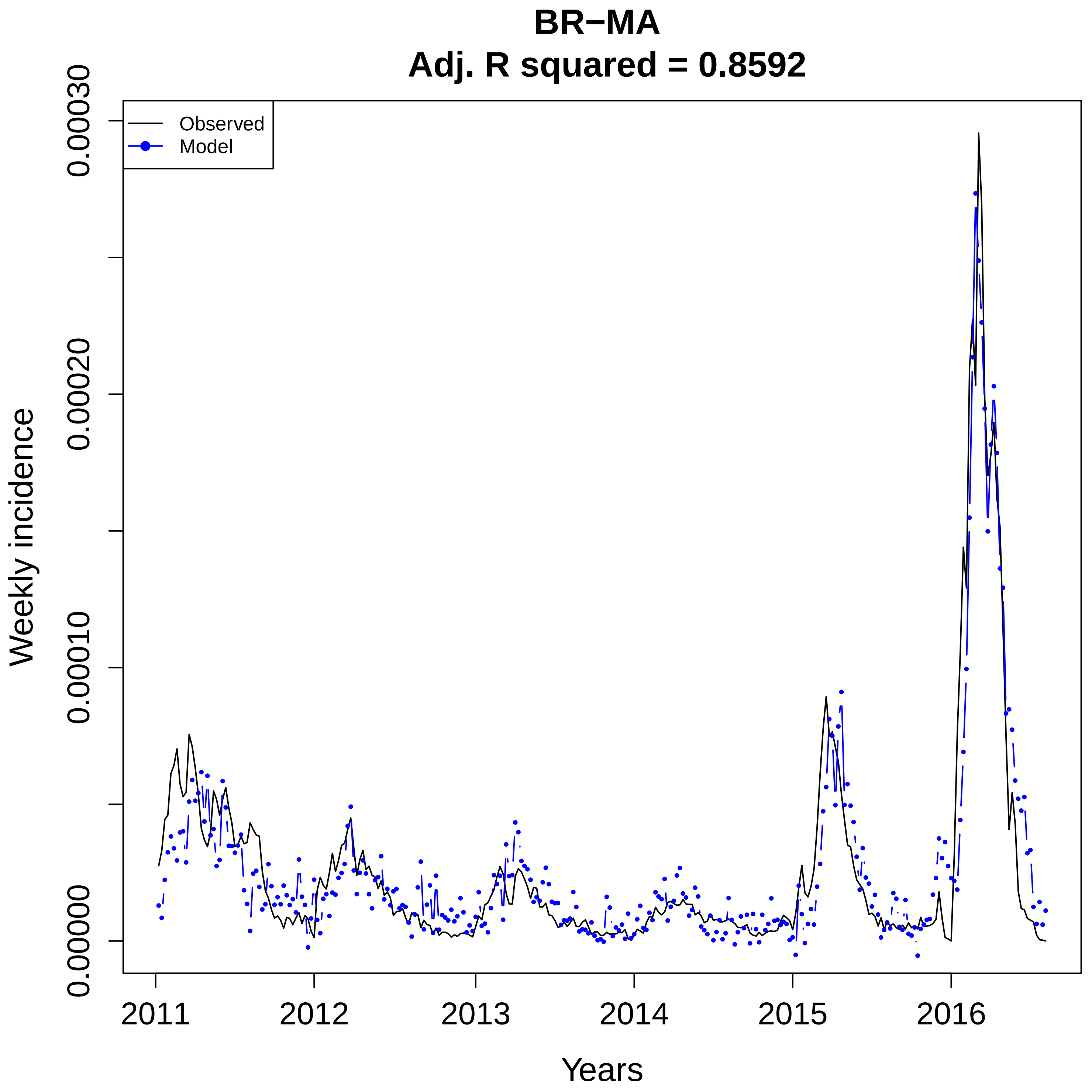
BR−MA
Adj. R squared = 0.8592
Observed
Model
Weekly incidence
0.00000
0.00010
0.00020
0.00030
2011
2012
2013
2014
2015
2016
Years

Four

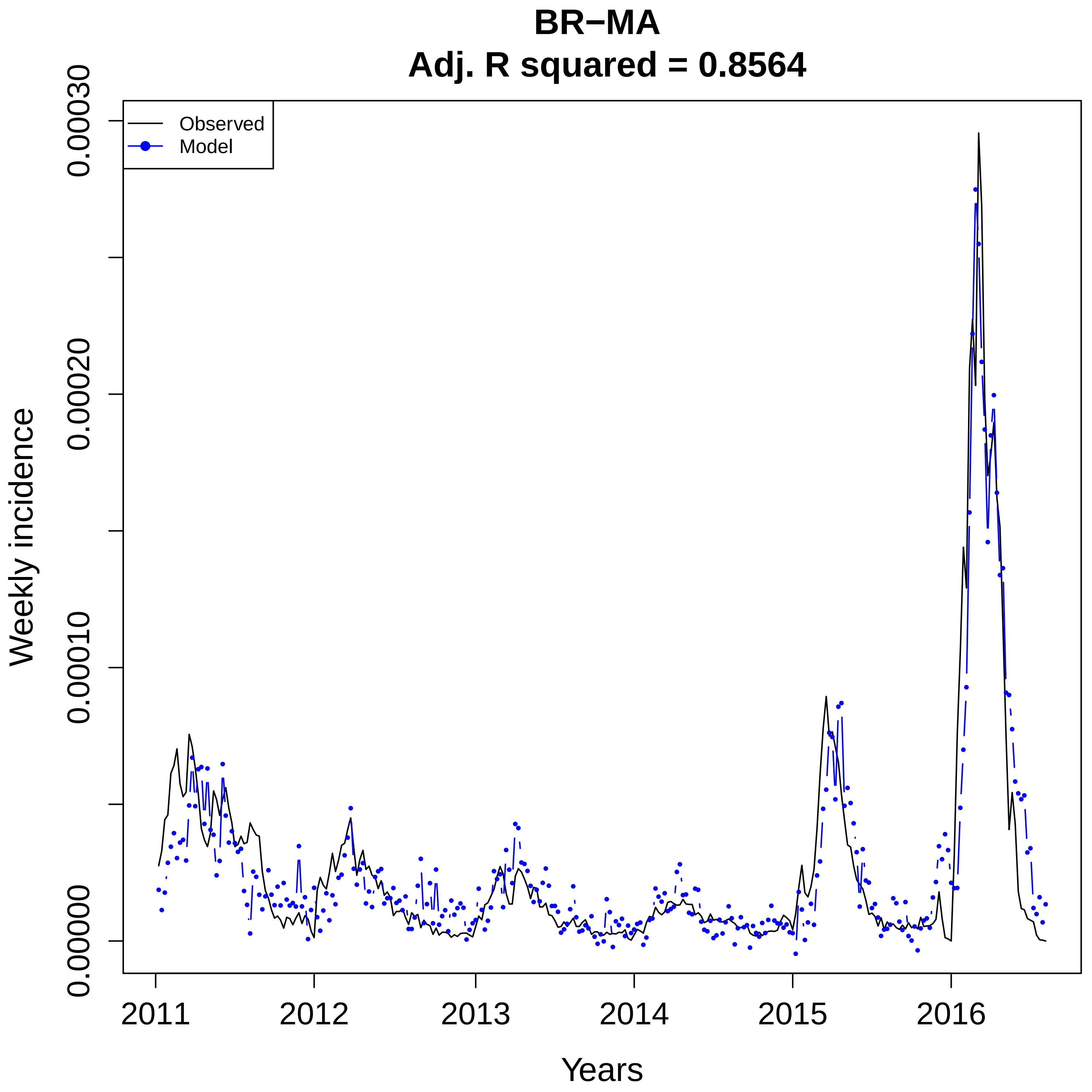

BR−MA
Adj. R squared = 0.8564
Observed
Model
0.00000
0.00010
0.00020
0.00030
Weekly incidence
2011
2012
2013
2014
2015
2016
Years

Uncorrelated
(n = 2)

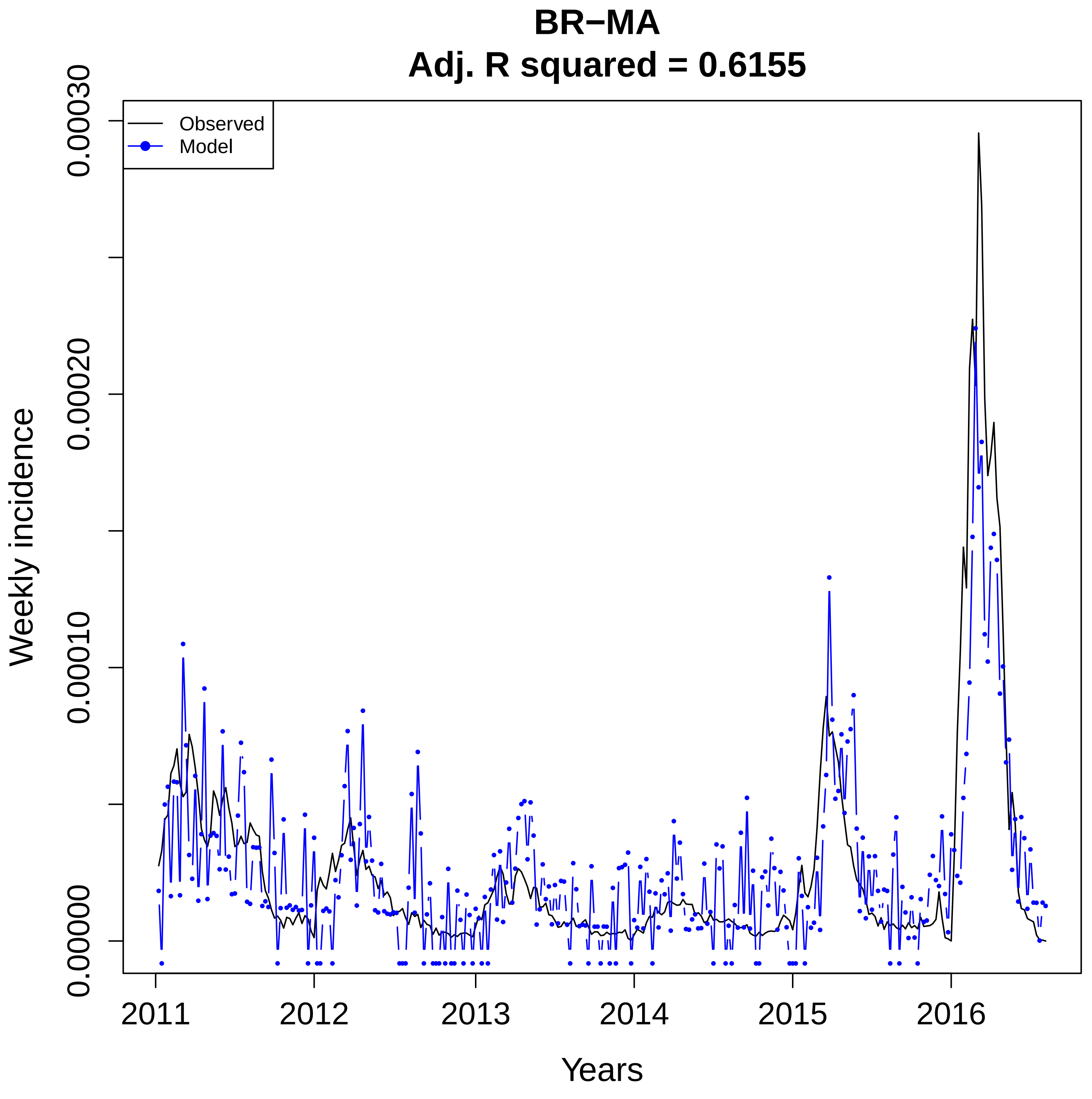

Individual terms
Adjusted R squared

Maranhão (MA)

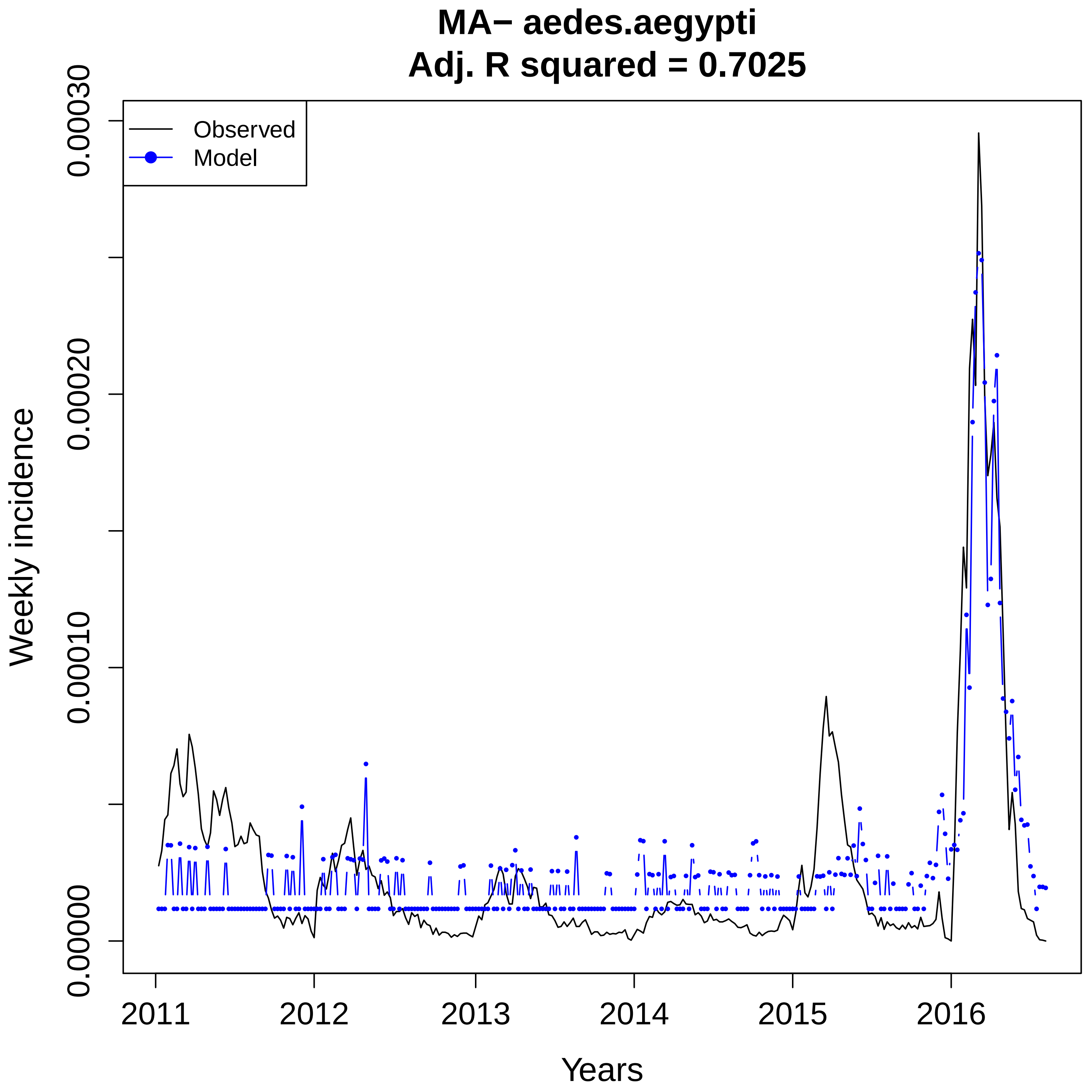

MA− aedes.aegypti
Adj. R squared = 0.7025
Observed
Model
Weekly incidence
0.00000
0.00010
0.00020
0.00030
2011
2012
2013
2014
2015
2016
Years

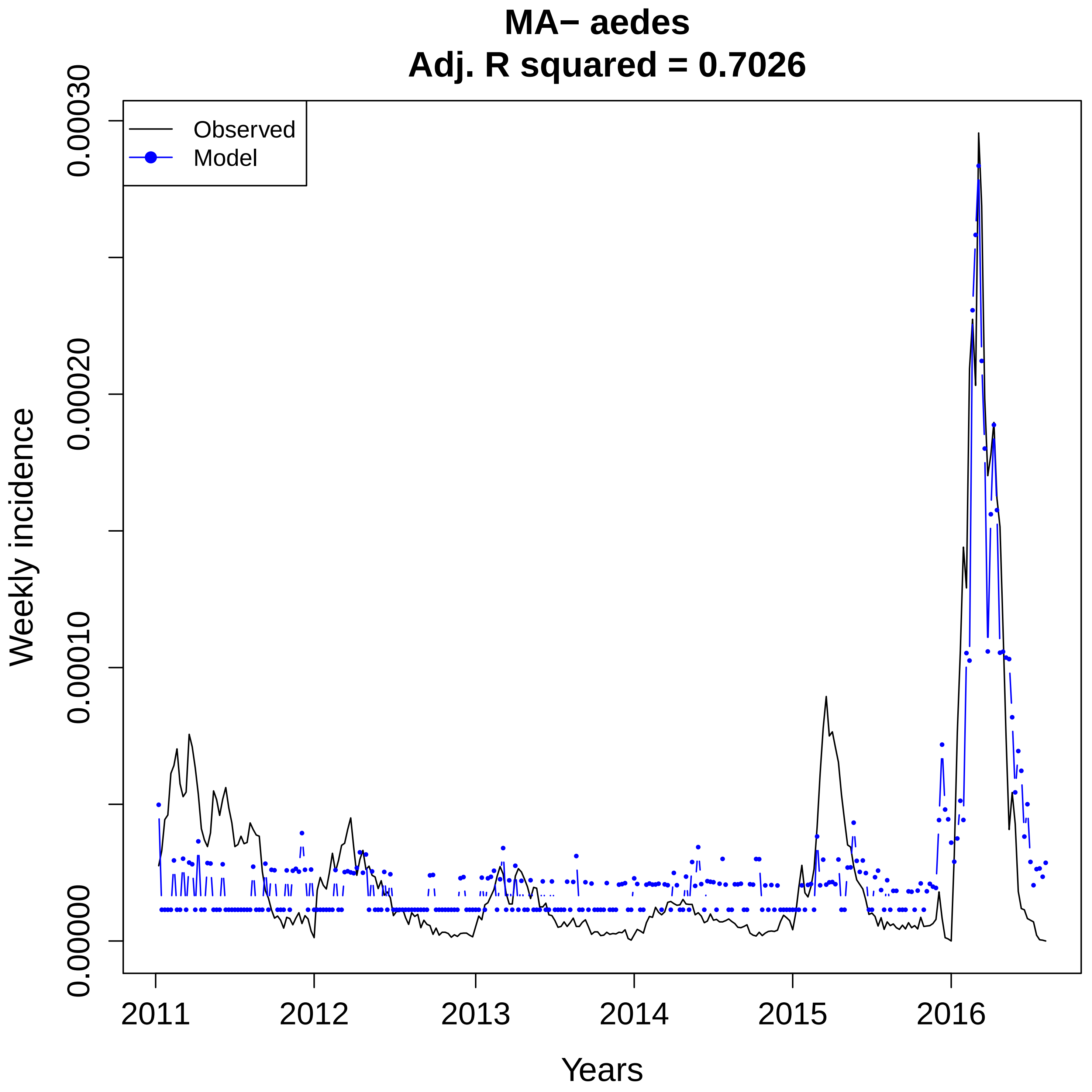

MA− aedes
Adj. R squared = 0.7026
Observed
Model
Weekly incidence
0.00000
0.00010
0.00020
0.00030
2011
2012
2013
2014
2015
2016
Years

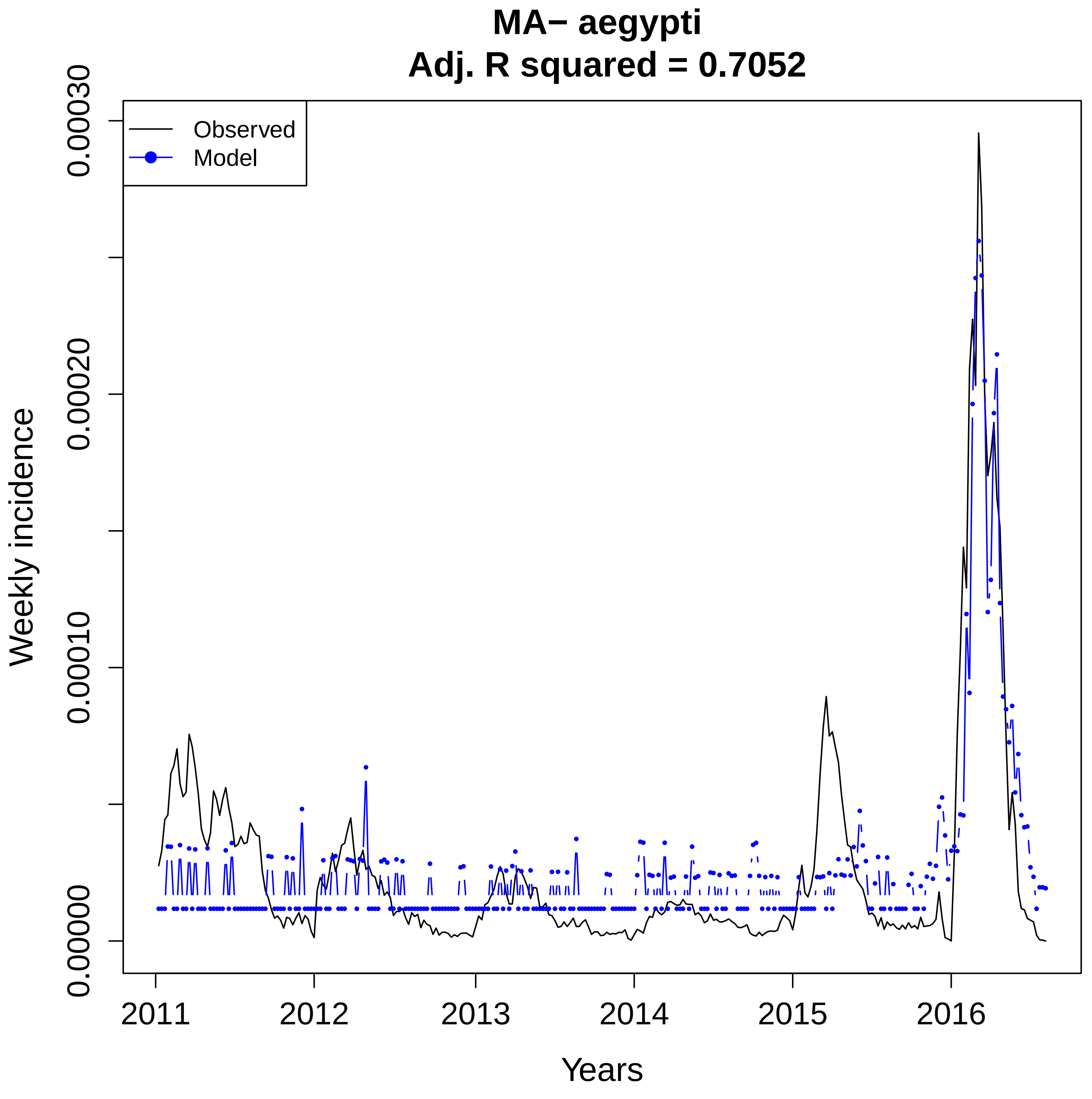

MA− aegypti
Adj. R squared = 0.7052
Observed
Model
Weekly incidence
0.00000
0.00010
0.00020
0.00030
2011
2012
2013
2014
2015
2016
Years

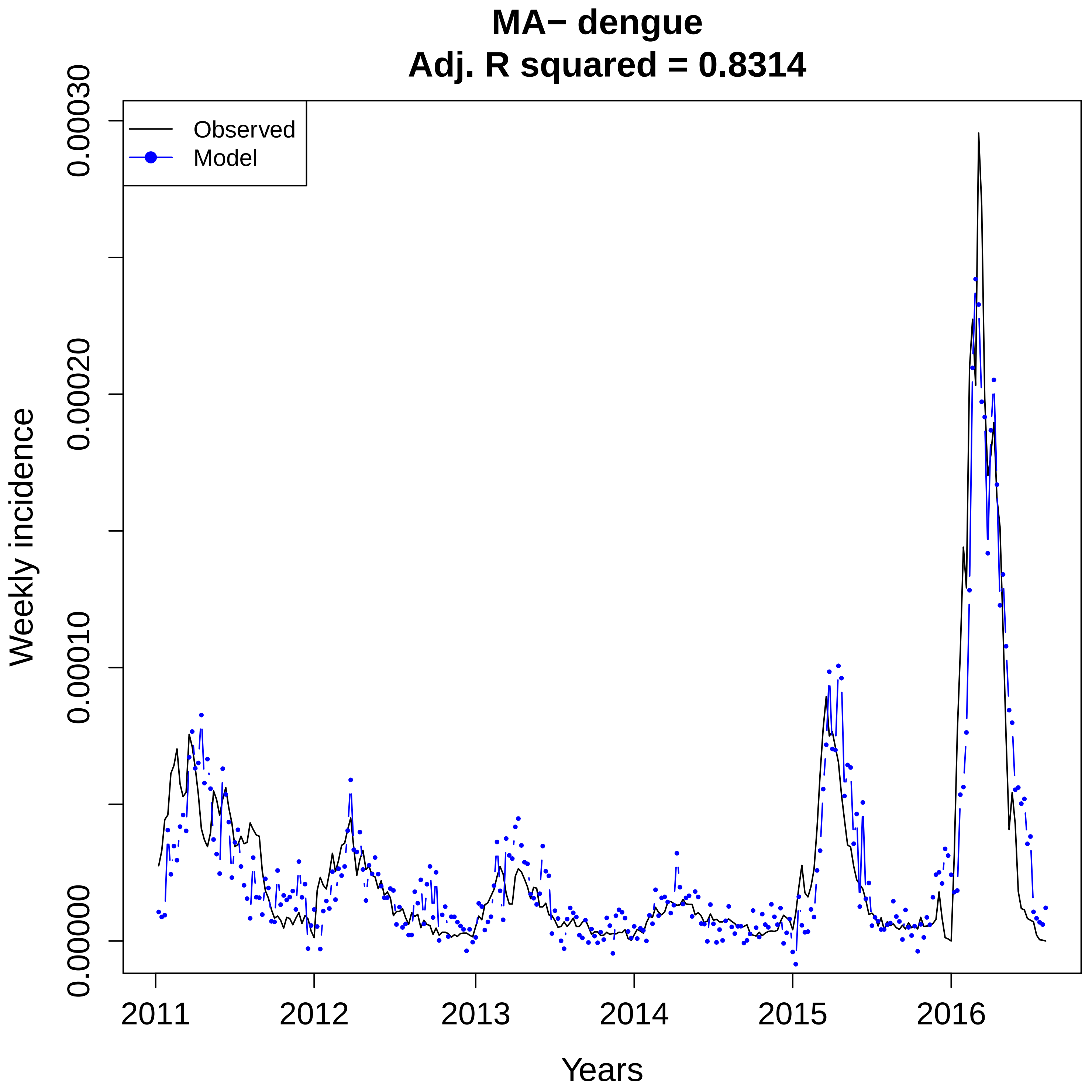
MA− dengue
Adj. R squared = 0.8314
Observed
Model
Weekly incidence
0.00000
0.00010
0.00020
0.00030
2011
2012
2013
2014
2015
2016
Years

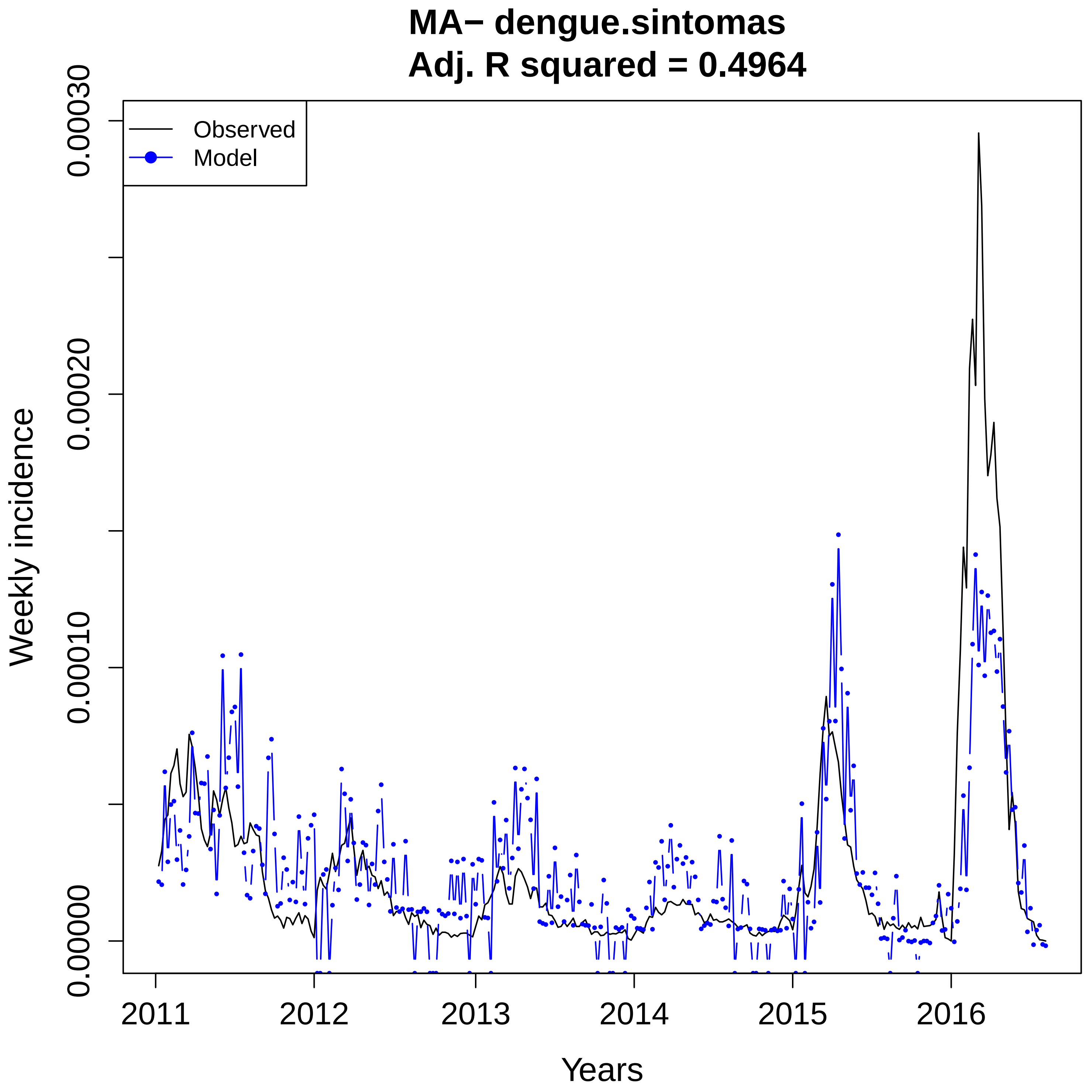

MA− dengue.sintomas
Adj. R squared = 0.4964
Observed
Model
Weekly incidence
0.00000
0.00010
0.00020
0.00030
2011
2012
2013
2014
2015
2016
Years

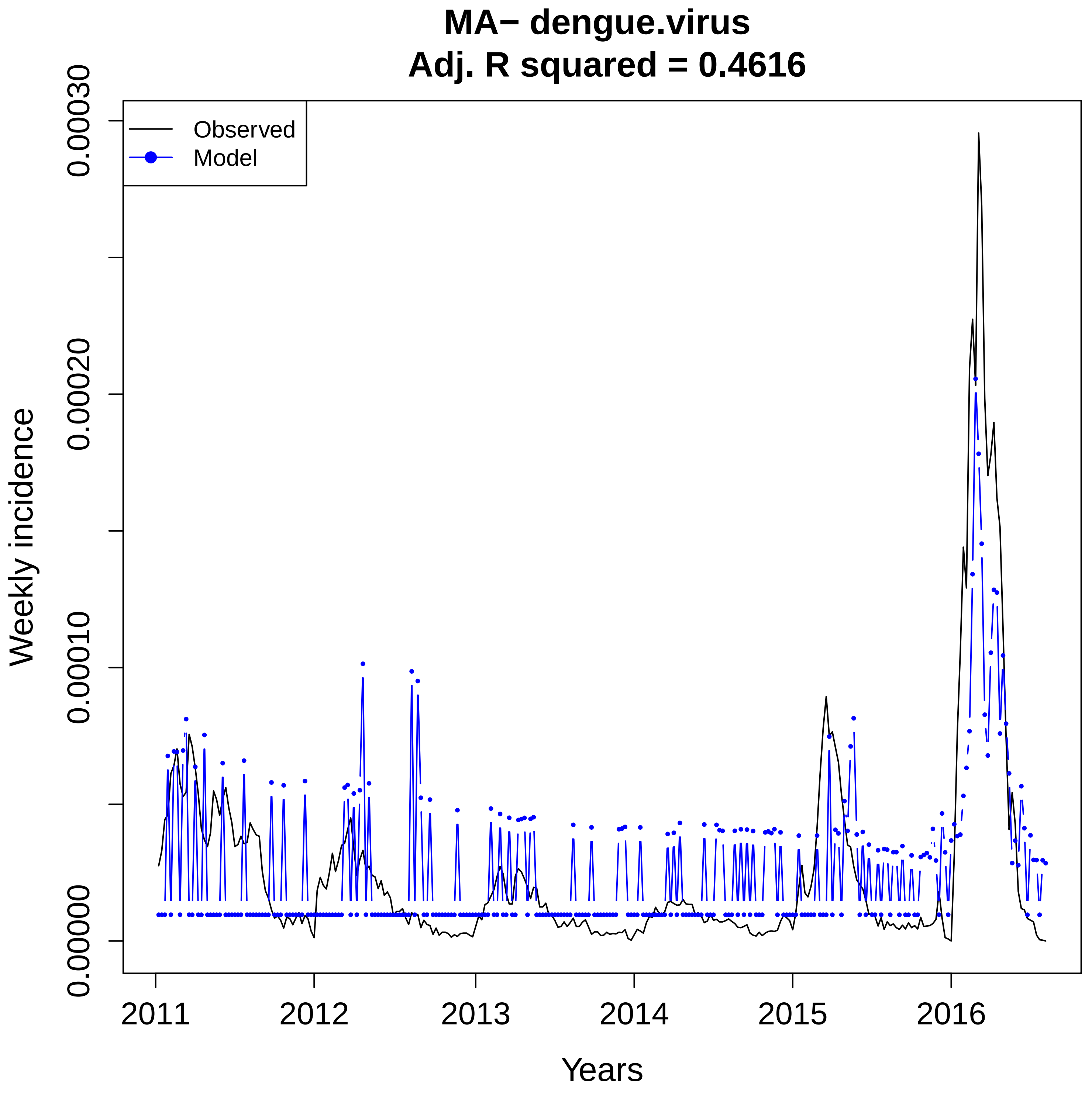
MA− dengue.virus
Adj. R squared = 0.4616
Observed
Model
Weekly incidence
0.00000
0.00010
0.00020
0.00030
2011
2012
2013
2014
2015
2016
Years

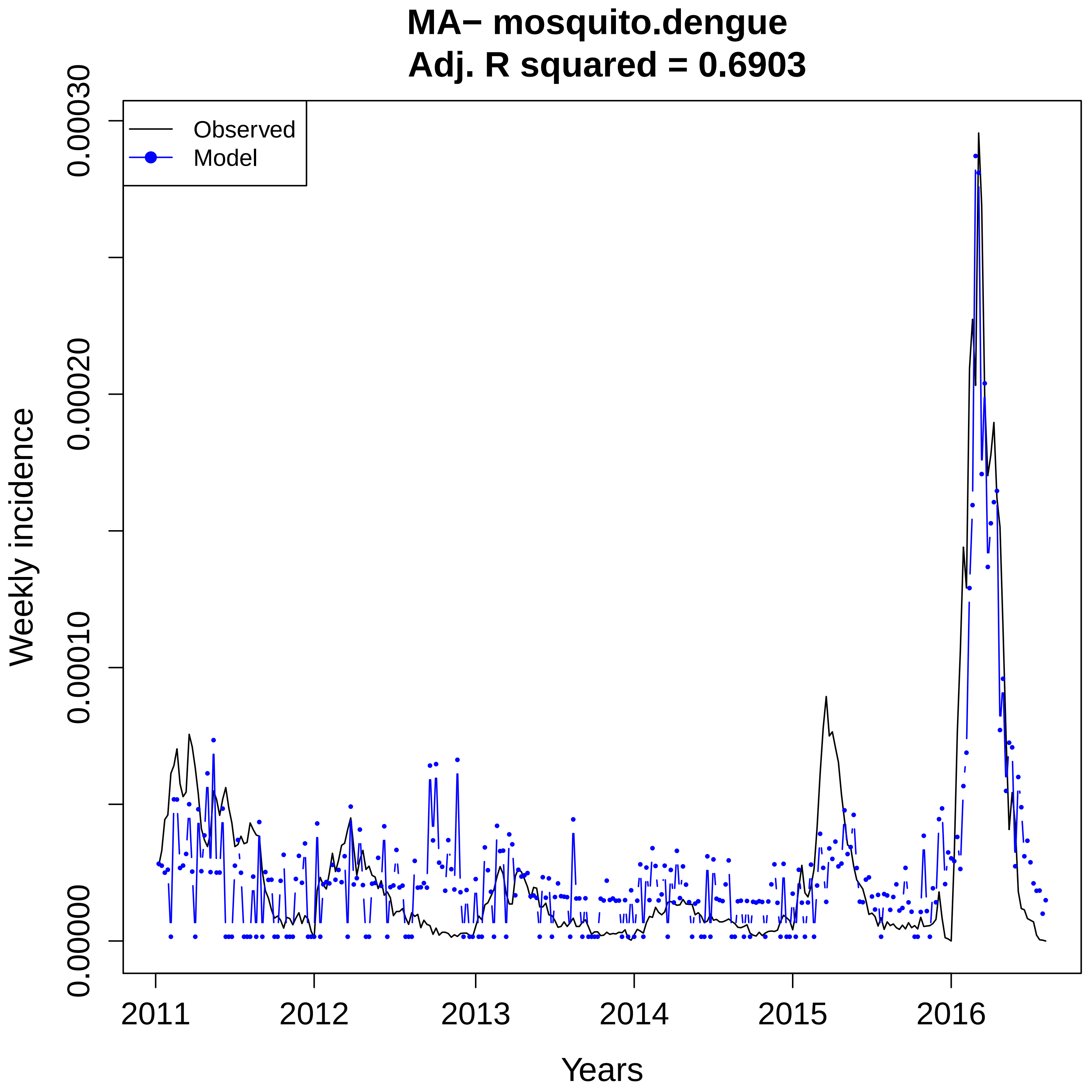

MA− mosquito.dengue
Adj. R squared = 0.6903
Observed
Model
Weekly incidence
0.00000
0.00010
0.00020
0.00030
2011
2012
2013
2014
2015
2016
Years

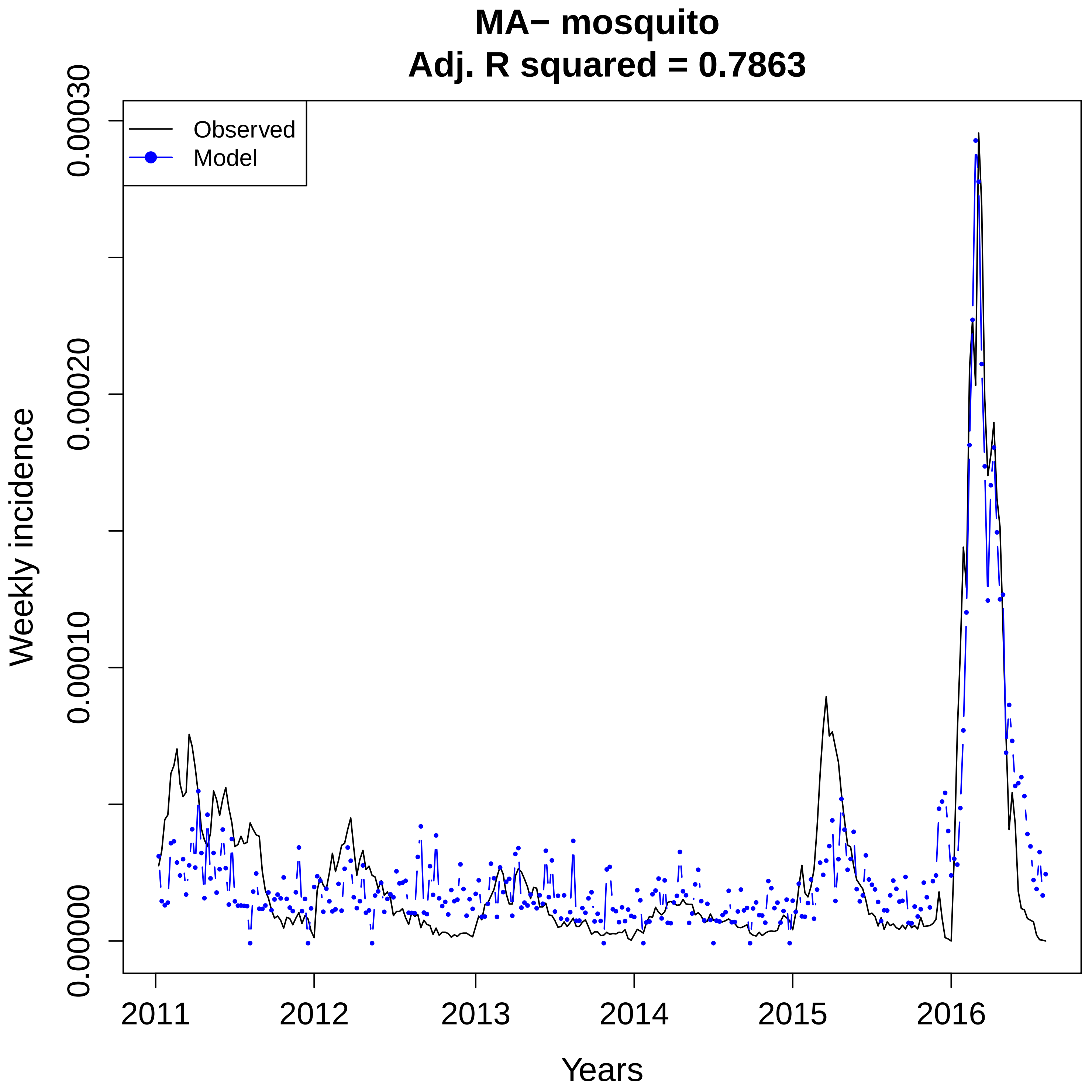
MA− mosquito
Adj. R squared = 0.7863
Observed
Model
Weekly incidence
0.00000
0.00010
0.00020
0.00030
2011
2012
2013
2014
2015
2016
Years

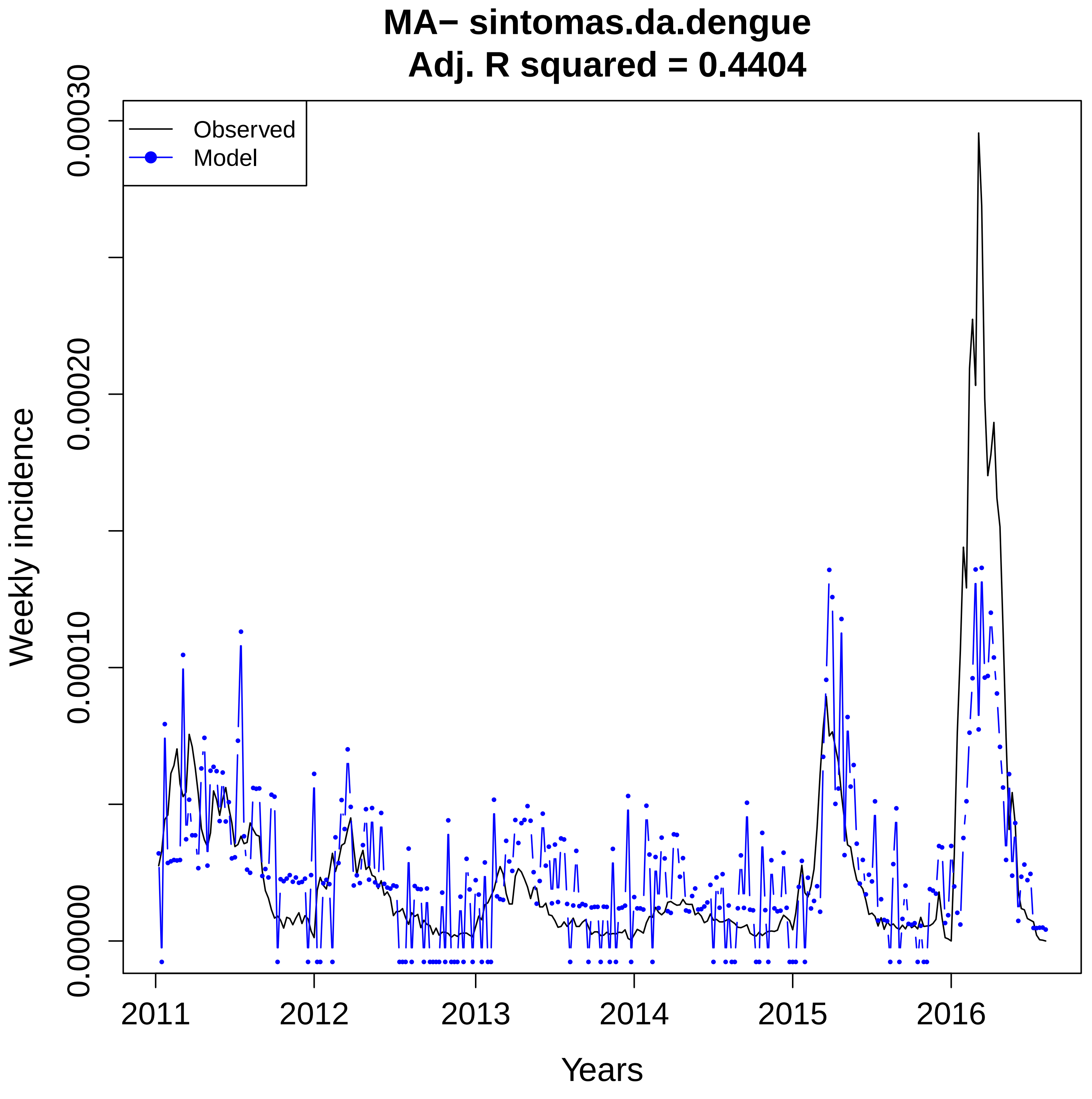

MA− sintomas.da.dengue
Adj. R squared = 0.4404
Observed
Model
Weekly incidence
0.00000
0.00010
0.00020
0.00030
2011
2012
2013
2014
2015
2016
Years

Minas Gerais (MG)

Dengue incidence vs. Google Health Trends (2011–2016)

Definitions:

- **Combined terms:**
  - **All:** model with all the available terms. n= number of terms
  - **Four:** model with "dengue", "dengue sintomas", "aedes", and "mosquito".
  - **Uncorrelated:** model with non-correlated terms. n = number of uncorrelated terms
- **Individual terms:** Plots developed with one term at a time.

- Terms for all and uncorrelated models can be found in additional file 3.

Combined terms
Adjusted R squared

Minas Gerais (MG)

All
(n = 12)

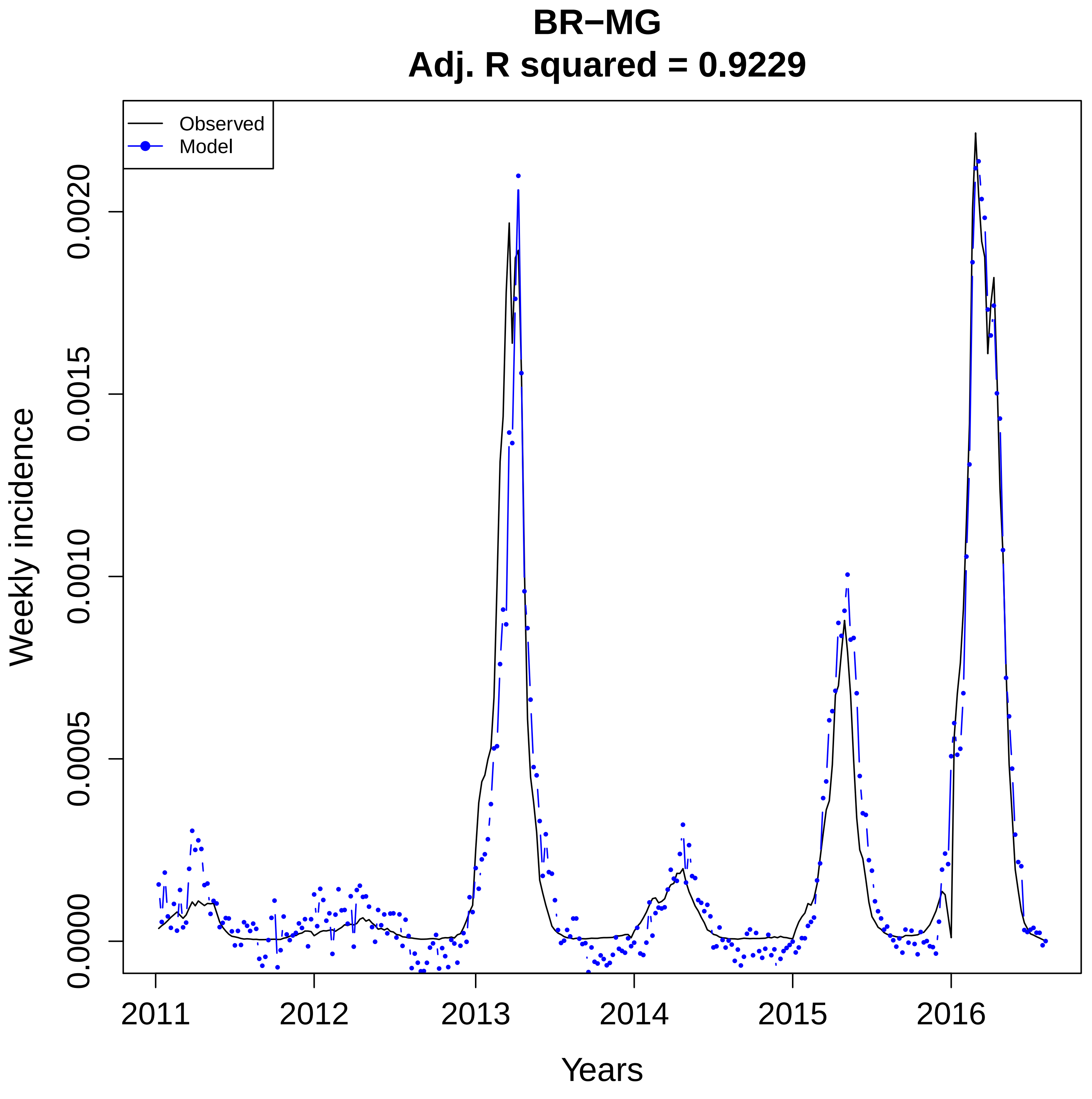

BR−MG
Adj. R squared = 0.9229
Observed
Model
Weekly incidence
0.0000
0.0005
0.0010
0.0015
0.0020
2011
2012
2013
2014
2015
2016
Years

Four

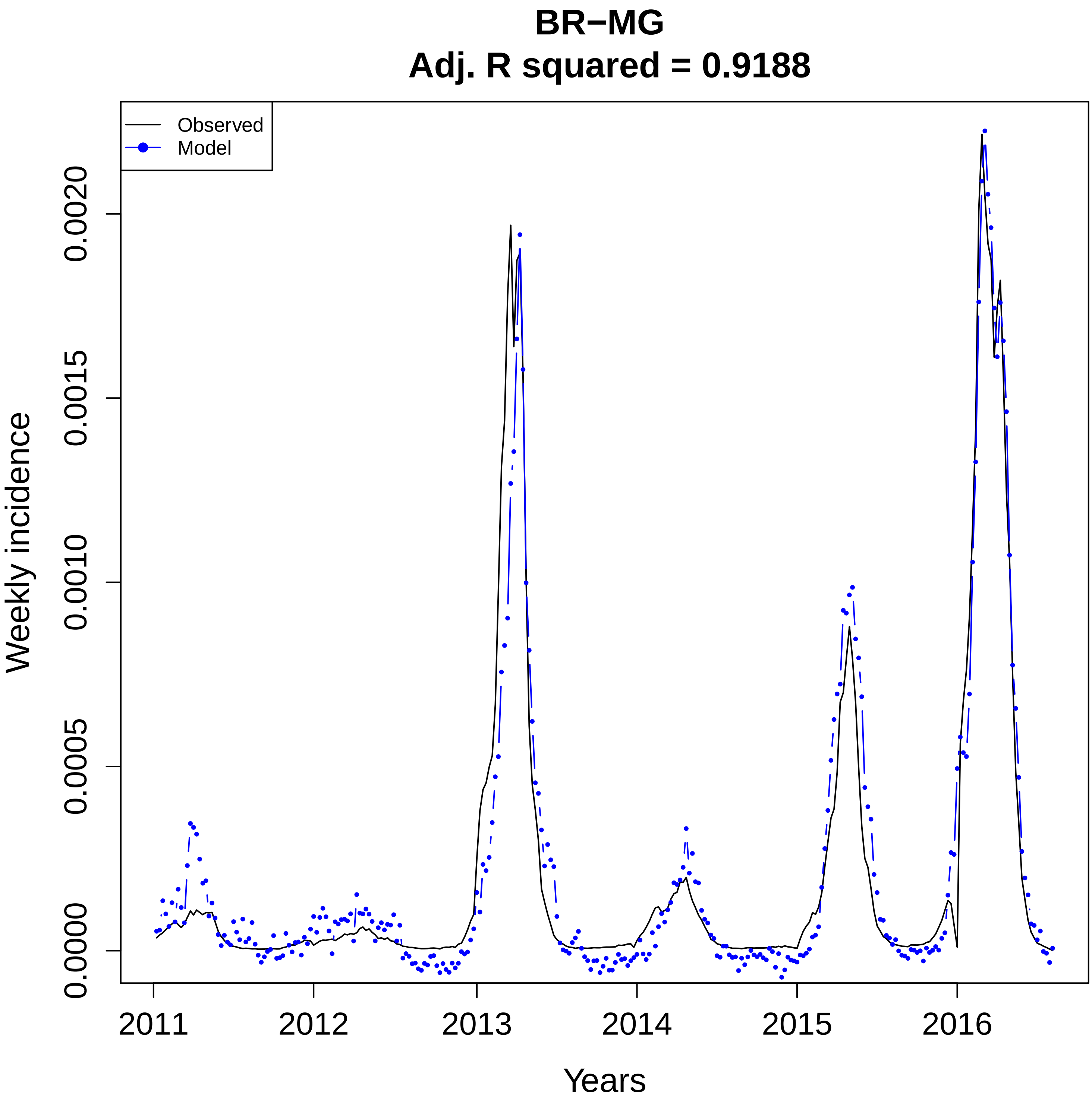
BR−MG
Adj. R squared = 0.9188
Observed
Model
Weekly incidence
0.0000
0.0005
0.0010
0.0015
0.0020
2011
2012
2013
2014
2015
2016
Years

Uncorrelated
(n = 4)

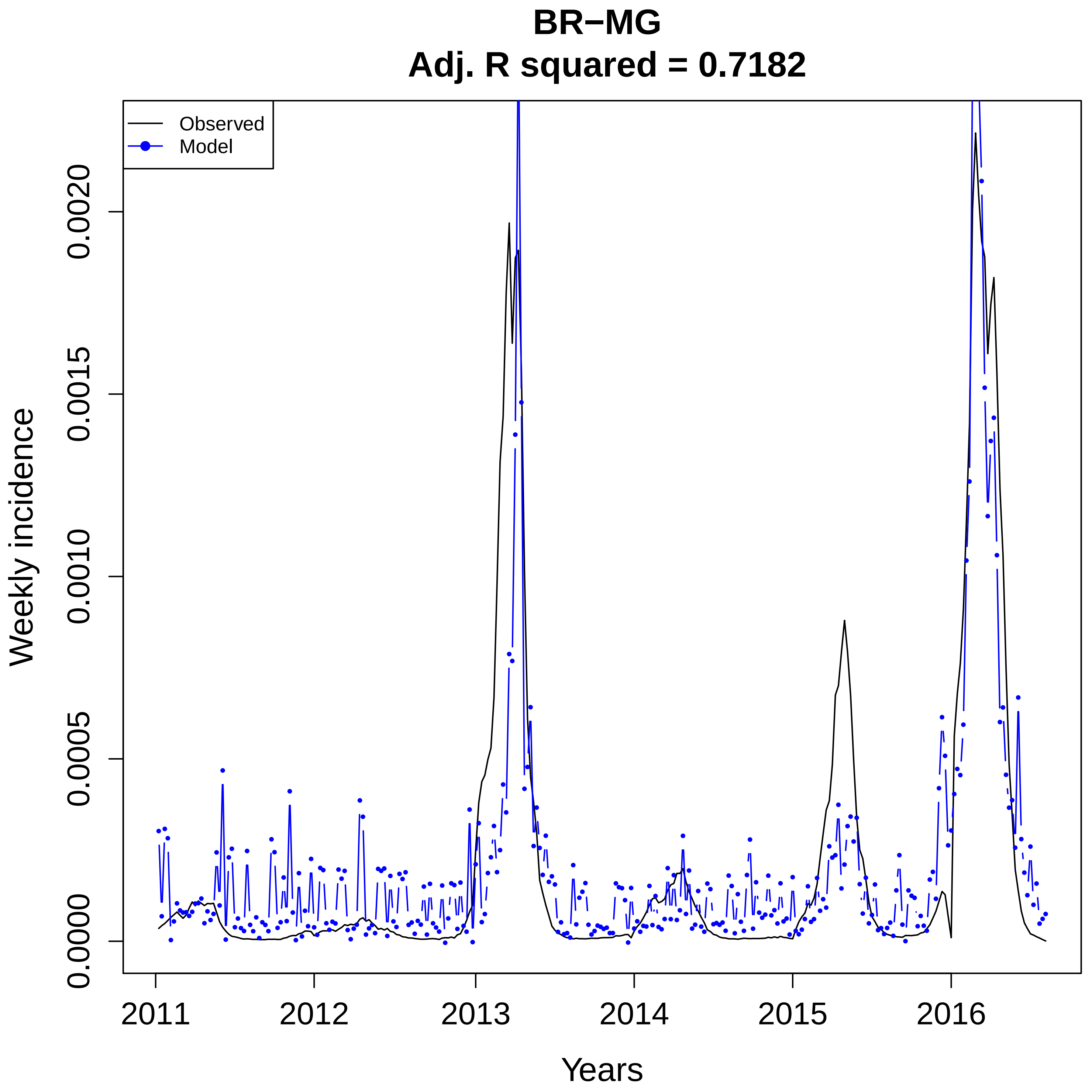

Individual terms
Adjusted R squared

Minas Gerais (MG)

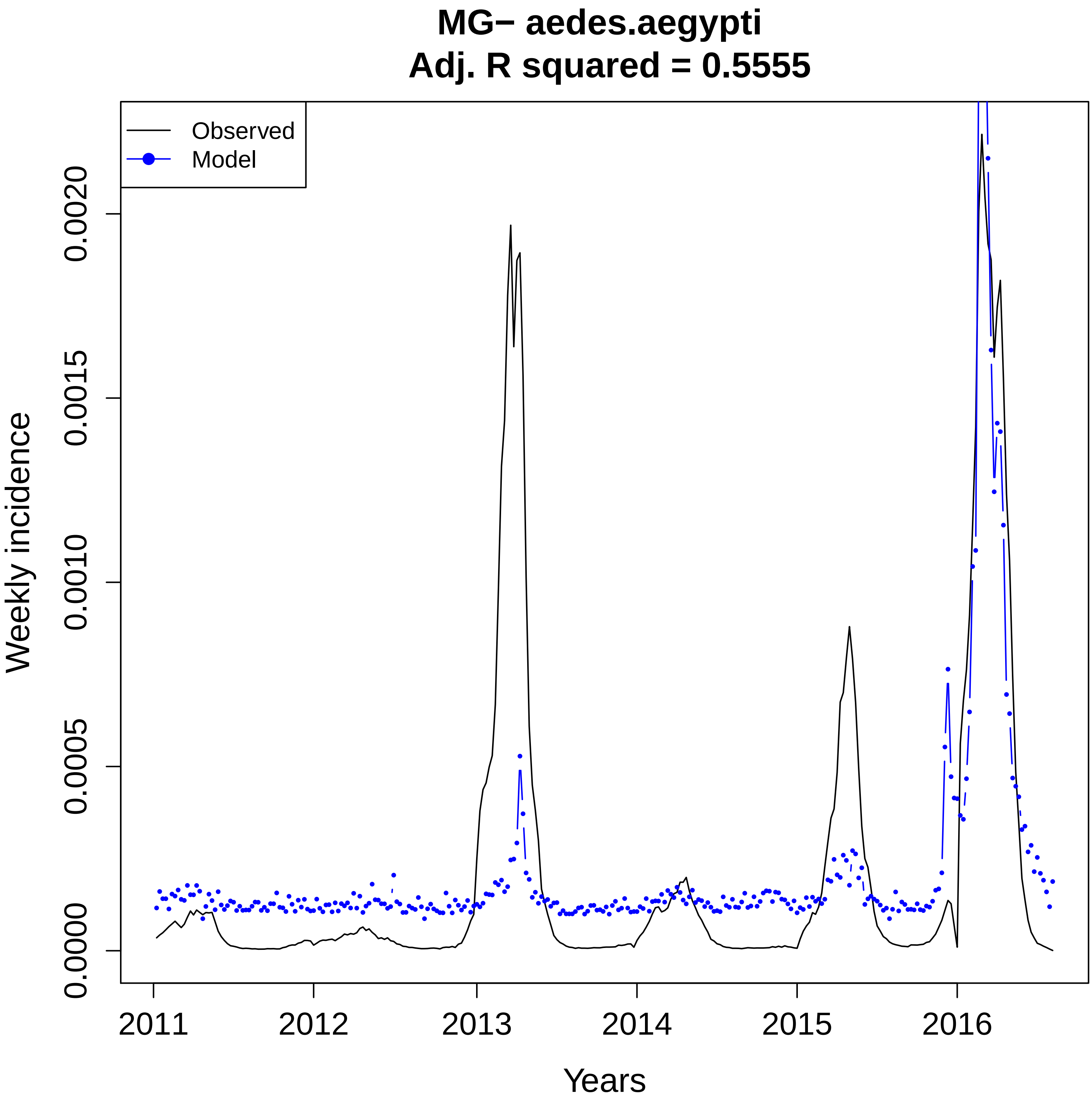
MG− aedes.aegypti
Adj. R squared = 0.5555
Observed
Model
Weekly incidence
0.0000
0.0005
0.0010
0.0015
0.0020
2011
2012
2013
2014
2015
2016
Years

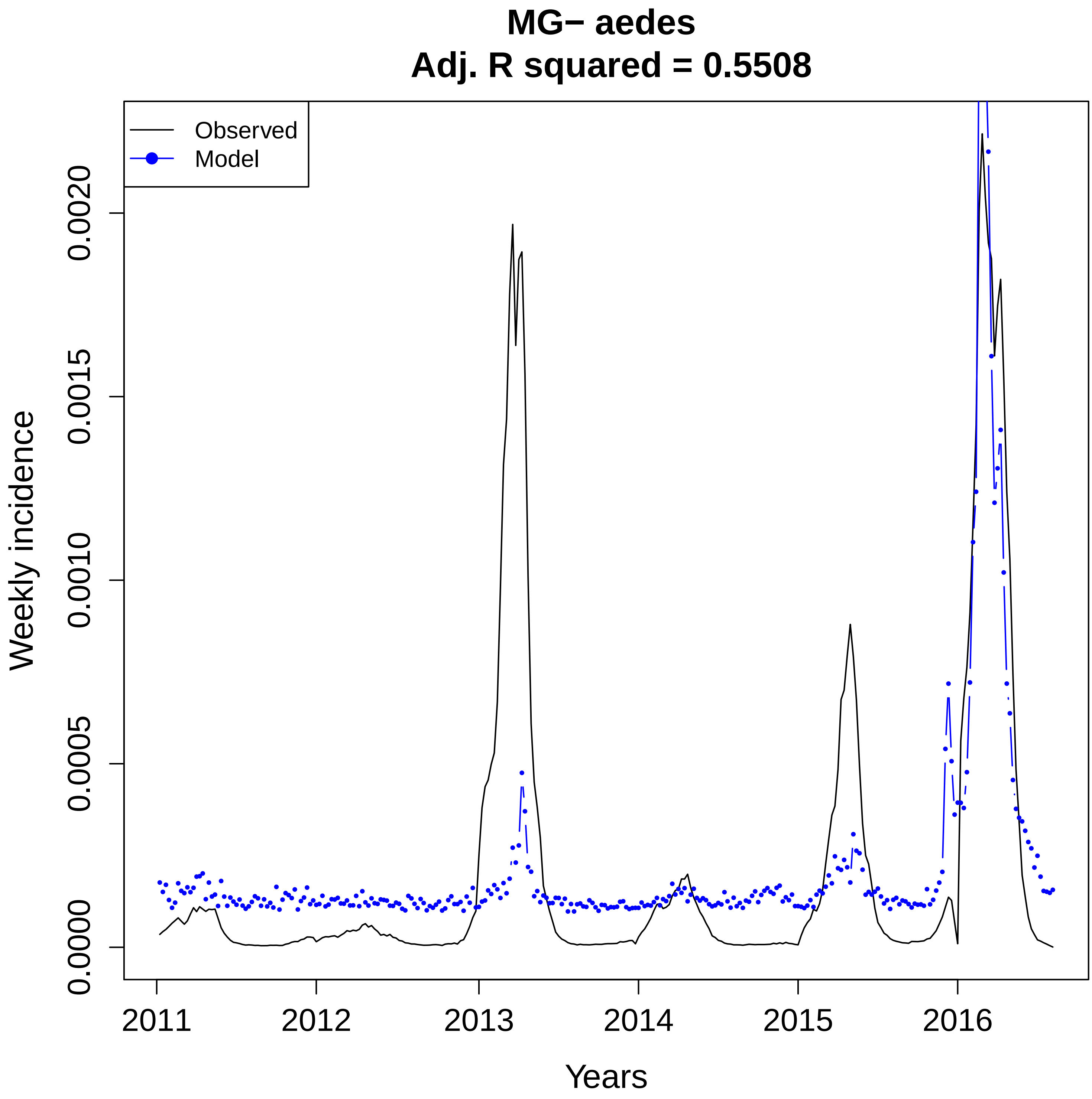
MG− aedes
Adj. R squared = 0.5508
Observed
Model
Weekly incidence
0.0000
0.0005
0.0010
0.0015
0.0020
2011
2012
2013
2014
2015
2016
Years

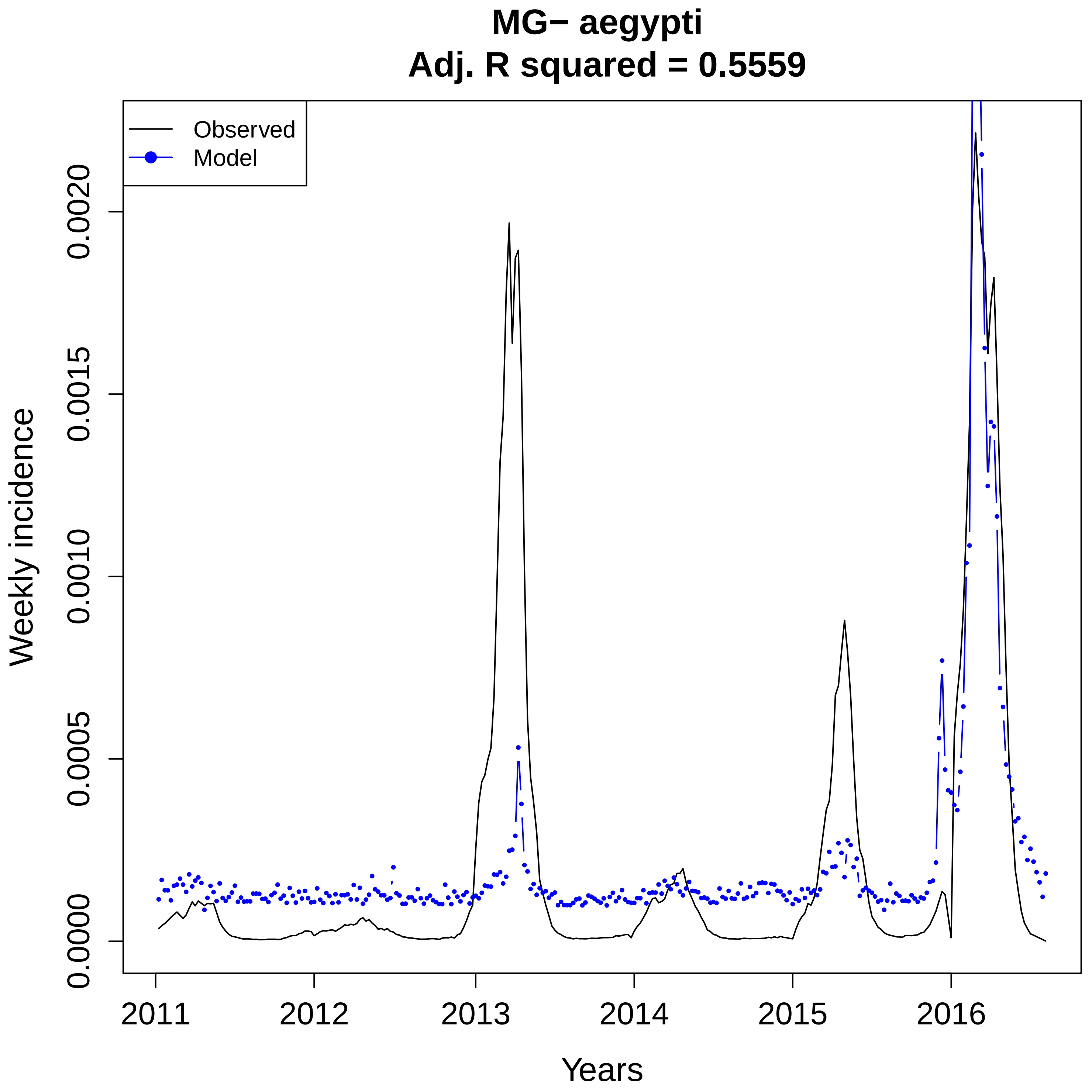
MG− aegypti
Adj. R squared = 0.5559
Observed
Model
Weekly incidence
0.0000
0.0005
0.0010
0.0015
0.0020
2011
2012
2013
2014
2015
2016
Years

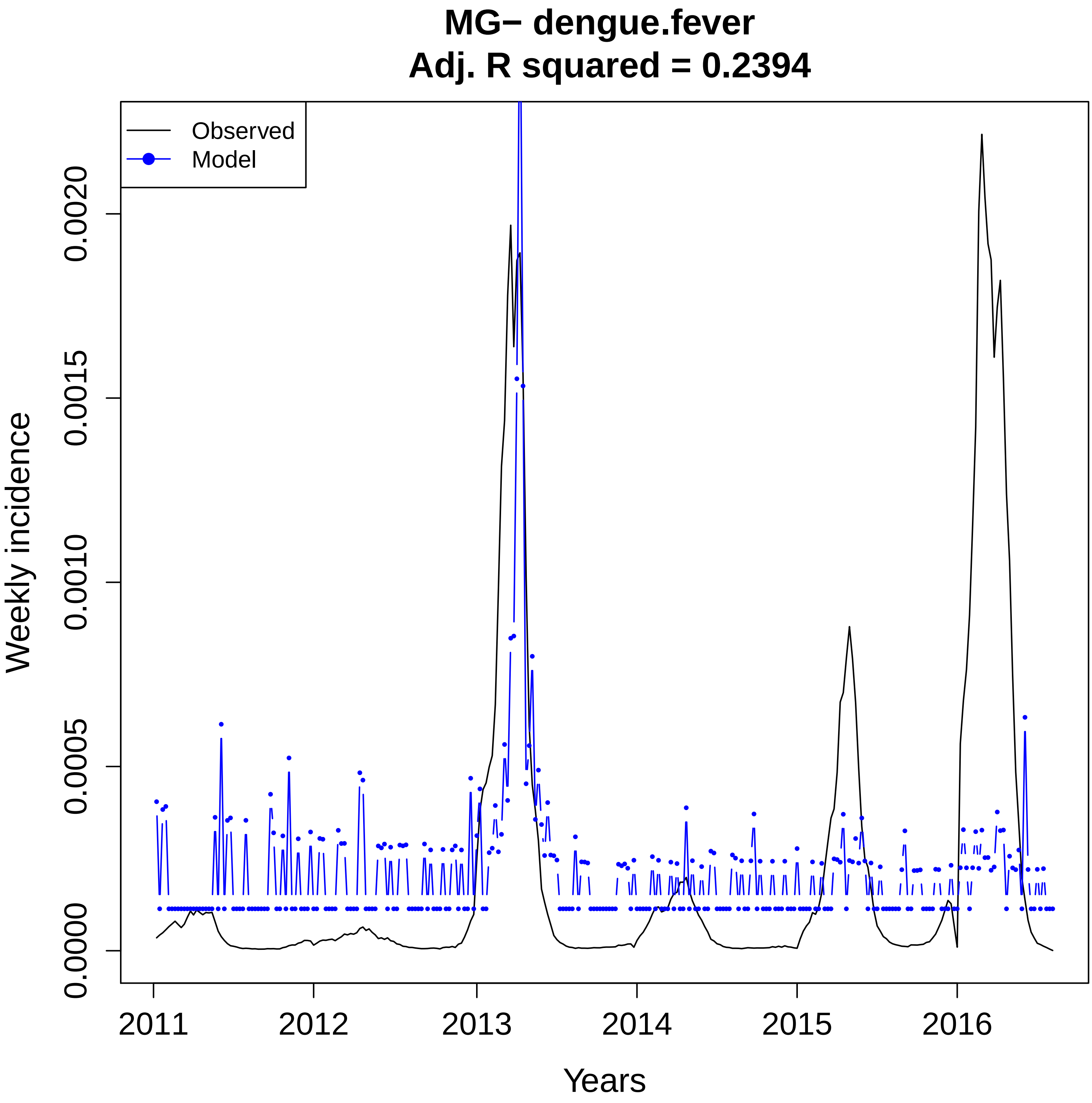

MG− dengue.fever
Adj. R squared = 0.2394
Observed
Model
Weekly incidence
0.0000
0.0005
0.0010
0.0015
0.0020
2011
2012
2013
2014
2015
2016
Years

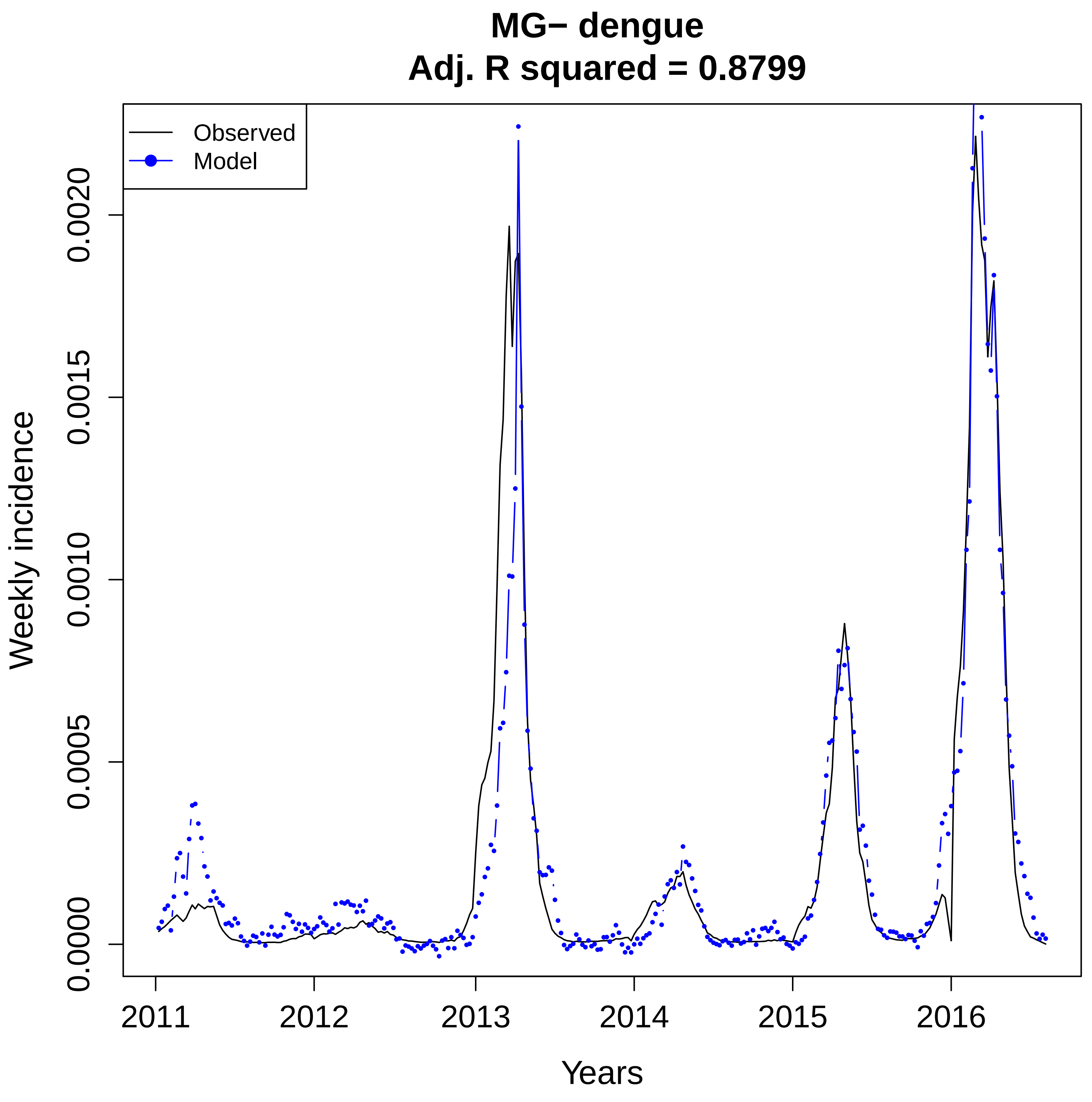

MG− dengue
Adj. R squared = 0.8799
Observed
Model
Weekly incidence
0.0000
0.0005
0.0010
0.0015
0.0020
2011
2012
2013
2014
2015
2016
Years

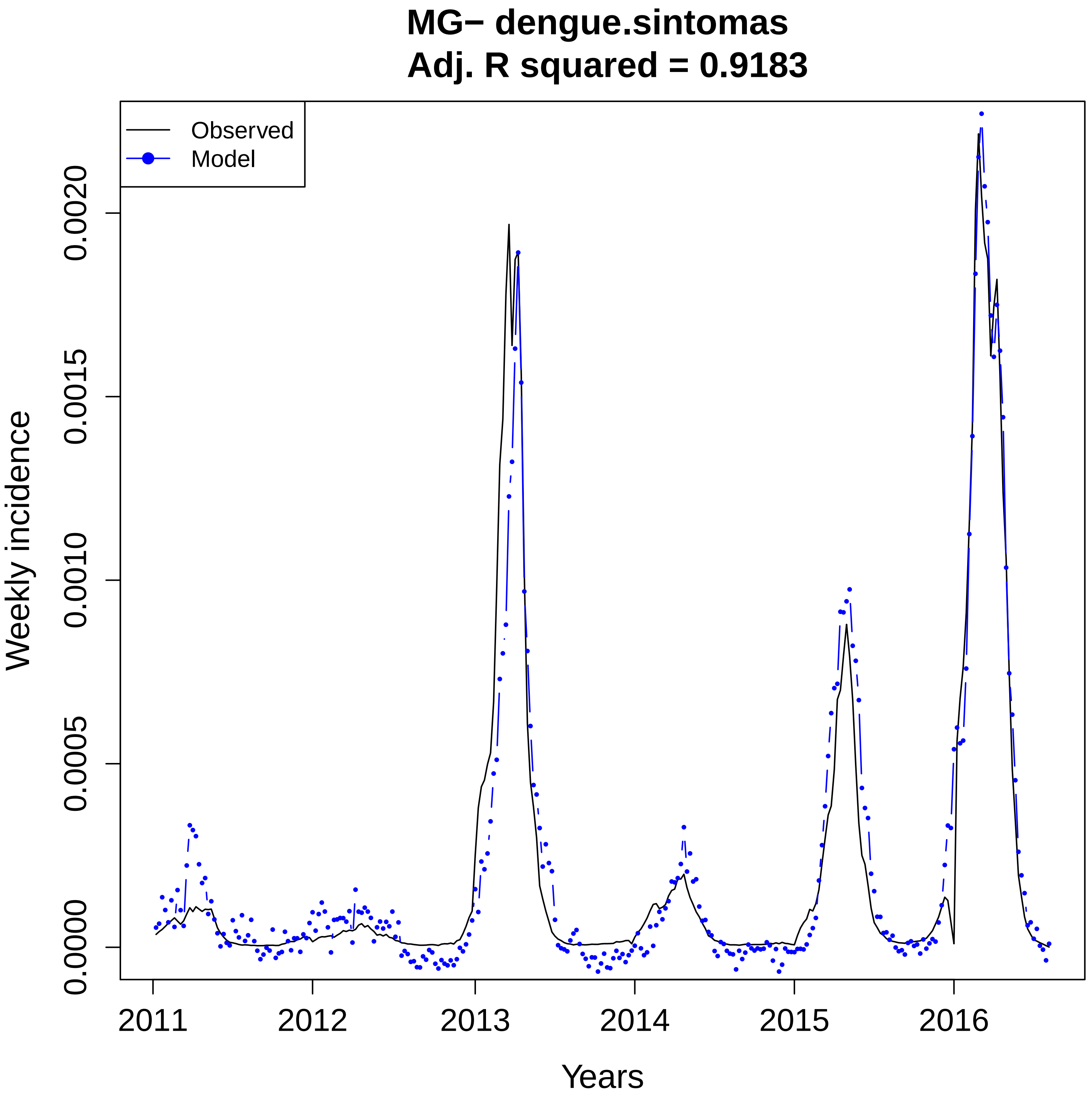

MG− dengue.sintomas
Adj. R squared = 0.9183
Observed
Model
Weekly incidence
0.0000
0.0005
0.0010
0.0015
0.0020
2011
2012
2013
2014
2015
2016
Years

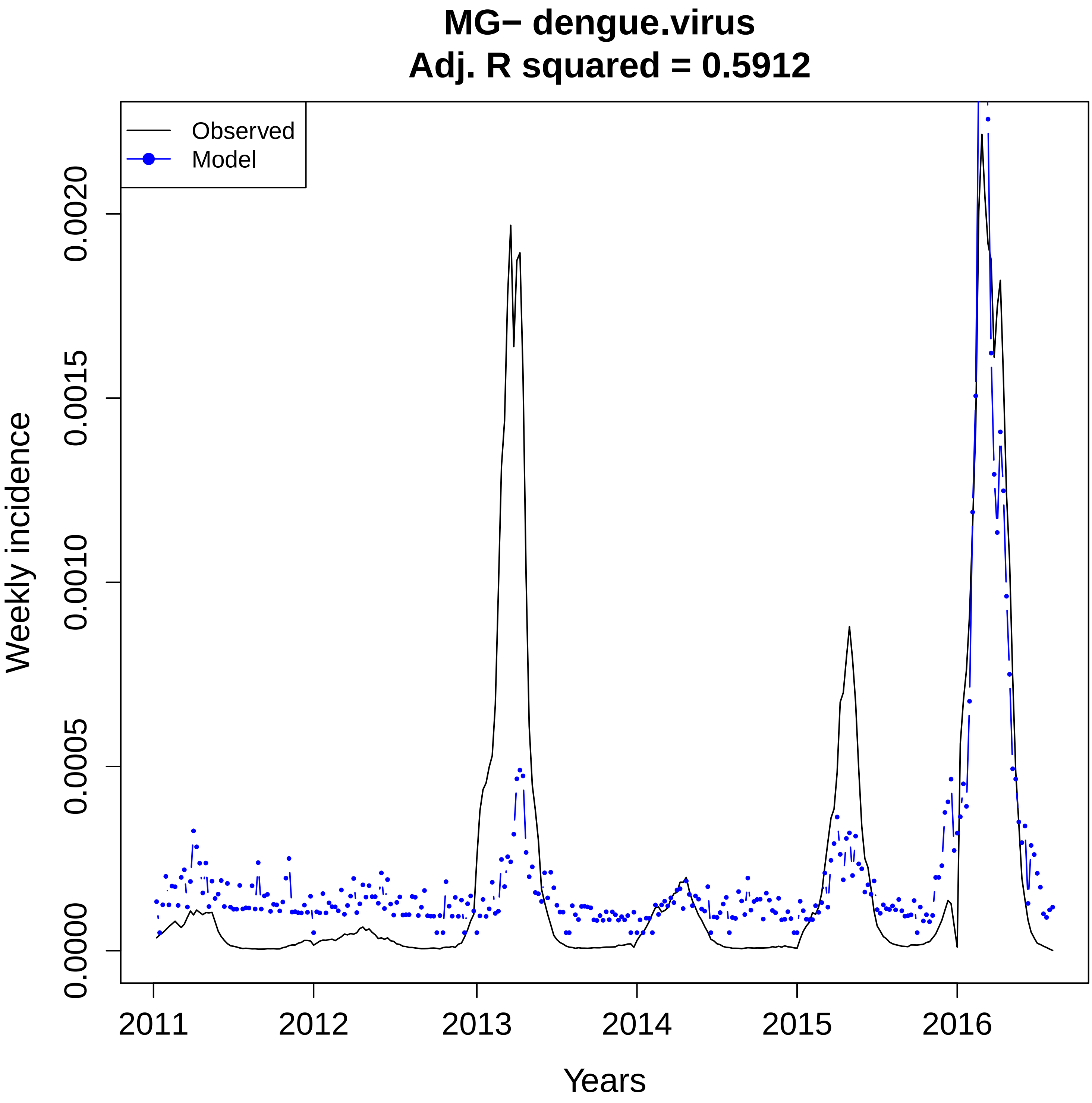

MG− dengue.virus
Adj. R squared = 0.5912
Observed
Model
Weekly incidence
0.0000
0.0005
0.0010
0.0015
0.0020
2011
2012
2013
2014
2015
2016
Years

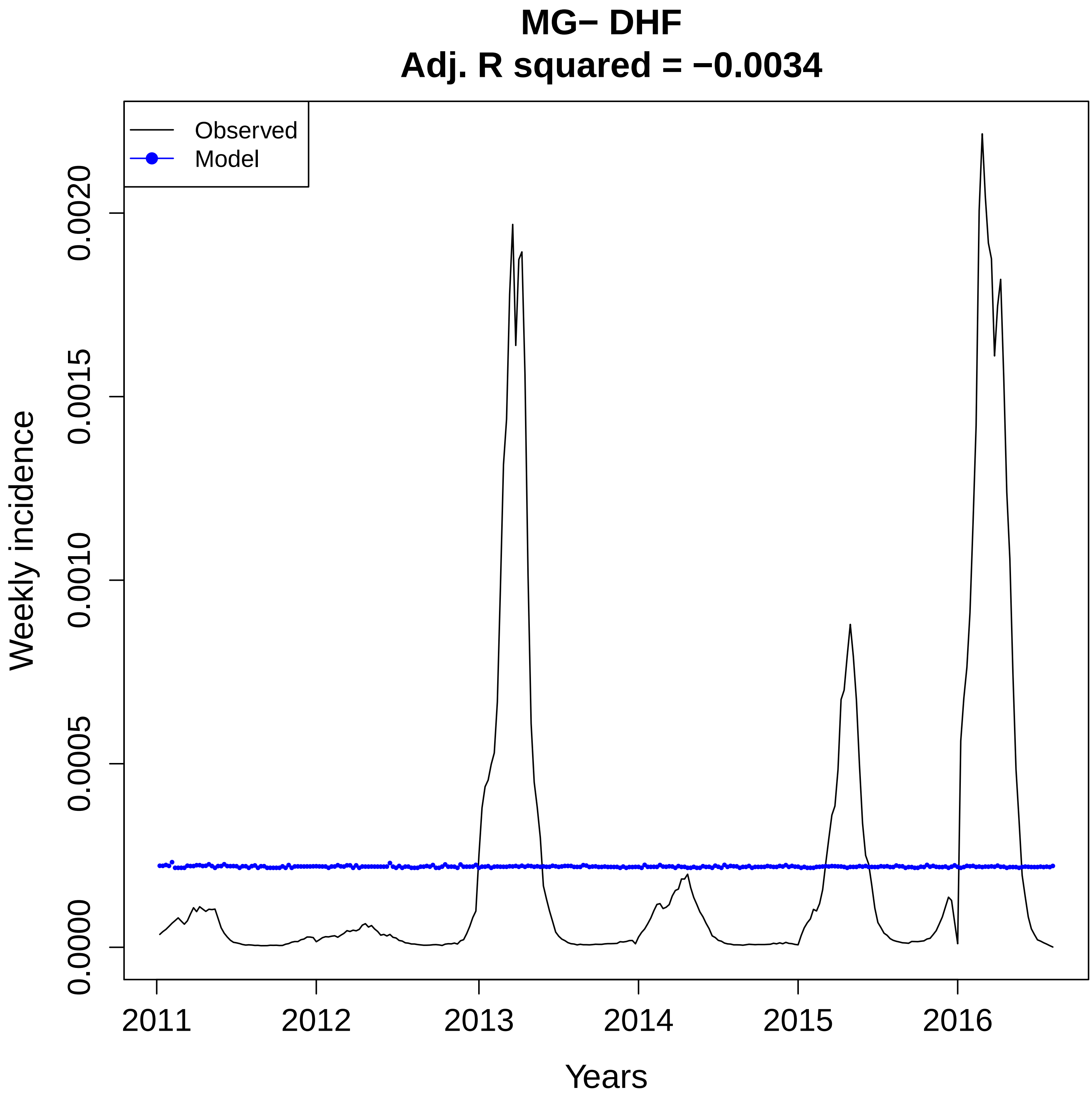
MG− DHF
Adj. R squared = −0.0034
Observed
Model
Weekly incidence
0.0000
0.0005
0.0010
0.0015
0.0020
2011
2012
2013
2014
2015
2016
Years

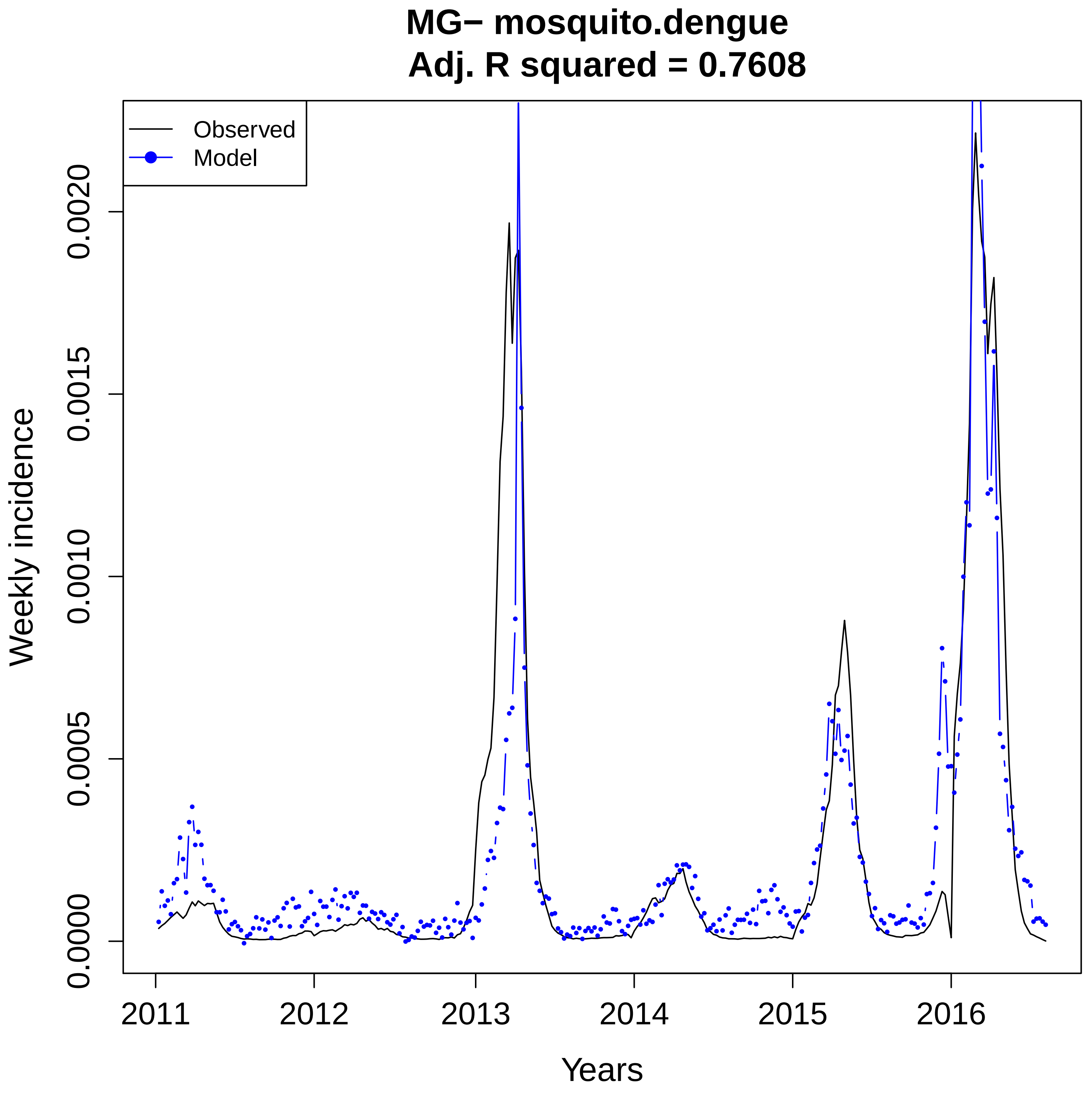

MG− mosquito.dengue
Adj. R squared = 0.7608
Observed
Model
Weekly incidence
0.0000
0.0005
0.0010
0.0015
0.0020
2011
2012
2013
2014
2015
2016
Years

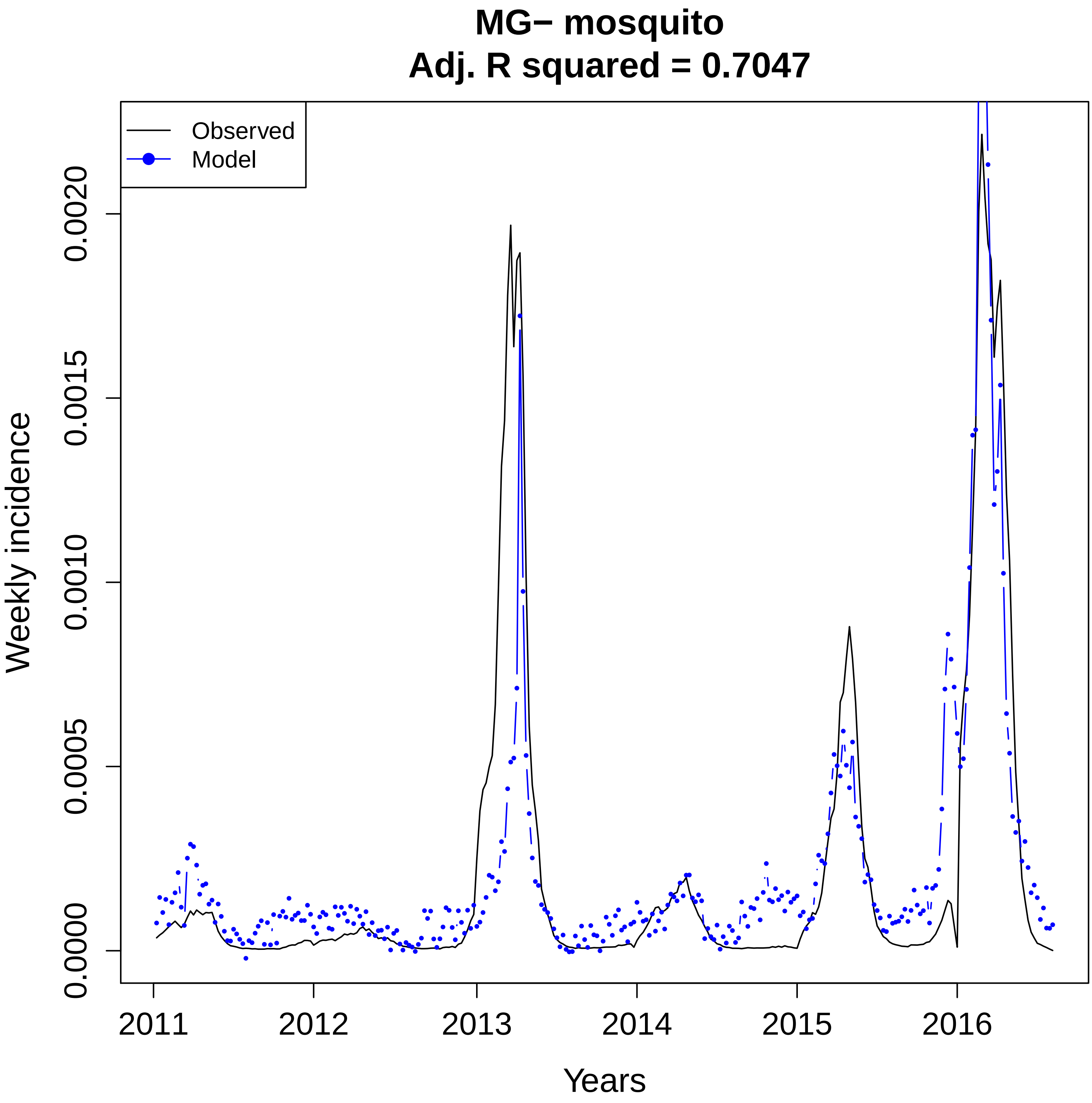

MG− mosquito
Adj. R squared = 0.7047
Observed
Model
Weekly incidence
0.0000
0.0005
0.0010
0.0015
0.0020
2011
2012
2013
2014
2015
2016
Years

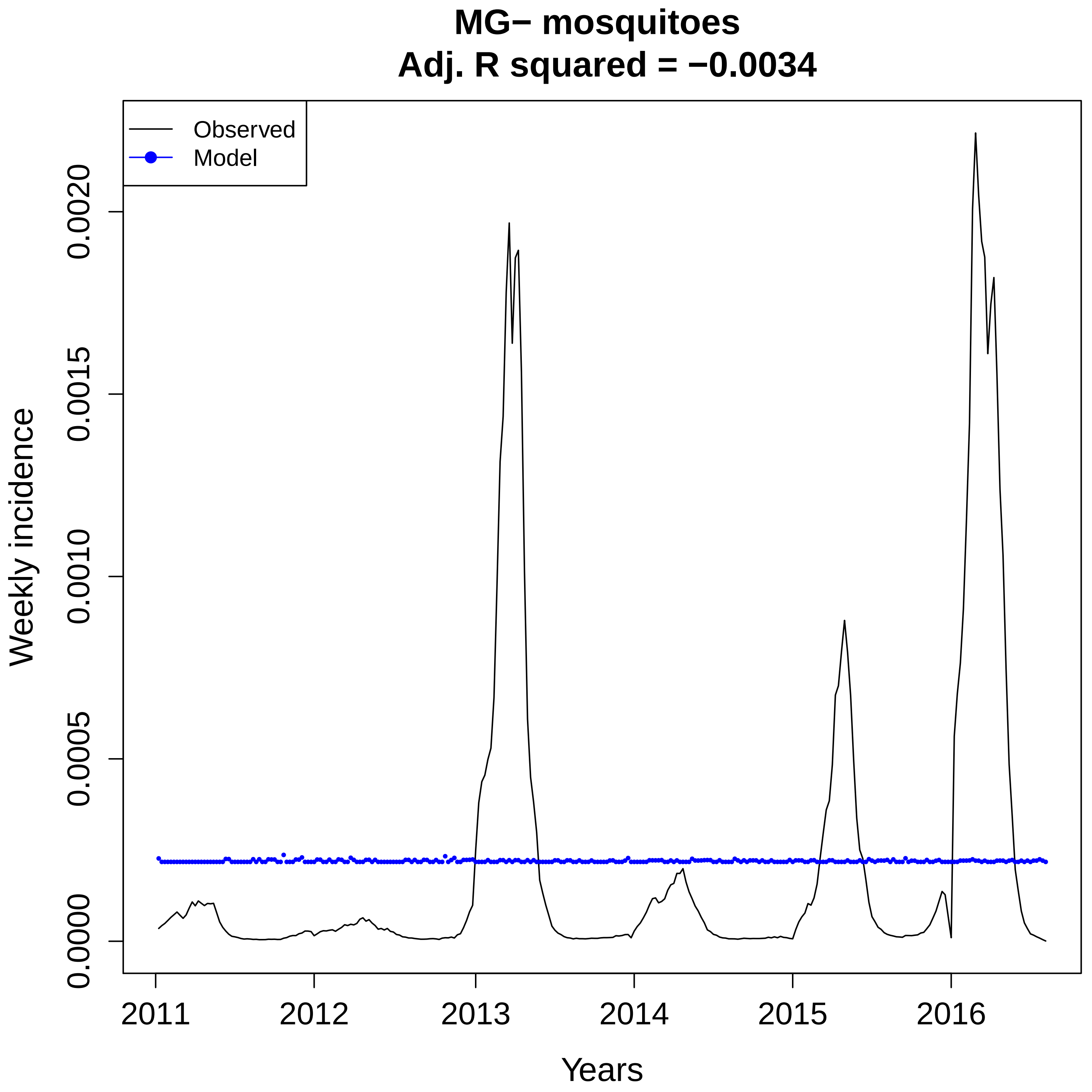

MG− mosquitoes
Adj. R squared = −0.0034
Observed
Model
Weekly incidence
0.0000
0.0005
0.0010
0.0015
0.0020
2011
2012
2013
2014
2015
2016
Years

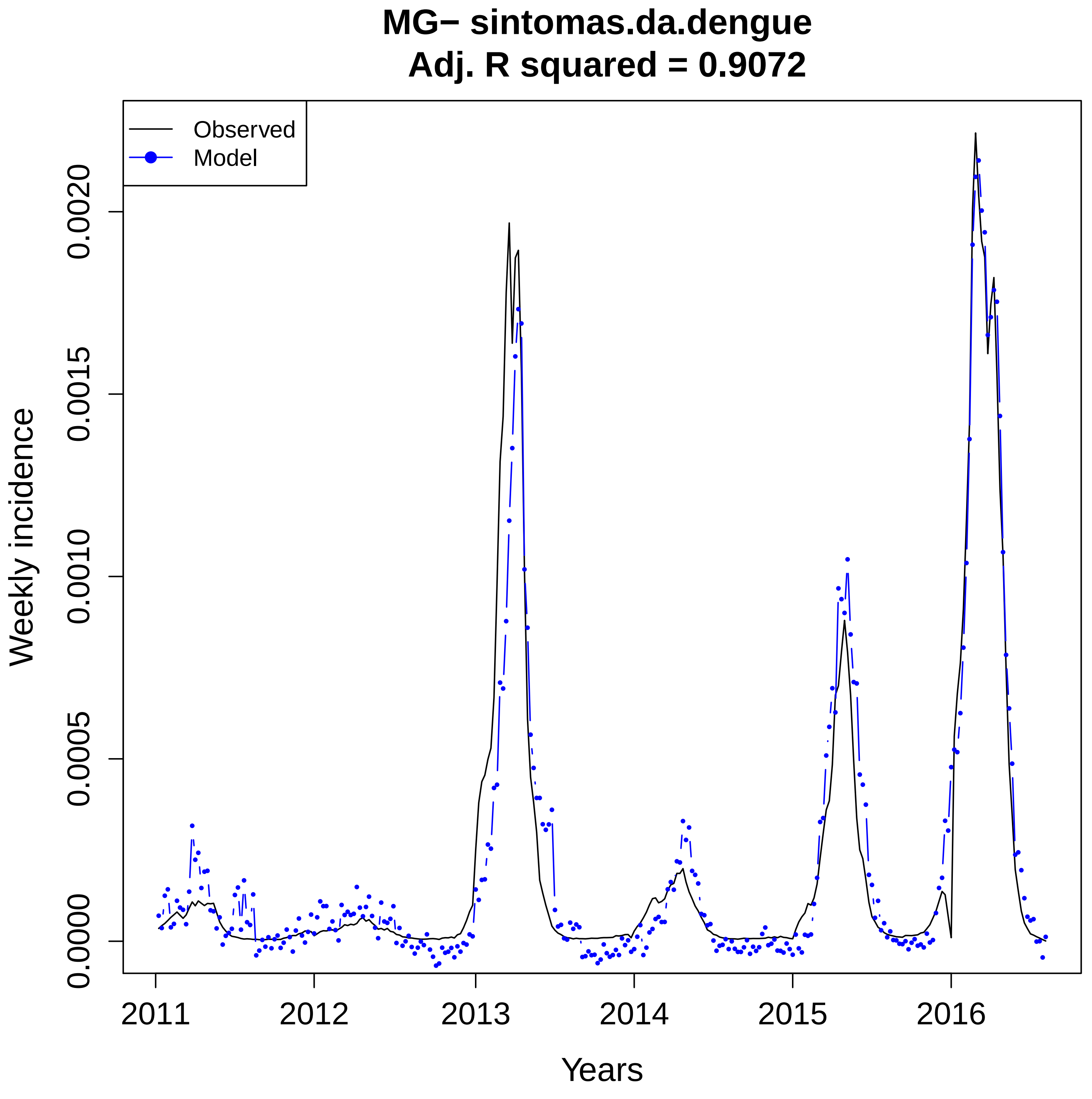
MG− sintomas.da.dengue
Adj. R squared = 0.9072
Observed
Model
Weekly incidence
0.0000
0.0005
0.0010
0.0015
0.0020
2011
2012
2013
2014
2015
2016
Years

Mato Grosso do Sul (MS)

Dengue incidence vs. Google Health Trends (2011–2016)

Definitions:

- **Combined terms:**
  - **All:** model with all the available terms. n= number of terms
  - **Four:** model with "dengue", "dengue sintomas", "aedes", and "mosquito".
  - **Uncorrelated:** model with non-correlated terms. n = number of uncorrelated terms
- **Individual terms:** Plots developed with one term at a time.

- Terms for all and uncorrelated models can be found in additional file 3.

Combined terms
Adjusted R squared

Mato Grosso do Sul (MS)

All
(n = 9)

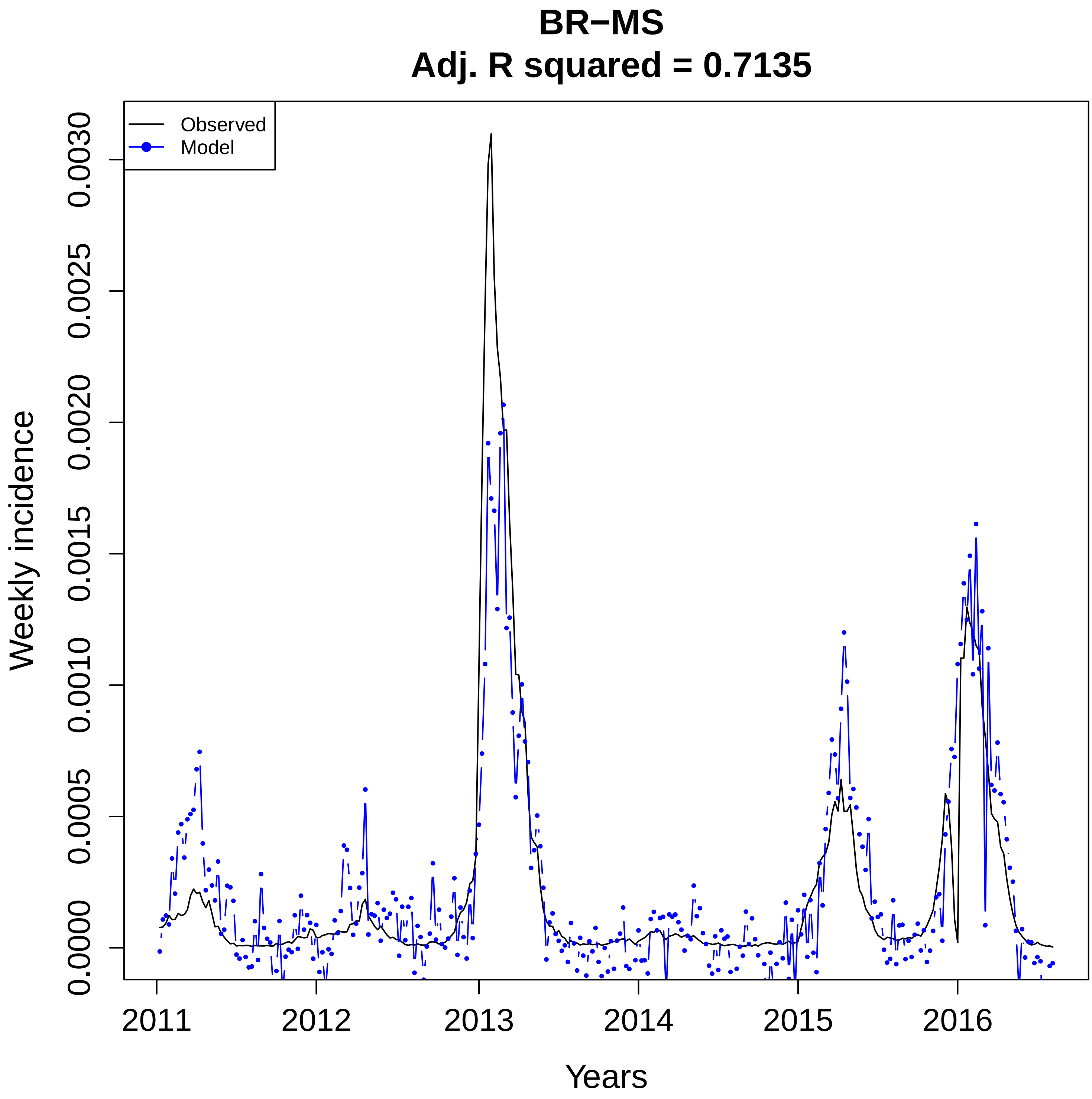

BR−MS
Adj. R squared = 0.7135
Observed
Model
Weekly incidence
0.0000
0.0005
0.0010
0.0015
0.0020
0.0025
0.0030
2011
2012
2013
2014
2015
2016
Years

Four

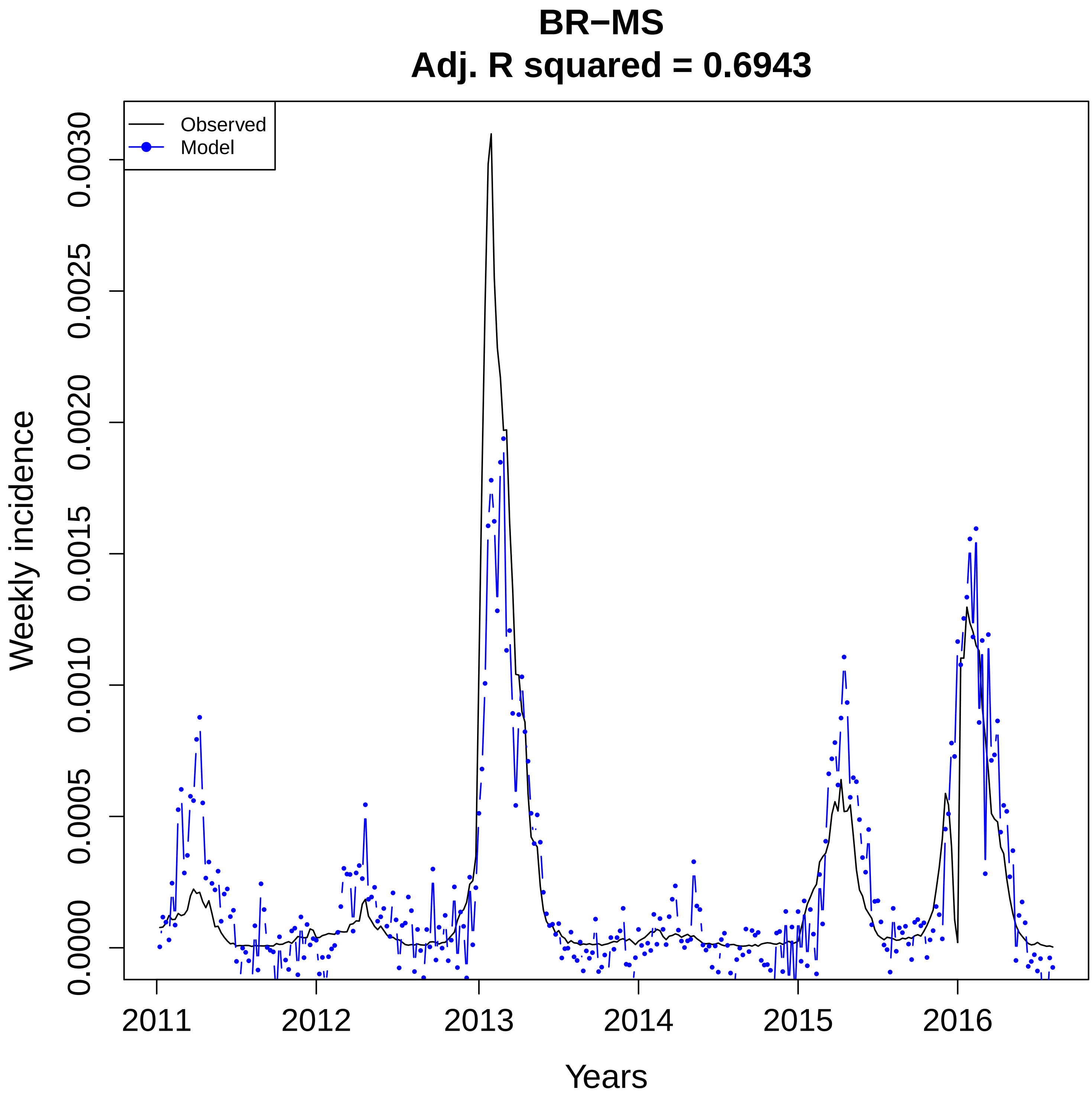

BR−MS
Adj. R squared = 0.6943
Observed
Model
Weekly incidence
0.0000
0.0005
0.0010
0.0015
0.0020
0.0025
0.0030
2011
2012
2013
2014
2015
2016
Years

Uncorrelated
(n = 3)

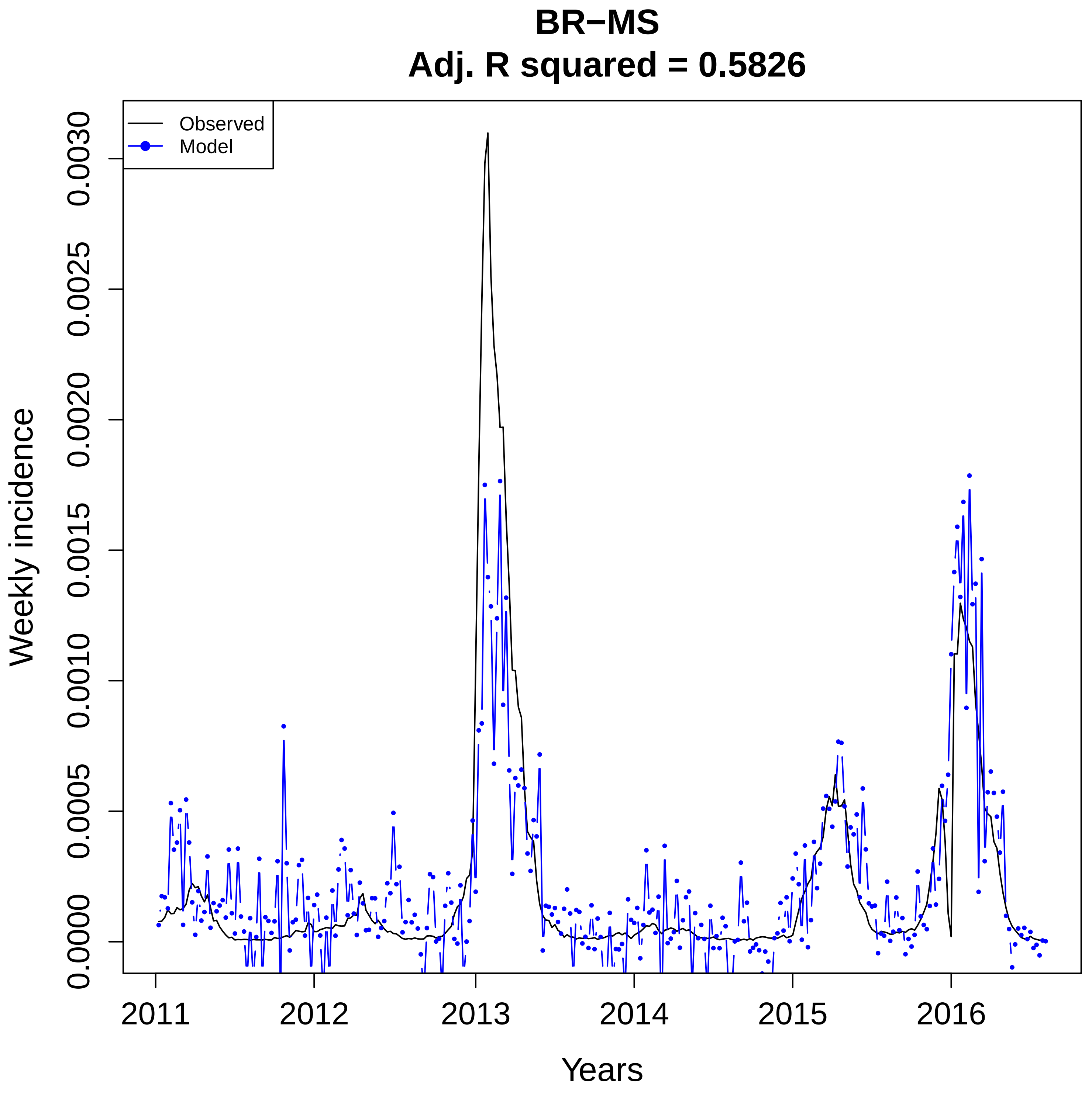

Individual terms
Adjusted R squared

Mato Grosso do Sul
(MS)

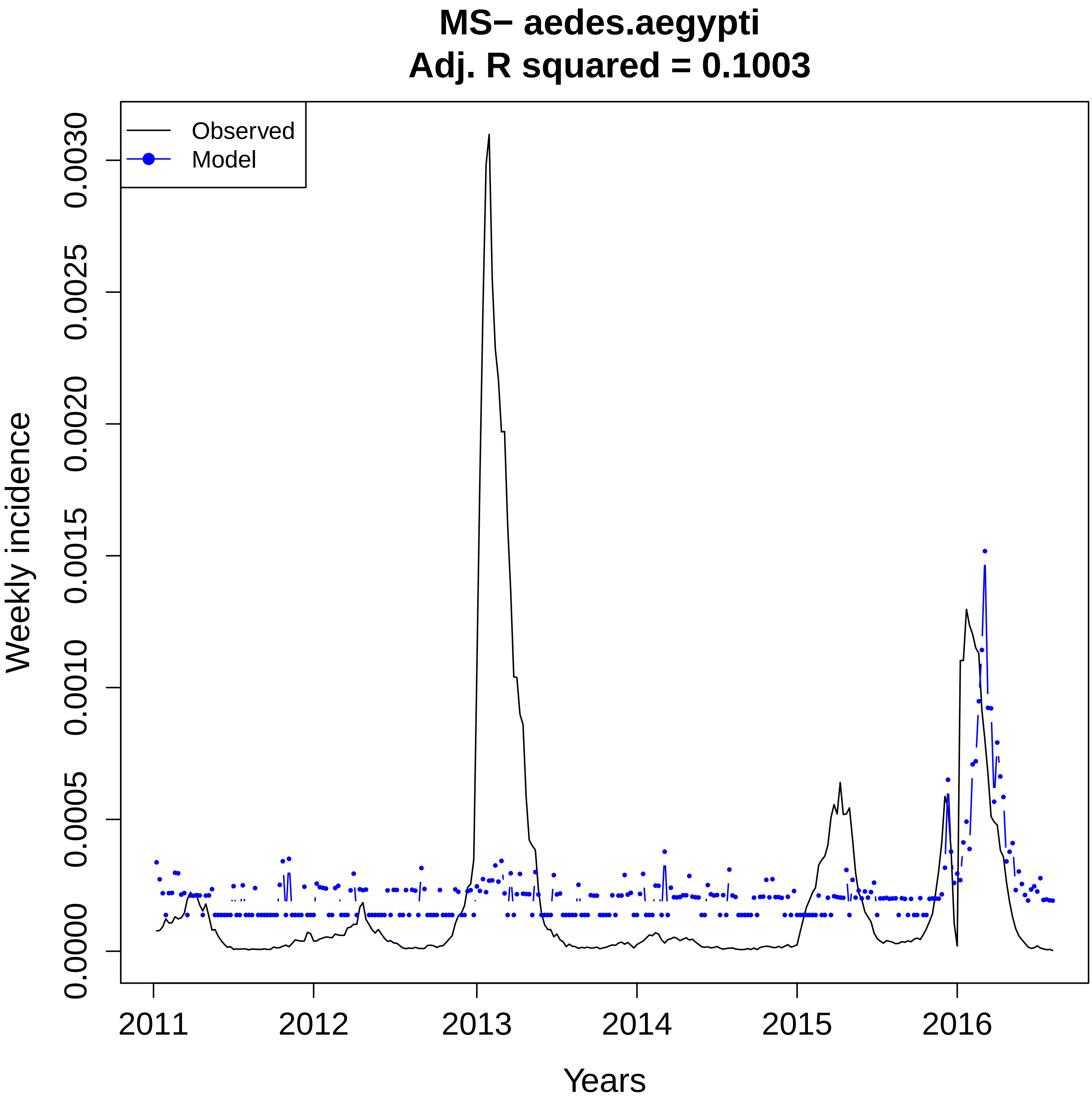
MS− aedes.aegypti
Adj. R squared = 0.1003
Observed
Model
Weekly incidence
0.0000
0.0005
0.0010
0.0015
0.0020
0.0025
0.0030
2011
2012
2013
2014
2015
2016
Years

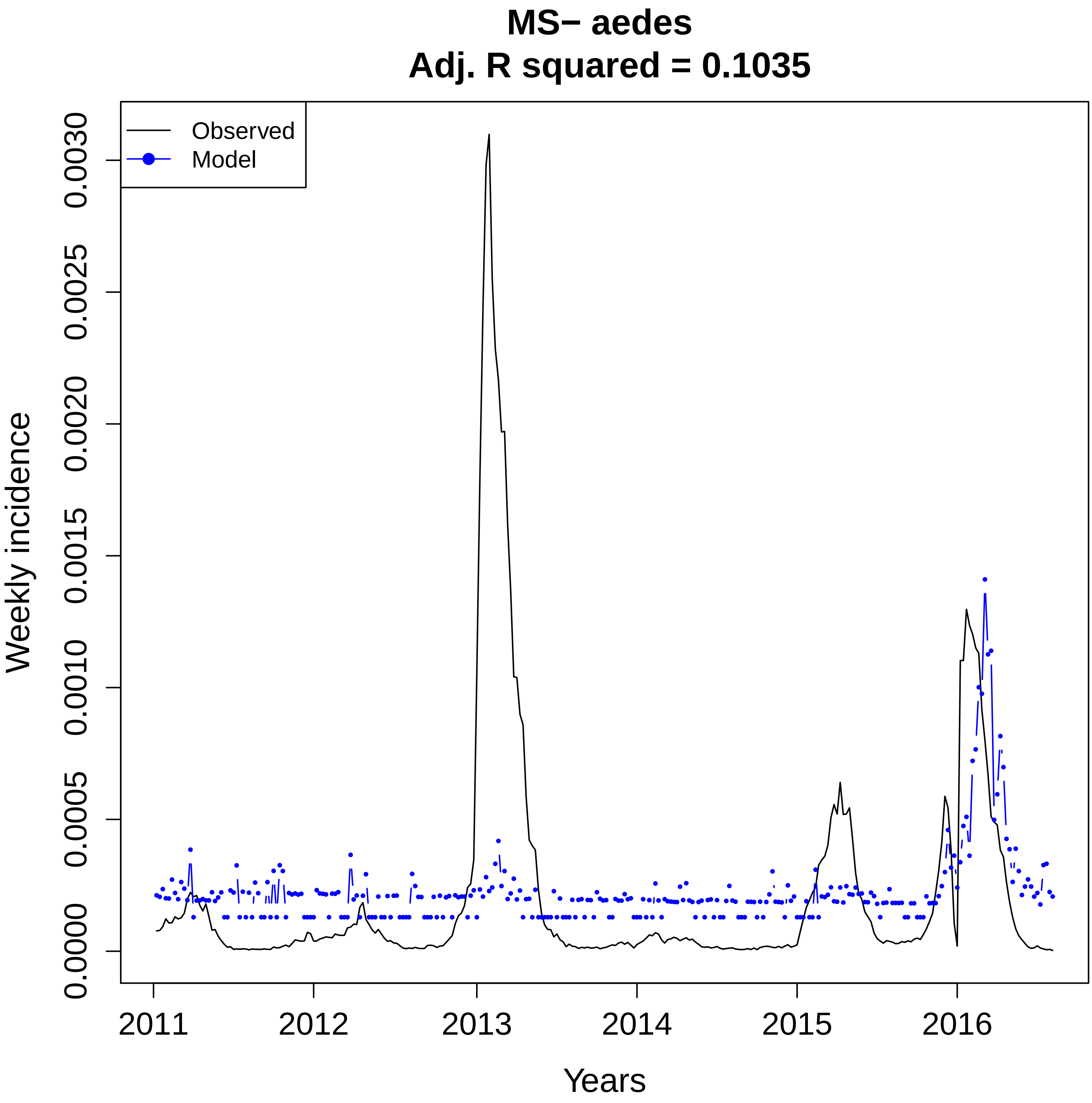

MS− aedes
Adj. R squared = 0.1035
Observed
Model
Weekly incidence
0.0000
0.0005
0.0010
0.0015
0.0020
0.0025
0.0030
2011
2012
2013
2014
2015
2016
Years

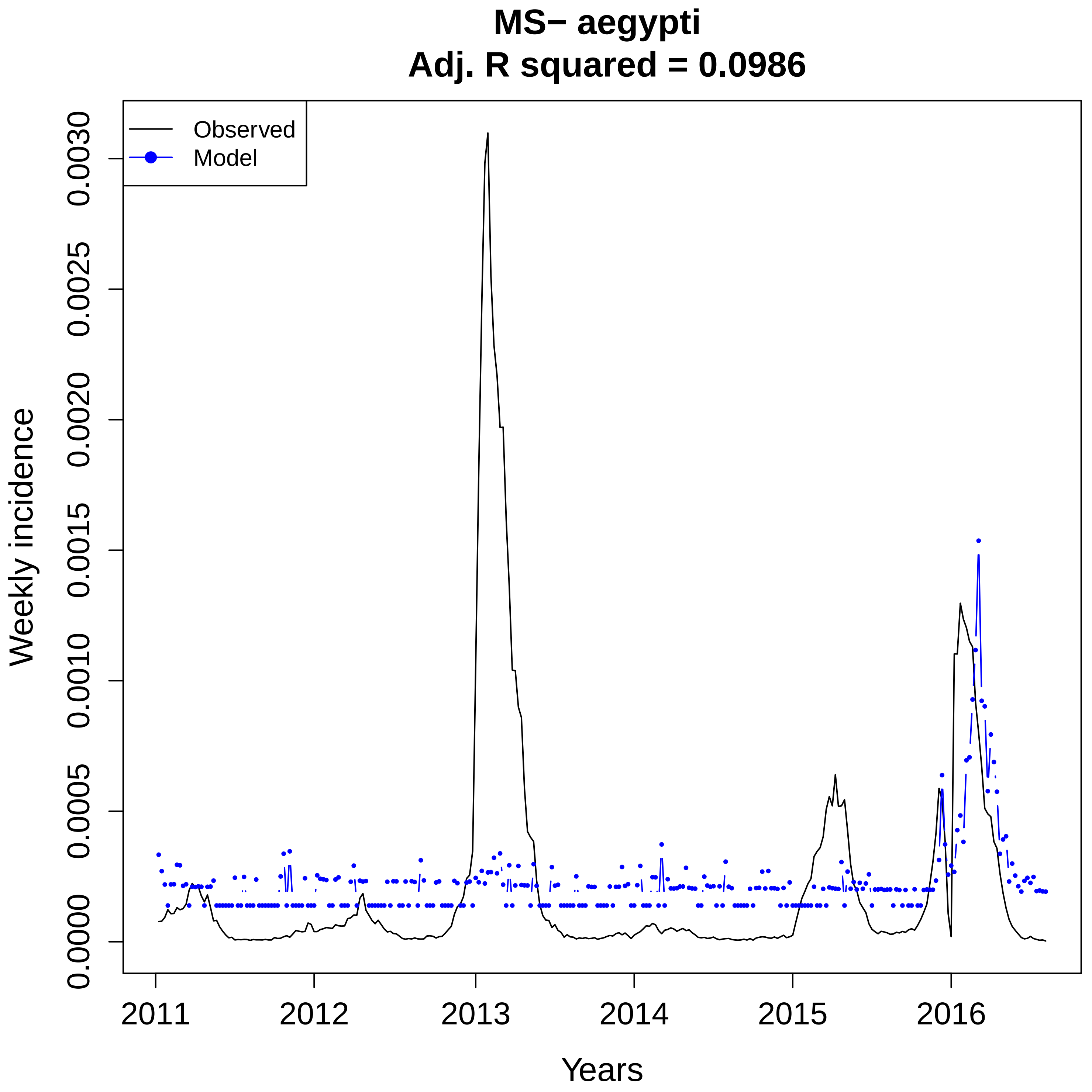

MS− aegypti
Adj. R squared = 0.0986
Observed
Model
Weekly incidence
0.0000
0.0005
0.0010
0.0015
0.0020
0.0025
0.0030
2011
2012
2013
2014
2015
2016
Years

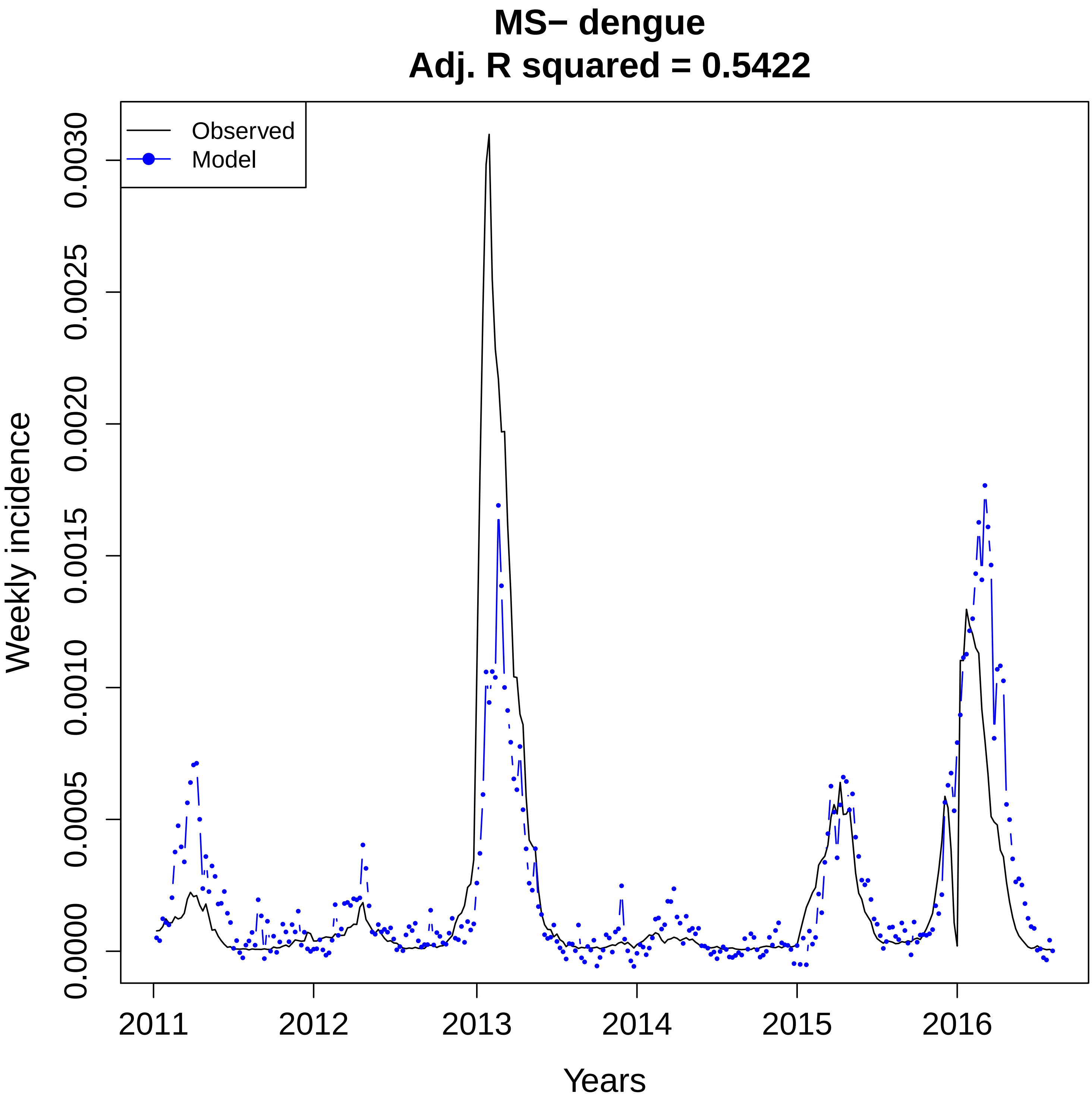

MS− dengue
Adj. R squared = 0.5422
Observed
Model
Weekly incidence
0.0000
0.0005
0.0010
0.0015
0.0020
0.0025
0.0030
2011
2012
2013
2014
2015
2016
Years

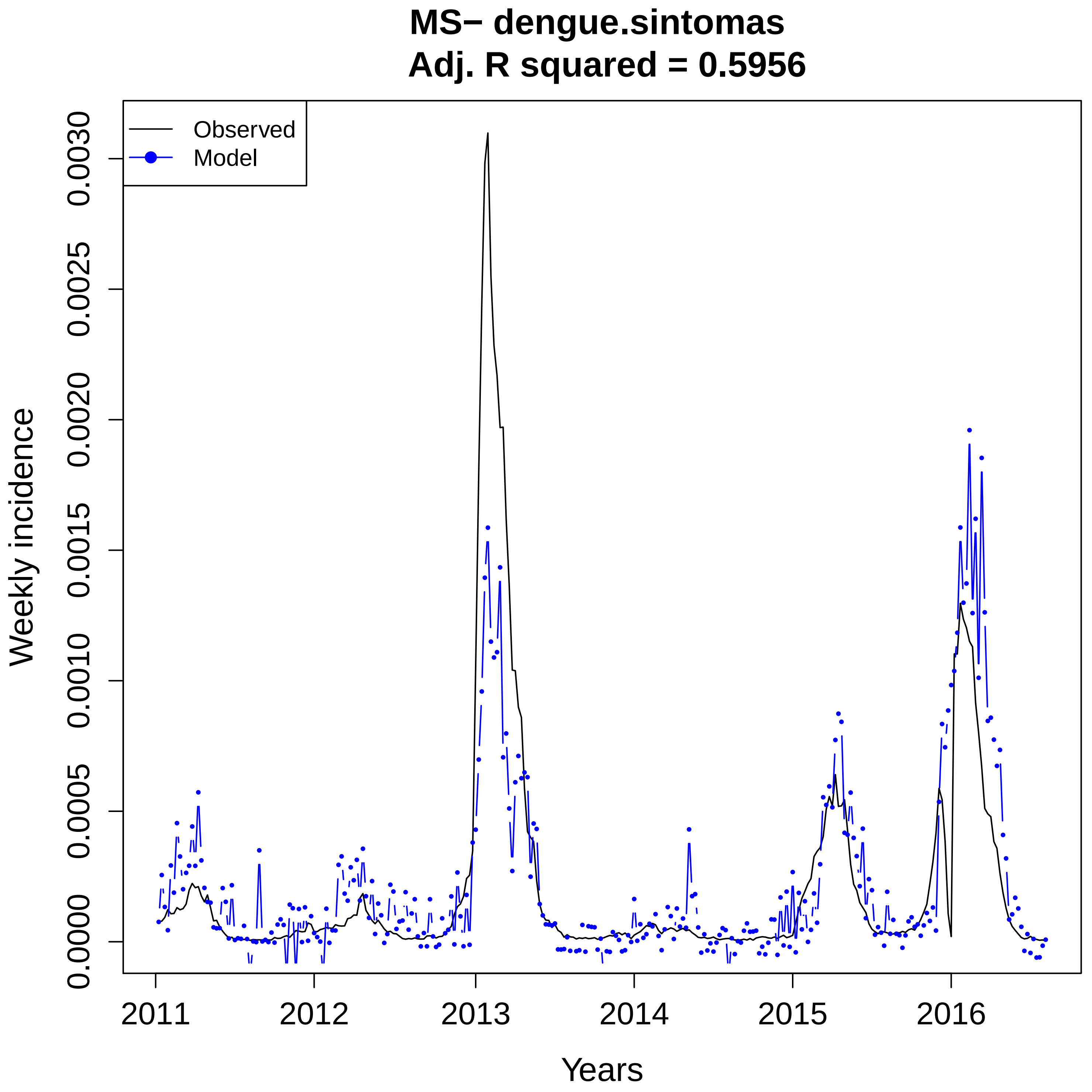
MS− dengue.sintomas
Adj. R squared = 0.5956
Observed
Model
Weekly incidence
0.0000
0.0005
0.0010
0.0015
0.0020
0.0025
0.0030
2011
2012
2013
2014
2015
2016
Years

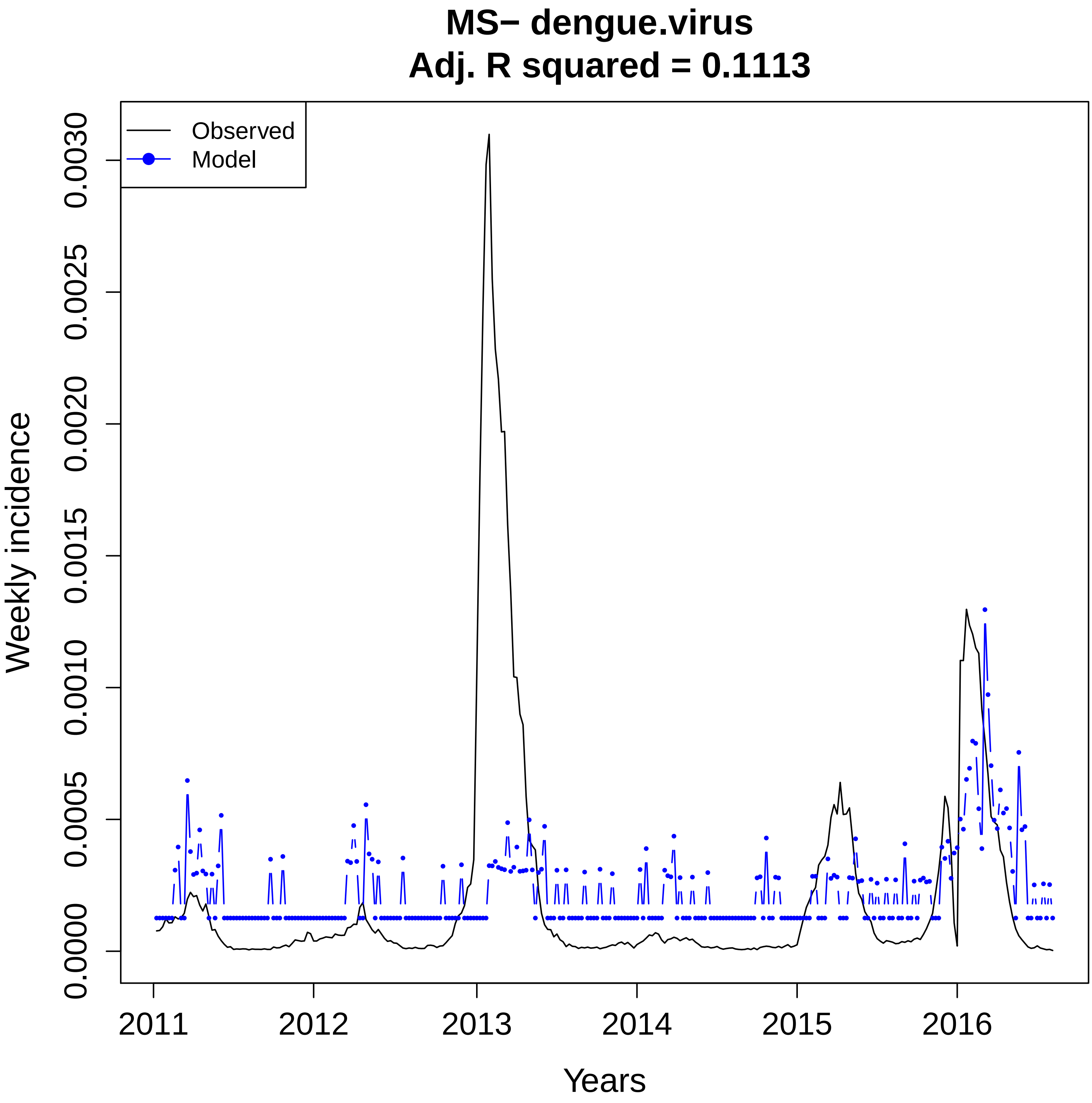

MS− dengue.virus
Adj. R squared = 0.1113
Observed
Model
Weekly incidence
0.0000
0.0005
0.0010
0.0015
0.0020
0.0025
0.0030
2011
2012
2013
2014
2015
2016
Years

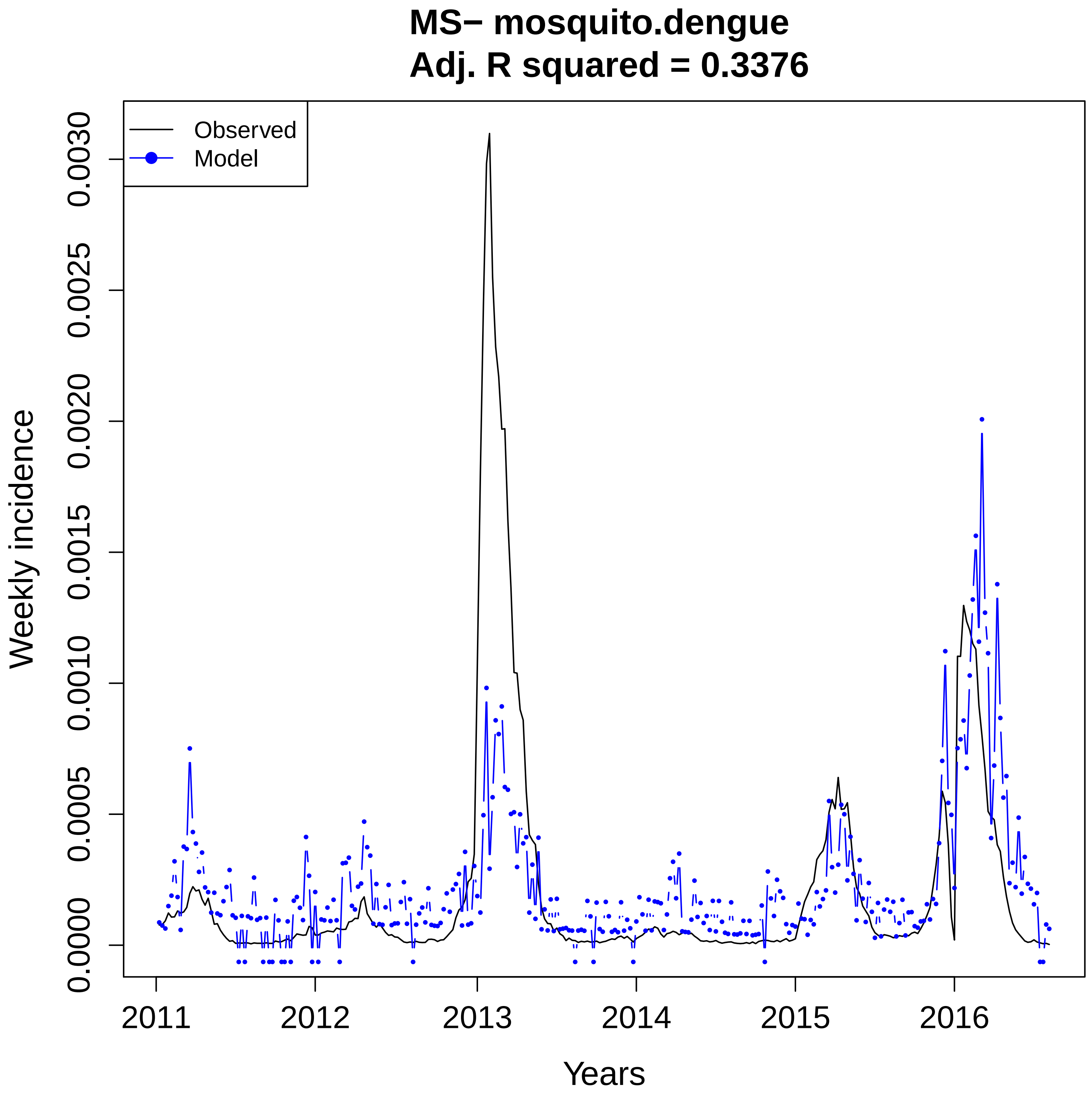
MS− mosquito.dengue
Adj. R squared = 0.3376
Observed
Model
Weekly incidence
0.0000
0.0005
0.0010
0.0015
0.0020
0.0025
0.0030
2011
2012
2013
2014
2015
2016
Years

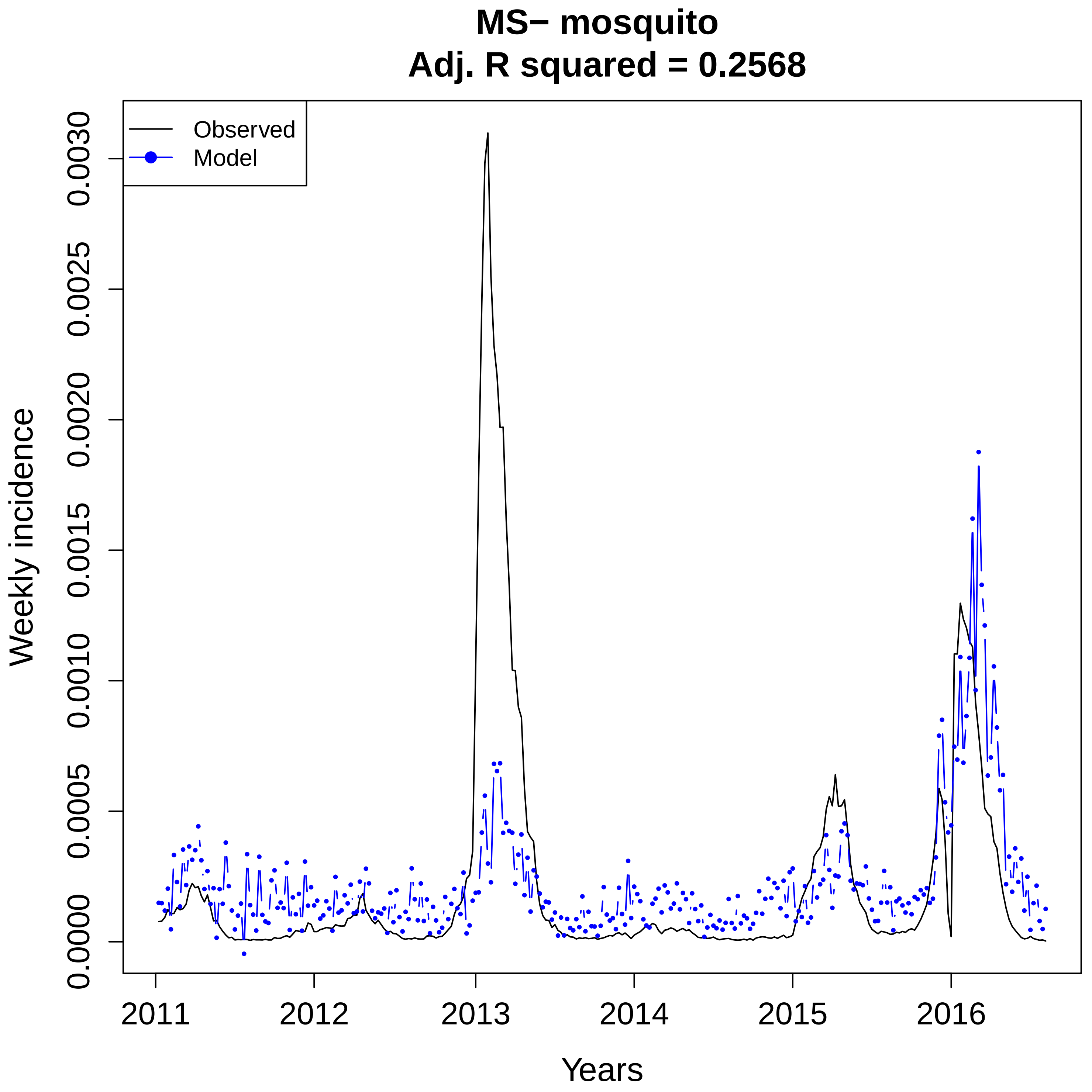
MS− mosquito
Adj. R squared = 0.2568
Observed
Model
Weekly incidence
0.0000
0.0005
0.0010
0.0015
0.0020
0.0025
0.0030
2011
2012
2013
2014
2015
2016
Years

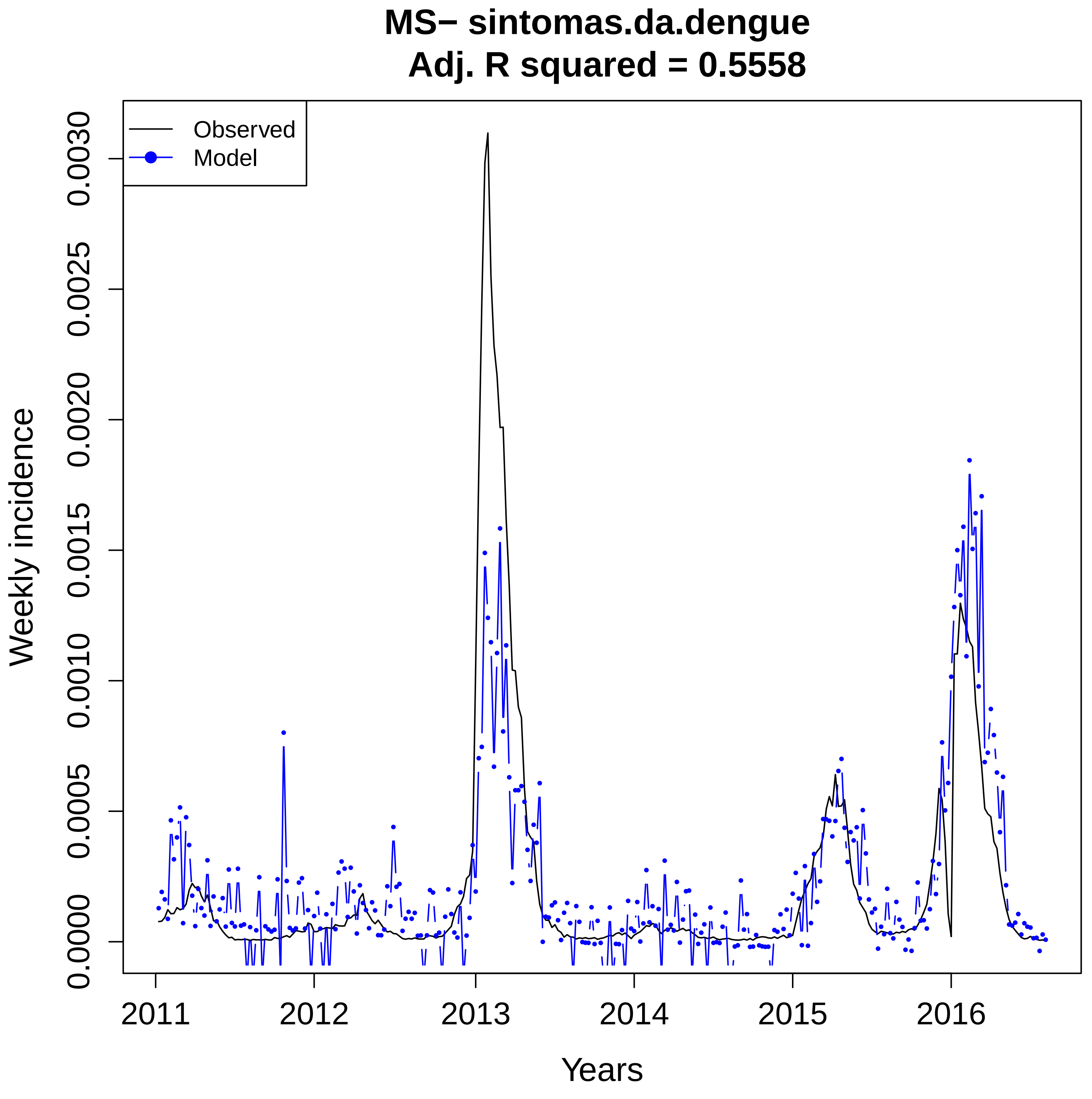
MS− sintomas.da.dengue
Adj. R squared = 0.5558
Observed
Model
Weekly incidence
0.0000
0.0005
0.0010
0.0015
0.0020
0.0025
0.0030
2011
2012
2013
2014
2015
2016
Years

Mato Grosso (MT)

Dengue incidence vs. Google Health Trends (2011–2016)

Definitions:

- **Combined terms:**
  - **All:** model with all the available terms. n= number of terms
  - **Four:** model with "dengue", "dengue sintomas", "aedes", and "mosquito".
  - **Uncorrelated:** model with non-correlated terms. n = number of uncorrelated terms
- **Individual terms:** Plots developed with one term at a time.

- Terms for all and uncorrelated models can be found in additional file 3.

Combined terms
Adjusted R squared

Mato Grosso (MT)

All
(n = 9)

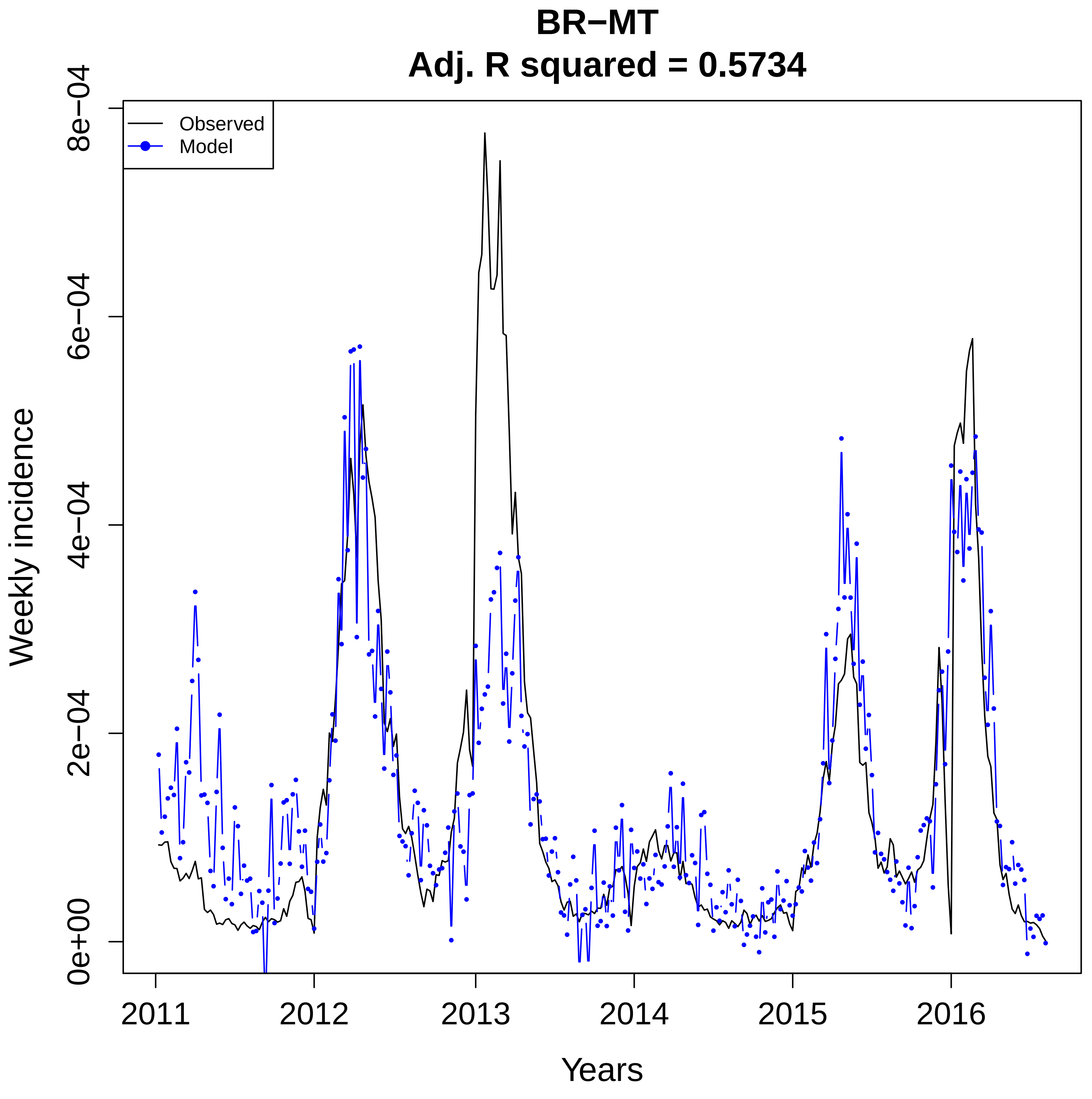
BR−MT
Adj. R squared = 0.5734
Observed
Model
Weekly incidence
0e+00
2e−04
4e−04
6e−04
8e−04
2011
2012
2013
2014
2015
2016
Years

Four

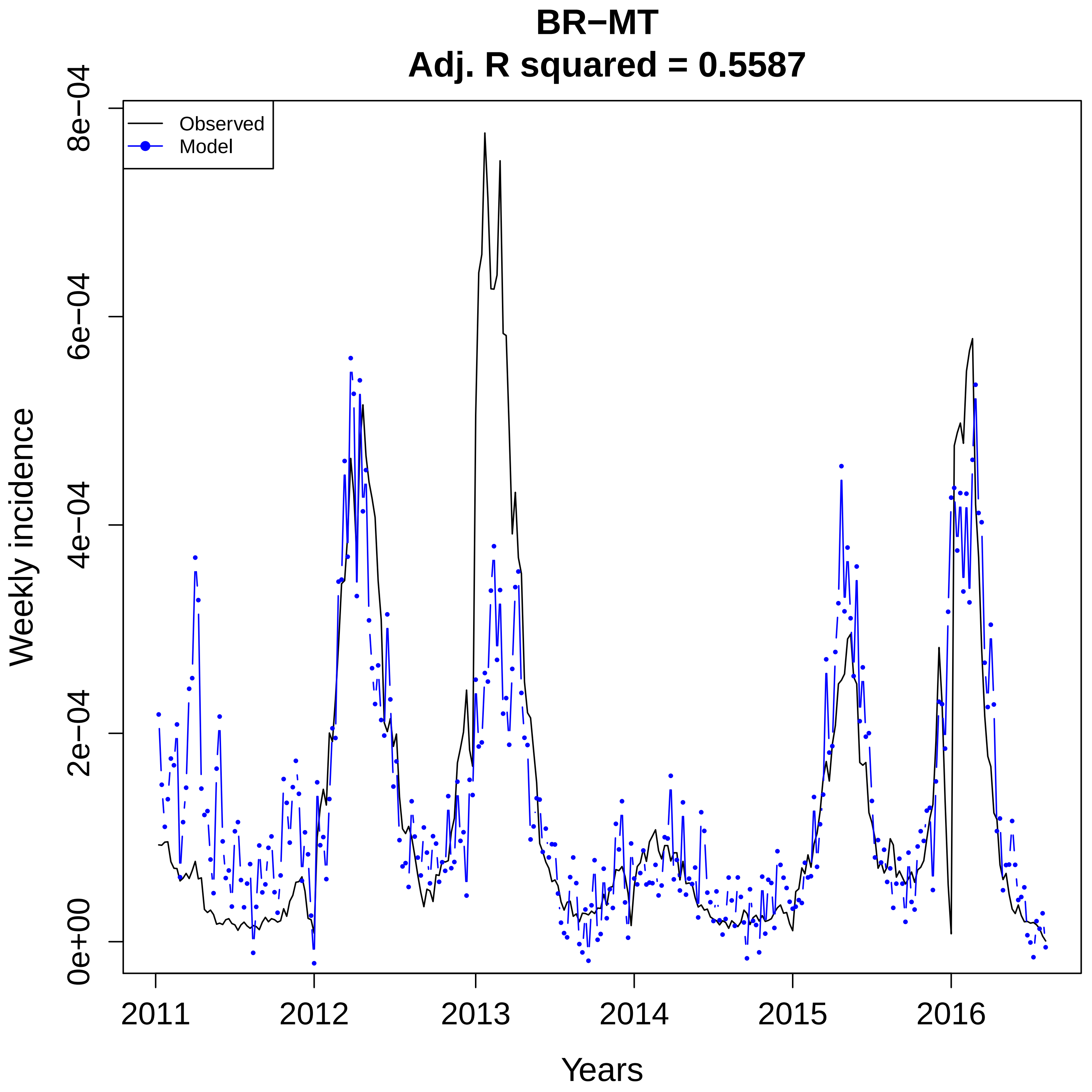
BR−MT
Adj. R squared = 0.5587
Observed
Model
Weekly incidence
0e+00
2e−04
4e−04
6e−04
8e−04
2011
2012
2013
2014
2015
2016
Years

Uncorrelated
(n = 3)

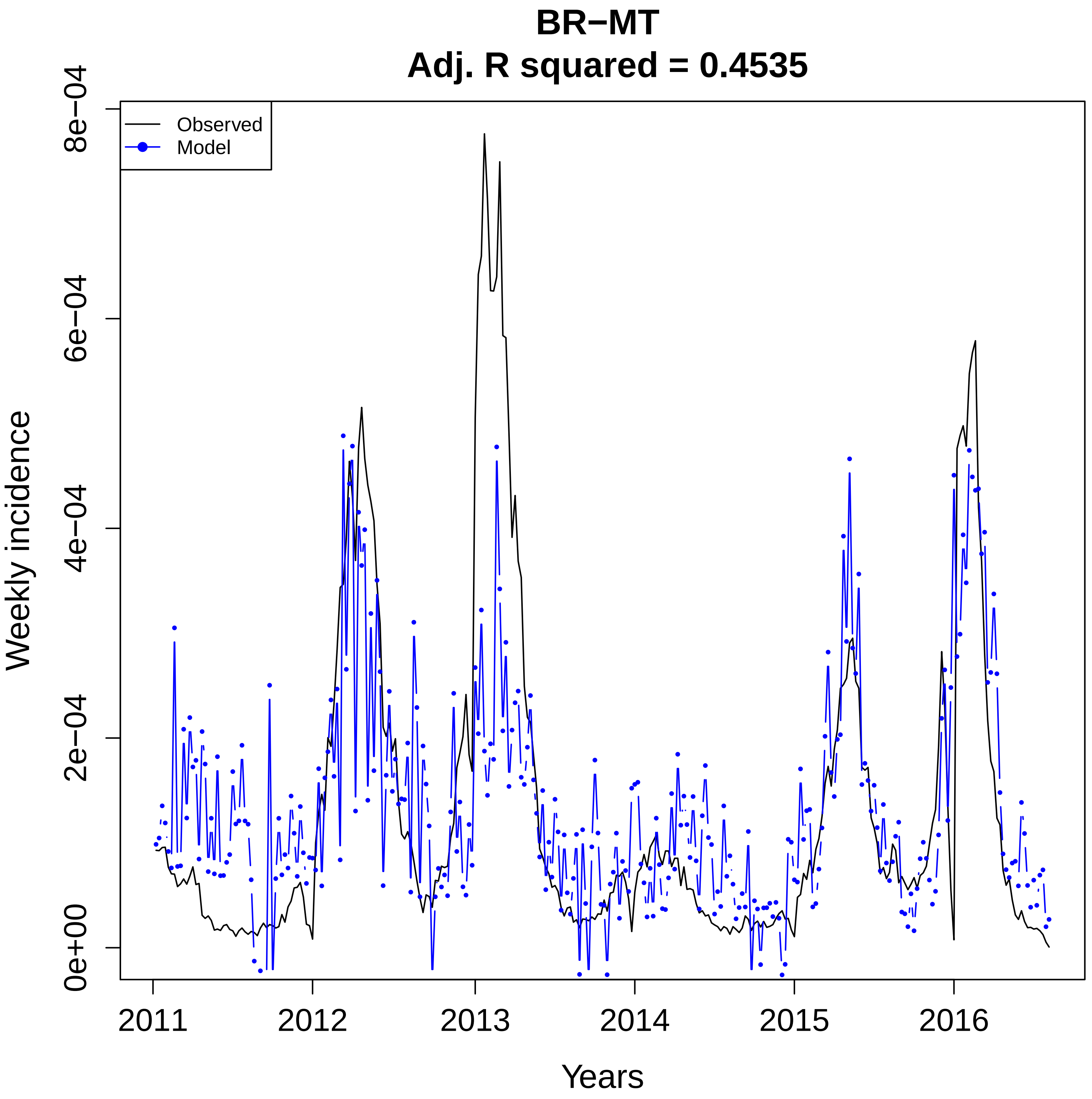

Individual terms
Adjusted R squared

Mato Grosso (MT)

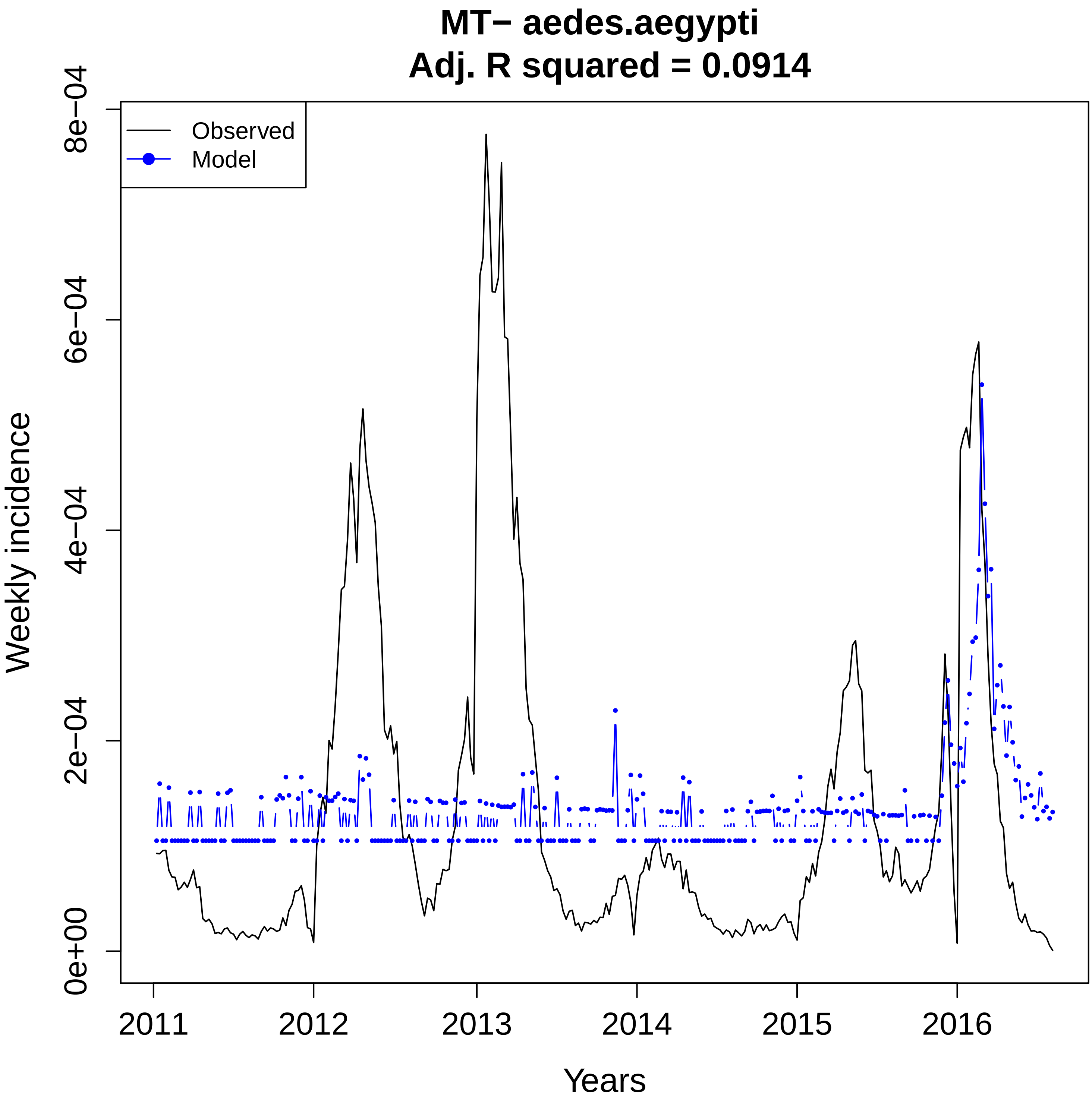
MT− aedes.aegypti
Adj. R squared = 0.0914
Observed
Model
Weekly incidence
0e+00
2e−04
4e−04
6e−04
8e−04
2011
2012
2013
2014
2015
2016
Years

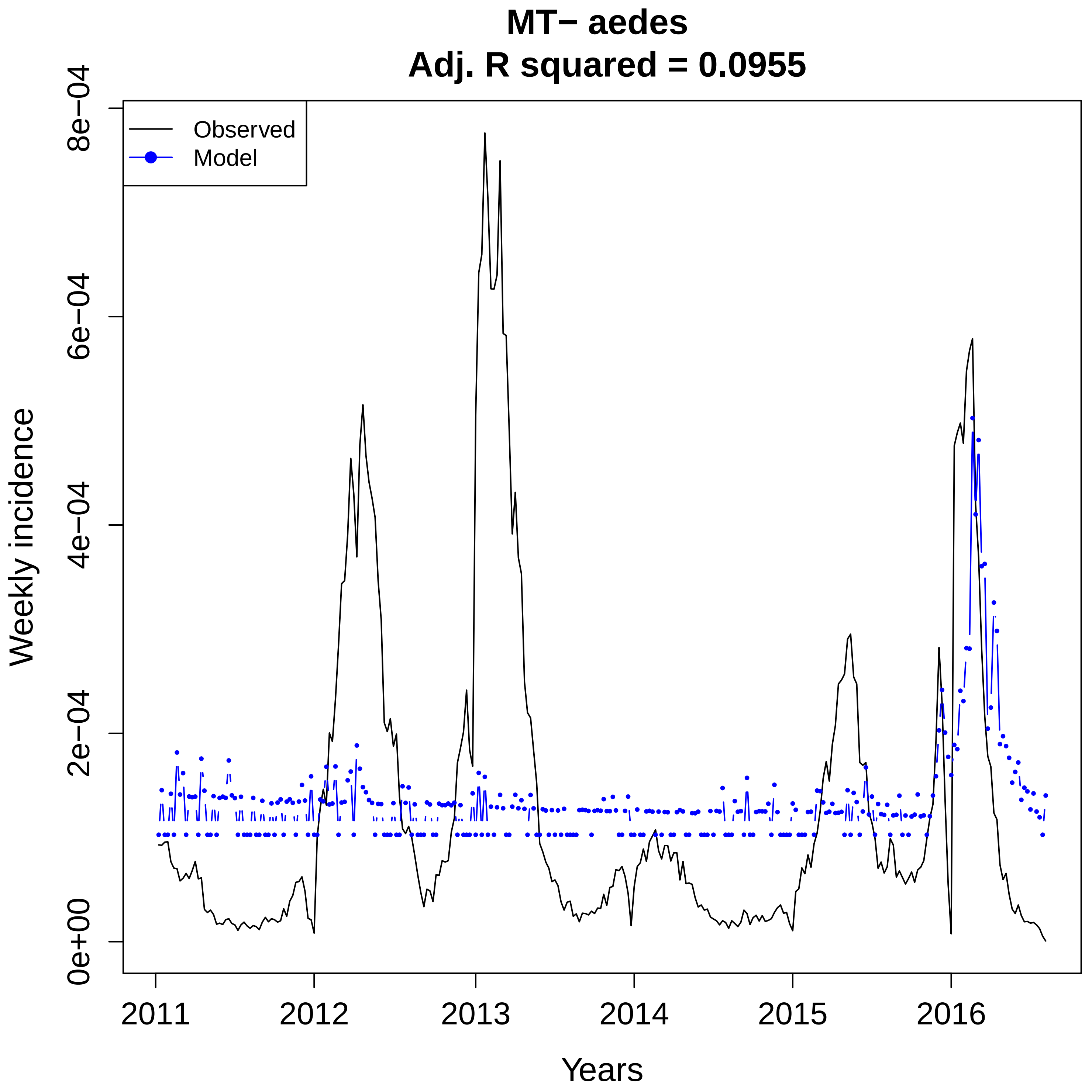

MT− aedes
Adj. R squared = 0.0955
Observed
Model
Weekly incidence
0e+00
2e−04
4e−04
6e−04
8e−04
2011
2012
2013
2014
2015
2016
Years

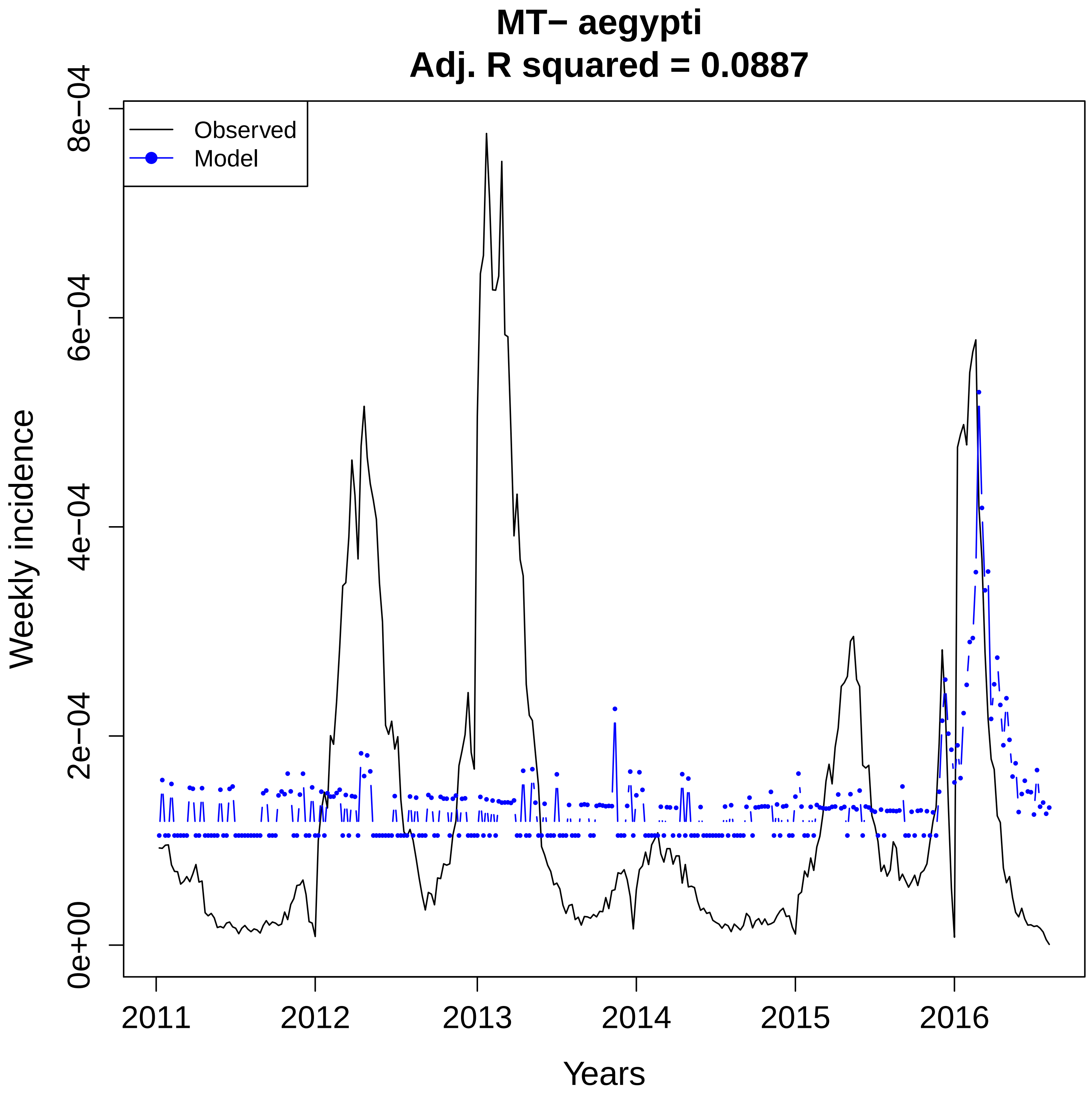
MT− aegypti
Adj. R squared = 0.0887
Observed
Model
Weekly incidence
0e+00
2e−04
4e−04
6e−04
8e−04
Years
2011
2012
2013
2014
2015
2016

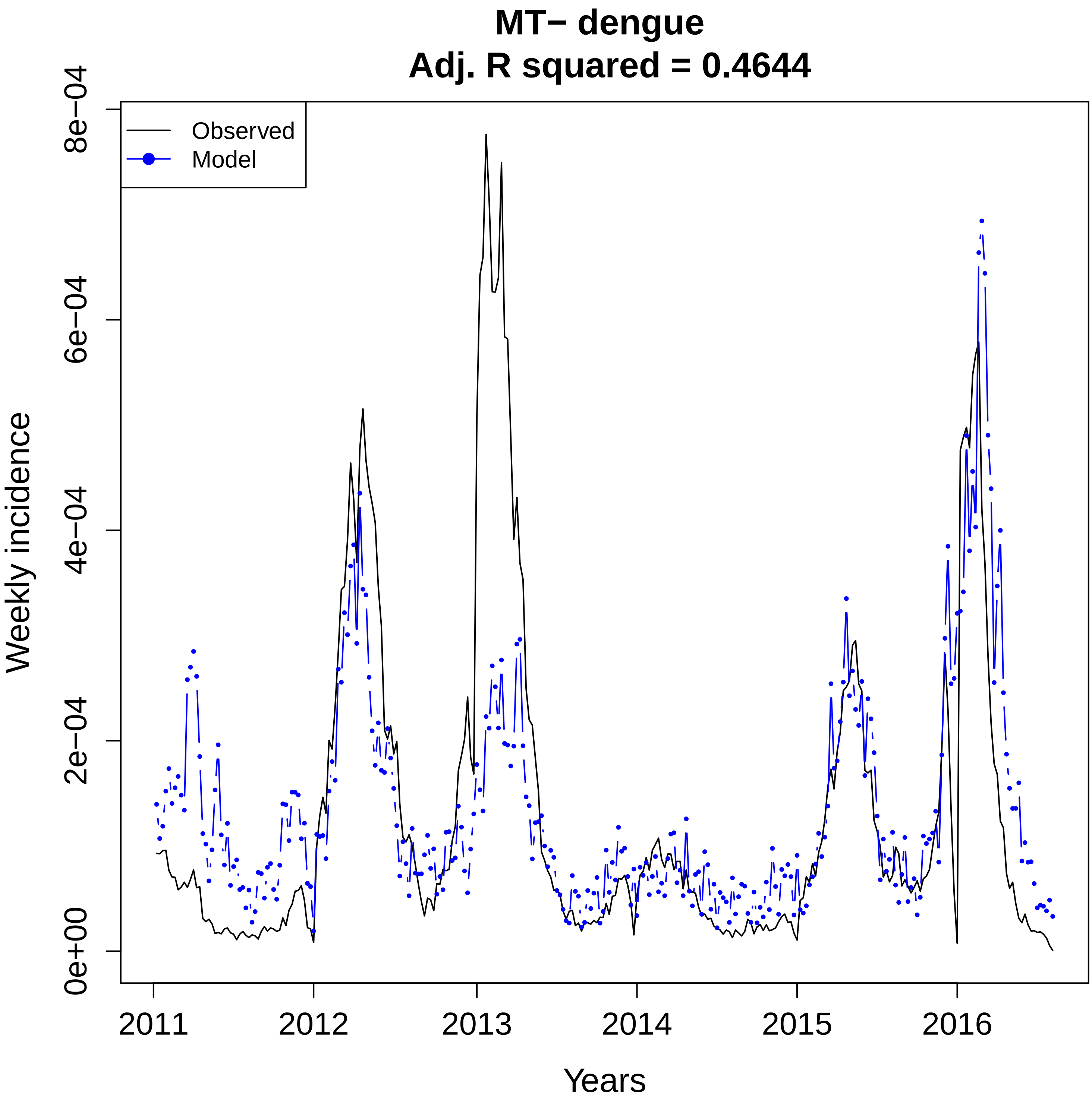

MT− dengue
Adj. R squared = 0.4644
Observed
Model
Weekly incidence
0e+00
2e−04
4e−04
6e−04
8e−04
2011
2012
2013
2014
2015
2016
Years

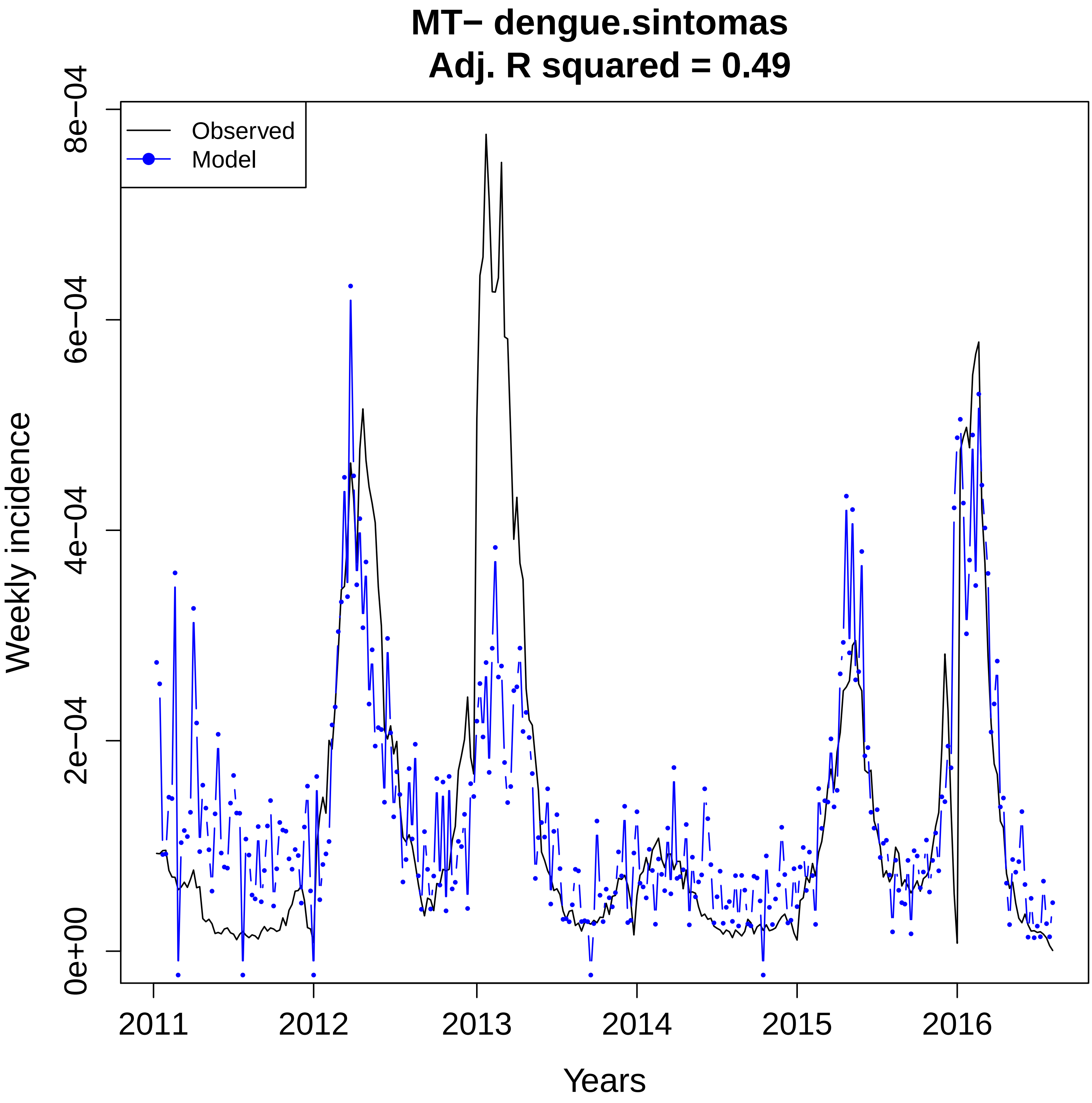

MT− dengue.sintomas
Adj. R squared = 0.49
Observed
Model
Weekly incidence
0e+00
2e−04
4e−04
6e−04
8e−04
2011
2012
2013
2014
2015
2016
Years

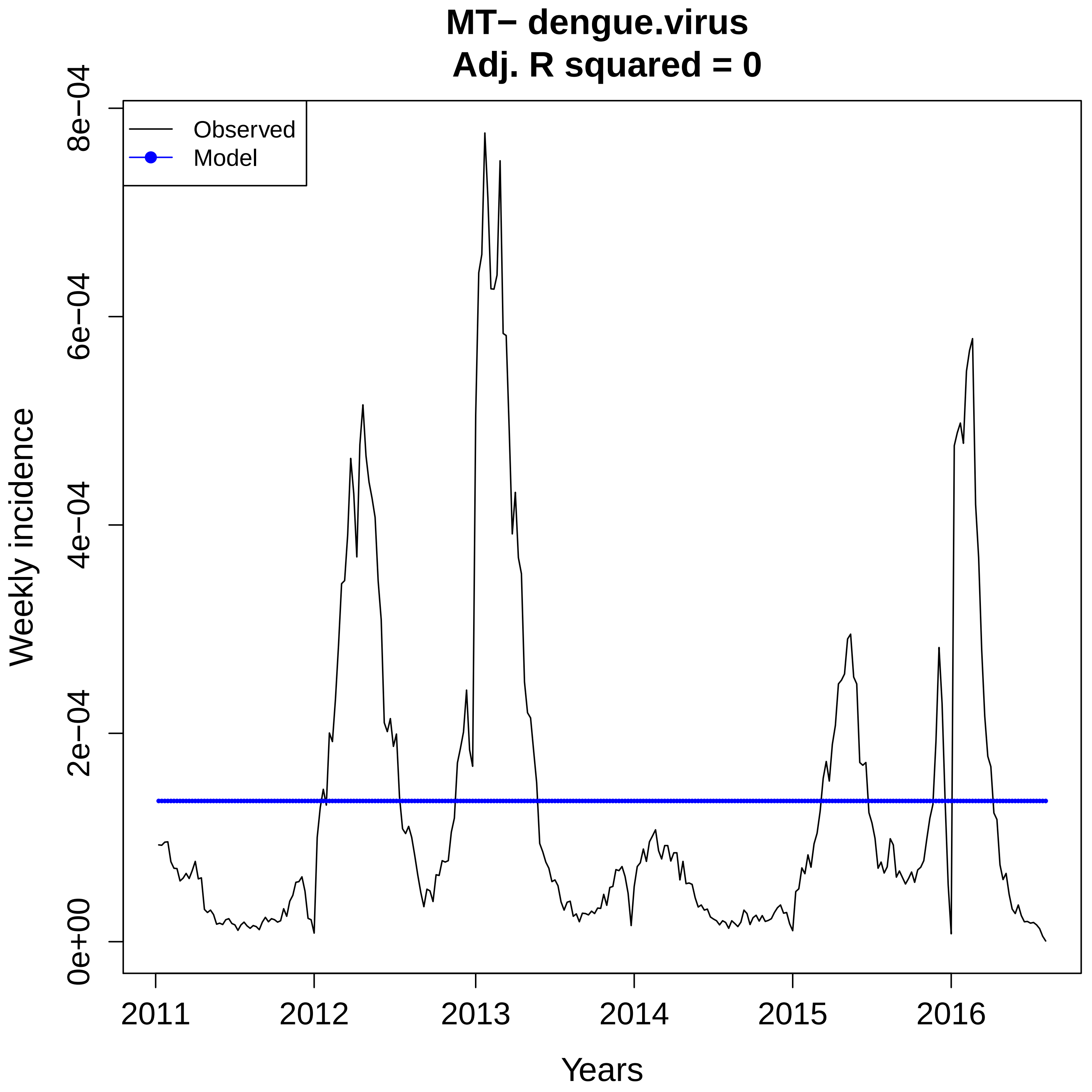
MT− dengue.virus
Adj. R squared = 0
Observed
Model
Weekly incidence
0e+00
2e−04
4e−04
6e−04
8e−04
2011
2012
2013
2014
2015
2016
Years

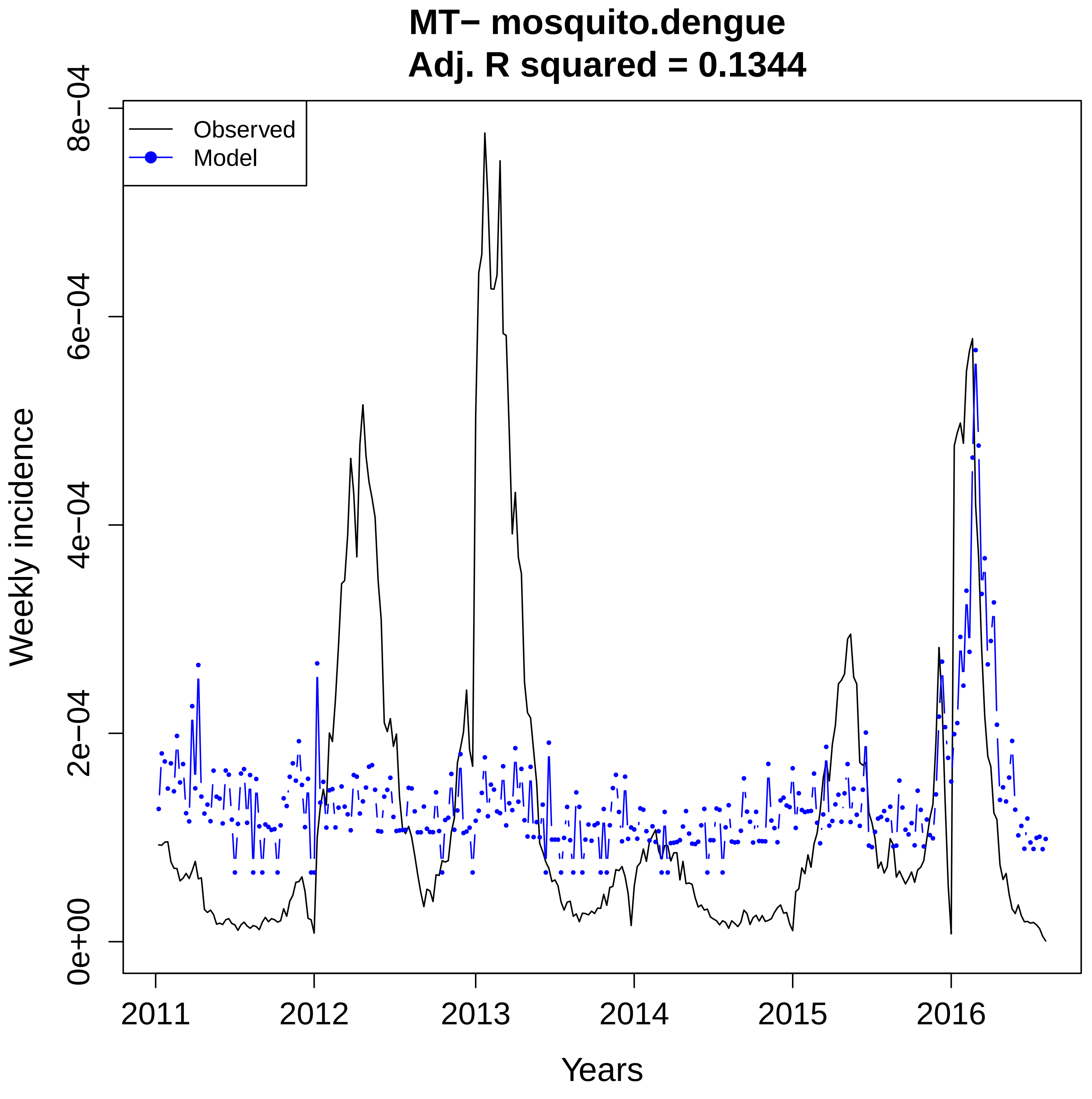

MT− mosquito.dengue
Adj. R squared = 0.1344
Observed
Model
Weekly incidence
0e+00
2e−04
4e−04
6e−04
8e−04
2011
2012
2013
2014
2015
2016
Years

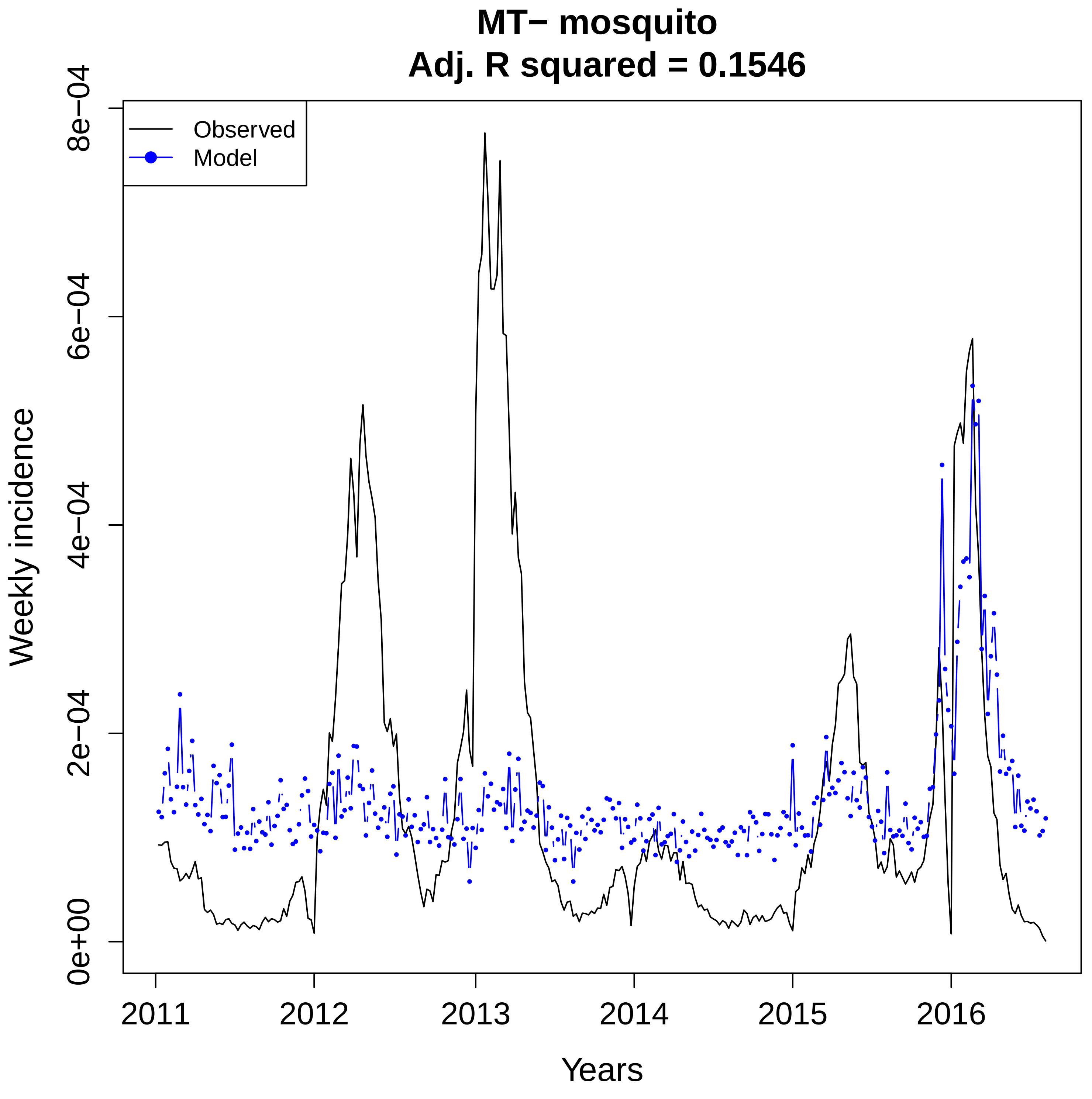

MT− mosquito
Adj. R squared = 0.1546
Observed
Model
Weekly incidence
0e+00
2e−04
4e−04
6e−04
8e−04
2011
2012
2013
2014
2015
2016
Years

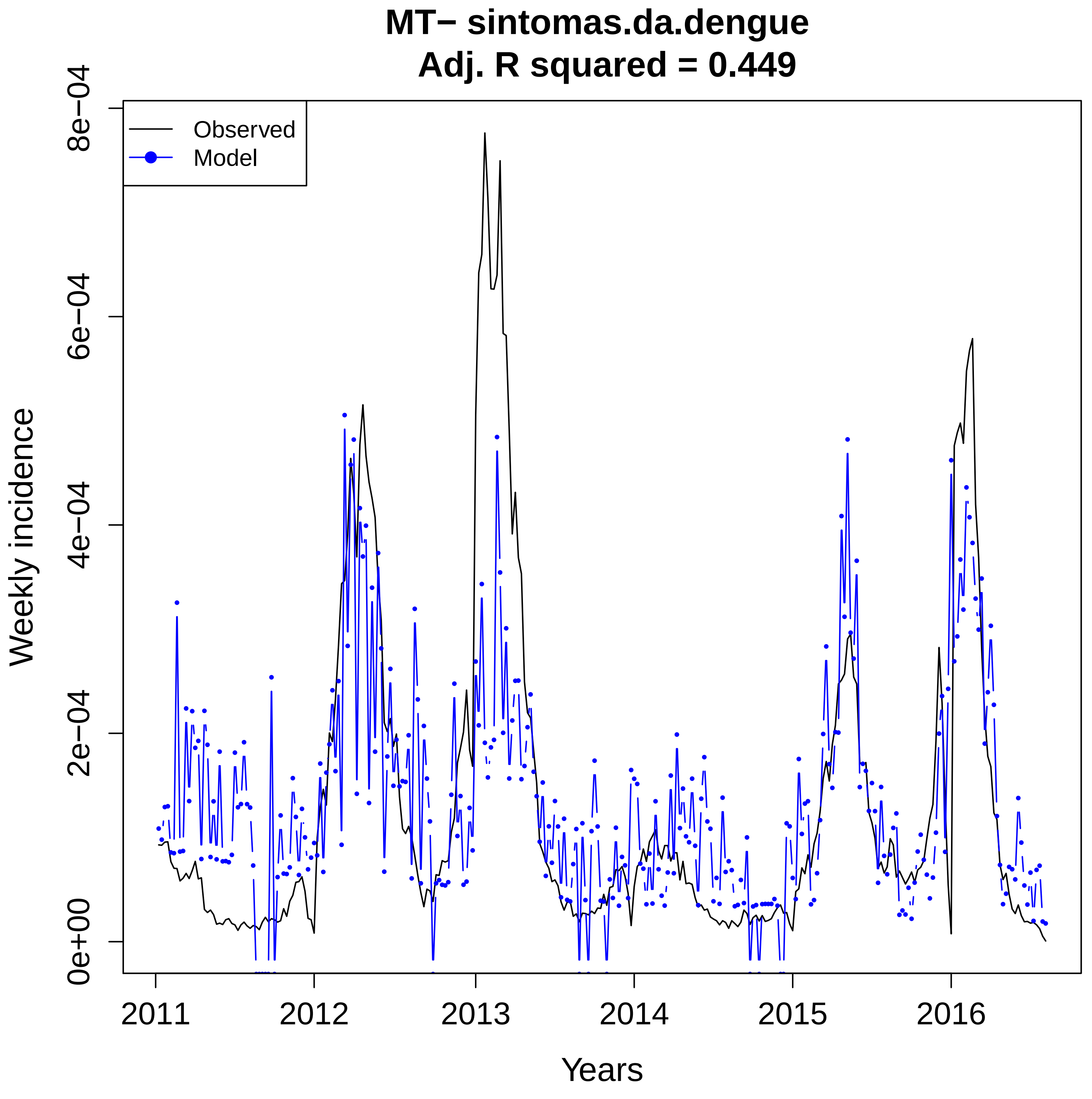
MT− sintomas.da.dengue
Adj. R squared = 0.449
Observed
Model
Weekly incidence
0e+00
2e−04
4e−04
6e−04
8e−04
2011
2012
2013
2014
2015
2016
Years

Pará (PA)
Dengue incidence vs. Google Health Trends (2011–2016)

Definitions:

- **Combined terms:**
  - **All:** model with all the available terms. n= number of terms
  - **Four:** model with "dengue", "dengue sintomas", "aedes", and "mosquito".
  - **Uncorrelated:** model with non-correlated terms. n = number of uncorrelated terms
- **Individual terms:** Plots developed with one term at a time.

- Terms for all and uncorrelated models can be found in additional file 3.

Combined terms
Adjusted R squared

Pará (PA)

All
(n = 9)

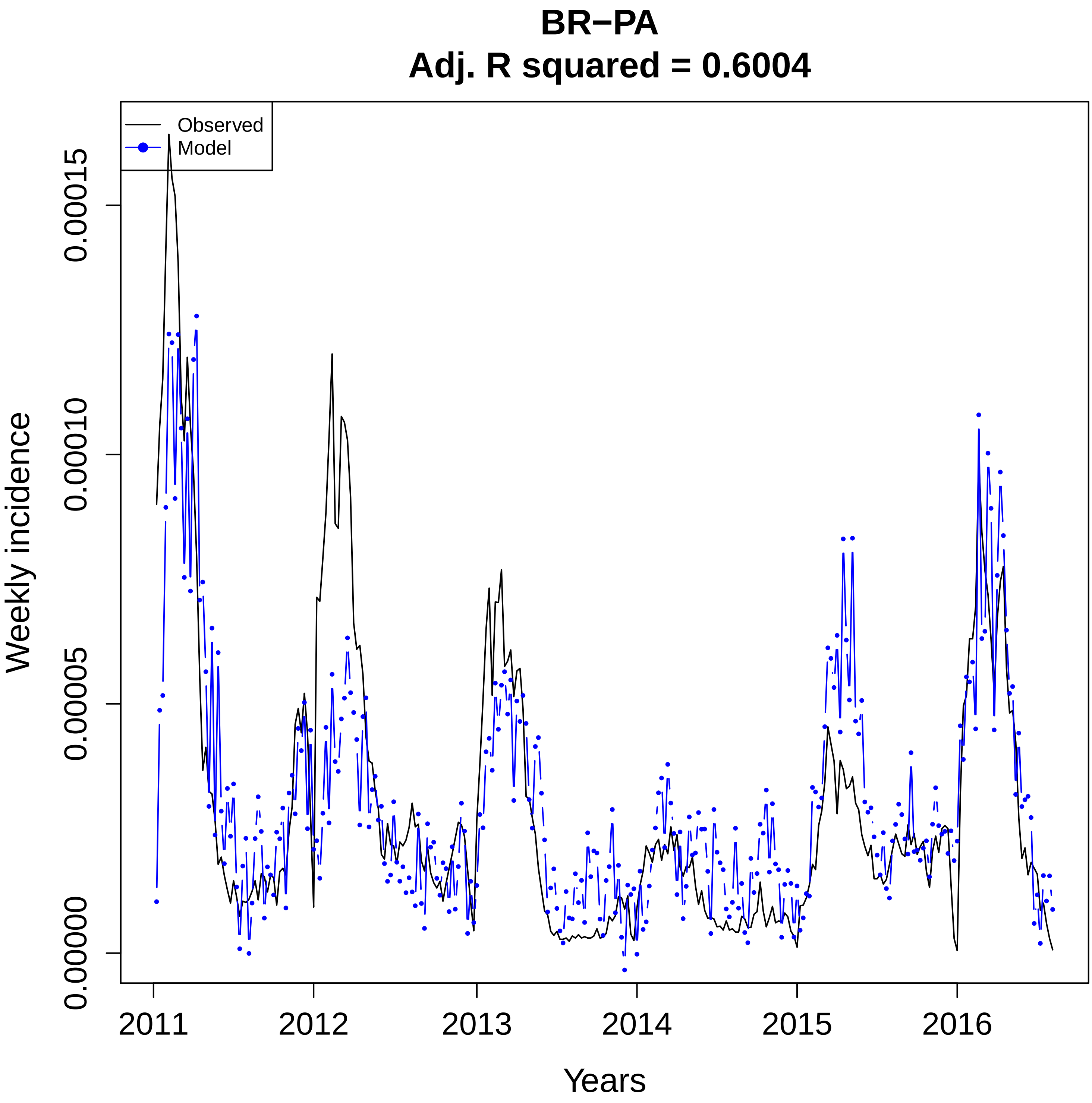
BR−PA
Adj. R squared = 0.6004
Observed
Model
Weekly incidence
0.00000
0.00005
0.00010
0.00015
2011
2012
2013
2014
2015
2016
Years

Four

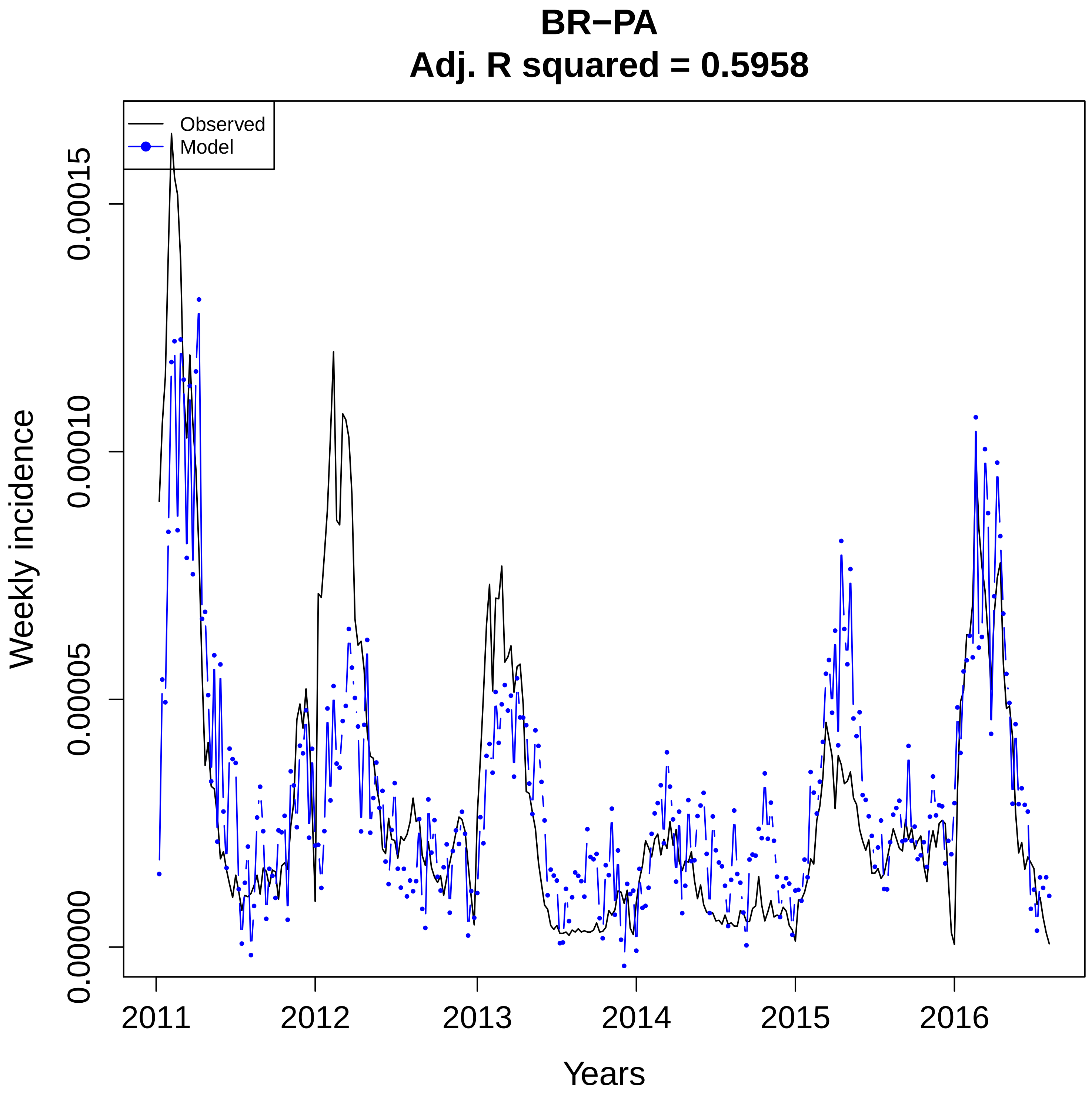

BR−PA
Adj. R squared = 0.5958
Observed
Model
Weekly incidence
0.00000
0.00005
0.00010
0.00015
2011
2012
2013
2014
2015
2016
Years

Uncorrelated
(n = 2)

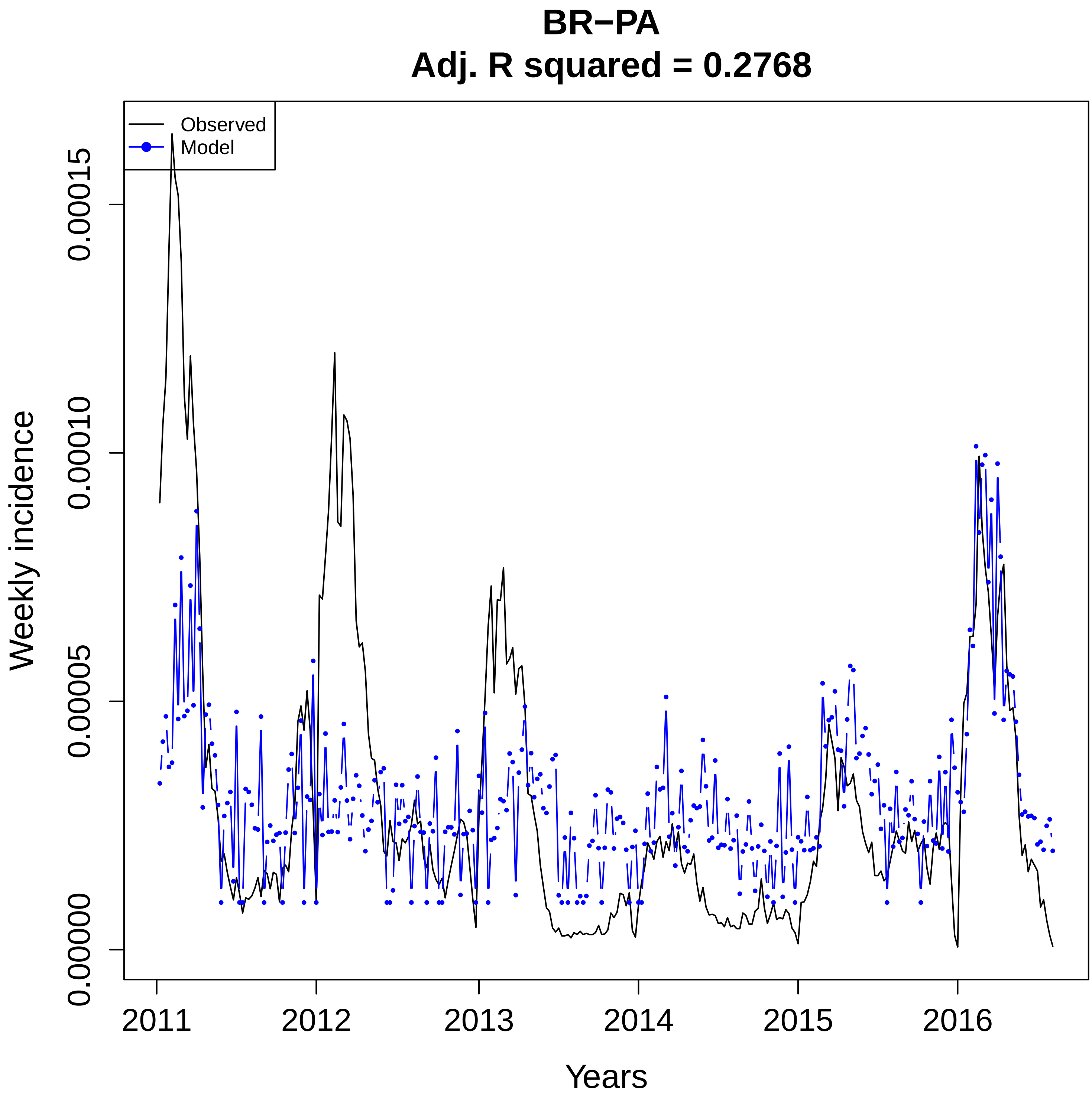

Individual terms
Adjusted R squared

Pará (PA)

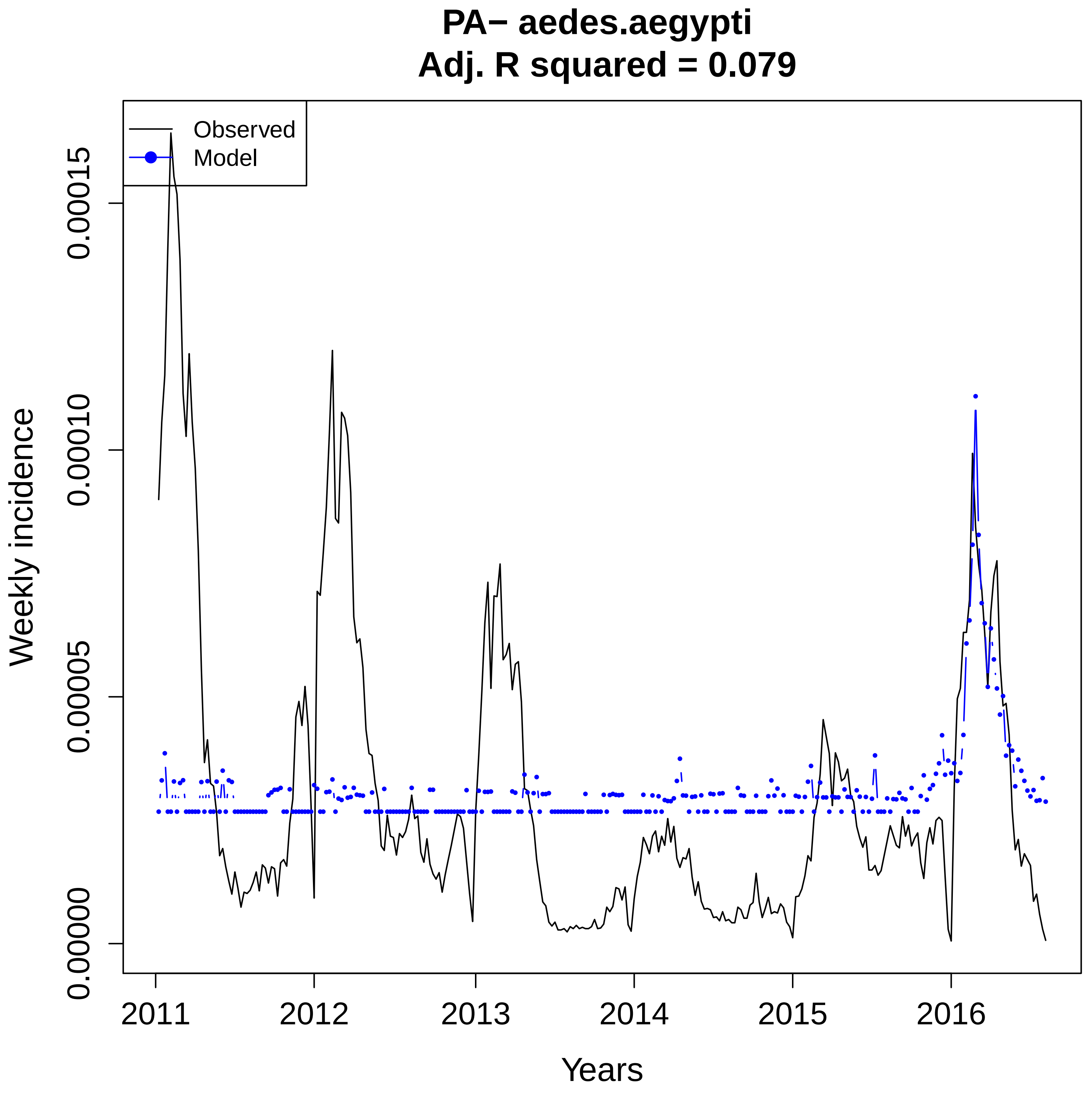

PA− aedes.aegypti
Adj. R squared = 0.079
Observed
Model
Weekly incidence
0.00000
0.00005
0.00010
0.00015
2011
2012
2013
2014
2015
2016
Years

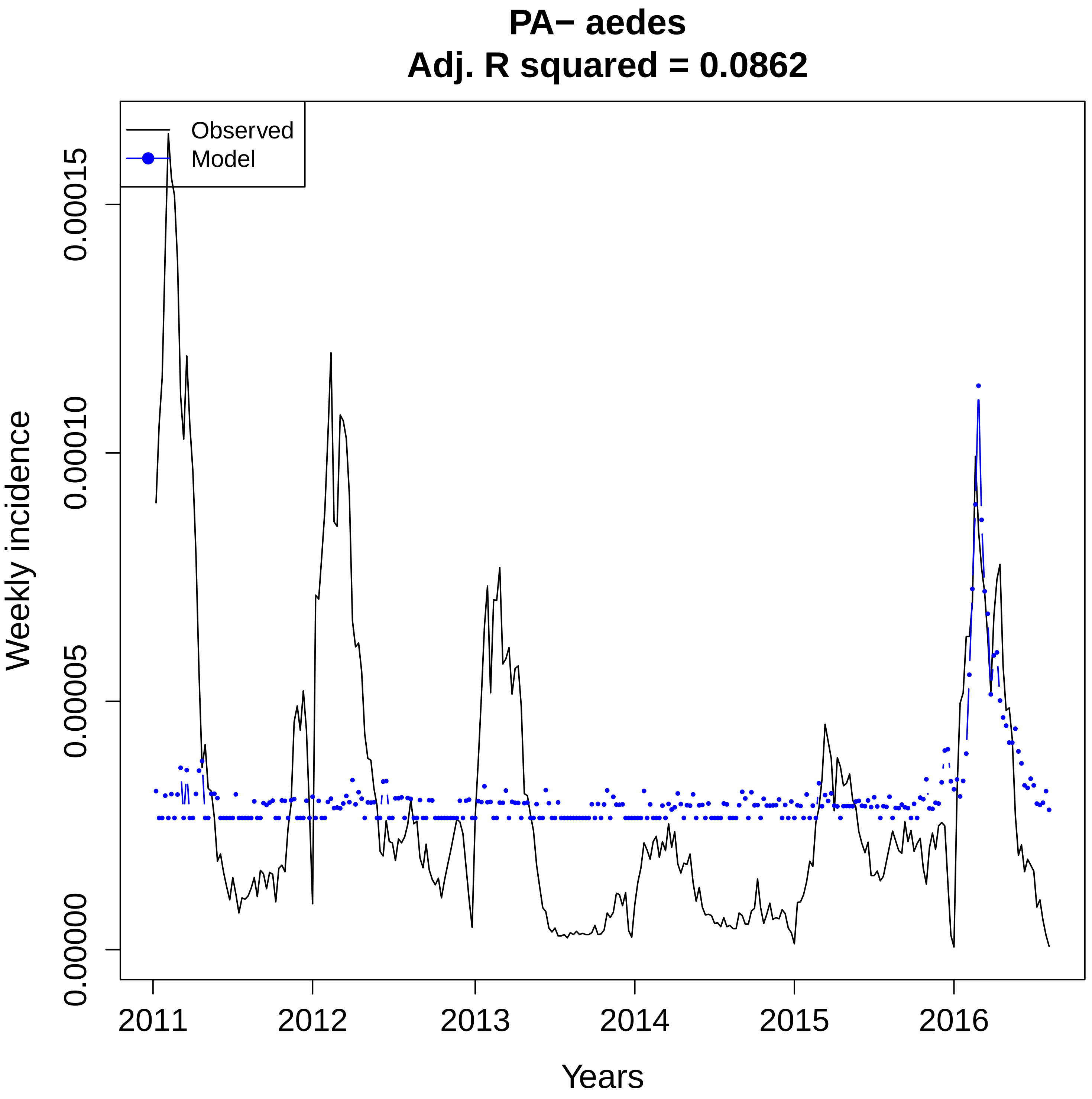
PA− aedes
Adj. R squared = 0.0862
Observed
Model
Weekly incidence
0.00000
0.00005
0.00010
0.00015
2011
2012
2013
2014
2015
2016
Years

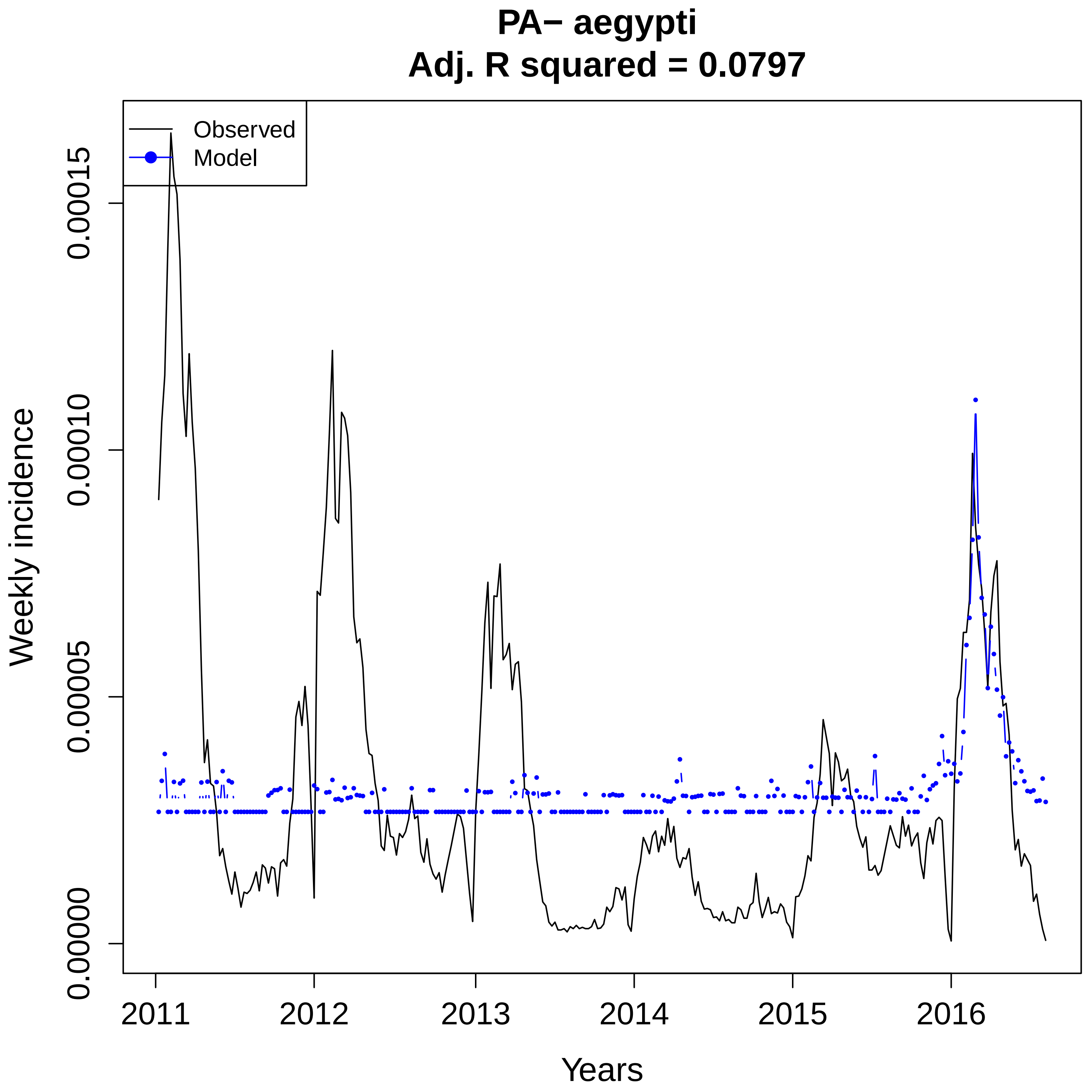

PA− aegypti
Adj. R squared = 0.0797
Observed
Model
Weekly incidence
0.00000
0.00005
0.00010
0.00015
2011
2012
2013
2014
2015
2016
Years

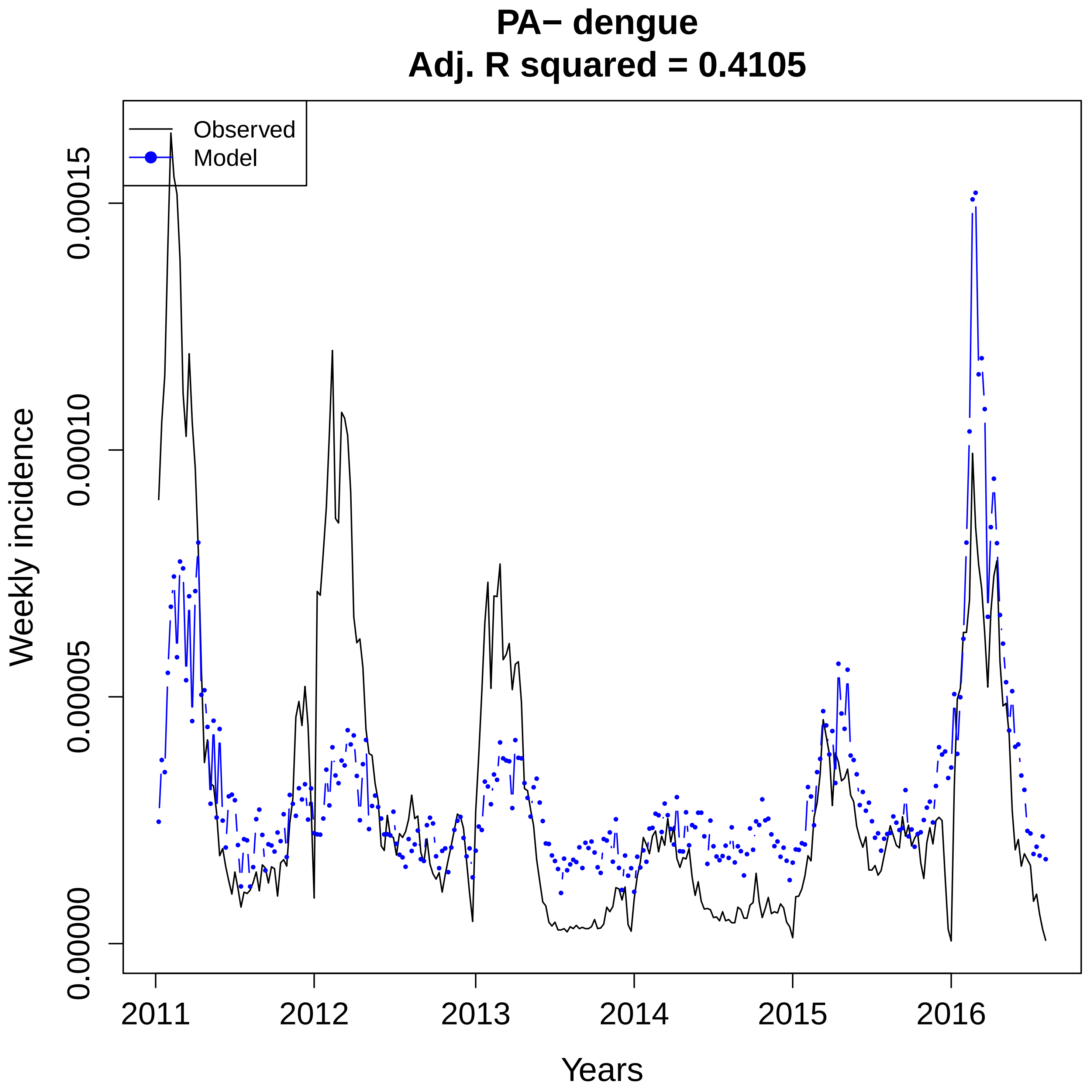

PA− dengue
Adj. R squared = 0.4105
Observed
Model
Weekly incidence
0.00000
0.00005
0.00010
0.00015
2011
2012
2013
2014
2015
2016
Years

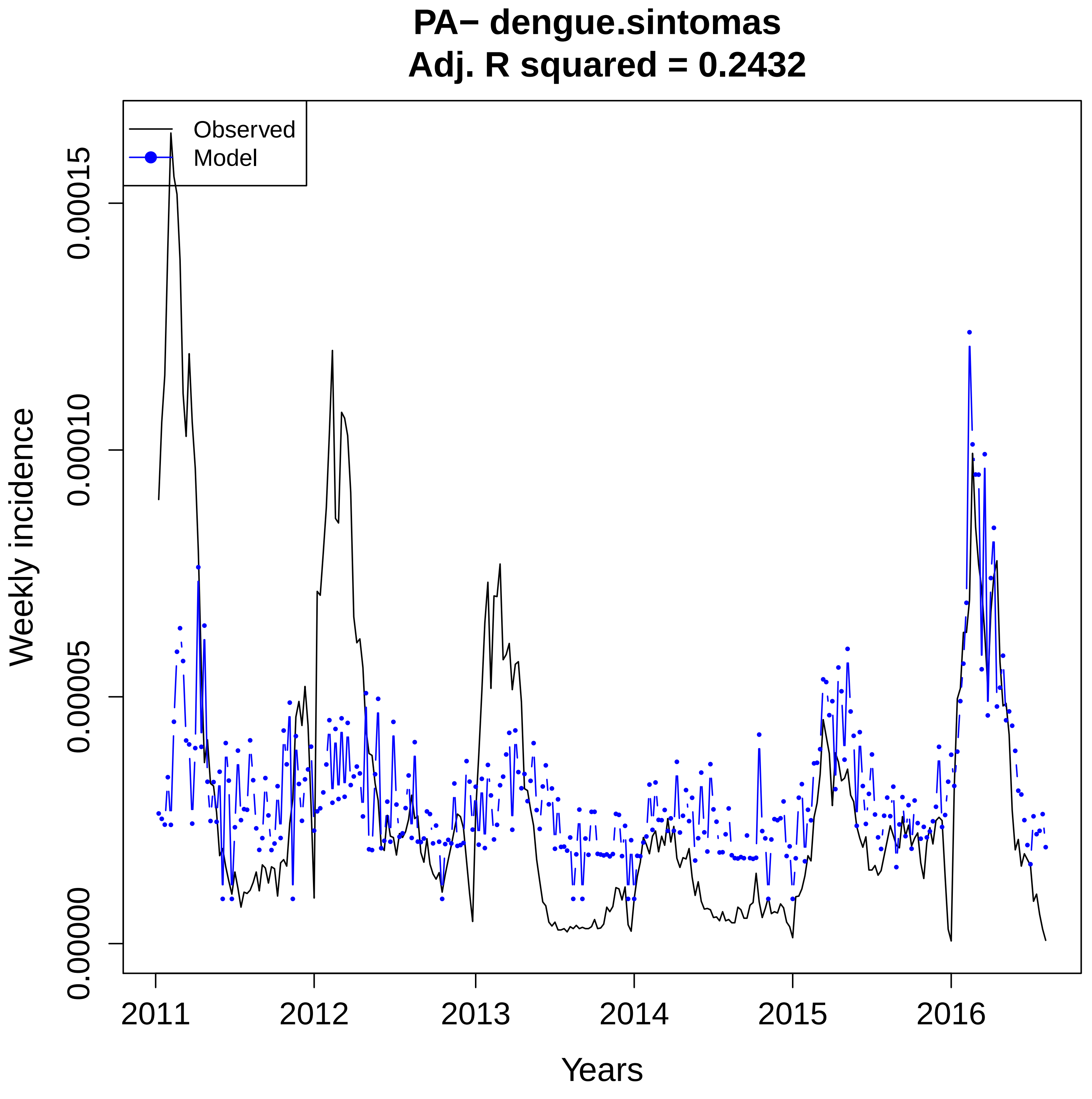
PA− dengue.sintomas
Adj. R squared = 0.2432
Observed
Model
Weekly incidence
0.00000
0.00005
0.00010
0.00015
2011
2012
2013
2014
2015
2016
Years

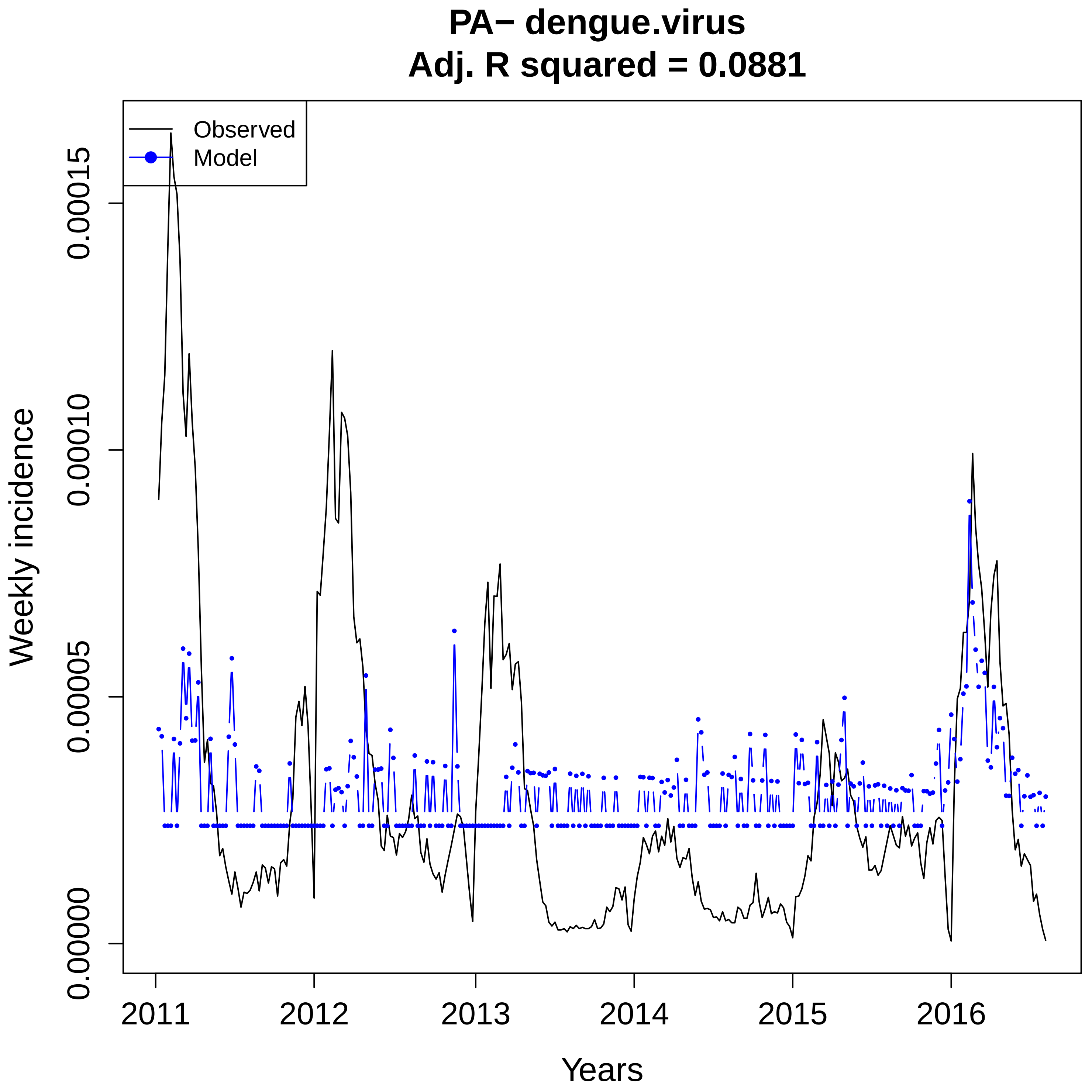

PA− dengue.virus
Adj. R squared = 0.0881
Observed
Model
Weekly incidence
0.00000
0.00005
0.00010
0.00015
2011
2012
2013
2014
2015
2016
Years

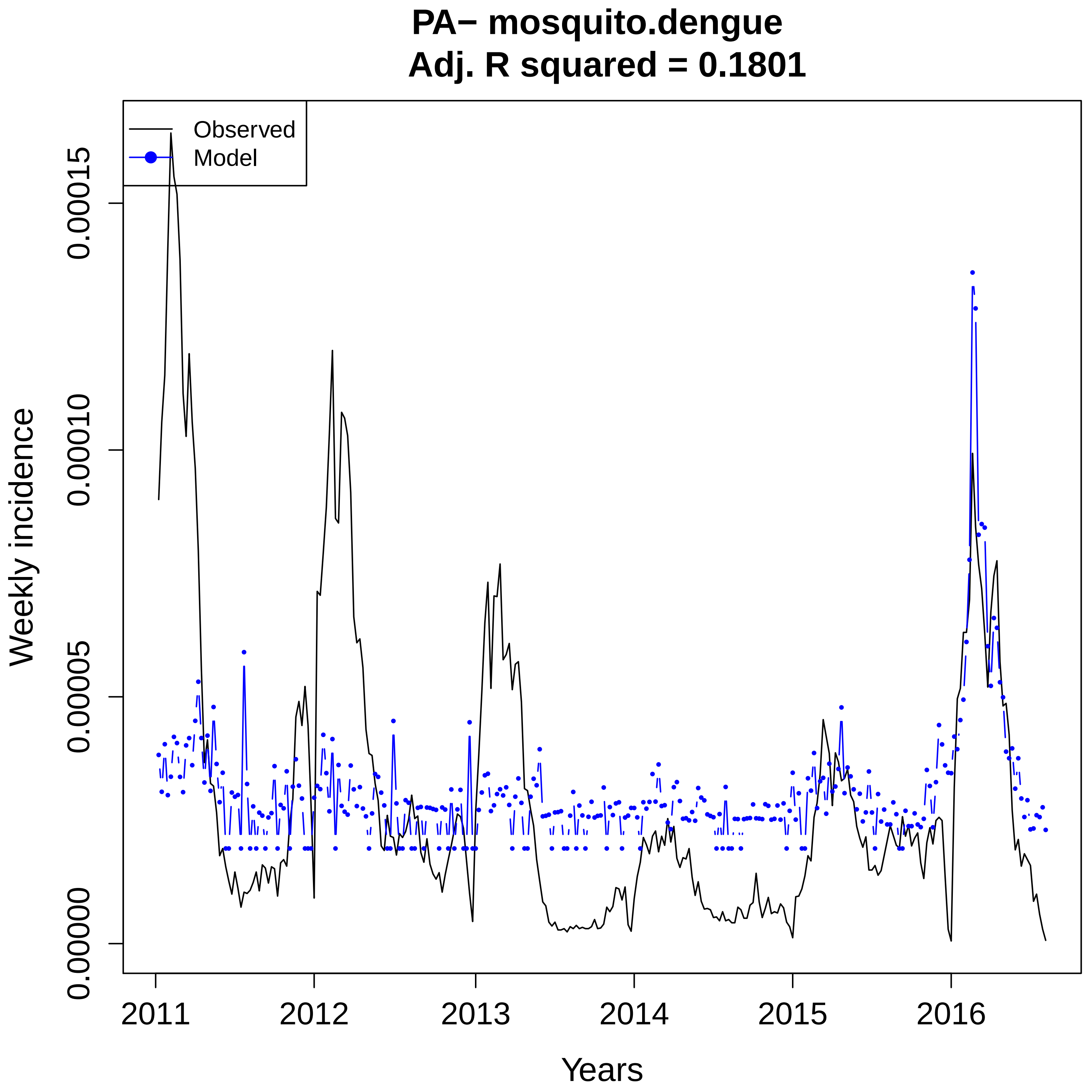
PA− mosquito.dengue
Adj. R squared = 0.1801
Observed
Model
Weekly incidence
0.00000
0.00005
0.00010
0.00015
2011
2012
2013
2014
2015
2016
Years

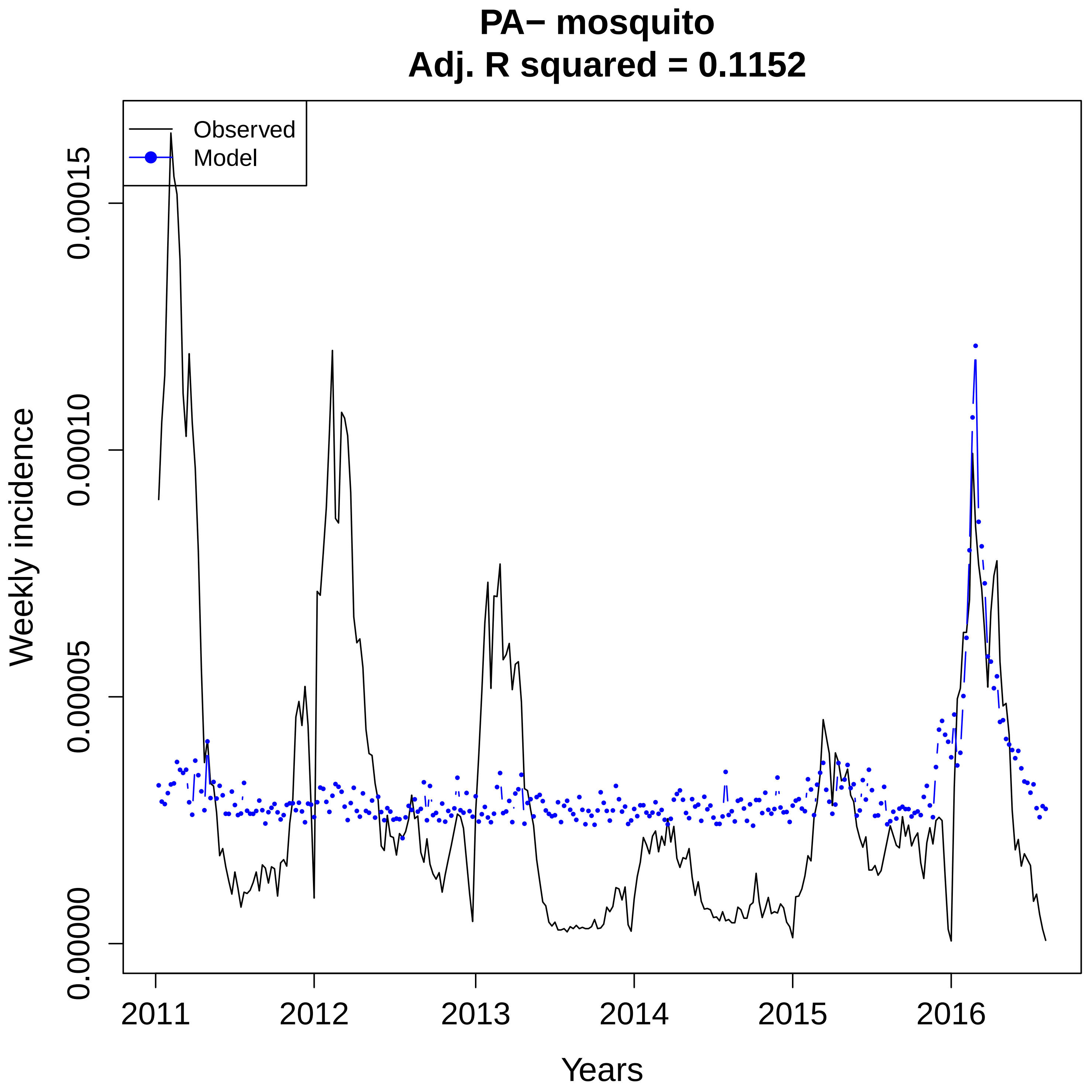
PA− mosquito
Adj. R squared = 0.1152
Observed
Model
Weekly incidence
0.00000
0.00005
0.00010
0.00015
2011
2012
2013
2014
2015
2016
Years

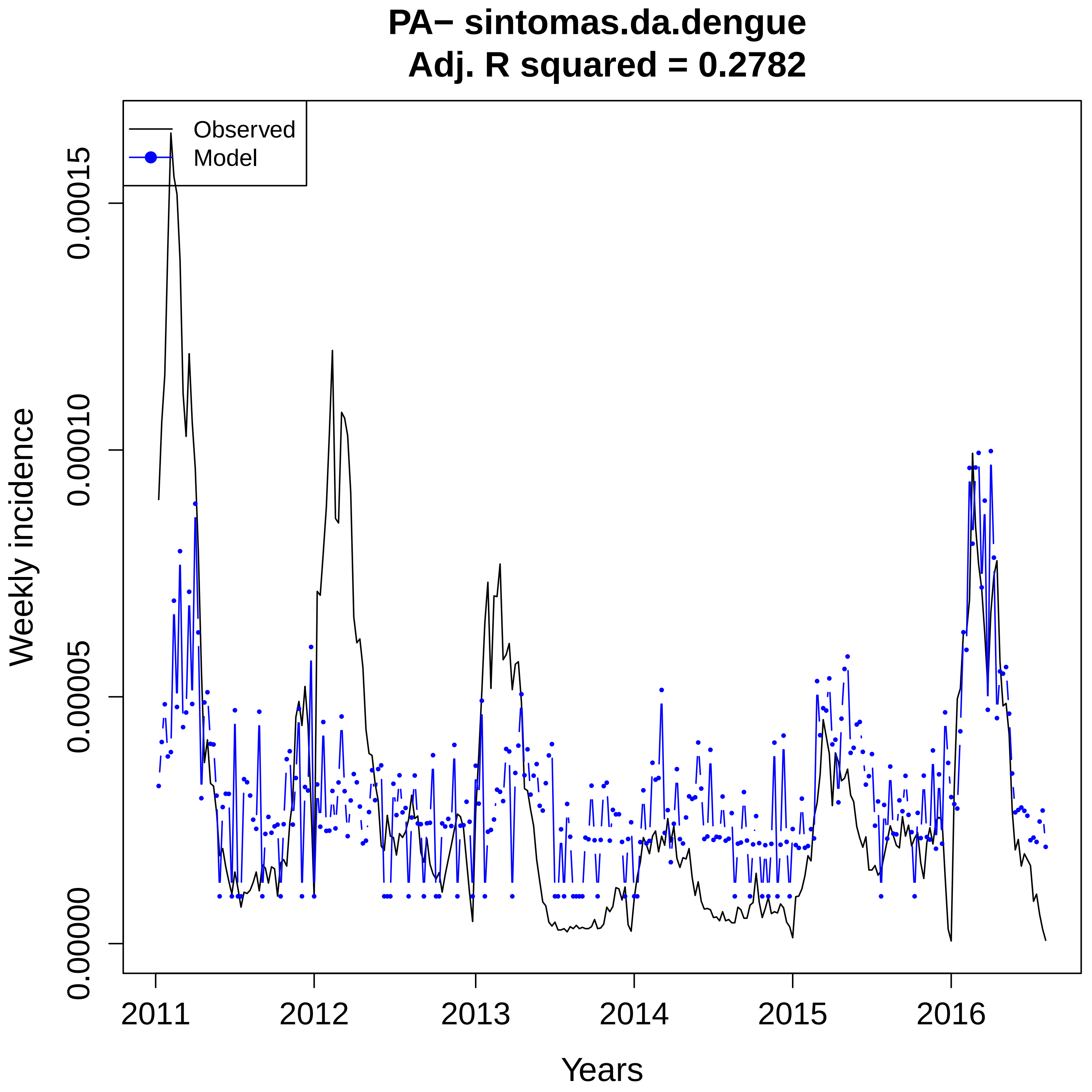

PA− sintomas.da.dengue
Adj. R squared = 0.2782
Observed
Model
Weekly incidence
0.00000
0.00005
0.00010
0.00015
2011
2012
2013
2014
2015
2016
Years

Paraiba (PB)

Dengue incidence vs. Google Health Trends (2011–2016)

Definitions:

- **Combined terms:**
  - **All:** model with all the available terms. n= number of terms
  - **Four:** model with "dengue", "dengue sintomas", "aedes", and "mosquito".
  - **Uncorrelated:** model with non-correlated terms. n = number of uncorrelated terms
- **Individual terms:** Plots developed with one term at a time.

- Terms for all and uncorrelated models can be found in additional file 3.

Combined terms
Adjusted R squared

Paraiba (PB)

All
(n = 10)

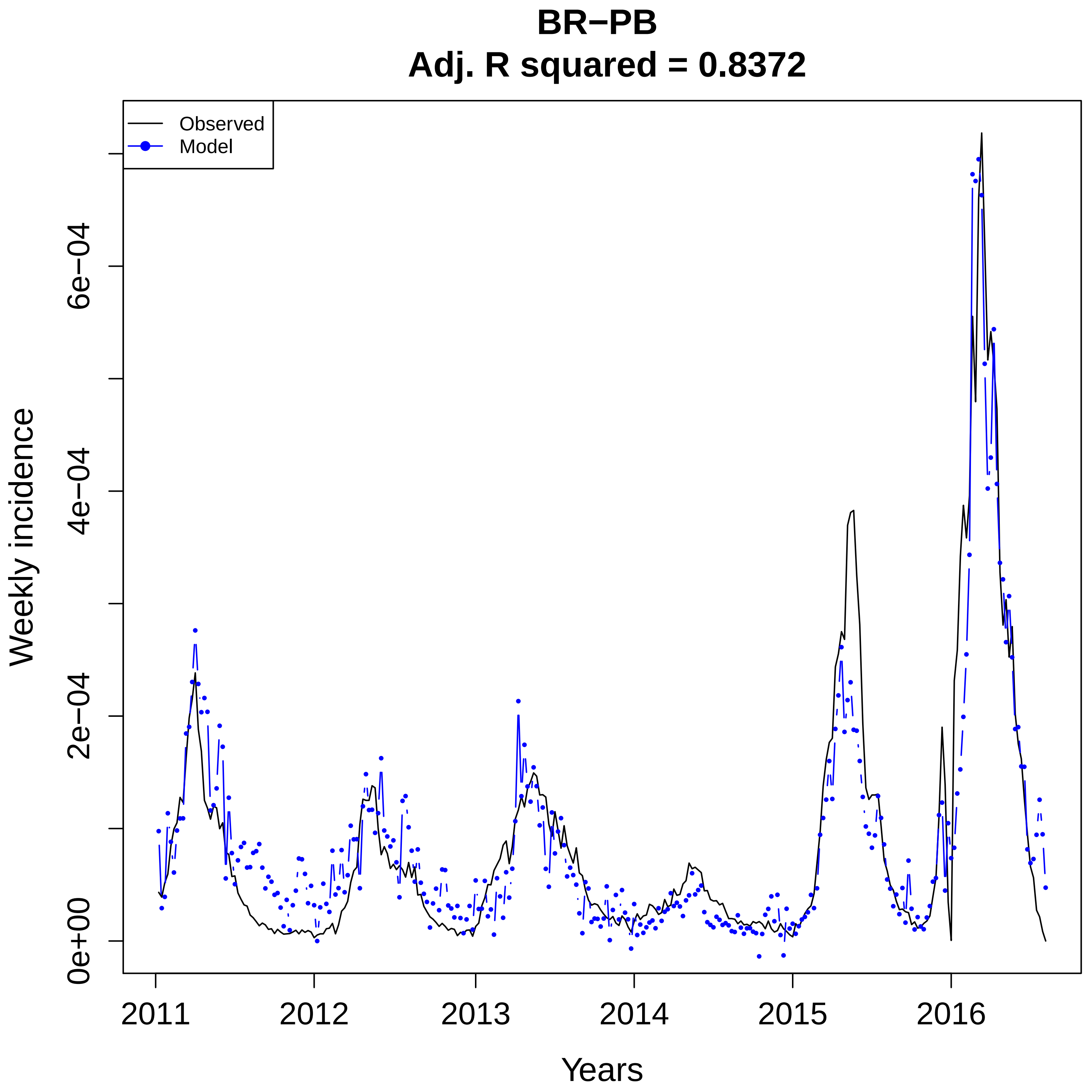
BR−PB
Adj. R squared = 0.8372
Observed
Model
Weekly incidence
0e+00
2e−04
4e−04
6e−04
2011
2012
2013
2014
2015
2016
Years

Four

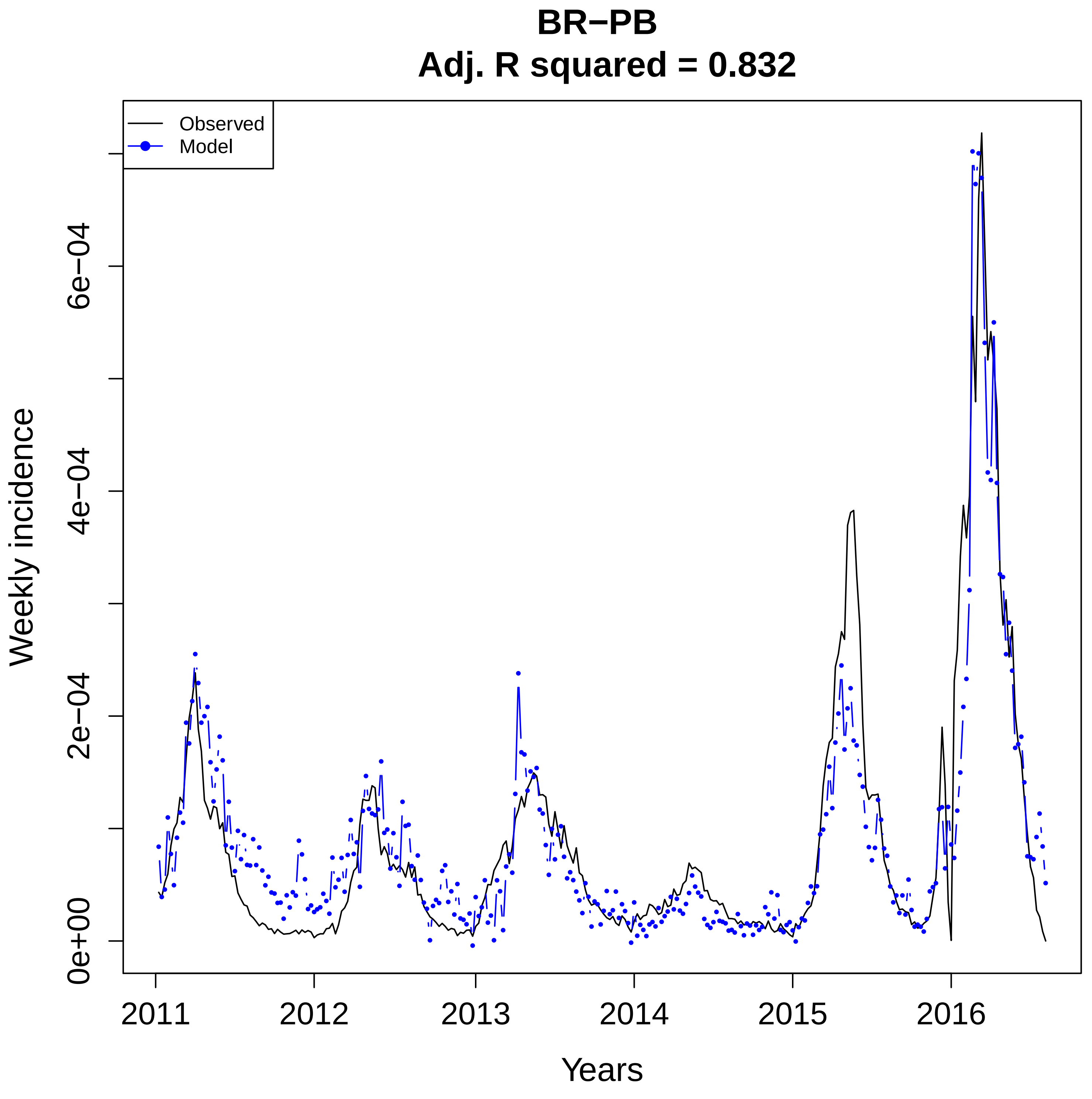

BR−PB
Adj. R squared = 0.832
Observed
Model
Weekly incidence
0e+00
2e−04
4e−04
6e−04
2011
2012
2013
2014
2015
2016
Years

Uncorrelated
(n = 3)

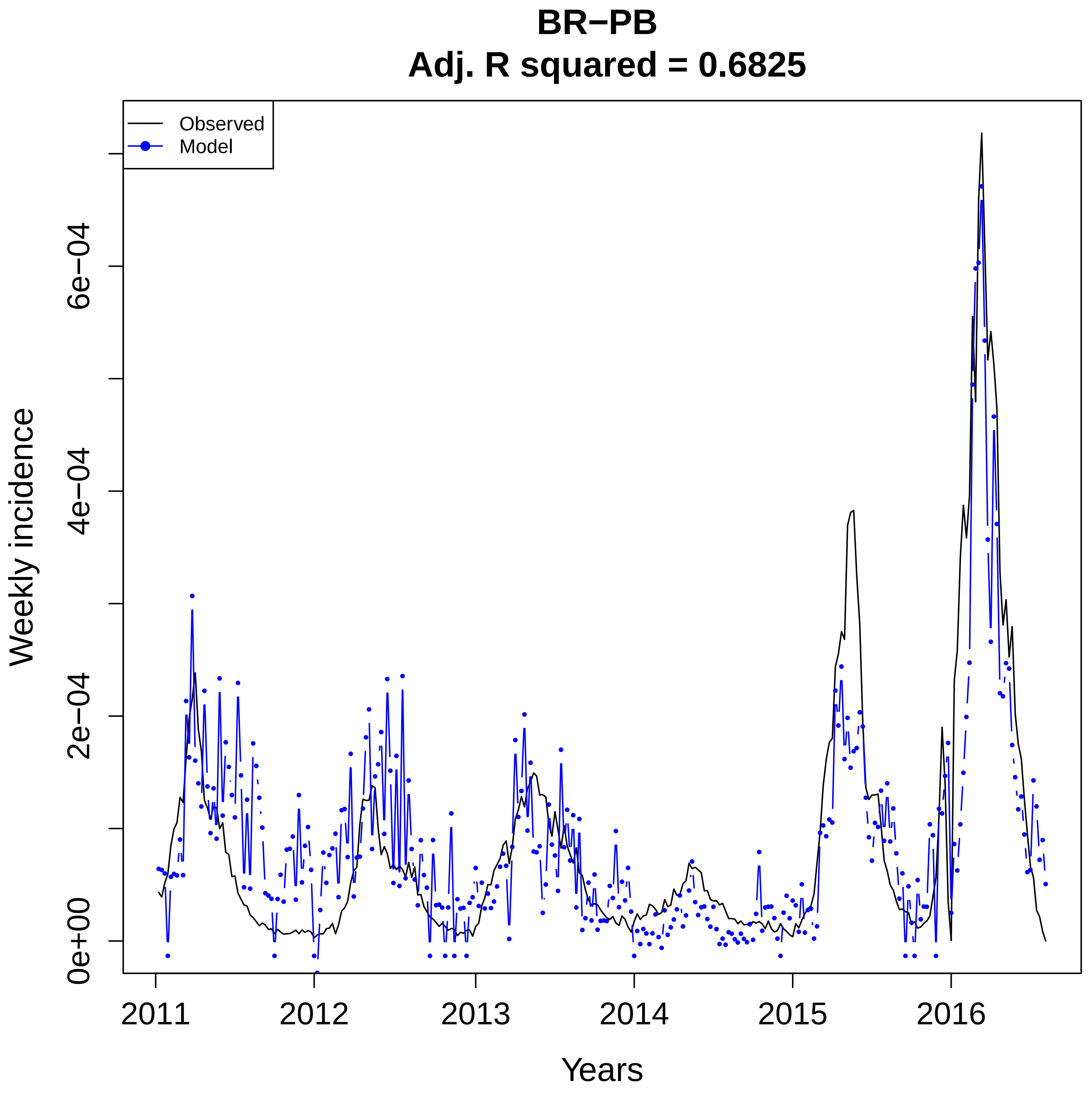

Individual terms
Adjusted R squared

Paraiba (PB)

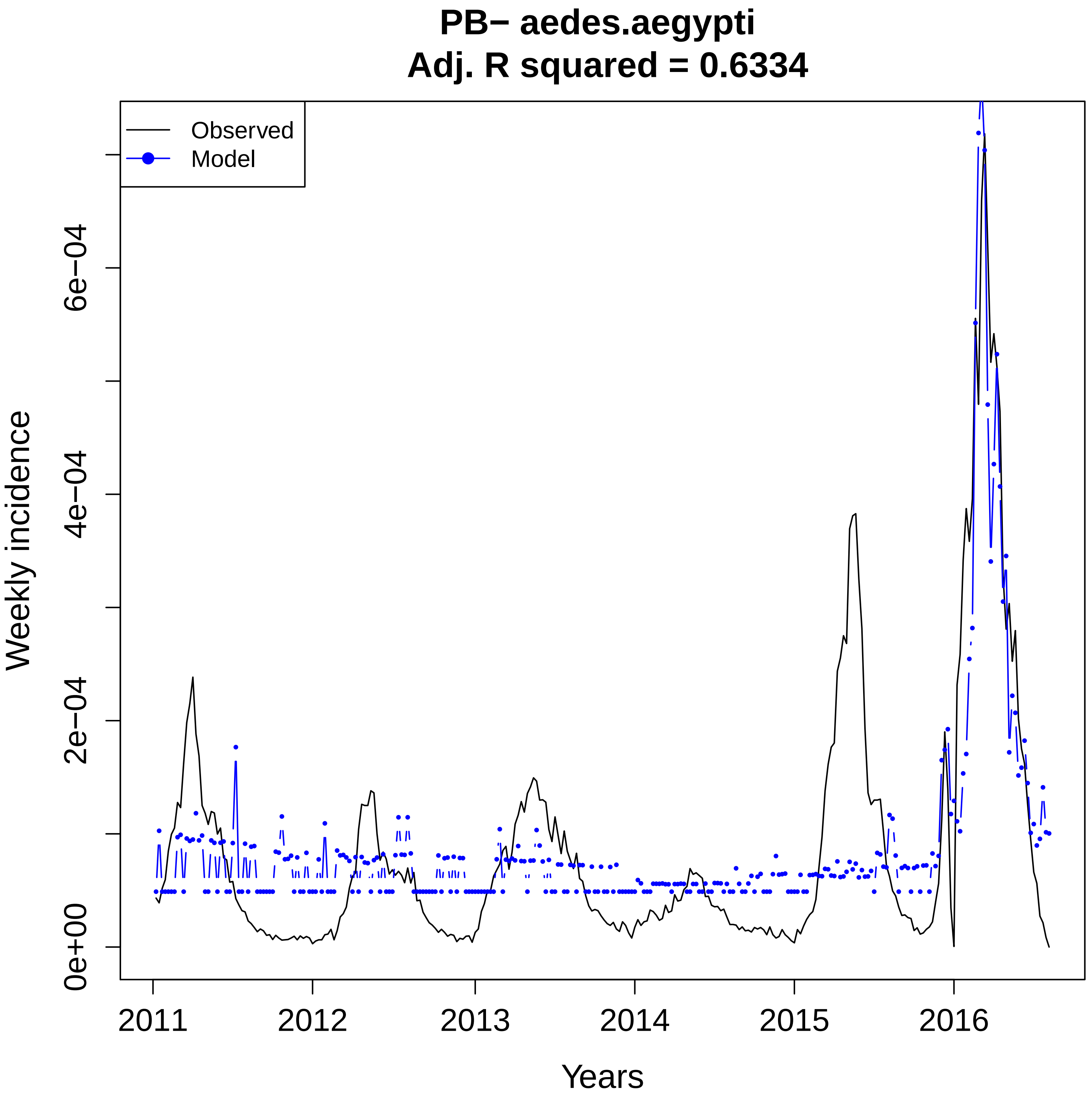
PB− aedes.aegypti
Adj. R squared = 0.6334
Observed
Model
Weekly incidence
0e+00
2e−04
4e−04
6e−04
2011
2012
2013
2014
2015
2016
Years

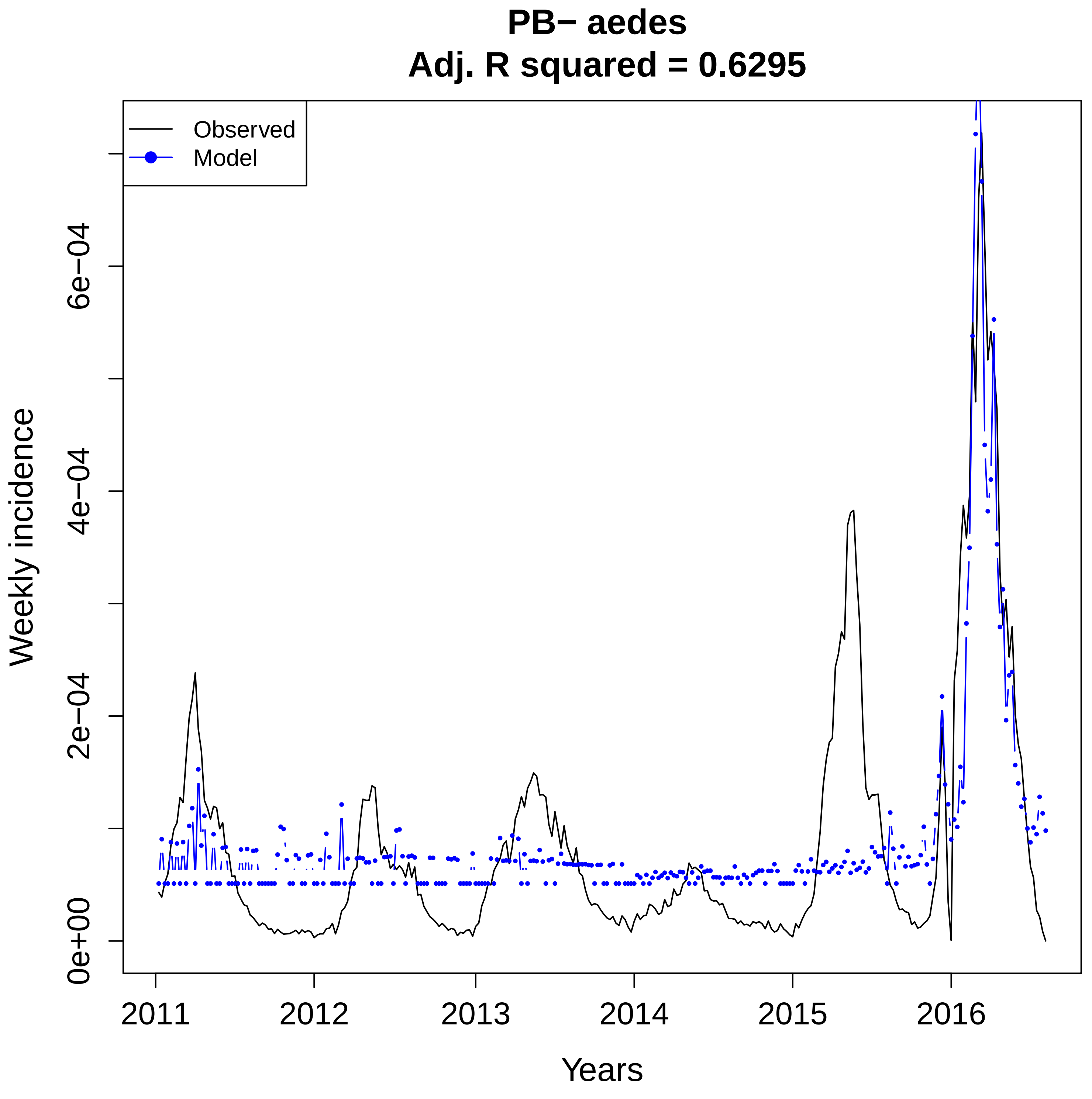
PB− aedes
Adj. R squared = 0.6295
Observed
Model
Weekly incidence
0e+00
2e−04
4e−04
6e−04
2011
2012
2013
2014
2015
2016
Years

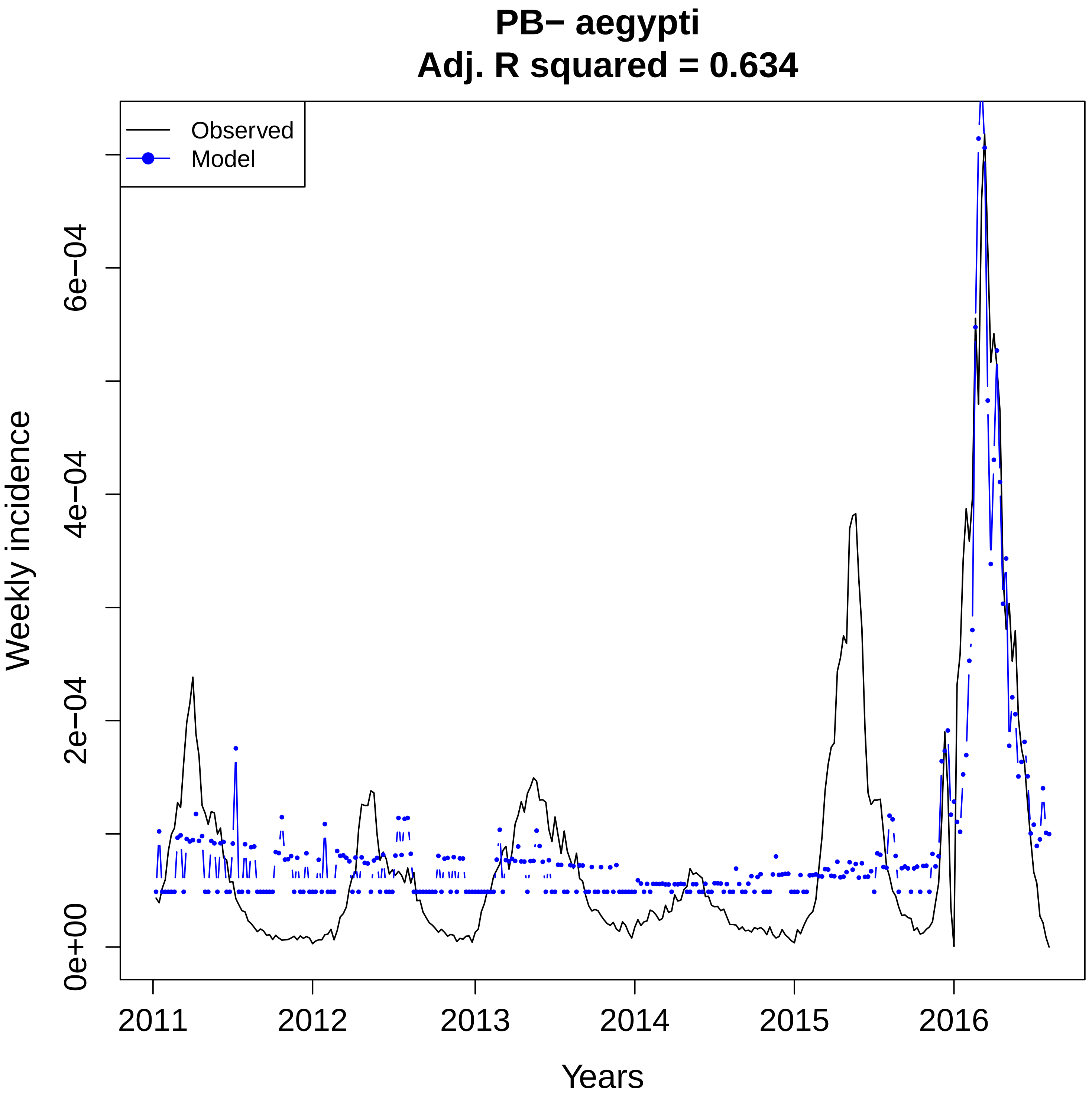

PB− aegypti
Adj. R squared = 0.634
Observed
Model
Weekly incidence
0e+00
2e−04
4e−04
6e−04
2011
2012
2013
2014
2015
2016
Years

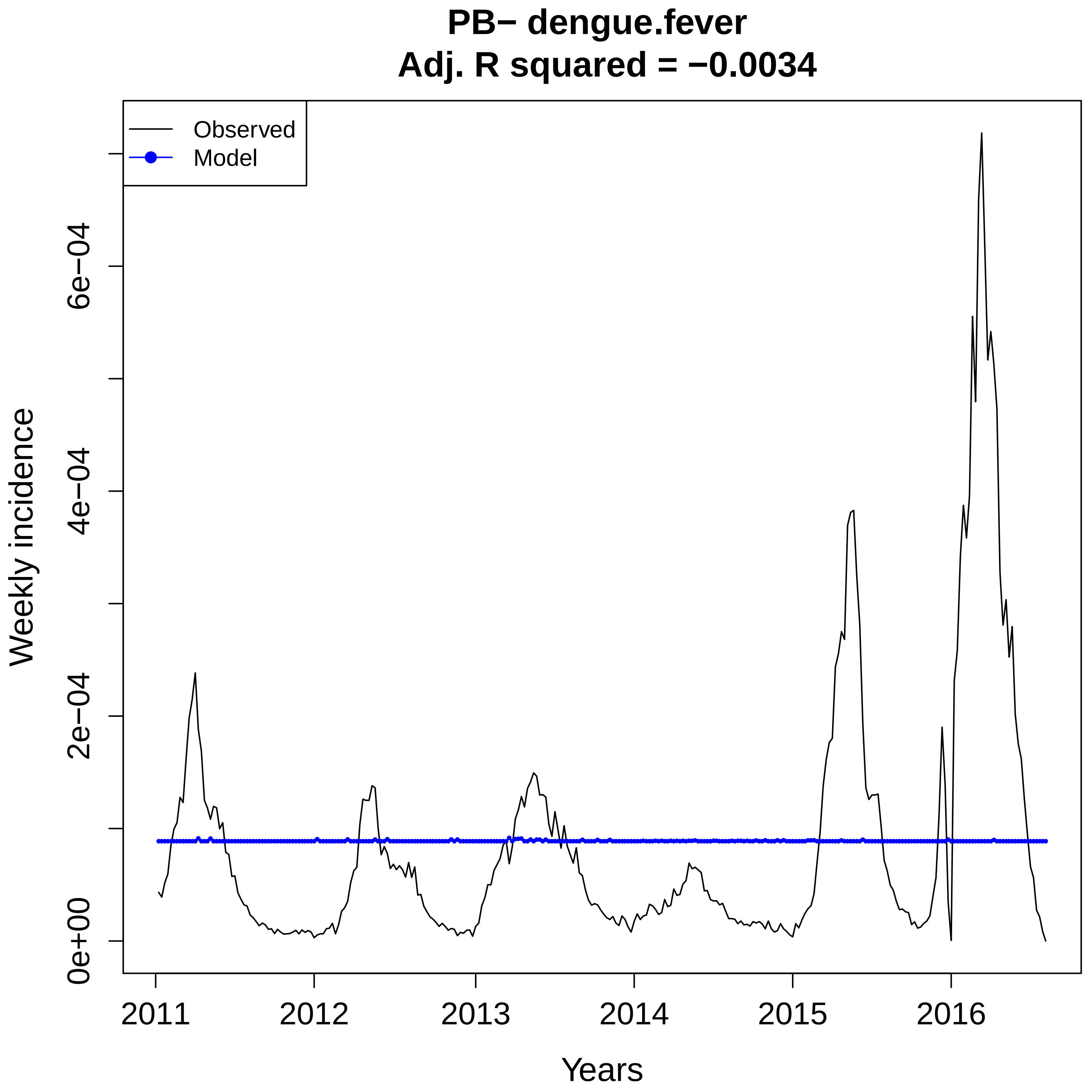

PB− dengue.fever
Adj. R squared = −0.0034
Observed
Model
Weekly incidence
0e+00
2e−04
4e−04
6e−04
2011
2012
2013
2014
2015
2016
Years

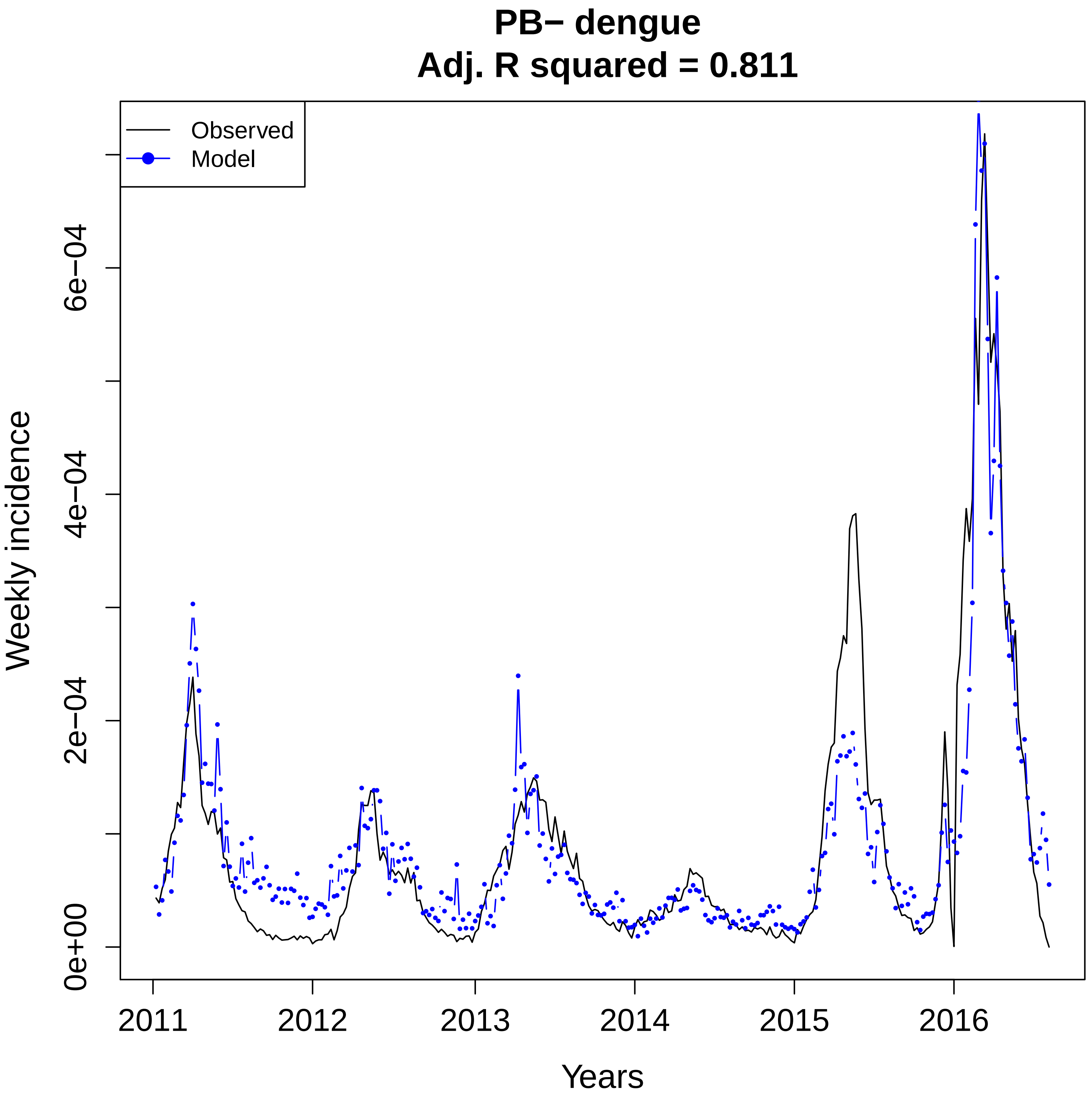
PB− dengue
Adj. R squared = 0.811
Observed
Model
Weekly incidence
0e+00
2e−04
4e−04
6e−04
2011
2012
2013
2014
2015
2016
Years

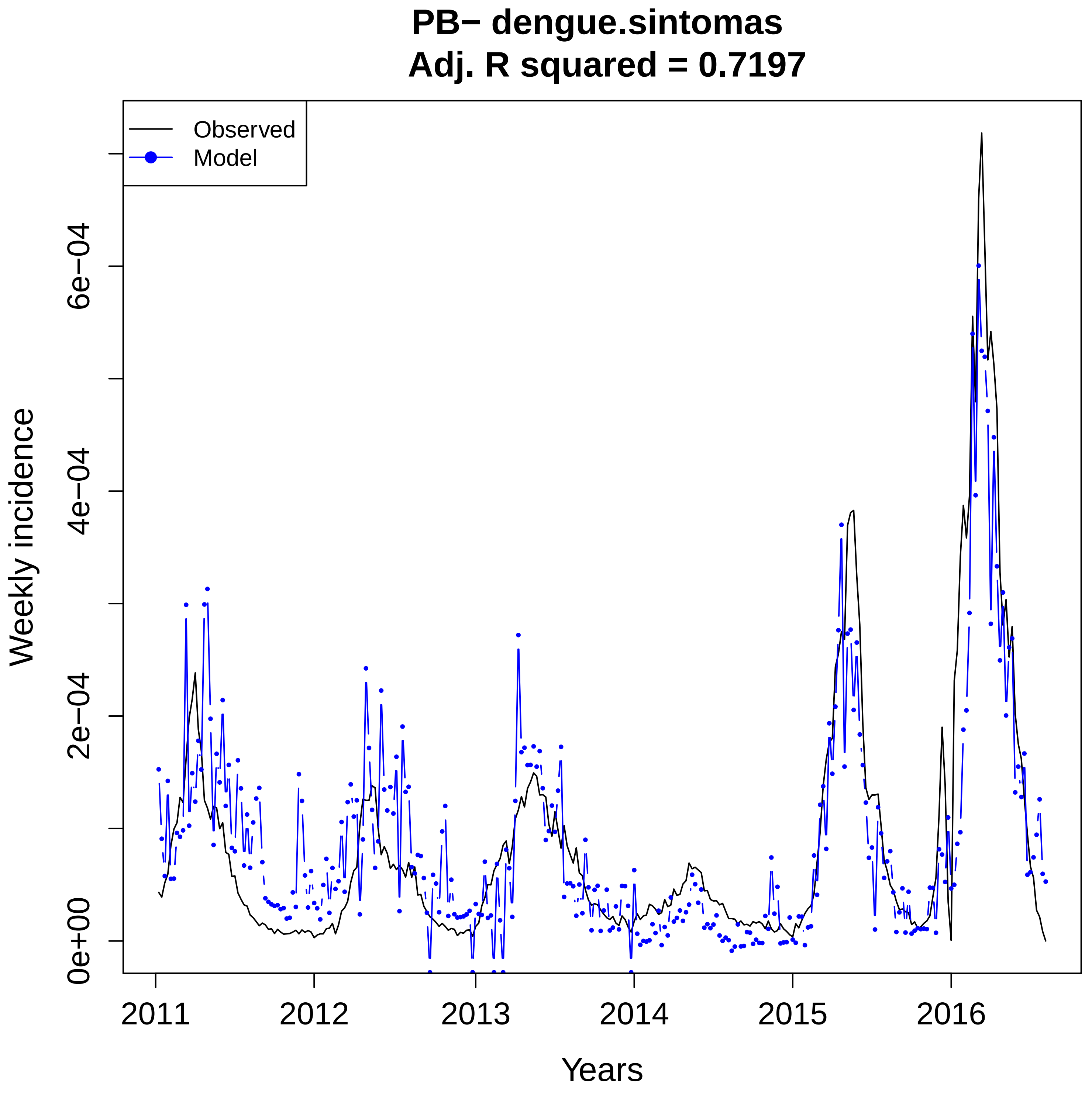

PB− dengue.sintomas
Adj. R squared = 0.7197
Observed
Model
Weekly incidence
0e+00
2e−04
4e−04
6e−04
2011
2012
2013
2014
2015
2016
Years

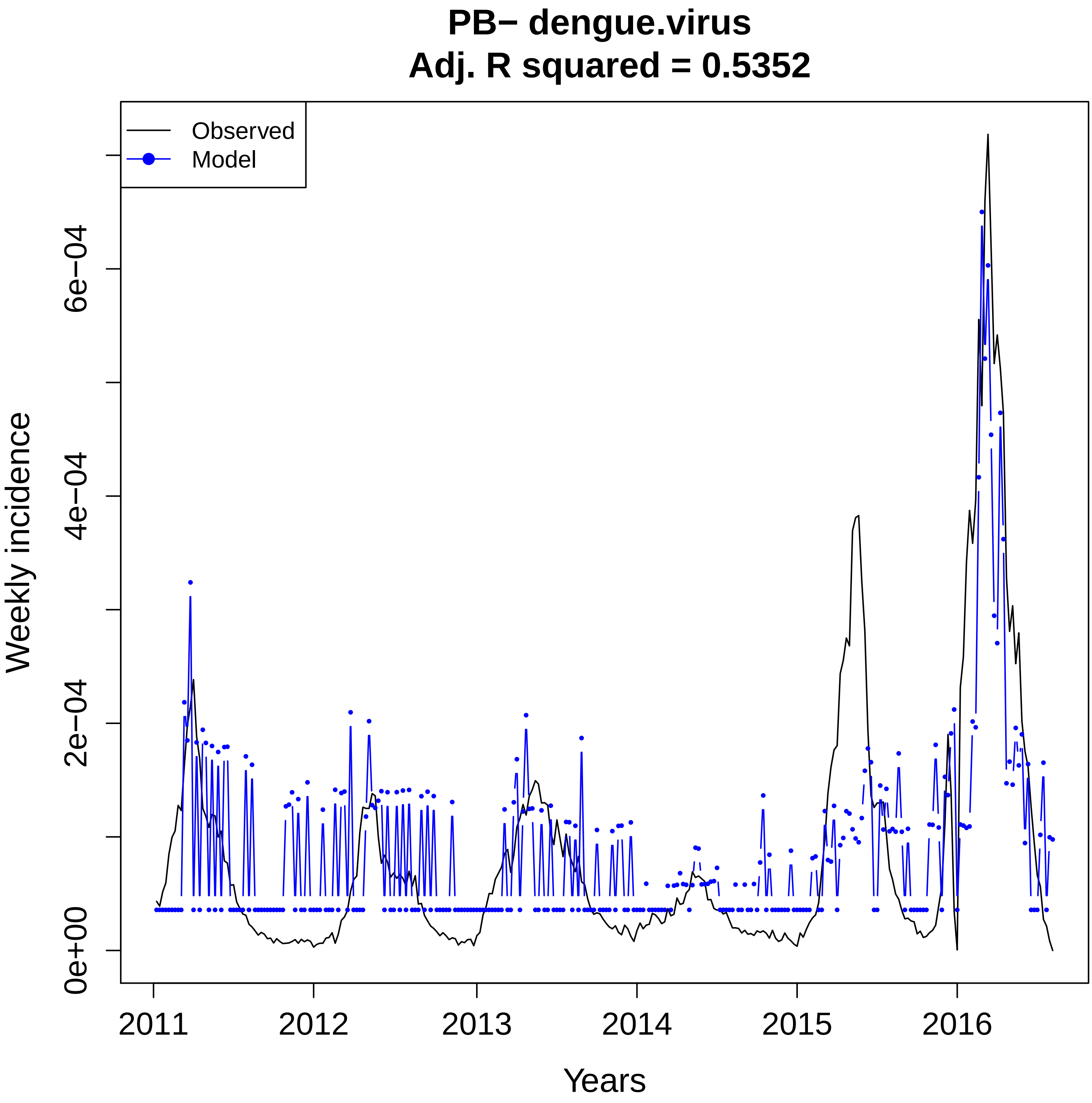
PB− dengue.virus
Adj. R squared = 0.5352
Observed
Model
Weekly incidence
0e+00
2e−04
4e−04
6e−04
Years
2011
2012
2013
2014
2015
2016

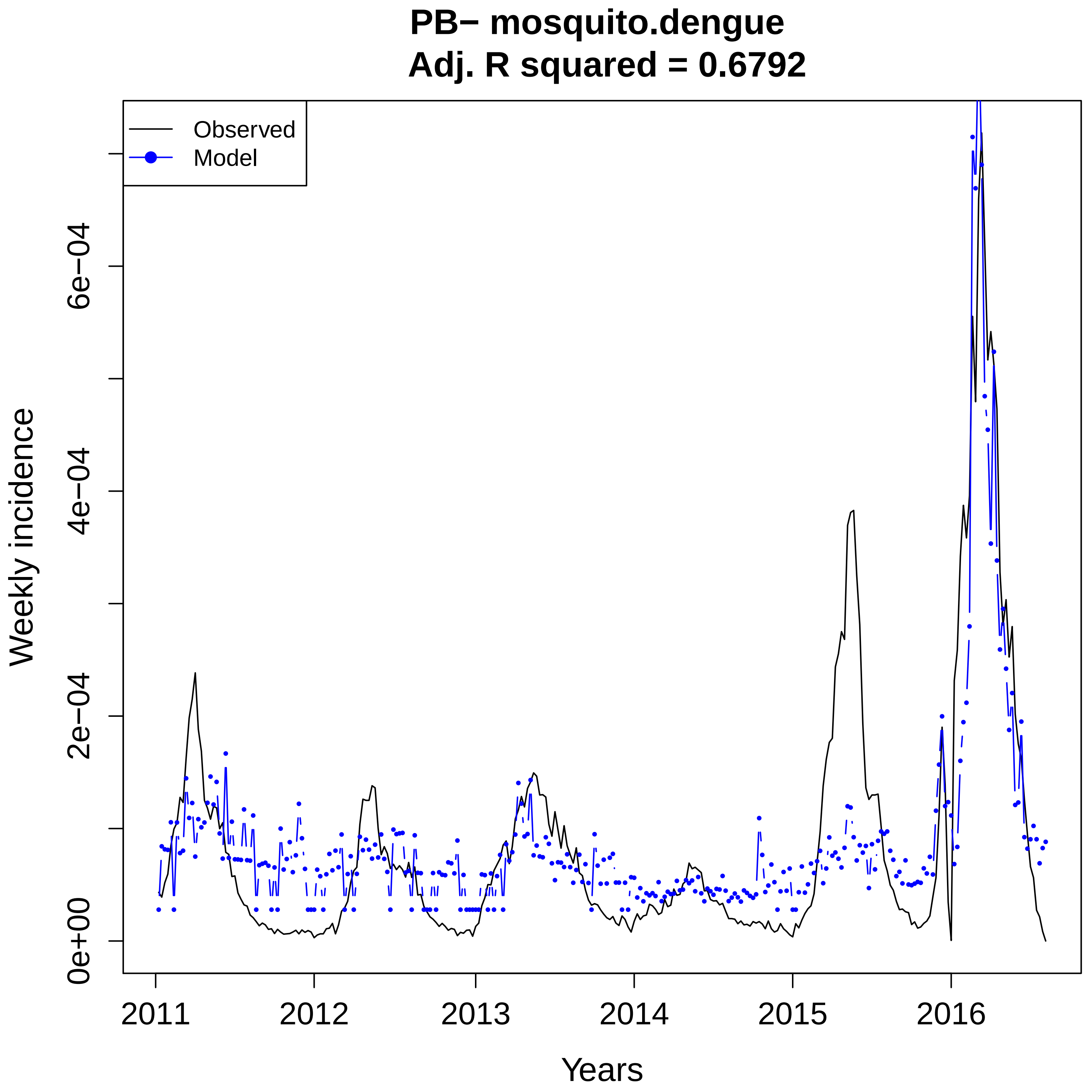
PB− mosquito.dengue
Adj. R squared = 0.6792
Observed
Model
Weekly incidence
0e+00
2e−04
4e−04
6e−04
2011
2012
2013
2014
2015
2016
Years

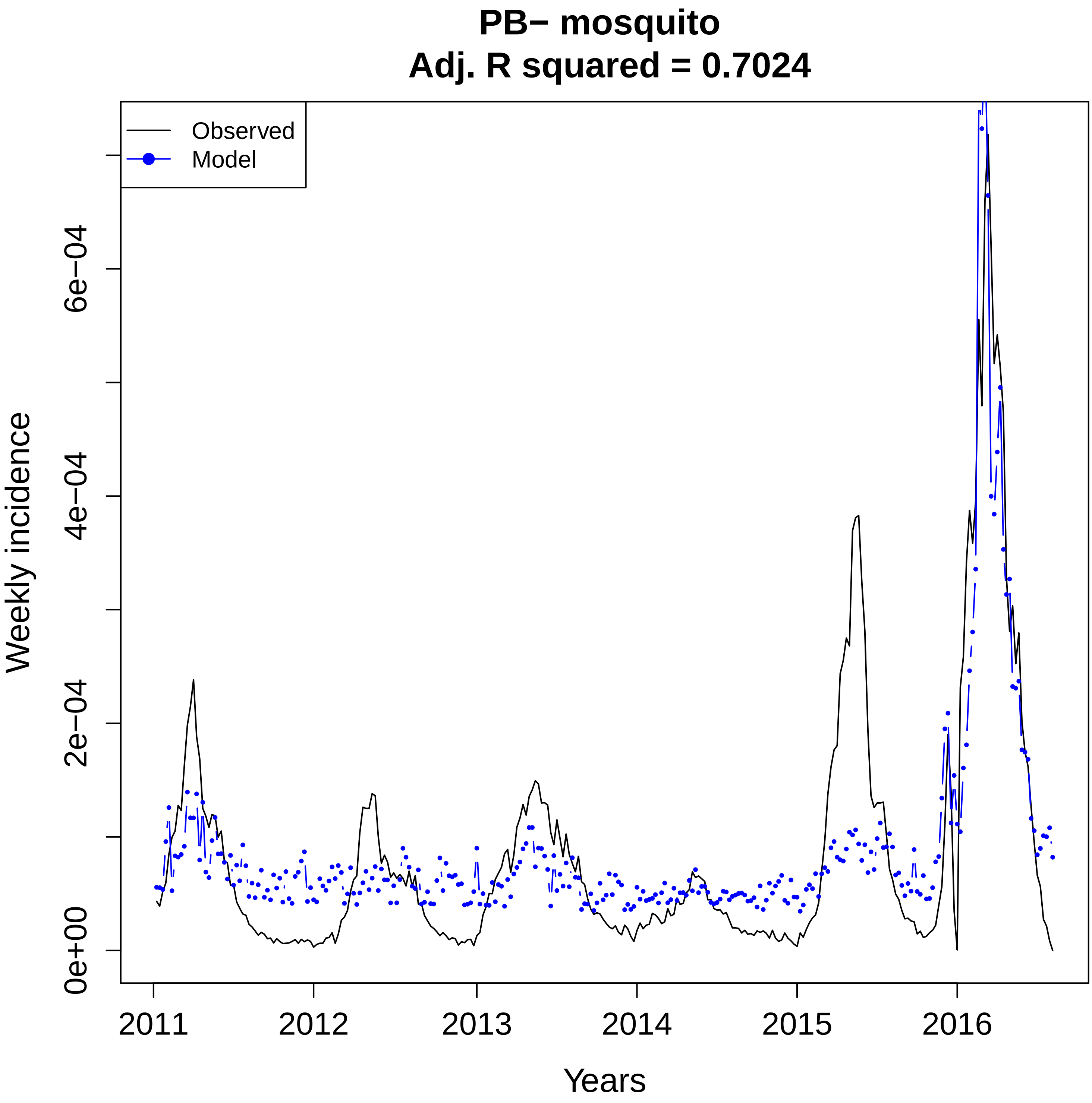
PB− mosquito
Adj. R squared = 0.7024
Observed
Model
Weekly incidence
0e+00
2e−04
4e−04
6e−04
2011
2012
2013
2014
2015
2016
Years

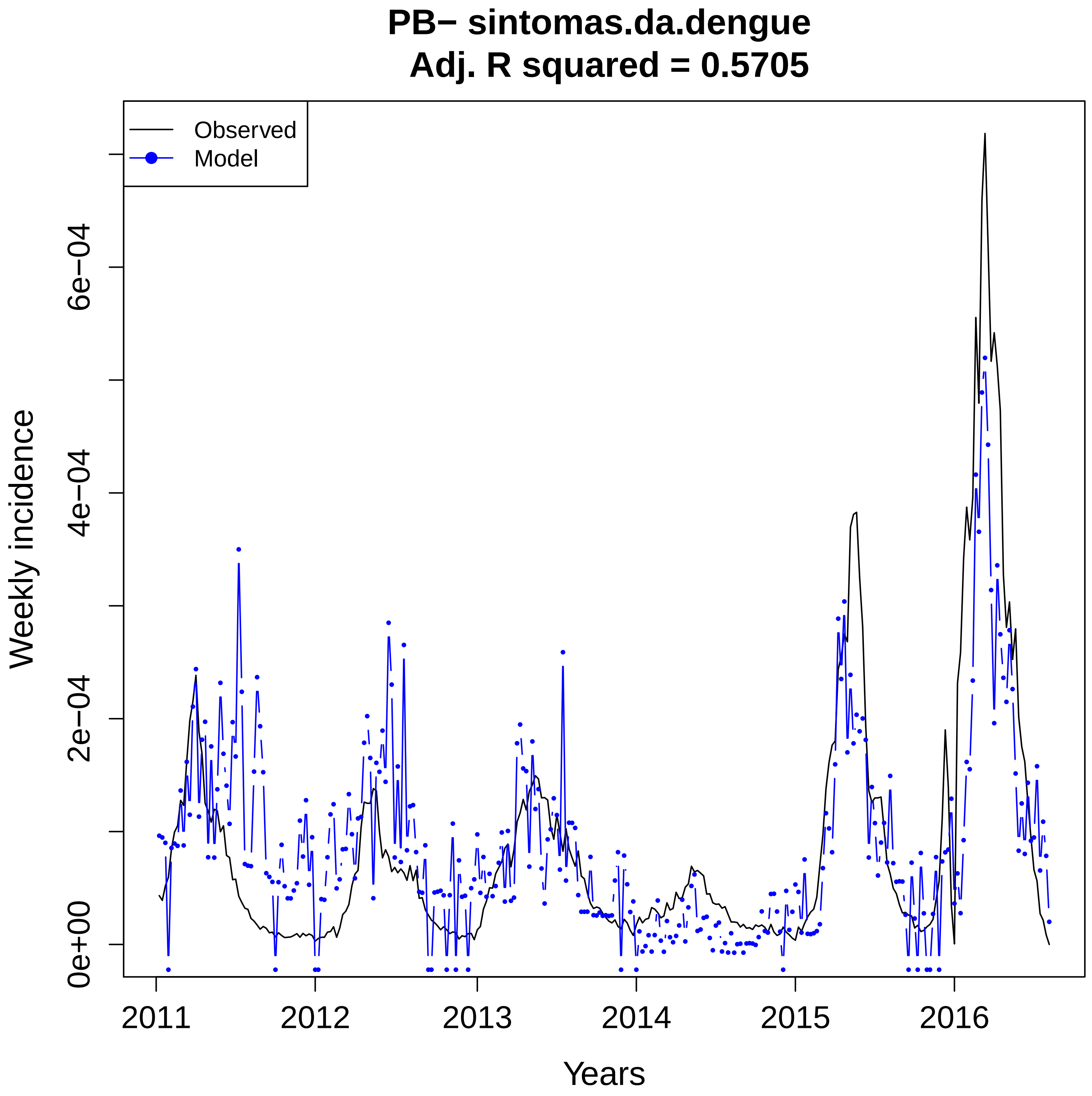
PB− sintomas.da.dengue
Adj. R squared = 0.5705
Observed
Model
Weekly incidence
0e+00
2e−04
4e−04
6e−04
2011
2012
2013
2014
2015
2016
Years

Pernambuco (PE)
Dengue incidence vs. Google Health Trends (2011–2016)

Definitions:

- **Combined terms:**
  - **All:** model with all the available terms. n= number of terms
  - **Four:** model with "dengue", "dengue sintomas", "aedes", and "mosquito".
  - **Uncorrelated:** model with non-correlated terms. n = number of uncorrelated terms
- **Individual terms:** Plots developed with one term at a time.

- Terms for all and uncorrelated models can be found in additional file 3.

Combined terms
Adjusted R squared

Pernambuco (PE)

All
(n = 10)

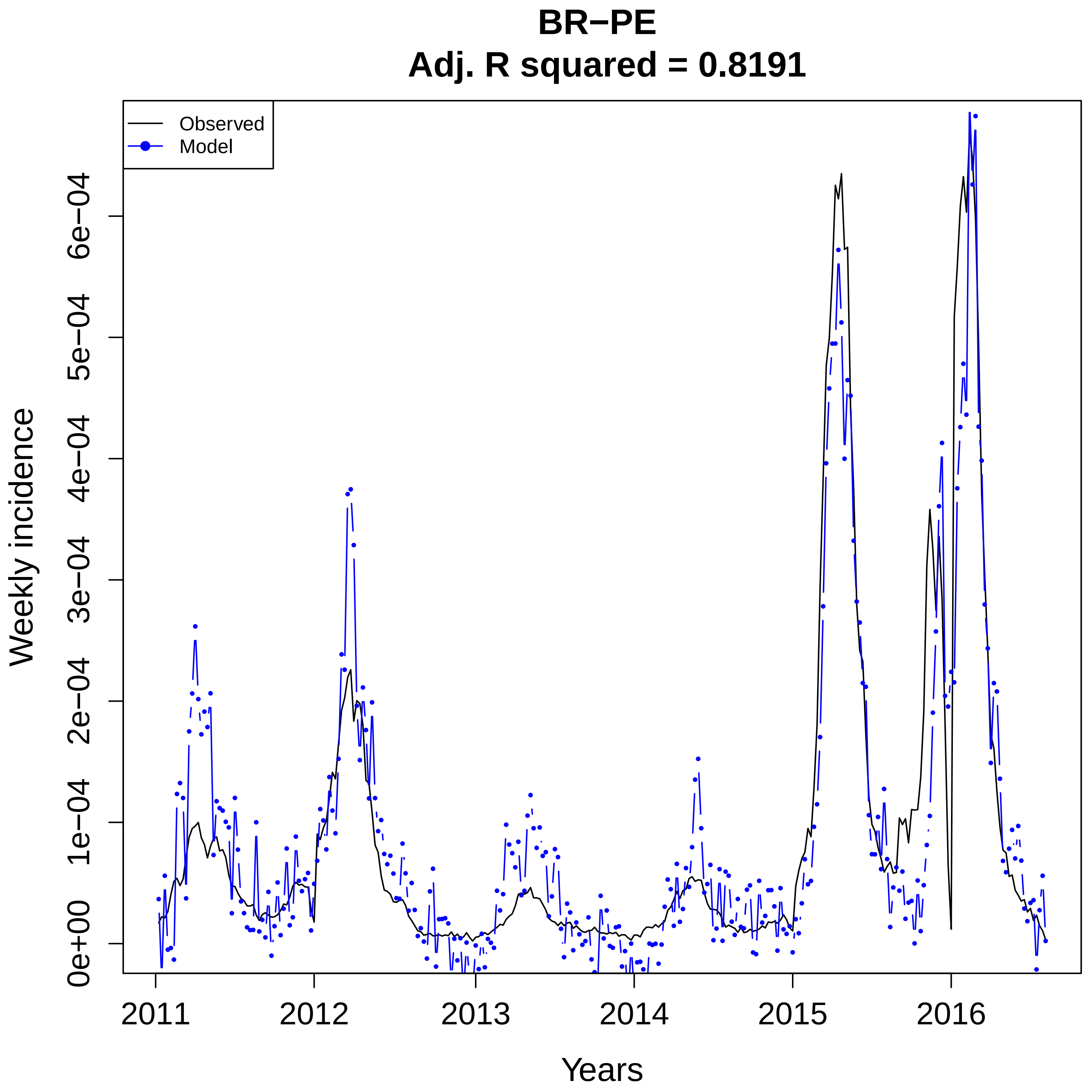

BR−PE
Adj. R squared = 0.8191
Observed
Model
Weekly incidence
0e+00
1e−04
2e−04
3e−04
4e−04
5e−04
6e−04
2011
2012
2013
2014
2015
2016
Years

Four

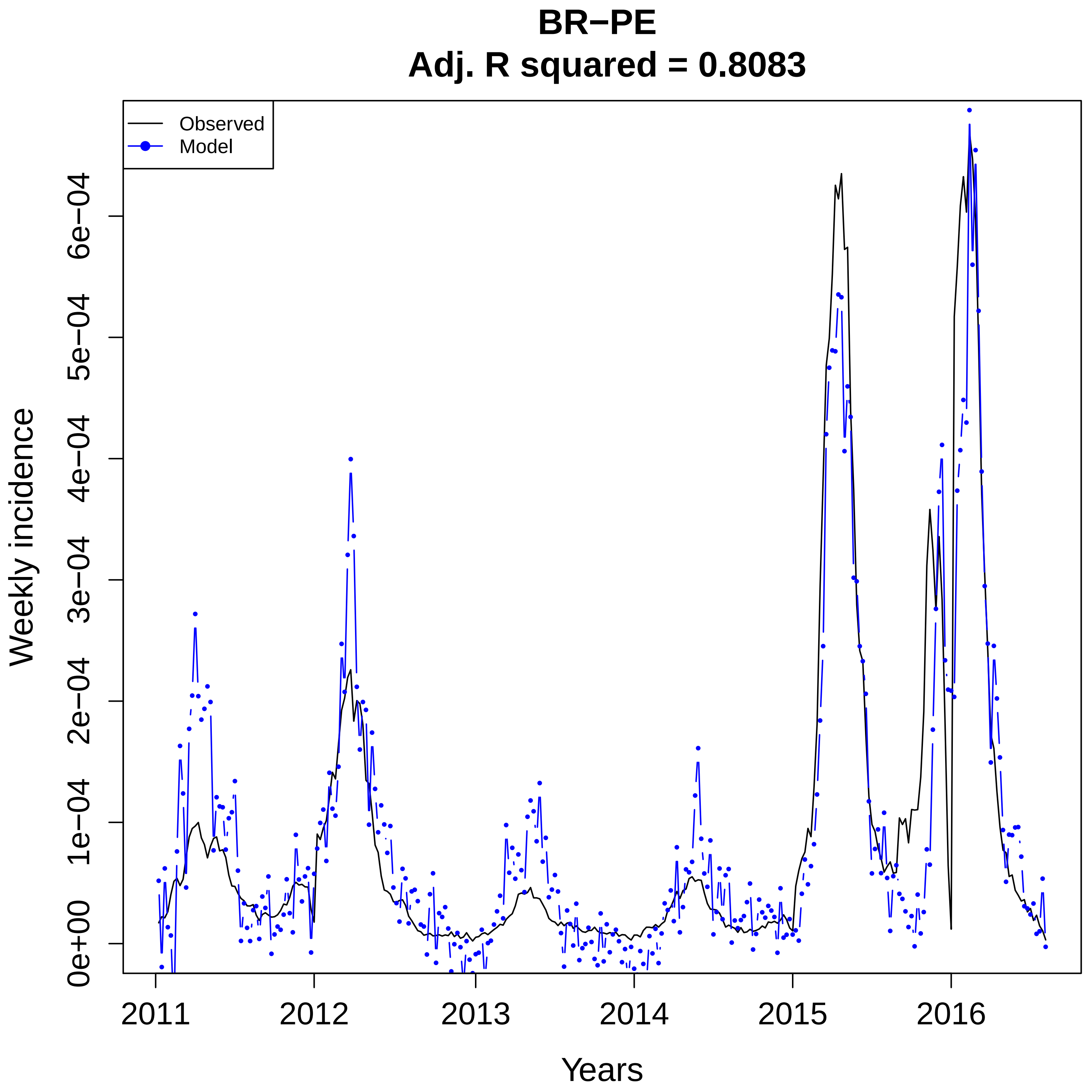

BR−PE
Adj. R squared = 0.8083
Observed
Model
Weekly incidence
0e+00
1e−04
2e−04
3e−04
4e−04
5e−04
6e−04
2011
2012
2013
2014
2015
2016
Years

Uncorrelated
(n = 2)

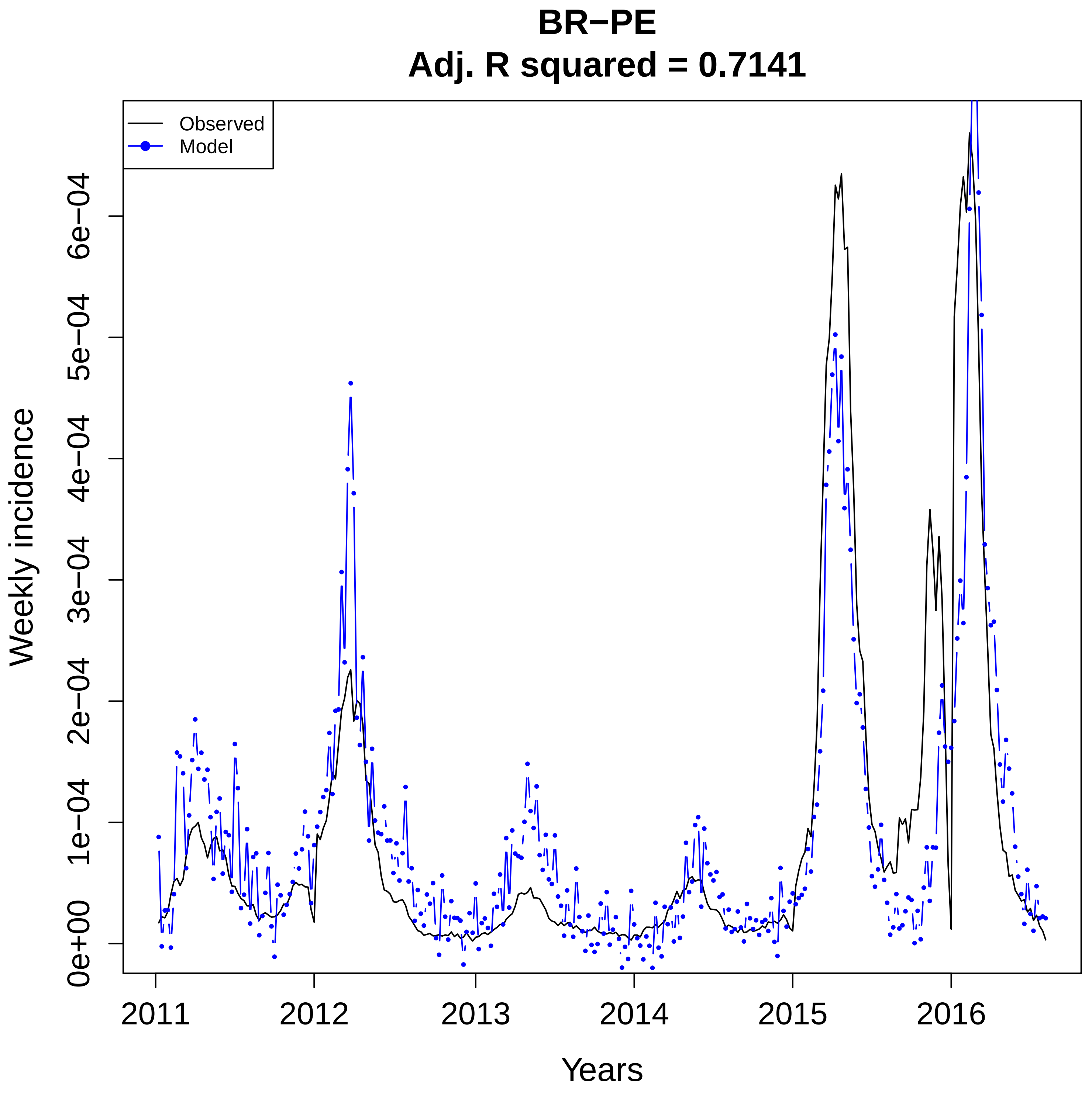

Individual terms
Adjusted R squared

Pernambuco (PE)

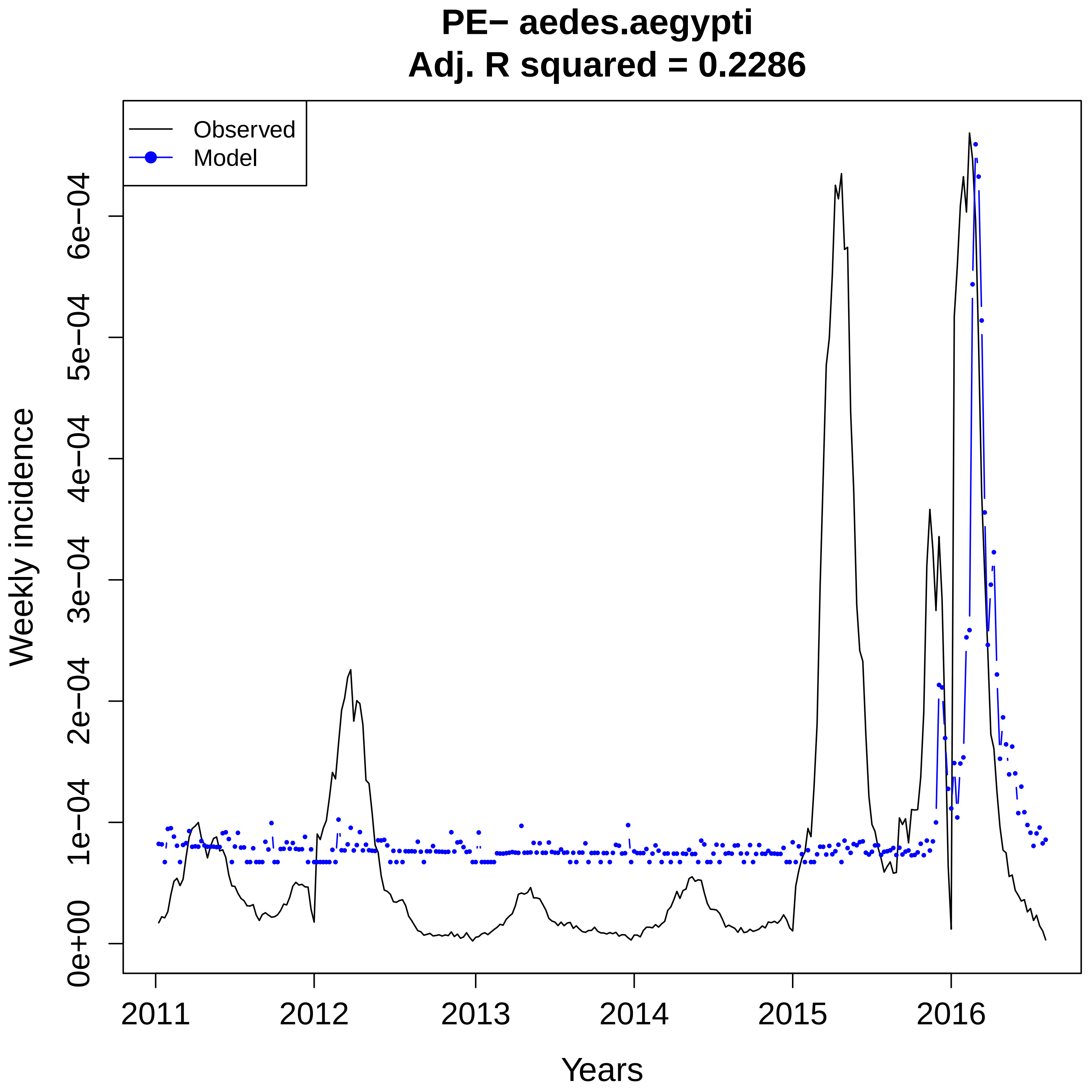
PE− aedes.aegypti
Adj. R squared = 0.2286
Observed
Model
Weekly incidence
0e+00
1e−04
2e−04
3e−04
4e−04
5e−04
6e−04
2011
2012
2013
2014
2015
2016
Years

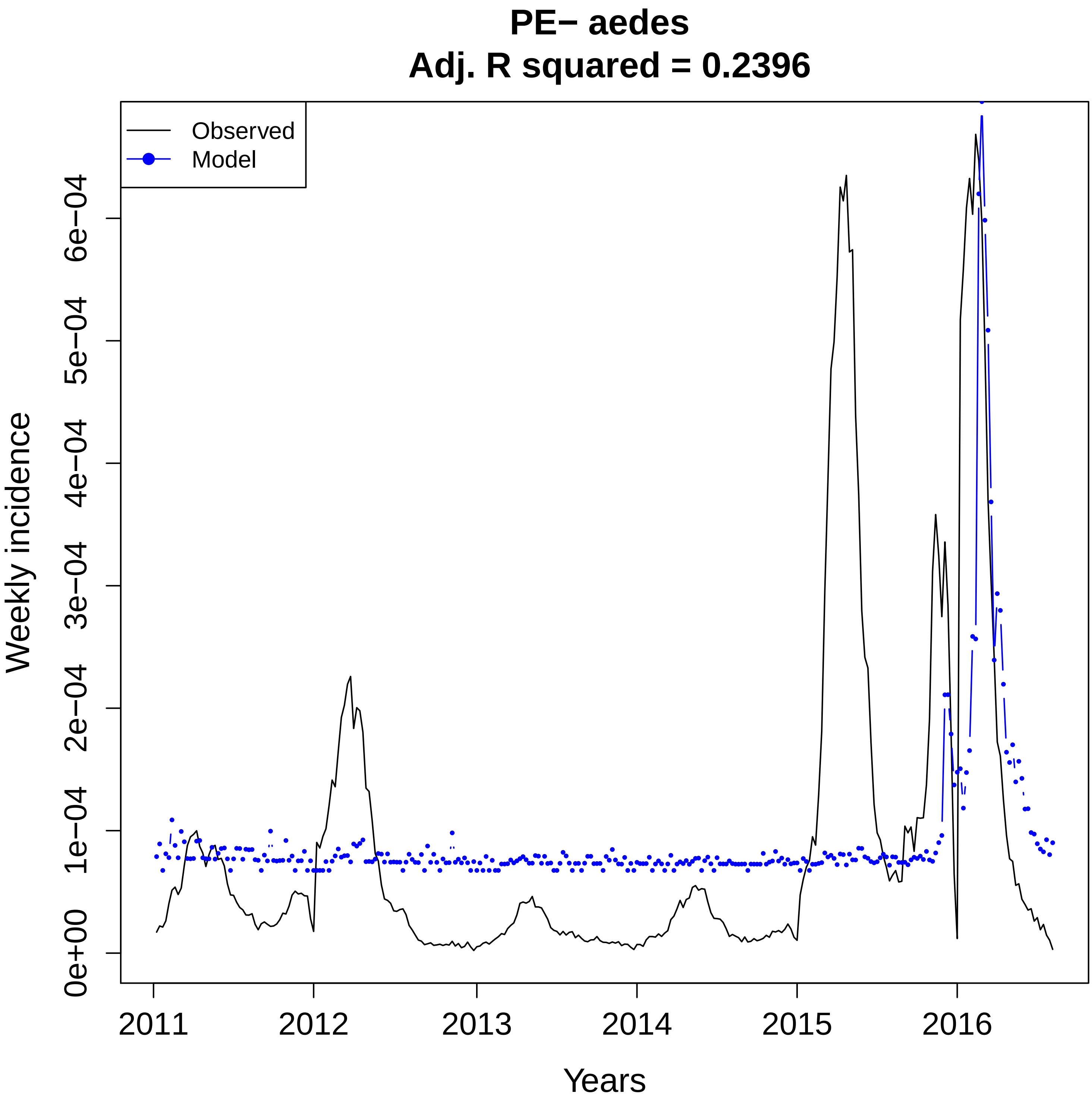
PE− aedes
Adj. R squared = 0.2396
Observed
Model
Weekly incidence
0e+00
1e−04
2e−04
3e−04
4e−04
5e−04
6e−04
2011
2012
2013
2014
2015
2016
Years

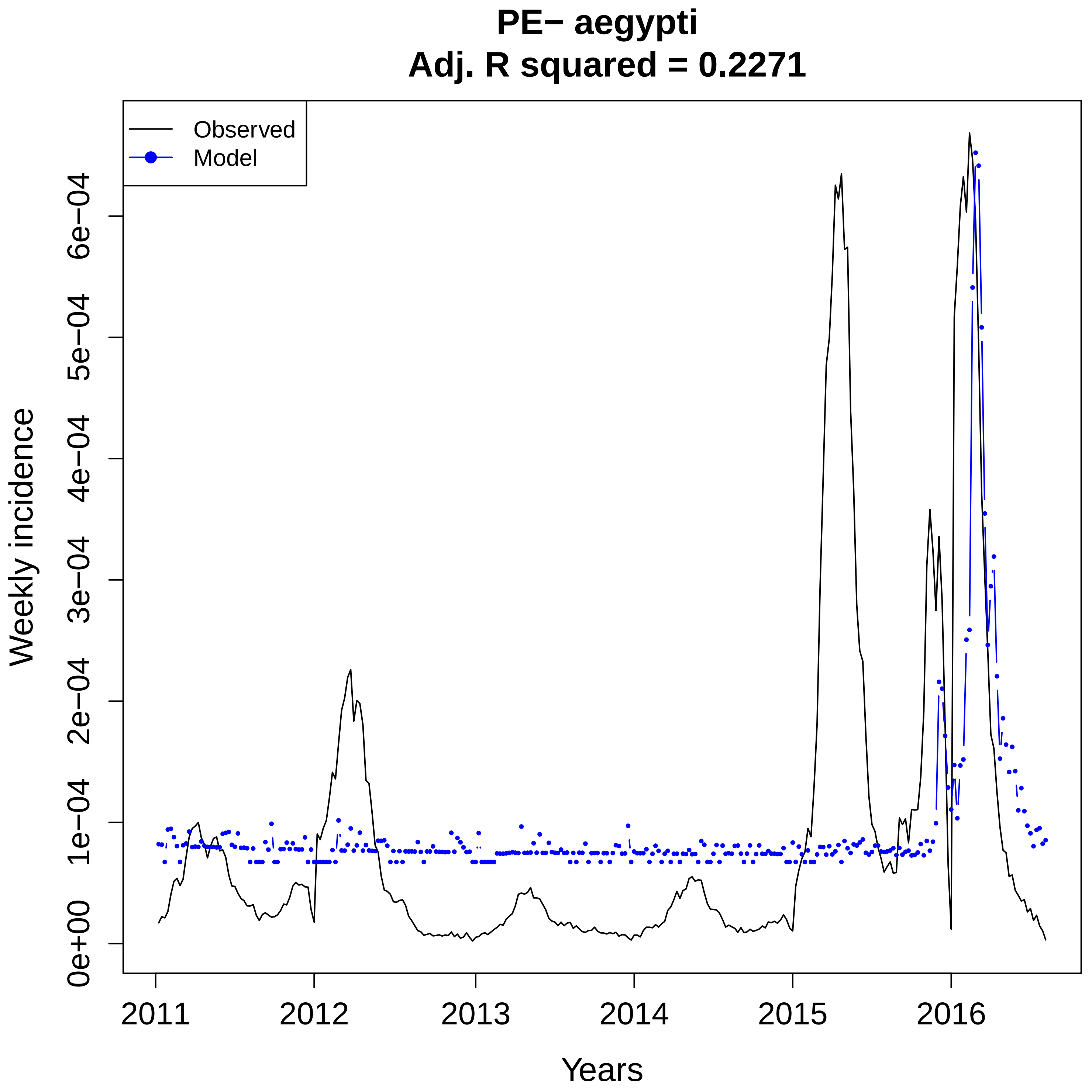

PE− aegypti
Adj. R squared = 0.2271
Observed
Model
Weekly incidence
0e+00
1e−04
2e−04
3e−04
4e−04
5e−04
6e−04
2011
2012
2013
2014
2015
2016
Years

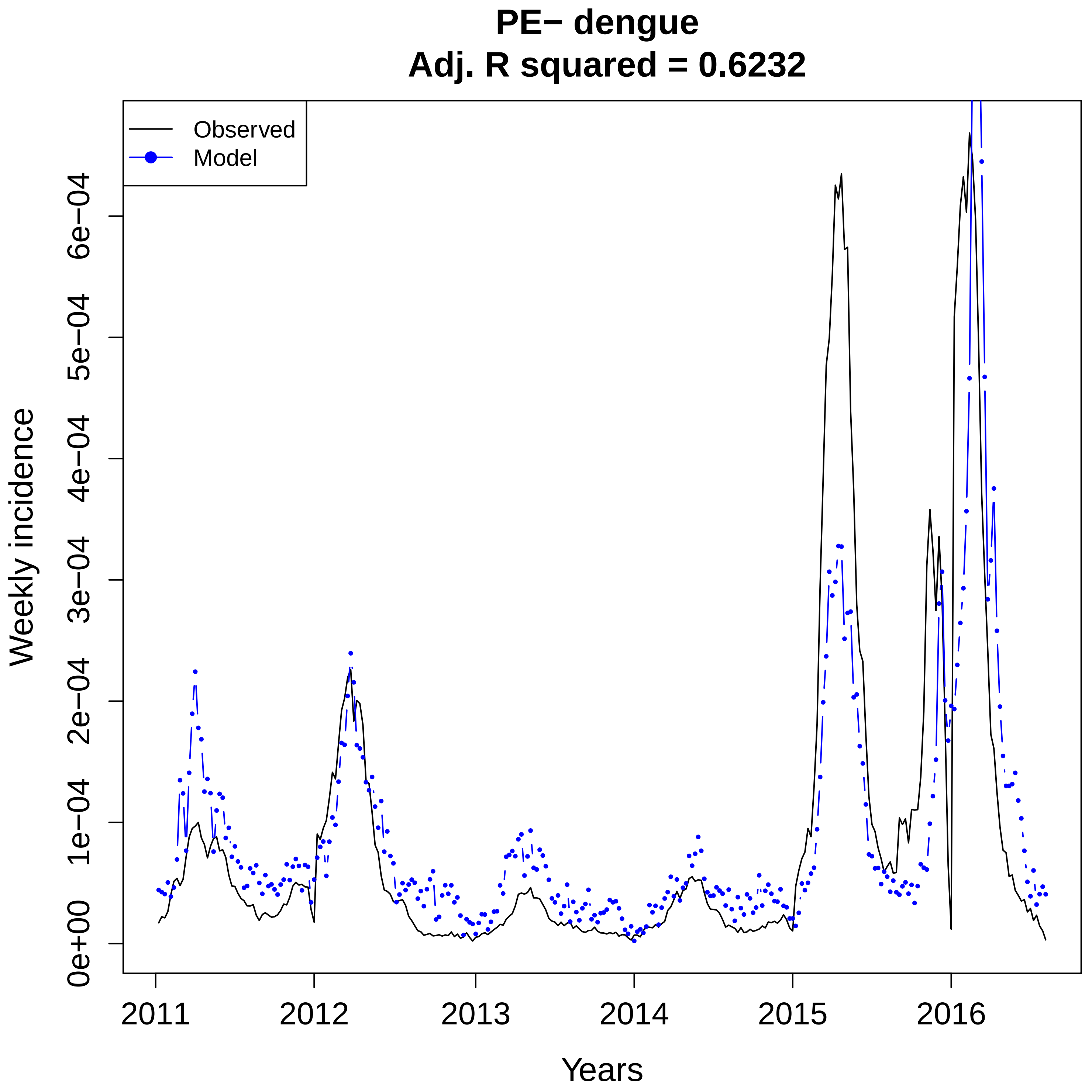
PE− dengue
Adj. R squared = 0.6232
Observed
Model
Weekly incidence
0e+00
1e−04
2e−04
3e−04
4e−04
5e−04
6e−04
2011
2012
2013
2014
2015
2016
Years

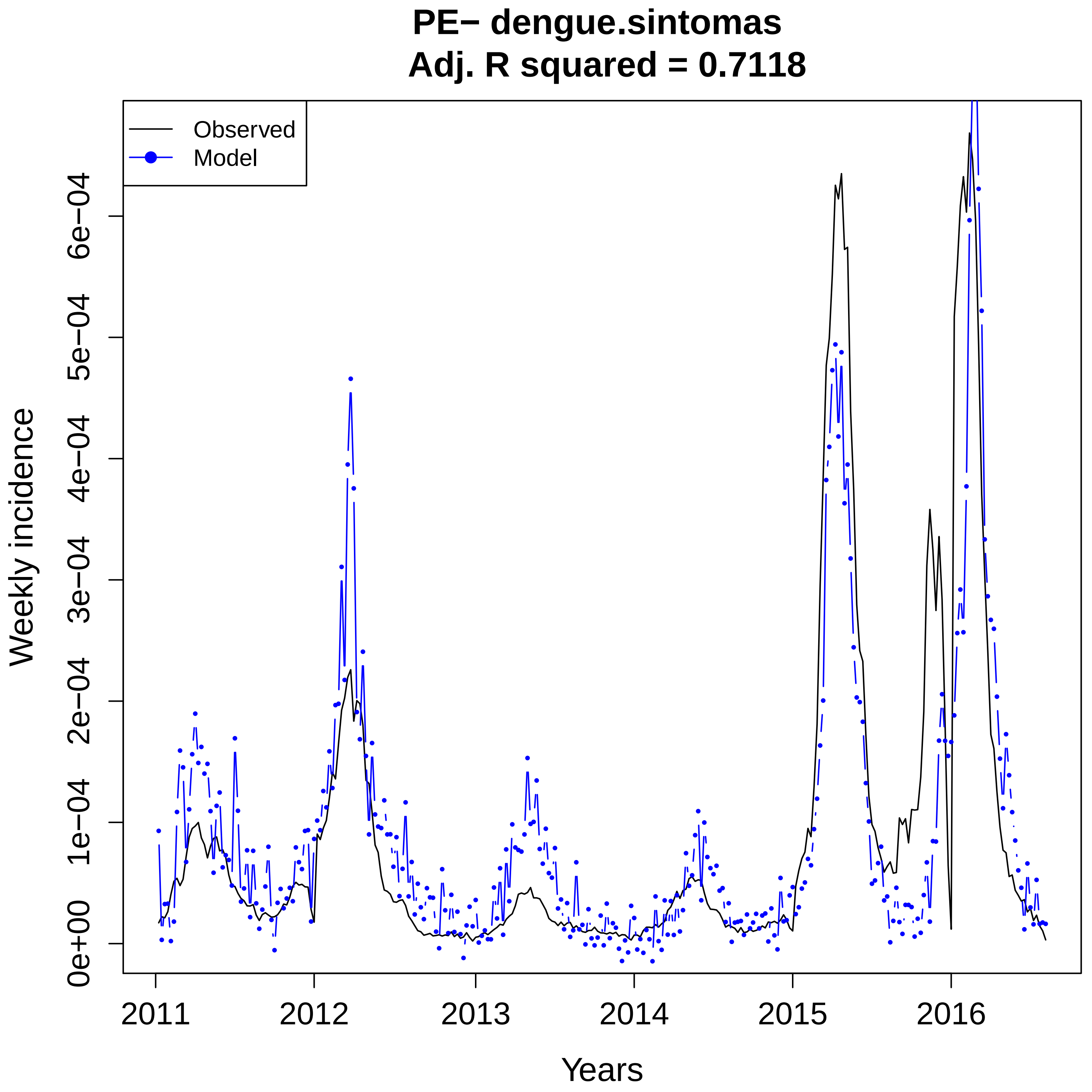
PE− dengue.sintomas
Adj. R squared = 0.7118
Observed
Model
Weekly incidence
0e+00
1e−04
2e−04
3e−04
4e−04
5e−04
6e−04
2011
2012
2013
2014
2015
2016
Years

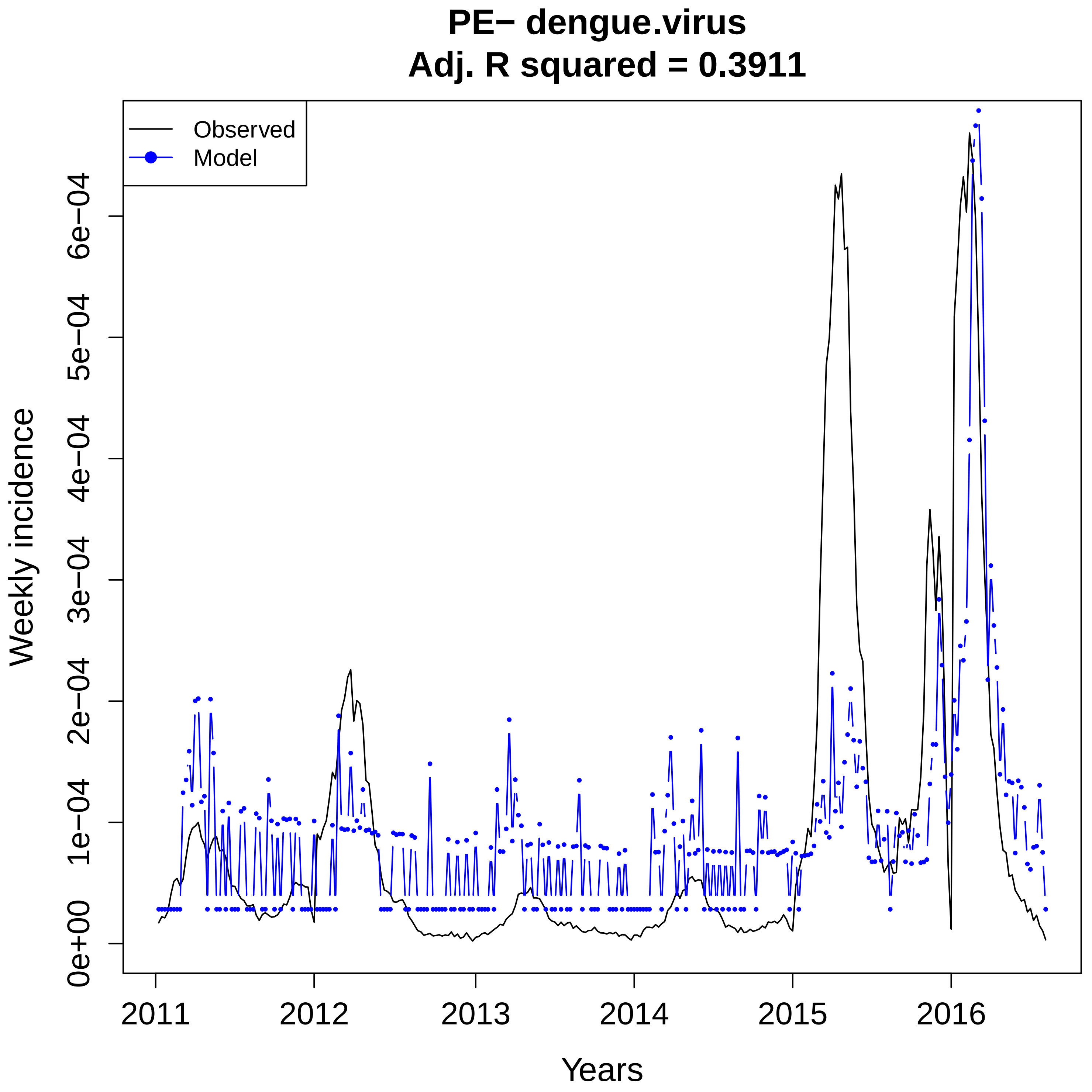
PE− dengue.virus
Adj. R squared = 0.3911
Observed
Model
Weekly incidence
0e+00
1e−04
2e−04
3e−04
4e−04
5e−04
6e−04
2011
2012
2013
2014
2015
2016
Years

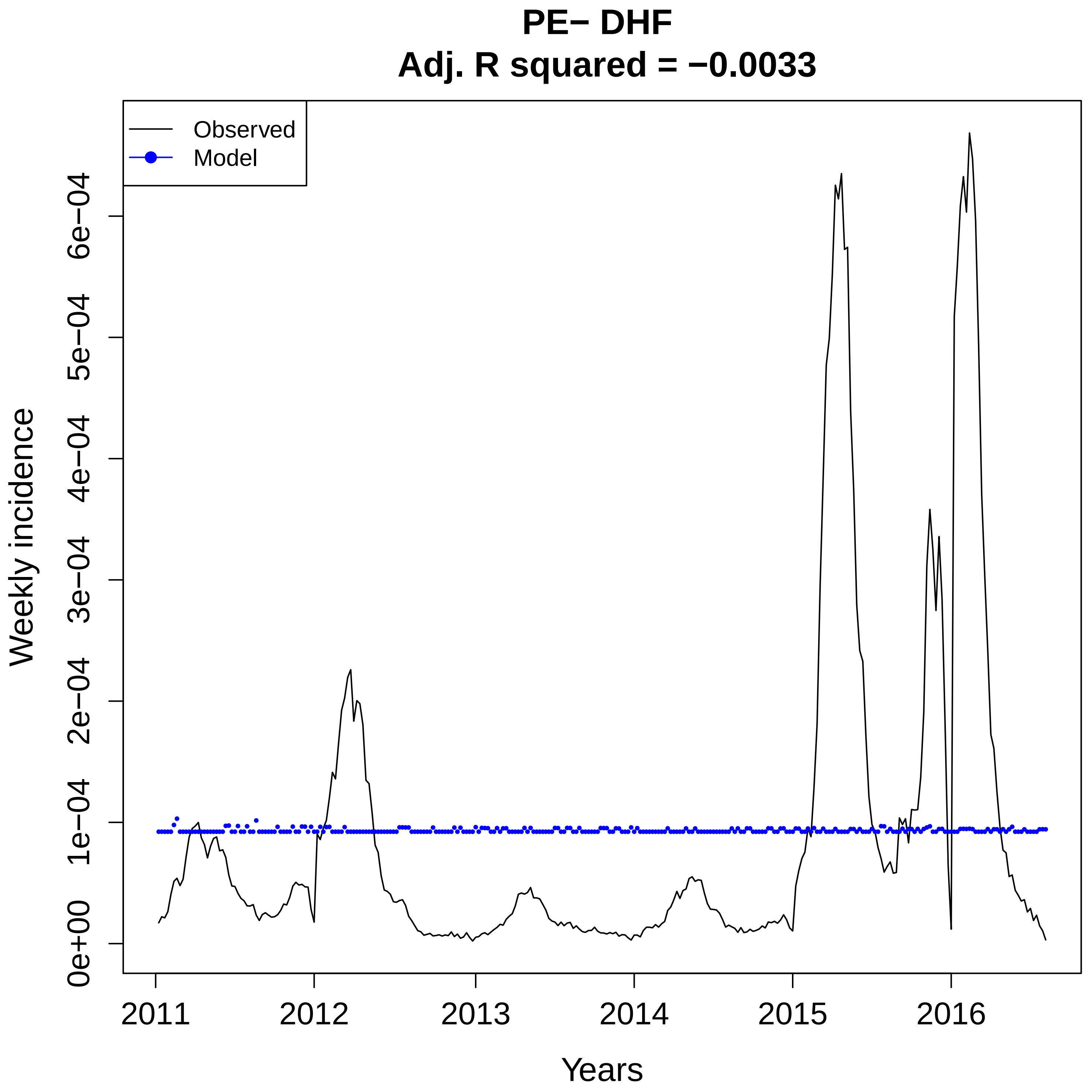
PE− DHF
Adj. R squared = −0.0033
Observed
Model
Weekly incidence
0e+00
1e−04
2e−04
3e−04
4e−04
5e−04
6e−04
2011
2012
2013
2014
2015
2016
Years

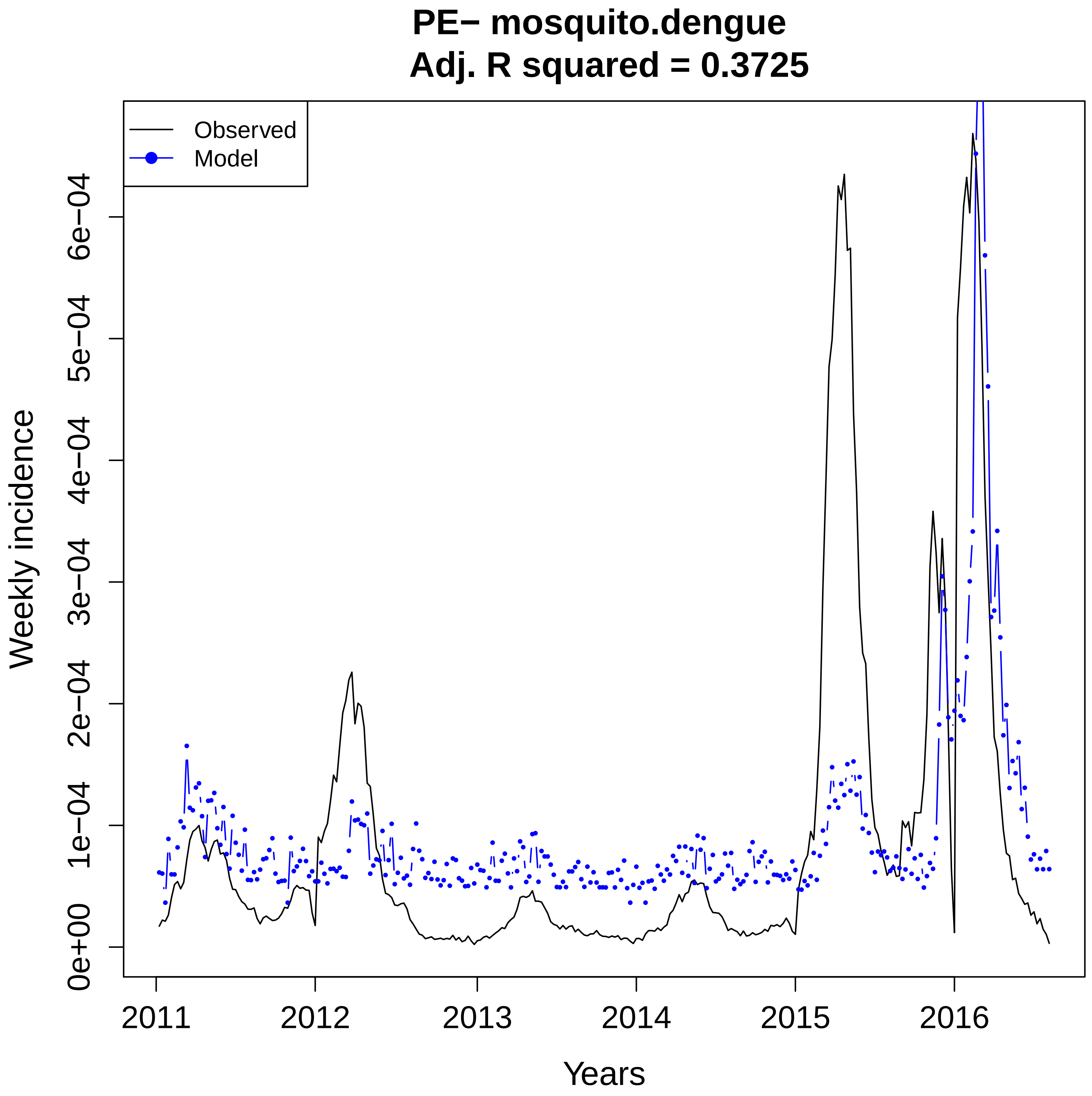
PE− mosquito.dengue
Adj. R squared = 0.3725
Observed
Model
Weekly incidence
0e+00
1e−04
2e−04
3e−04
4e−04
5e−04
6e−04
2011
2012
2013
2014
2015
2016
Years

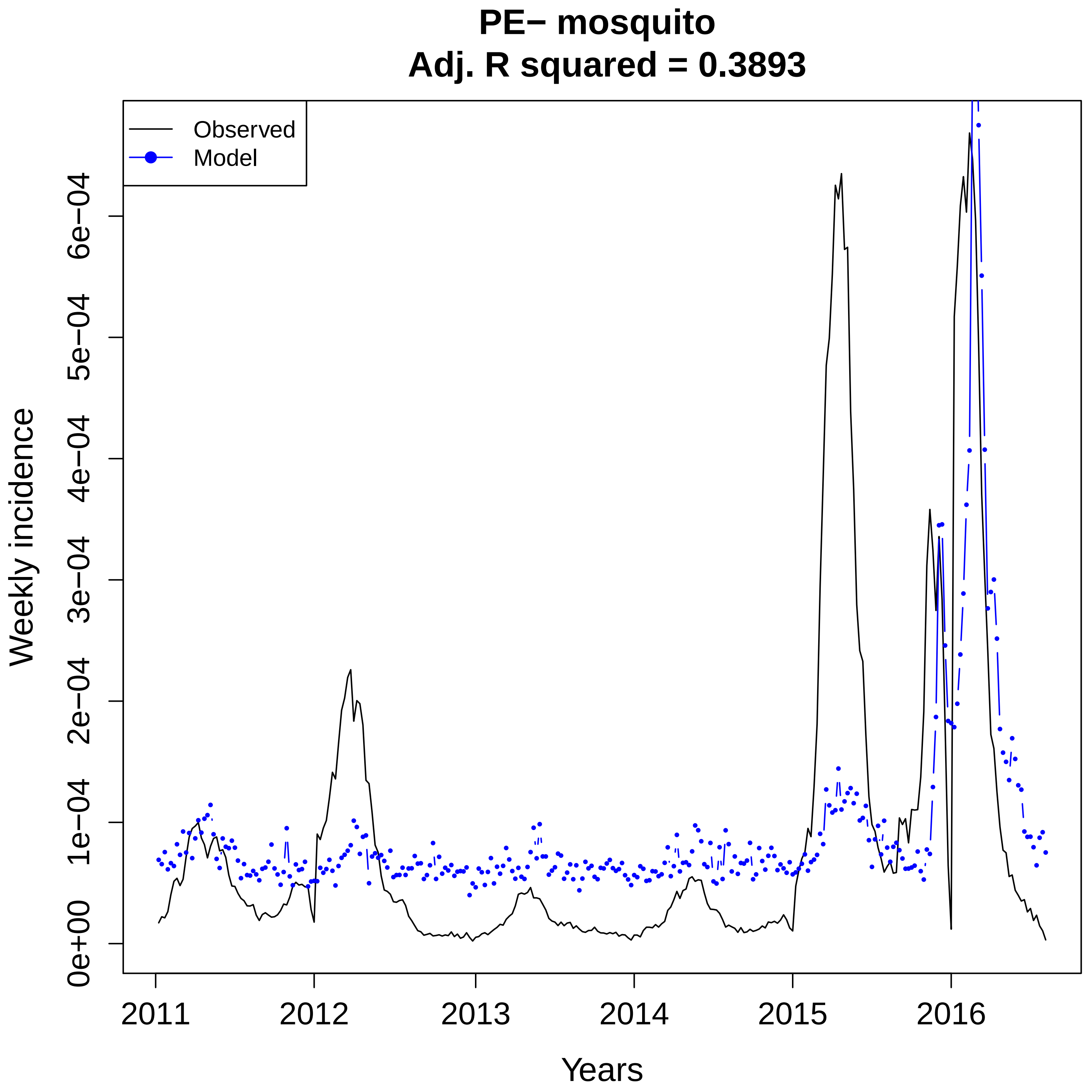

PE− mosquito
Adj. R squared = 0.3893
Observed
Model
Weekly incidence
0e+00
1e−04
2e−04
3e−04
4e−04
5e−04
6e−04
2011
2012
2013
2014
2015
2016
Years

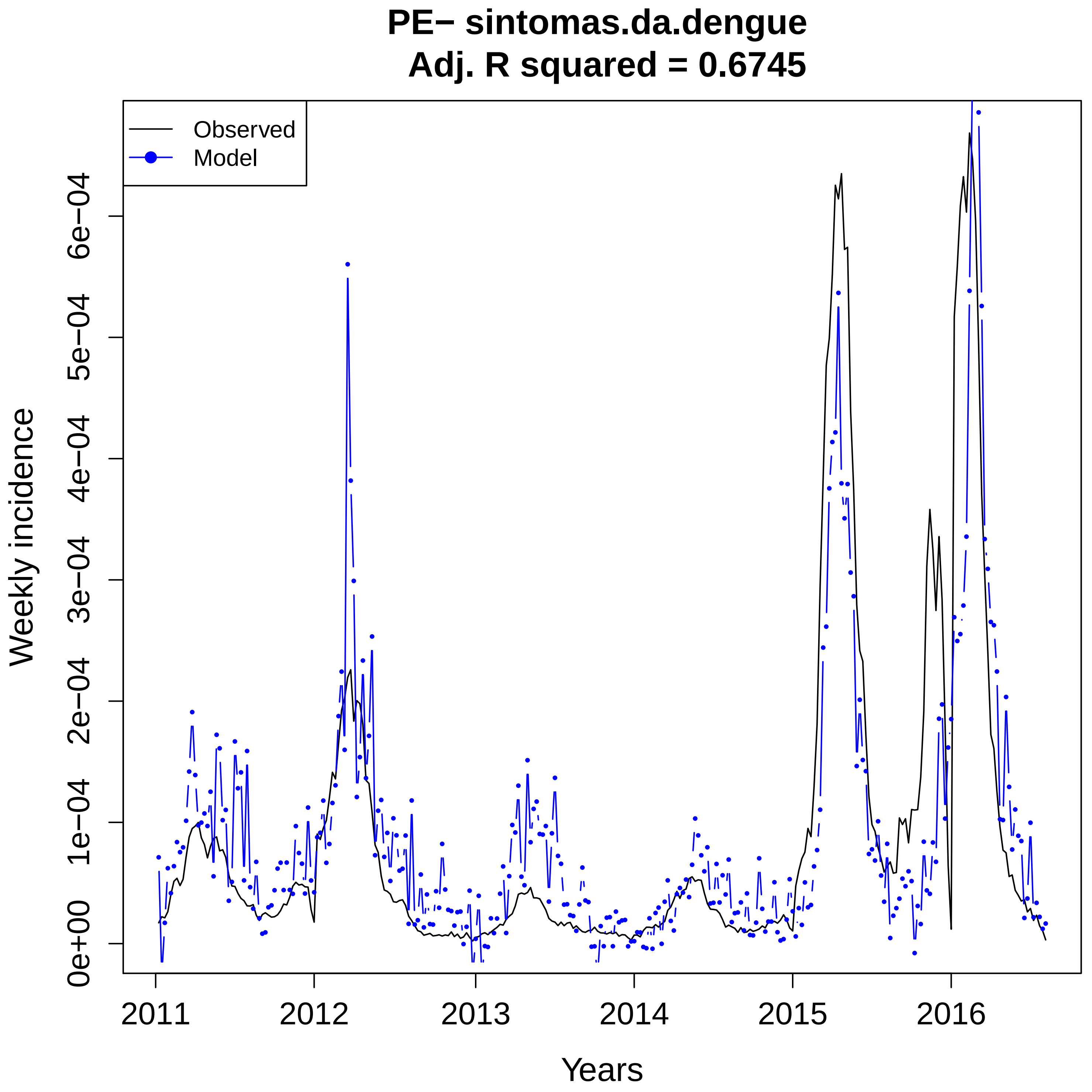

PE− sintomas.da.dengue
Adj. R squared = 0.6745
Observed
Model
Weekly incidence
0e+00
1e−04
2e−04
3e−04
4e−04
5e−04
6e−04
2011
2012
2013
2014
2015
2016
Years

Piauí (PI)
Dengue incidence vs. Google Health Trends (2011–2016)

Definitions:

- **Combined terms:**
    - **All:** model with all the available terms. n= number of terms
    - **Four:** model with "dengue", "dengue sintomas", "aedes", and "mosquito".
    - **Uncorrelated:** model with non-correlated terms. n = number of uncorrelated terms
- **Individual terms:** Plots developed with one term at a time.

- Terms for all and uncorrelated models can be found in additional file 3.

Combined terms
Adjusted R squared

Piauí (PI)

All
(n = 8)

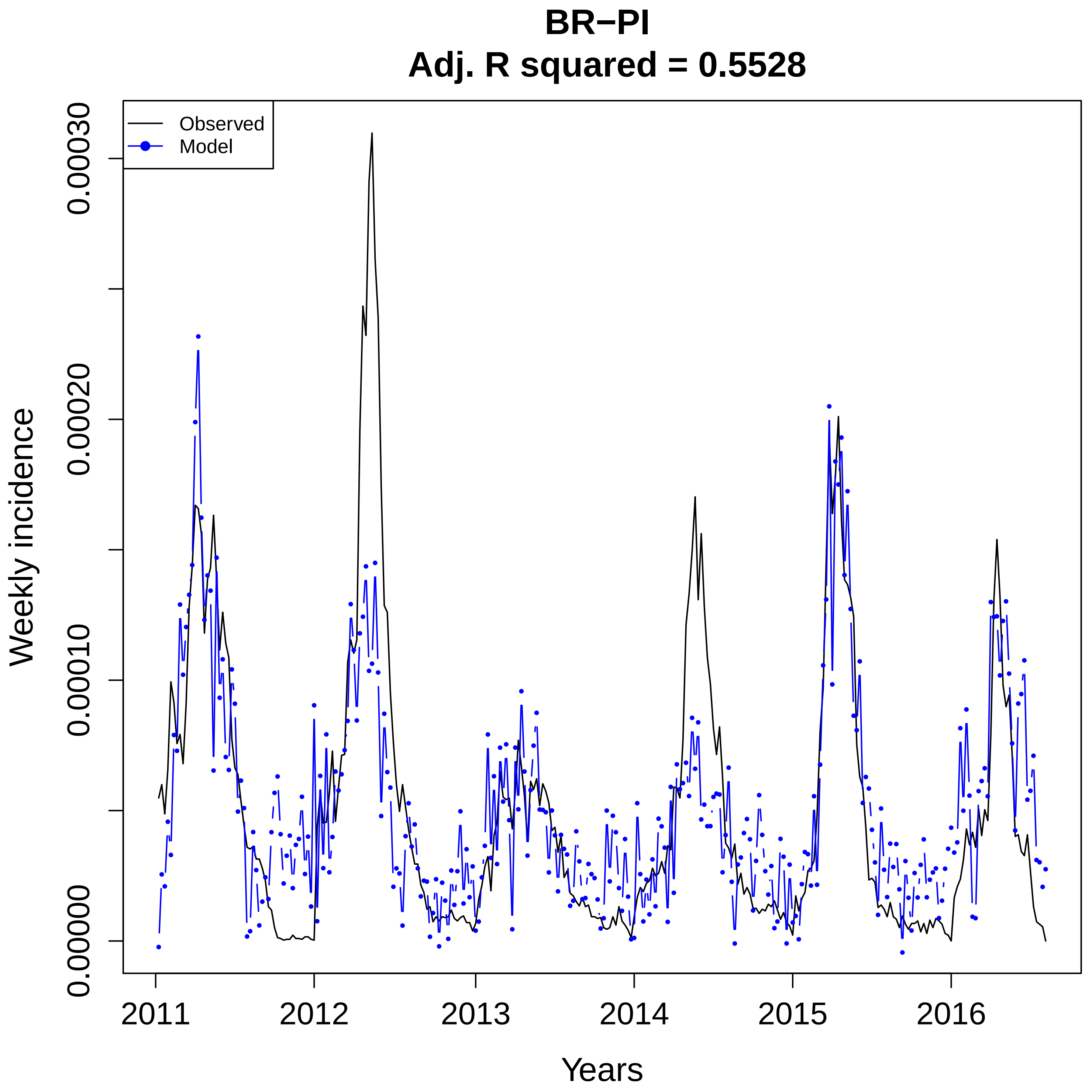
BR−PI
Adj. R squared = 0.5528
Observed
Model
Weekly incidence
0.00000
0.00010
0.00020
0.00030
2011
2012
2013
2014
2015
2016
Years

Four

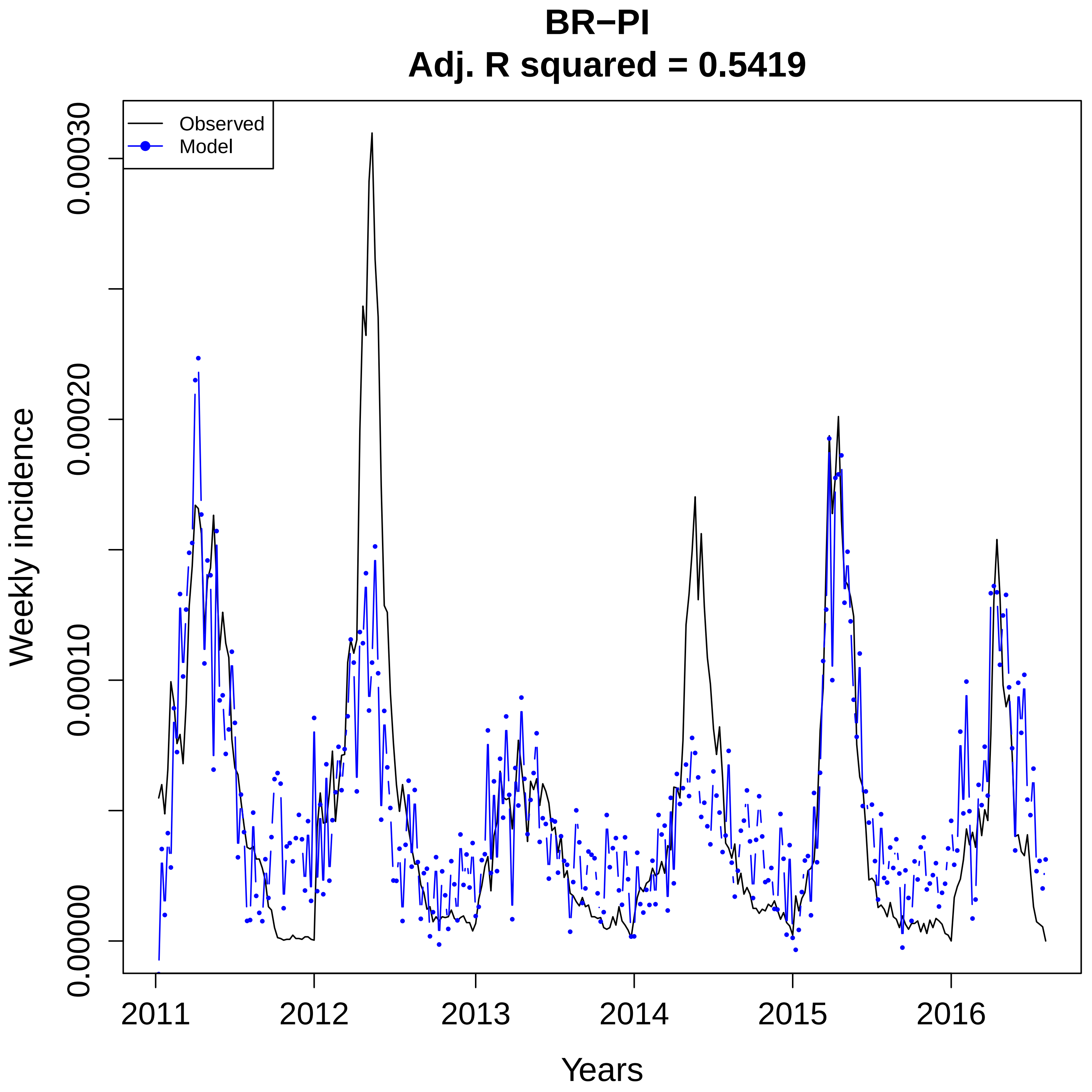

BR−PI
Adj. R squared = 0.5419
Observed
Model
Weekly incidence
0.00000
0.00010
0.00020
0.00030
2011
2012
2013
2014
2015
2016
Years

Uncorrelated (n = 3)

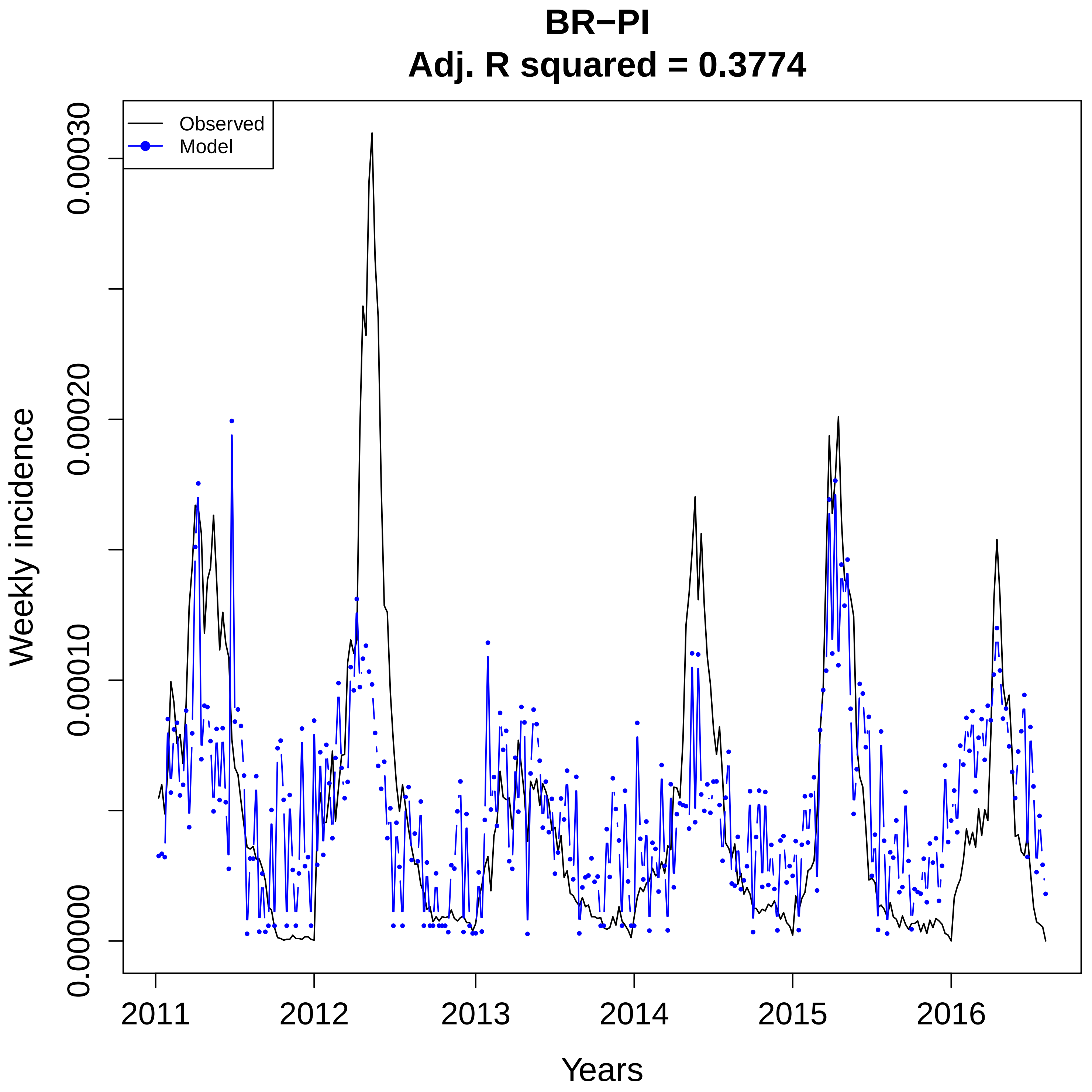

Individual terms
Adjusted R squared

Piauí (PI)

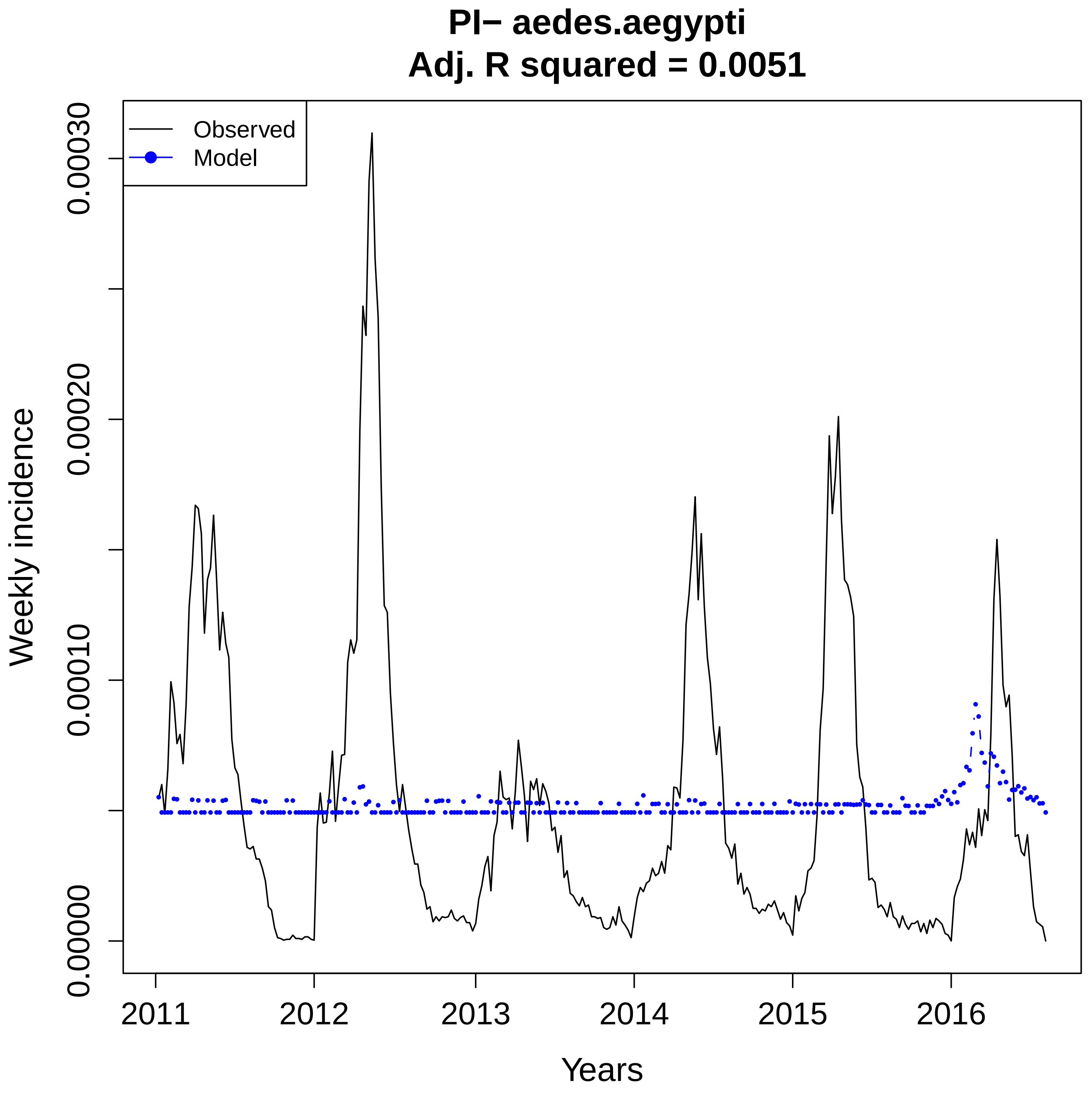

PI− aedes.aegypti
Adj. R squared = 0.0051
Observed
Model
Weekly incidence
0.00000
0.00010
0.00020
0.00030
2011
2012
2013
2014
2015
2016
Years

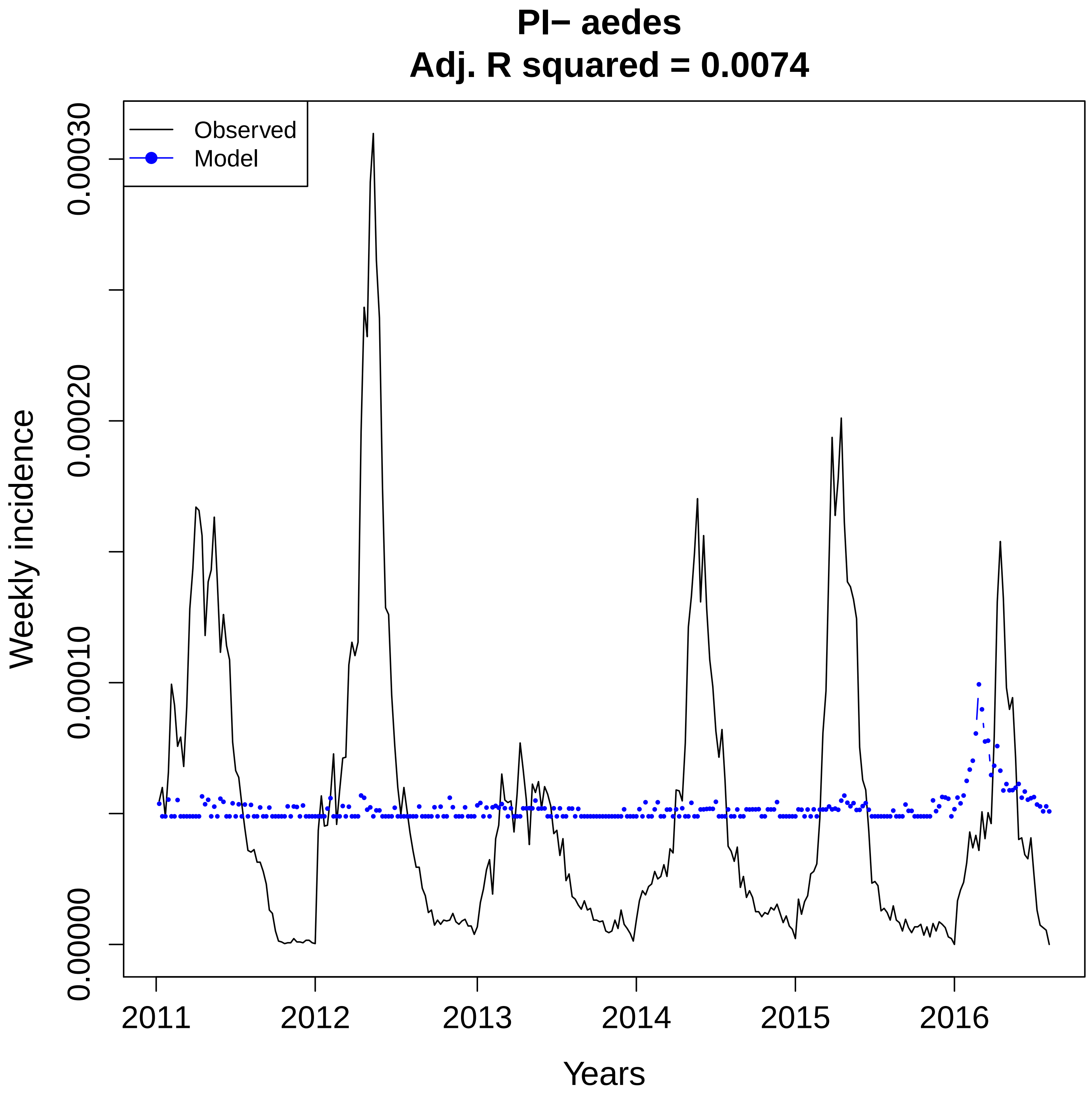
PI− aedes
Adj. R squared = 0.0074
Observed
Model
Weekly incidence
0.00000
0.00010
0.00020
0.00030
2011
2012
2013
2014
2015
2016
Years

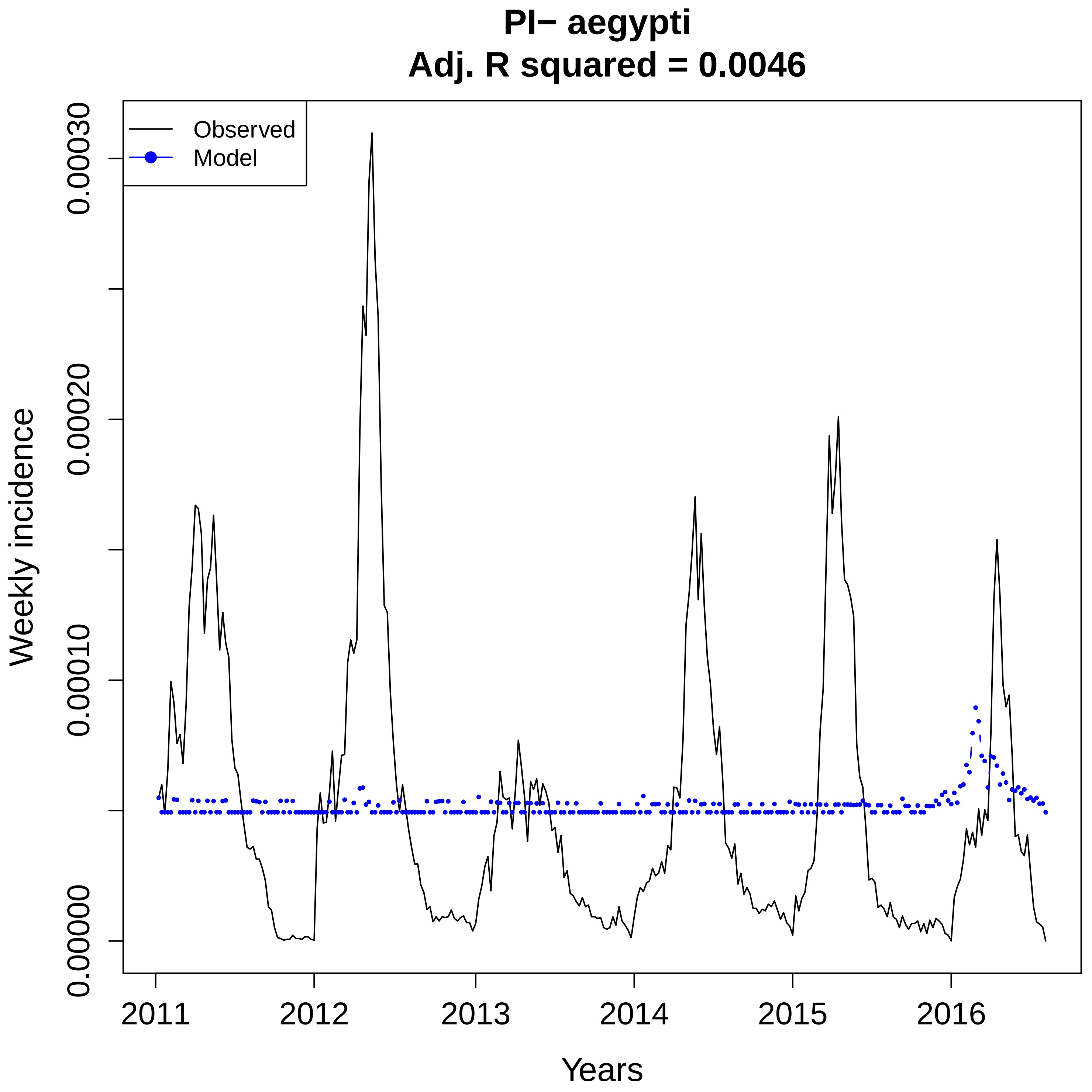
PI− aegypti
Adj. R squared = 0.0046
Observed
Model
Weekly incidence
0.00000
0.00010
0.00020
0.00030
2011
2012
2013
2014
2015
2016
Years

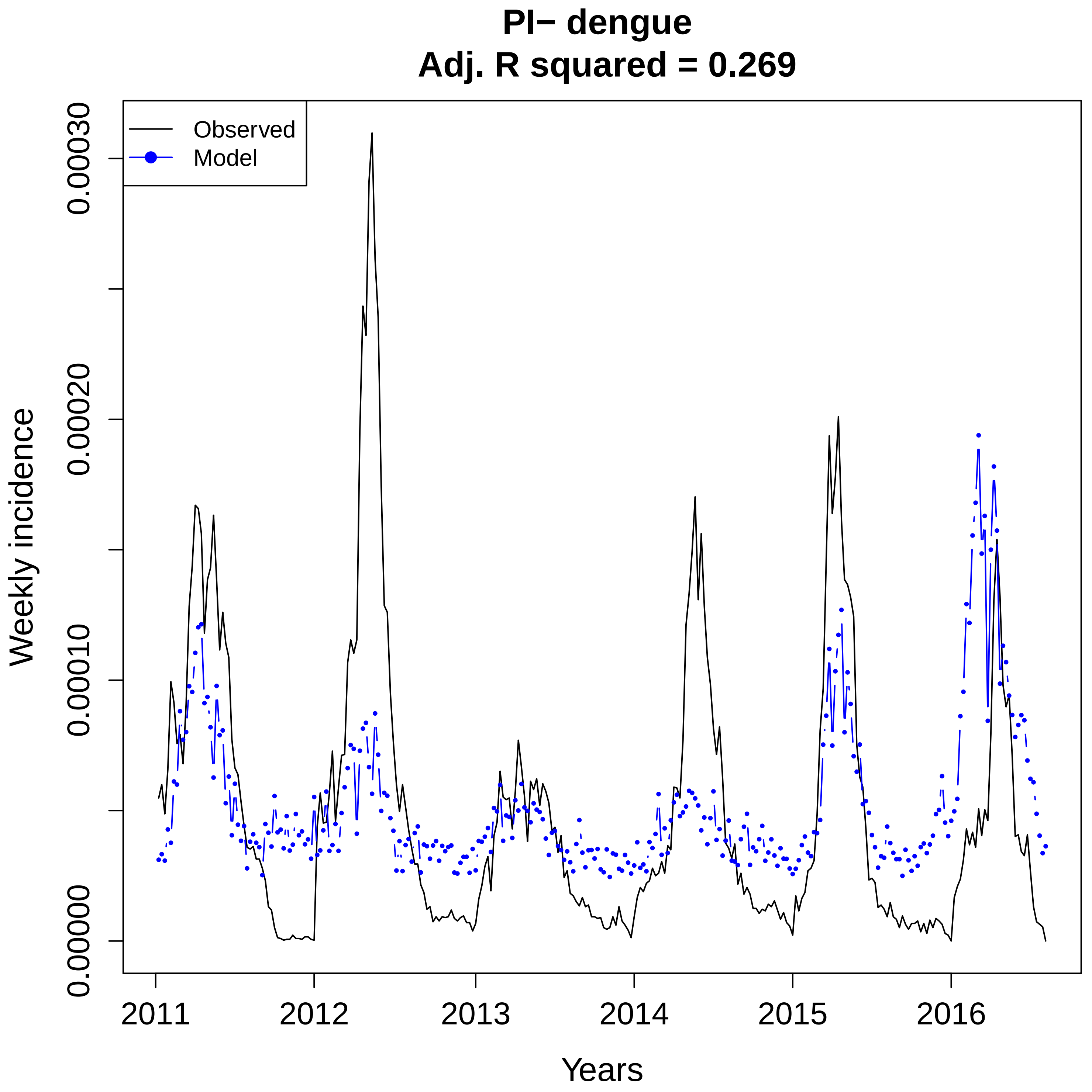

PI− dengue
Adj. R squared = 0.269
Observed
Model
Weekly incidence
0.00000
0.00010
0.00020
0.00030
2011
2012
2013
2014
2015
2016
Years

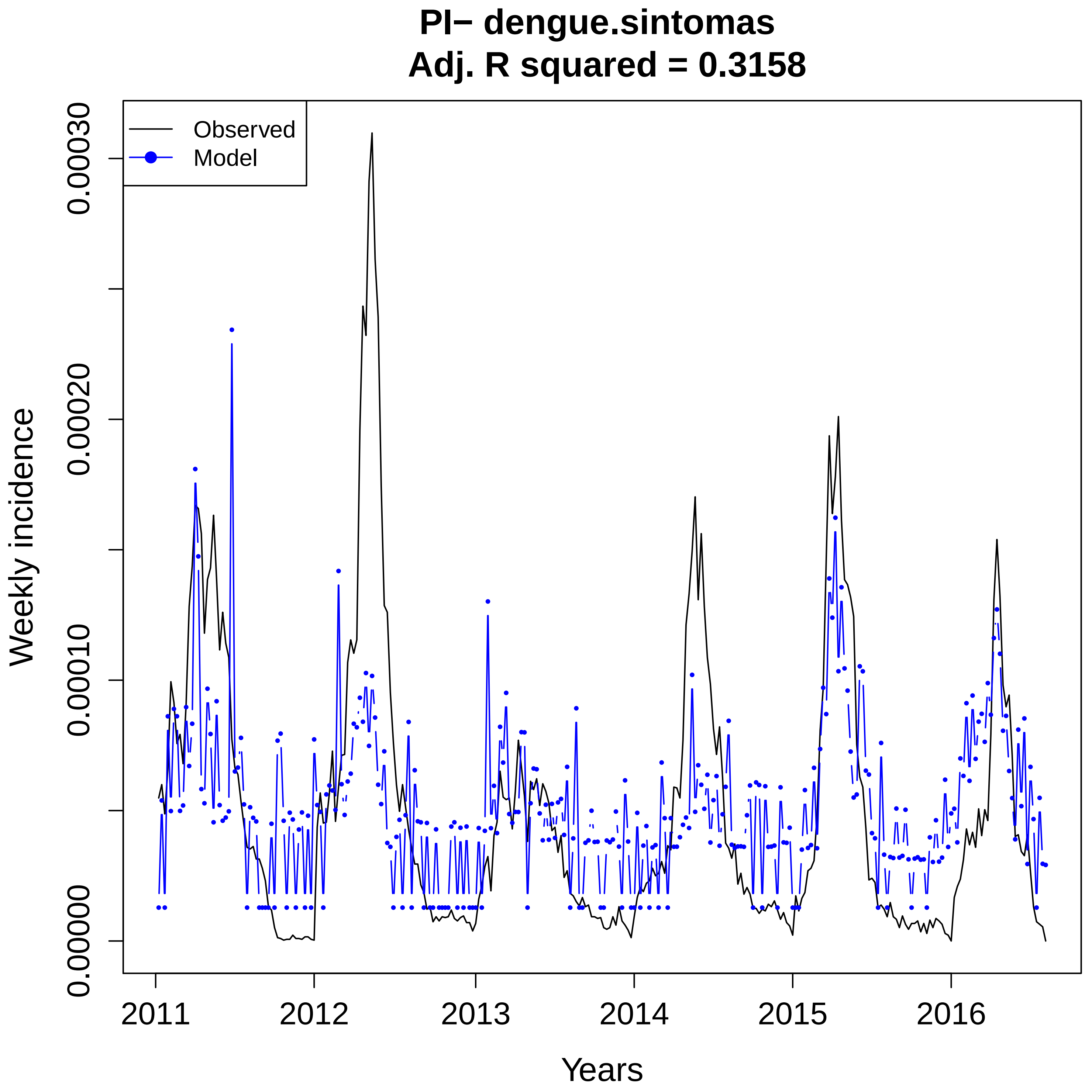

PI− dengue.sintomas
Adj. R squared = 0.3158
Observed
Model
Weekly incidence
0.00000
0.00010
0.00020
0.00030
2011
2012
2013
2014
2015
2016
Years

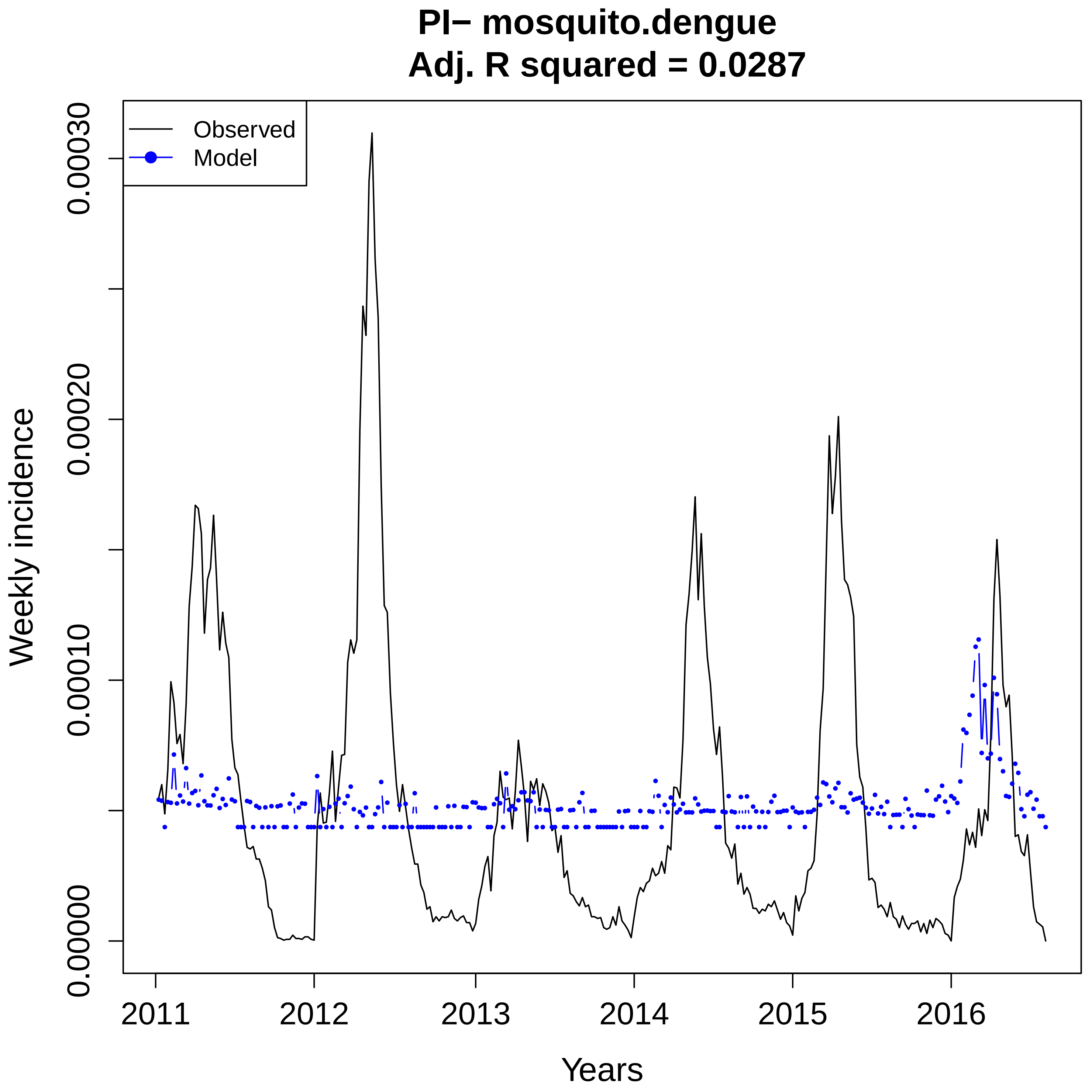
PI− mosquito.dengue
Adj. R squared = 0.0287
Observed
Model
Weekly incidence
0.00000
0.00010
0.00020
0.00030
2011
2012
2013
2014
2015
2016
Years

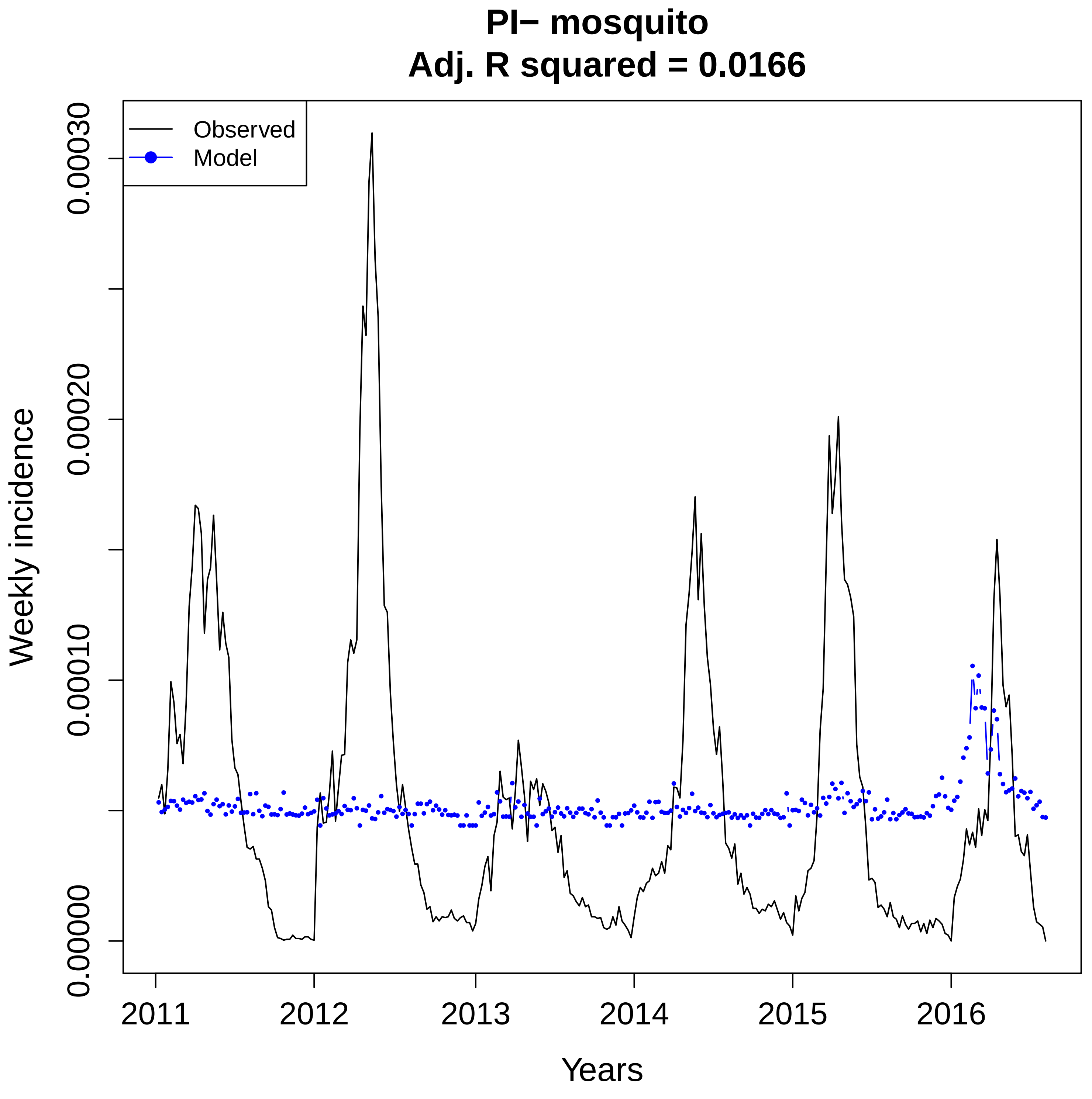
PI− mosquito
Adj. R squared = 0.0166
Observed
Model
Weekly incidence
0.00000
0.00010
0.00020
0.00030
2011
2012
2013
2014
2015
2016
Years

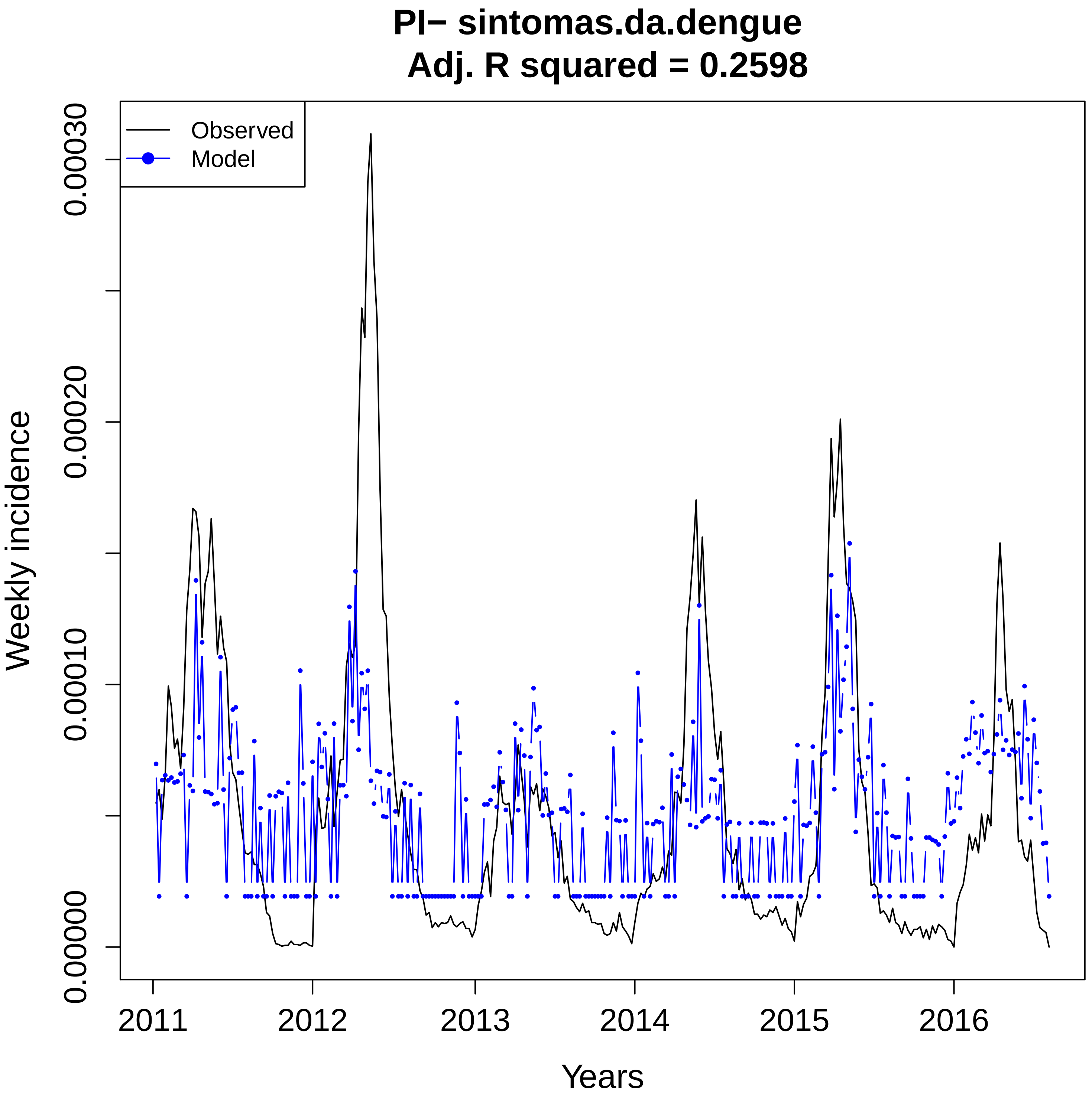

PI− sintomas.da.dengue
Adj. R squared = 0.2598
Observed
Model
Weekly incidence
0.00000
0.00010
0.00020
0.00030
2011
2012
2013
2014
2015
2016
Years

Paraná (PR)

Dengue incidence vs. Google Health Trends (2011–2016)

Definitions:

- **Combined terms:**
  - **All:** model with all the available terms. n= number of terms
  - **Four:** model with "dengue", "dengue sintomas", "aedes", and "mosquito".
  - **Uncorrelated:** model with non-correlated terms. n = number of uncorrelated terms
- **Individual terms:** Plots developed with one term at a time.

- Terms for all and uncorrelated models can be found in additional file 3.

Combined terms
Adjusted R squared

Paraná (PR)

All
(n = 12)

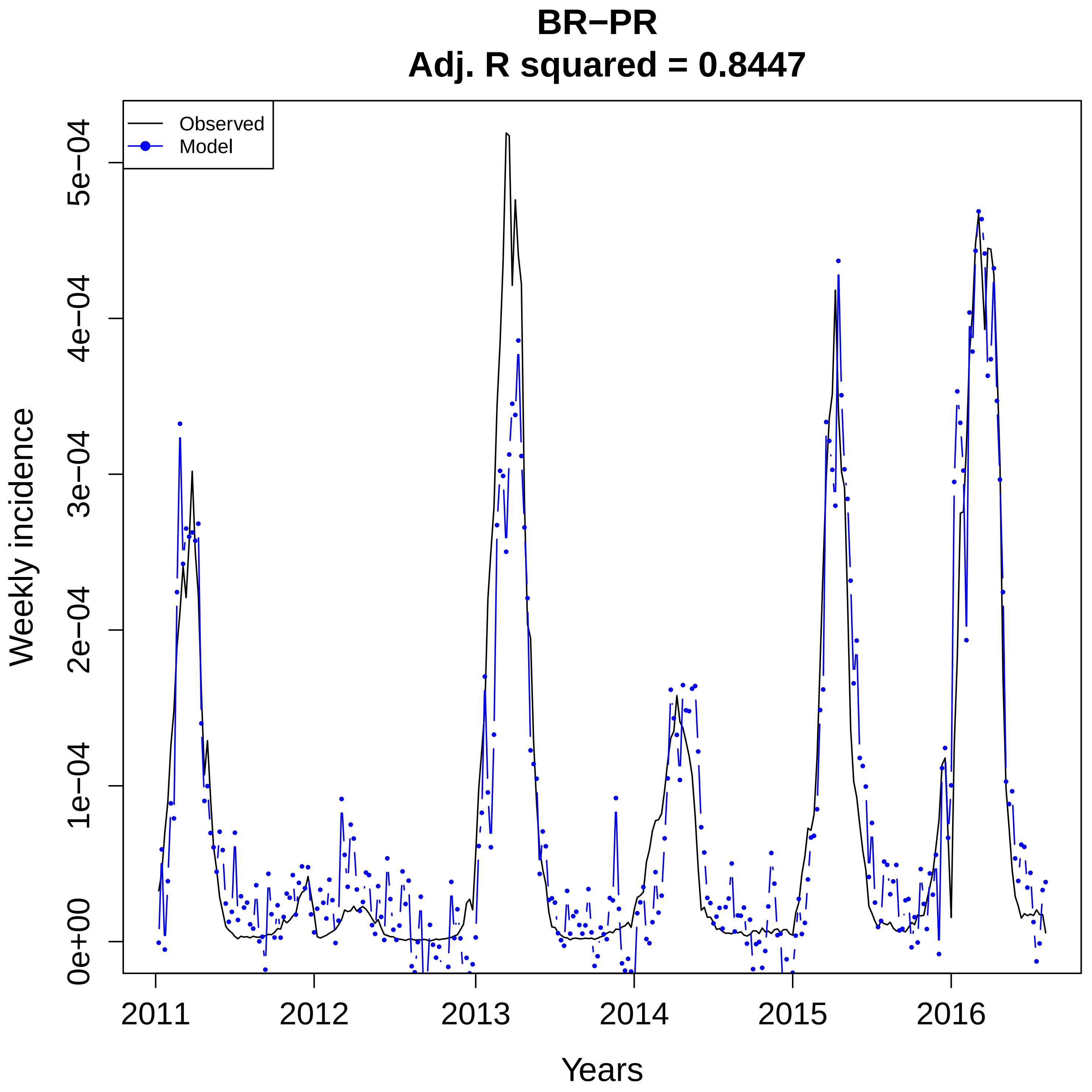

BR−PR
Adj. R squared = 0.8447
Observed
Model
Weekly incidence
0e+00
1e−04
2e−04
3e−04
4e−04
5e−04
2011
2012
2013
2014
2015
2016
Years

Four

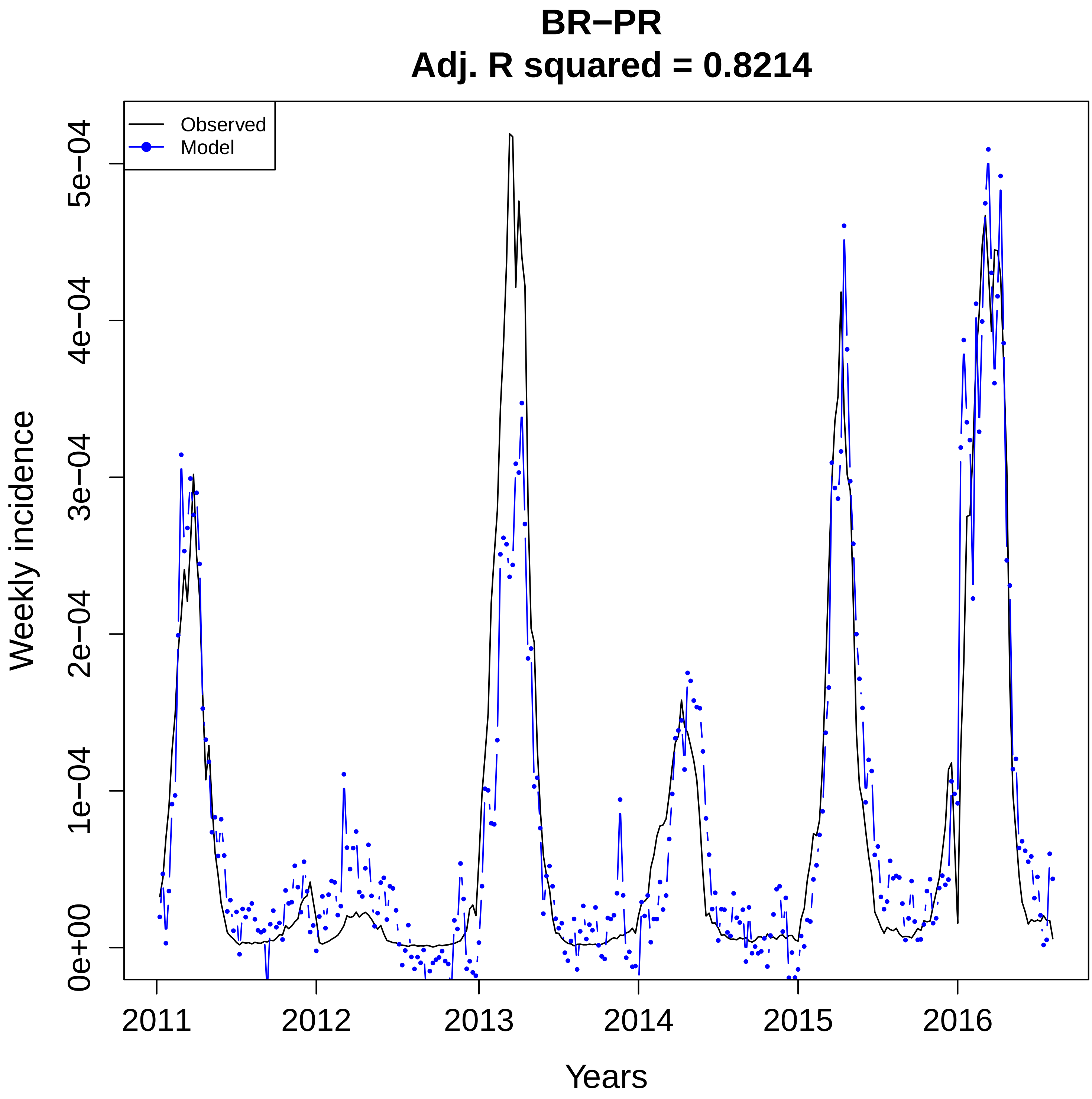

BR−PR
Adj. R squared = 0.8214
Observed
Model
Weekly incidence
0e+00
1e−04
2e−04
3e−04
4e−04
5e−04
2011
2012
2013
2014
2015
2016
Years

Uncorrelated
(n = 4)

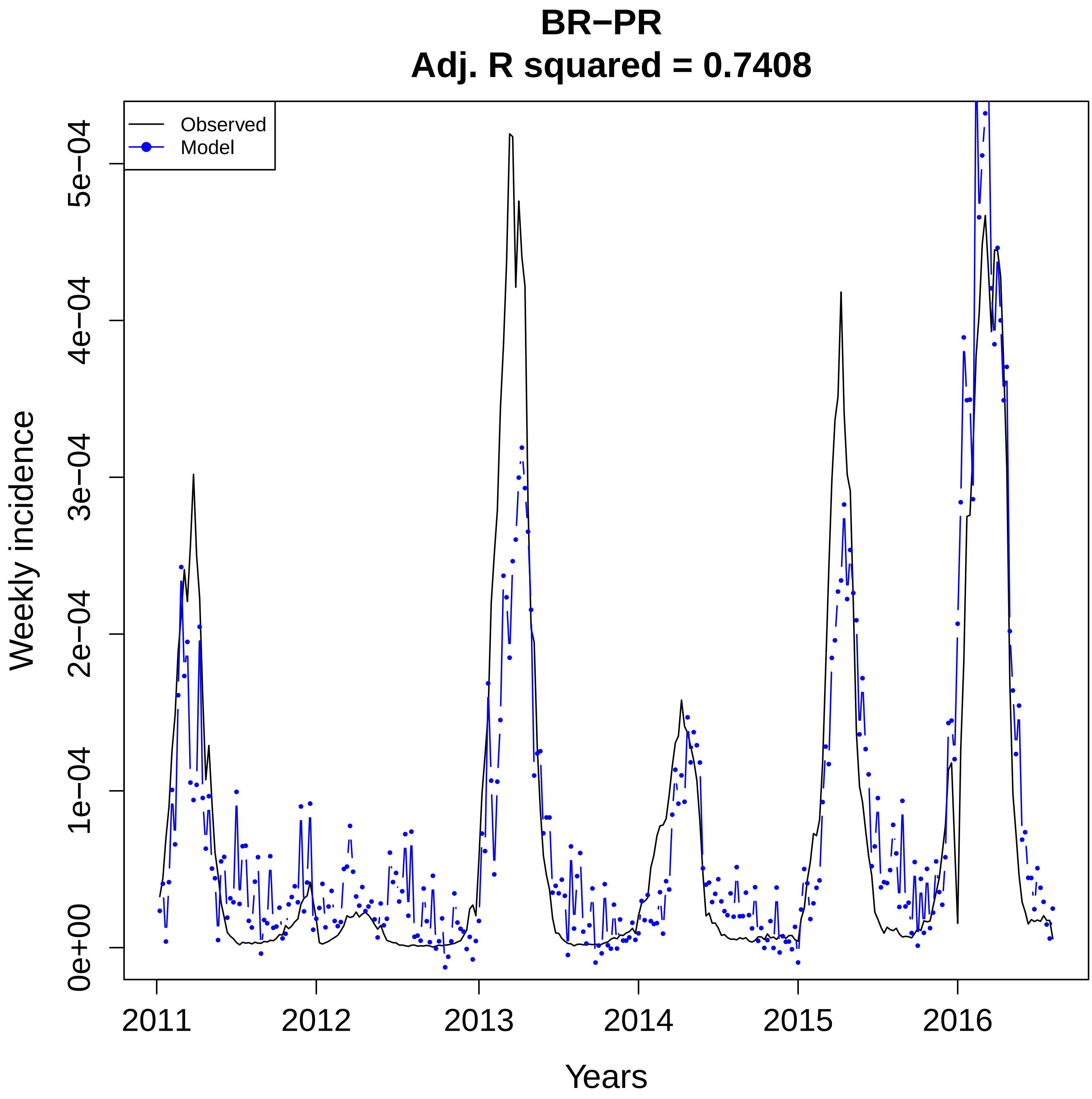

Individual terms
Adjusted R squared

Paraná (PR)

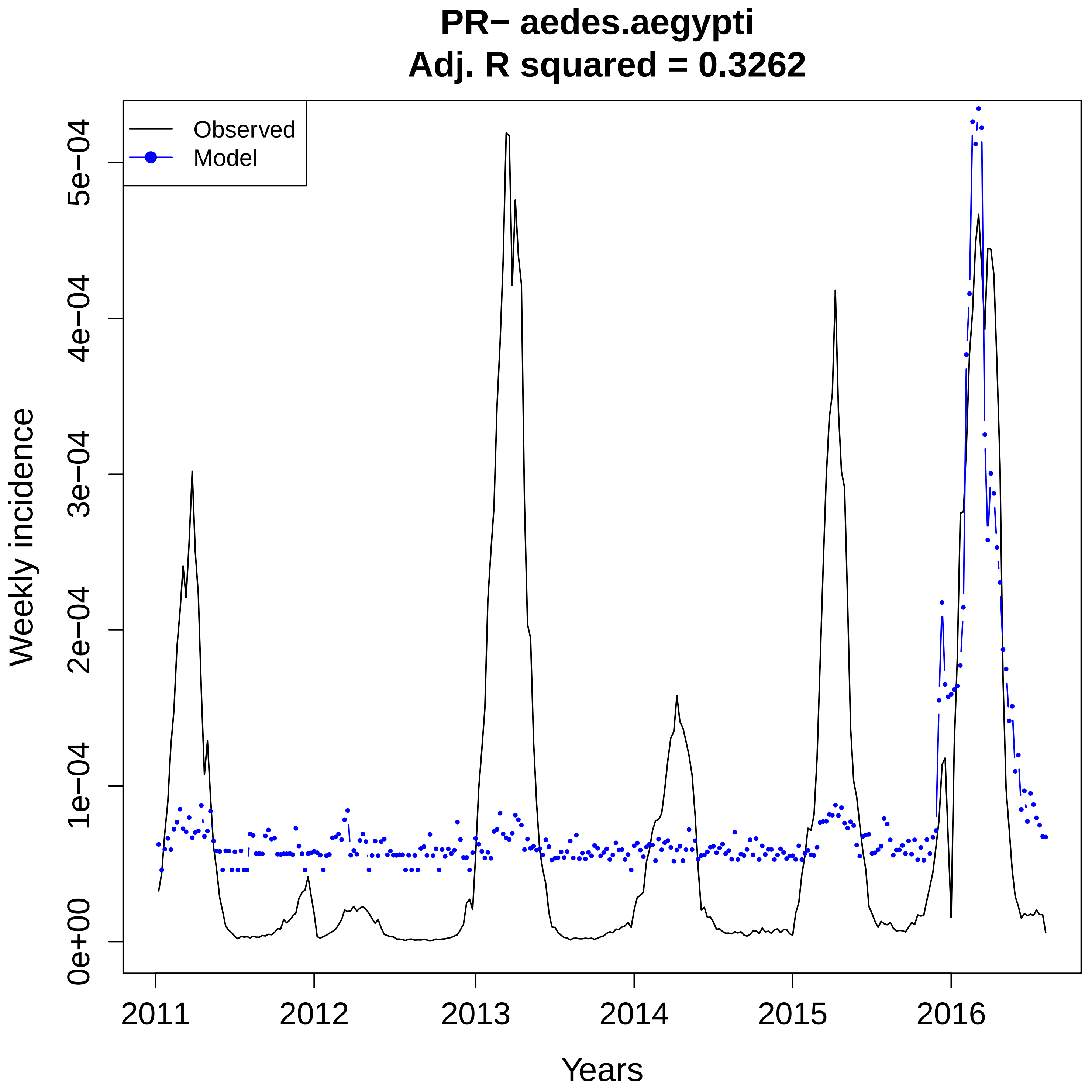

PR− aedes.aegypti
Adj. R squared = 0.3262
Observed
Model
Weekly incidence
0e+00
1e−04
2e−04
3e−04
4e−04
5e−04
2011
2012
2013
2014
2015
2016
Years

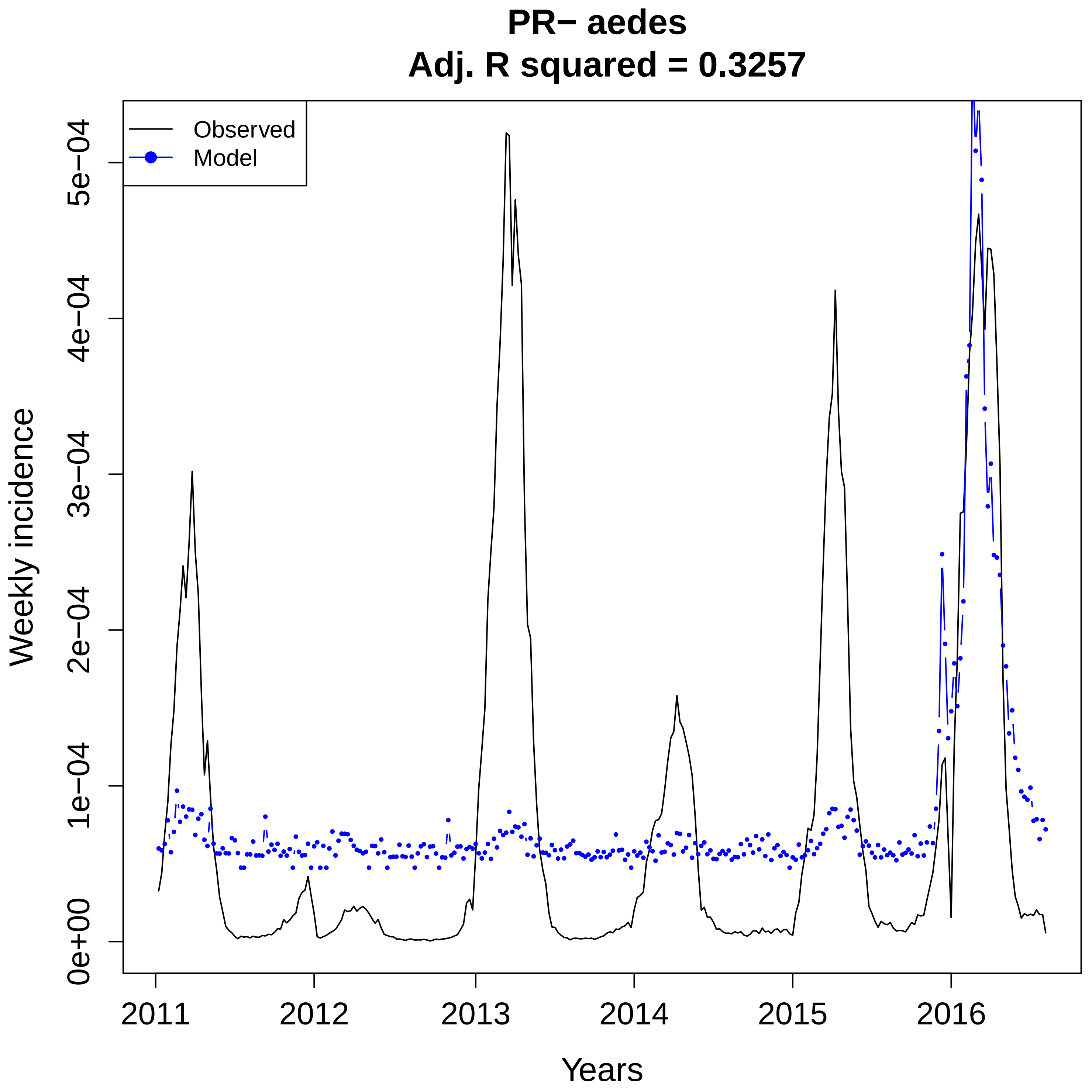
PR− aedes
Adj. R squared = 0.3257
Observed
Model
Weekly incidence
0e+00
1e−04
2e−04
3e−04
4e−04
5e−04
2011
2012
2013
2014
2015
2016
Years

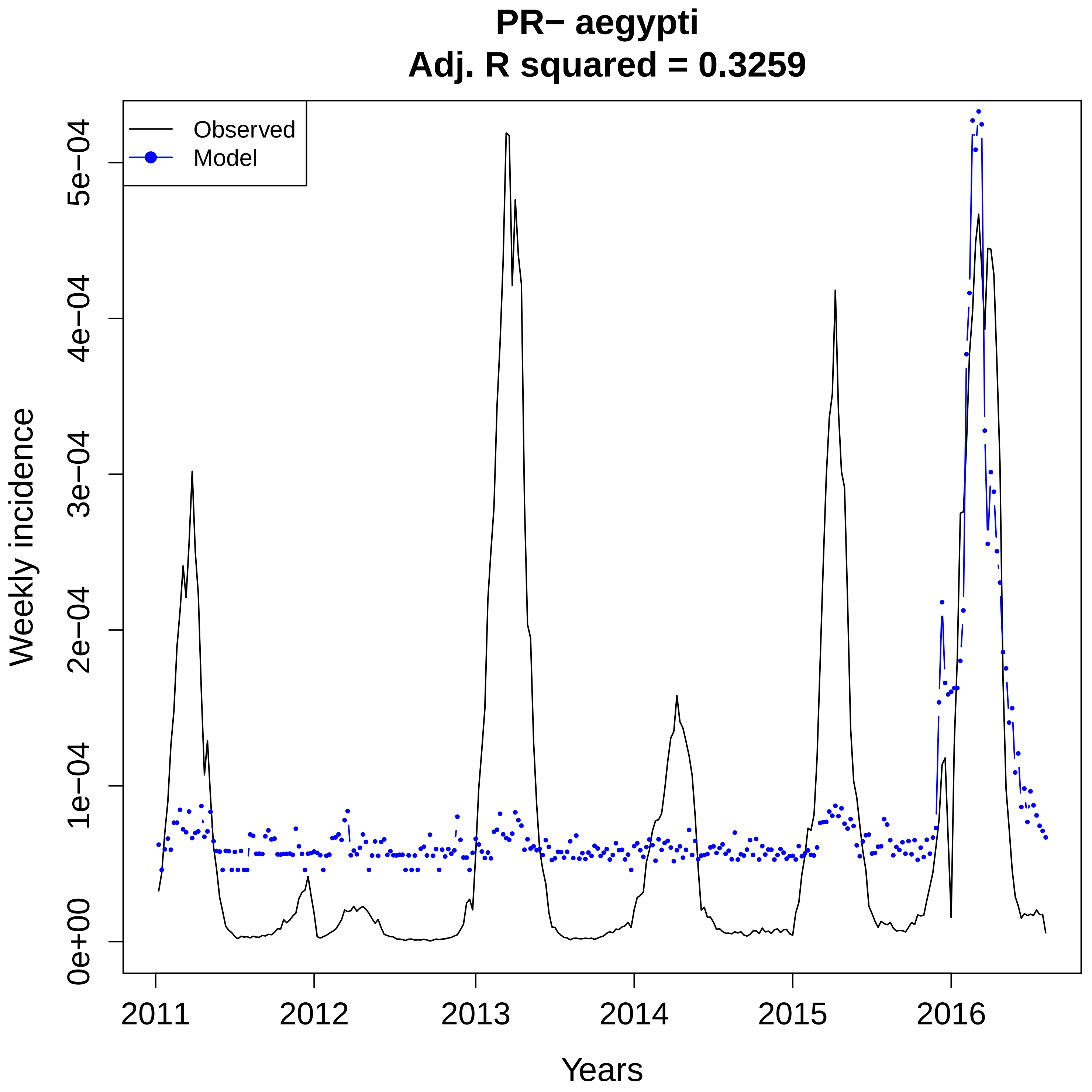

PR− aegypti
Adj. R squared = 0.3259
Observed
Model
Weekly incidence
0e+00
1e−04
2e−04
3e−04
4e−04
5e−04
2011
2012
2013
2014
2015
2016
Years

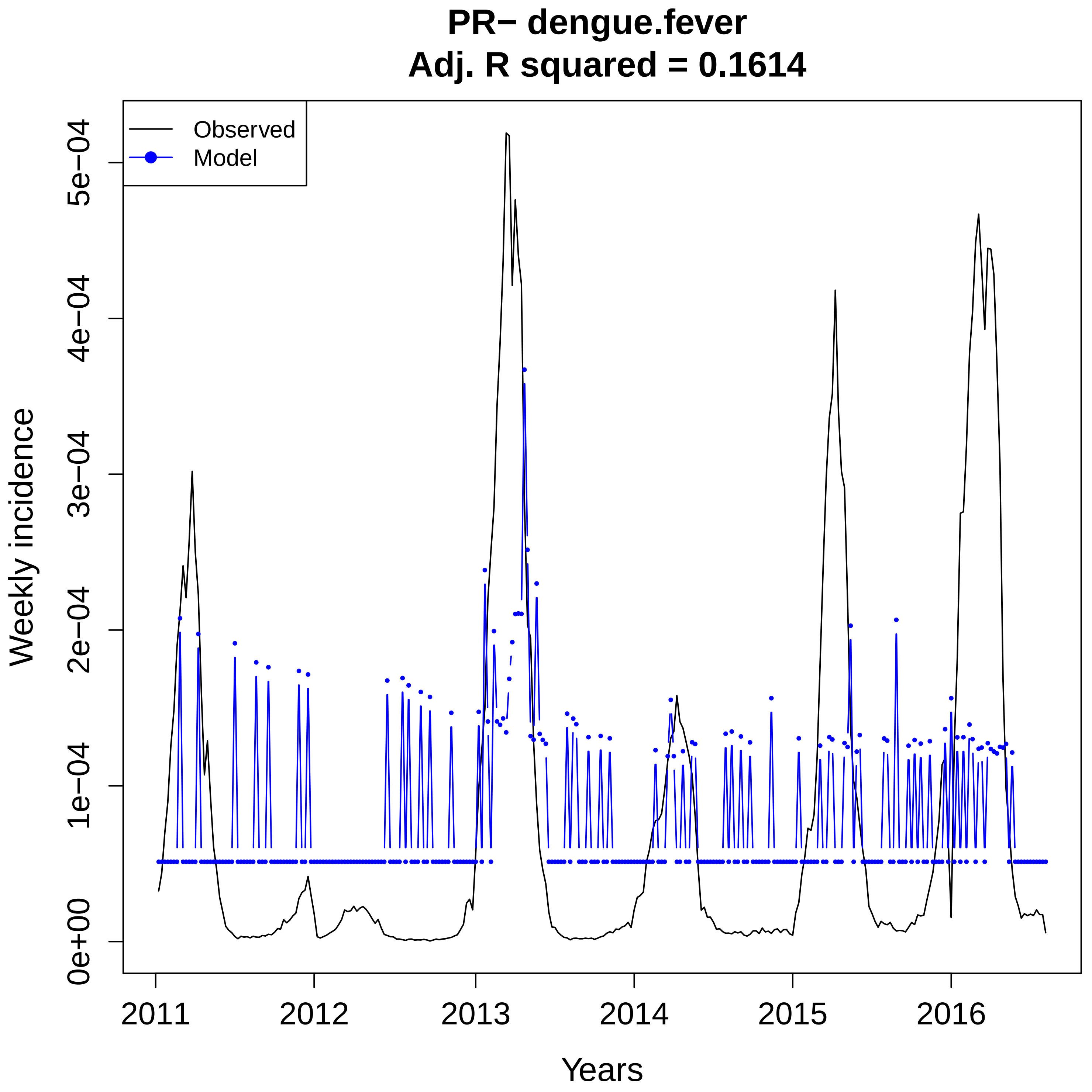

PR− dengue.fever
Adj. R squared = 0.1614
Observed
Model
Weekly incidence
0e+00
1e−04
2e−04
3e−04
4e−04
5e−04
2011
2012
2013
2014
2015
2016
Years

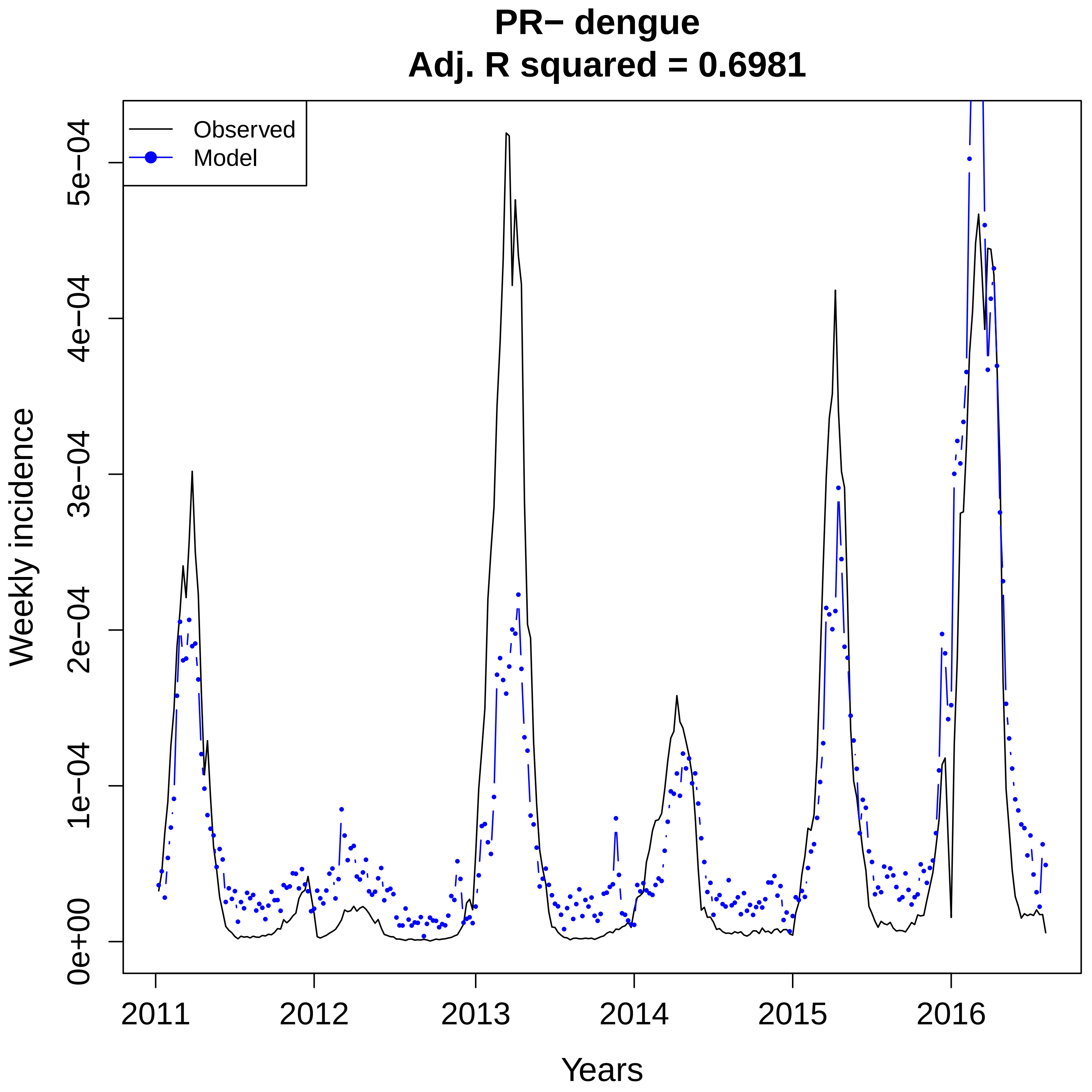

PR− dengue
Adj. R squared = 0.6981
Observed
Model
Weekly incidence
0e+00
1e−04
2e−04
3e−04
4e−04
5e−04
2011
2012
2013
2014
2015
2016
Years

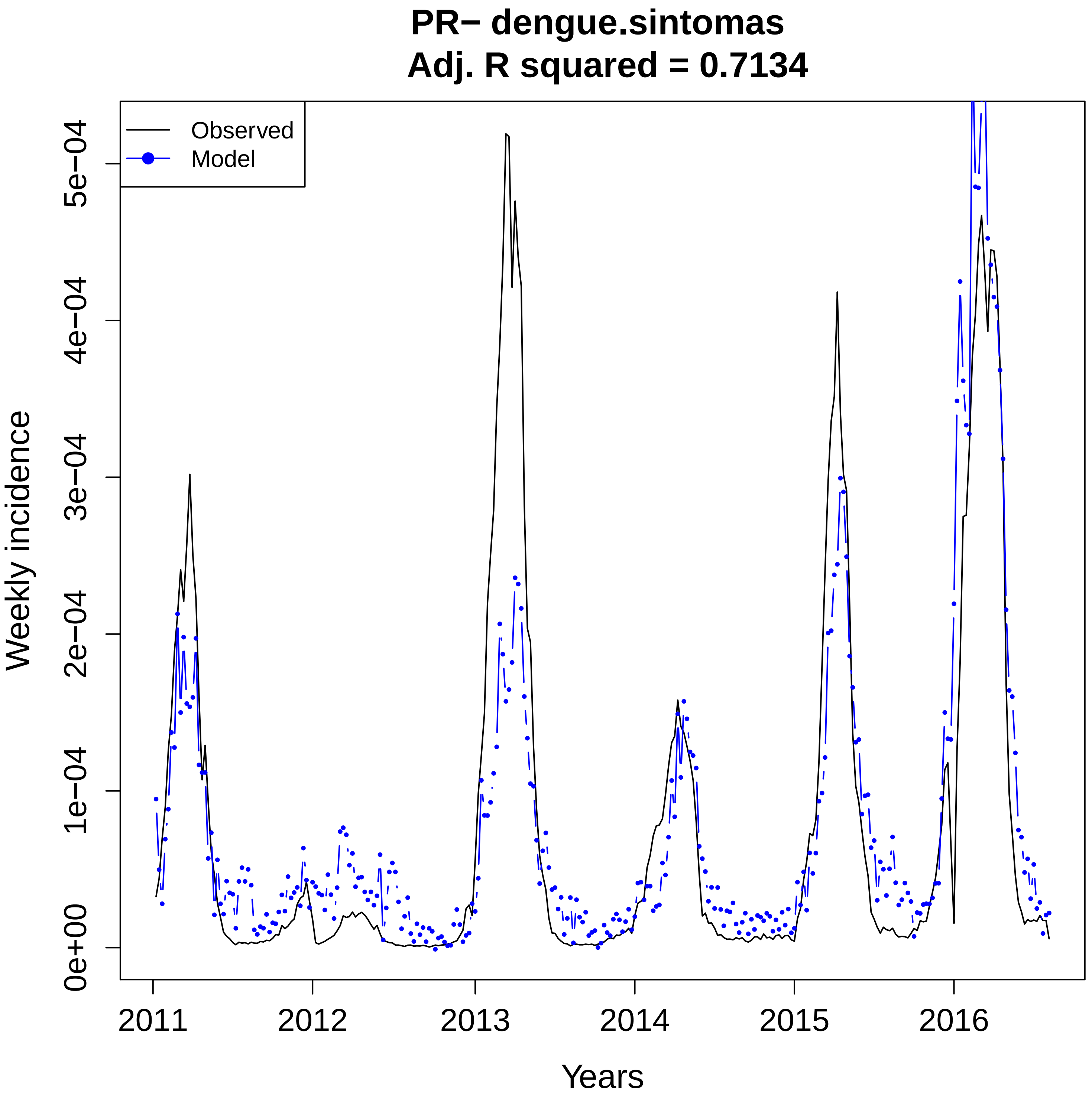
PR− dengue.sintomas
Adj. R squared = 0.7134
Observed
Model
Weekly incidence
0e+00
1e−04
2e−04
3e−04
4e−04
5e−04
2011
2012
2013
2014
2015
2016
Years

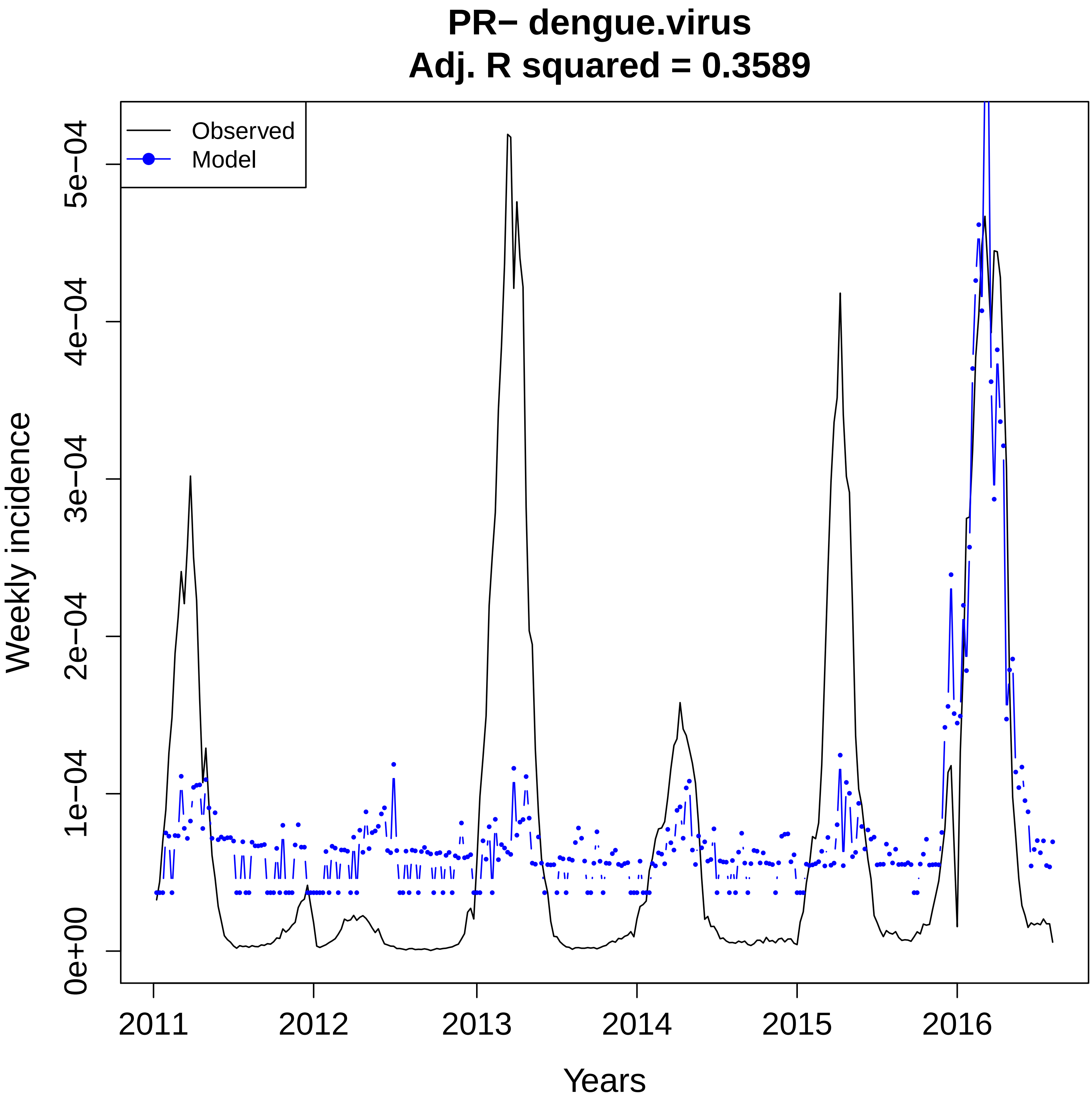

PR− dengue.virus
Adj. R squared = 0.3589
Observed
Model
Weekly incidence
0e+00
1e−04
2e−04
3e−04
4e−04
5e−04
2011
2012
2013
2014
2015
2016
Years

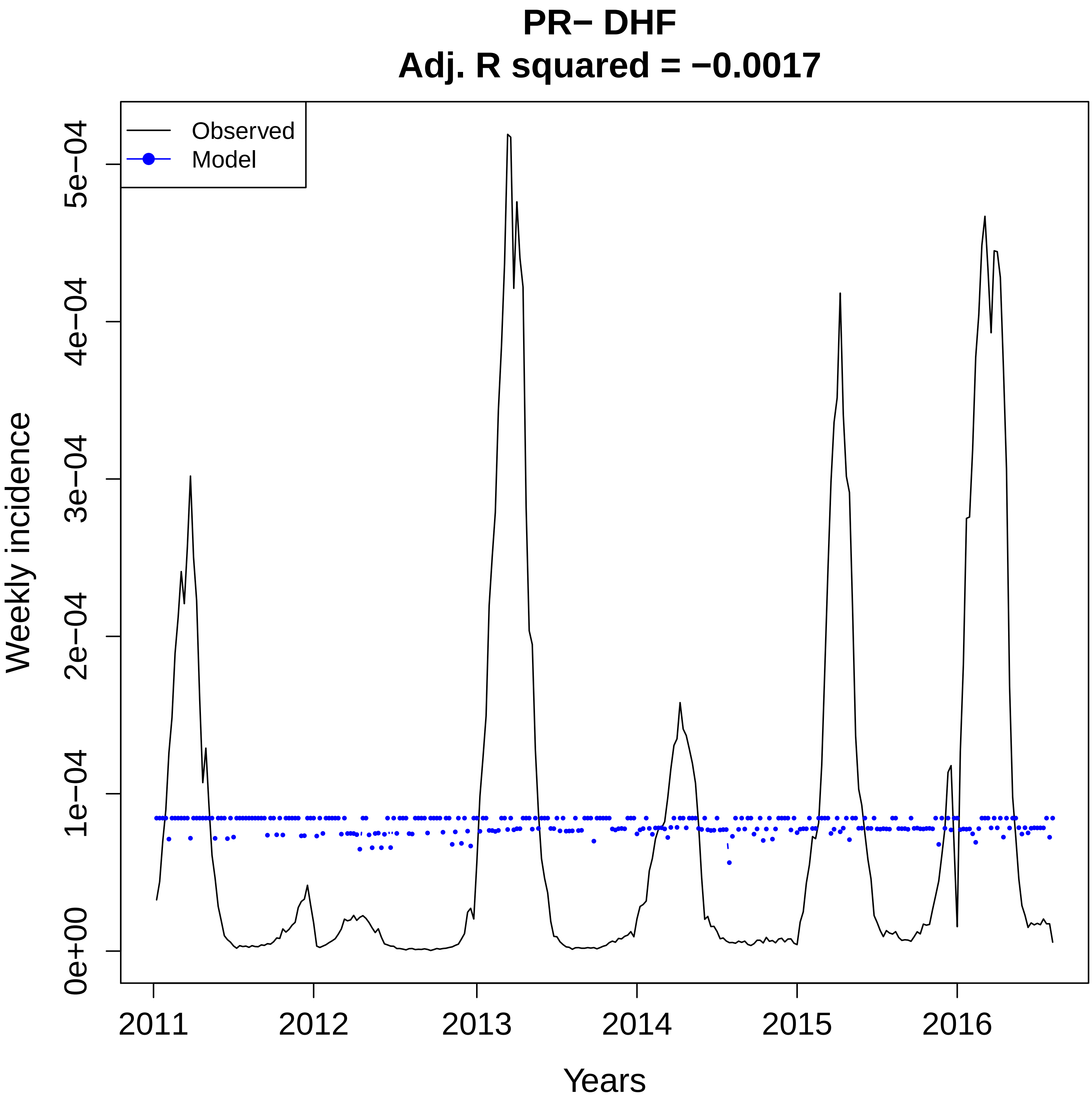
PR− DHF
Adj. R squared = −0.0017
Observed
Model
Weekly incidence
0e+00
1e−04
2e−04
3e−04
4e−04
5e−04
2011
2012
2013
2014
2015
2016
Years

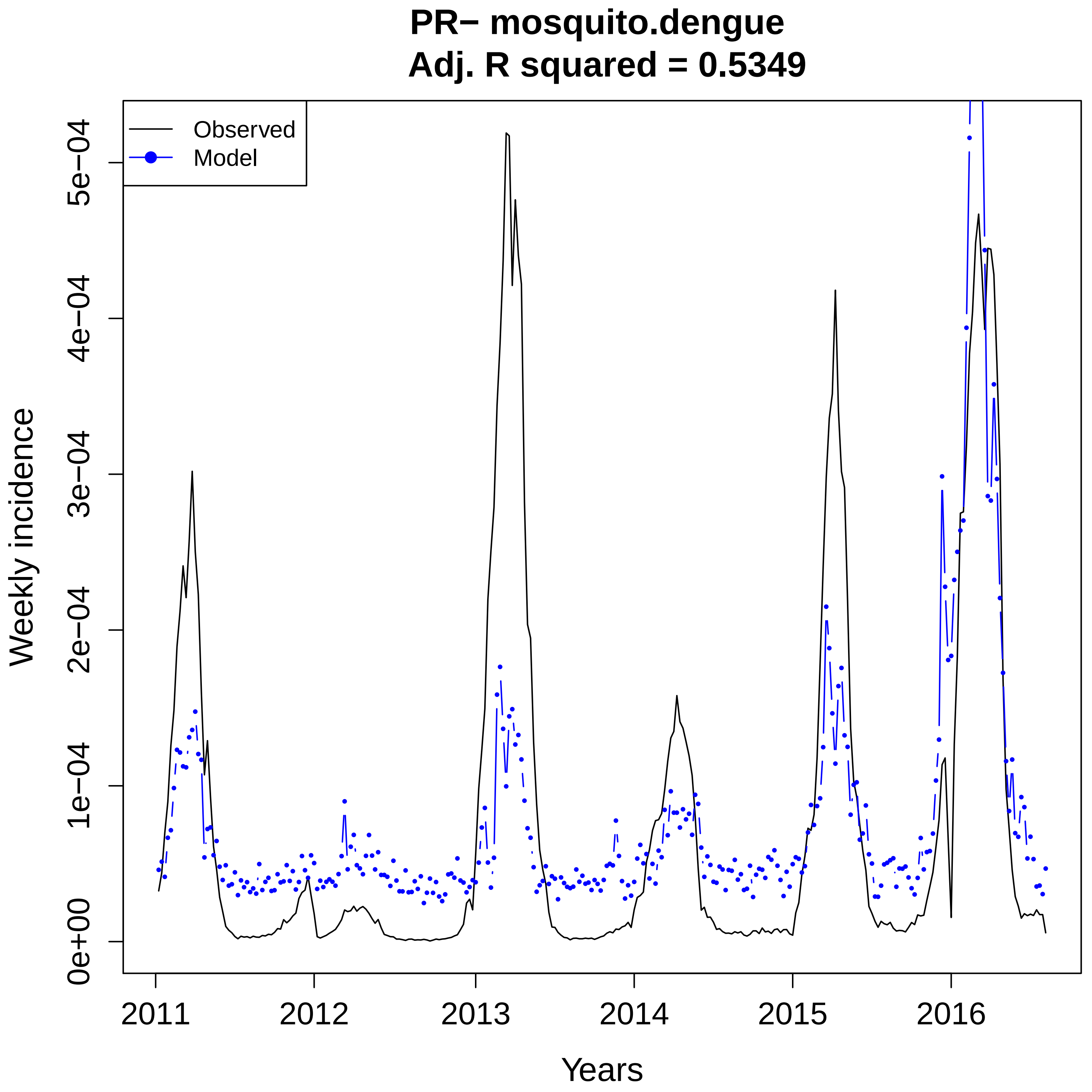

PR− mosquito.dengue
Adj. R squared = 0.5349
Observed
Model
Weekly incidence
0e+00
1e−04
2e−04
3e−04
4e−04
5e−04
2011
2012
2013
2014
2015
2016
Years

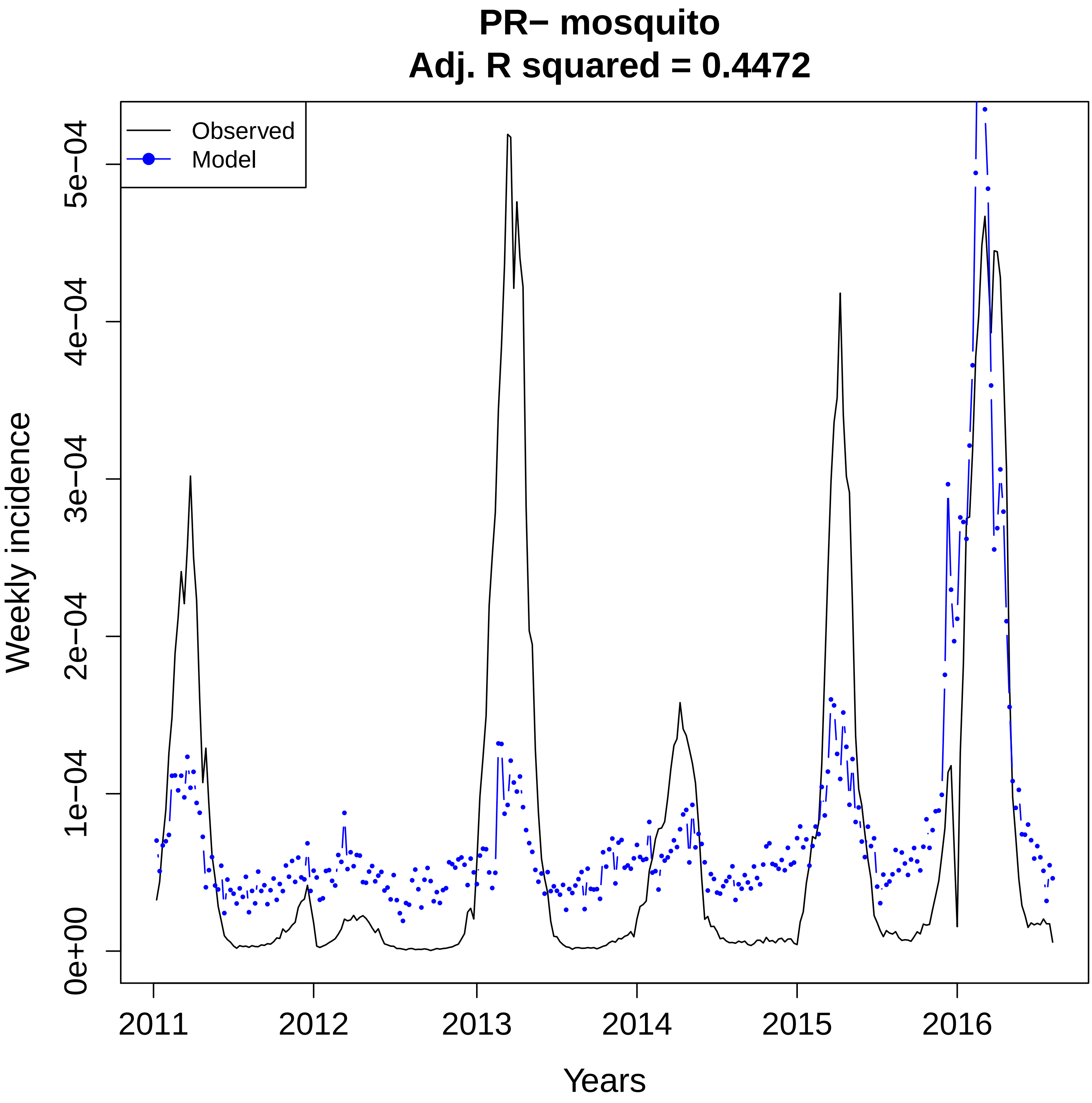

PR− mosquito
Adj. R squared = 0.4472
Observed
Model
Weekly incidence
0e+00
1e−04
2e−04
3e−04
4e−04
5e−04
2011
2012
2013
2014
2015
2016
Years

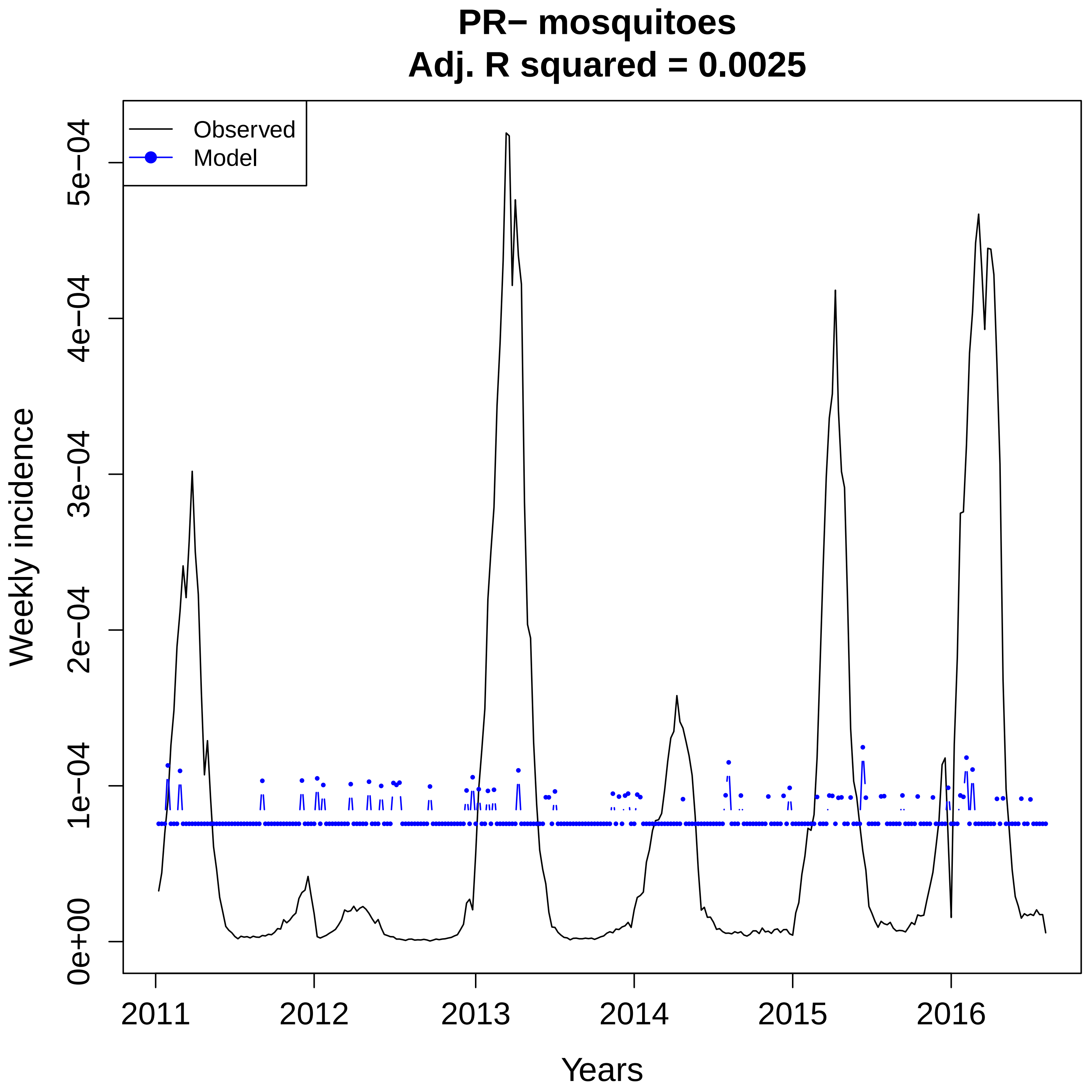
PR− mosquitoes
Adj. R squared = 0.0025
Observed
Model
Weekly incidence
0e+00
1e−04
2e−04
3e−04
4e−04
5e−04
2011
2012
2013
2014
2015
2016
Years

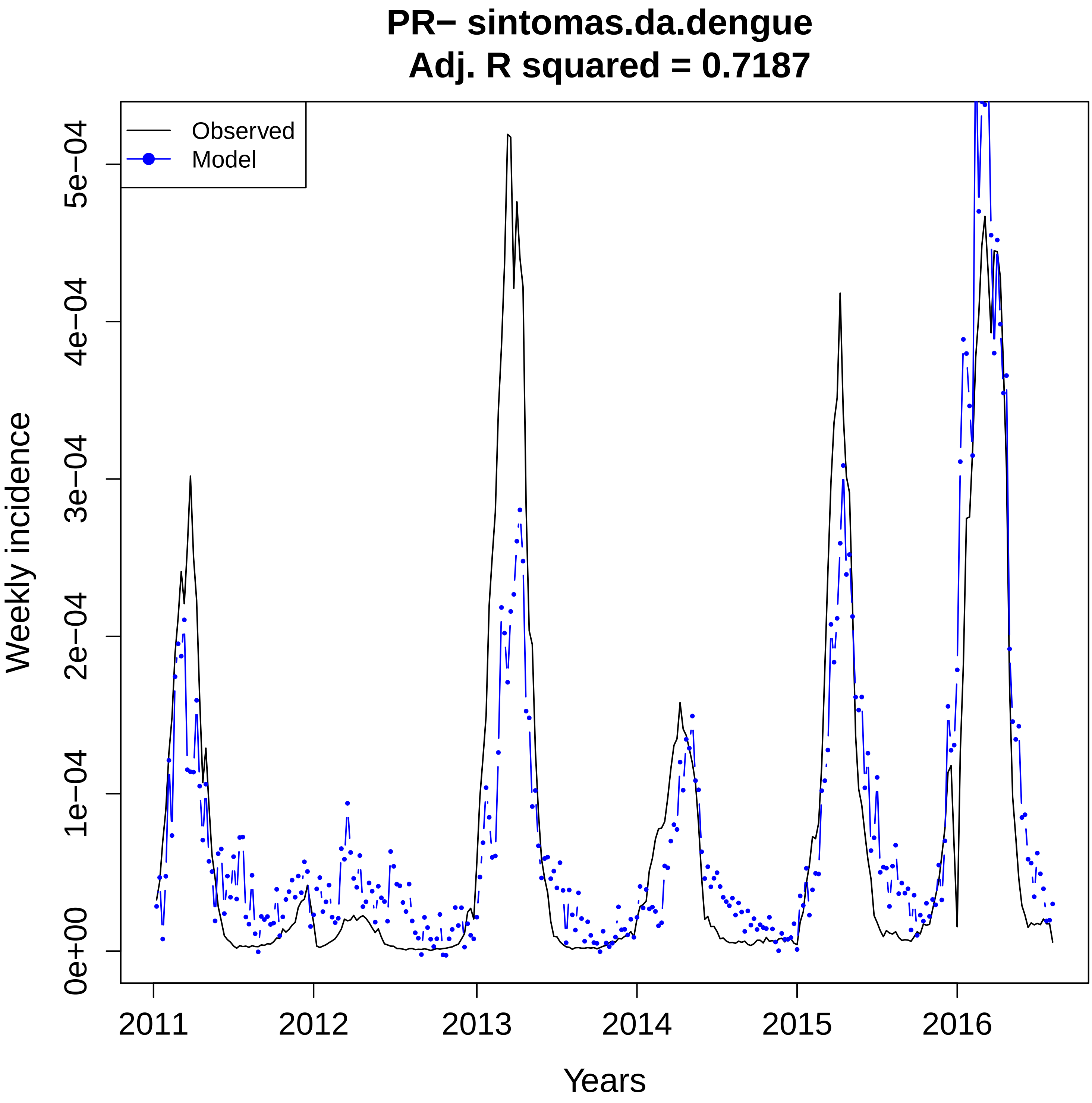
PR− sintomas.da.dengue
Adj. R squared = 0.7187
Observed
Model
Weekly incidence
0e+00
1e−04
2e−04
3e−04
4e−04
5e−04
2011
2012
2013
2014
2015
2016
Years

Rio de Janeiro (RJ)
Dengue incidence vs. Google Health Trends (2011–2016)

Definitions:

- **Combined terms:**
  - **All:** model with all the available terms. n= number of terms
  - **Four:** model with "dengue", "dengue sintomas", "aedes", and "mosquito".
  - **Uncorrelated:** model with non-correlated terms. n = number of uncorrelated terms
- **Individual terms:** Plots developed with one term at a time.

- Terms for all and uncorrelated models can be found in additional file 3.

Combined terms
Adjusted R squared

Rio de Janeiro (RJ)

All
(n = 12)

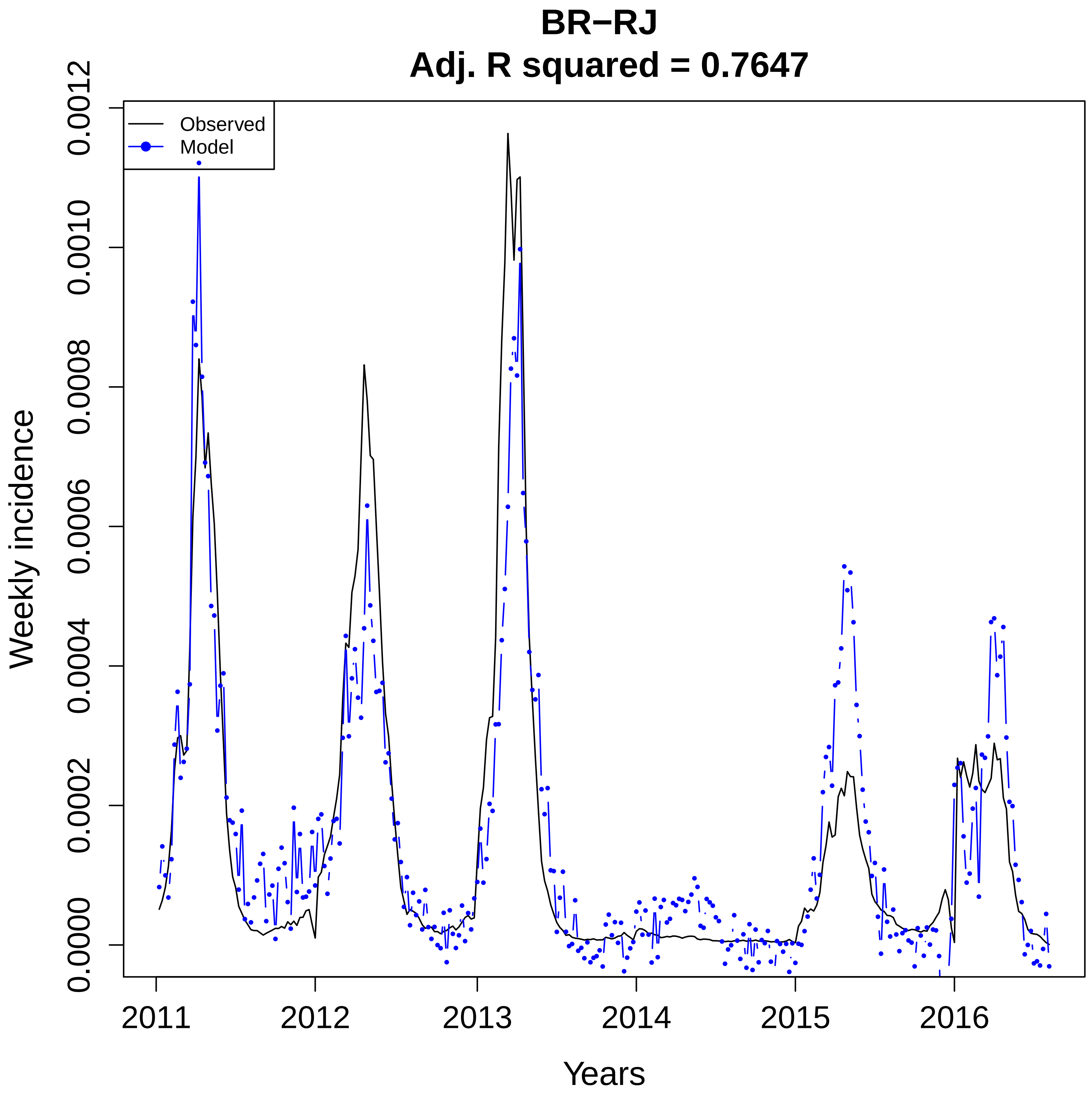

BR−RJ
Adj. R squared = 0.7647
Observed
Model
Weekly incidence
0.0000
0.0002
0.0004
0.0006
0.0008
0.0010
0.0012
2011
2012
2013
2014
2015
2016
Years

Four

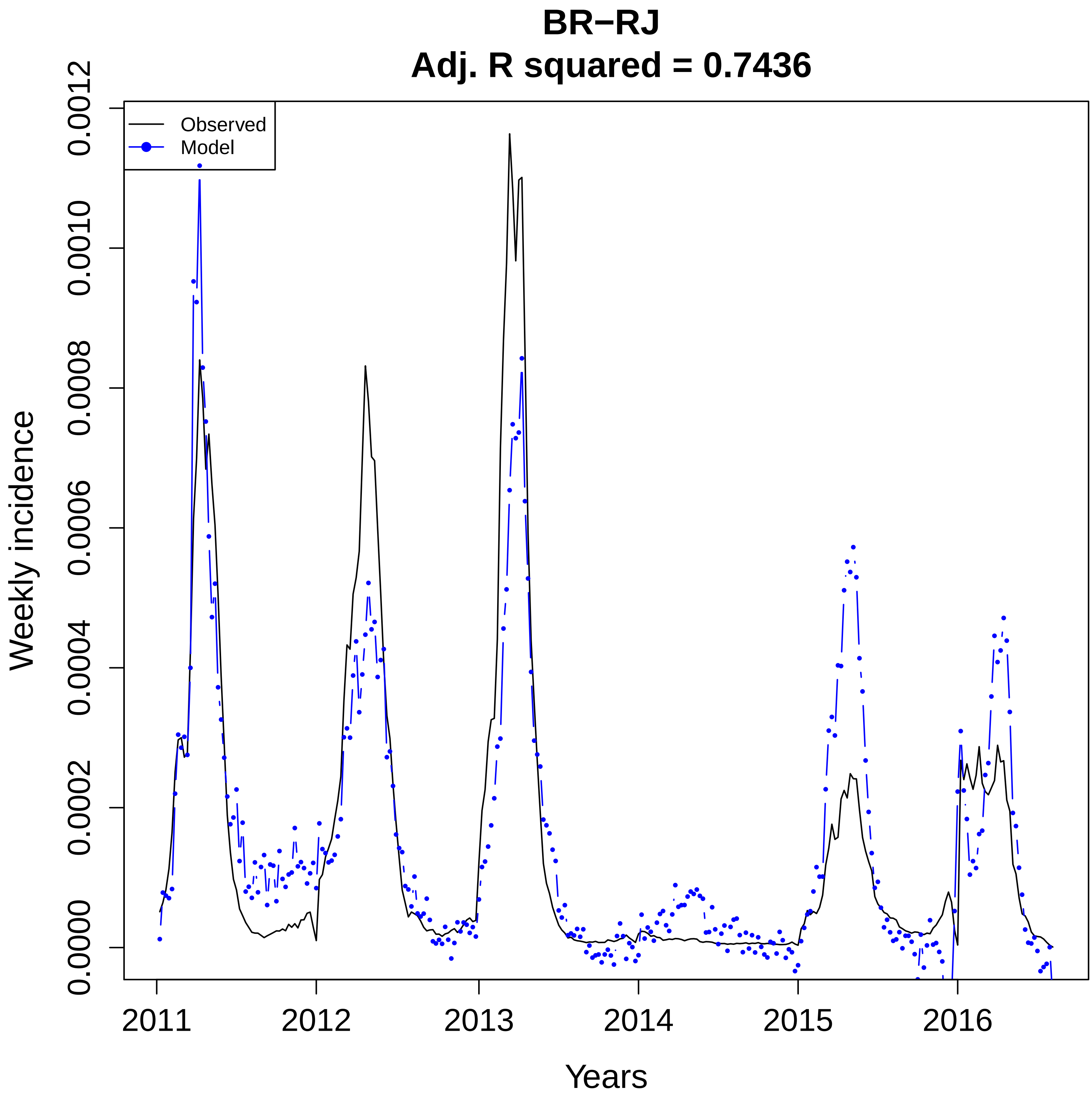

BR−RJ
Adj. R squared = 0.7436
Observed
Model
Weekly incidence
0.0000
0.0002
0.0004
0.0006
0.0008
0.0010
0.0012
2011
2012
2013
2014
2015
2016
Years

Uncorrelated
(n = 5)

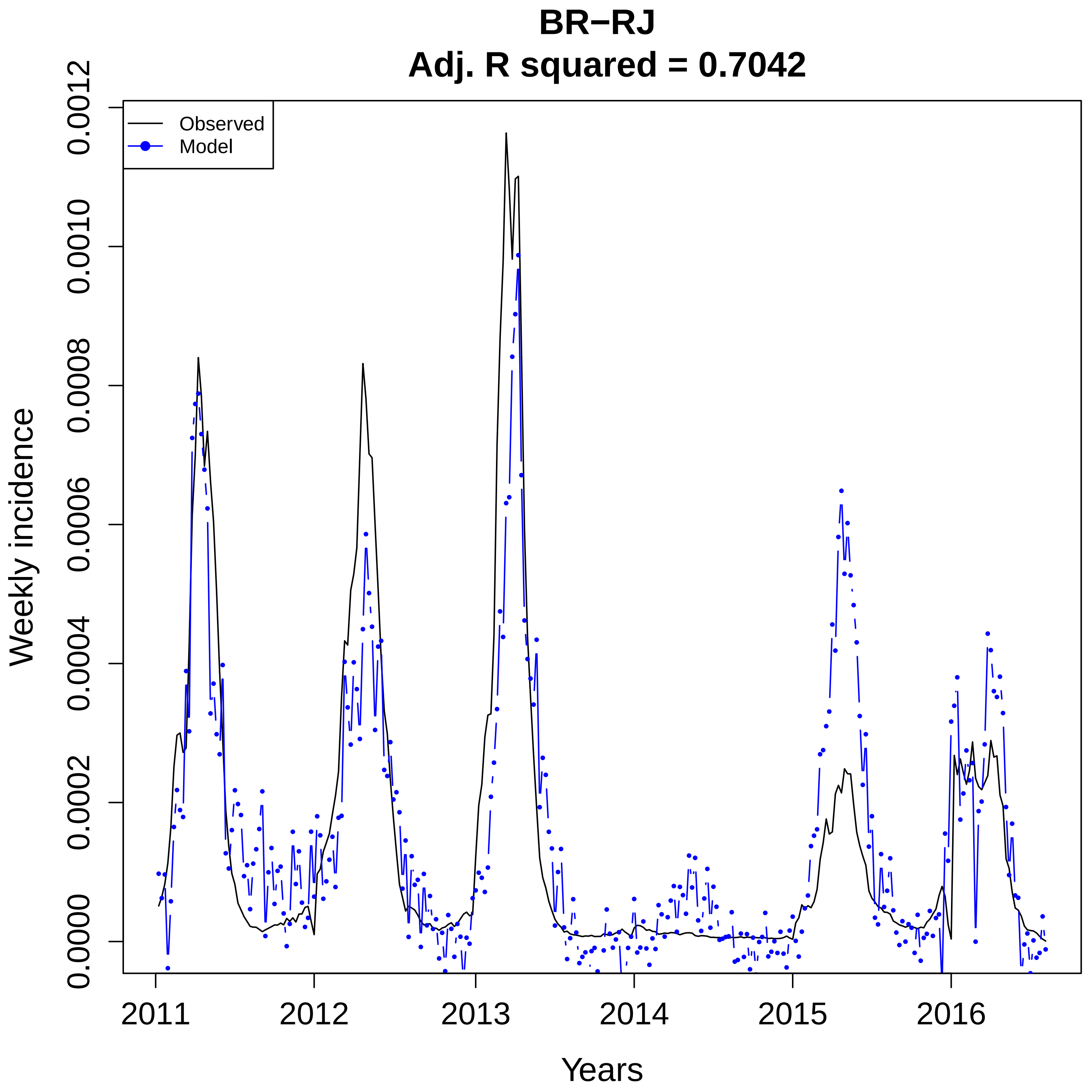

Individual terms
Adjusted R squared

Rio de Janeiro (RJ)

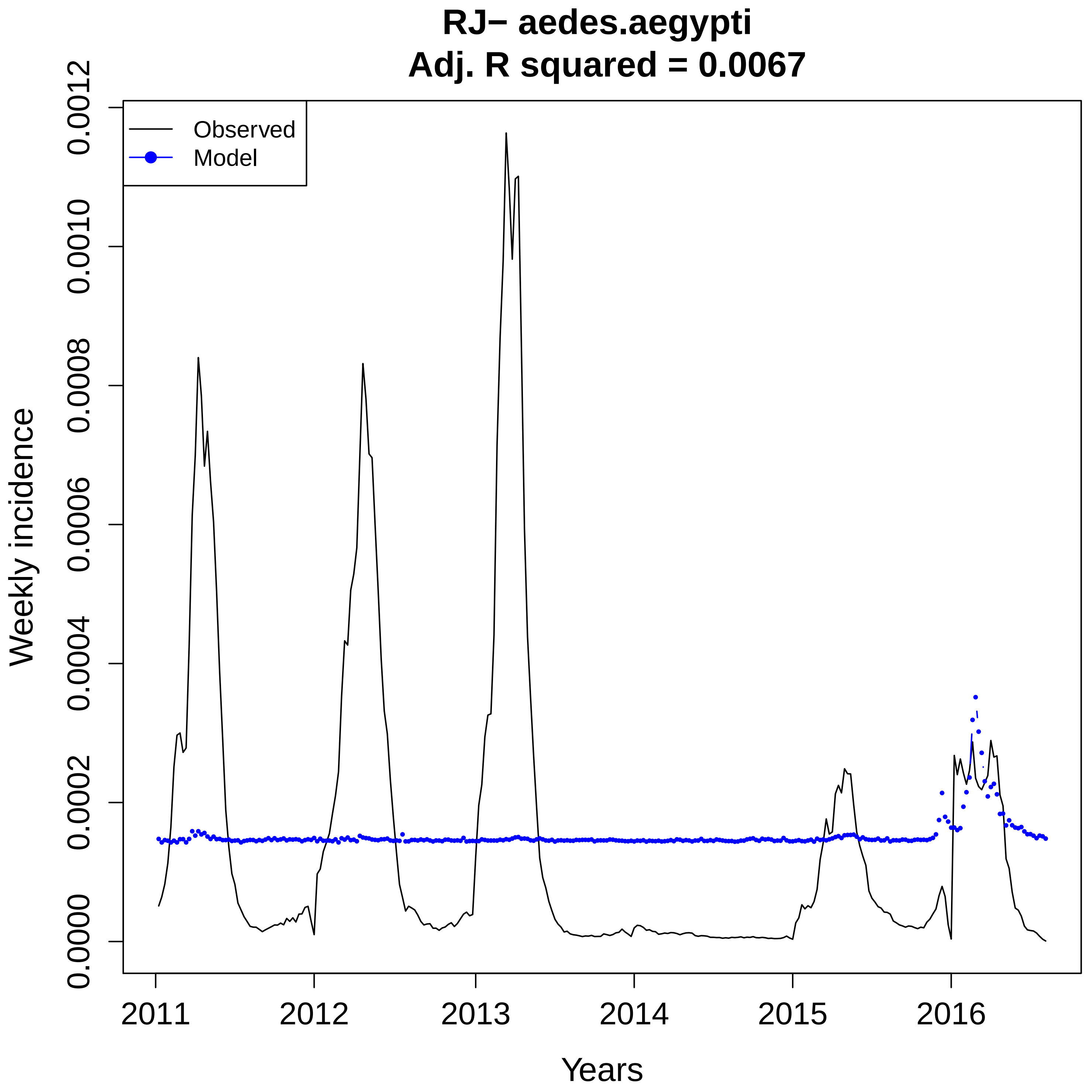

RJ− aedes.aegypti
Adj. R squared = 0.0067
Observed
Model
Weekly incidence
0.0000
0.0002
0.0004
0.0006
0.0008
0.0010
0.0012
2011
2012
2013
2014
2015
2016
Years

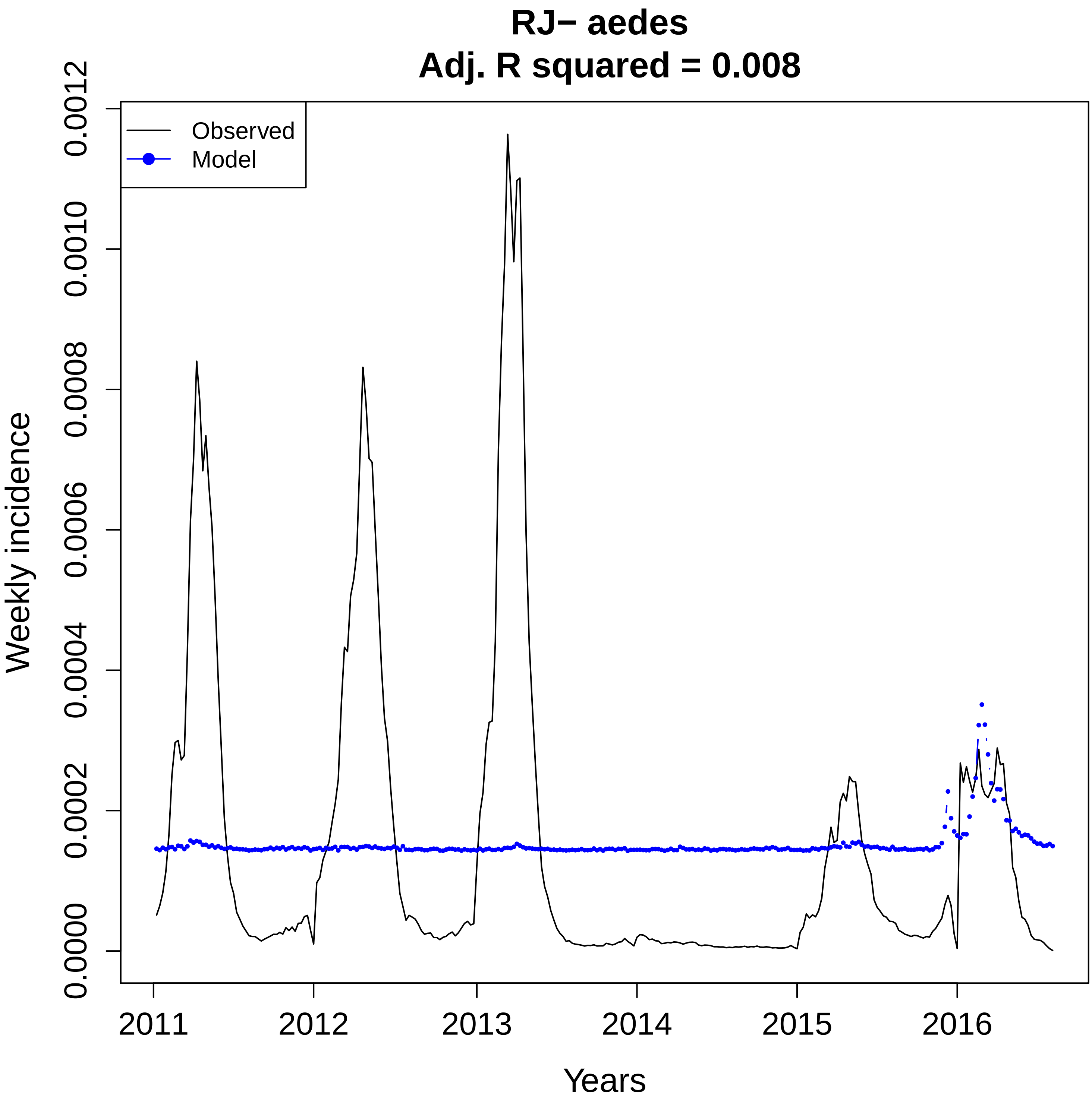
RJ− aedes
Adj. R squared = 0.008
Observed
Model
Weekly incidence
0.0000
0.0002
0.0004
0.0006
0.0008
0.0010
0.0012
2011
2012
2013
2014
2015
2016
Years

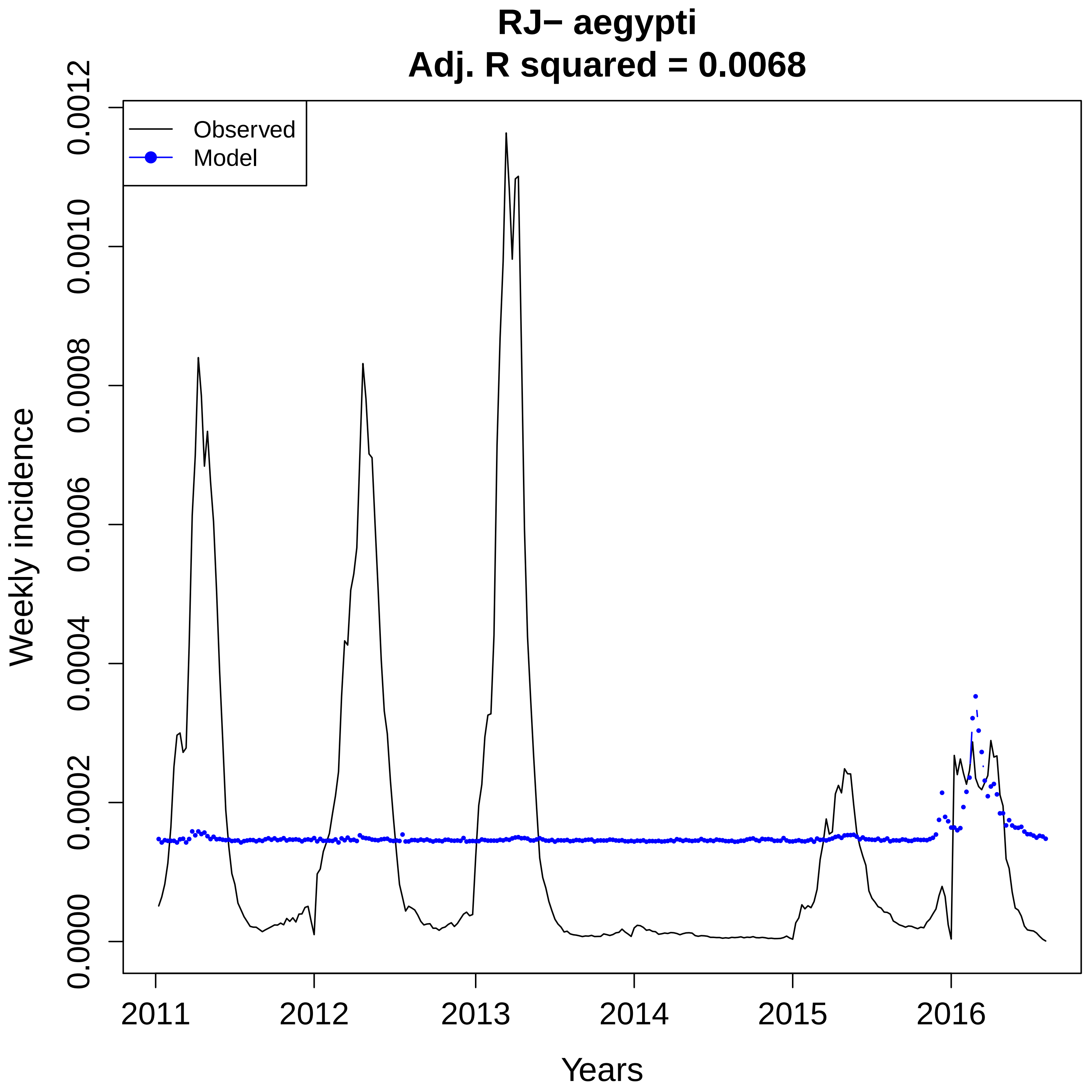
RJ− aegypti
Adj. R squared = 0.0068
Observed
Model
Weekly incidence
0.0000
0.0002
0.0004
0.0006
0.0008
0.0010
0.0012
2011
2012
2013
2014
2015
2016
Years

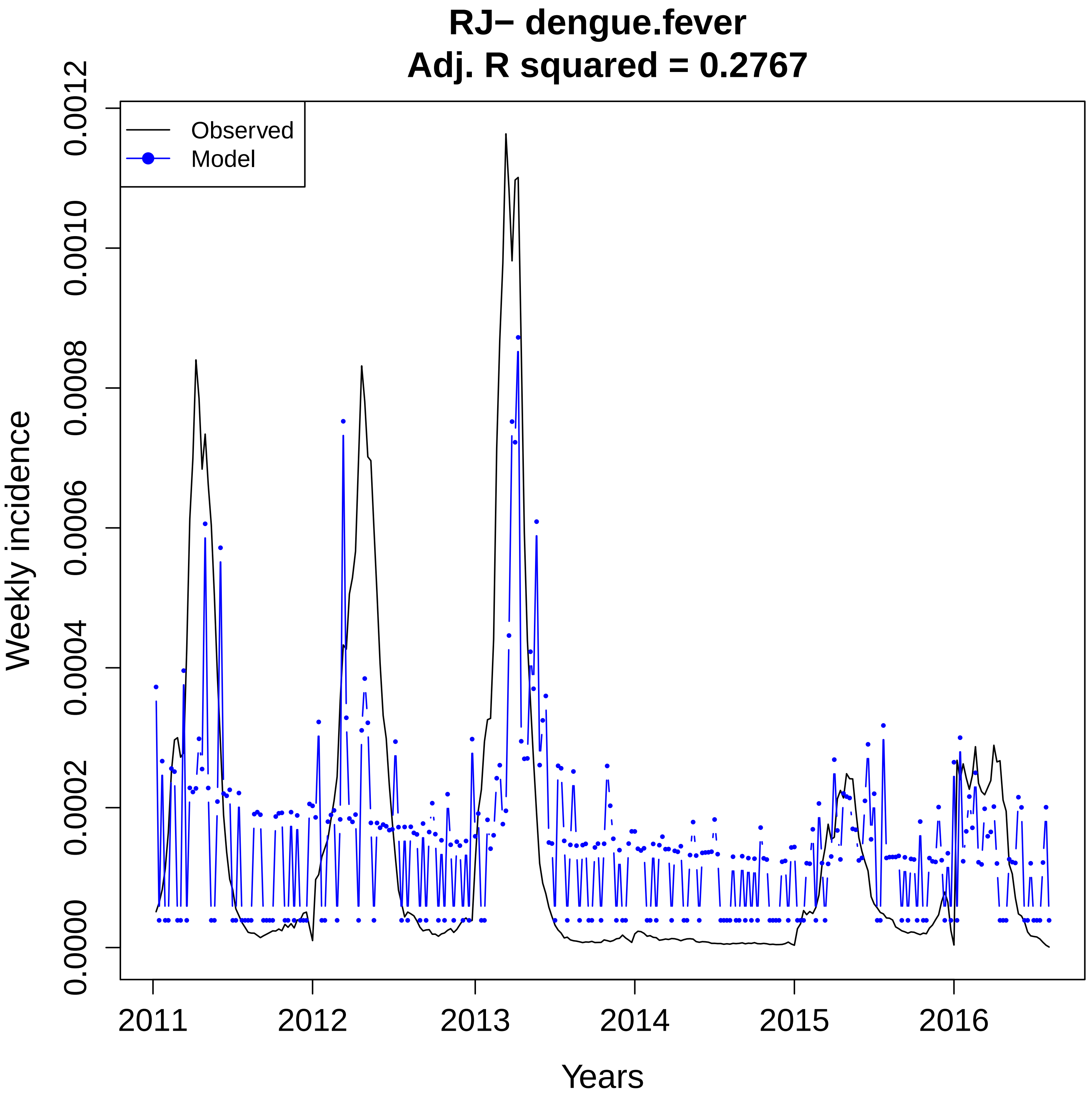

RJ– dengue.fever
Adj. R squared = 0.2767
Observed
Model
Weekly incidence
0.0000
0.0002
0.0004
0.0006
0.0008
0.0010
0.0012
2011
2012
2013
2014
2015
2016
Years

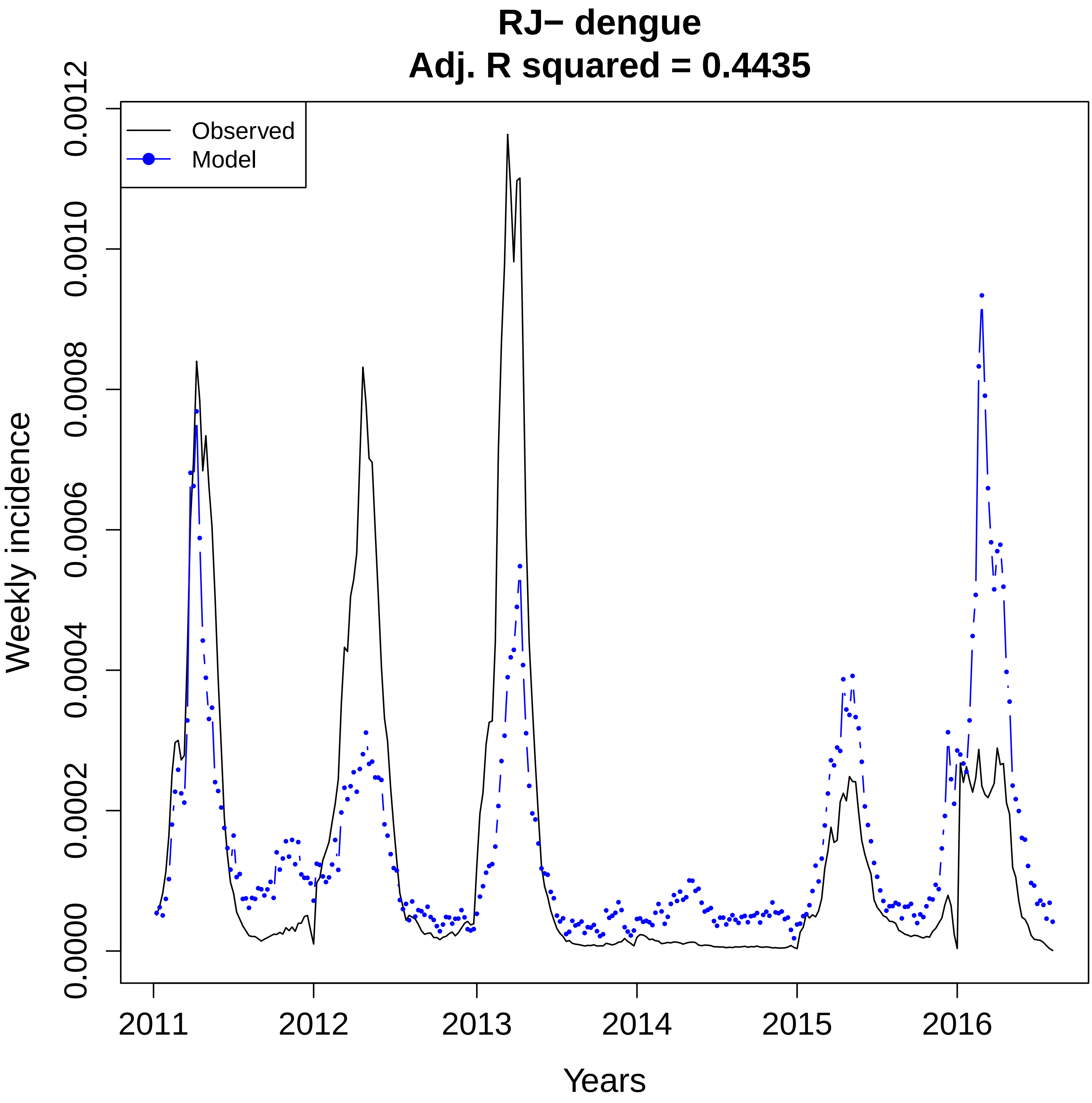
RJ− dengue
Adj. R squared = 0.4435
Observed
Model
Weekly incidence
0.0000
0.0002
0.0004
0.0006
0.0008
0.0010
0.0012
2011
2012
2013
2014
2015
2016
Years

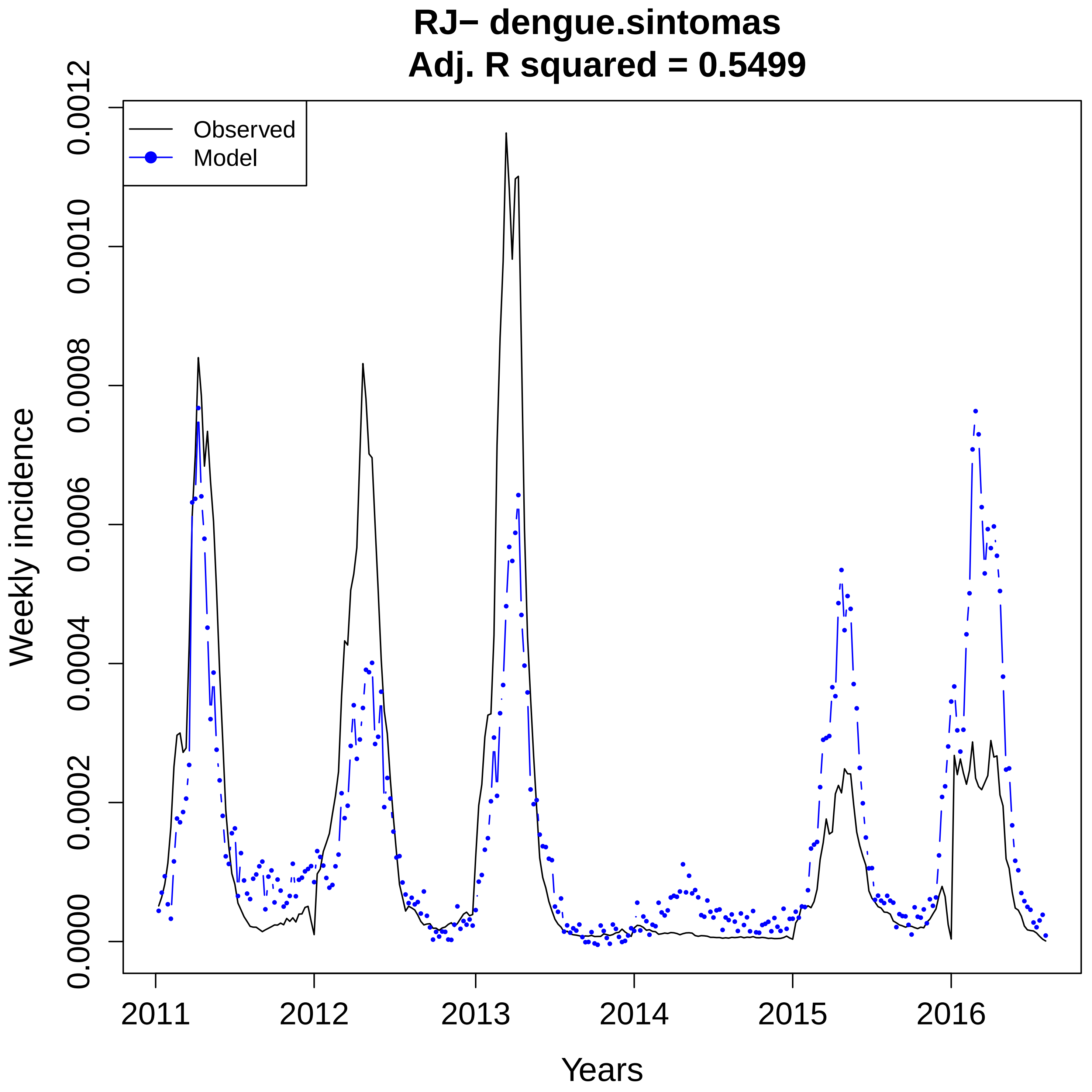

RJ− dengue.sintomas
Adj. R squared = 0.5499
Observed
Model
Weekly incidence
0.0000
0.0002
0.0004
0.0006
0.0008
0.0010
0.0012
2011
2012
2013
2014
2015
2016
Years

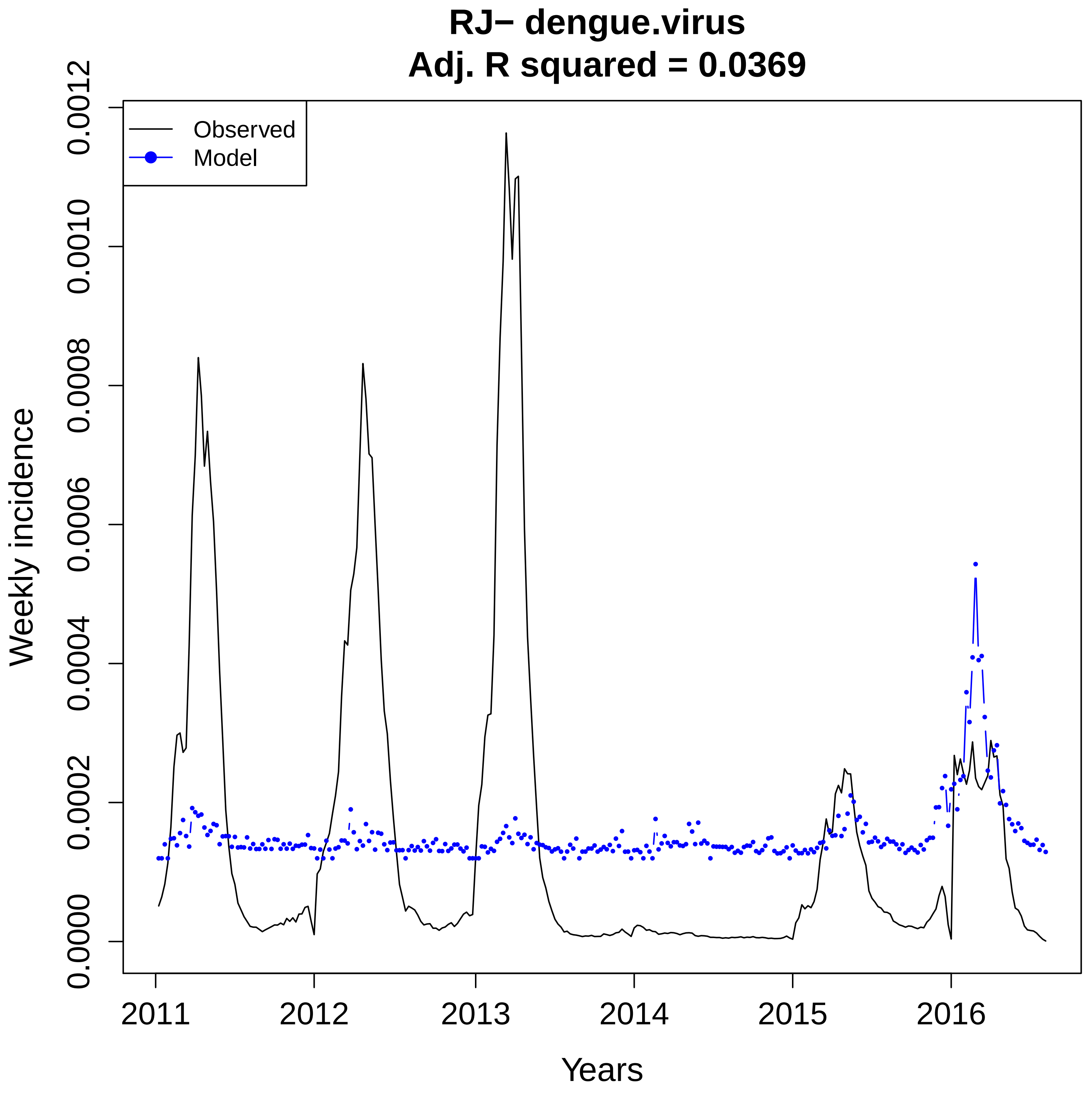
RJ− dengue.virus
Adj. R squared = 0.0369
Observed
Model
Weekly incidence
0.0000
0.0002
0.0004
0.0006
0.0008
0.0010
0.0012
2011
2012
2013
2014
2015
2016
Years

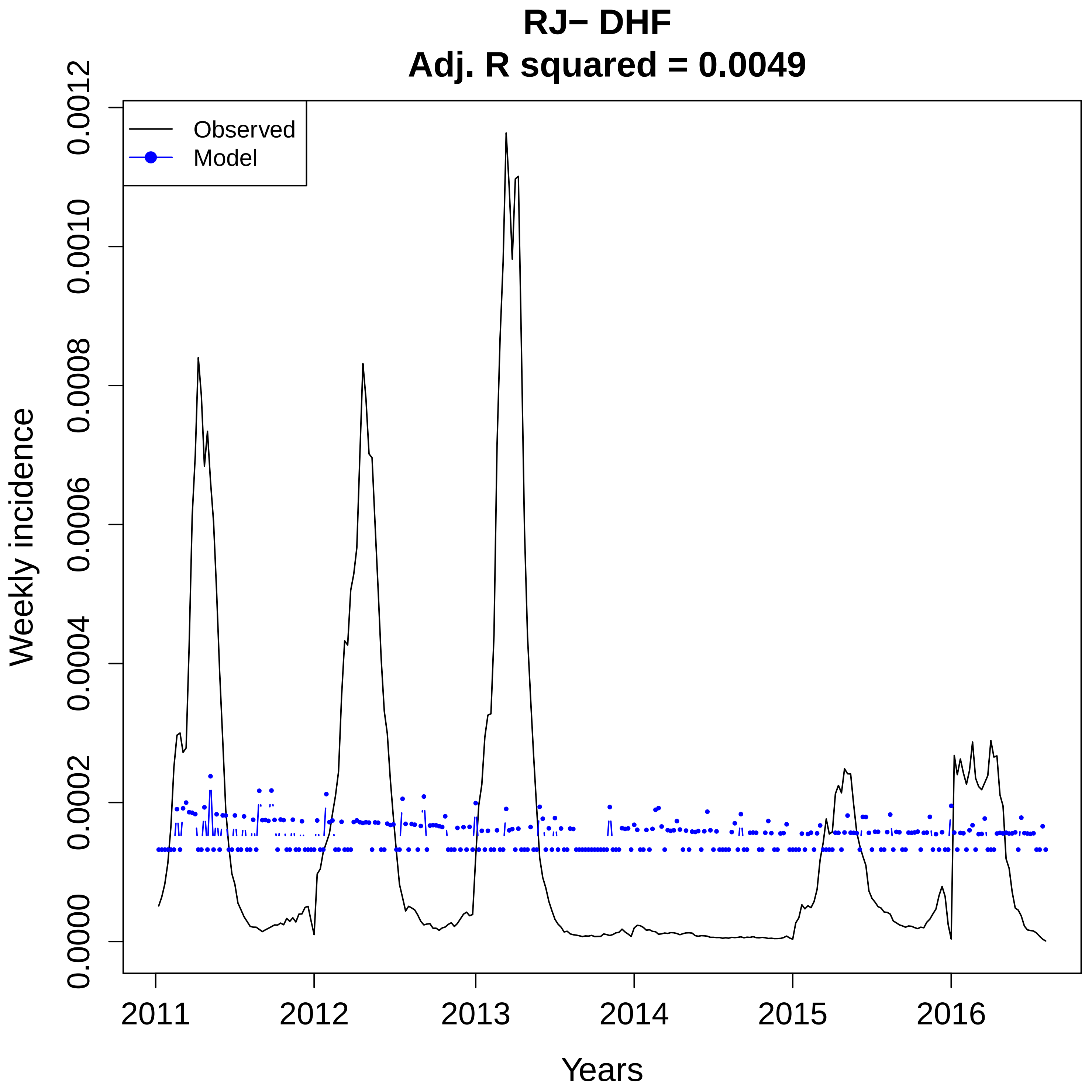

RJ− DHF
Adj. R squared = 0.0049
Observed
Model
Weekly incidence
0.0000
0.0002
0.0004
0.0006
0.0008
0.0010
0.0012
2011
2012
2013
2014
2015
2016
Years

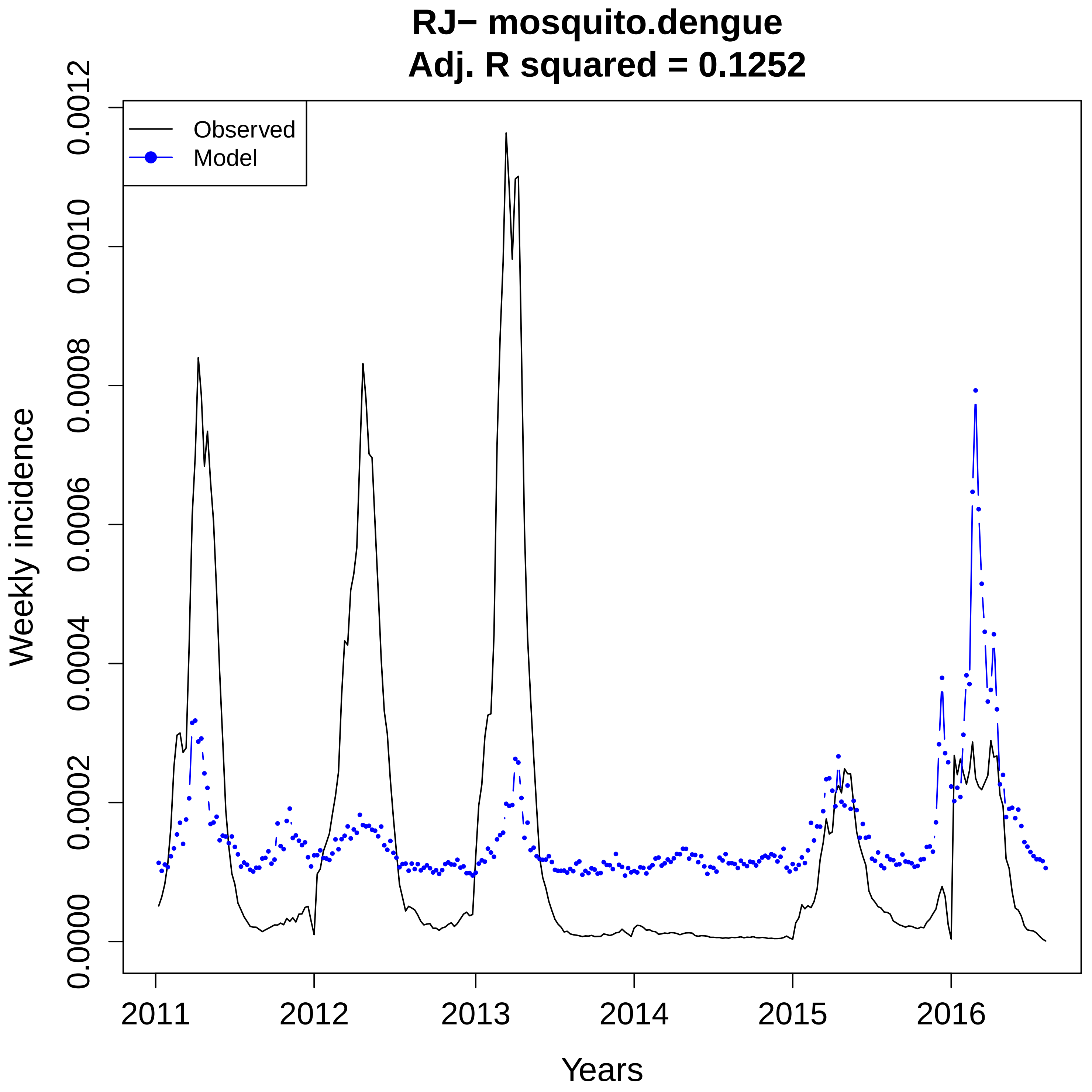

RJ− mosquito.dengue
Adj. R squared = 0.1252
Observed
Model
Weekly incidence
0.0000
0.0002
0.0004
0.0006
0.0008
0.0010
0.0012
2011
2012
2013
2014
2015
2016
Years

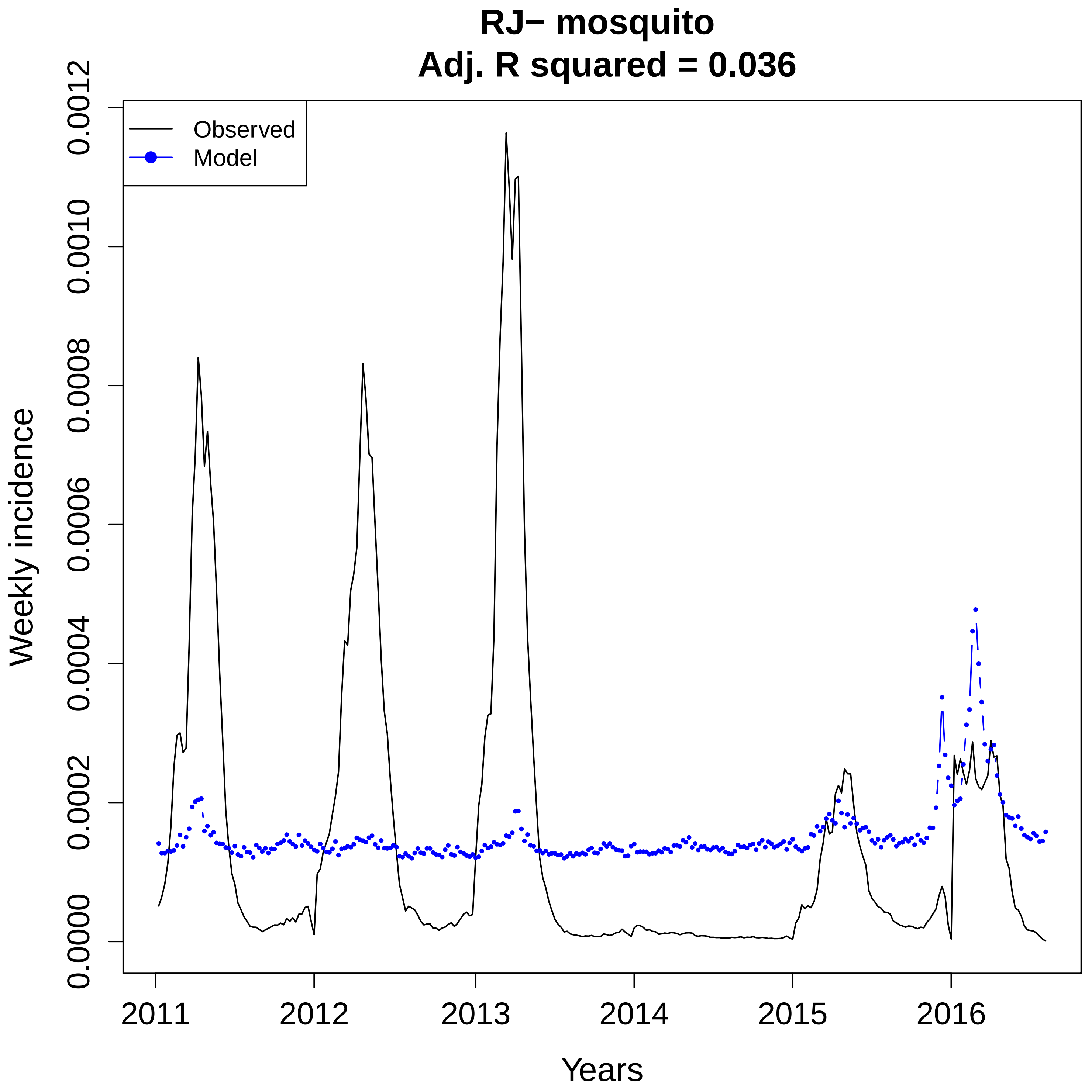
RJ− mosquito
Adj. R squared = 0.036
Observed
Model
Weekly incidence
0.0000
0.0002
0.0004
0.0006
0.0008
0.0010
0.0012
2011
2012
2013
2014
2015
2016
Years

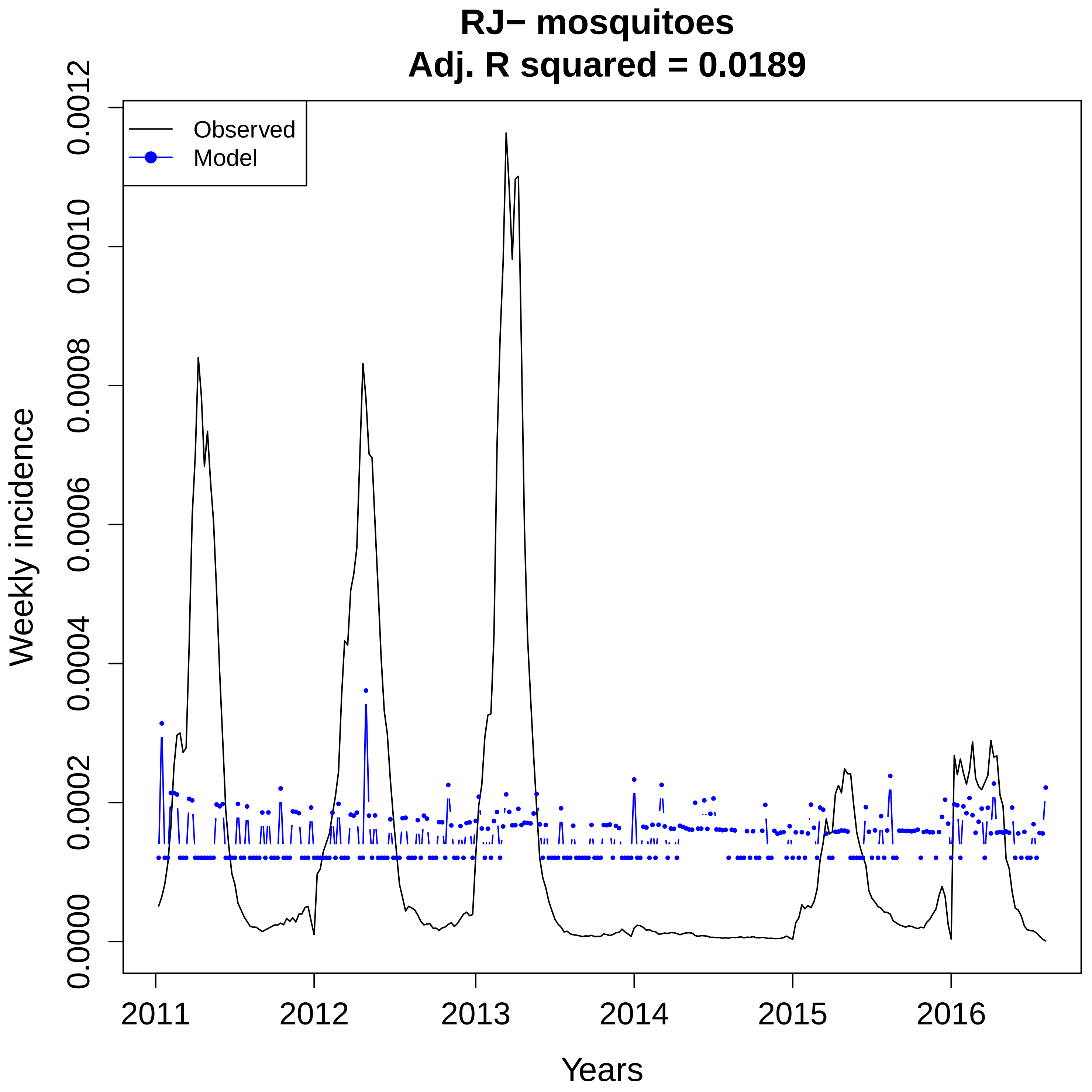
RJ− mosquitoes
Adj. R squared = 0.0189
Observed
Model
Weekly incidence
0.0000
0.0002
0.0004
0.0006
0.0008
0.0010
0.0012
2011
2012
2013
2014
2015
2016
Years

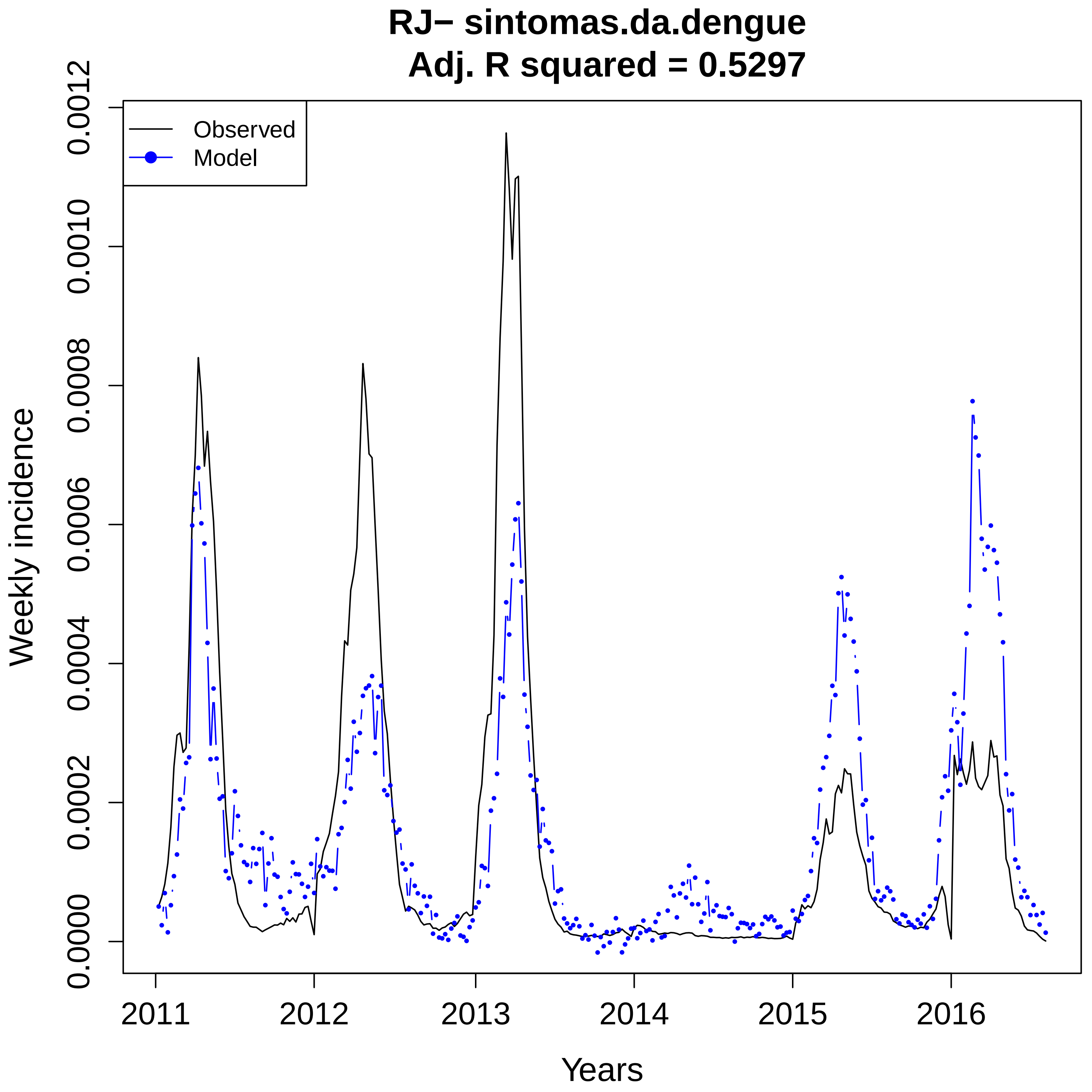

RJ− sintomas.da.dengue
Adj. R squared = 0.5297
Observed
Model
Weekly incidence
0.0000
0.0002
0.0004
0.0006
0.0008
0.0010
0.0012
2011
2012
2013
2014
2015
2016
Years

Rio Grande do Norte (RN)

Dengue incidence vs. Google Health Trends (2011–2016)

Definitions:

- **Combined terms:**
  - **All:** model with all the available terms. n= number of terms
  - **Four:** model with "dengue", "dengue sintomas", "aedes", and "mosquito".
  - **Uncorrelated:** model with non-correlated terms. n = number of uncorrelated terms
- **Individual terms:** Plots developed with one term at a time.

- Terms for all and uncorrelated models can be found in additional file 3.

Combined terms
Adjusted R squared

Rio Grande do Norte (RN)

All
(n = 9)

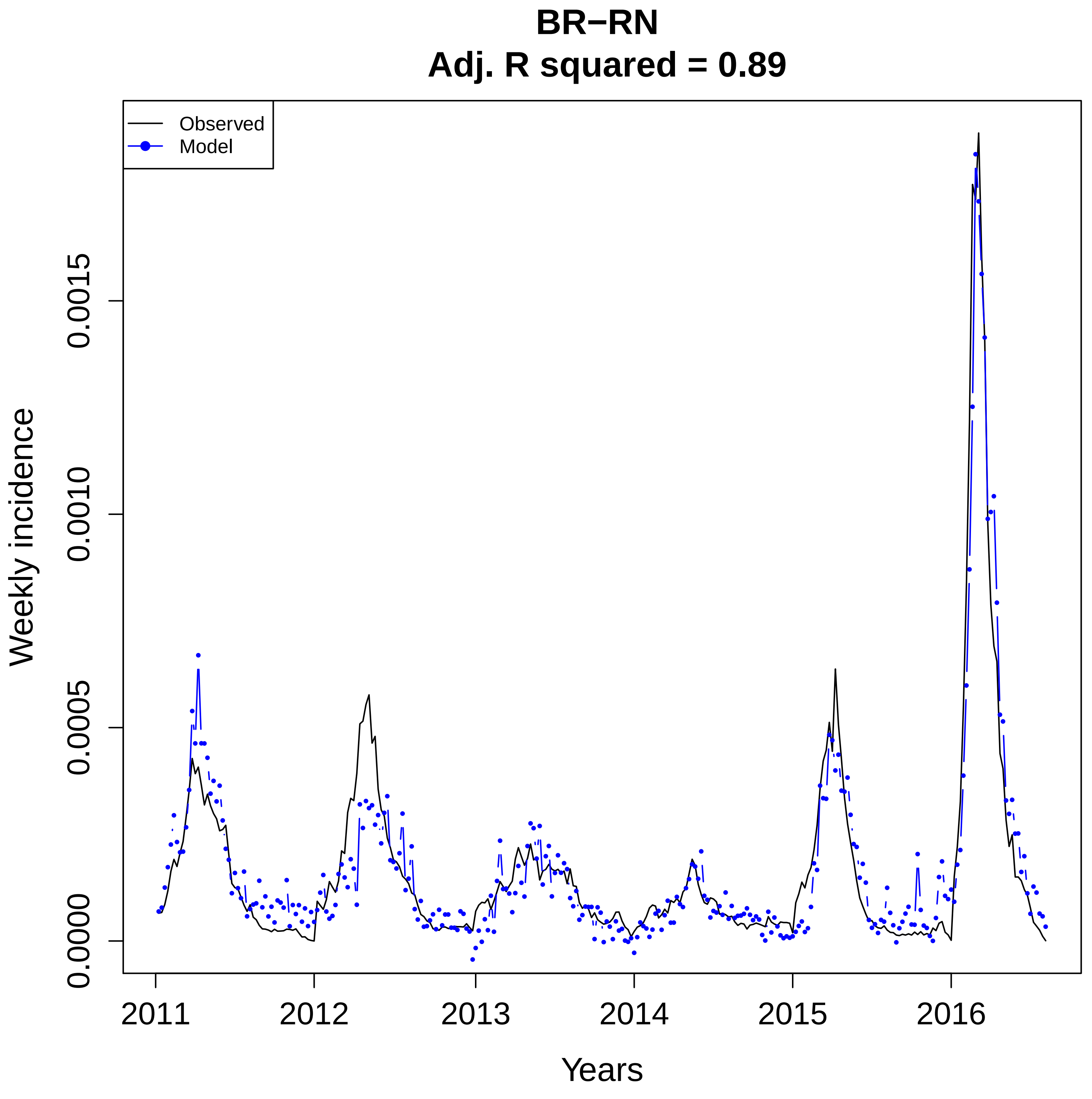
BR−RN
Adj. R squared = 0.89
Observed
Model
Weekly incidence
0.0000
0.0005
0.0010
0.0015
2011
2012
2013
2014
2015
2016
Years

Four

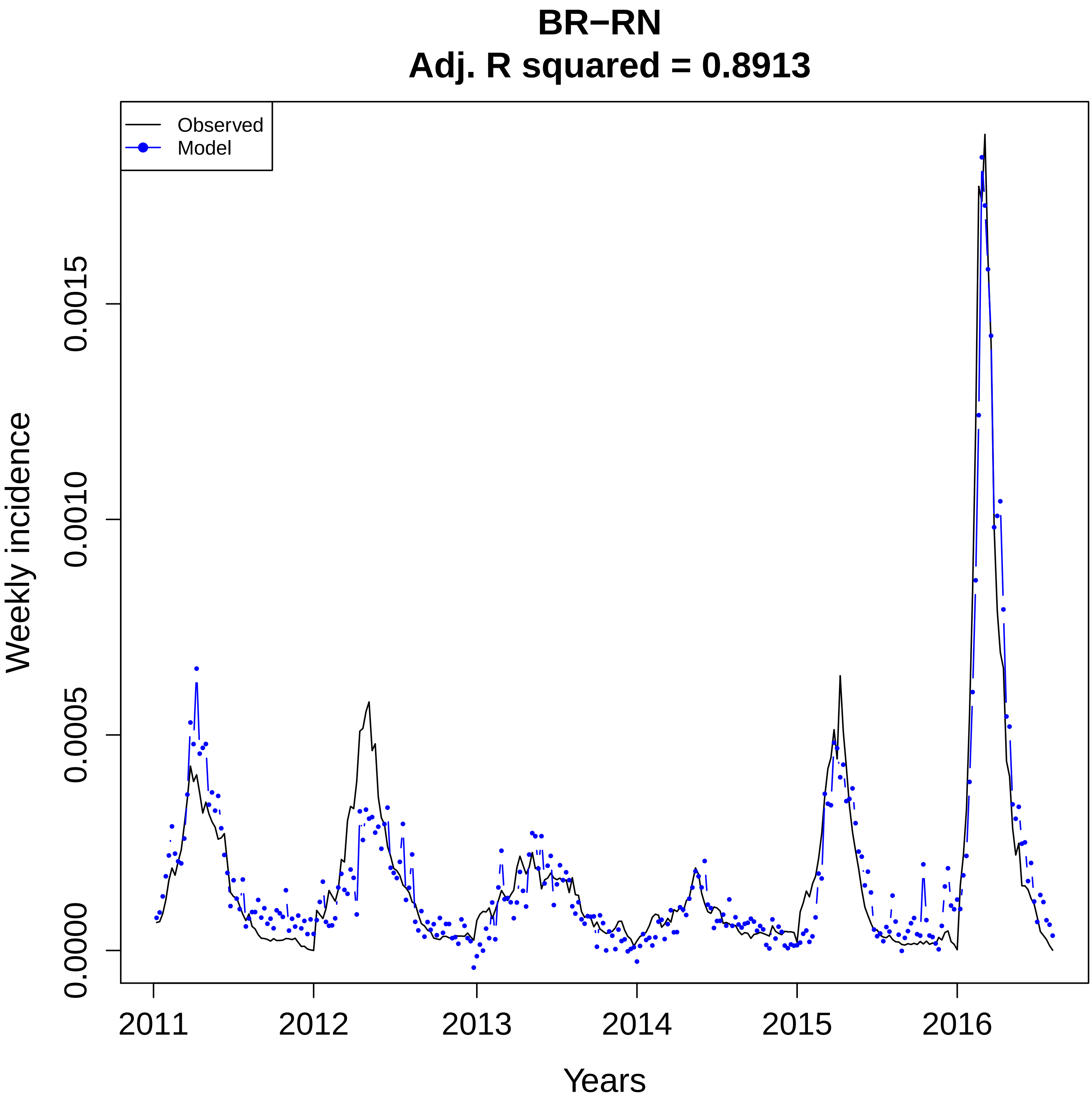

BR−RN
Adj. R squared = 0.8913
Observed
Model
Weekly incidence
0.0000
0.0005
0.0010
0.0015
2011
2012
2013
2014
2015
2016
Years

Uncorrelated
(n = 2)

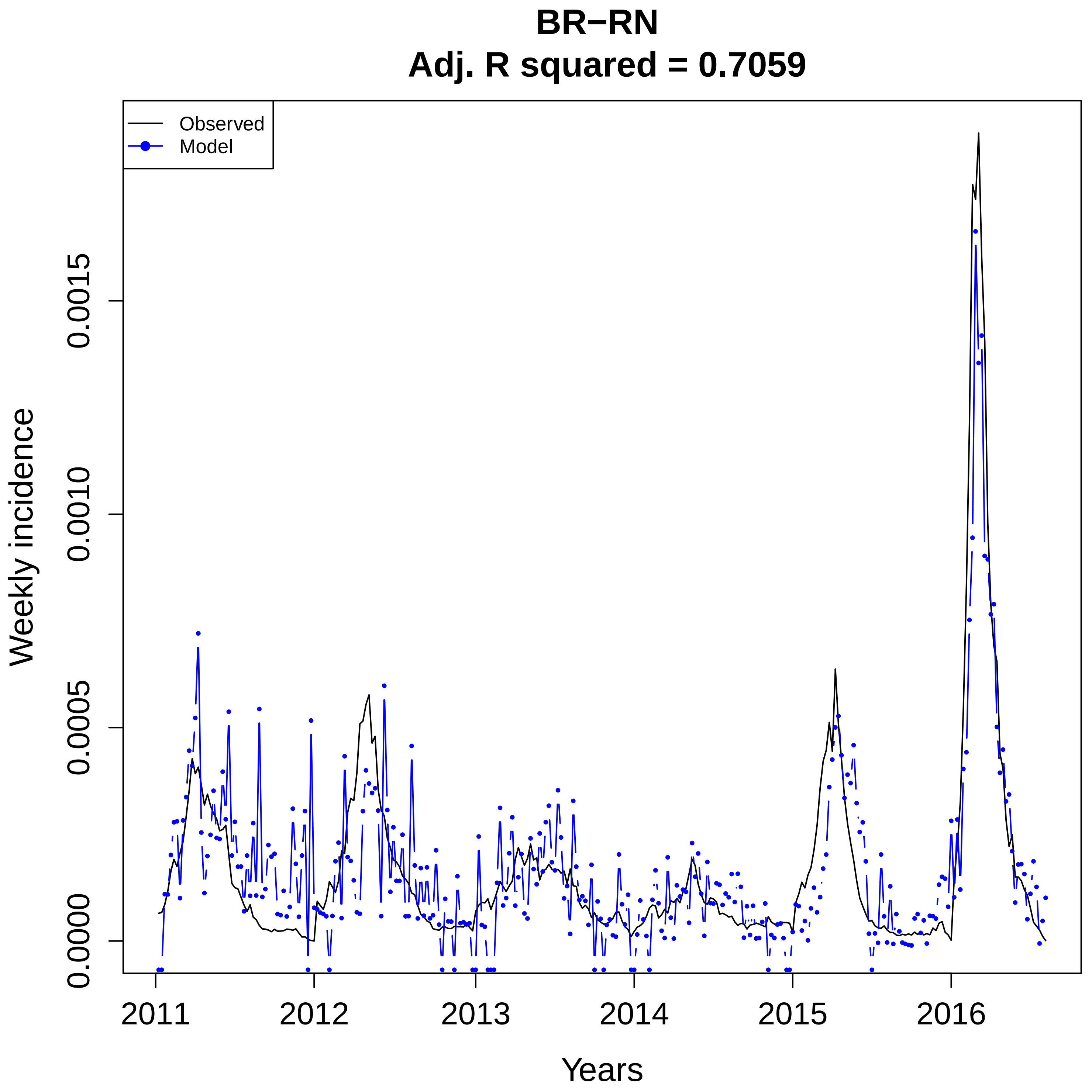

Individual terms
Adjusted R squared

Rio Grande do
Norte (RN)

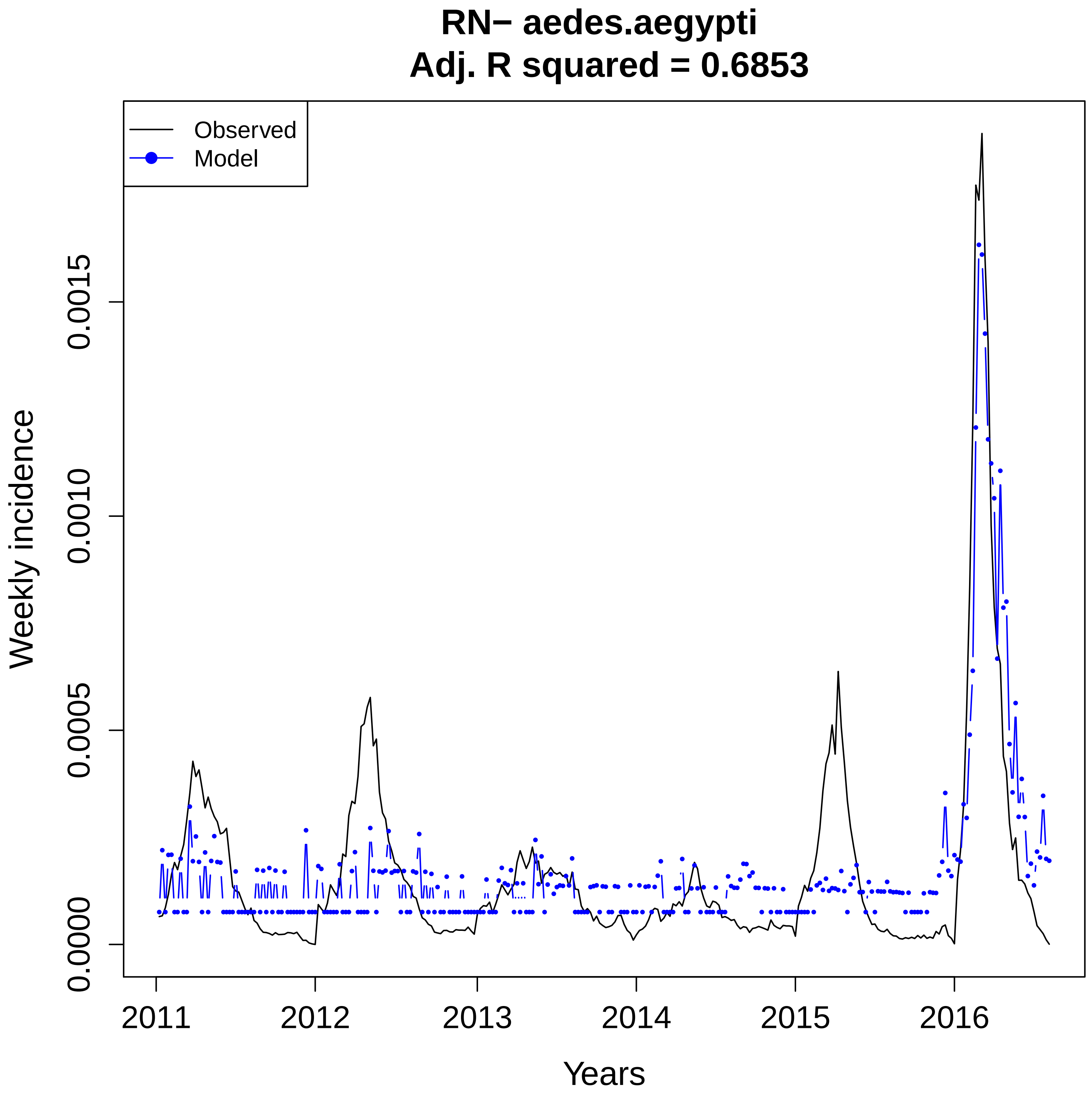

RN− aedes.aegypti
Adj. R squared = 0.6853
Observed
Model
Weekly incidence
0.0000
0.0005
0.0010
0.0015
2011
2012
2013
2014
2015
2016
Years

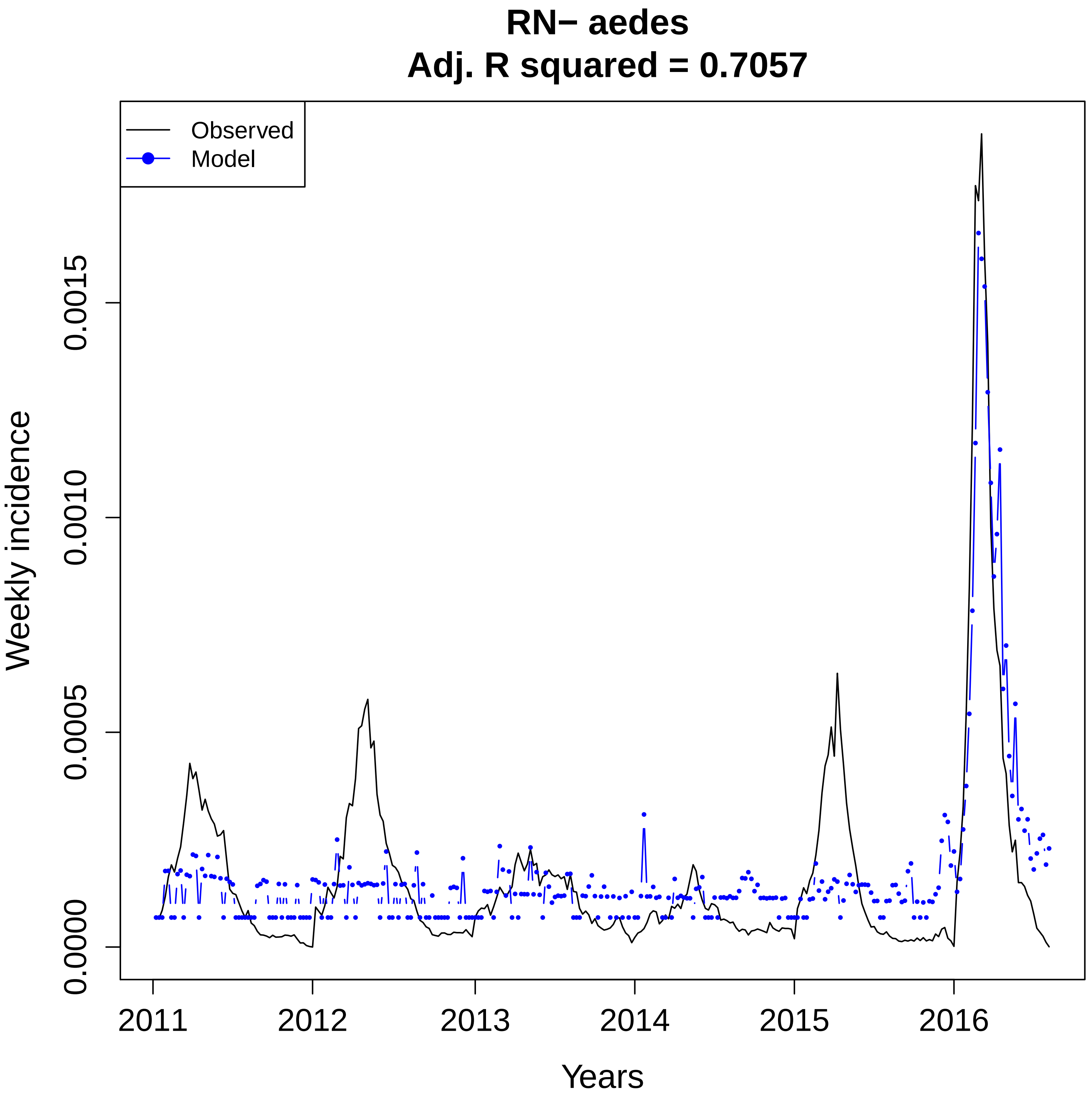
RN− aedes
Adj. R squared = 0.7057
Observed
Model
Weekly incidence
0.0000
0.0005
0.0010
0.0015
2011
2012
2013
2014
2015
2016
Years

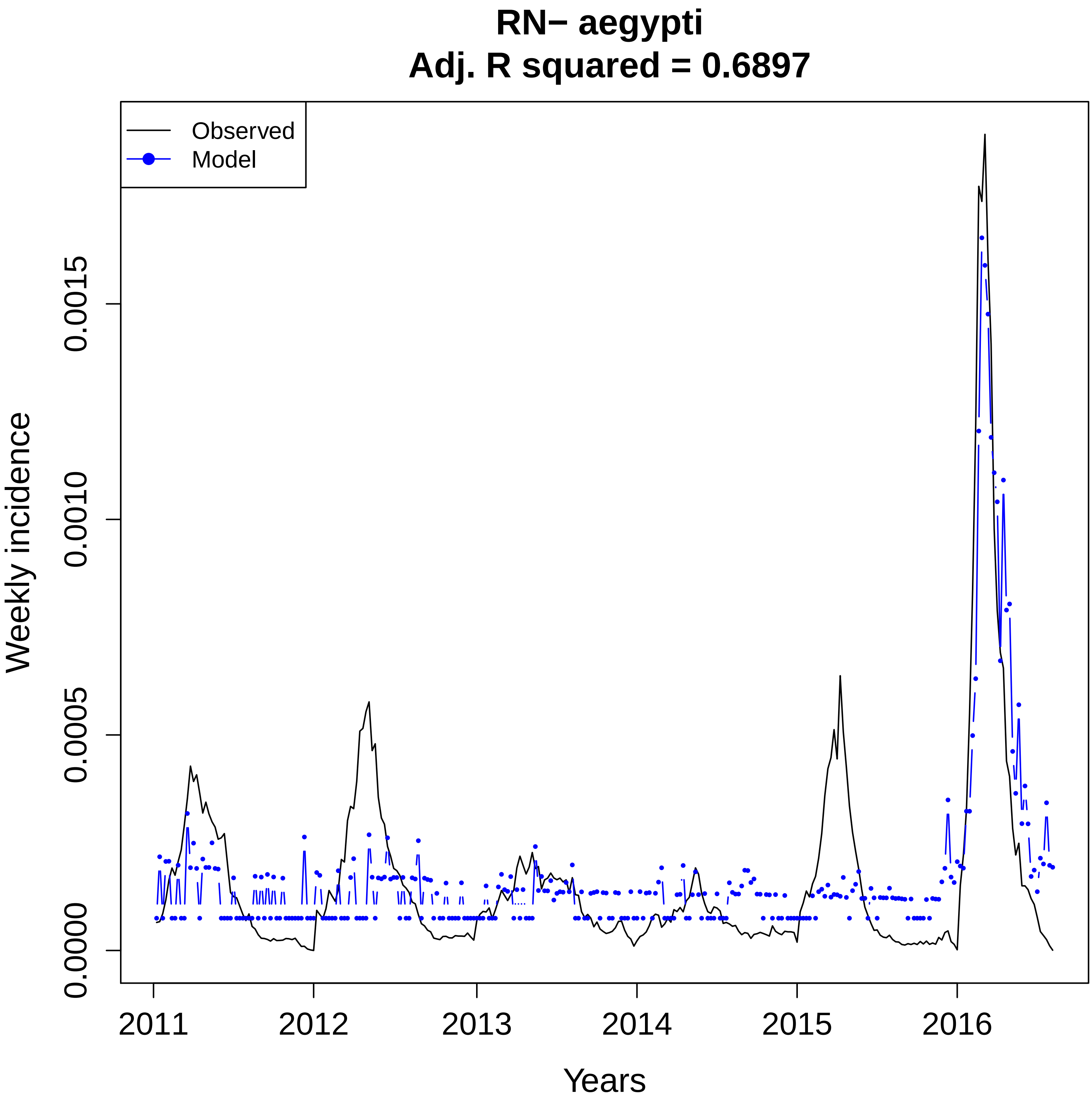
RN− aegypti
Adj. R squared = 0.6897
Observed
Model
Weekly incidence
0.0000
0.0005
0.0010
0.0015
2011
2012
2013
2014
2015
2016
Years

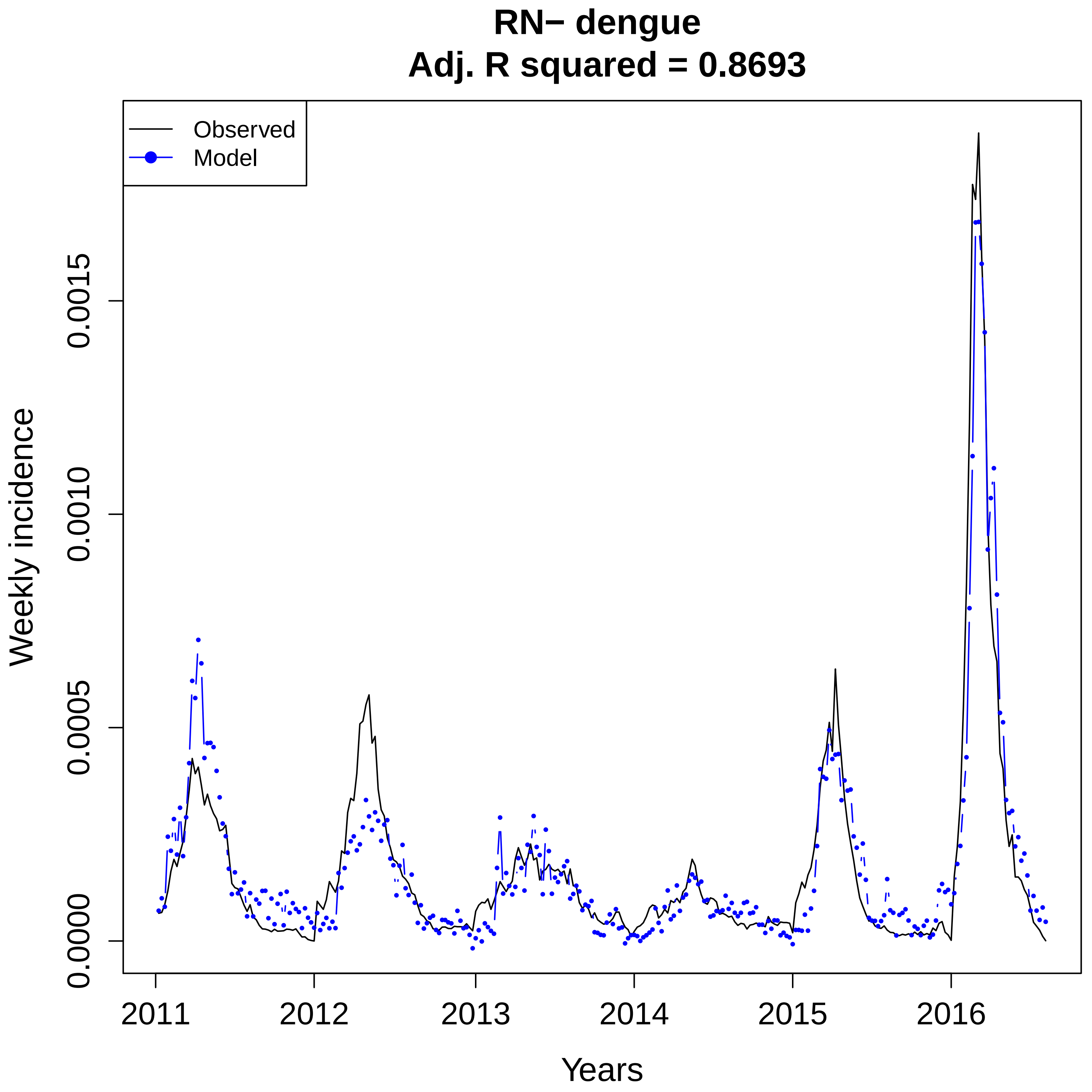

RN− dengue
Adj. R squared = 0.8693
Observed
Model
Weekly incidence
0.0000
0.0005
0.0010
0.0015
2011
2012
2013
2014
2015
2016
Years

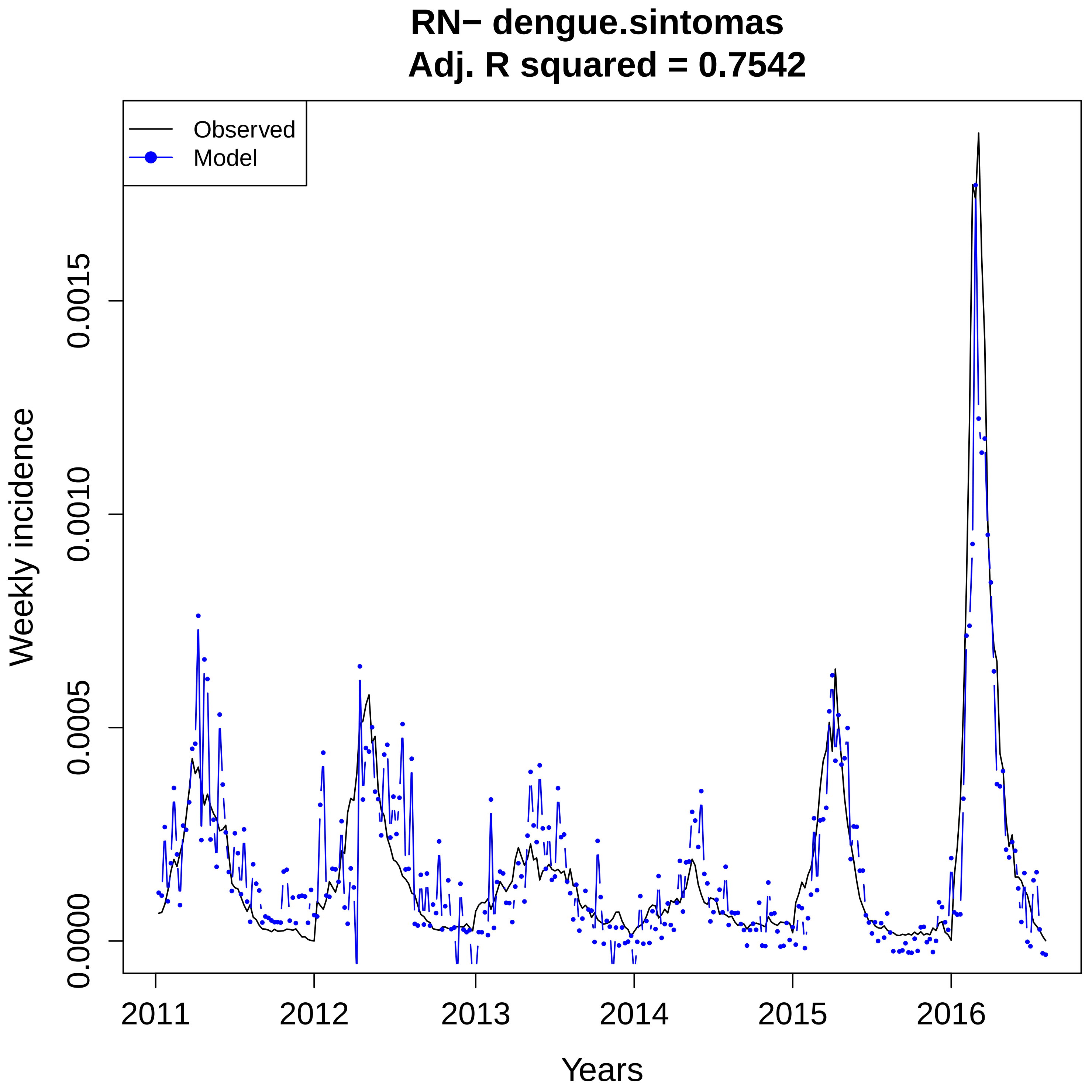

RN− dengue.sintomas
Adj. R squared = 0.7542
Observed
Model
Weekly incidence
0.0000
0.0005
0.0010
0.0015
2011
2012
2013
2014
2015
2016
Years

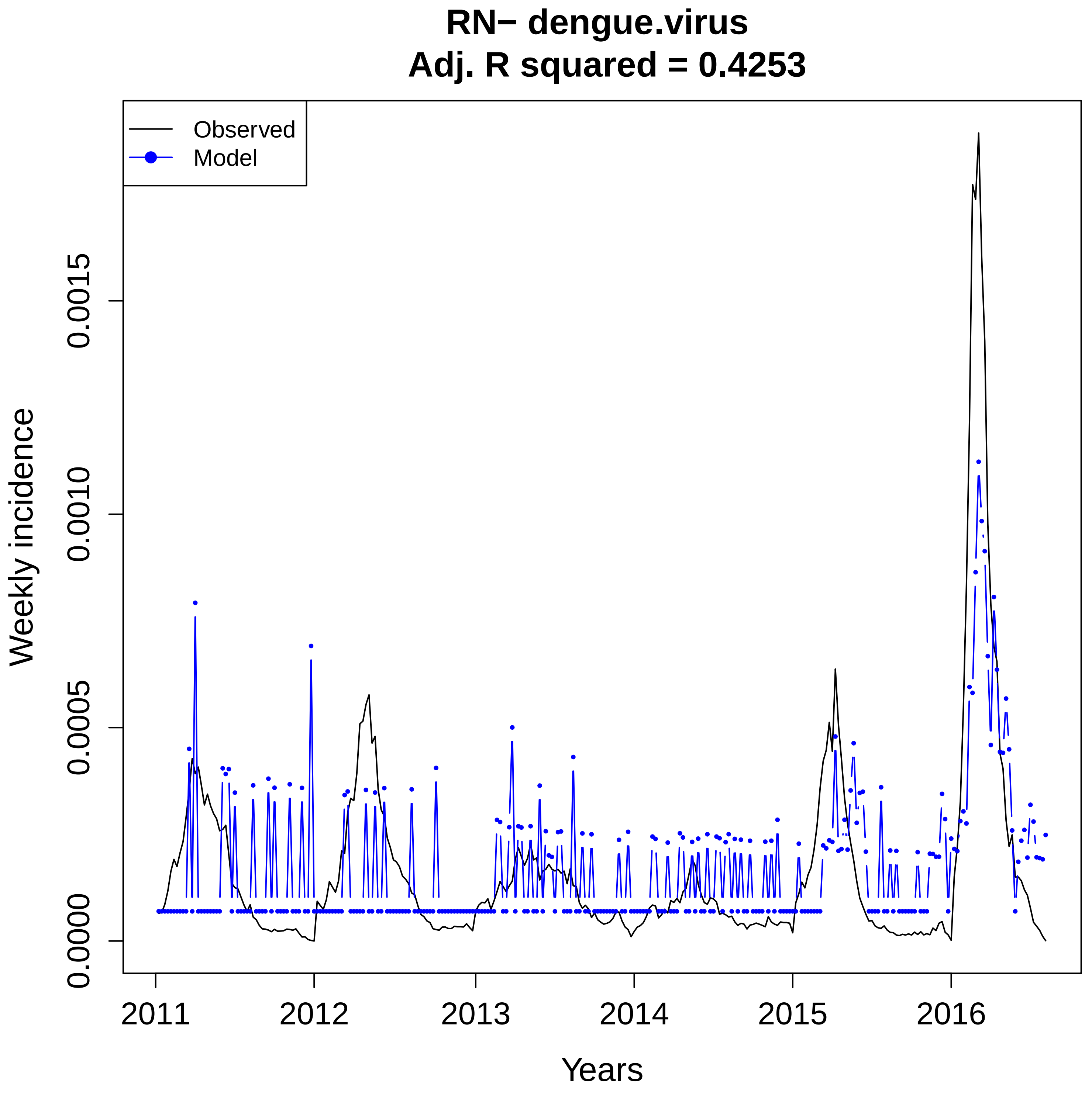

RN− dengue.virus
Adj. R squared = 0.4253
Observed
Model
Weekly incidence
0.0000
0.0005
0.0010
0.0015
2011
2012
2013
2014
2015
2016
Years

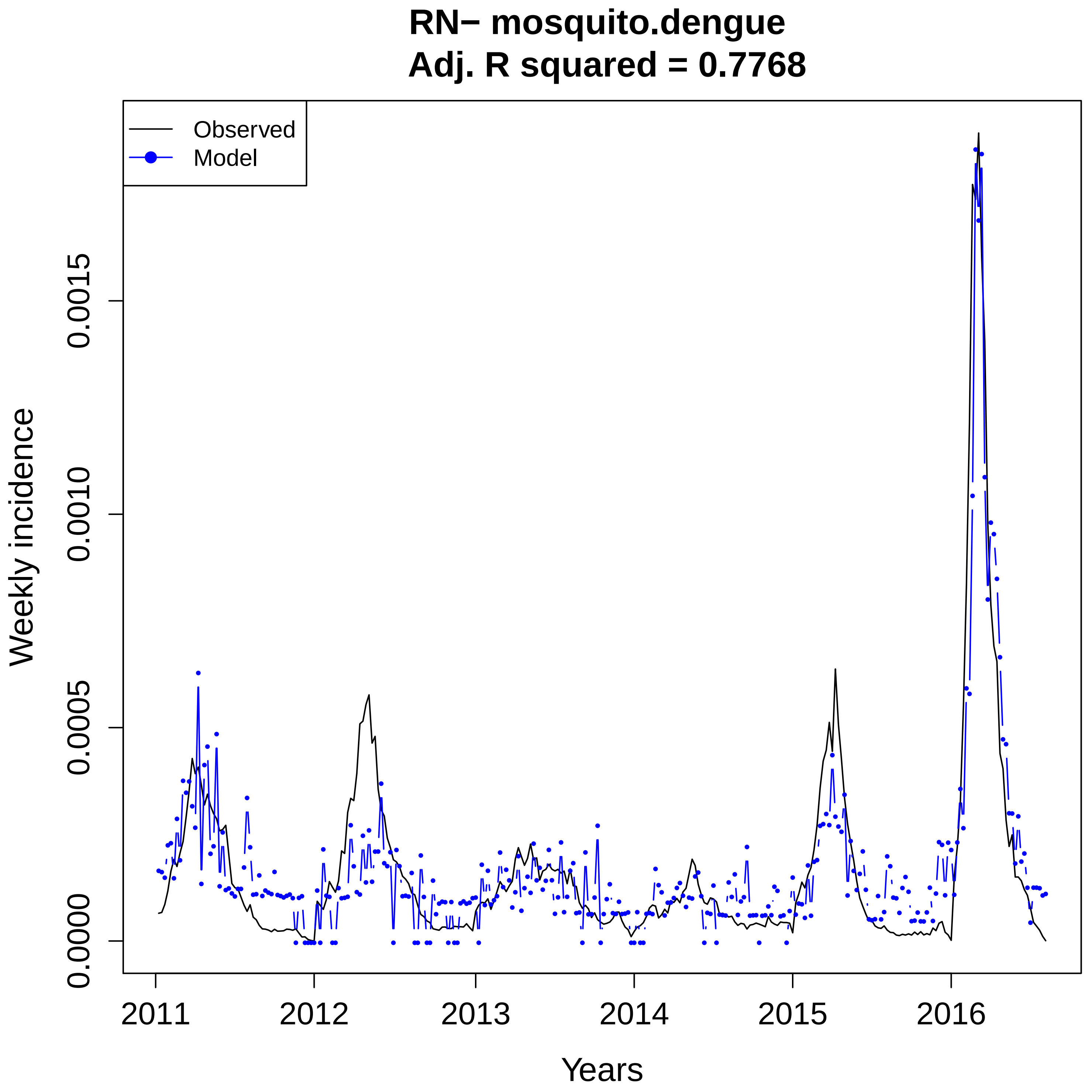

RN− mosquito.dengue
Adj. R squared = 0.7768
Observed
Model
Weekly incidence
0.0000
0.0005
0.0010
0.0015
2011
2012
2013
2014
2015
2016
Years

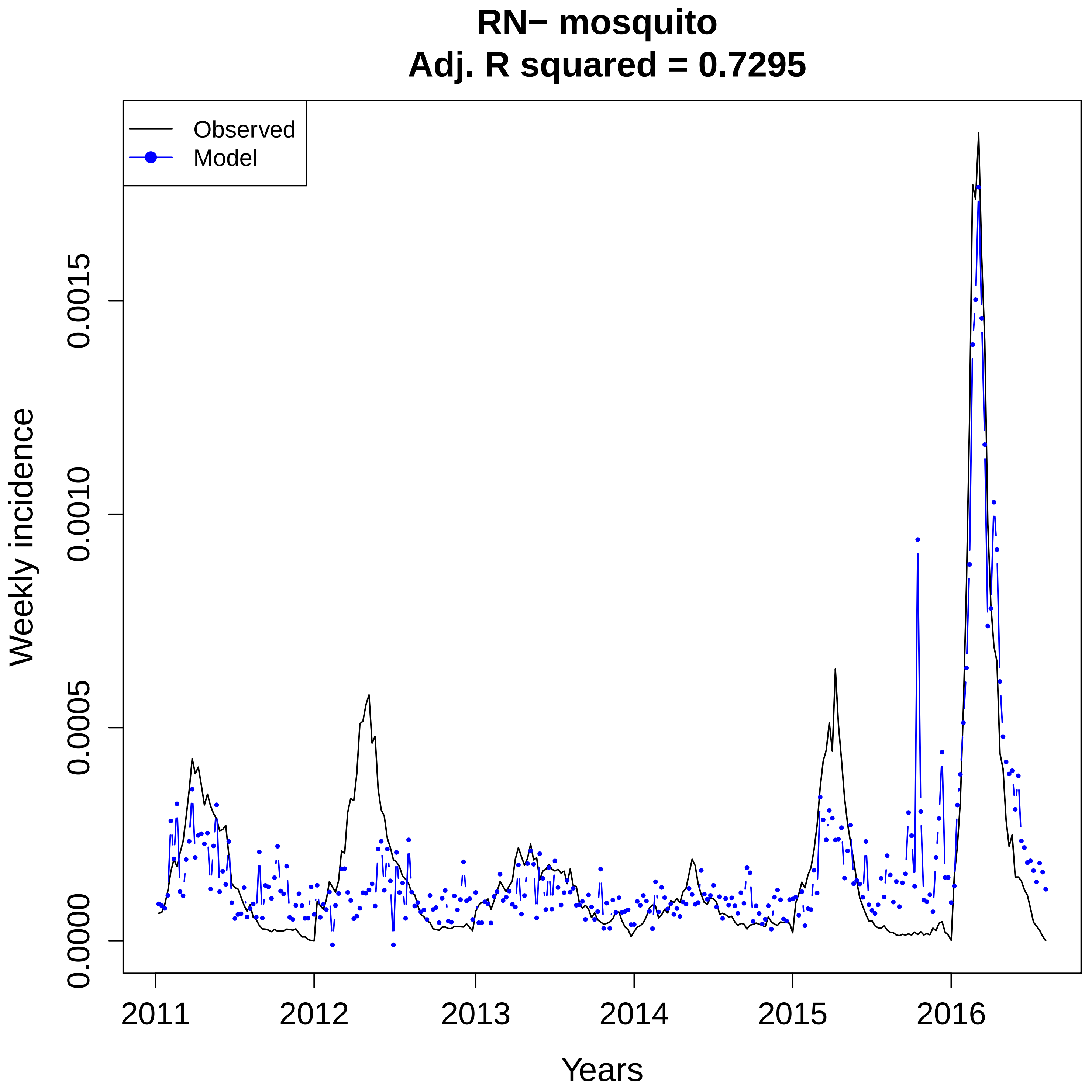
RN− mosquito
Adj. R squared = 0.7295
Observed
Model
Weekly incidence
0.0000
0.0005
0.0010
0.0015
2011
2012
2013
2014
2015
2016
Years

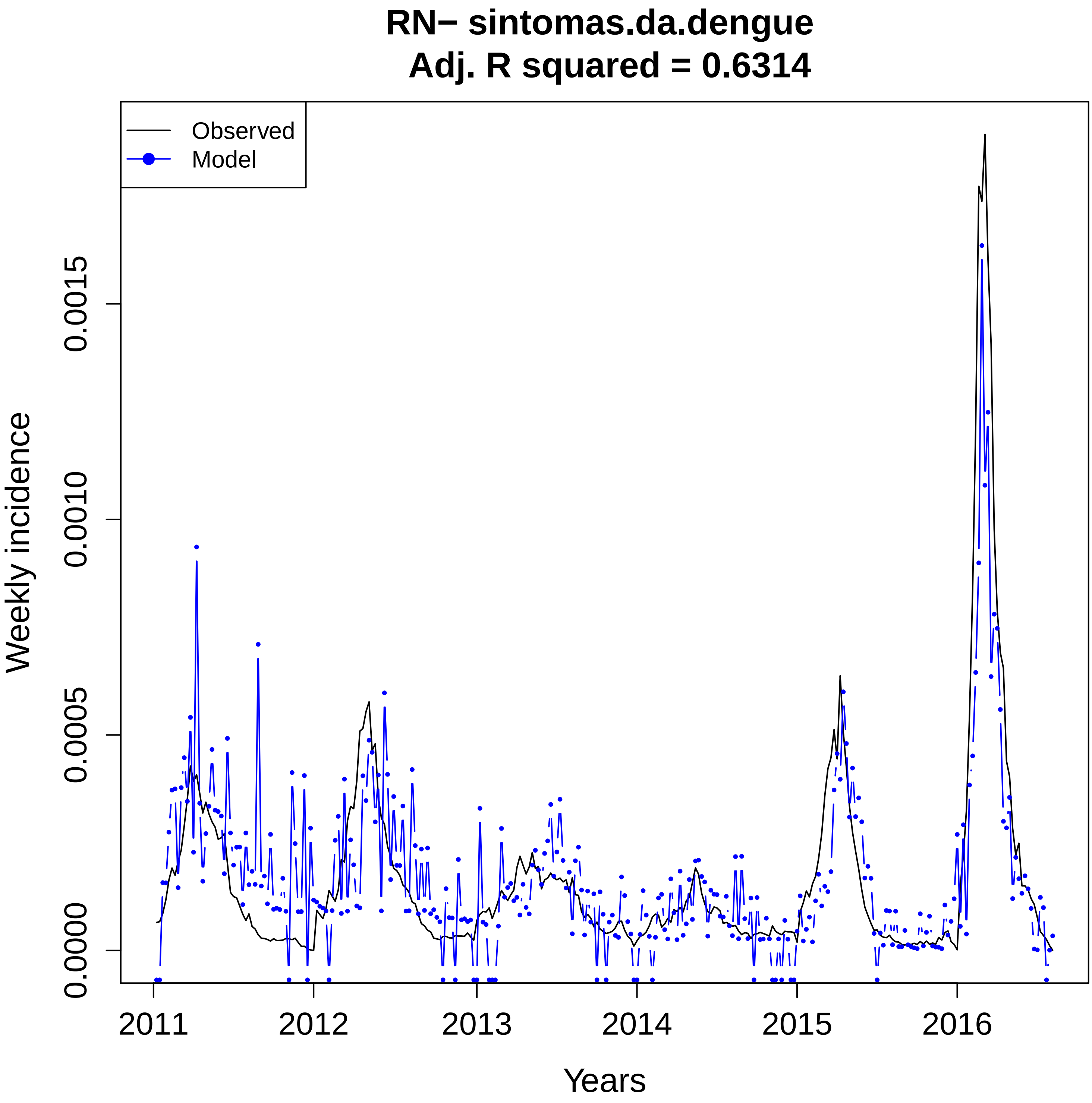
RN− sintomas.da.dengue
Adj. R squared = 0.6314
Observed
Model
Weekly incidence
0.0000
0.0005
0.0010
0.0015
2011
2012
2013
2014
2015
2016
Years

Rôndonia (RO)

Dengue incidence vs. Google Health Trends (2011–2016)

Definitions:

- **Combined terms:**
  - **All:** model with all the available terms. n= number of terms
  - **Four:** model with "dengue", "dengue sintomas", "aedes", and "mosquito".
  - **Uncorrelated:** model with non-correlated terms. n = number of uncorrelated terms
- **Individual terms:** Plots developed with one term at a time.

- Terms for all and uncorrelated models can be found in additional file 3.

Combined terms
Adjusted R squared

Rôndonia (RO)

All
(n = 9)

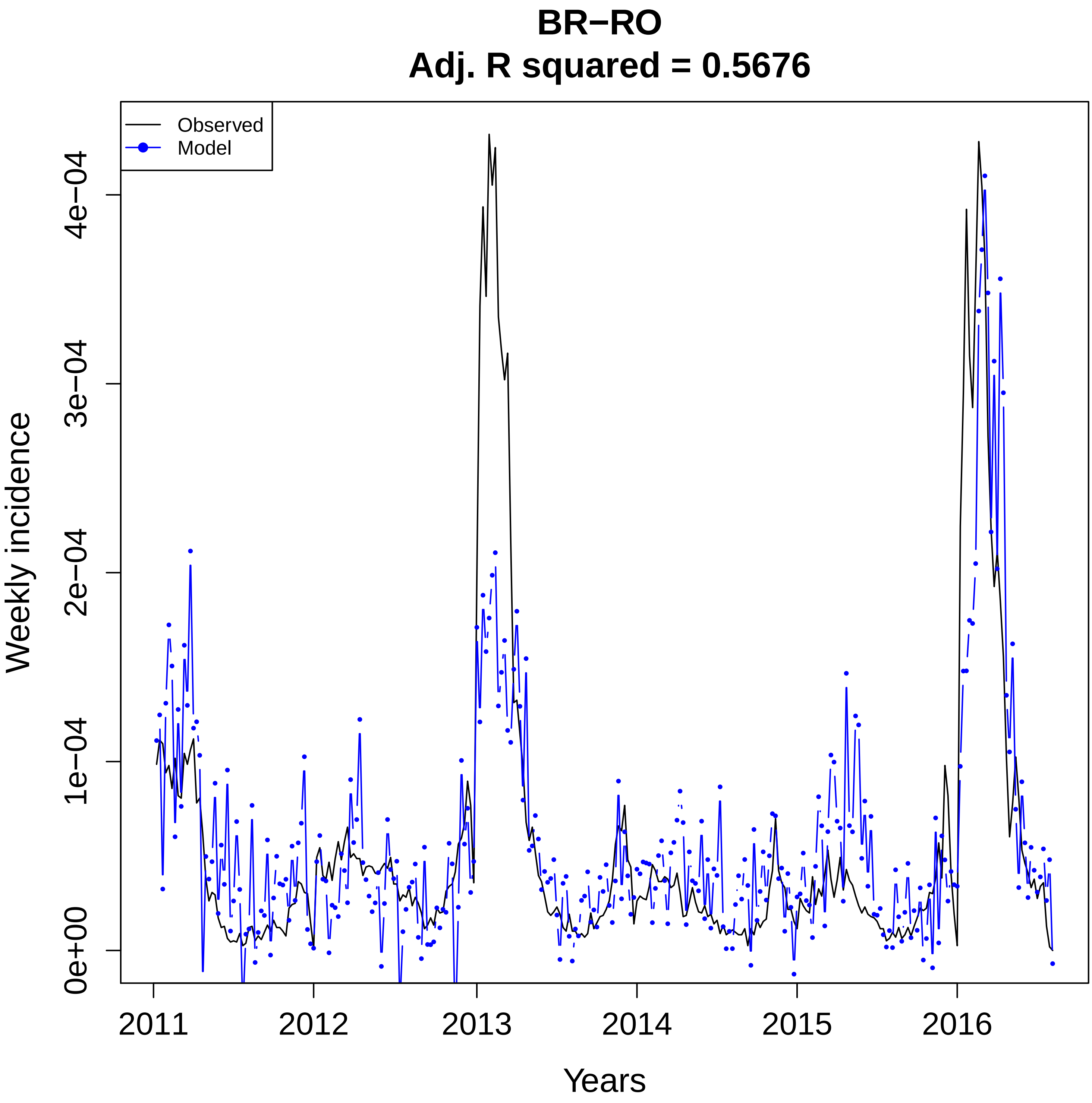
BR−RO
Adj. R squared = 0.5676
Observed
Model
Weekly incidence
0e+00
1e−04
2e−04
3e−04
4e−04
2011
2012
2013
2014
2015
2016
Years

Four

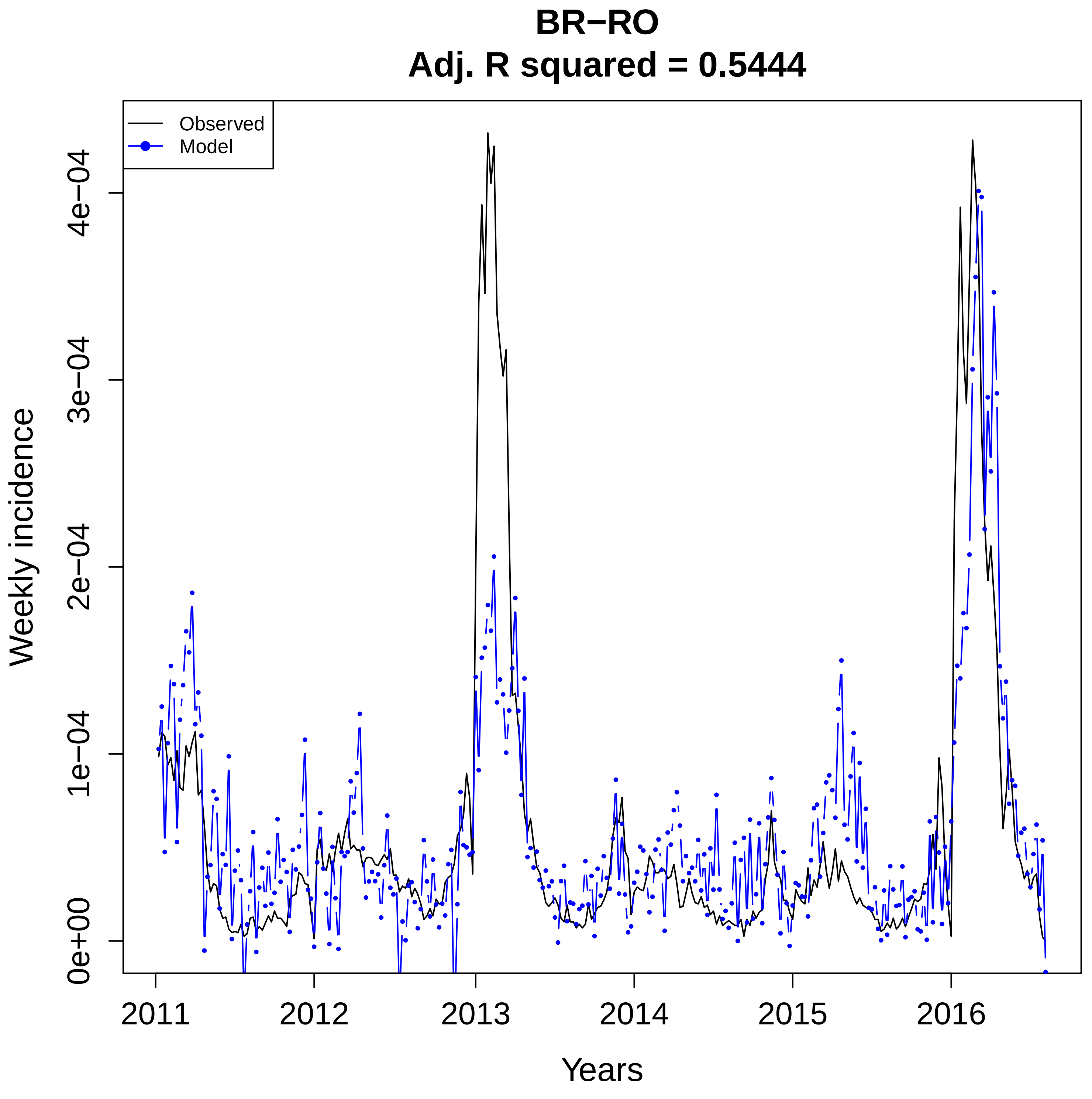

BR−RO
Adj. R squared = 0.5444
Observed
Model
Weekly incidence
0e+00
1e−04
2e−04
3e−04
4e−04
2011
2012
2013
2014
2015
2016
Years

Uncorrelated
(n = 4)

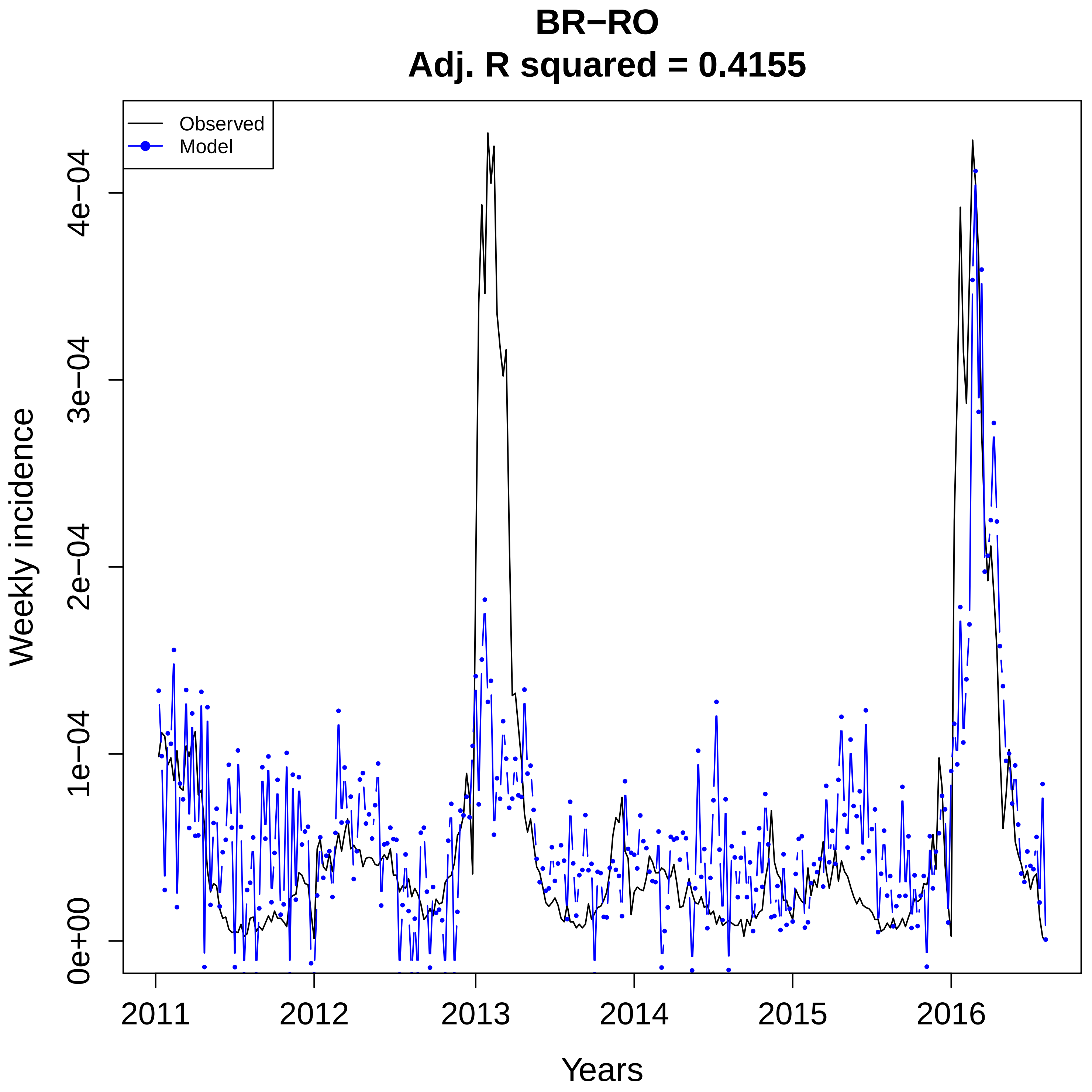

Individual terms
Adjusted R squared

Rôndonia (RO)

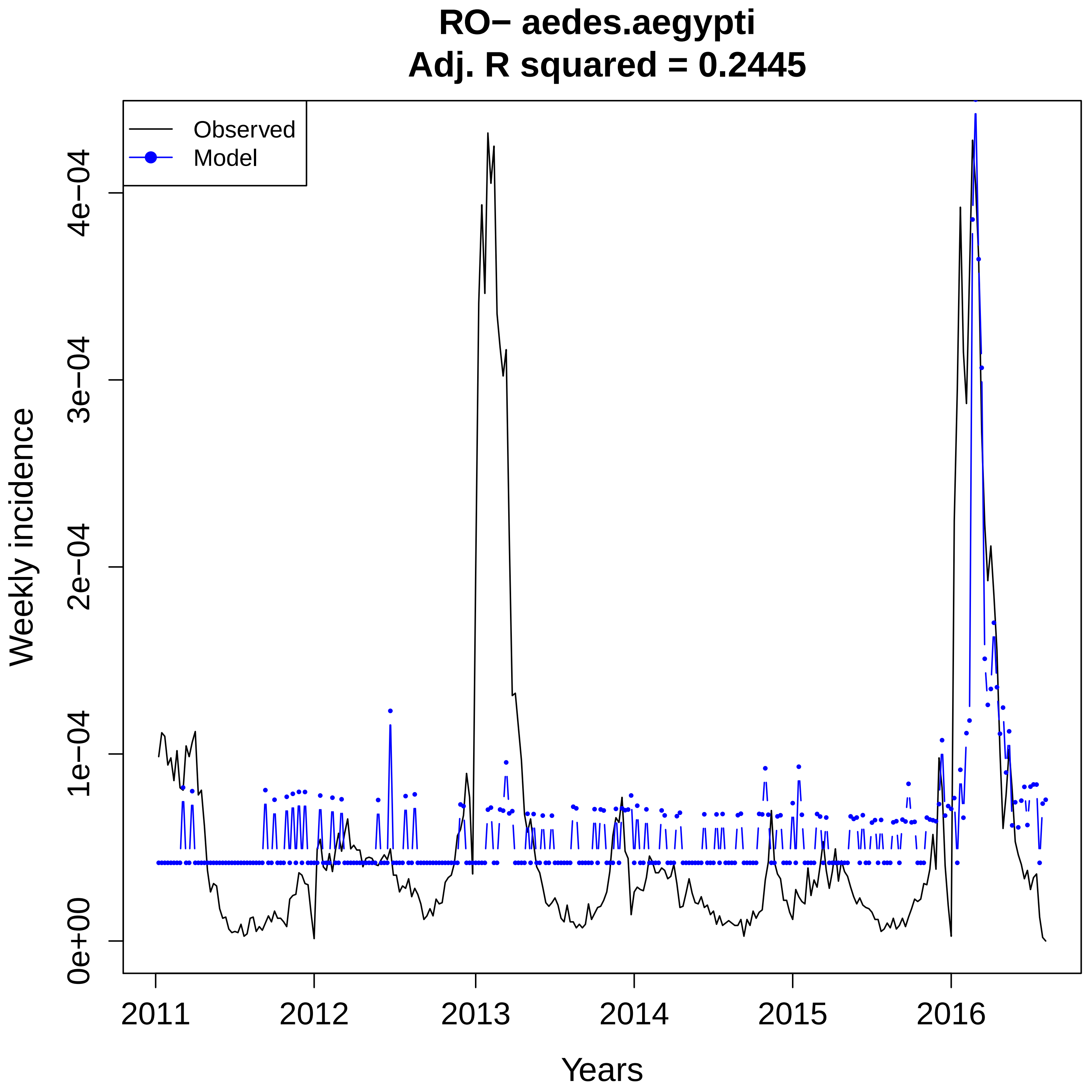

RO− aedes.aegypti
Adj. R squared = 0.2445
Observed
Model
Weekly incidence
0e+00
1e−04
2e−04
3e−04
4e−04
Years
2011
2012
2013
2014
2015
2016

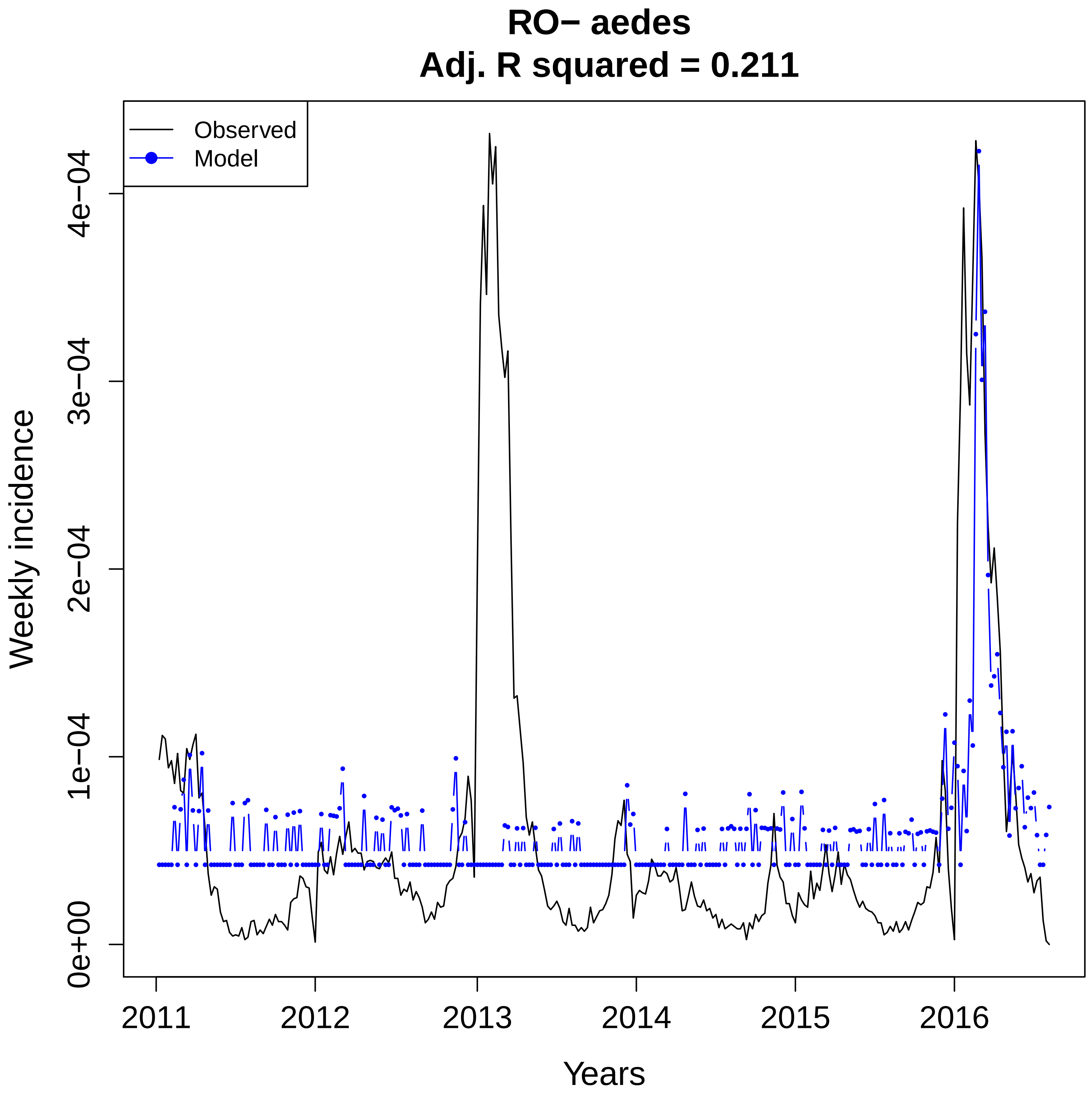
RO− aedes
Adj. R squared = 0.211
Observed
Model
Weekly incidence
0e+00
1e−04
2e−04
3e−04
4e−04
2011
2012
2013
2014
2015
2016
Years

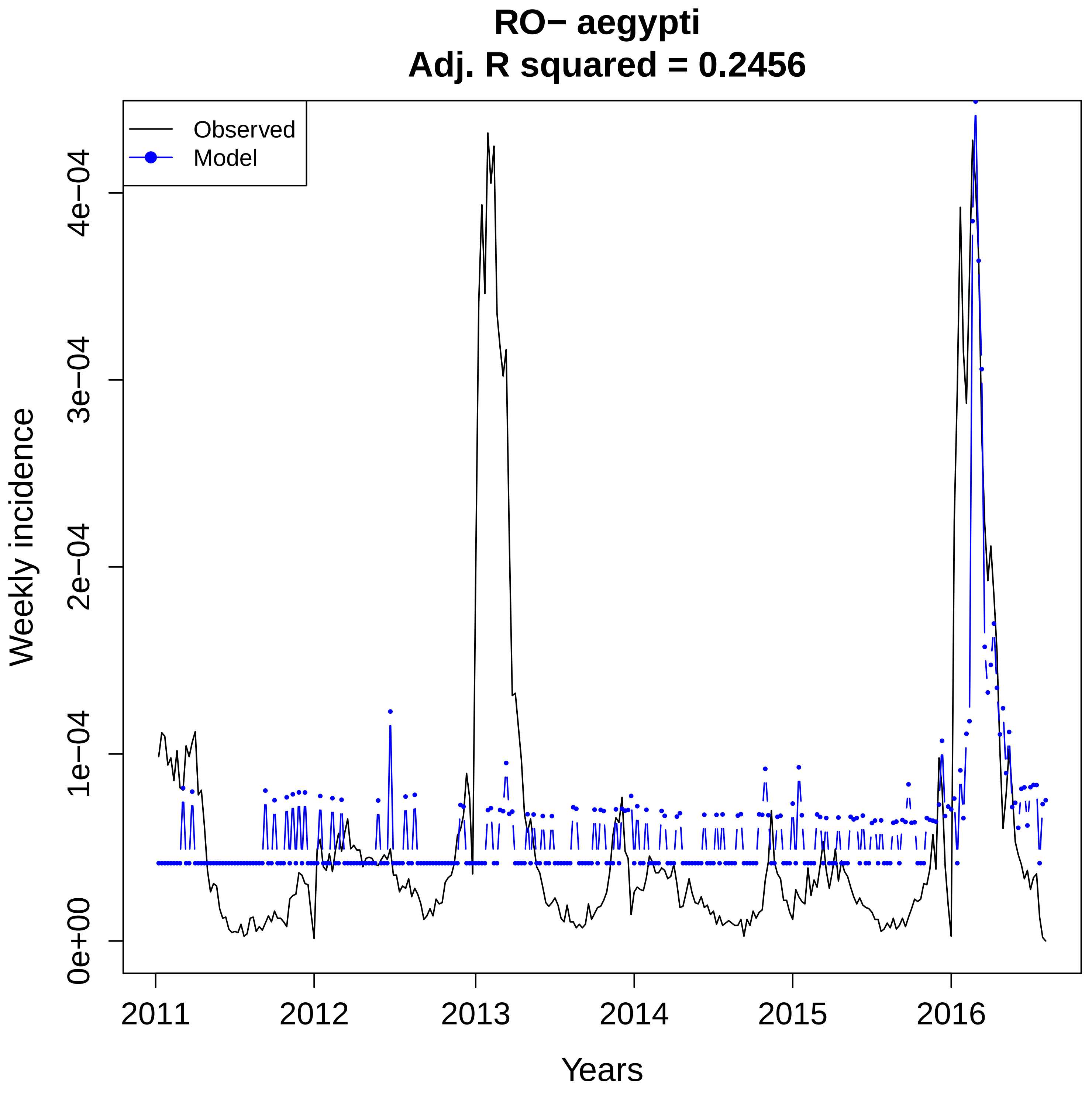
RO− aegypti
Adj. R squared = 0.2456
Observed
Model
Weekly incidence
0e+00
1e−04
2e−04
3e−04
4e−04
2011
2012
2013
2014
2015
2016
Years

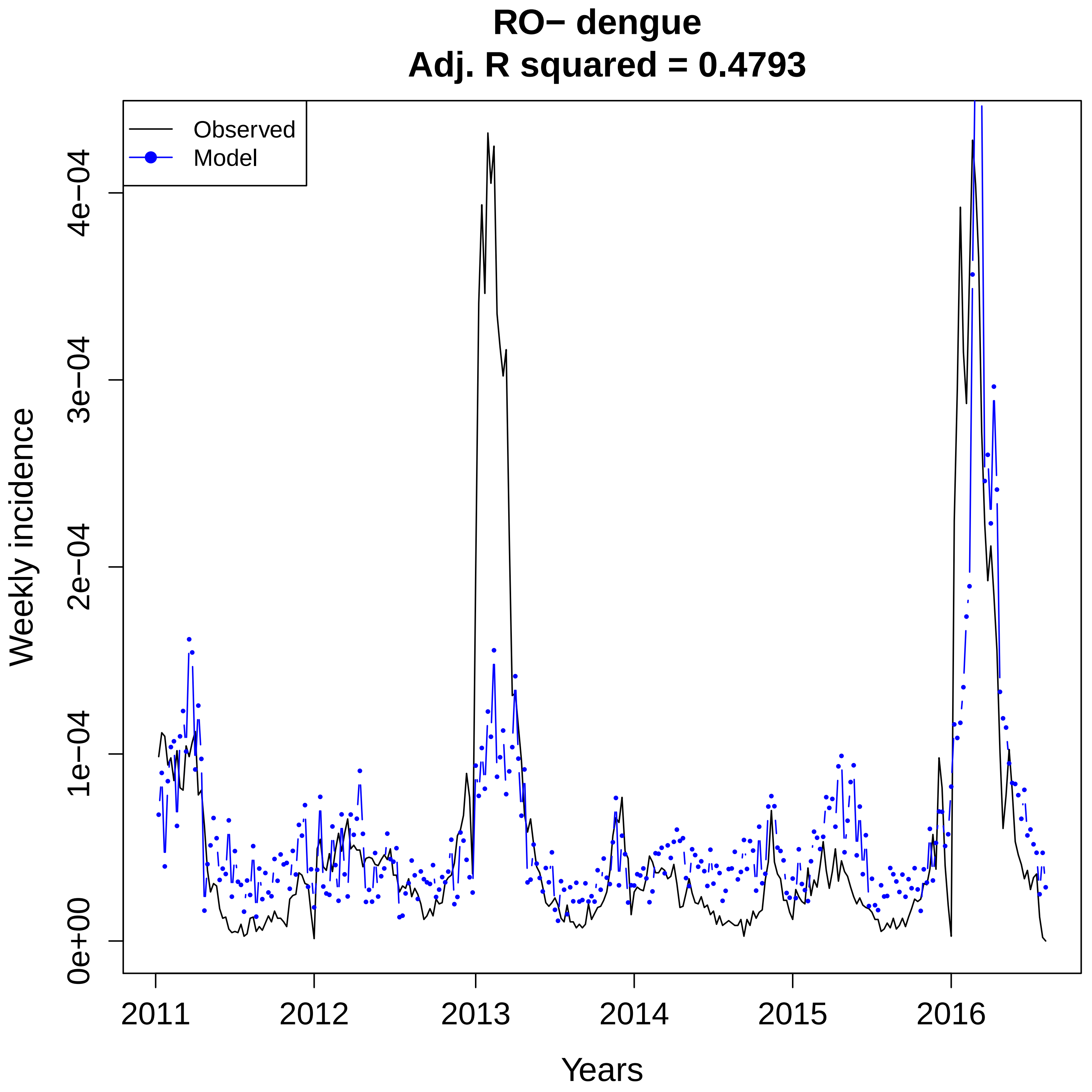
RO− dengue
Adj. R squared = 0.4793
Observed
Model
Weekly incidence
0e+00
1e−04
2e−04
3e−04
4e−04
2011
2012
2013
2014
2015
2016
Years

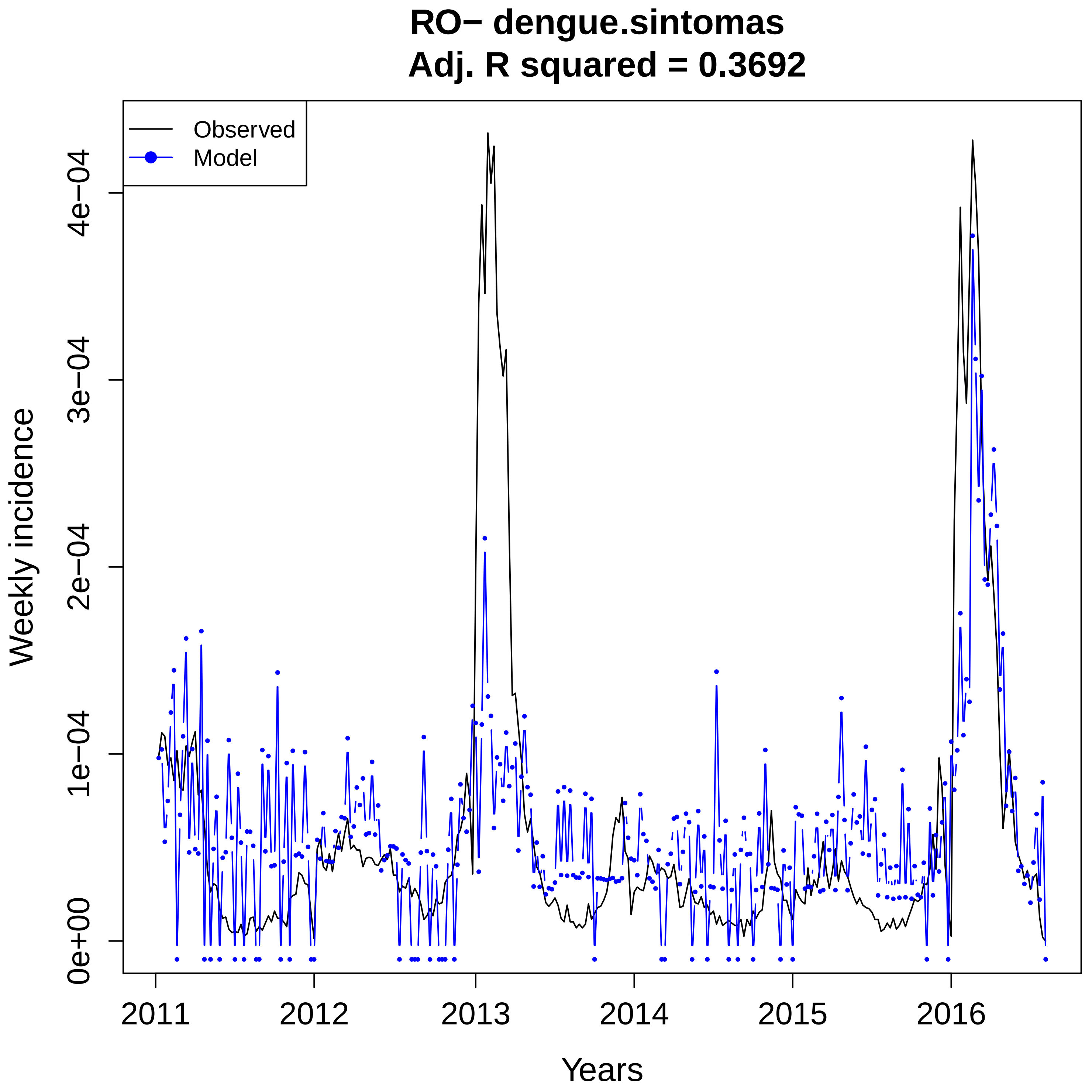
RO− dengue.sintomas
Adj. R squared = 0.3692
Observed
Model
Weekly incidence
0e+00
1e−04
2e−04
3e−04
4e−04
2011
2012
2013
2014
2015
2016
Years

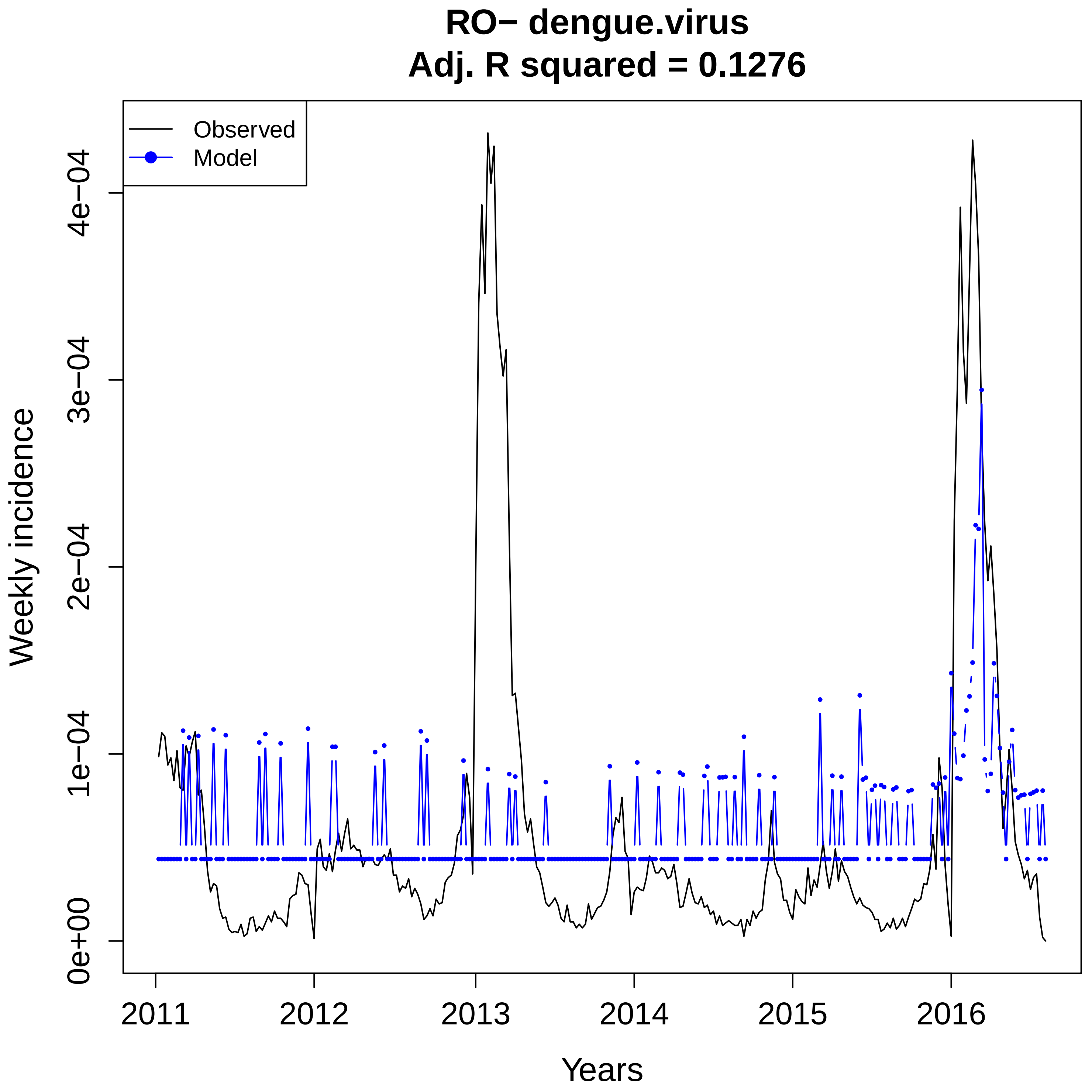

RO− dengue.virus
Adj. R squared = 0.1276
Observed
Model
Weekly incidence
0e+00
1e−04
2e−04
3e−04
4e−04
2011
2012
2013
2014
2015
2016
Years

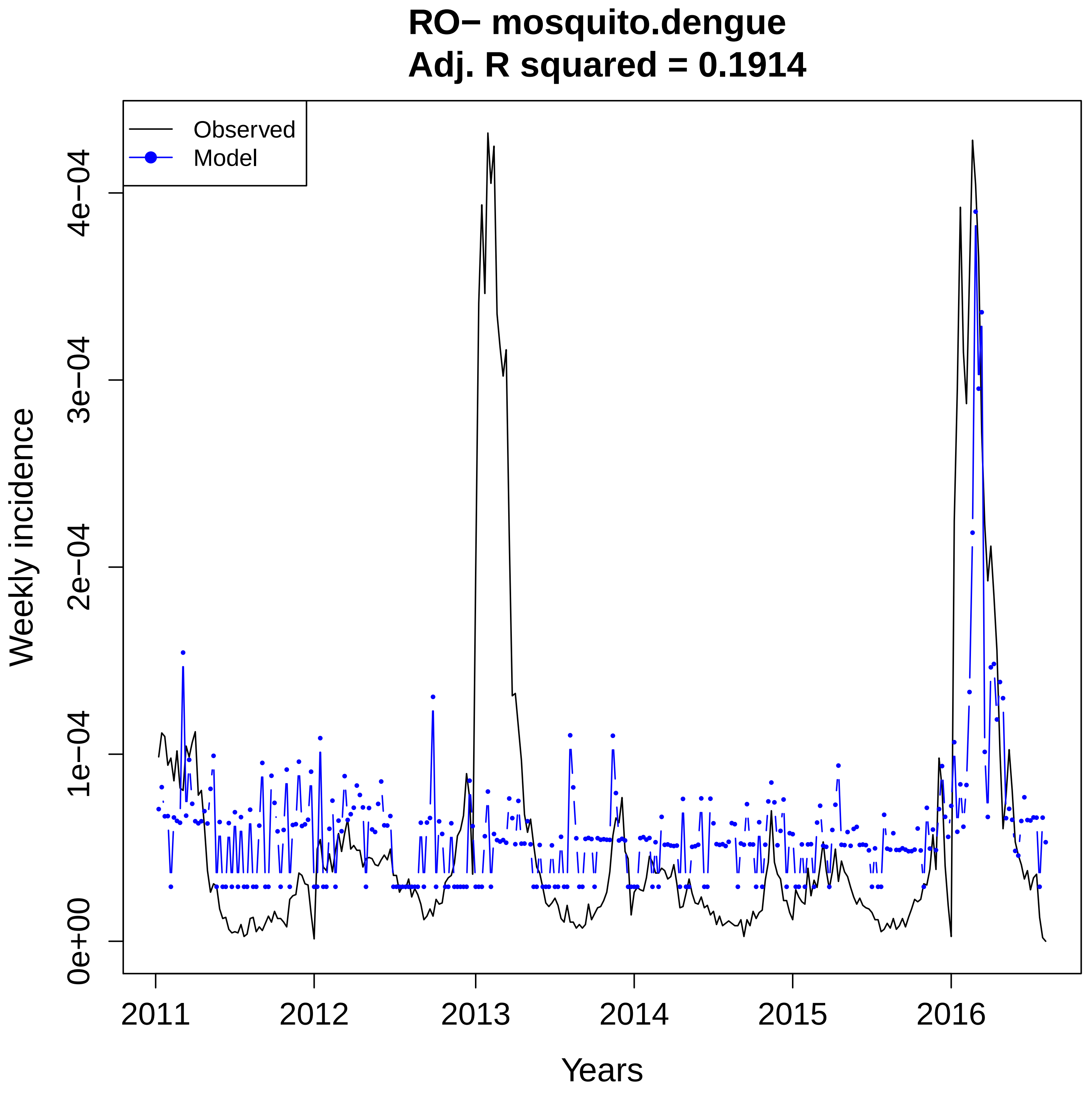

RO− mosquito.dengue
Adj. R squared = 0.1914
Observed
Model
Weekly incidence
0e+00
1e−04
2e−04
3e−04
4e−04
2011
2012
2013
2014
2015
2016
Years

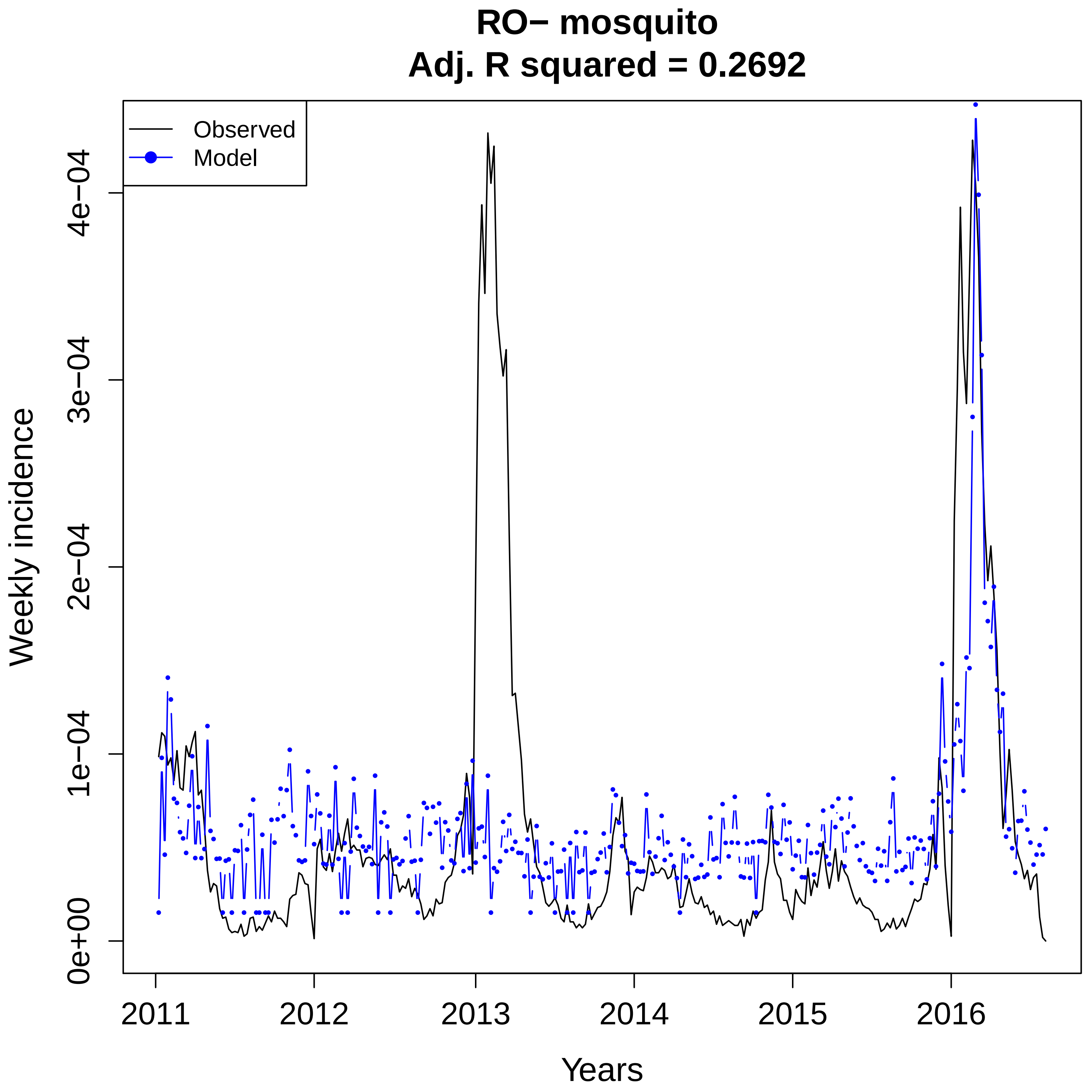
RO− mosquito
Adj. R squared = 0.2692
Observed
Model
Weekly incidence
0e+00
1e−04
2e−04
3e−04
4e−04
2011
2012
2013
2014
2015
2016
Years

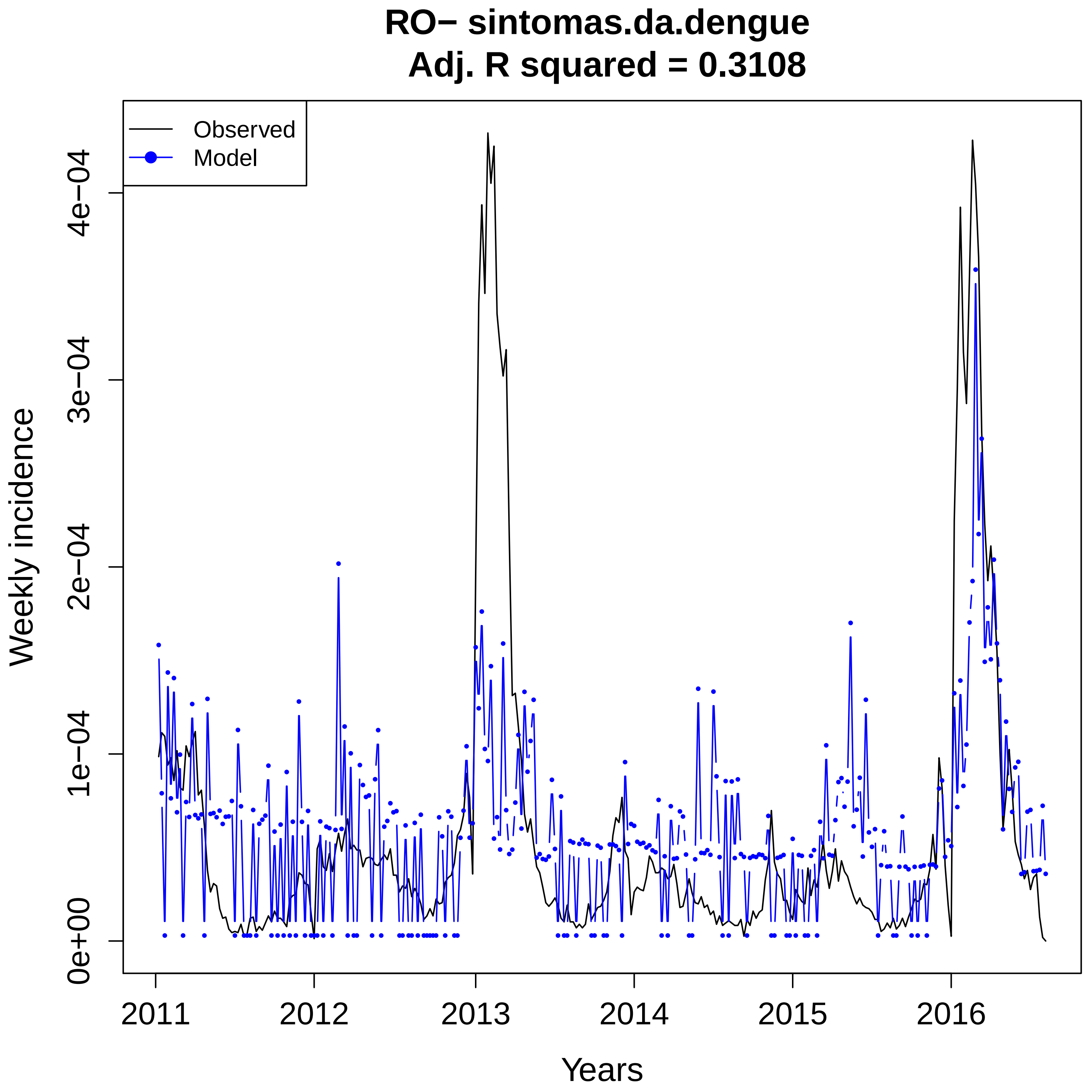
RO− sintomas.da.dengue
Adj. R squared = 0.3108
Observed
Model
Weekly incidence
0e+00
1e−04
2e−04
3e−04
4e−04
2011
2012
2013
2014
2015
2016
Years

Roraima (RR)
Dengue incidence vs. Google Health Trends (2011–2016)

Definitions:

- **Combined terms:**
  - **All:** model with all the available terms. n= number of terms
  - **Four:** model with "dengue", "dengue sintomas", "aedes", and "mosquito".
  - **Uncorrelated:** model with non-correlated terms. n = number of uncorrelated terms
- **Individual terms:** Plots developed with one term at a time.

- Terms for all and uncorrelated models can be found in additional file 3.

Combined terms
Adjusted R squared

Roraima (RR)

All
(n = 7)

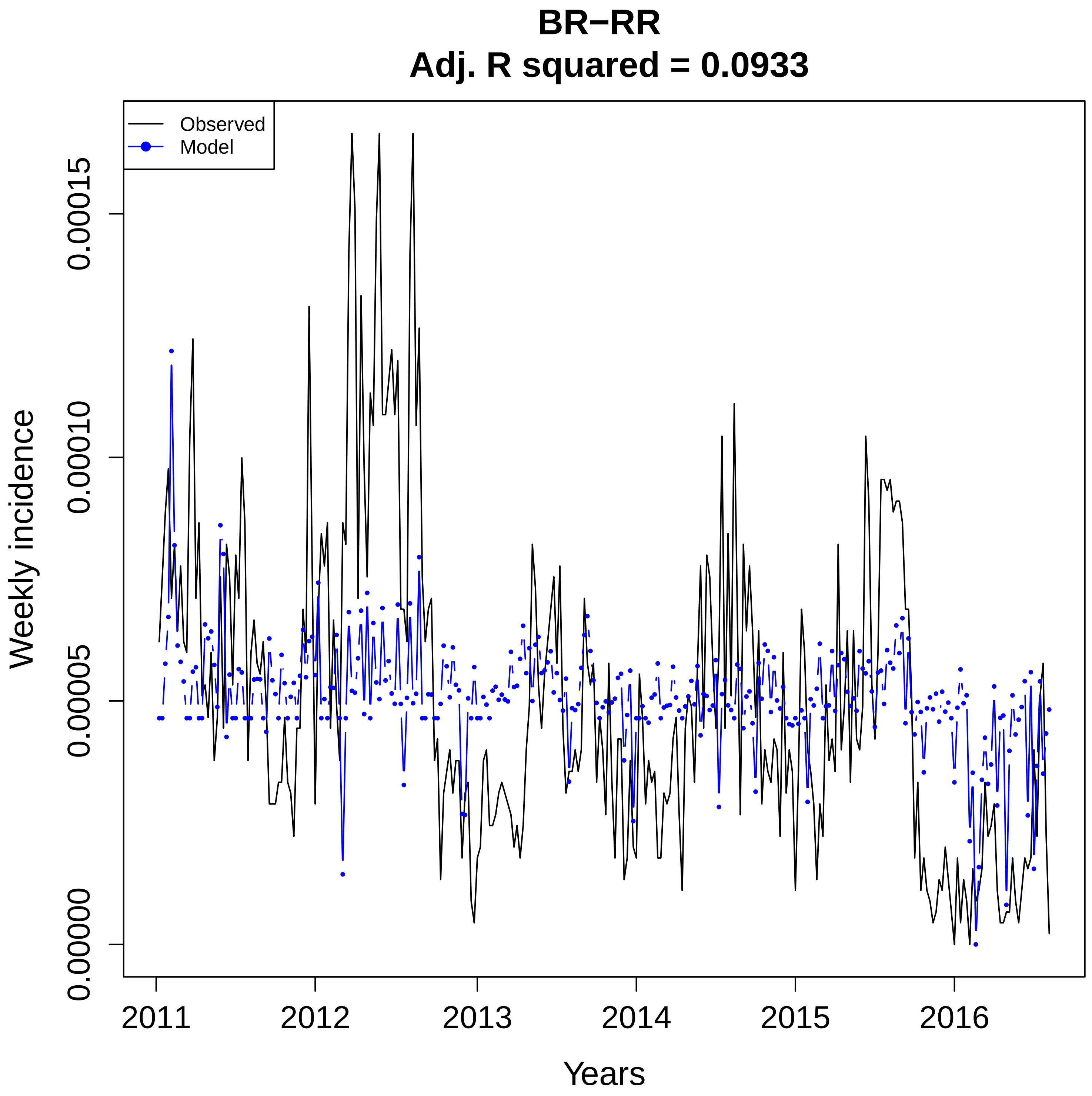
BR−RR
Adj. R squared = 0.0933
Observed
Model
Weekly incidence
0.00000
0.00005
0.00010
0.00015
2011
2012
2013
2014
2015
2016
Years

Four

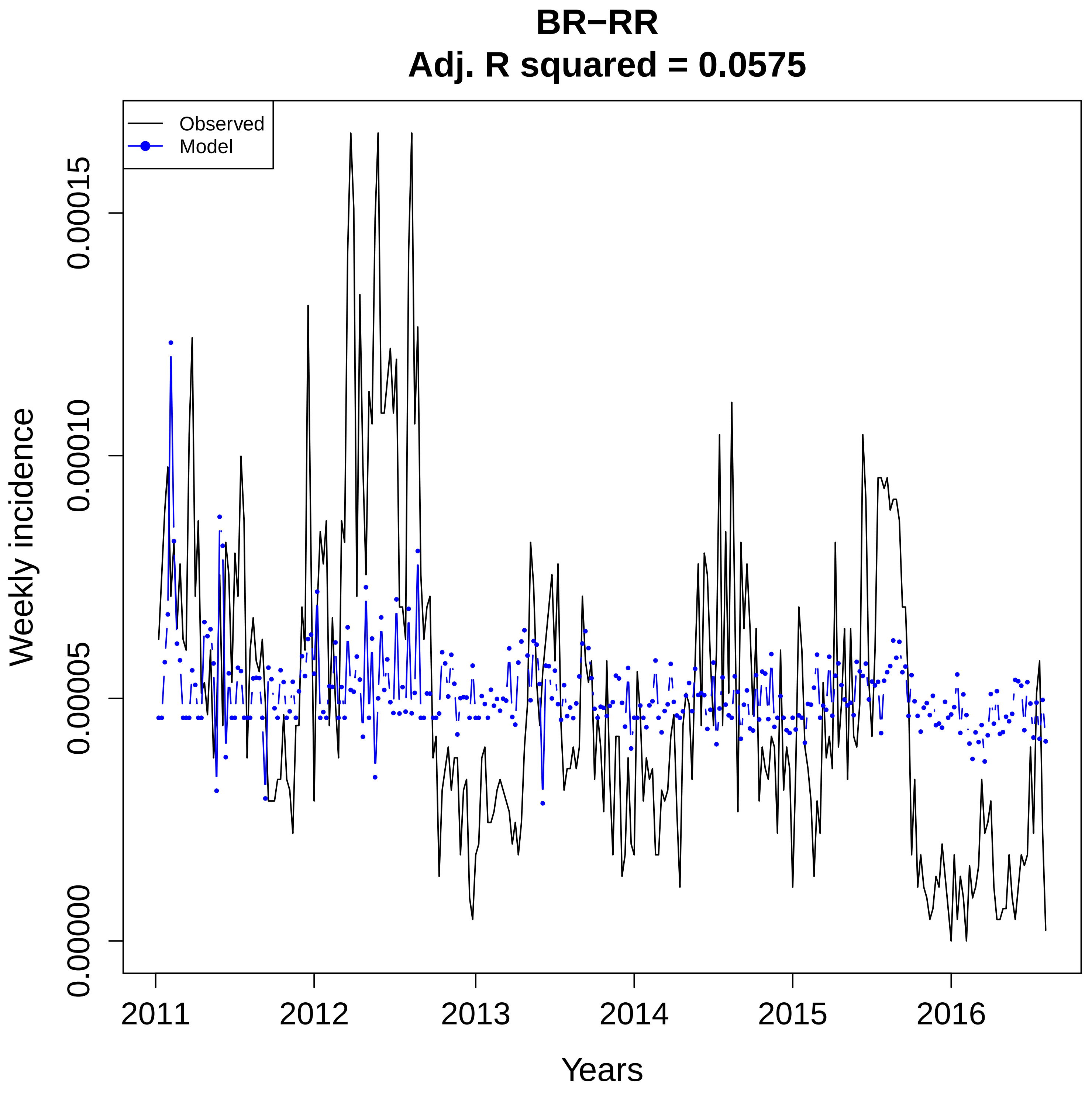

BR−RR
Adj. R squared = 0.0575
Observed
Model
Weekly incidence
0.00000
0.00005
0.00010
0.00015
2011
2012
2013
2014
2015
2016
Years

Uncorrelated
(n = 6)

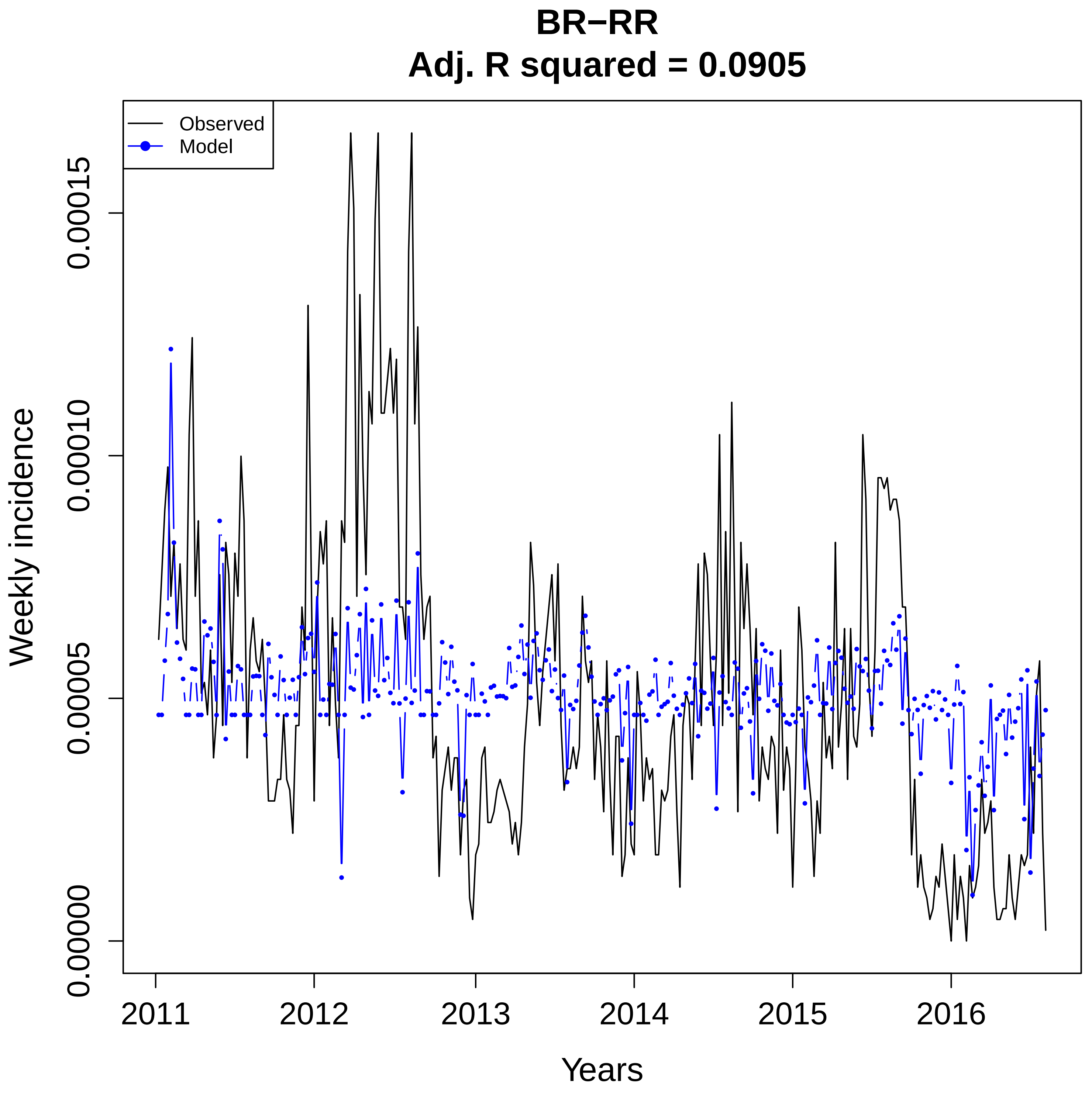

Individual terms
Adjusted R squared

Roraima (RR)

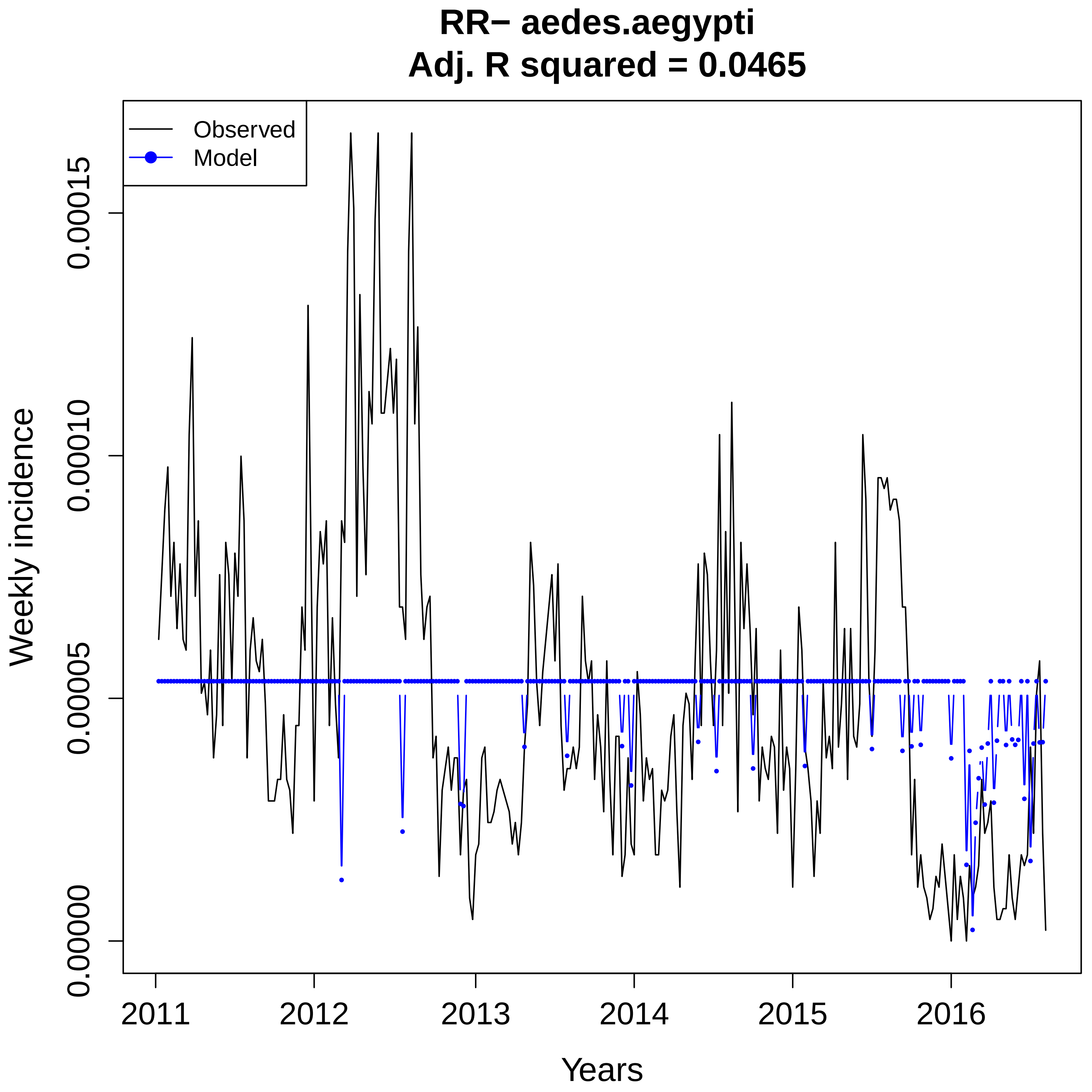
RR− aedes.aegypti
Adj. R squared = 0.0465
Observed
Model
Weekly incidence
0.00000
0.00005
0.00010
0.00015
2011
2012
2013
2014
2015
2016
Years

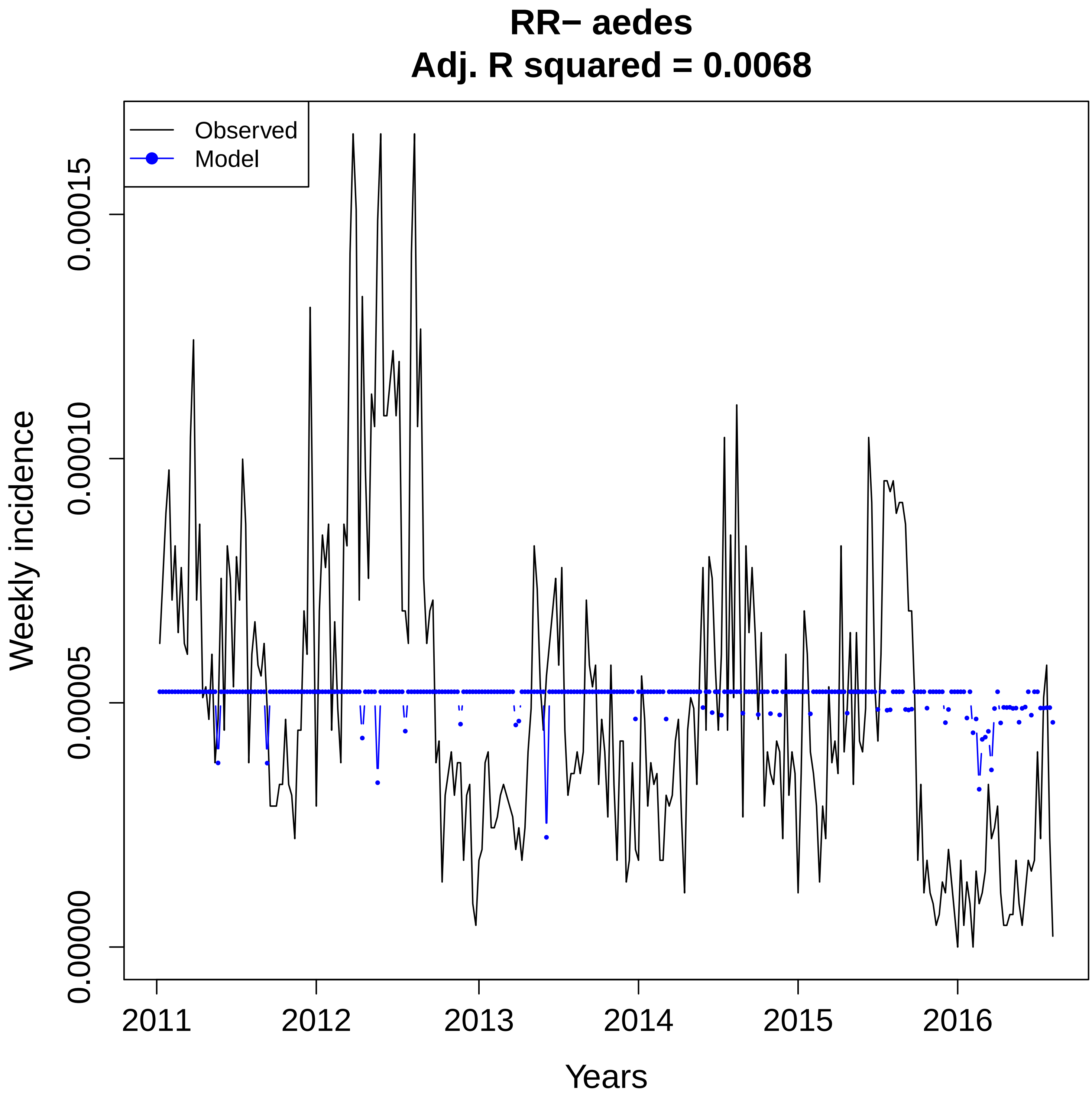

RR− aedes
Adj. R squared = 0.0068
Observed
Model
Weekly incidence
0.00000
0.00005
0.00010
0.00015
2011
2012
2013
2014
2015
2016
Years

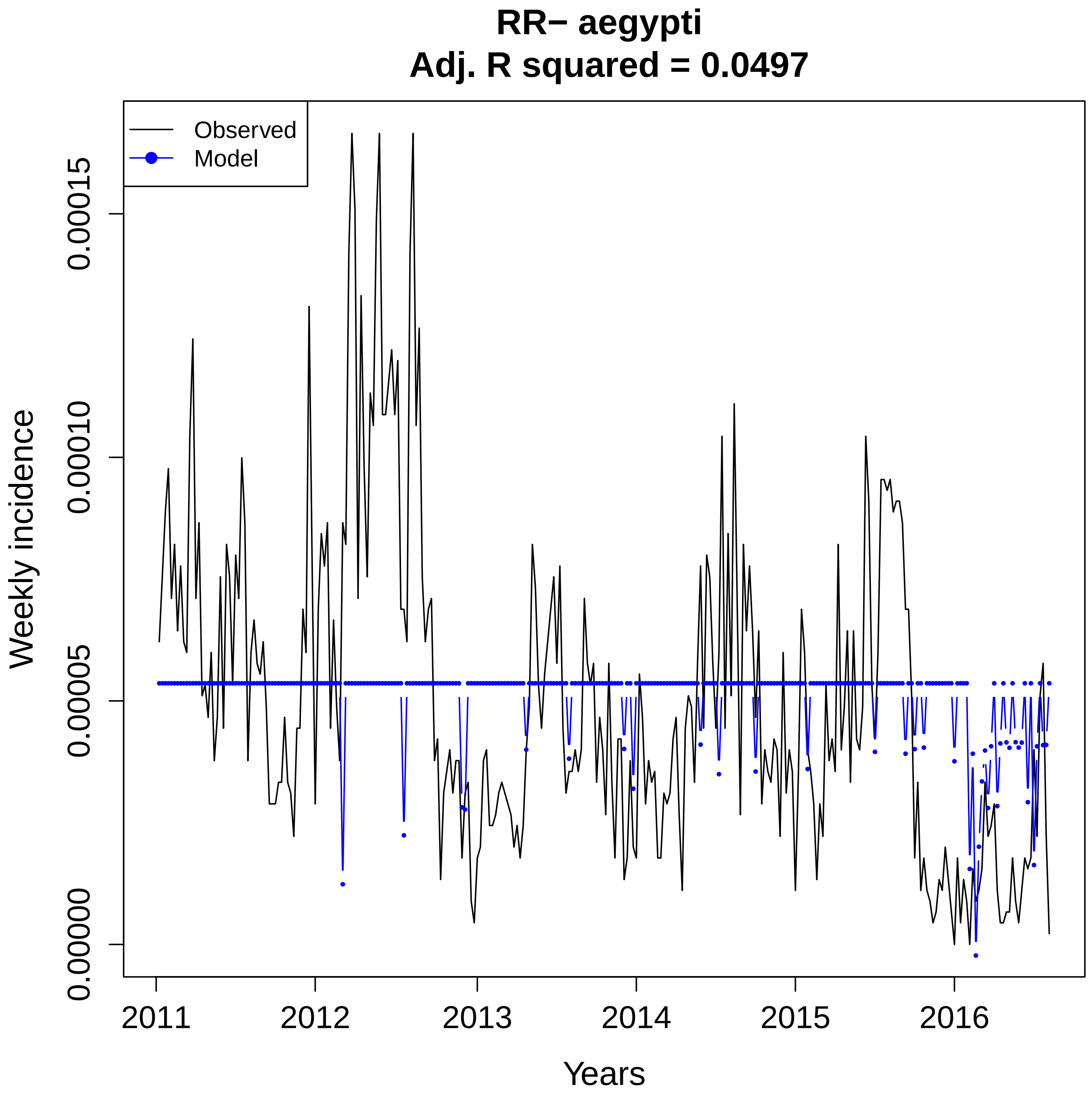
RR− aegypti
Adj. R squared = 0.0497
Observed
Model
Weekly incidence
0.00000
0.00005
0.00010
0.00015
2011
2012
2013
2014
2015
2016
Years

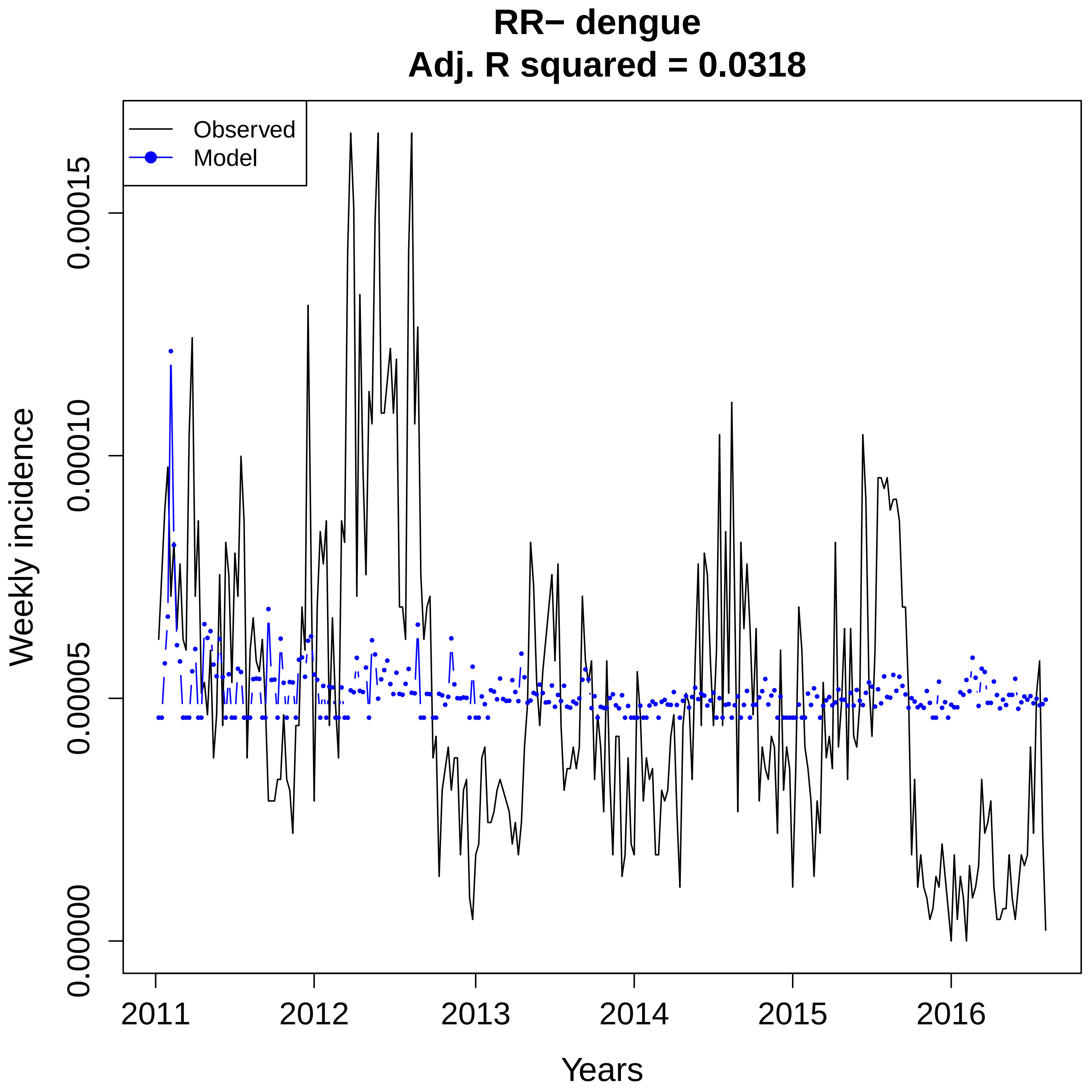

RR− dengue
Adj. R squared = 0.0318
Observed
Model
Weekly incidence
0.00000
0.00005
0.00010
0.00015
2011
2012
2013
2014
2015
2016
Years

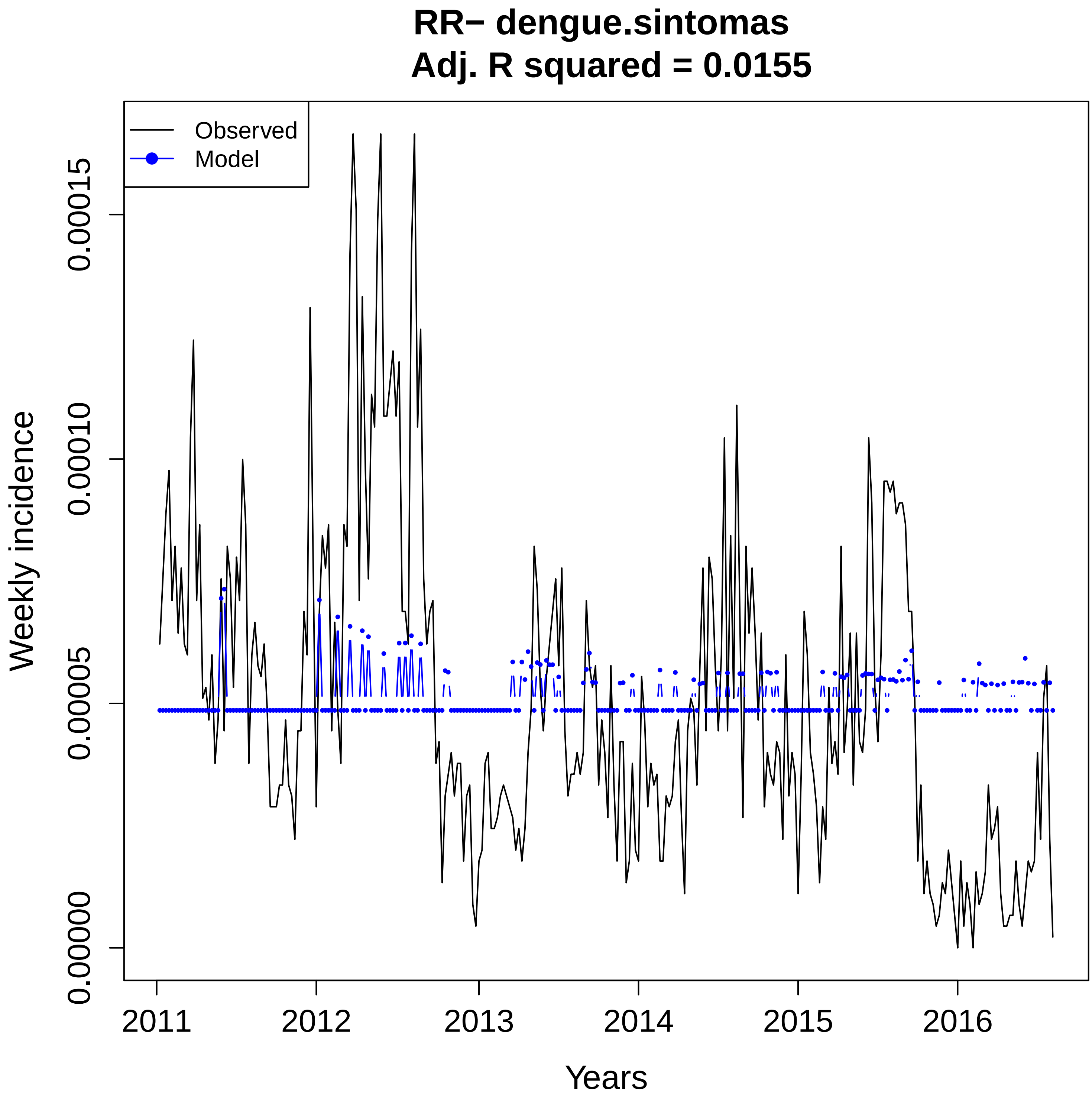

RR− dengue.sintomas
Adj. R squared = 0.0155
Observed
Model
Weekly incidence
0.00000
0.00005
0.00010
0.00015
2011
2012
2013
2014
2015
2016
Years

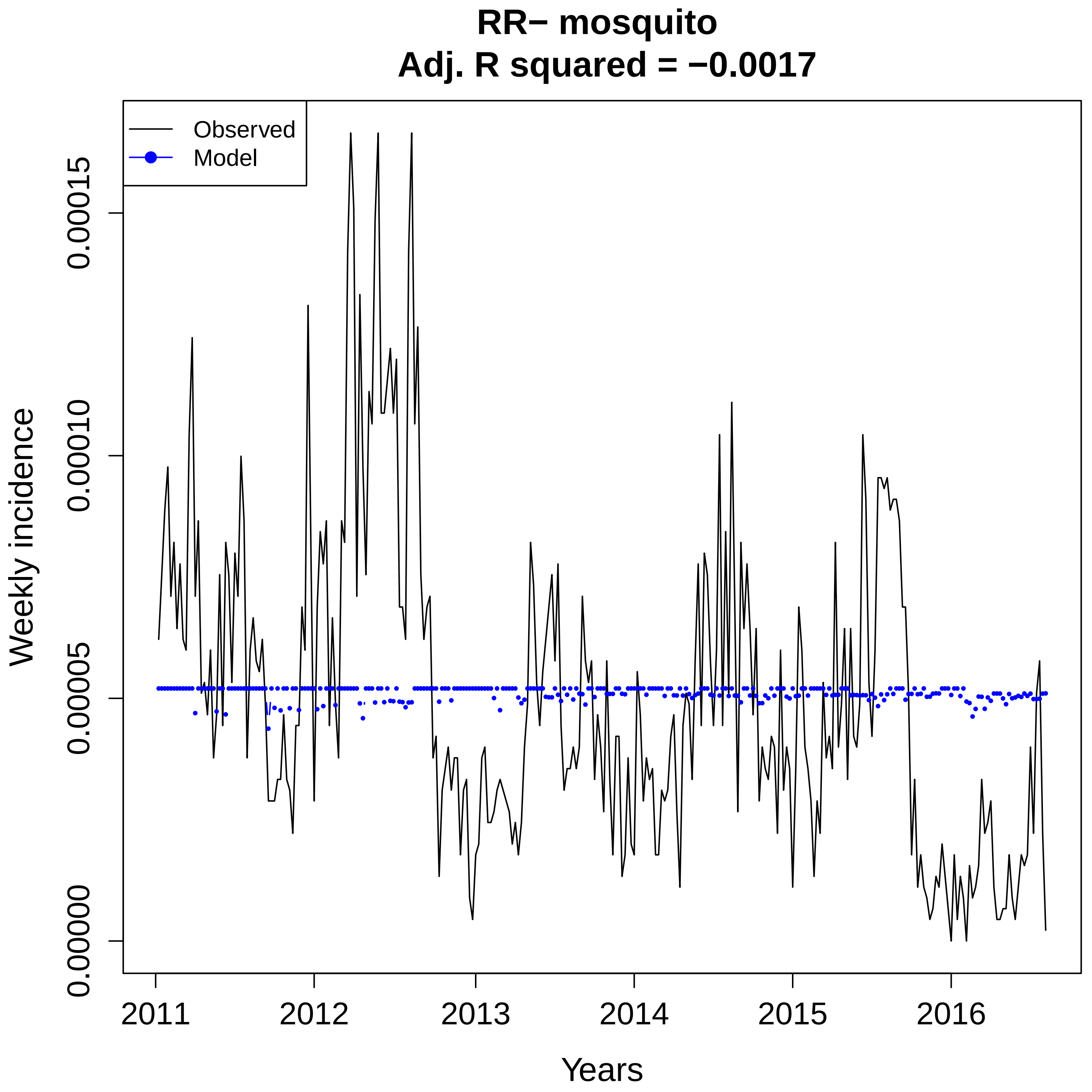

RR− mosquito
Adj. R squared = −0.0017
Observed
Model
Weekly incidence
0.00000
0.00005
0.00010
0.00015
2011
2012
2013
2014
2015
2016
Years

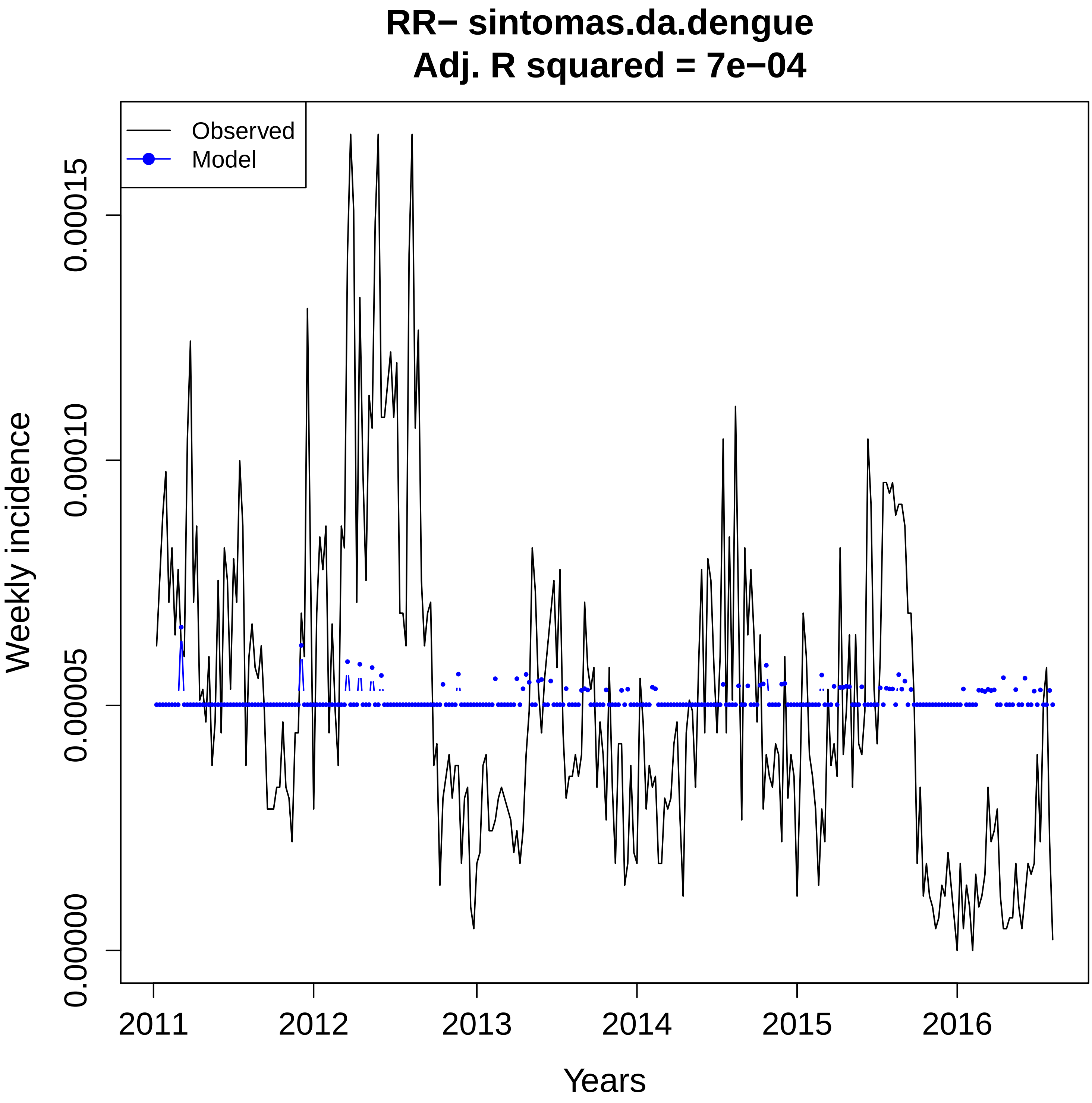
RR− sintomas.da.dengue
Adj. R squared = 7e−04
Observed
Model
Weekly incidence
0.00000
0.00005
0.00010
0.00015
2011
2012
2013
2014
2015
2016
Years

Rio Grande do Sul (RS)
Dengue incidence vs. Google Health Trends (2011–2016)

<u>Definitions:</u>

- **Combined terms:**
  - **All:** model with all the available terms. n= number of terms
  - **Four:** model with "dengue", "dengue sintomas", "aedes", and "mosquito".
  - **Uncorrelated:** model with non-correlated terms. n = number of uncorrelated terms
- **Individual terms:** Plots developed with one term at a time.

- Terms for all and uncorrelated models can be found in additional file 3.

Combined terms
Adjusted R squared

Rio Grande do Sul (RS)

All
(n = 12)

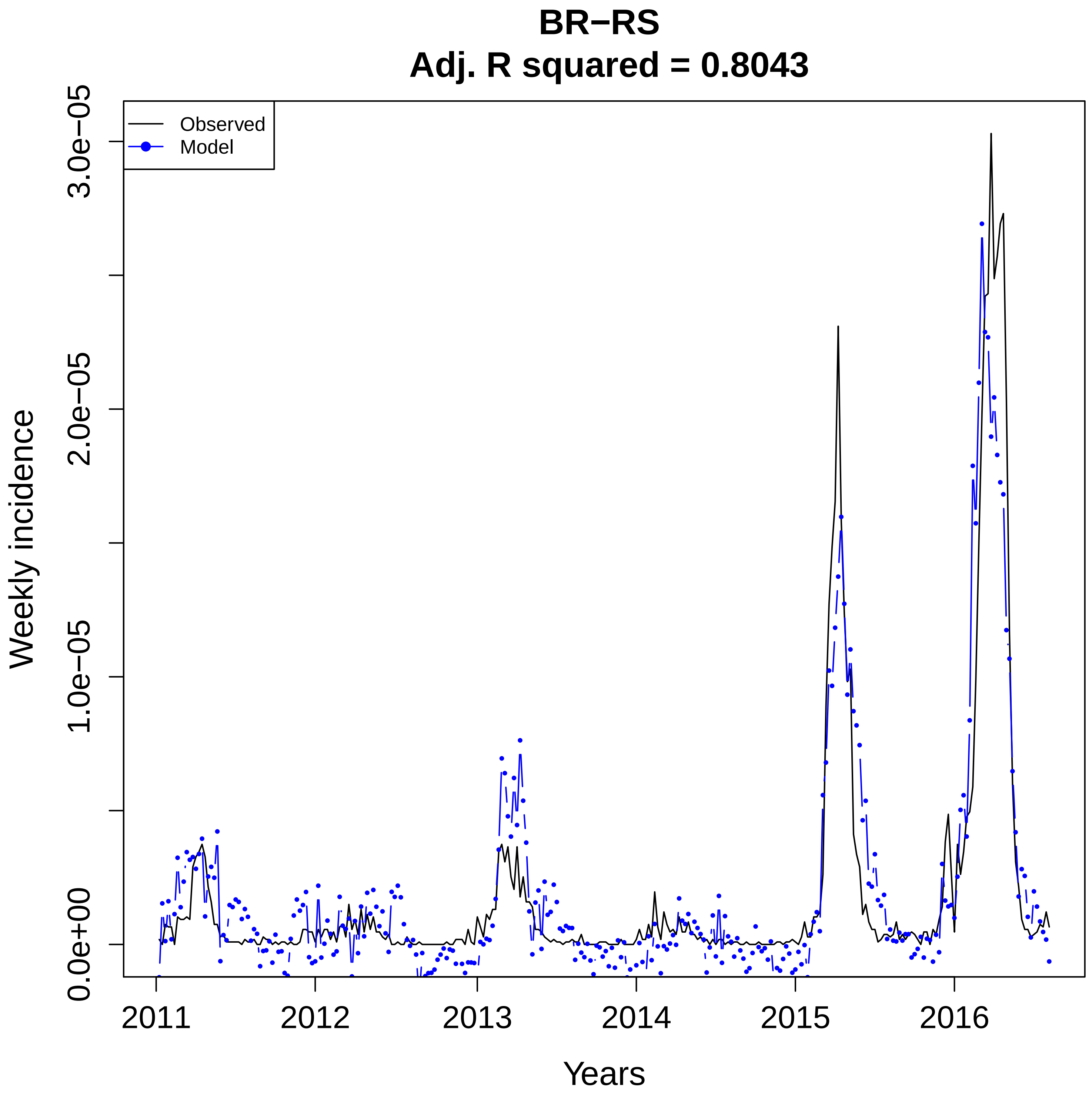

BR−RS
Adj. R squared = 0.8043
Observed
Model
Weekly incidence
0.0e+00
1.0e−05
2.0e−05
3.0e−05
2011
2012
2013
2014
2015
2016
Years

Four

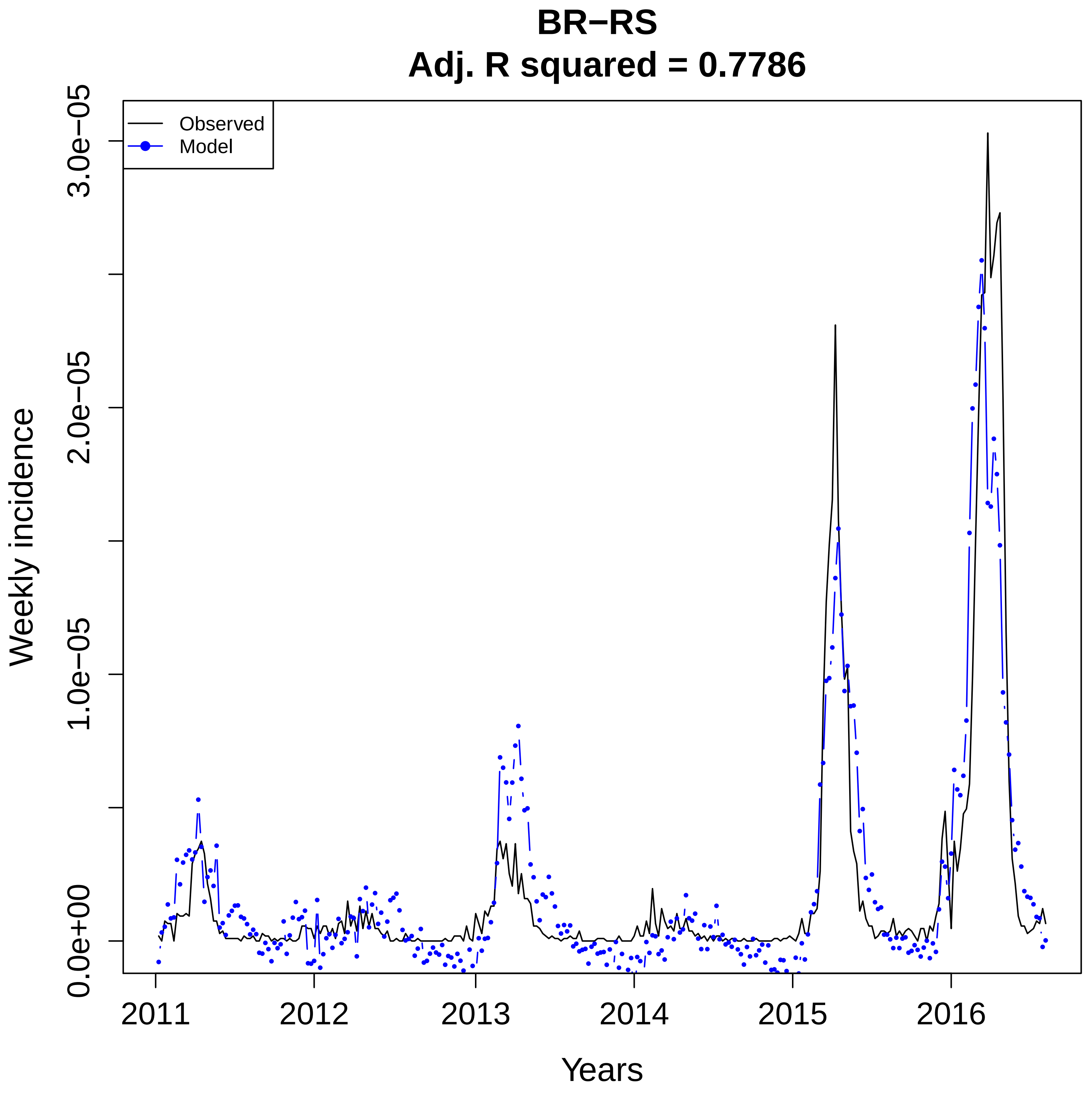
BR−RS
Adj. R squared = 0.7786
Observed
Model
Weekly incidence
0.0e+00
1.0e−05
2.0e−05
3.0e−05
2011
2012
2013
2014
2015
2016
Years

Uncorrelated
(n = 4)

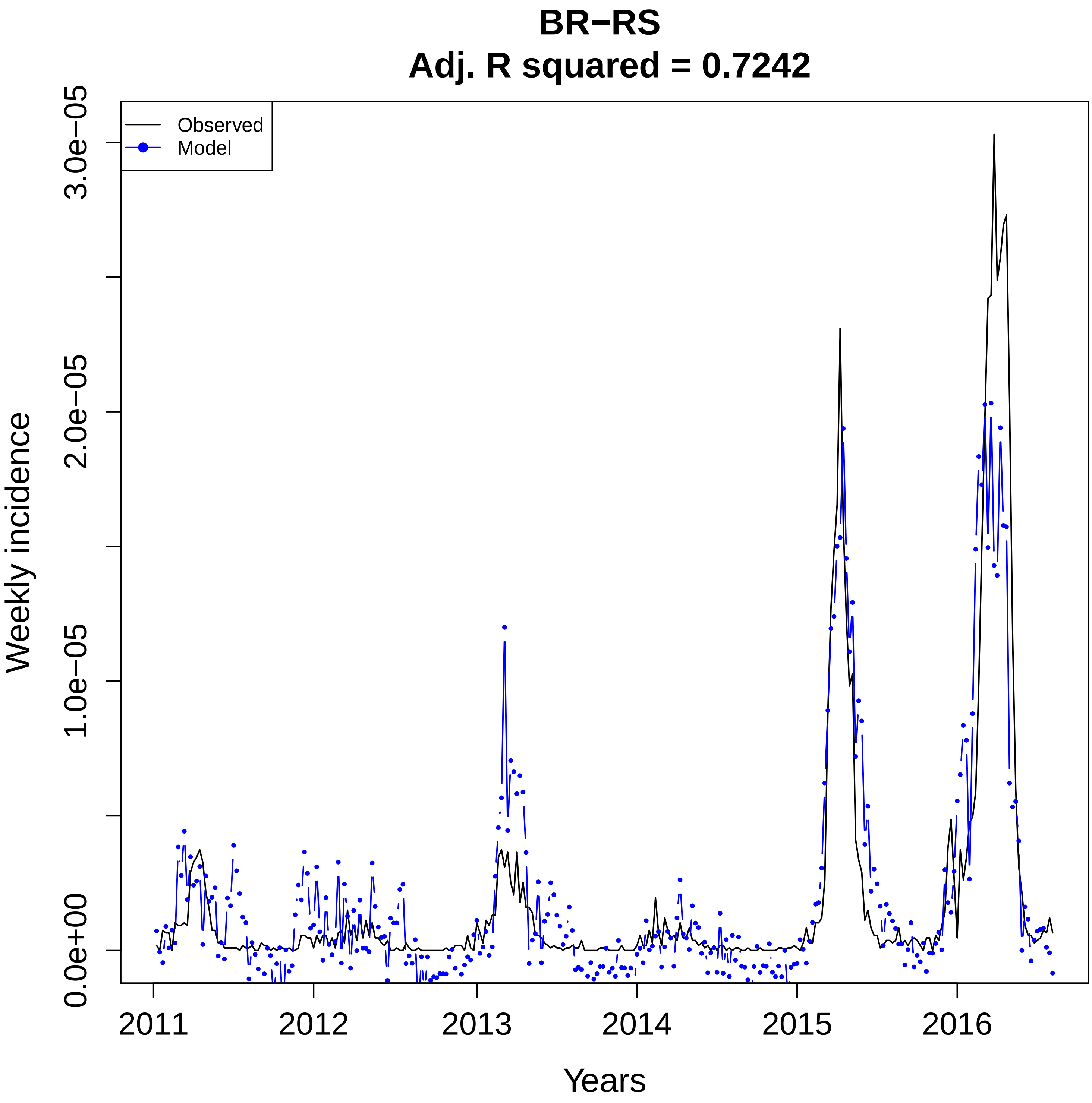

Individual terms
Adjusted R squared

Rio Grande do Sul
(RS)

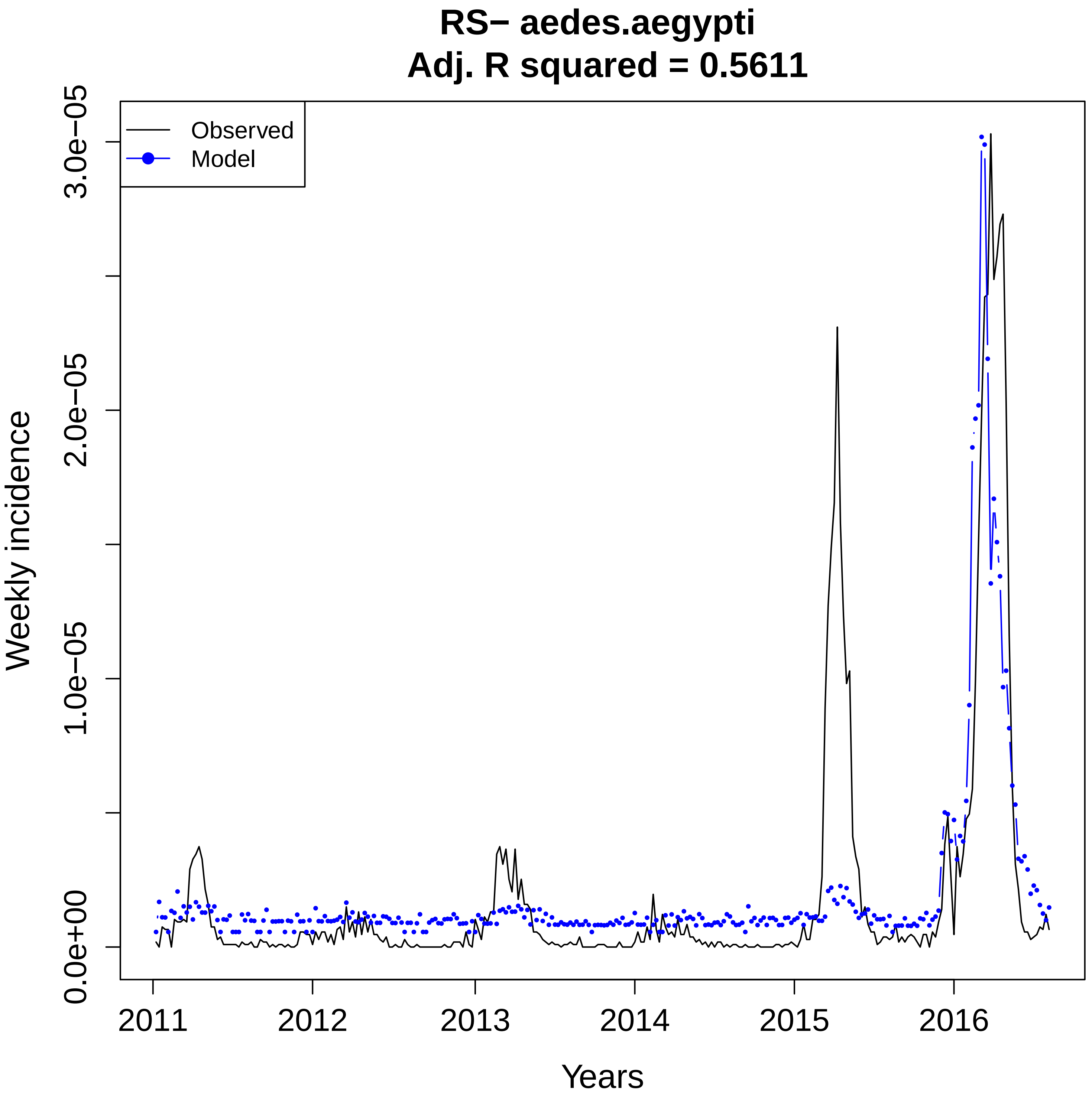
RS− aedes.aegypti
Adj. R squared = 0.5611
Observed
Model
Weekly incidence
3.0e−05
2.0e−05
1.0e−05
0.0e+00
2011
2012
2013
2014
2015
2016
Years

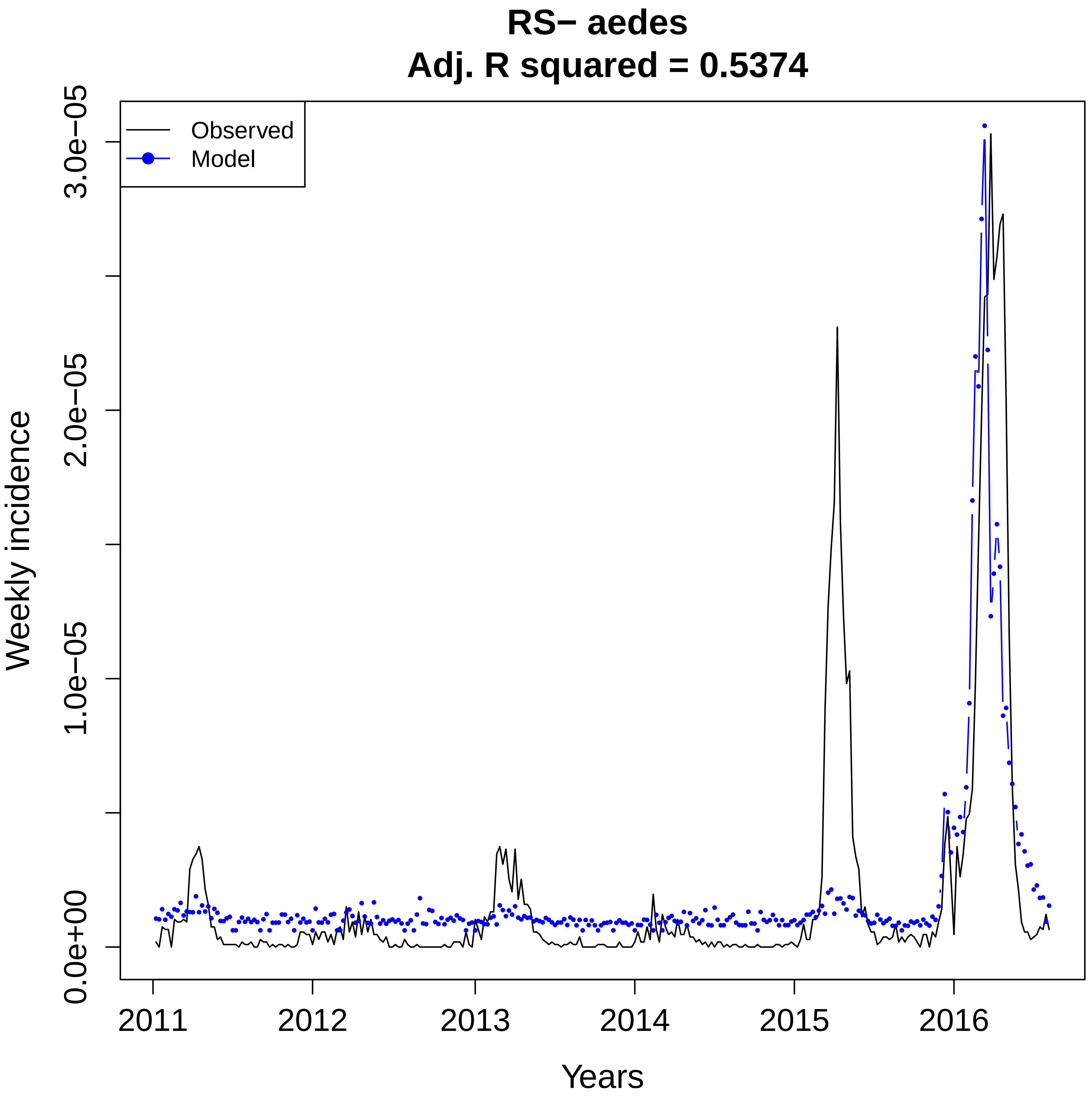

RS− aedes
Adj. R squared = 0.5374
Observed
Model
Weekly incidence
0.0e+00
1.0e−05
2.0e−05
3.0e−05
2011
2012
2013
2014
2015
2016
Years

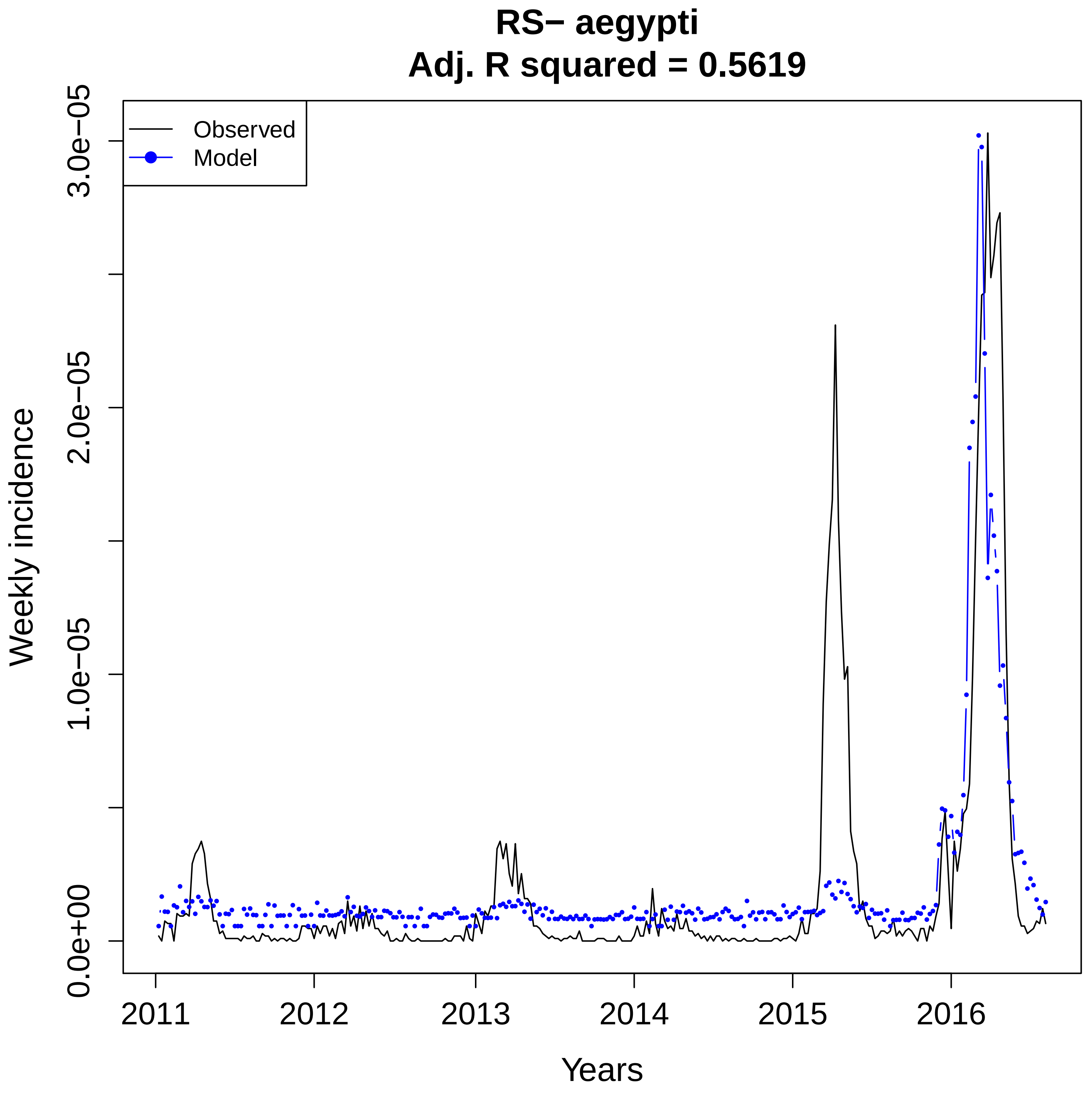
RS− aegypti
Adj. R squared = 0.5619
Observed
Model
Weekly incidence
0.0e+00
1.0e−05
2.0e−05
3.0e−05
2011
2012
2013
2014
2015
2016
Years

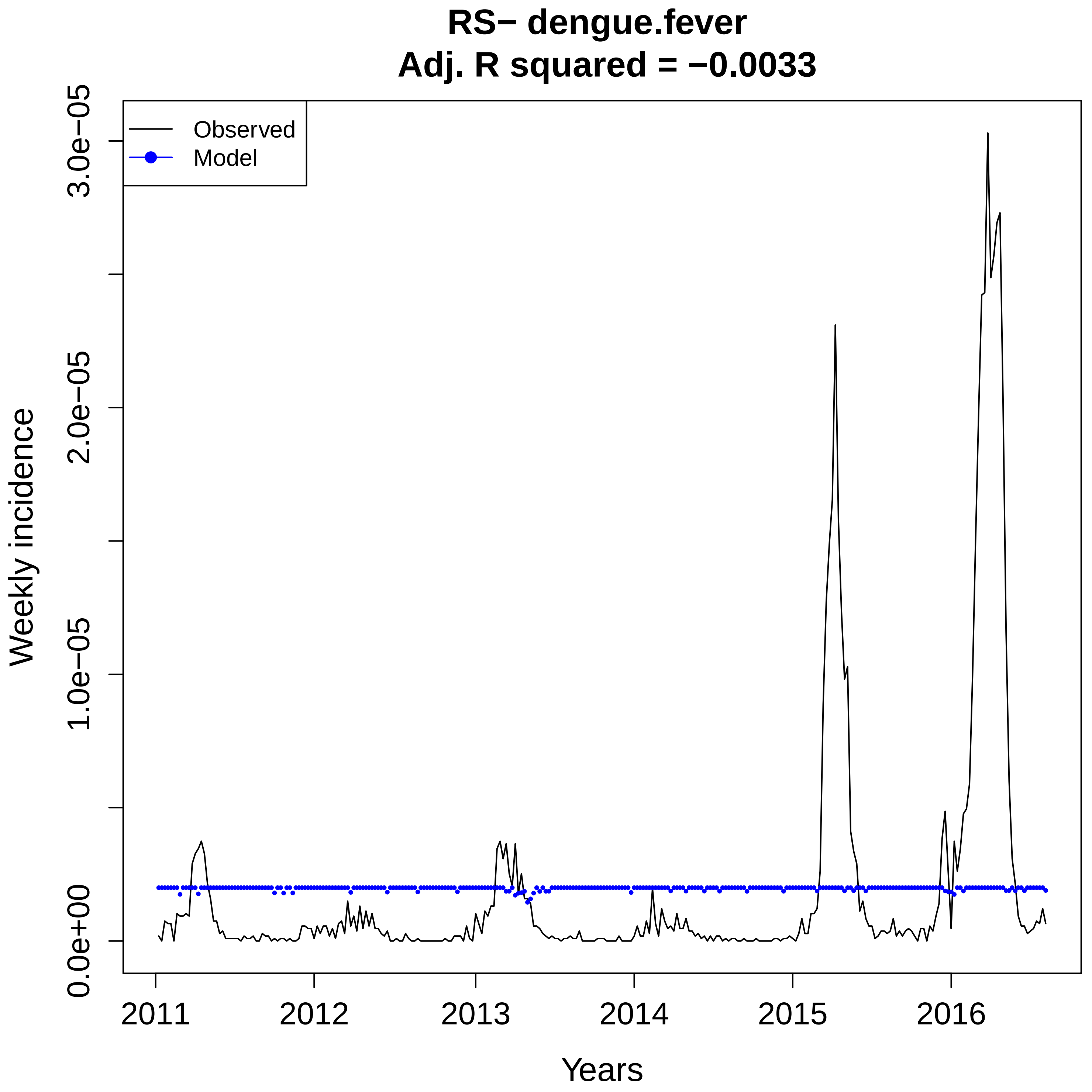

RS− dengue.fever
Adj. R squared = −0.0033
Observed
Model
Weekly incidence
0.0e+00
1.0e−05
2.0e−05
3.0e−05
2011
2012
2013
2014
2015
2016
Years

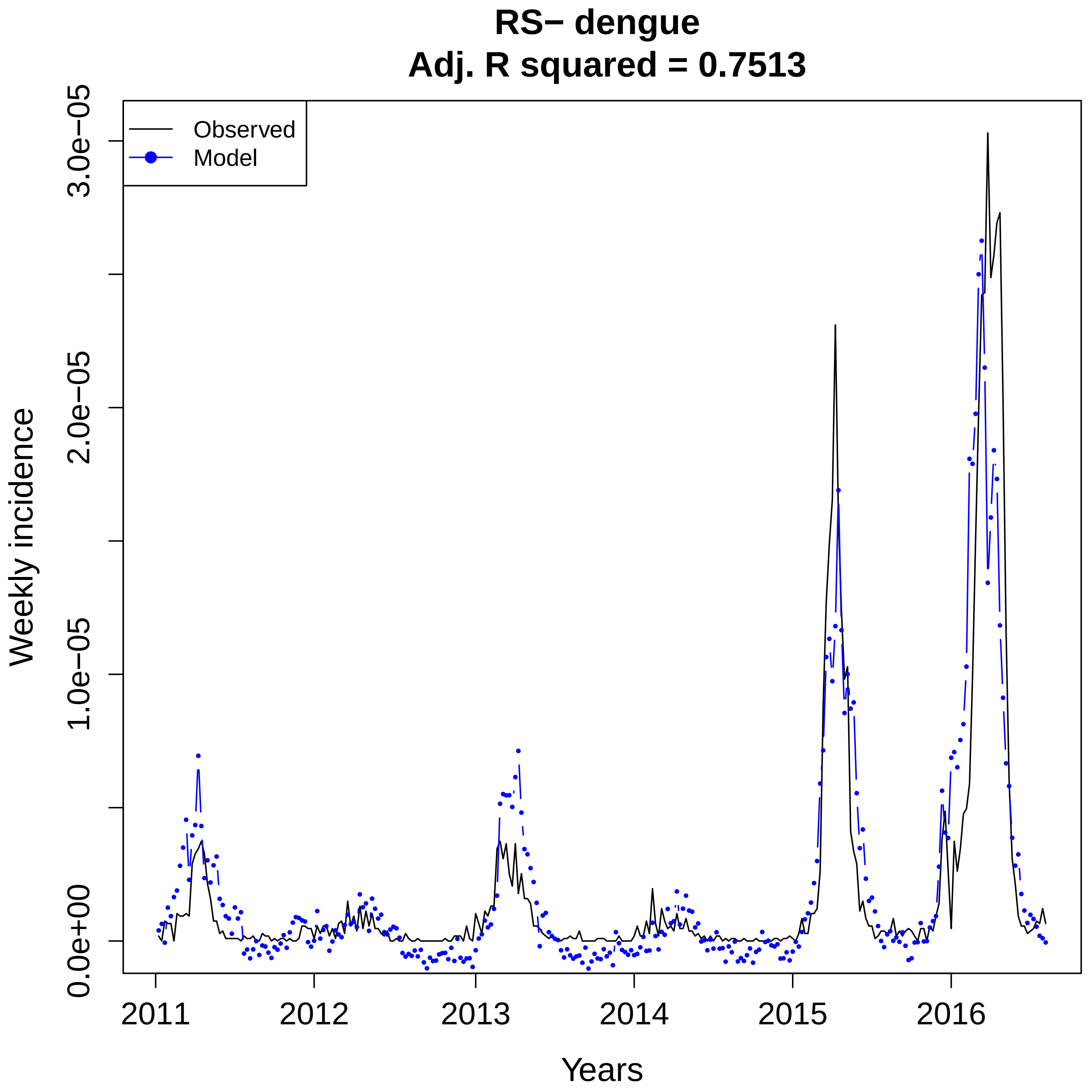

RS− dengue
Adj. R squared = 0.7513
Observed
Model
Weekly incidence
0.0e+00
1.0e−05
2.0e−05
3.0e−05
2011
2012
2013
2014
2015
2016
Years

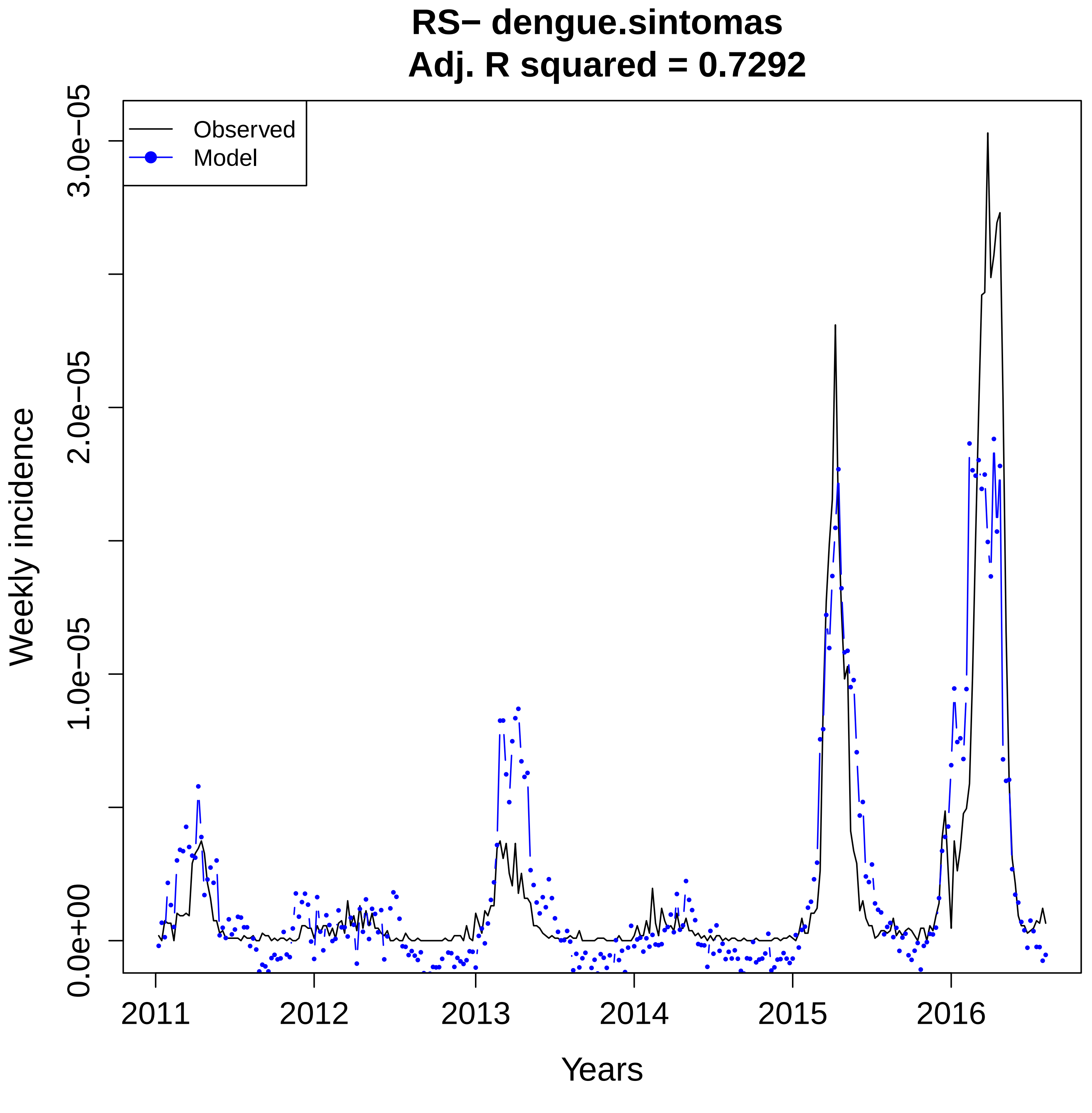

RS− dengue.sintomas
Adj. R squared = 0.7292
Observed
Model
Weekly incidence
0.0e+00
1.0e−05
2.0e−05
3.0e−05
2011
2012
2013
2014
2015
2016
Years

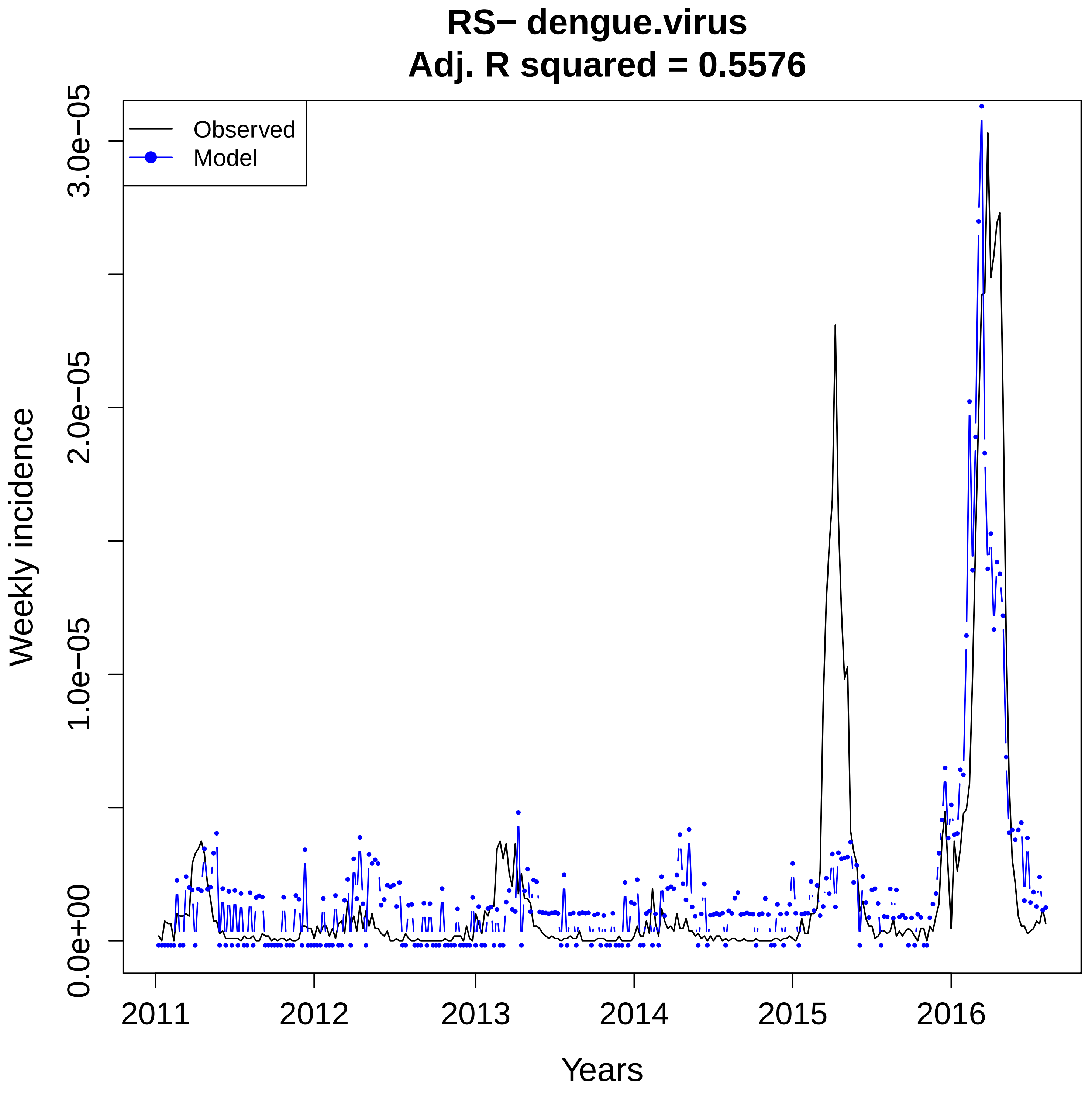
RS− dengue.virus
Adj. R squared = 0.5576
Observed
Model
Weekly incidence
0.0e+00
1.0e−05
2.0e−05
3.0e−05
2011
2012
2013
2014
2015
2016
Years

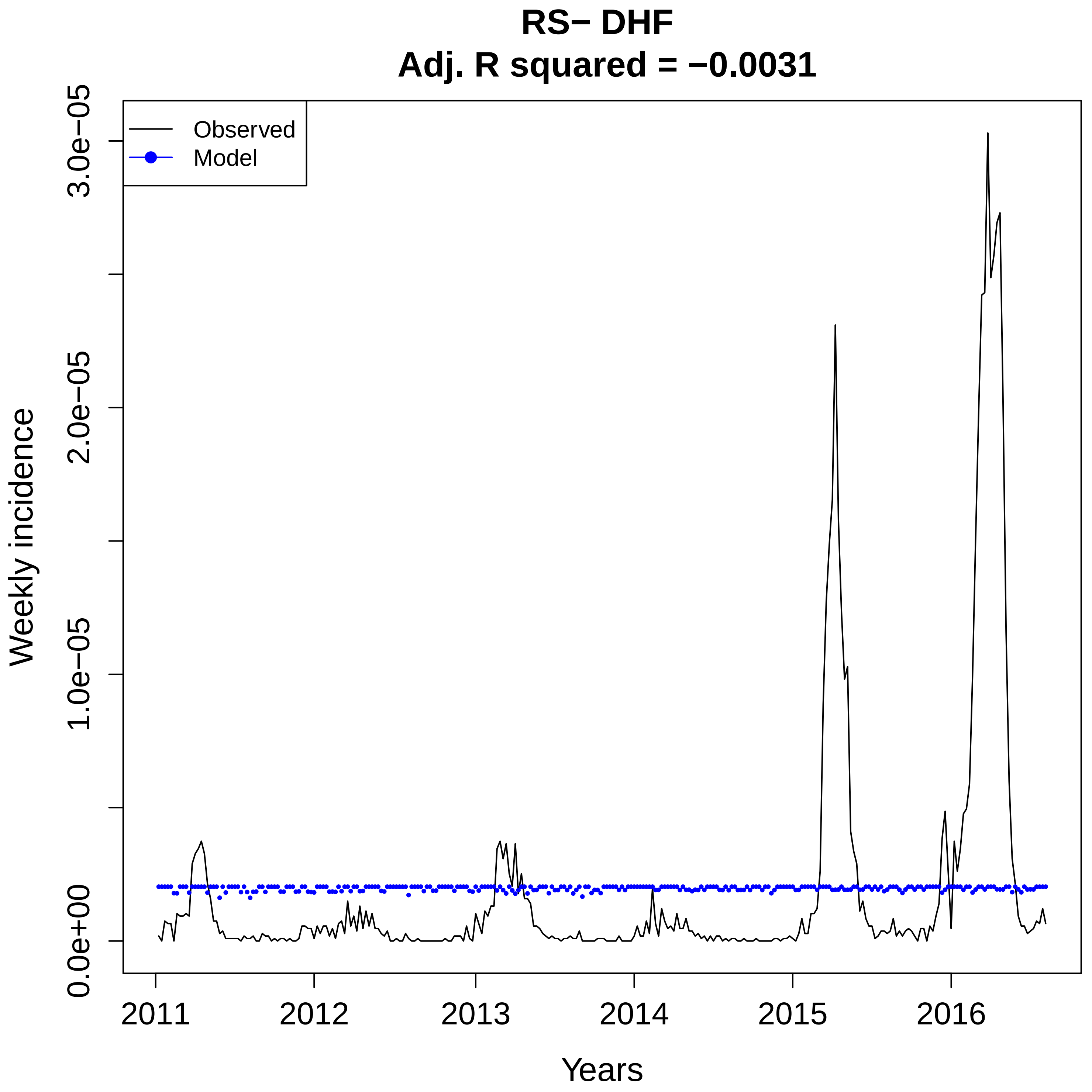
RS− DHF
Adj. R squared = −0.0031
Observed
Model
Weekly incidence
0.0e+00
1.0e−05
2.0e−05
3.0e−05
2011
2012
2013
2014
2015
2016
Years

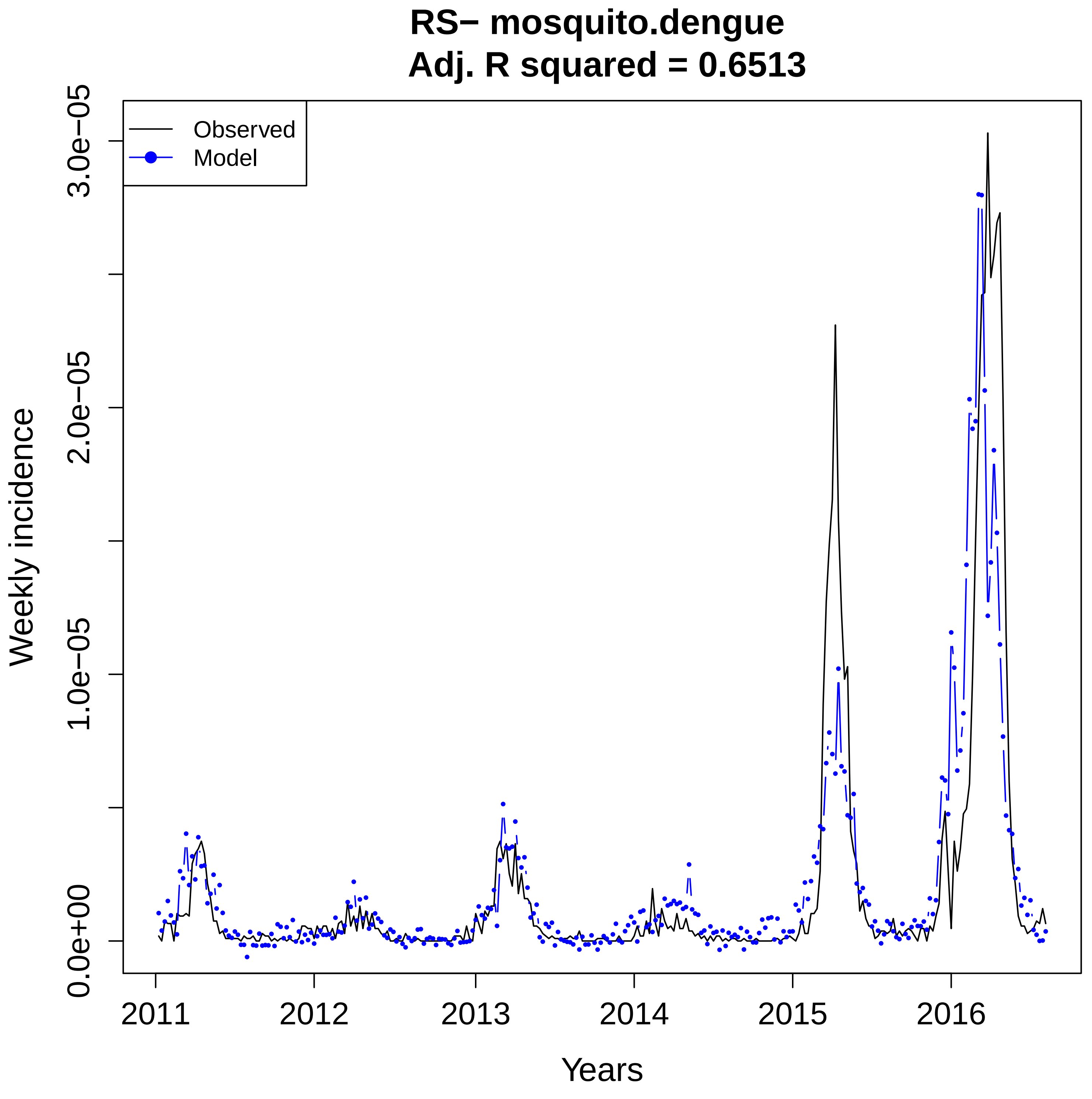
RS− mosquito.dengue
Adj. R squared = 0.6513
Observed
Model
Weekly incidence
0.0e+00
1.0e−05
2.0e−05
3.0e−05
2011
2012
2013
2014
2015
2016
Years

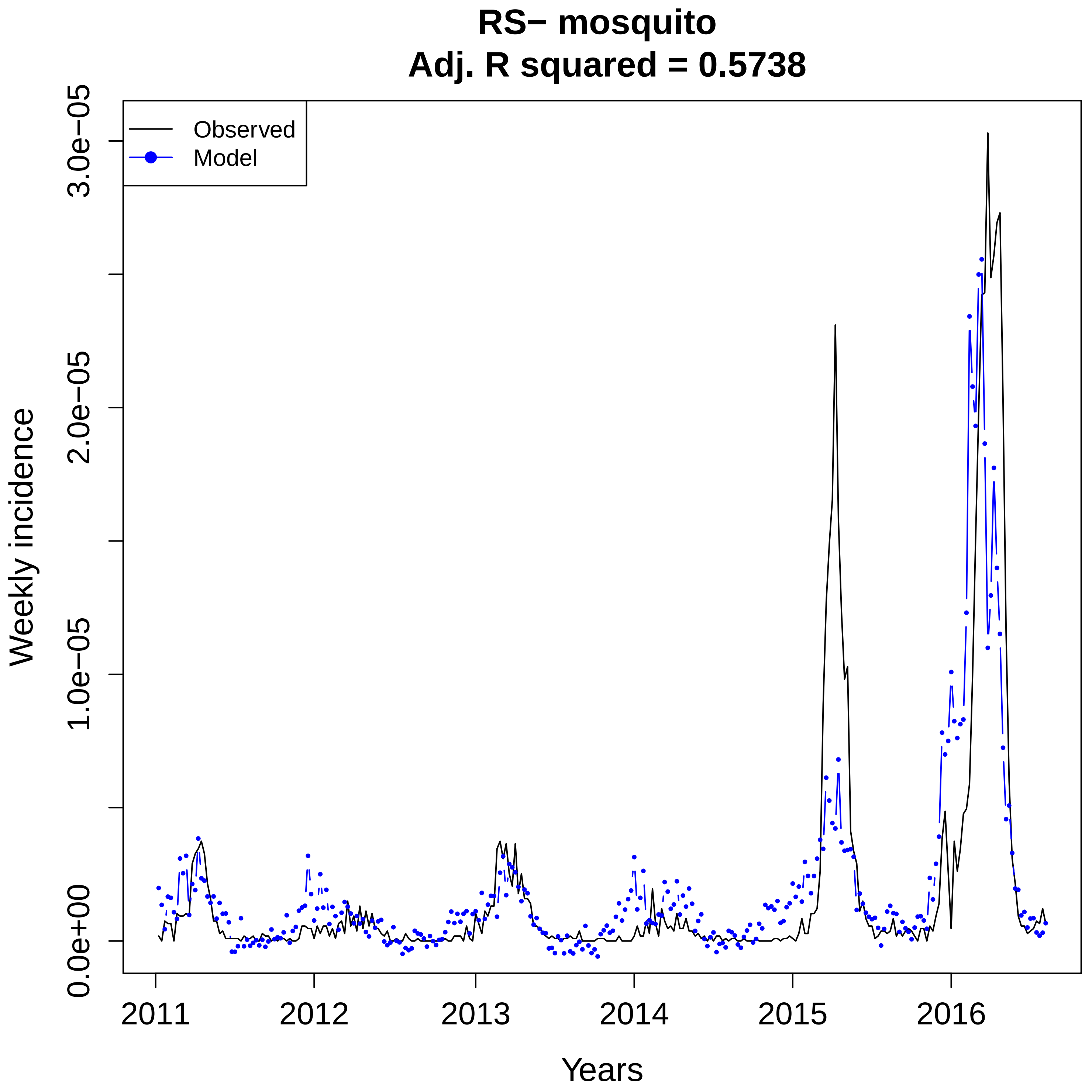
RS− mosquito
Adj. R squared = 0.5738
Observed
Model
Weekly incidence
0.0e+00
1.0e−05
2.0e−05
3.0e−05
2011
2012
2013
2014
2015
2016
Years

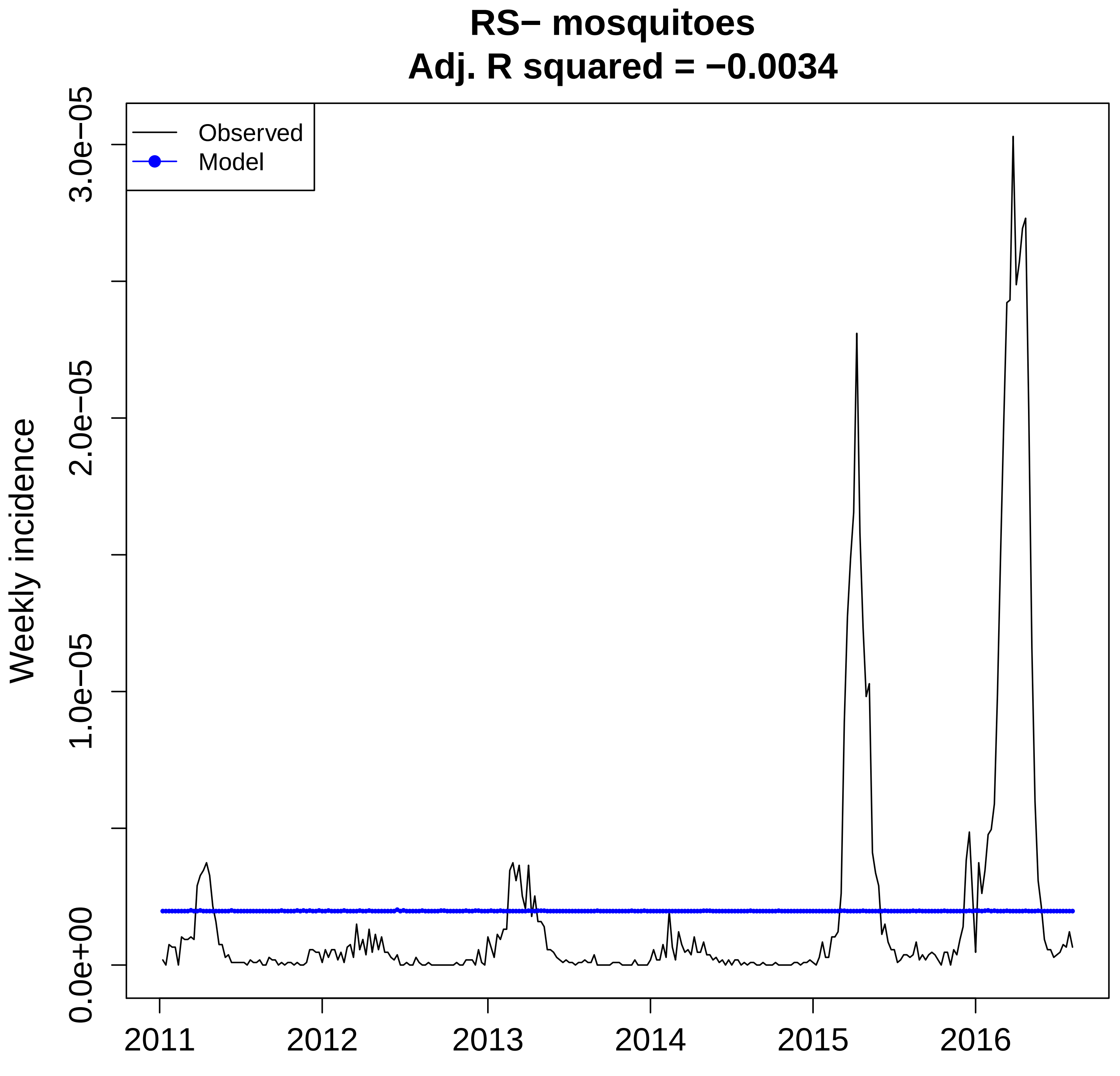
RS− mosquitoes
Adj. R squared = −0.0034
Observed
Model
Weekly incidence
0.0e+00
1.0e−05
2.0e−05
3.0e−05
2011
2012
2013
2014
2015
2016
Years

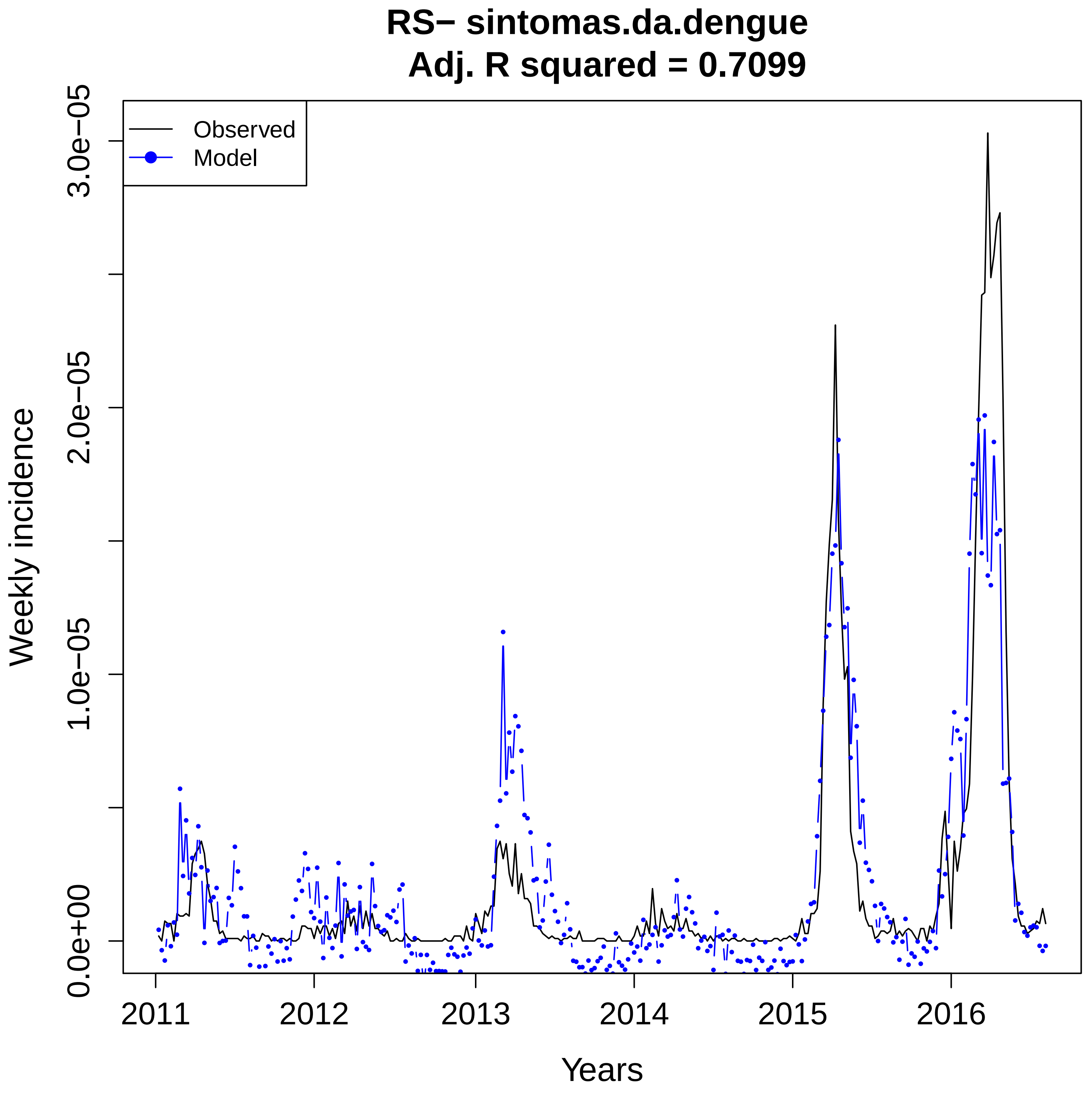

RS− sintomas.da.dengue
Adj. R squared = 0.7099
Observed
Model
Weekly incidence
0.0e+00
1.0e−05
2.0e−05
3.0e−05
2011
2012
2013
2014
2015
2016
Years

Santa Catarina (SC)
Dengue incidence vs. Google Health Trends (2011–2016)

Definitions:

- **Combined terms:**
    - **All:** model with all the available terms. n= number of terms
    - **Four:** model with "dengue", "dengue sintomas", "aedes", and "mosquito".
    - **Uncorrelated:** model with non-correlated terms. n = number of uncorrelated terms
- **Individual terms:** Plots developed with one term at a time.

- Terms for all and uncorrelated models can be found in additional file 3.

Combined terms
Adjusted R squared

Santa Catarina (SC)

All
(n = 9)

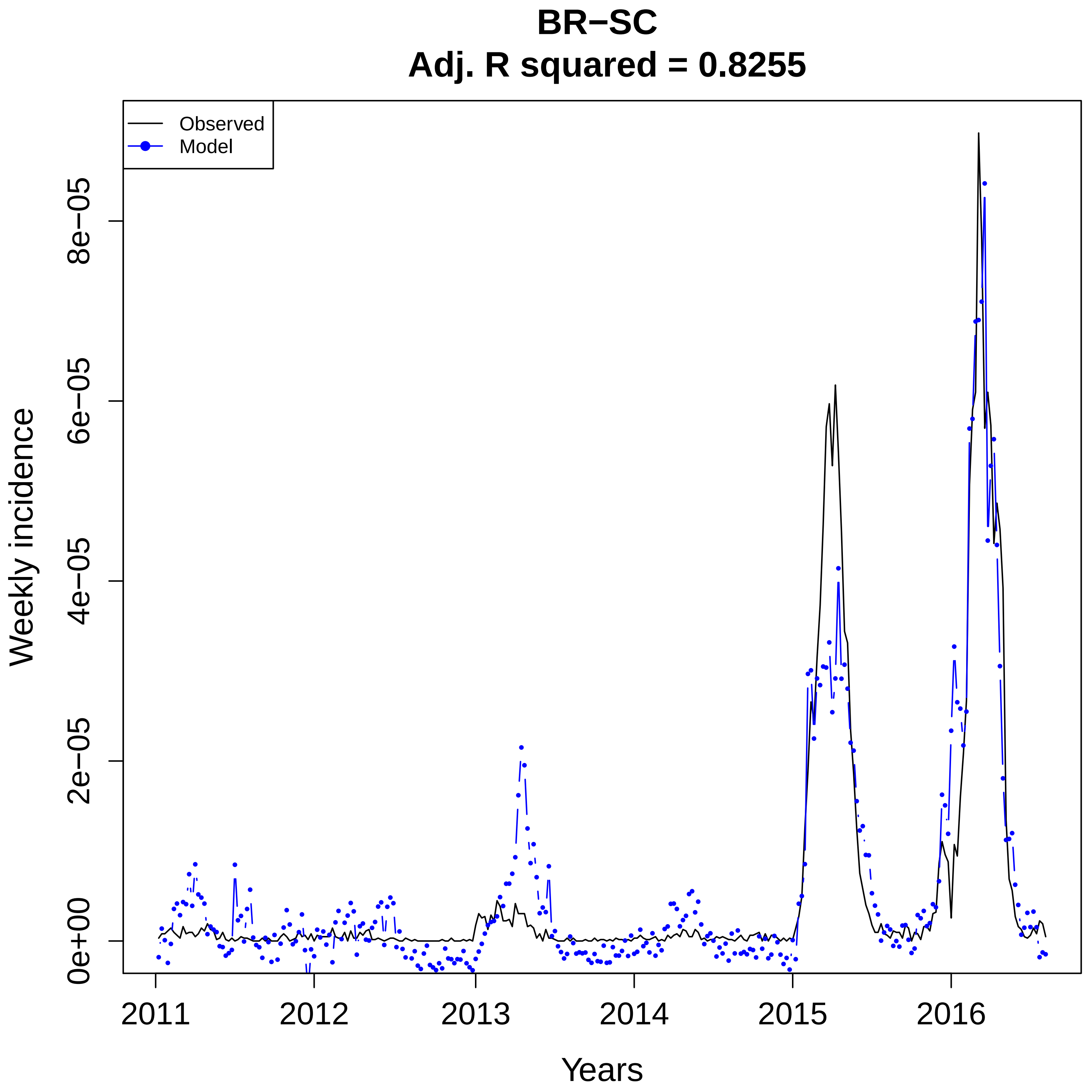

BR−SC
Adj. R squared = 0.8255
Observed
Model
Weekly incidence
0e+00
2e−05
4e−05
6e−05
8e−05
2011
2012
2013
2014
2015
2016
Years

Four

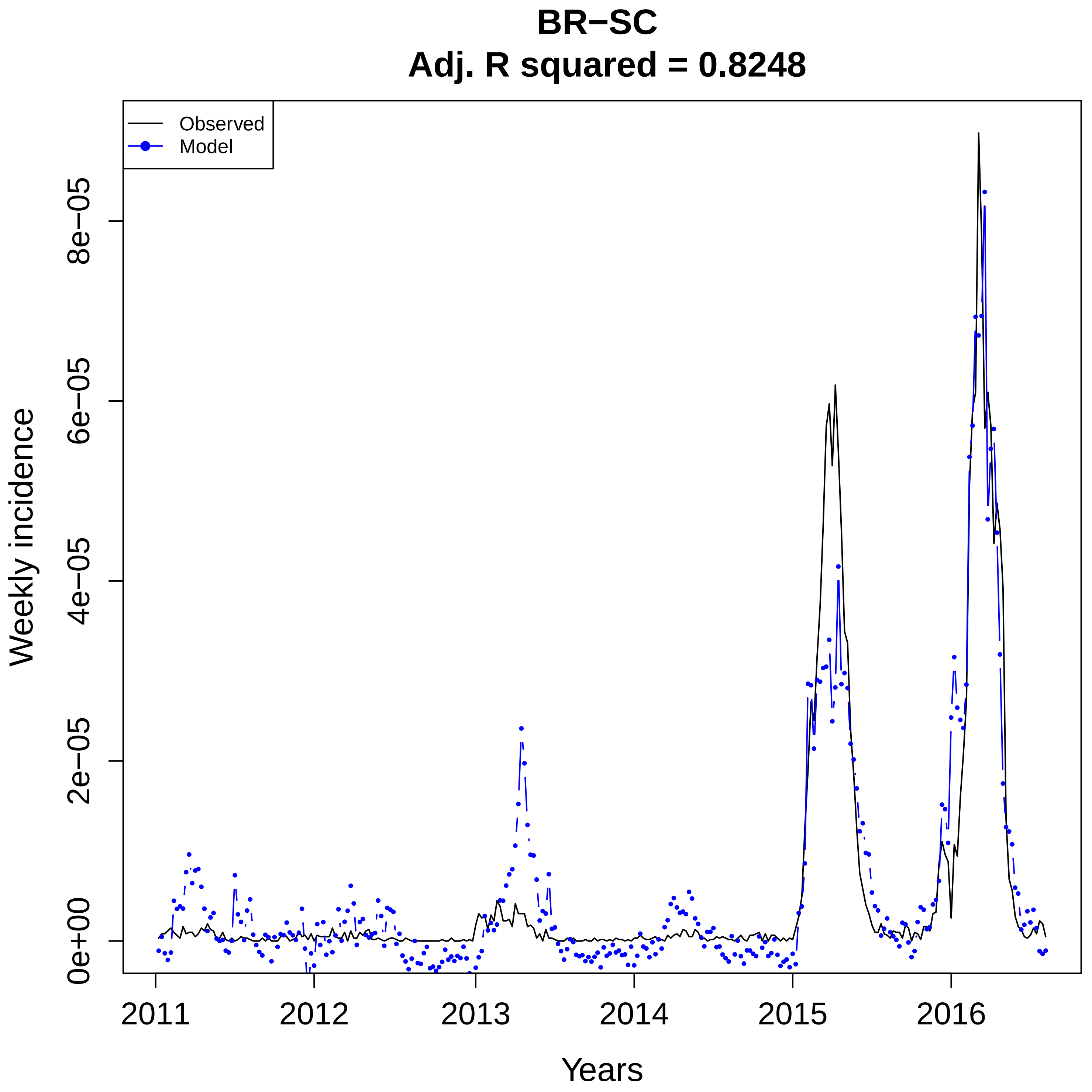

BR−SC
Adj. R squared = 0.8248
Observed
Model
Weekly incidence
0e+00
2e−05
4e−05
6e−05
8e−05
2011
2012
2013
2014
2015
2016
Years

Uncorrelated
(n = 2)

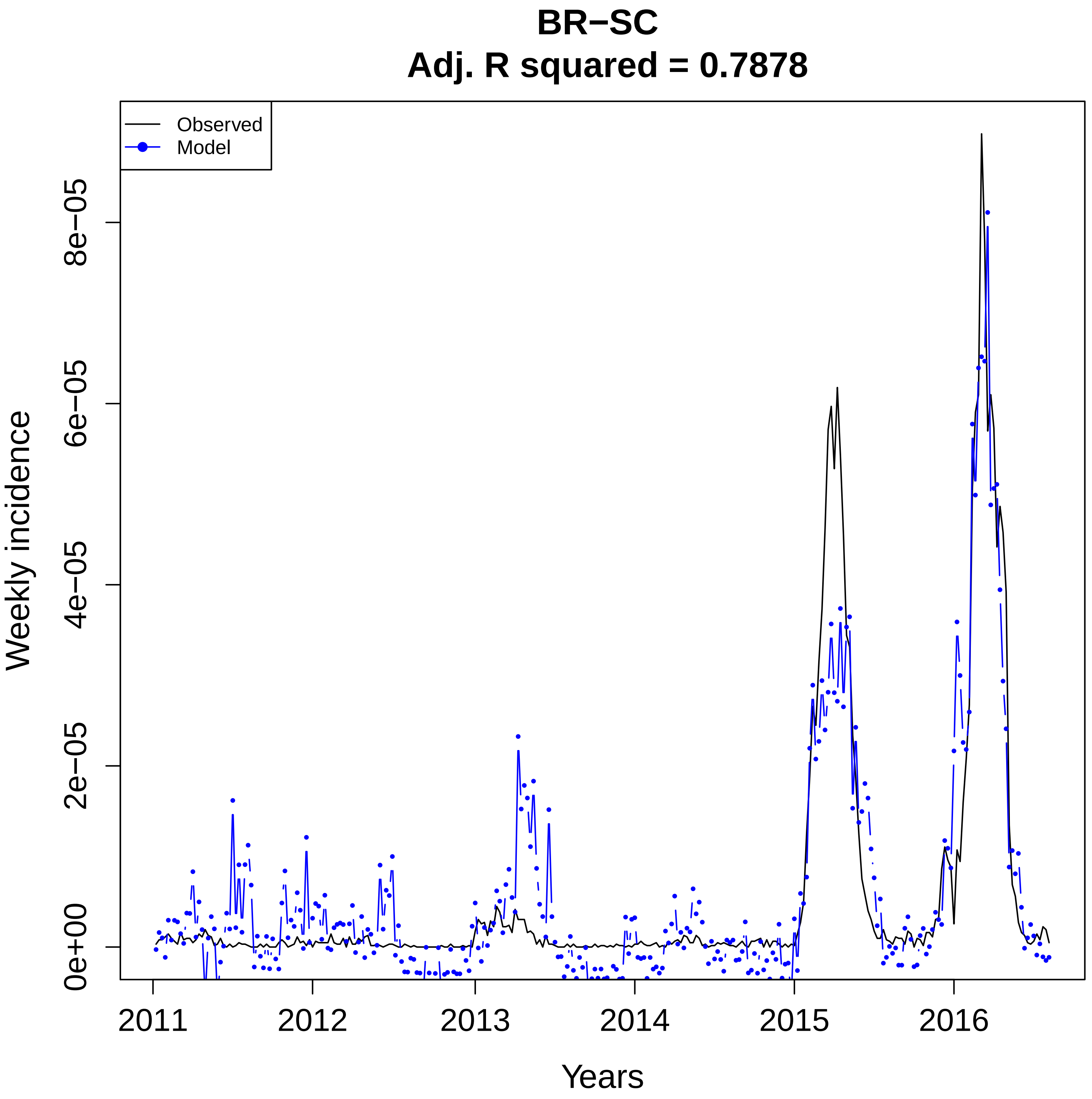

Individual terms
Adjusted R squared

Santa Catarina (SC)

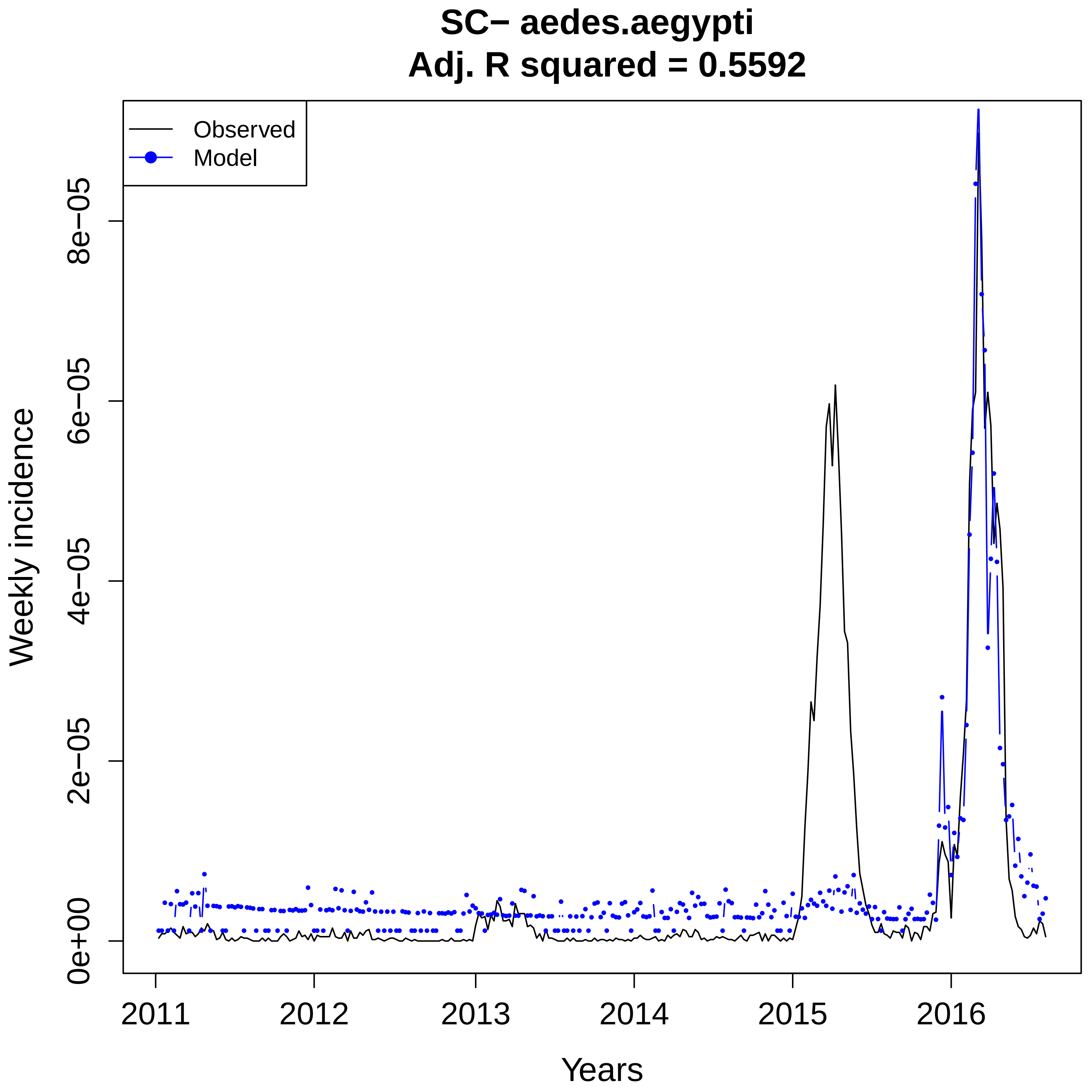
SC− aedes.aegypti
Adj. R squared = 0.5592
Observed
Model
Weekly incidence
0e+00
2e−05
4e−05
6e−05
8e−05
2011
2012
2013
2014
2015
2016
Years

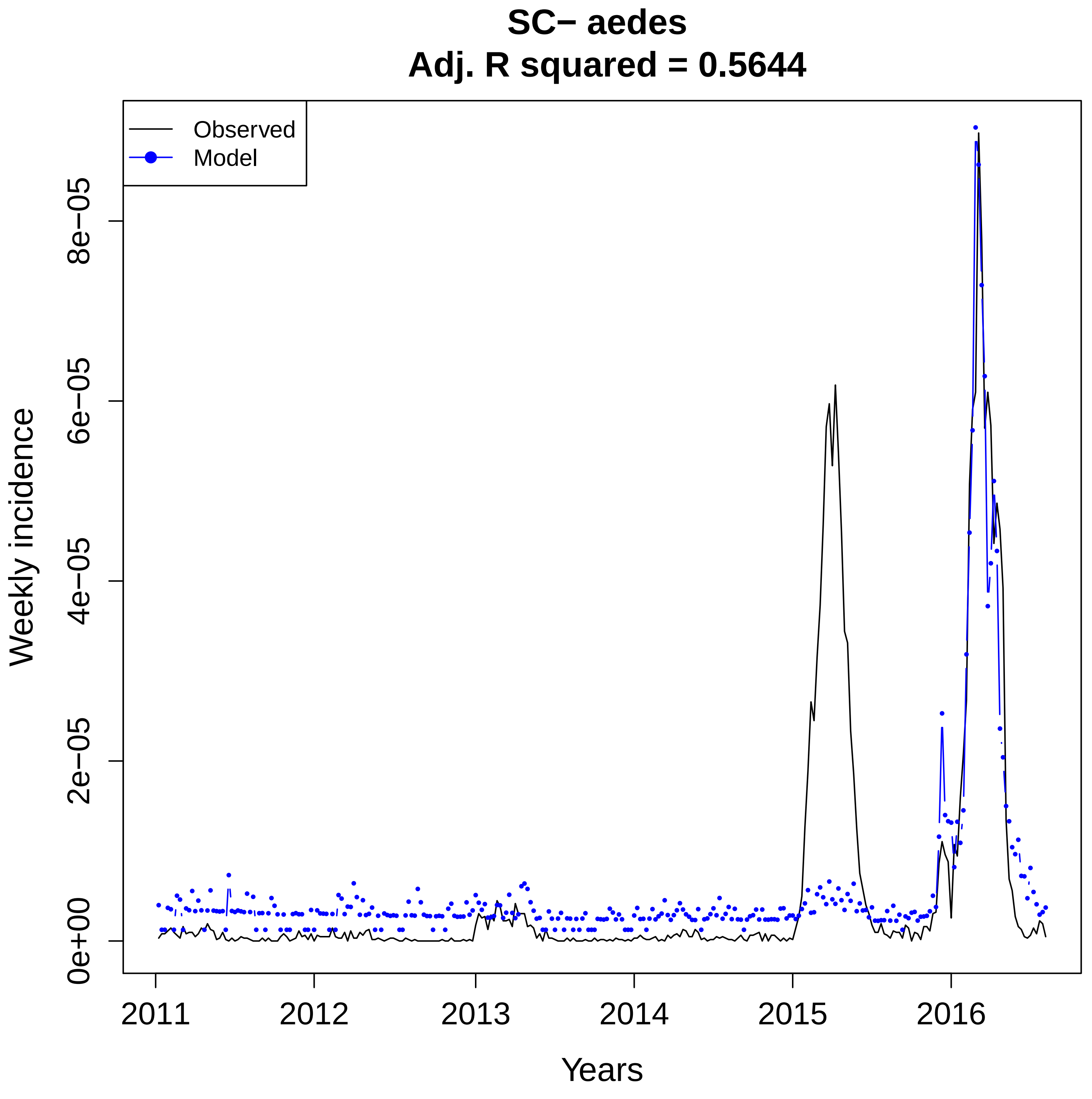

SC− aedes
Adj. R squared = 0.5644
Observed
Model
Weekly incidence
0e+00
2e−05
4e−05
6e−05
8e−05
2011
2012
2013
2014
2015
2016
Years

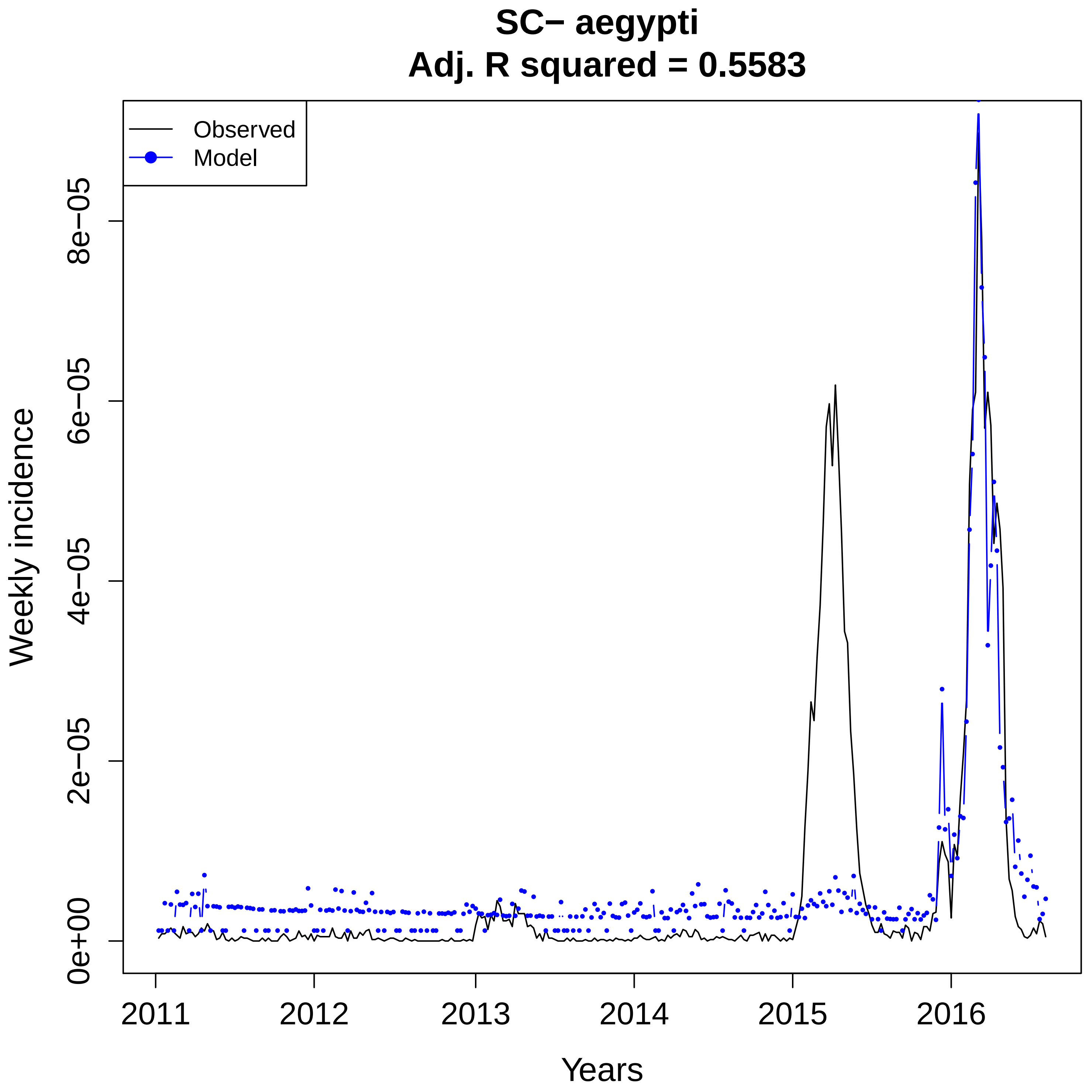
SC− aegypti
Adj. R squared = 0.5583
Observed
Model
Weekly incidence
0e+00
2e−05
4e−05
6e−05
8e−05
2011
2012
2013
2014
2015
2016
Years

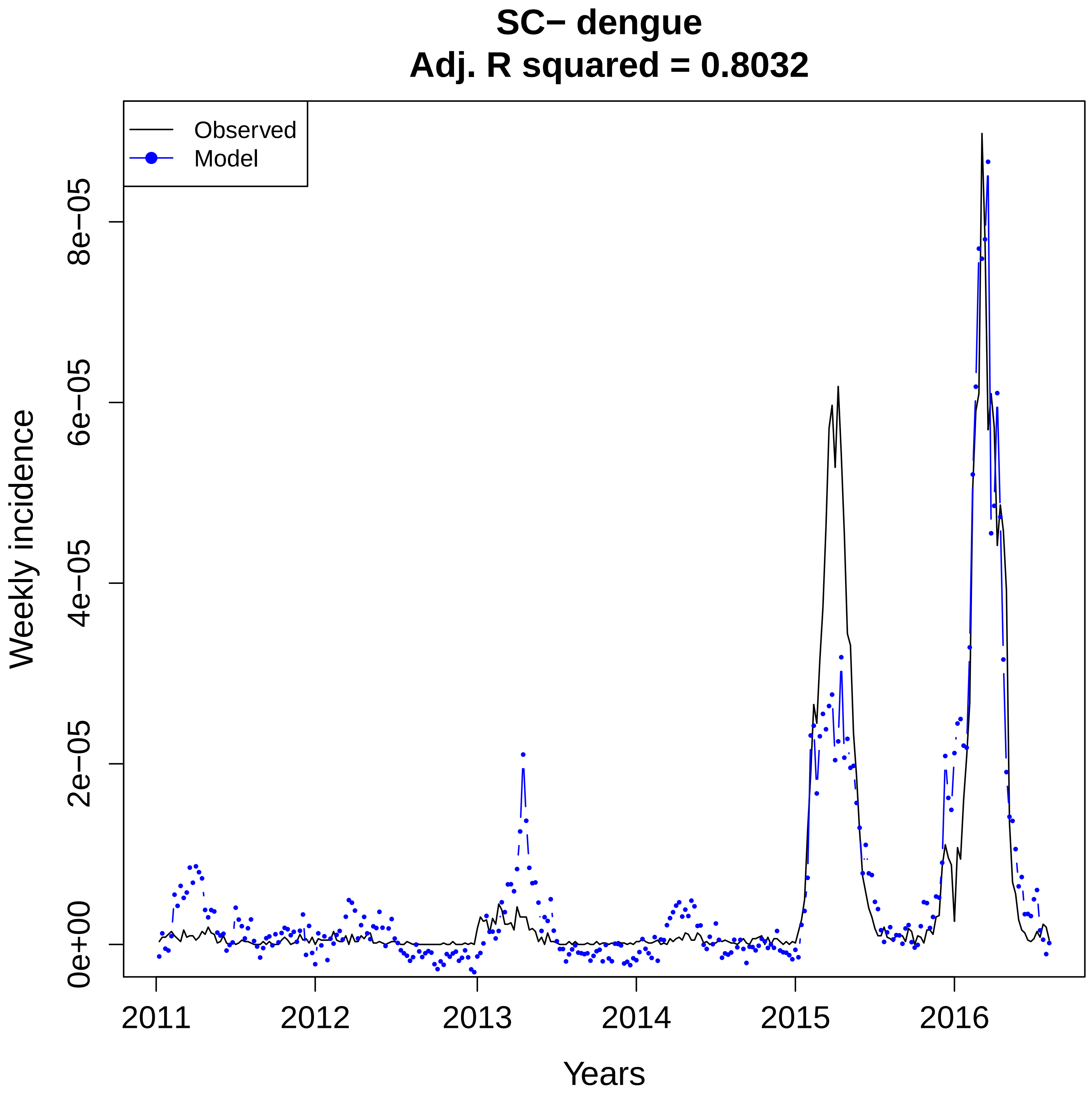
SC− dengue
Adj. R squared = 0.8032
Observed
Model
Weekly incidence
0e+00
2e−05
4e−05
6e−05
8e−05
2011
2012
2013
2014
2015
2016
Years

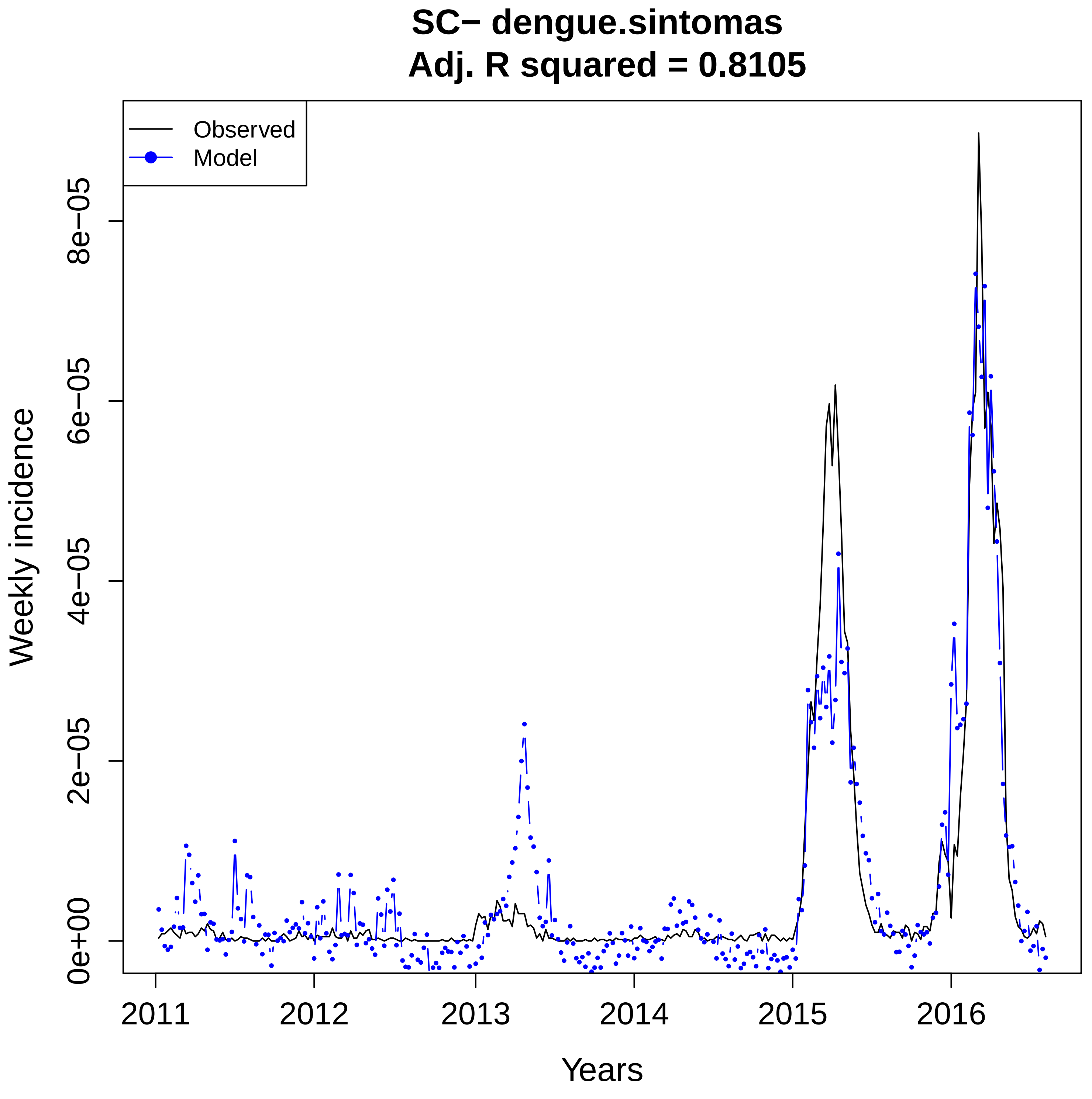

SC− dengue.sintomas
Adj. R squared = 0.8105
Observed
Model
Weekly incidence
0e+00
2e−05
4e−05
6e−05
8e−05
2011
2012
2013
2014
2015
2016
Years

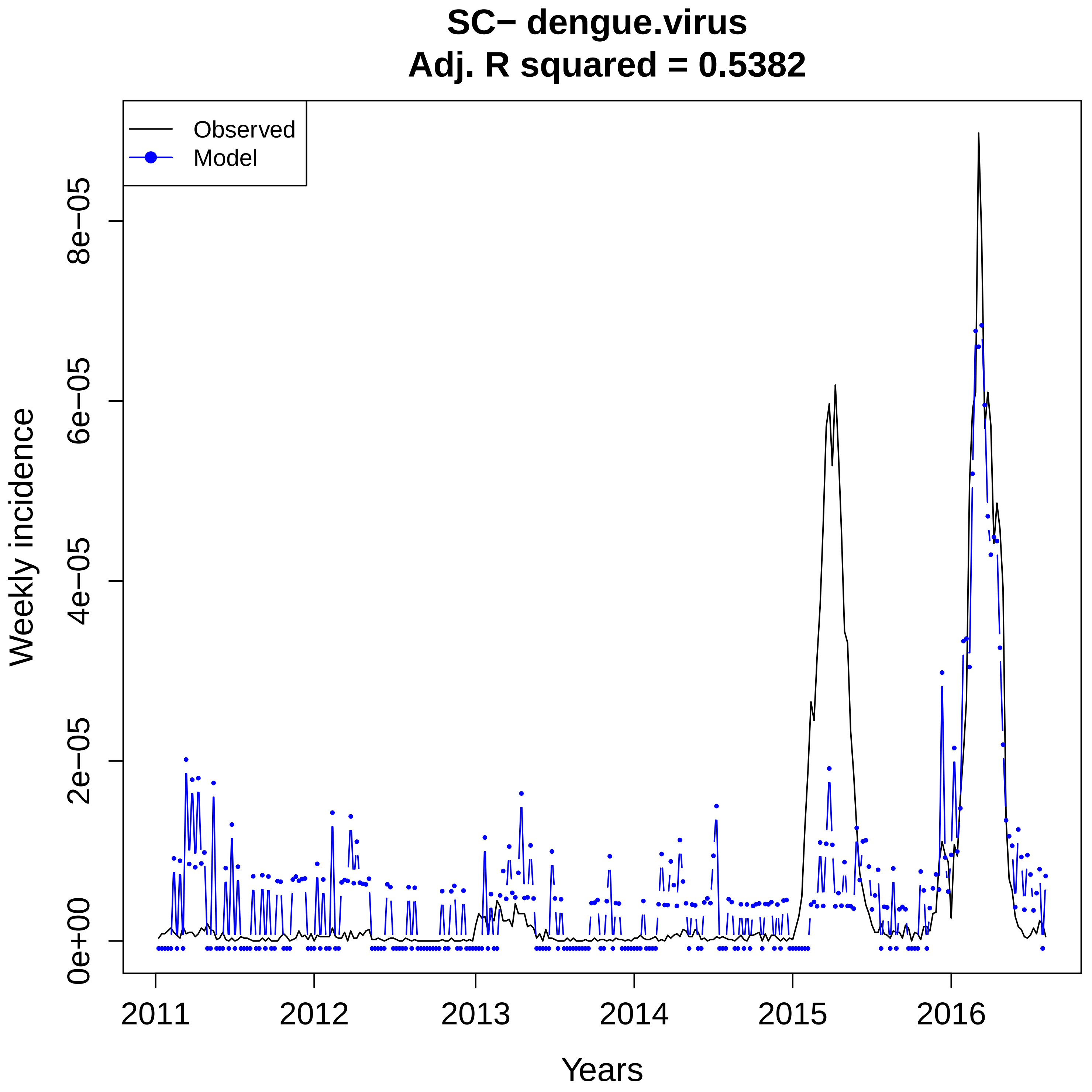
SC− dengue.virus
Adj. R squared = 0.5382
Observed
Model
Weekly incidence
0e+00
2e−05
4e−05
6e−05
8e−05
2011
2012
2013
2014
2015
2016
Years

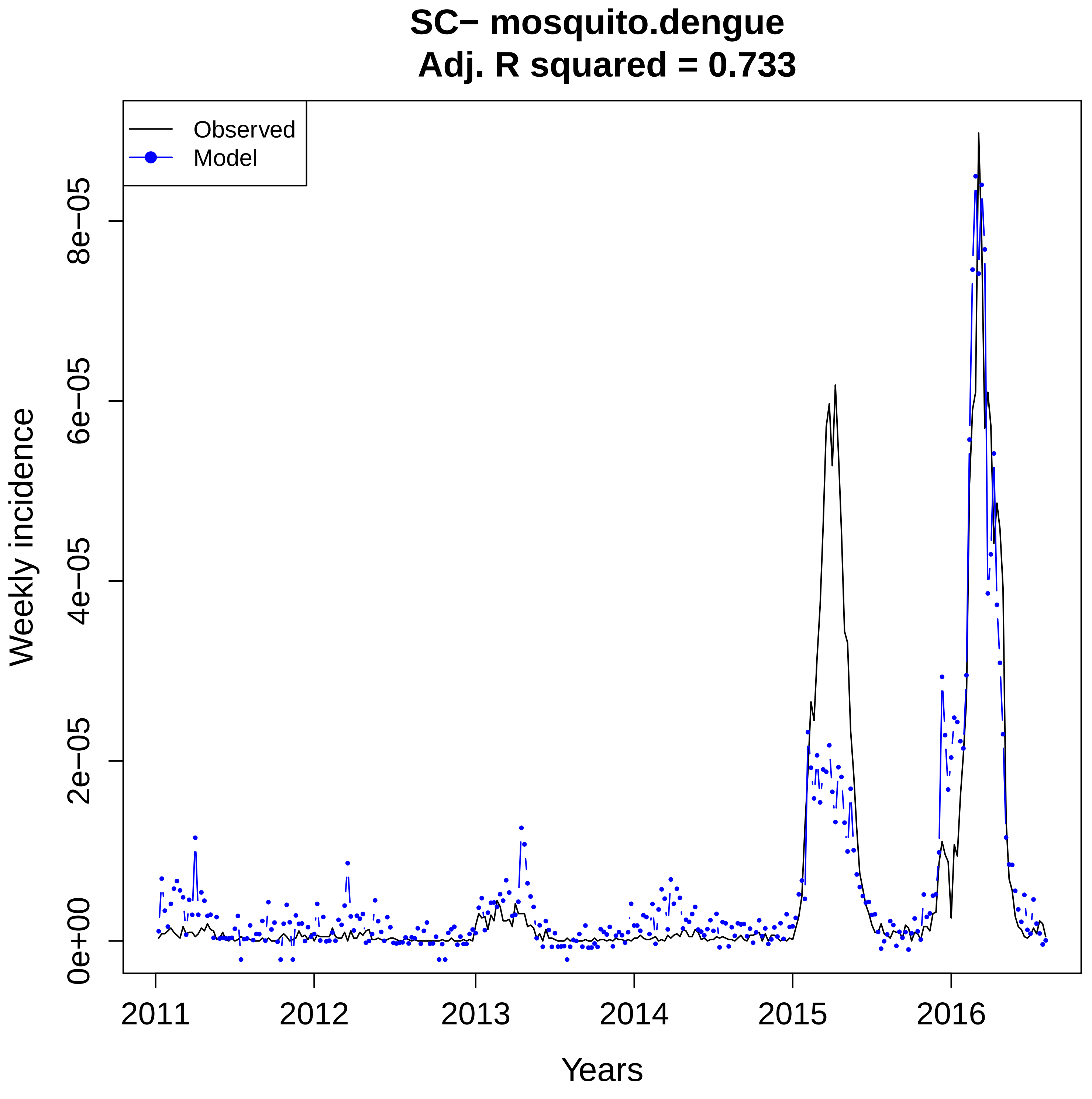
SC− mosquito.dengue
Adj. R squared = 0.733
Observed
Model
Weekly incidence
0e+00
2e−05
4e−05
6e−05
8e−05
2011
2012
2013
2014
2015
2016
Years

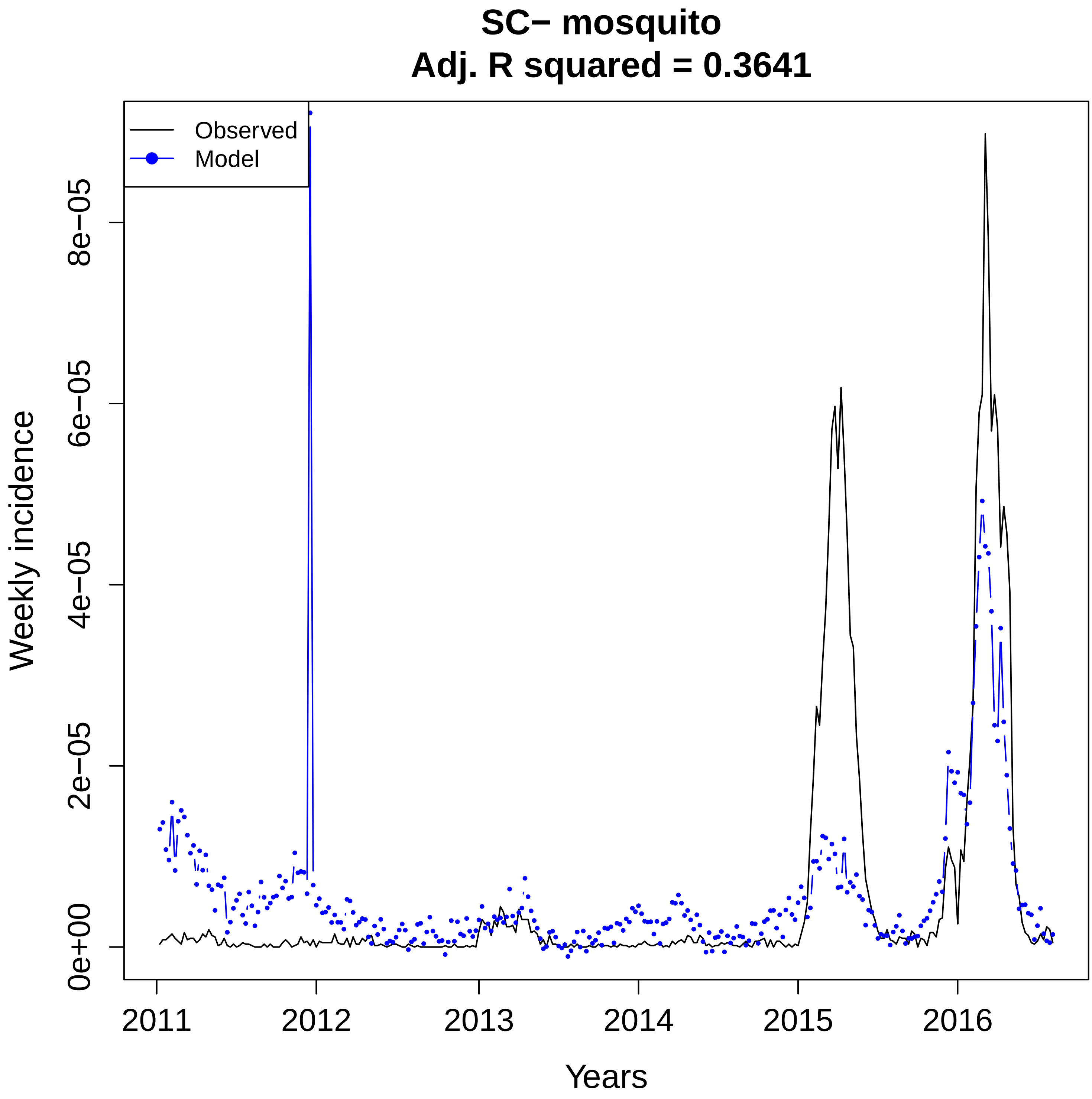
SC− mosquito
Adj. R squared = 0.3641
Observed
Model
Weekly incidence
0e+00
2e−05
4e−05
6e−05
8e−05
2011
2012
2013
2014
2015
2016
Years

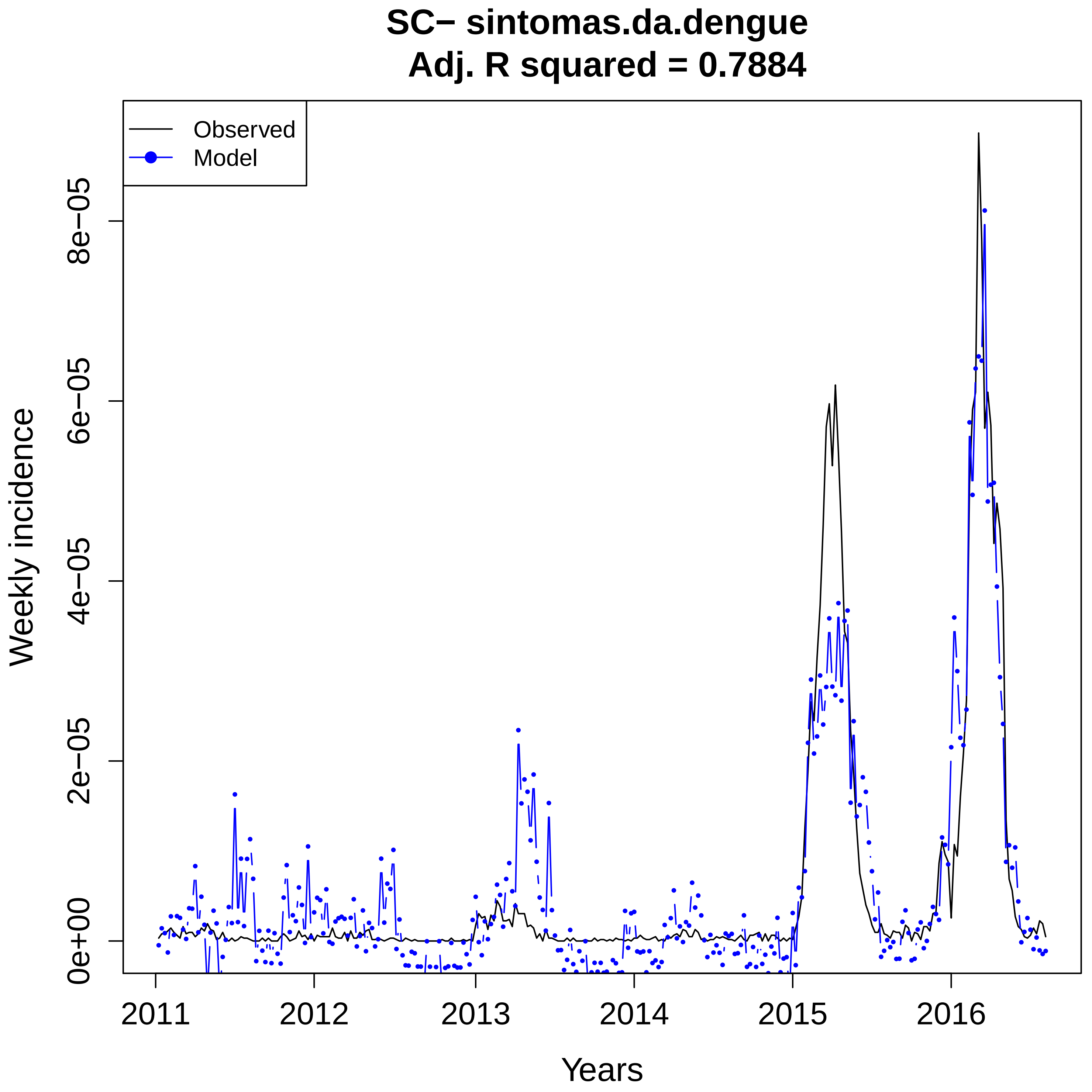

Sergipe (SE)
Dengue incidence vs. Google Health Trends (2011–2016)

Definitions:

- **Combined terms:**
    - **All:** model with all the available terms. n= number of terms
    - **Four:** model with "dengue", "dengue sintomas", "aedes", and "mosquito".
    - **Uncorrelated:** model with non-correlated terms. n = number of uncorrelated terms
- **Individual terms:** Plots developed with one term at a time.

- Terms for all and uncorrelated models can be found in additional file 3.

Combined terms
Adjusted R squared

Sergipe (SE)

All
(n = 8)

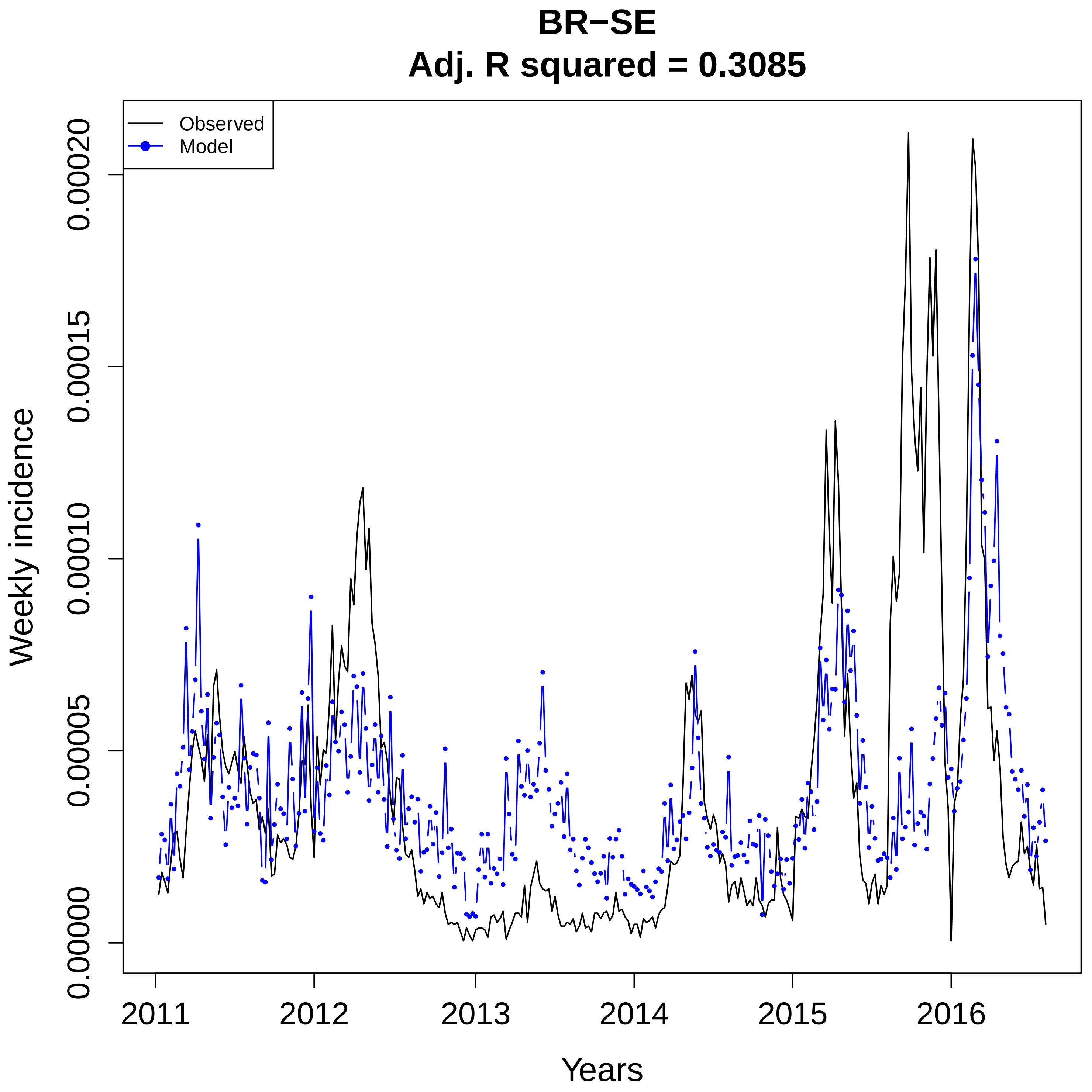

BR−SE
Adj. R squared = 0.3085
Observed
Model
Weekly incidence
0.00000
0.00005
0.00010
0.00015
0.00020
2011
2012
2013
2014
2015
2016
Years

Four

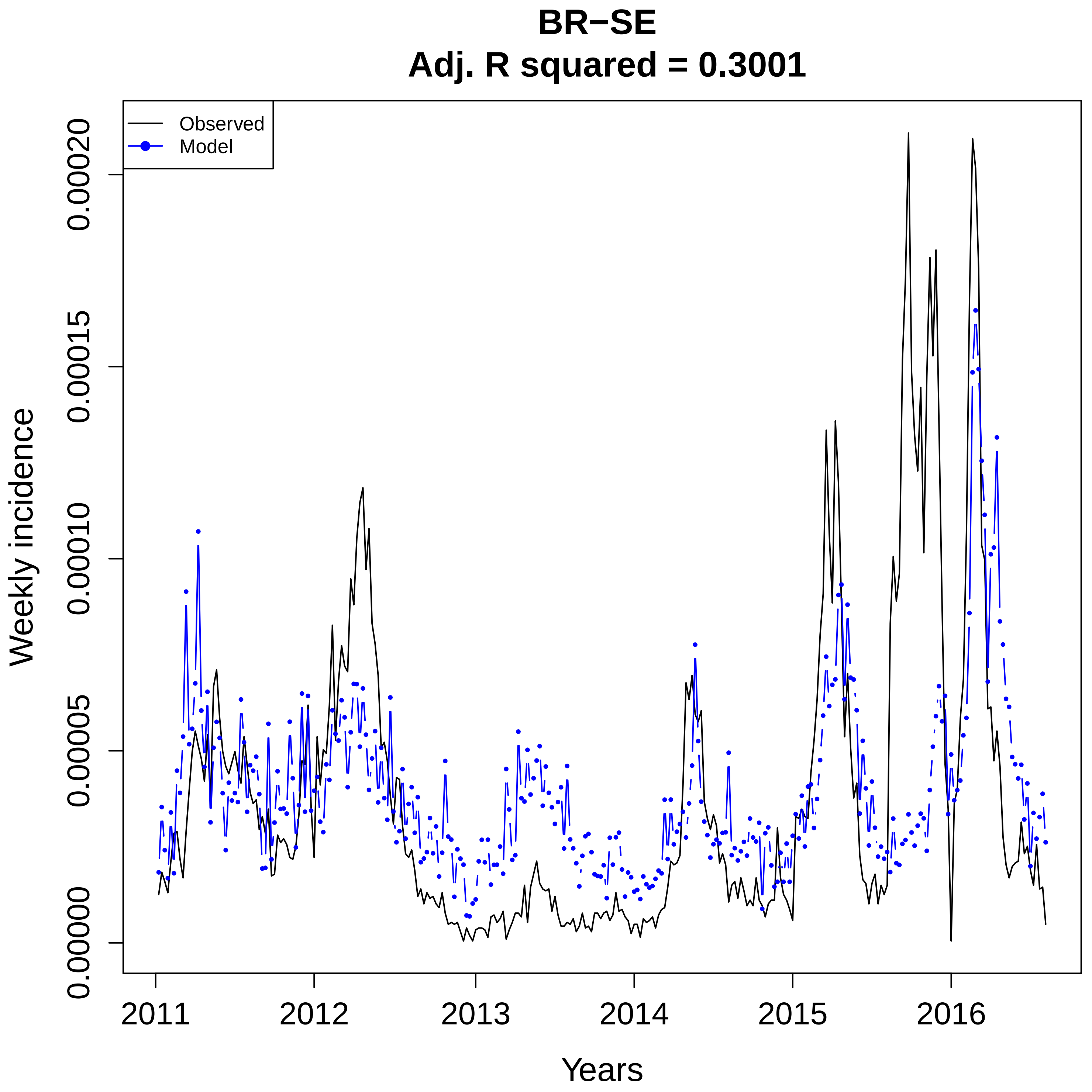

BR−SE
Adj. R squared = 0.3001
Observed
Model
Weekly incidence
0.00000
0.00005
0.00010
0.00015
0.00020
2011
2012
2013
2014
2015
2016
Years

Uncorrelated
(n = 3)

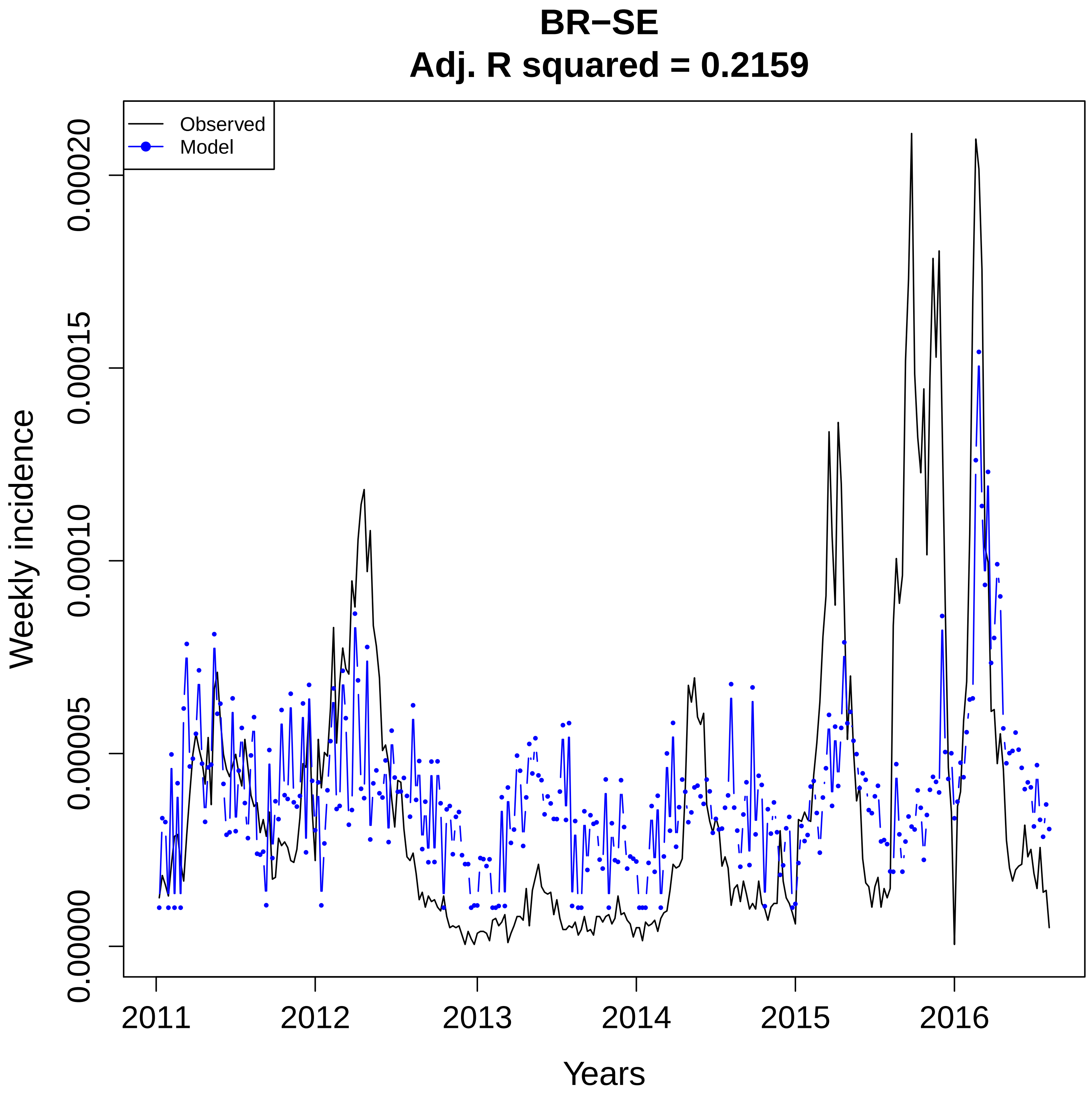

Individual terms
Adjusted R squared

Sergipe (SE)

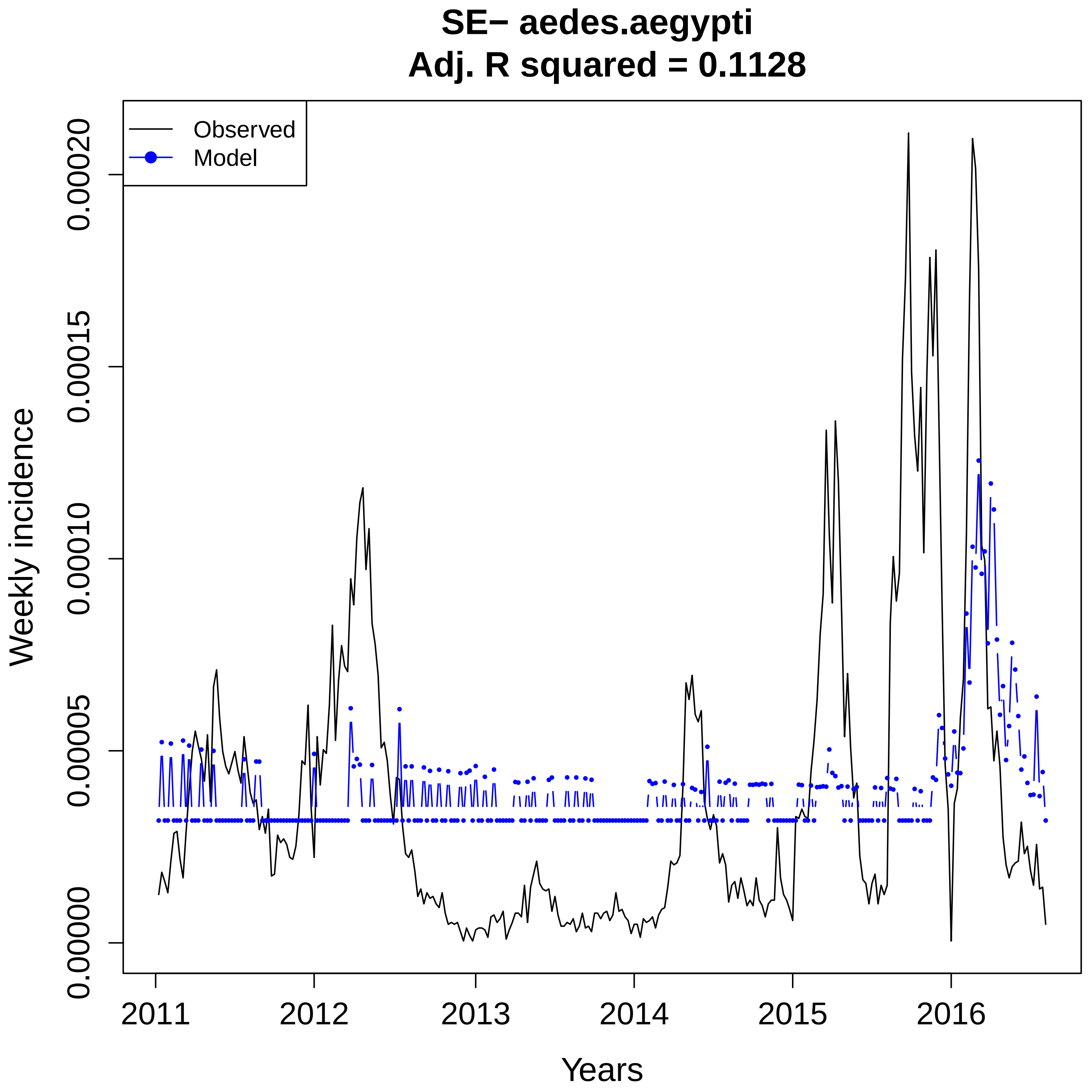

SE− aedes.aegypti
Adj. R squared = 0.1128
Observed
Model
Weekly incidence
0.00000
0.00005
0.00010
0.00015
0.00020
2011
2012
2013
2014
2015
2016
Years

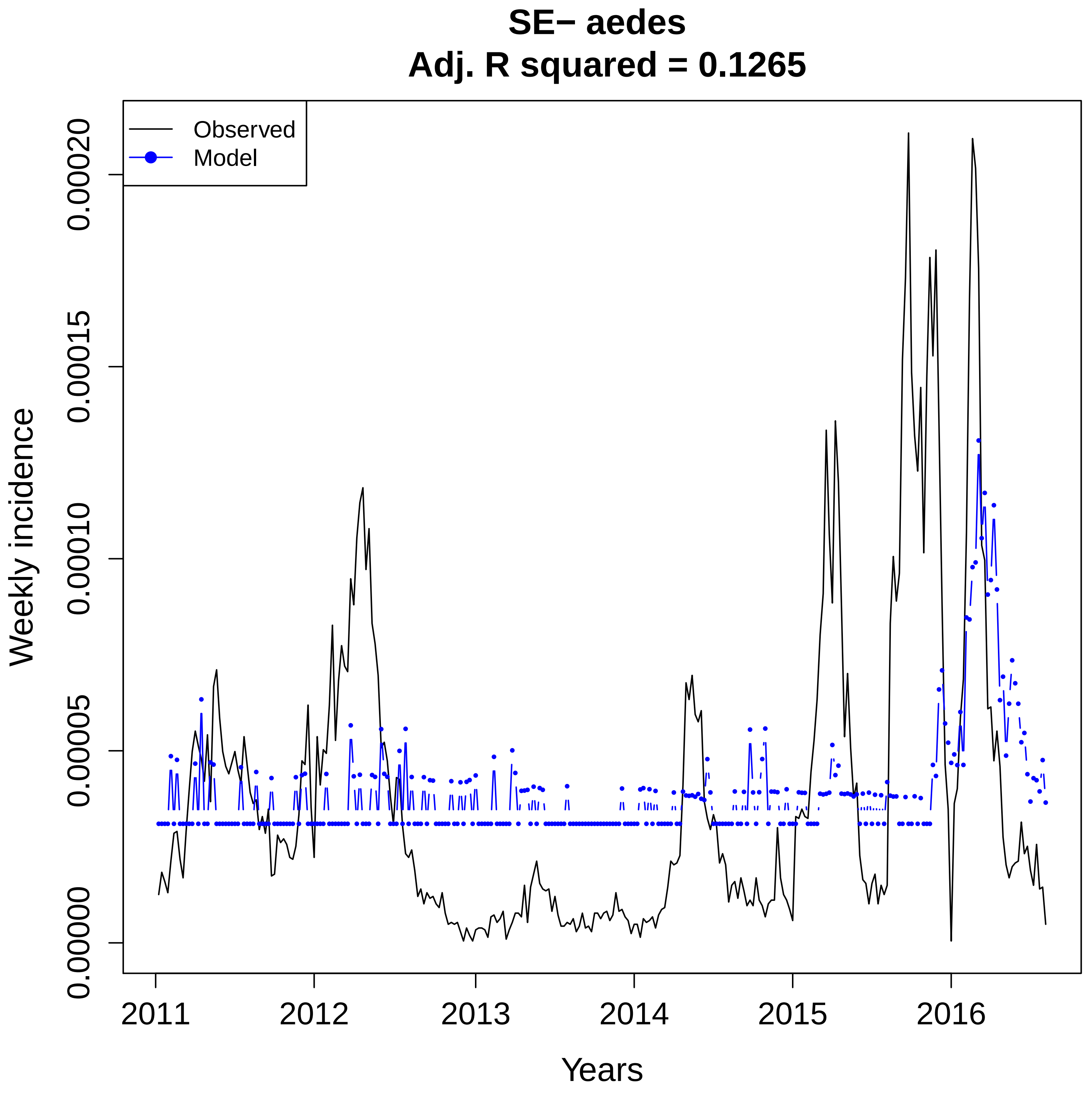

SE− aedes
Adj. R squared = 0.1265
Observed
Model
Weekly incidence
0.00000
0.00005
0.00010
0.00015
0.00020
2011
2012
2013
2014
2015
2016
Years

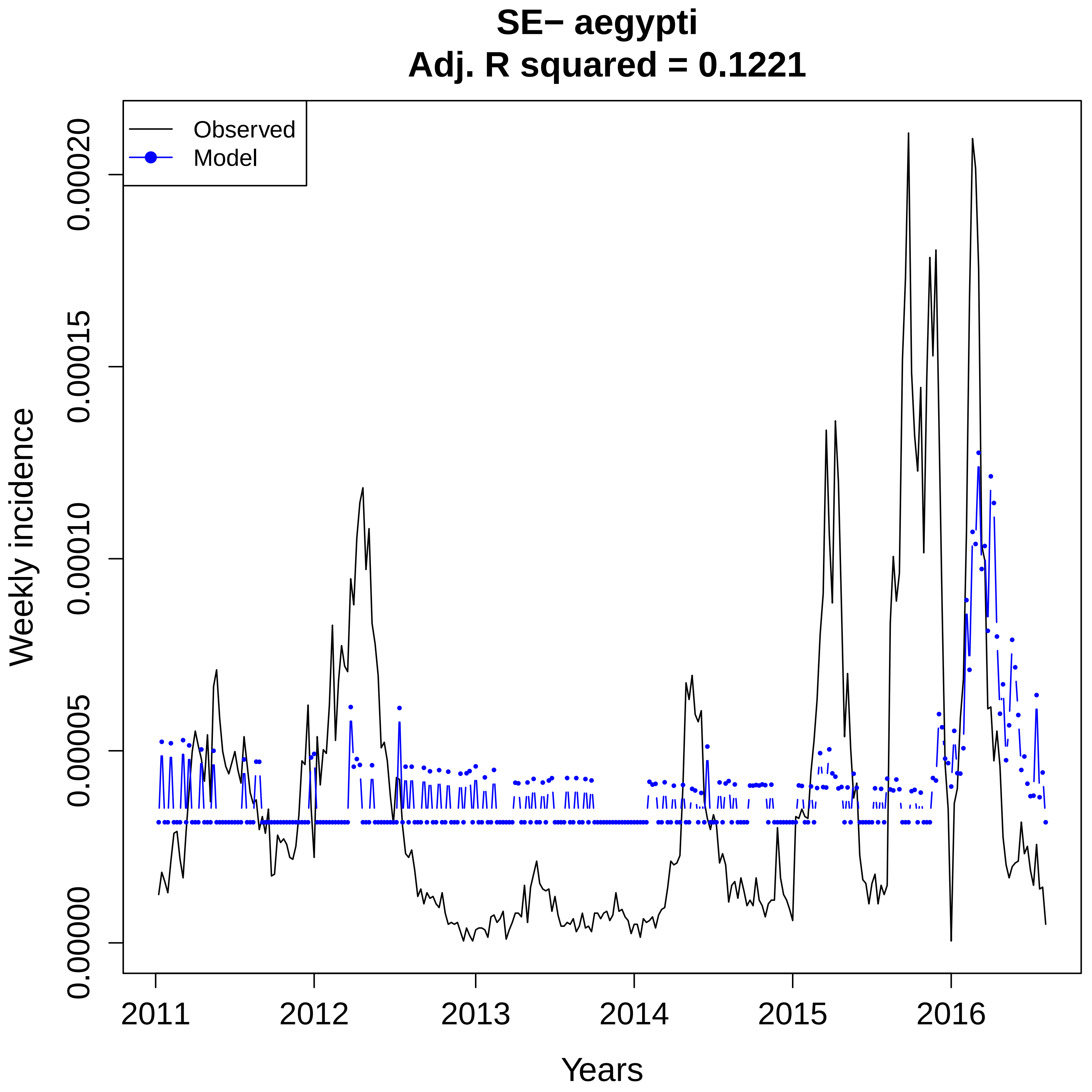

SE− aegypti
Adj. R squared = 0.1221
Observed
Model
Weekly incidence
0.00000
0.00005
0.00010
0.00015
0.00020
2011
2012
2013
2014
2015
2016
Years

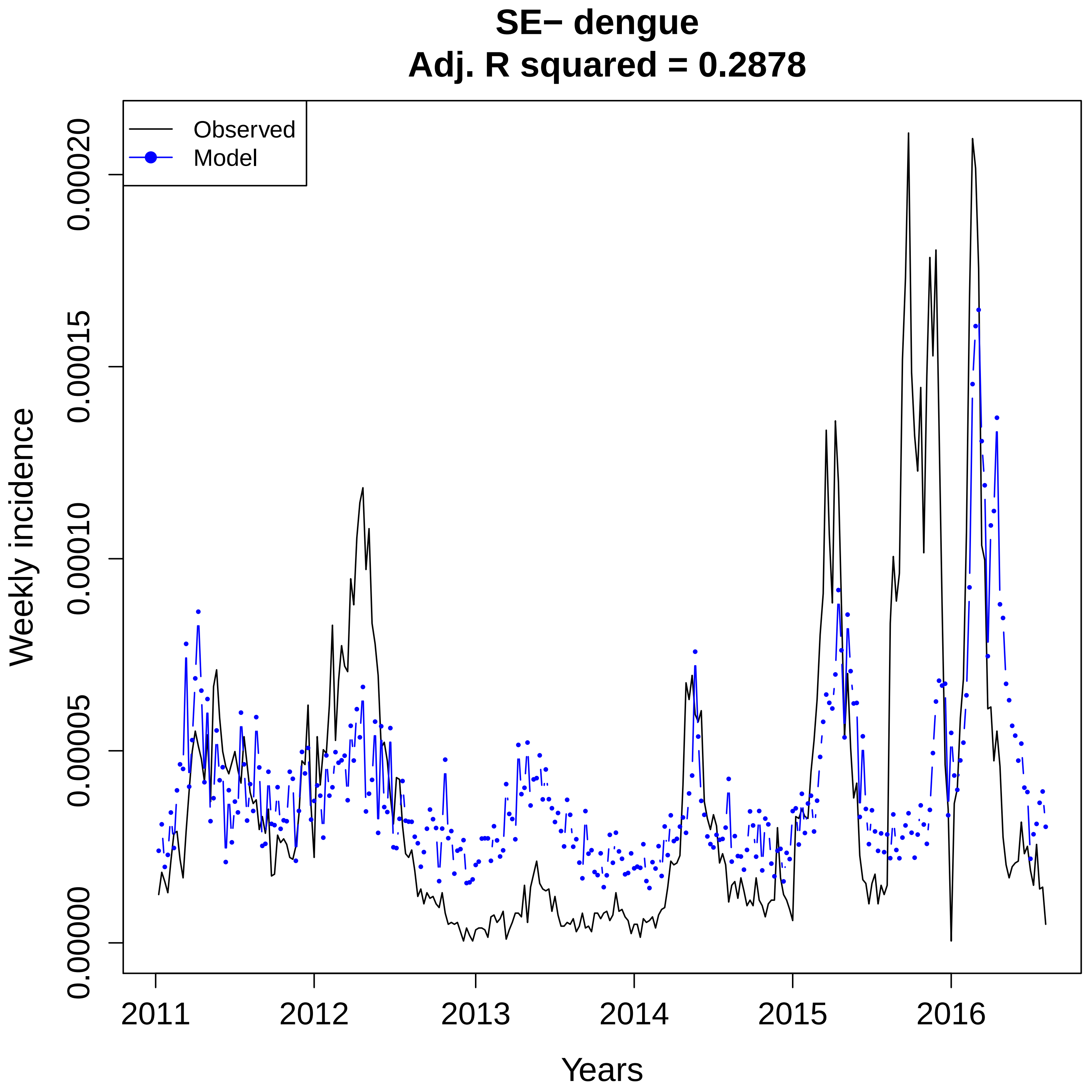

SE− dengue
Adj. R squared = 0.2878
Observed
Model
Weekly incidence
0.00000
0.00005
0.00010
0.00015
0.00020
2011
2012
2013
2014
2015
2016
Years

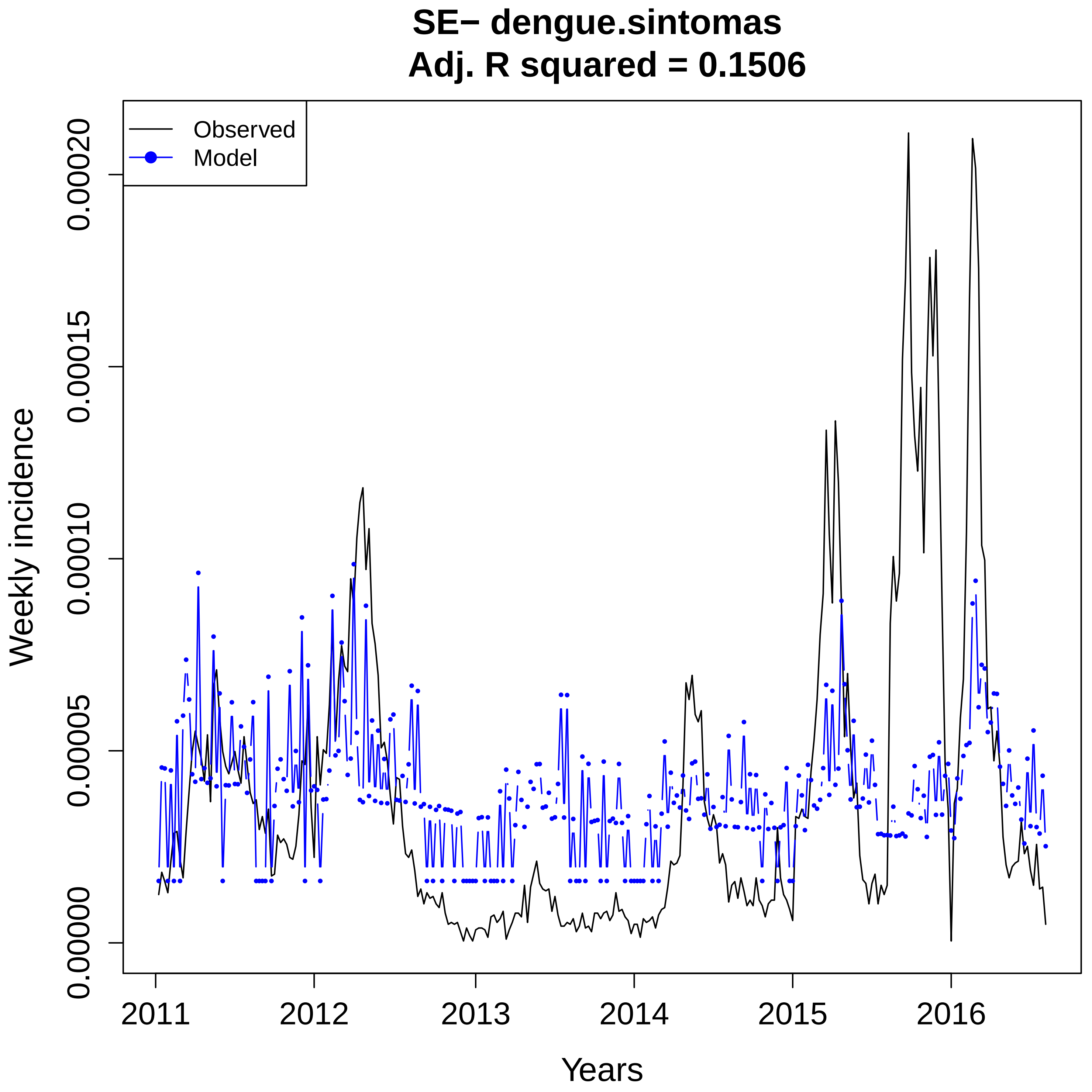

SE− dengue.sintomas
Adj. R squared = 0.1506
Observed
Model
Weekly incidence
0.00000
0.00005
0.00010
0.00015
0.00020
2011
2012
2013
2014
2015
2016
Years

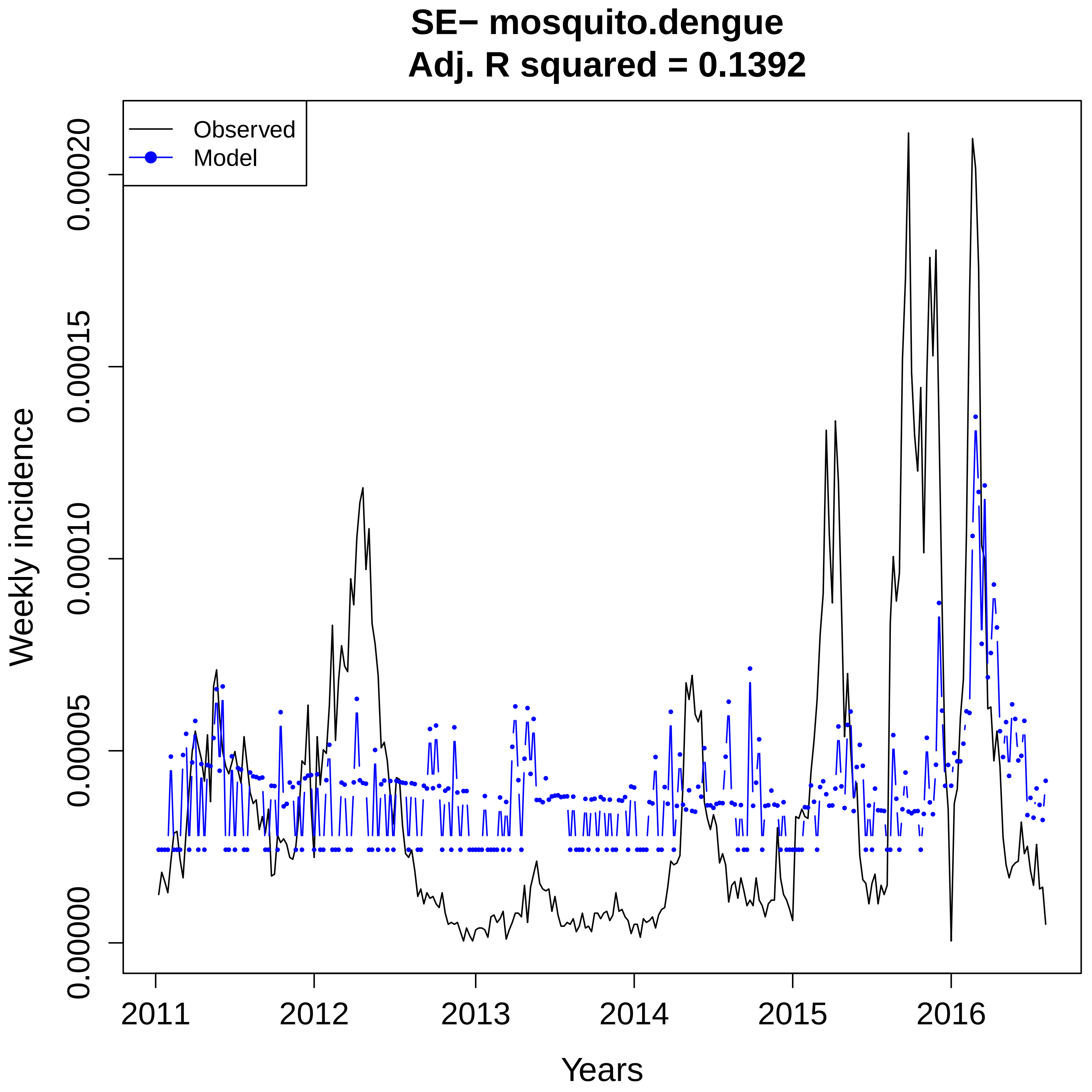
SE− mosquito.dengue
Adj. R squared = 0.1392
Observed
Model
Weekly incidence
0.00000
0.00005
0.00010
0.00015
0.00020
2011
2012
2013
2014
2015
2016
Years

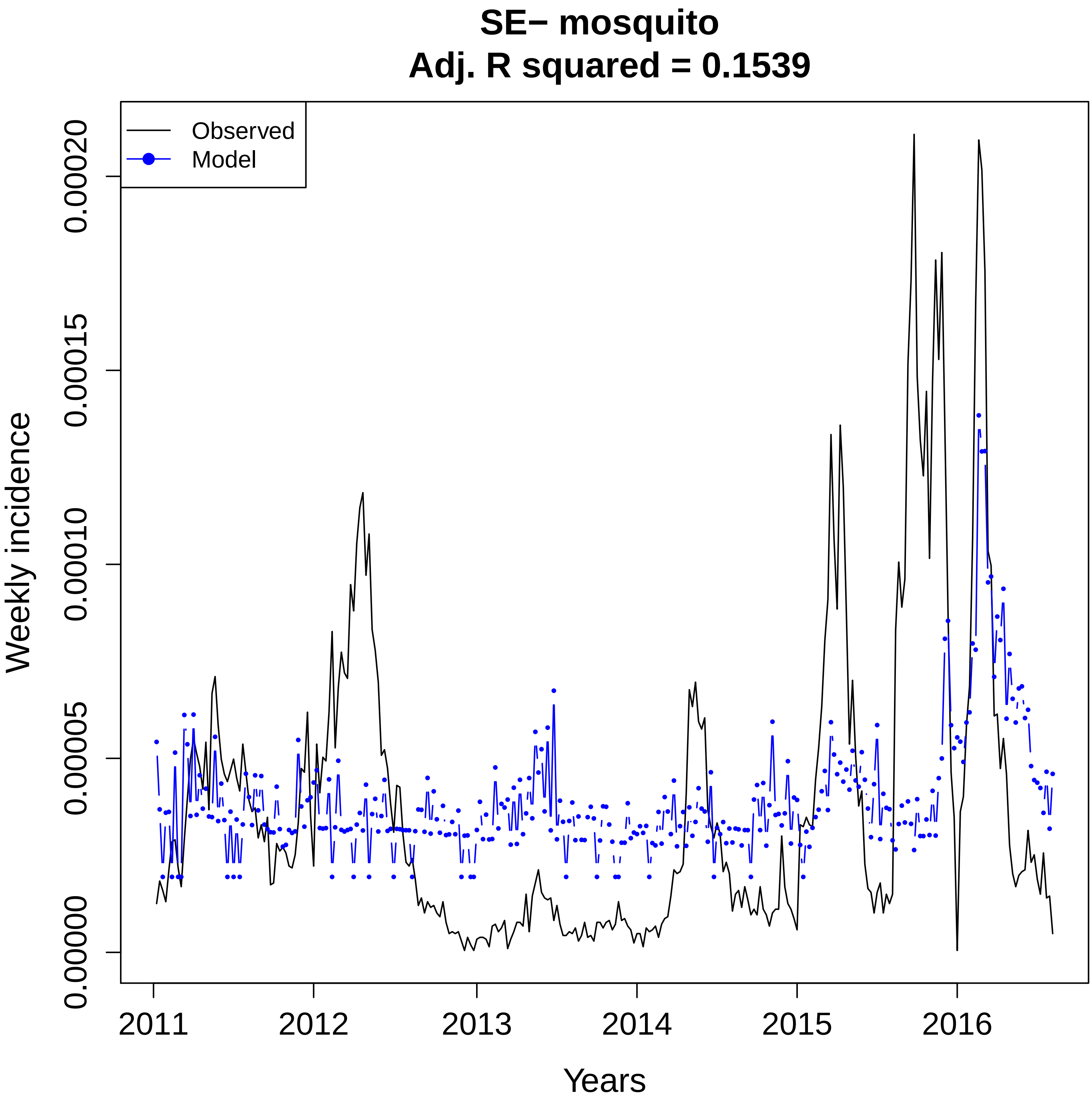

SE− mosquito
Adj. R squared = 0.1539
Observed
Model
Weekly incidence
0.00000
0.00005
0.00010
0.00015
0.00020
2011
2012
2013
2014
2015
2016
Years

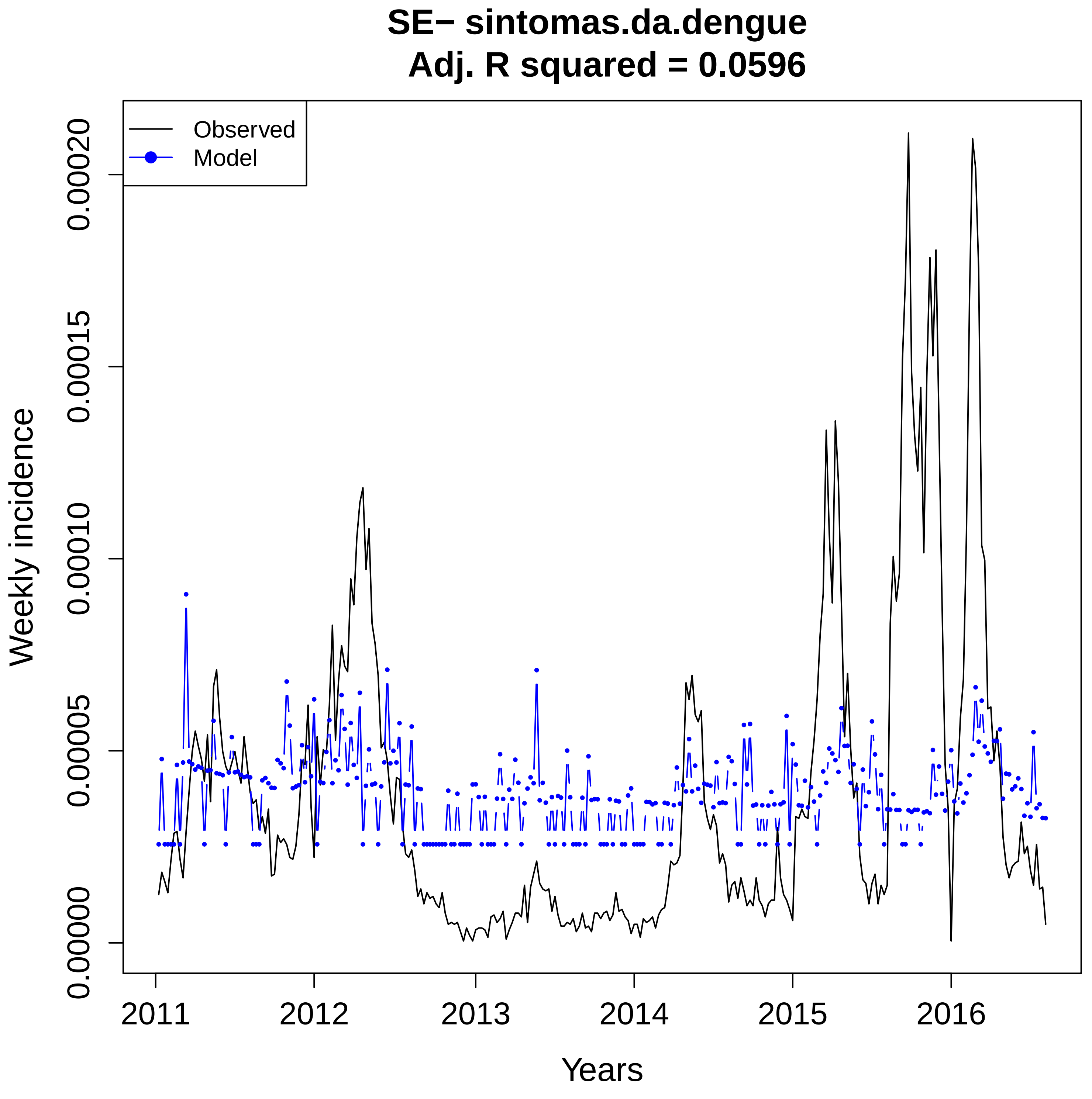

SE− sintomas.da.dengue
Adj. R squared = 0.0596
Observed
Model
Weekly incidence
0.00000
0.00005
0.00010
0.00015
0.00020
2011
2012
2013
2014
2015
2016
Years

São Paulo (SP)

Dengue incidence vs. Google Health Trends (2011–2016)

Definitions:

- **Combined terms:**
  - **All:** model with all the available terms. n= number of terms
  - **Four:** model with "dengue", "dengue sintomas", "aedes", and "mosquito".
  - **Uncorrelated:** model with non-correlated terms. n = number of uncorrelated terms
- **Individual terms:** Plots developed with one term at a time.

- Terms for all and uncorrelated models can be found in additional file 3.

Combined terms
Adjusted R squared

São Paulo (SP)

All
(n = 13)

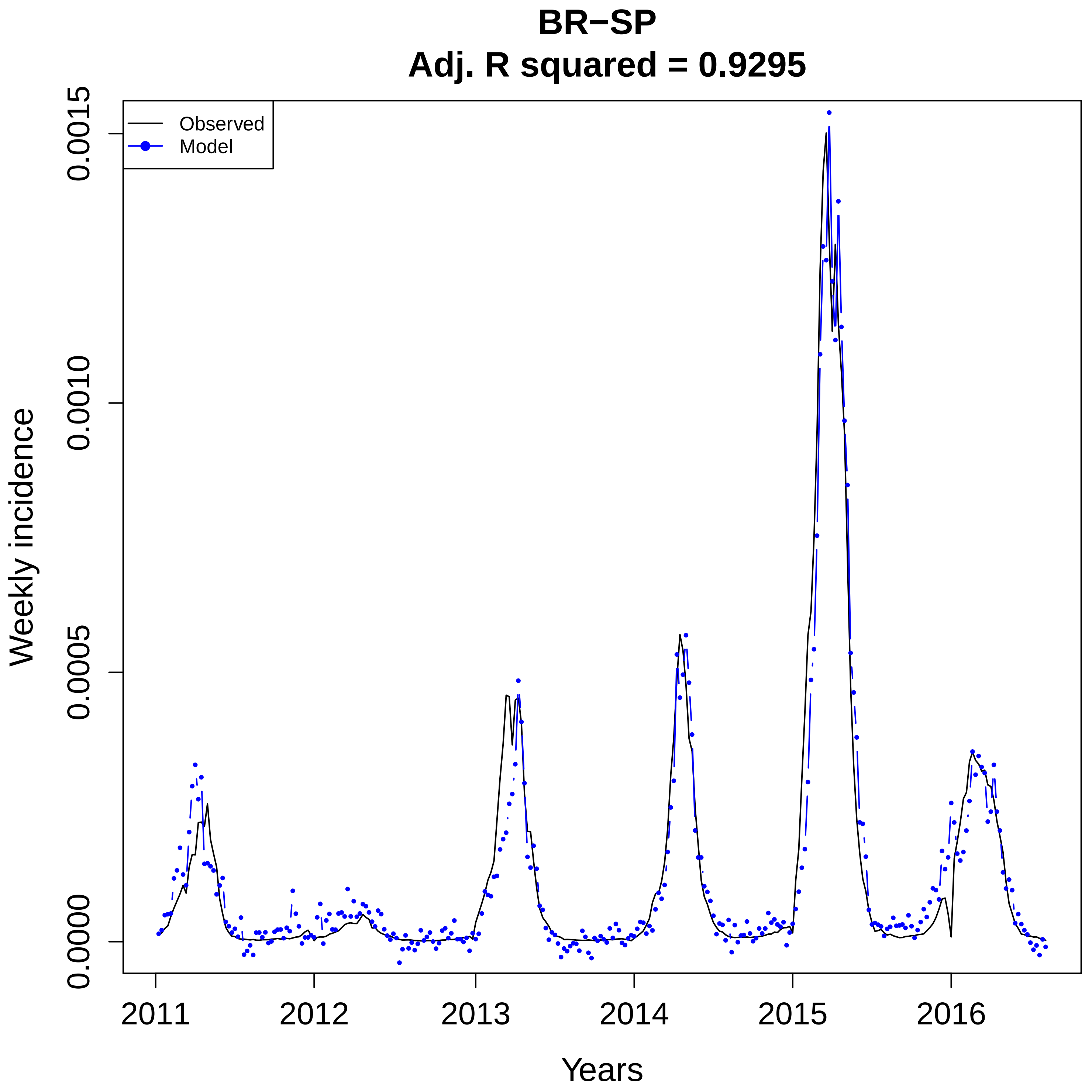

BR−SP
Adj. R squared = 0.9295
Observed
Model
Weekly incidence
0.0000
0.0005
0.0010
0.0015
2011
2012
2013
2014
2015
2016
Years

Four

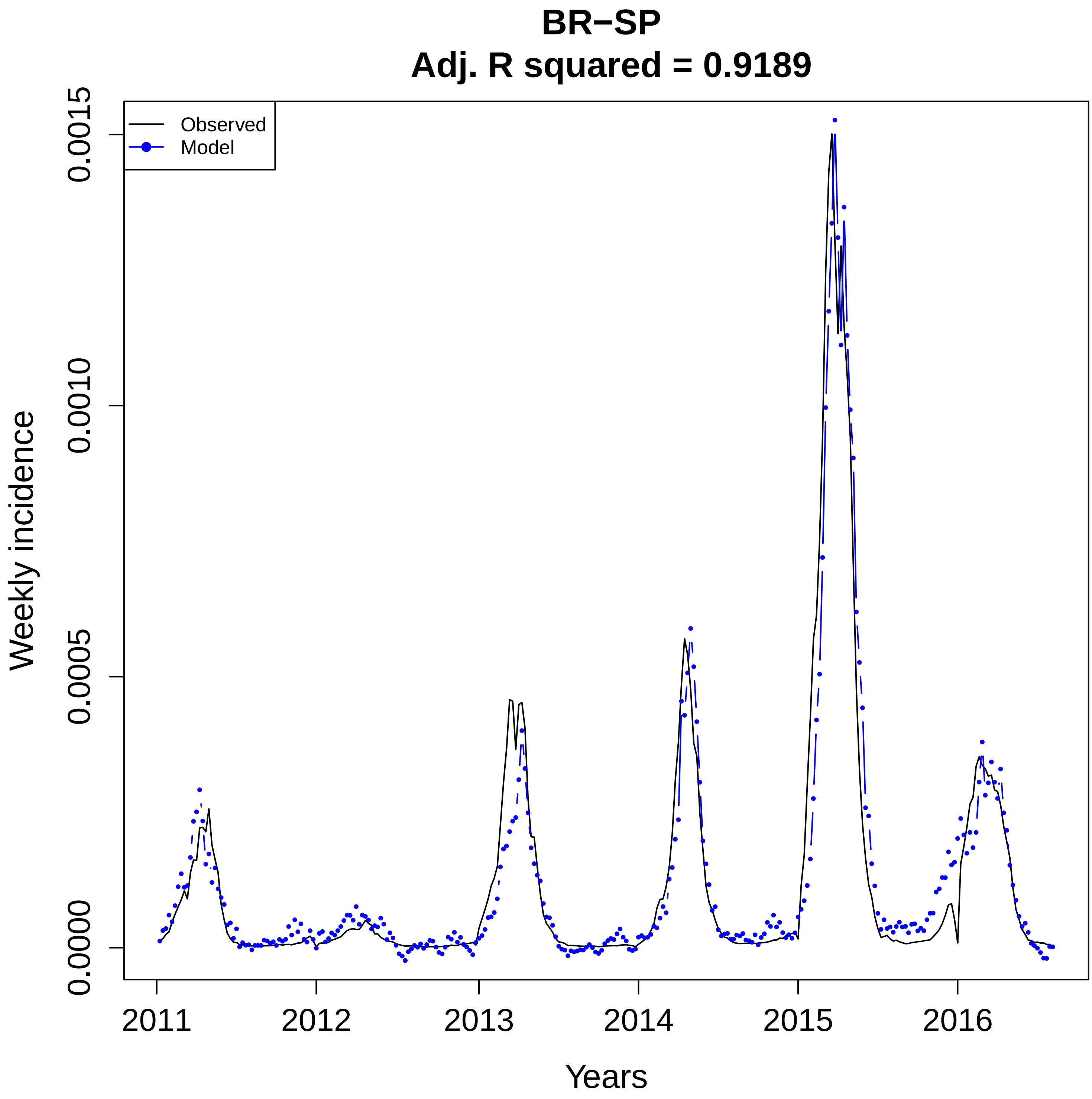

BR−SP
Adj. R squared = 0.9189
Observed
Model
Weekly incidence
0.0000
0.0005
0.0010
0.0015
2011
2012
2013
2014
2015
2016
Years

Uncorrelated (n = 6)

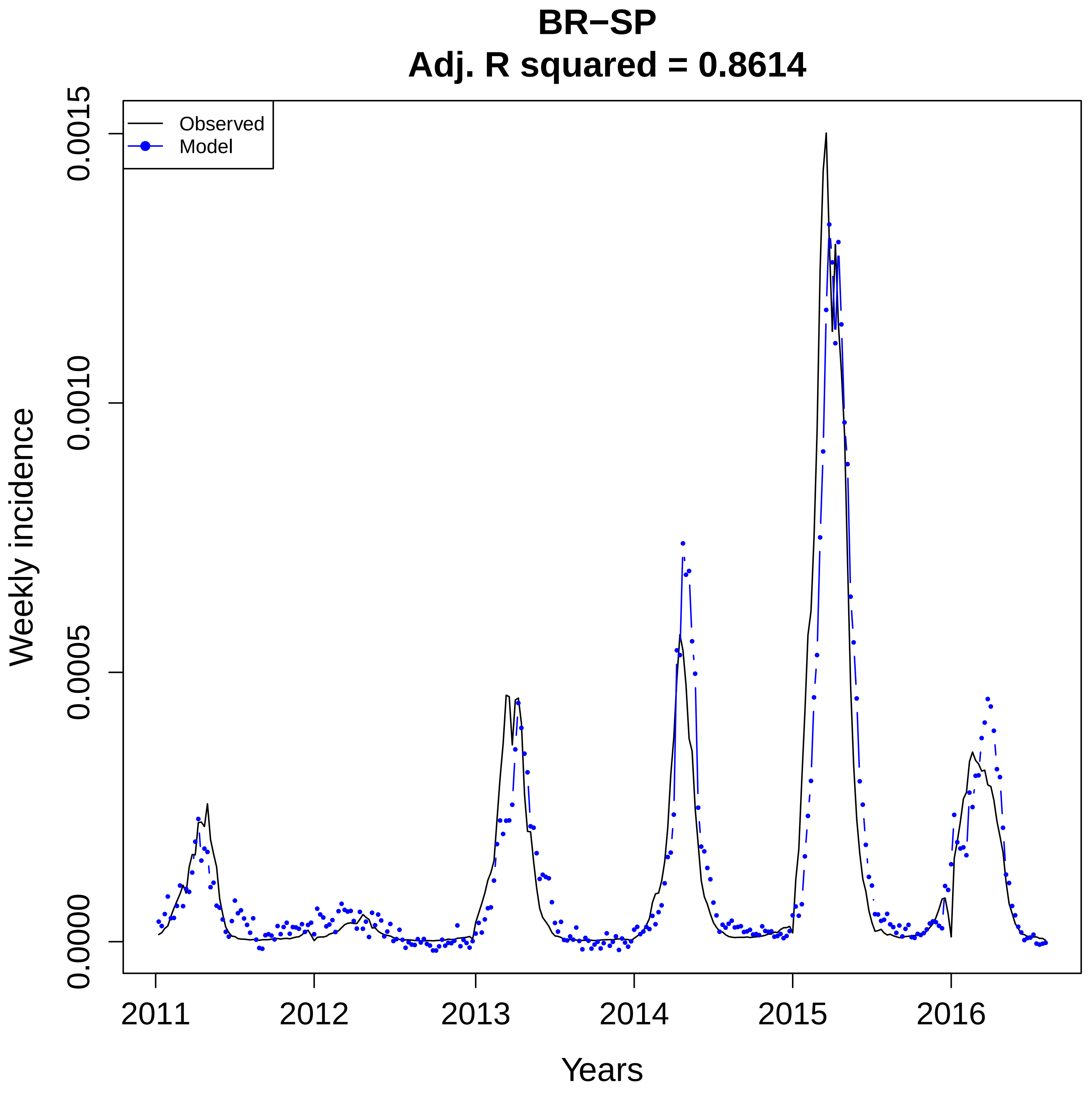

Individual terms
Adjusted R squared

São Paulo (SP)

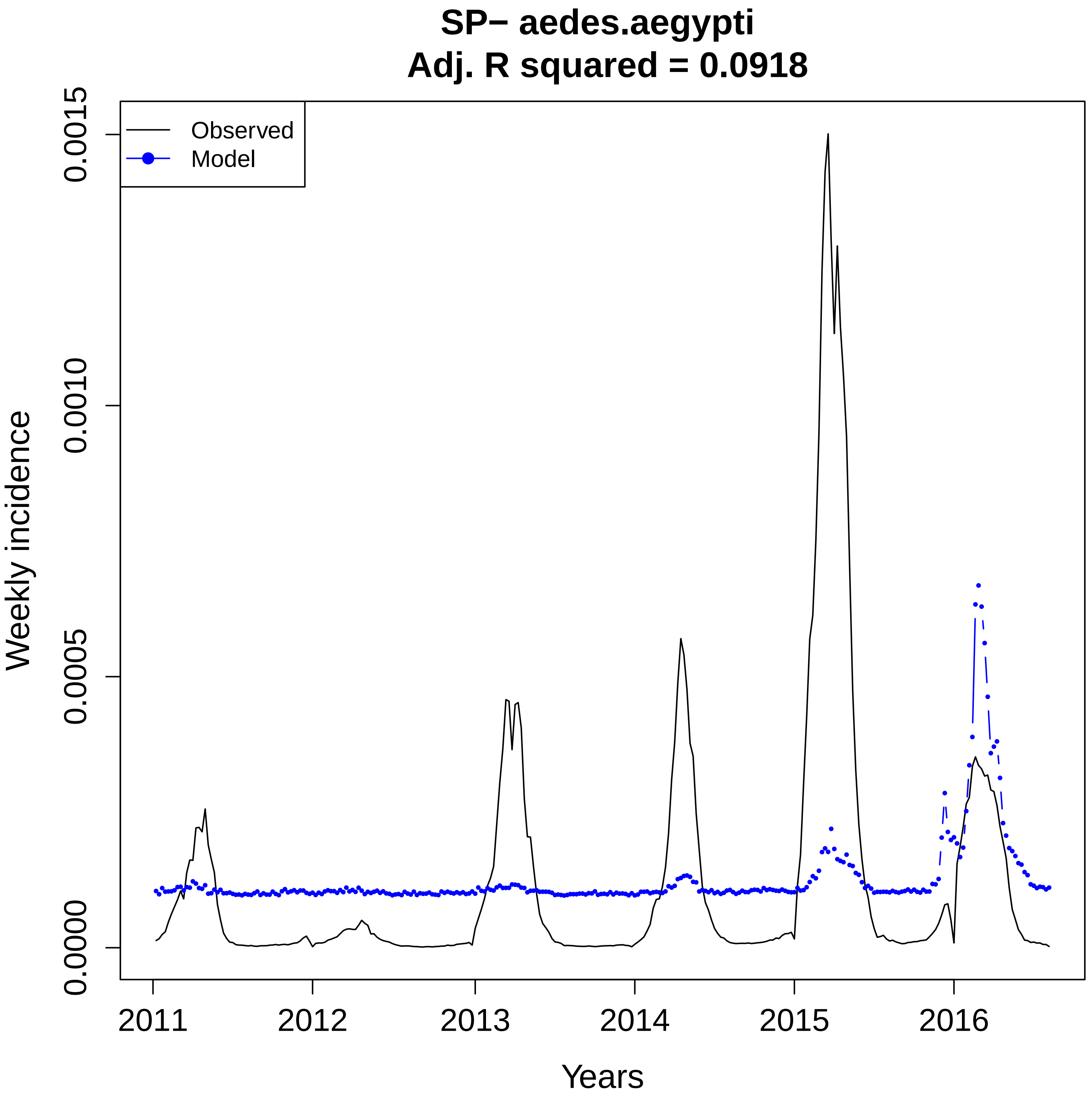
SP− aedes.aegypti
Adj. R squared = 0.0918
Observed
Model
Weekly incidence
0.0000
0.0005
0.0010
0.0015
2011
2012
2013
2014
2015
2016
Years

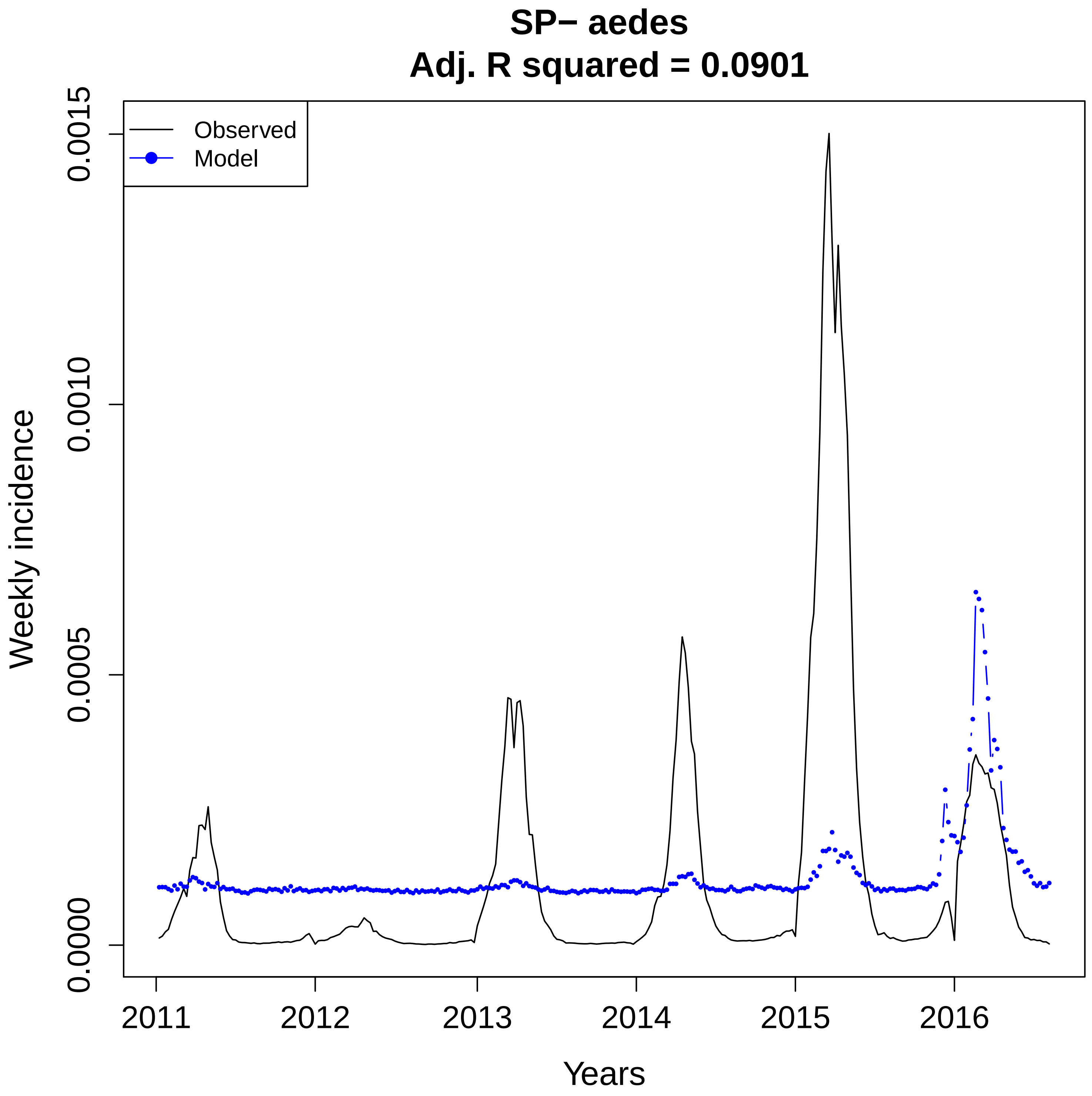

SP− aedes
Adj. R squared = 0.0901
Observed
Model
Weekly incidence
0.0000
0.0005
0.0010
0.0015
2011
2012
2013
2014
2015
2016
Years

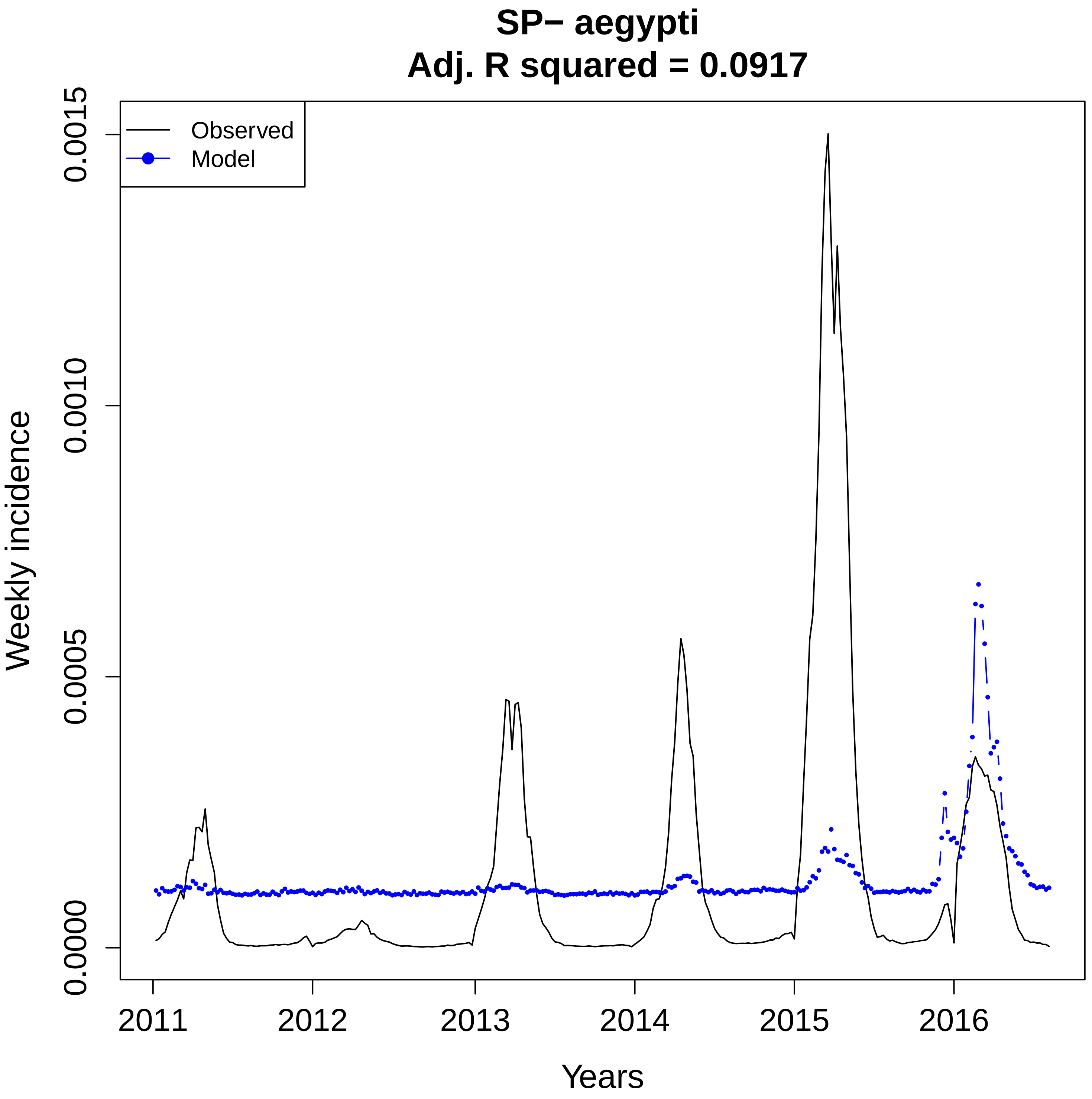
SP− aegypti
Adj. R squared = 0.0917
Observed
Model
Weekly incidence
0.0000
0.0005
0.0010
0.0015
2011
2012
2013
2014
2015
2016
Years

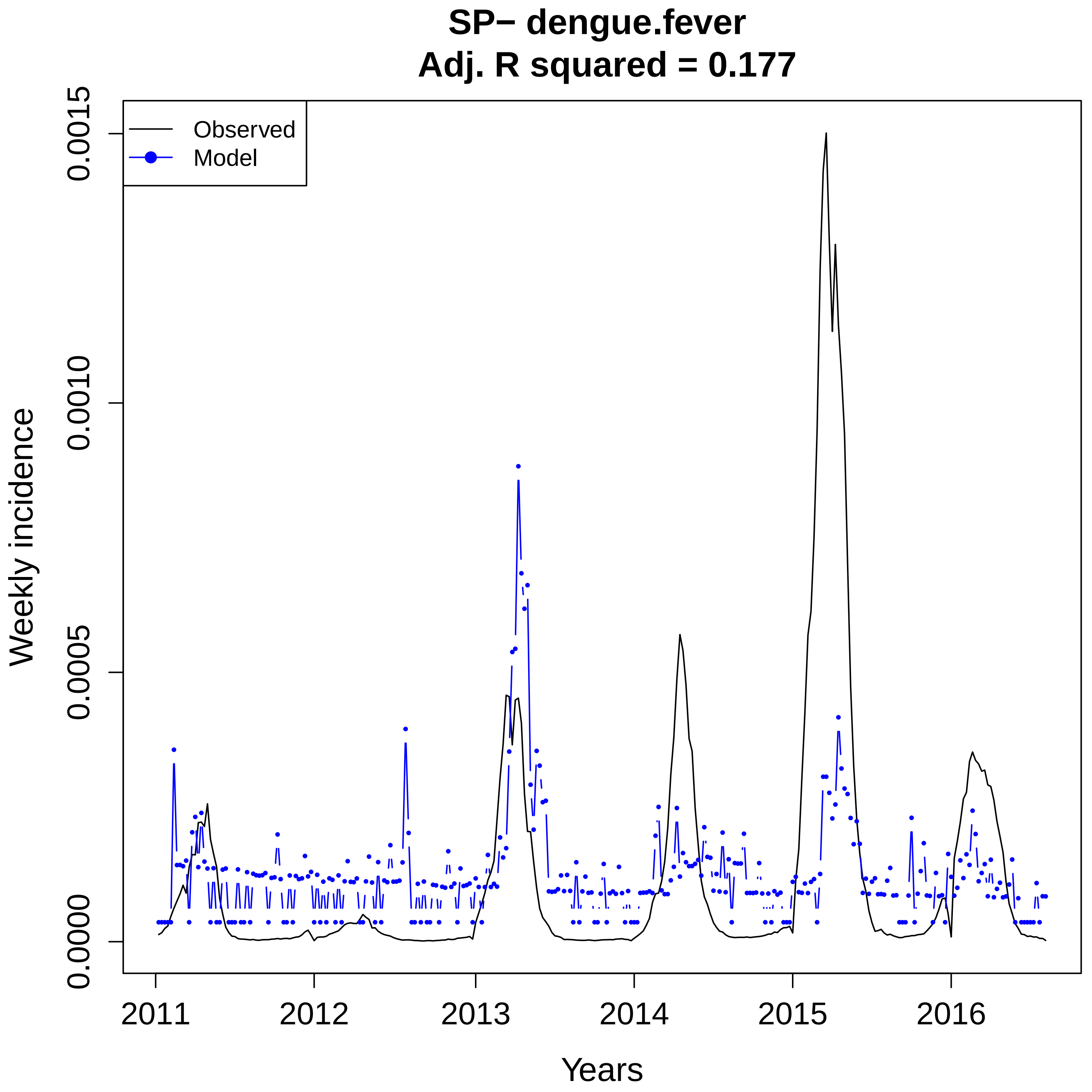

SP− dengue.fever
Adj. R squared = 0.177
Observed
Model
Weekly incidence
0.0000
0.0005
0.0010
0.0015
2011
2012
2013
2014
2015
2016
Years

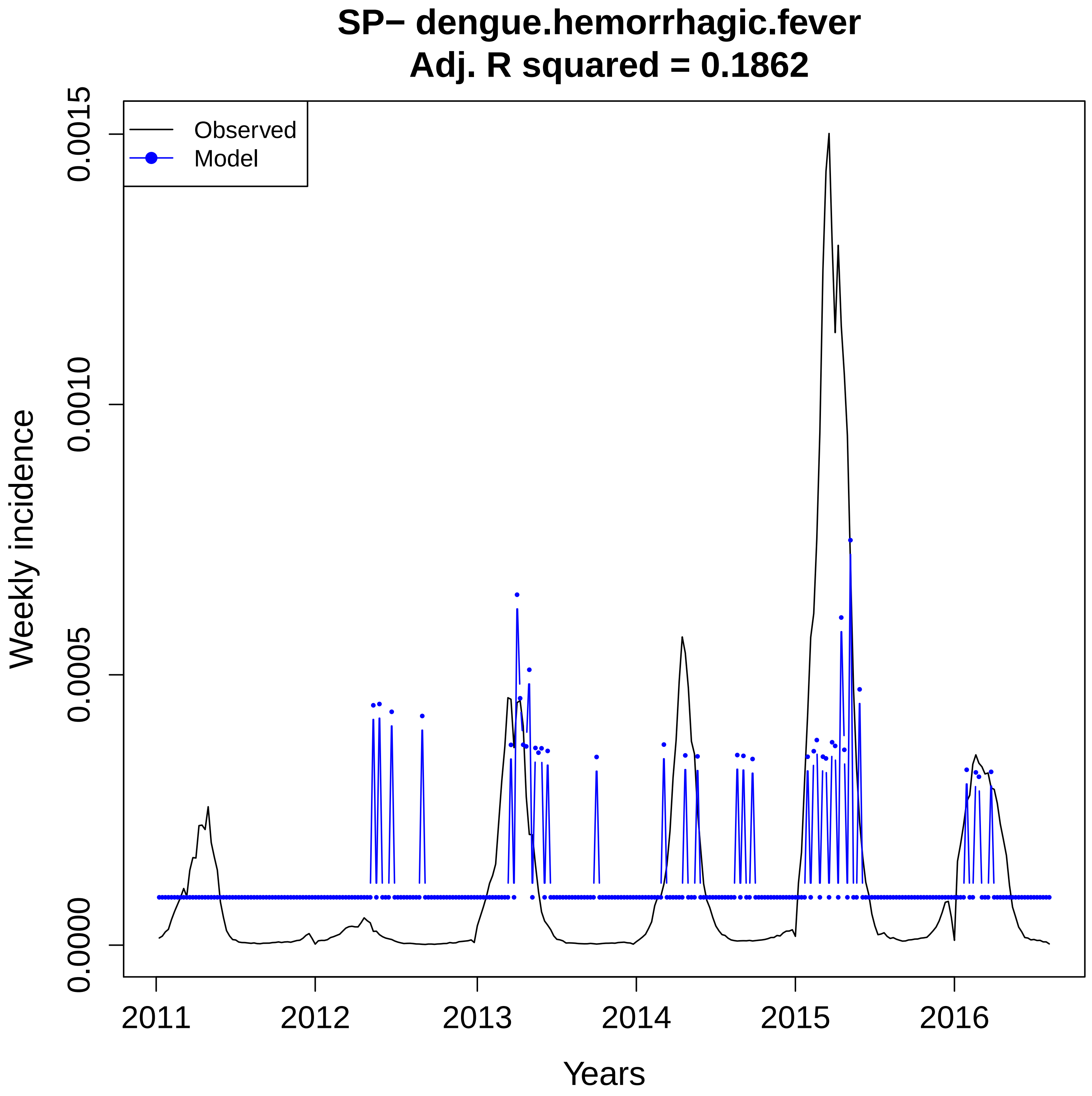
SP− dengue.hemorrhagic.fever
Adj. R squared = 0.1862
Observed
Model
Weekly incidence
0.0000
0.0005
0.0010
0.0015
2011
2012
2013
2014
2015
2016
Years

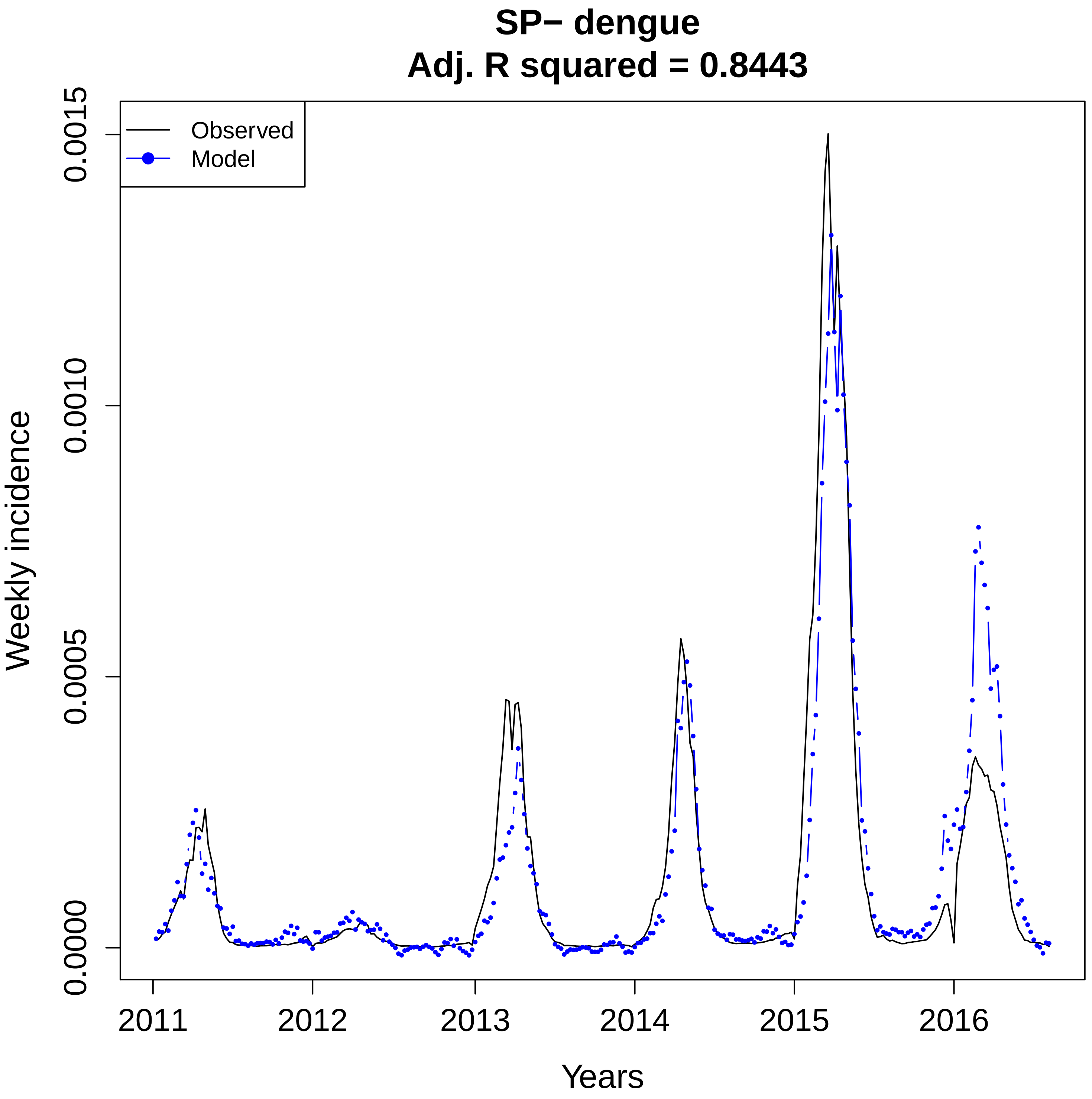
SP− dengue
Adj. R squared = 0.8443
Observed
Model
Weekly incidence
0.0000
0.0005
0.0010
0.0015
2011
2012
2013
2014
2015
2016
Years

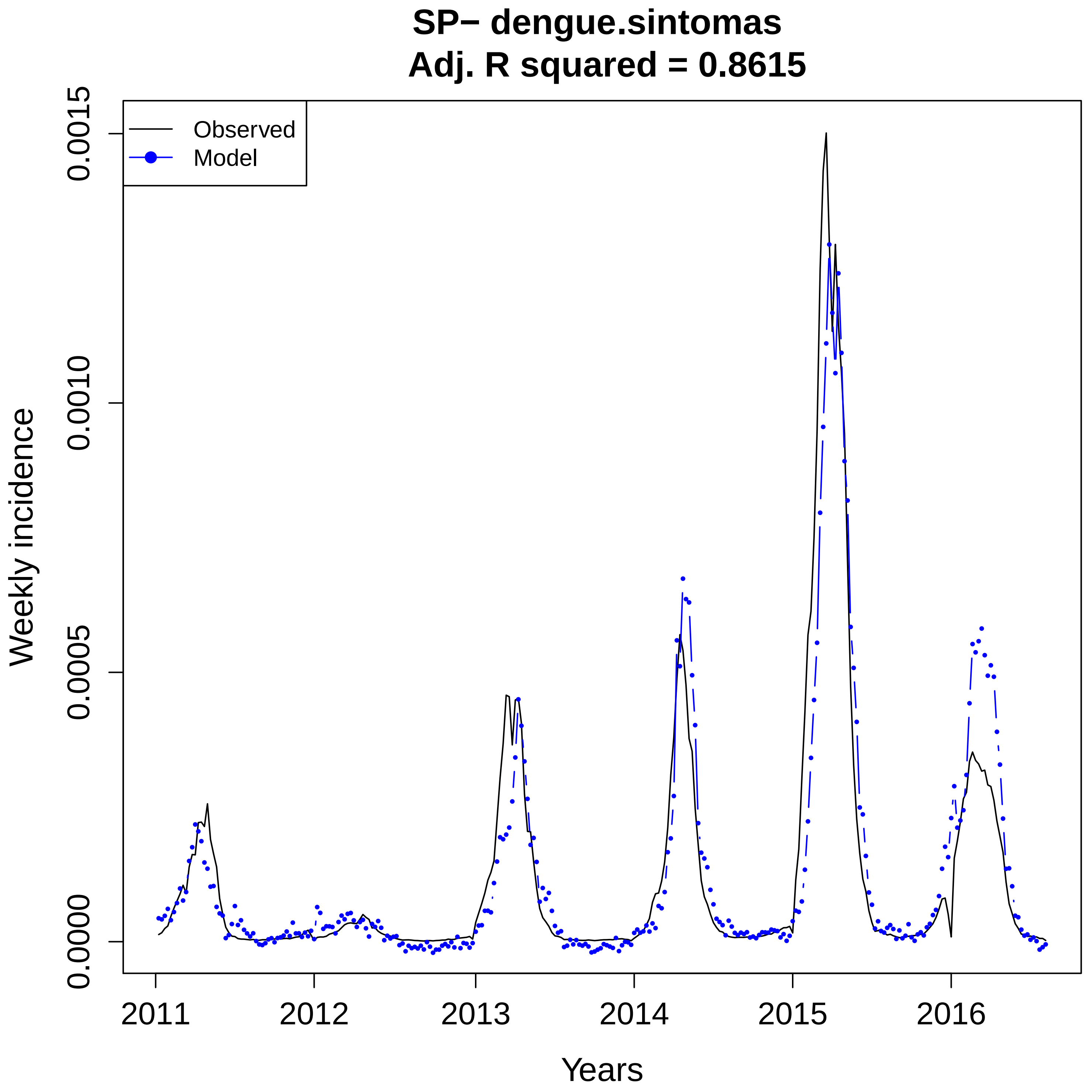
SP− dengue.sintomas
Adj. R squared = 0.8615
Observed
Model
Weekly incidence
0.0000
0.0005
0.0010
0.0015
2011
2012
2013
2014
2015
2016
Years

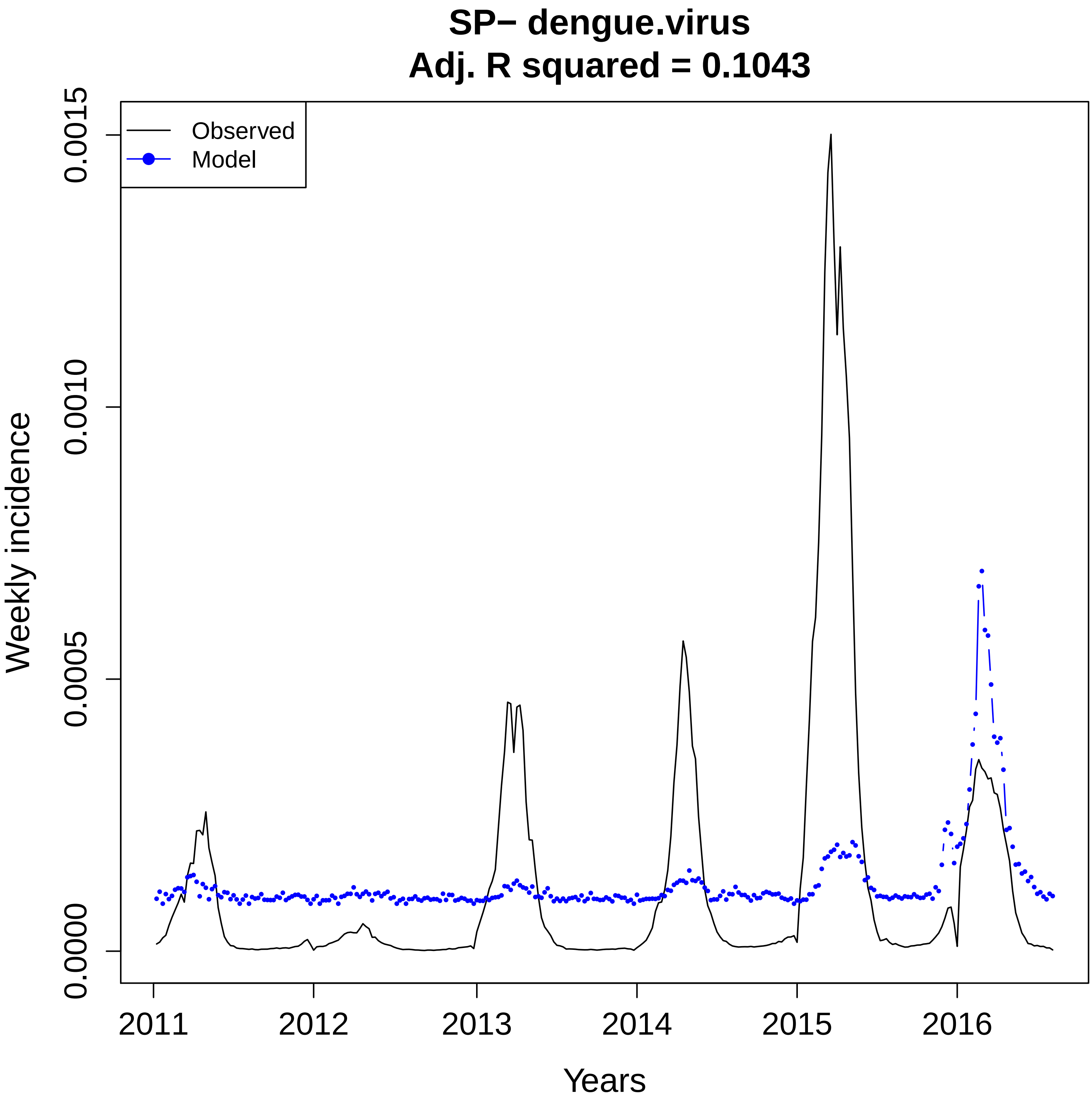
SP– dengue.virus
Adj. R squared = 0.1043
Observed
Model
Weekly incidence
0.0000
0.0005
0.0010
0.0015
2011
2012
2013
2014
2015
2016
Years

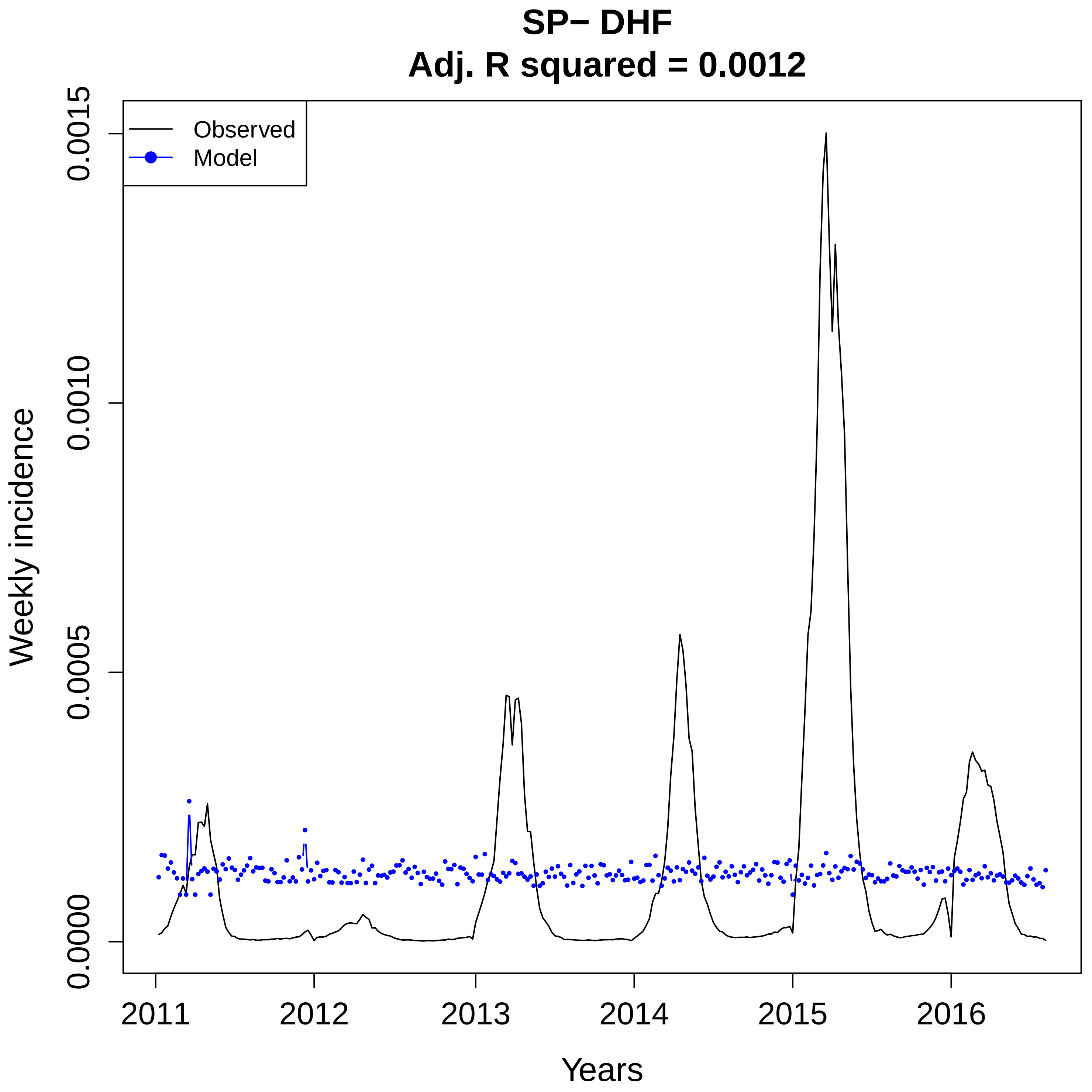
SP− DHF
Adj. R squared = 0.0012
Observed
Model
Weekly incidence
0.0000
0.0005
0.0010
0.0015
2011
2012
2013
2014
2015
2016
Years

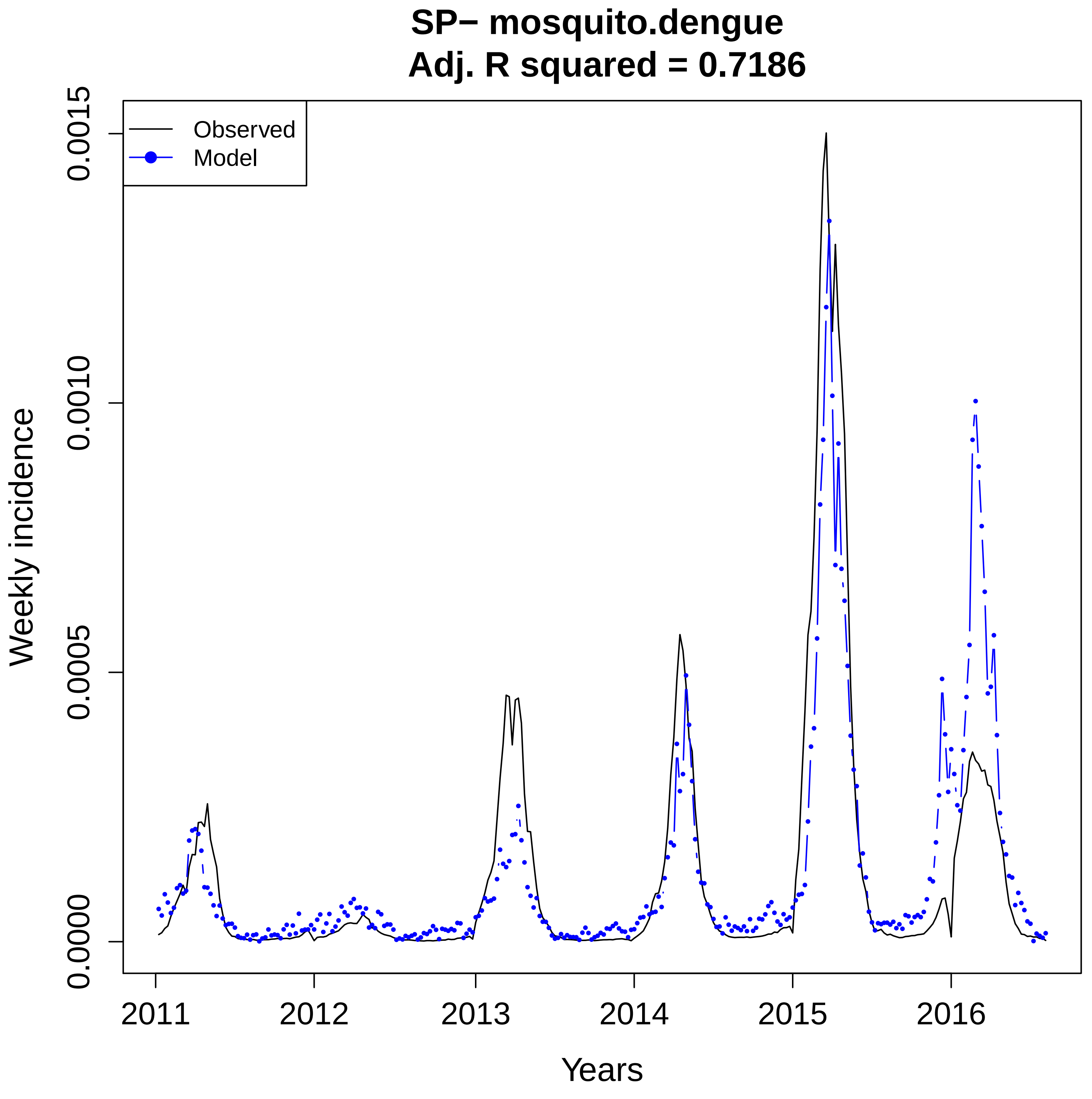

SP− mosquito.dengue
Adj. R squared = 0.7186
Observed
Model
Weekly incidence
0.0000
0.0005
0.0010
0.0015
2011
2012
2013
2014
2015
2016
Years

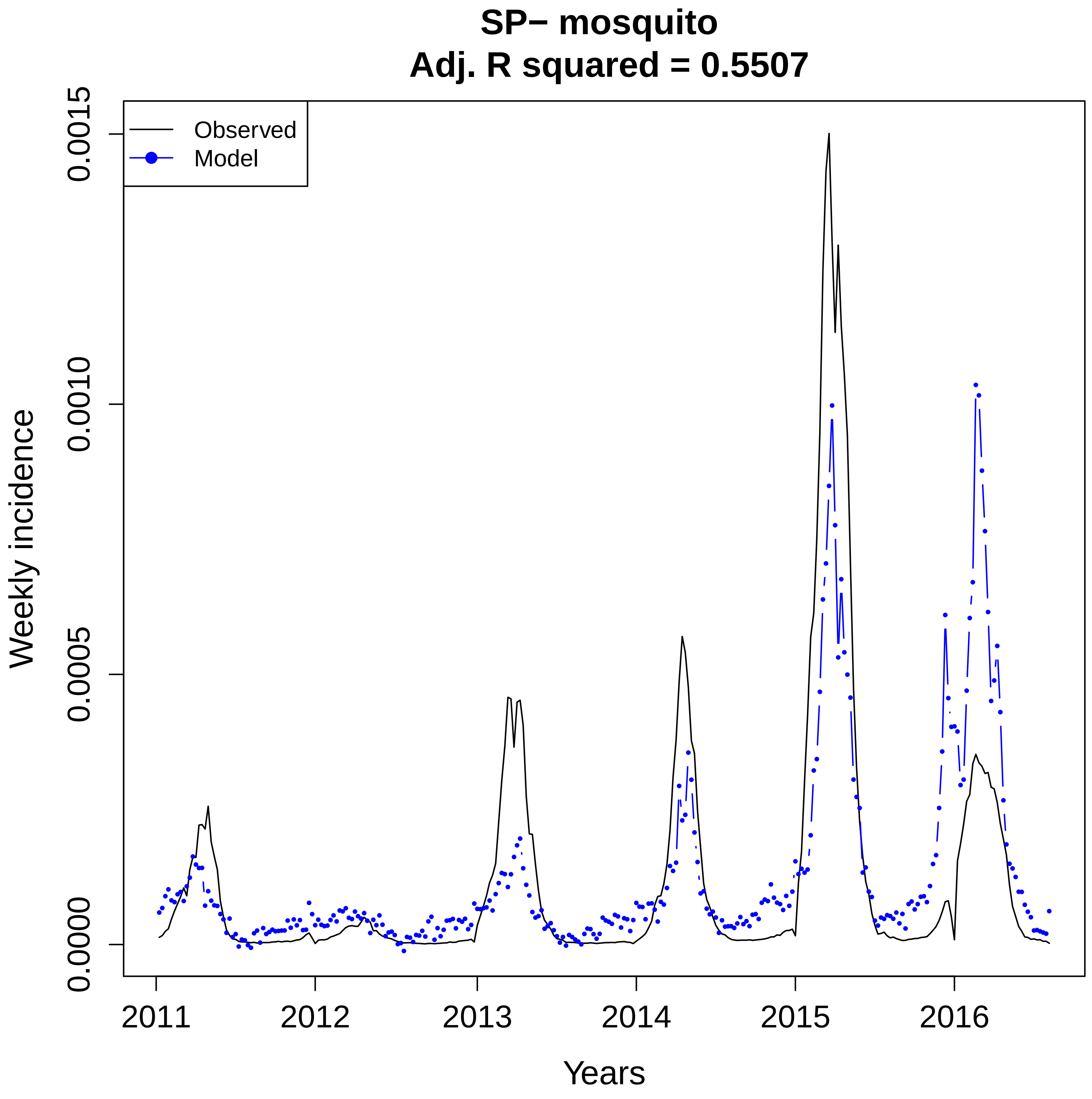

SP− mosquito
Adj. R squared = 0.5507
Observed
Model
Weekly incidence
0.0000
0.0005
0.0010
0.0015
2011
2012
2013
2014
2015
2016
Years

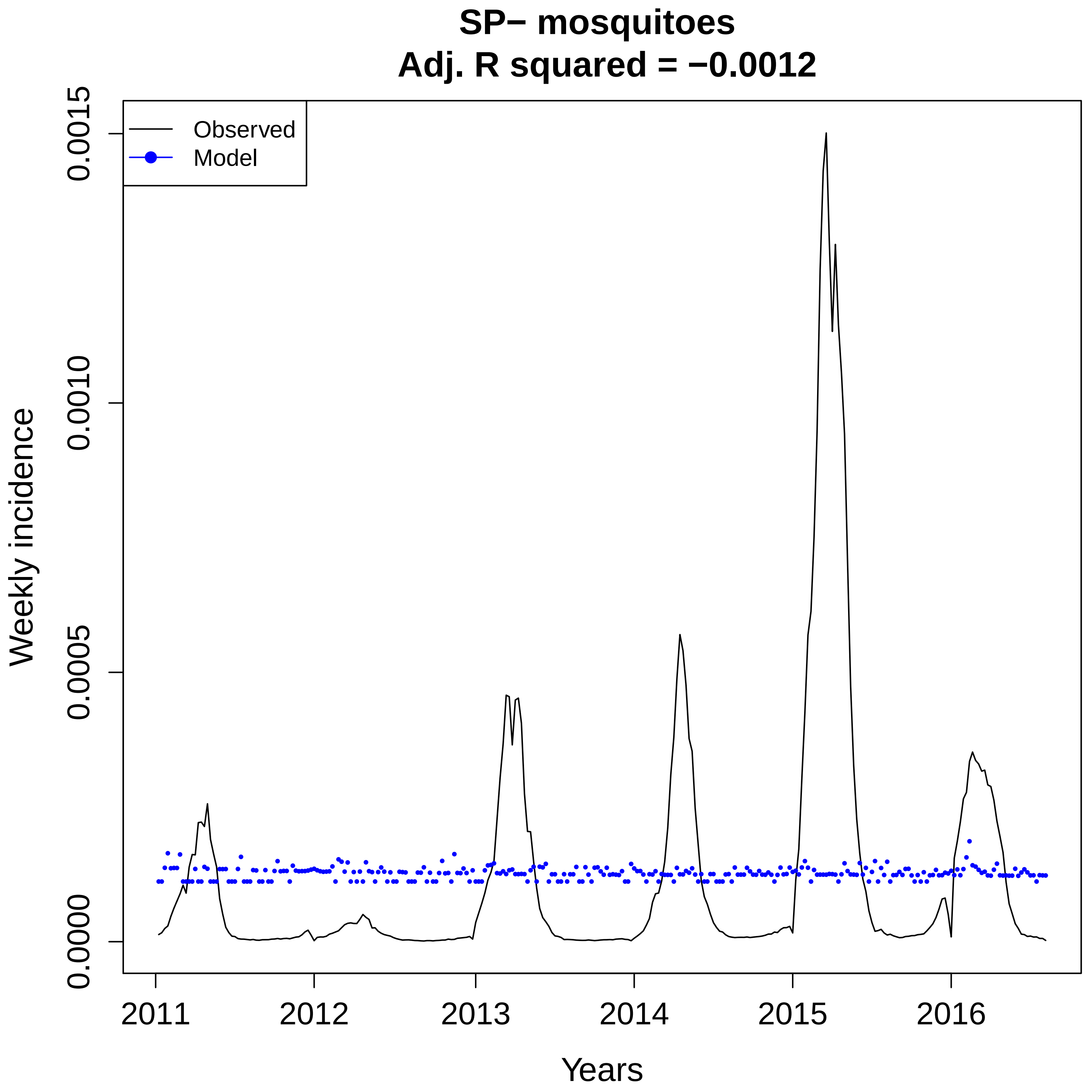
SP− mosquitoes
Adj. R squared = −0.0012
Observed
Model
Weekly incidence
0.0000
0.0005
0.0010
0.0015
2011
2012
2013
2014
2015
2016
Years

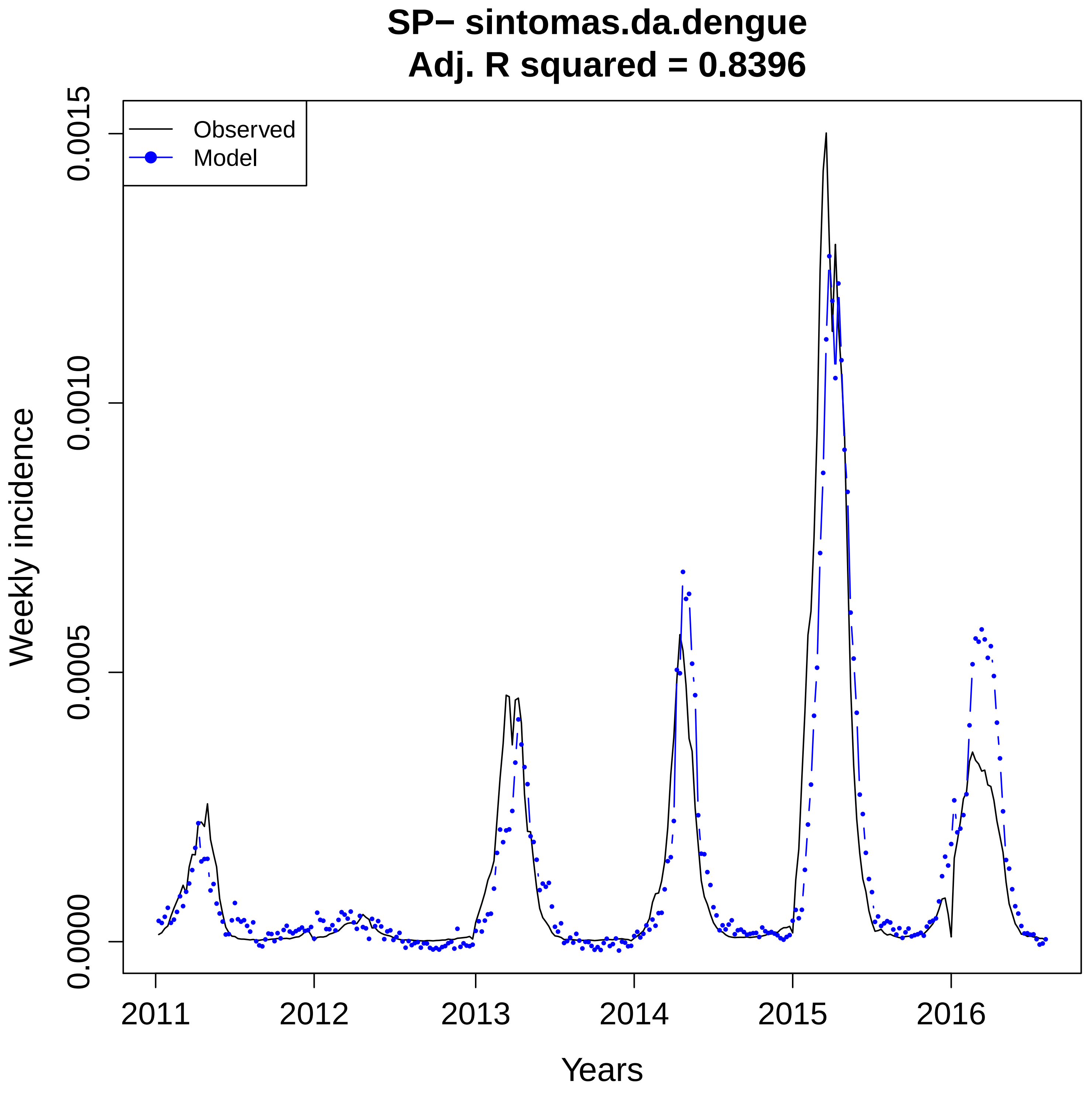

SP− sintomas.da.dengue
Adj. R squared = 0.8396
Observed
Model
Weekly incidence
0.0000
0.0005
0.0010
0.0015
2011
2012
2013
2014
2015
2016
Years

Tocantins (TO)

Dengue incidence vs. Google Health Trends (2011–2016)

Definitions:

- **Combined terms:**
  - **All:** model with all the available terms. n= number of terms
  - **Four:** model with "dengue", "dengue sintomas", "aedes", and "mosquito".
  - **Uncorrelated:** model with non-correlated terms. n = number of uncorrelated terms
- **Individual terms:** Plots developed with one term at a time.

- Terms for all and uncorrelated models can be found in additional file 3.

Combined terms
Adjusted R squared

Tocantins (TO)

All
(n = 8)

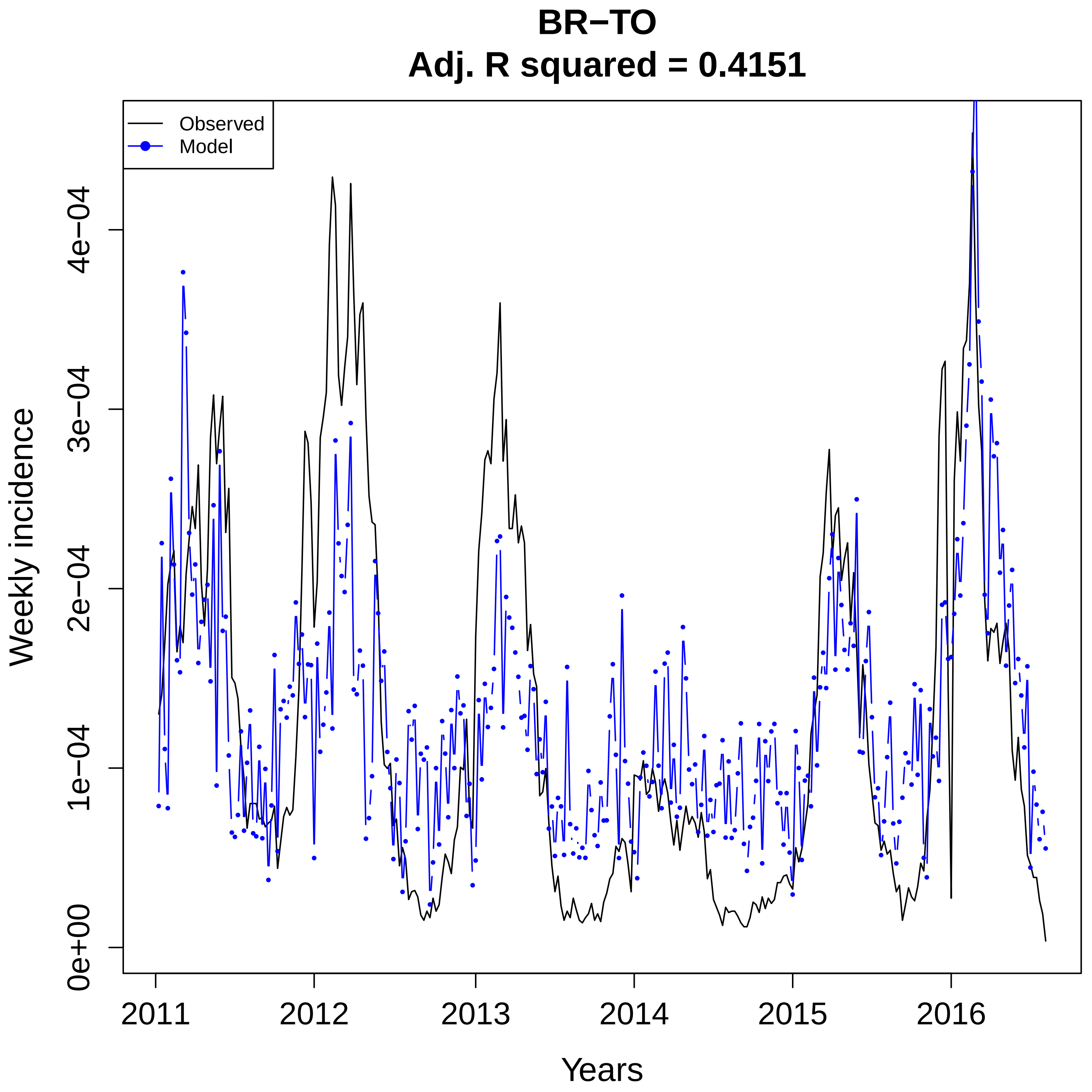

BR−TO
Adj. R squared = 0.4151
Observed
Model
Weekly incidence
0e+00
1e−04
2e−04
3e−04
4e−04
2011
2012
2013
2014
2015
2016
Years

Four

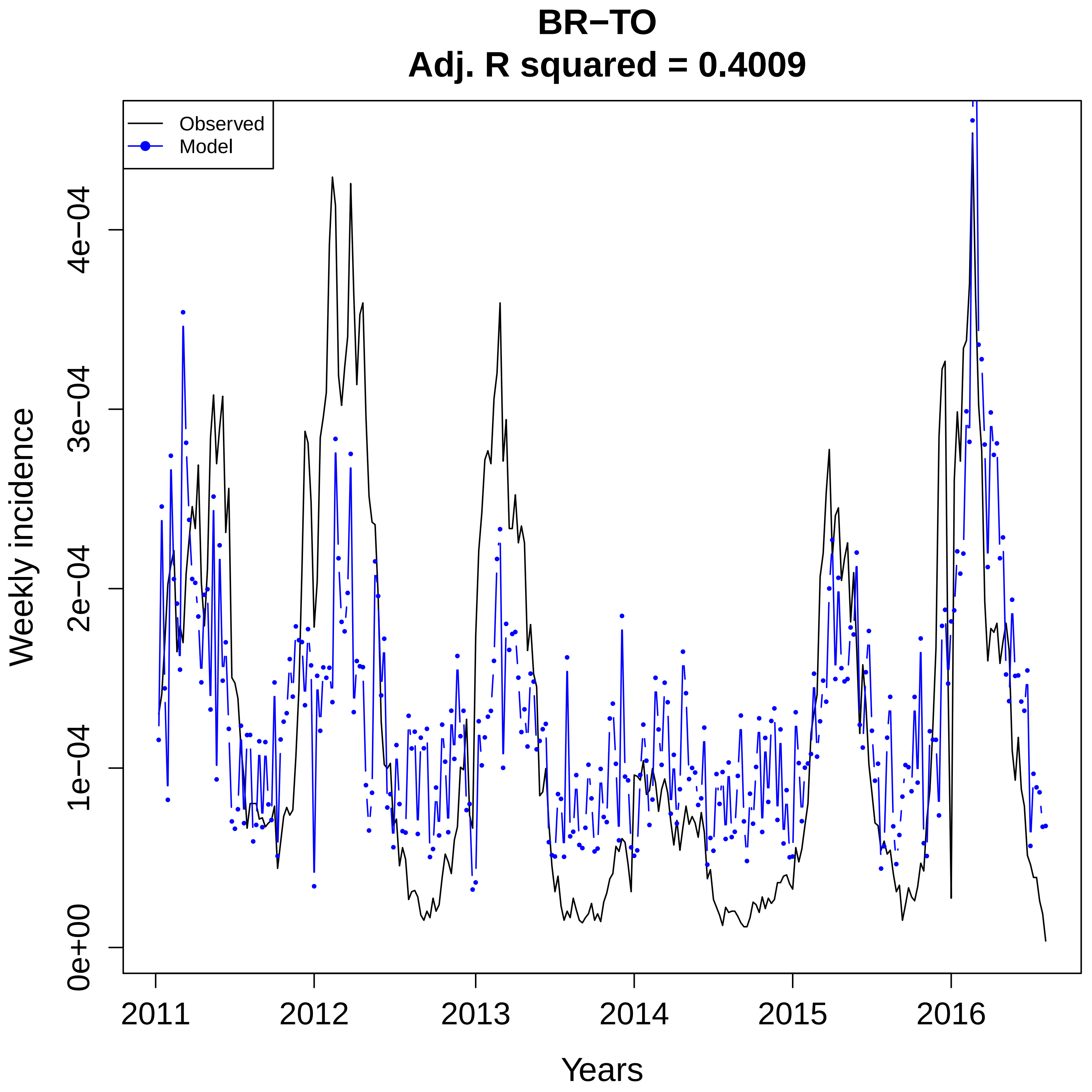

BR−TO
Adj. R squared = 0.4009
Observed
Model
Weekly incidence
0e+00
1e−04
2e−04
3e−04
4e−04
2011
2012
2013
2014
2015
2016
Years

Uncorrelated
(n = 3)

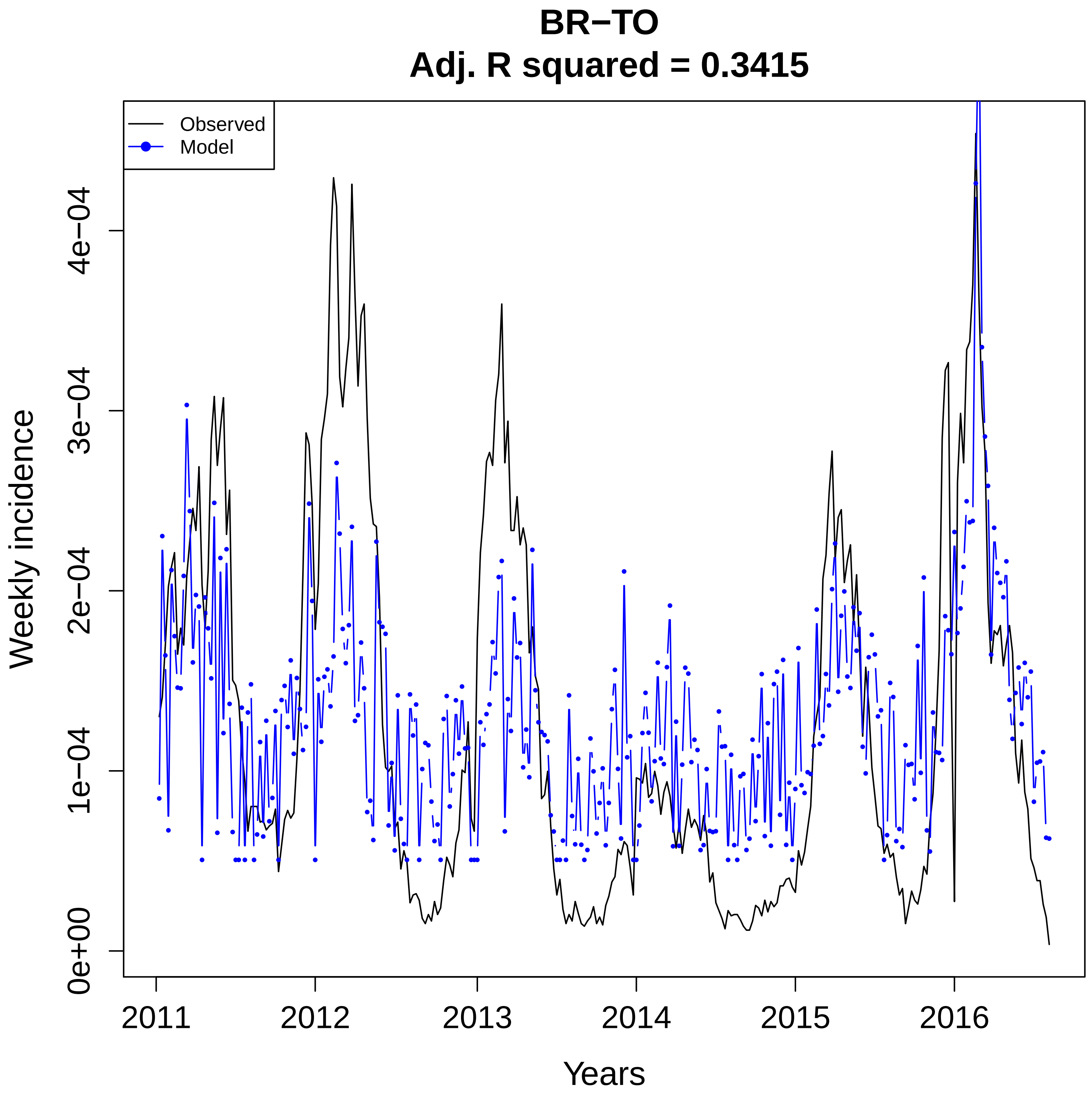

Individual terms
Adjusted R squared

Tocantins (TO)

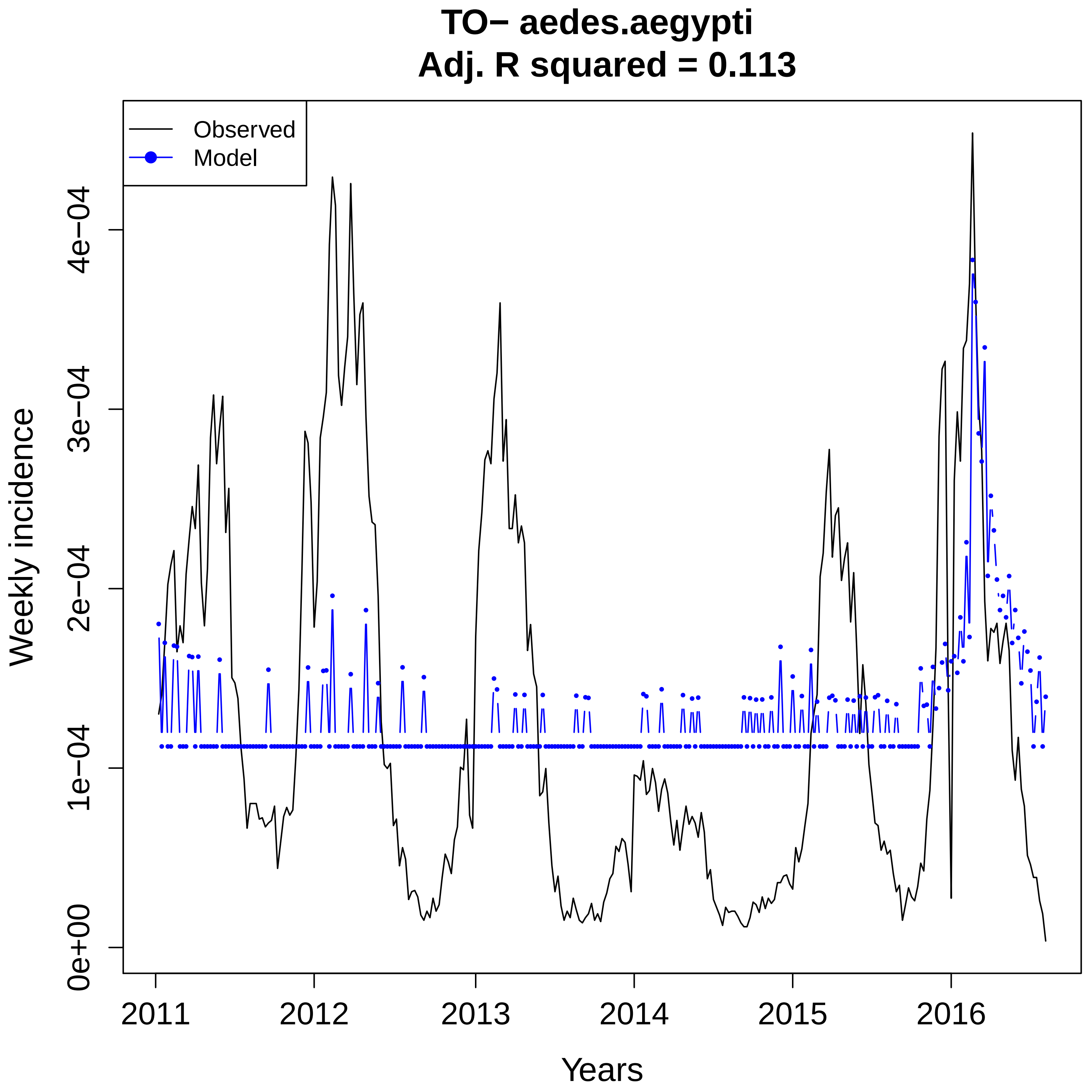
TO− aedes.aegypti
Adj. R squared = 0.113
Observed
Model
Weekly incidence
0e+00
1e−04
2e−04
3e−04
4e−04
2011
2012
2013
2014
2015
2016
Years

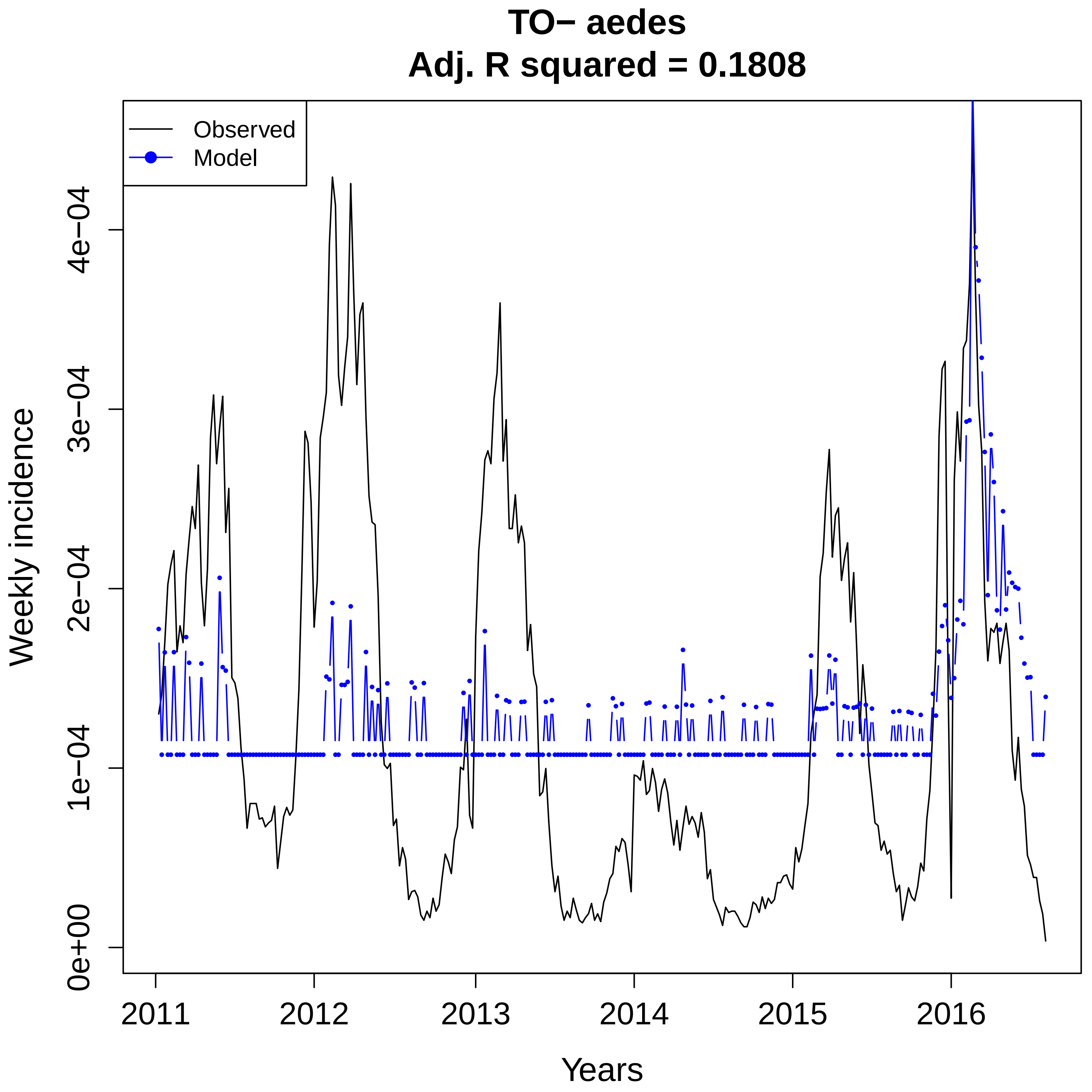
TO− aedes
Adj. R squared = 0.1808
Observed
Model
Weekly incidence
0e+00
1e−04
2e−04
3e−04
4e−04
2011
2012
2013
2014
2015
2016
Years

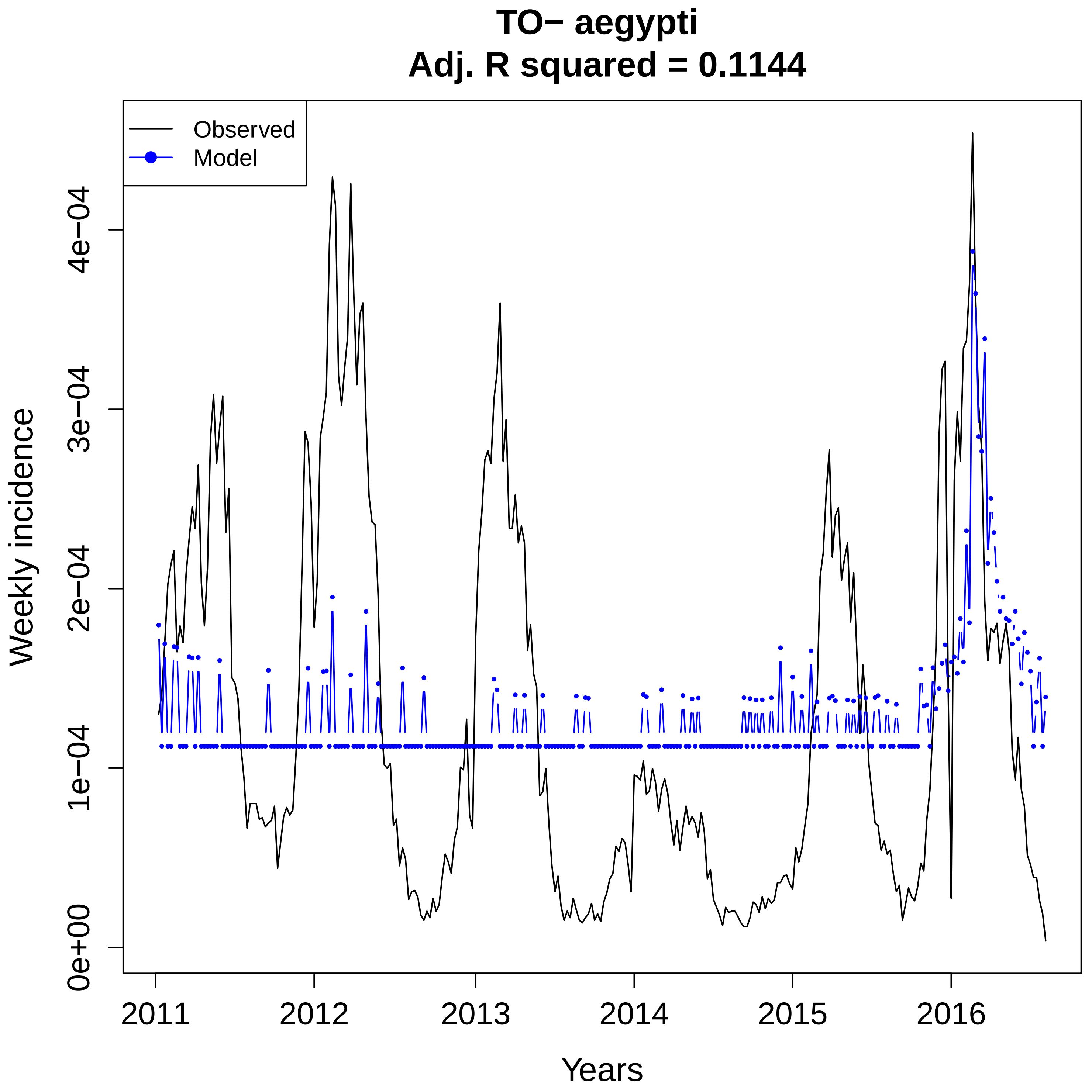

TO− aegypti
Adj. R squared = 0.1144
Observed
Model
Weekly incidence
0e+00
1e−04
2e−04
3e−04
4e−04
2011
2012
2013
2014
2015
2016
Years

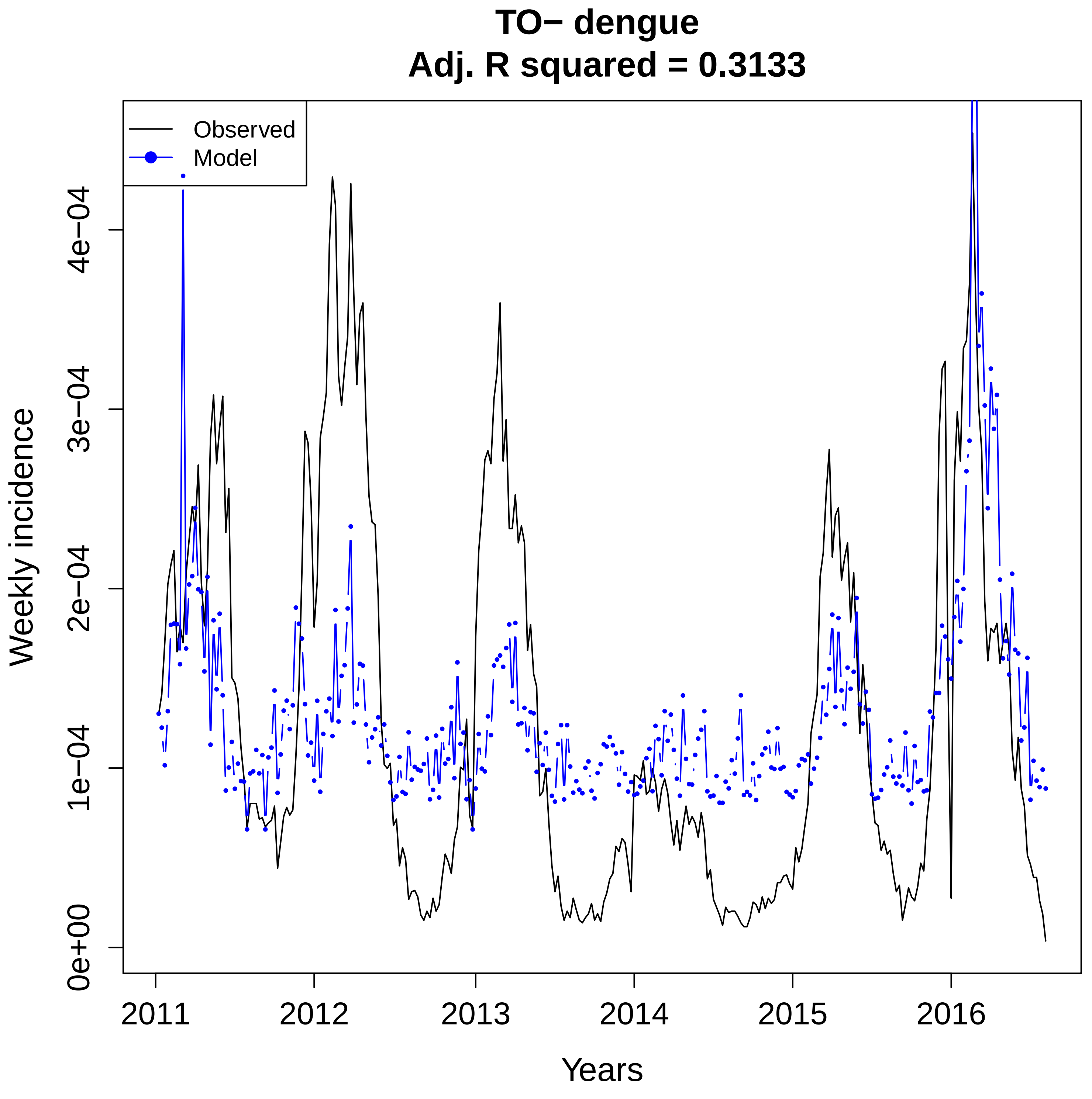

TO− dengue
Adj. R squared = 0.3133
Observed
Model
Weekly incidence
0e+00
1e−04
2e−04
3e−04
4e−04
2011
2012
2013
2014
2015
2016
Years

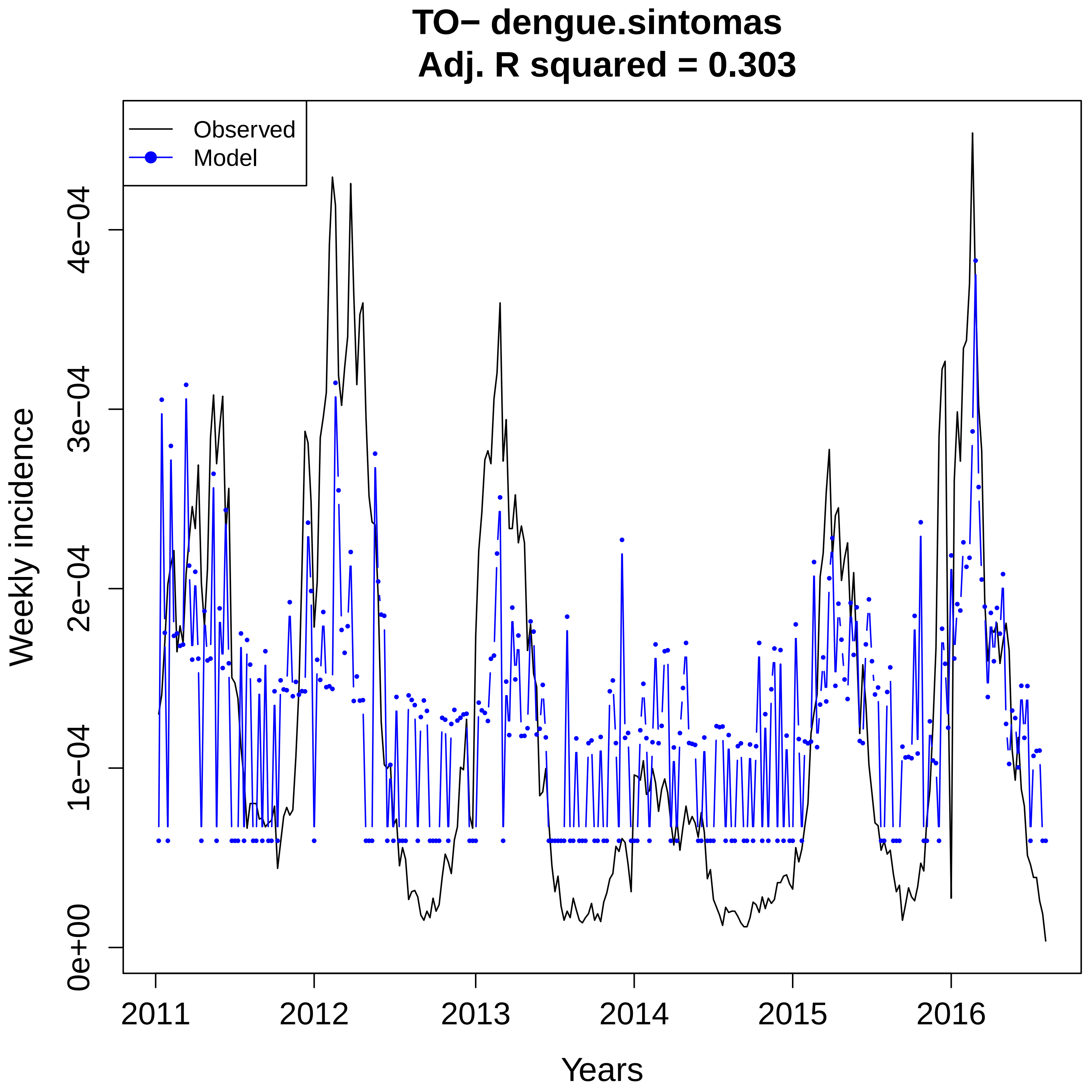
TO− dengue.sintomas
Adj. R squared = 0.303
Observed
Model
Weekly incidence
0e+00
1e−04
2e−04
3e−04
4e−04
2011
2012
2013
2014
2015
2016
Years

TO− mosquito.dengue
Adj. R squared = 0.15

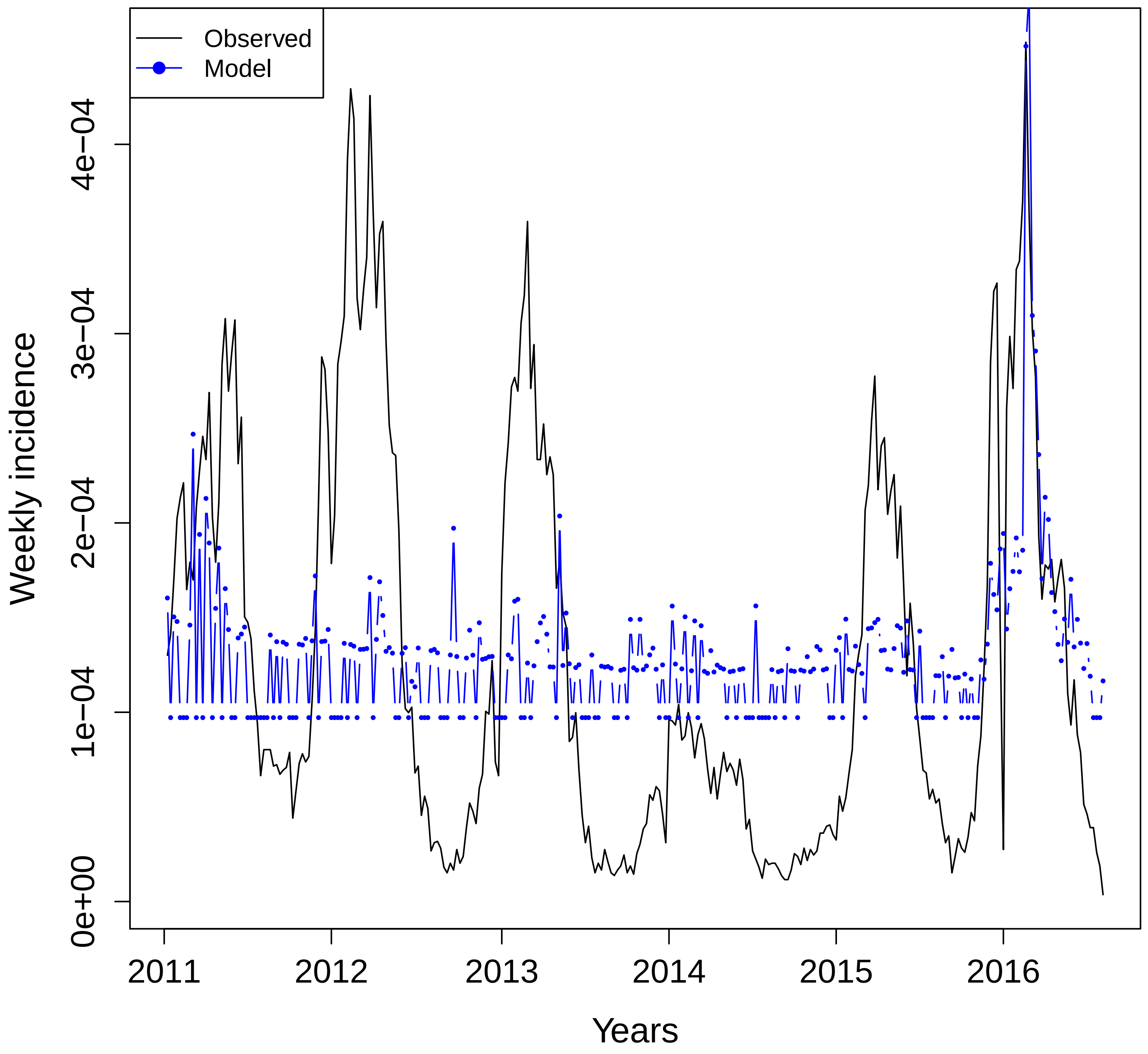

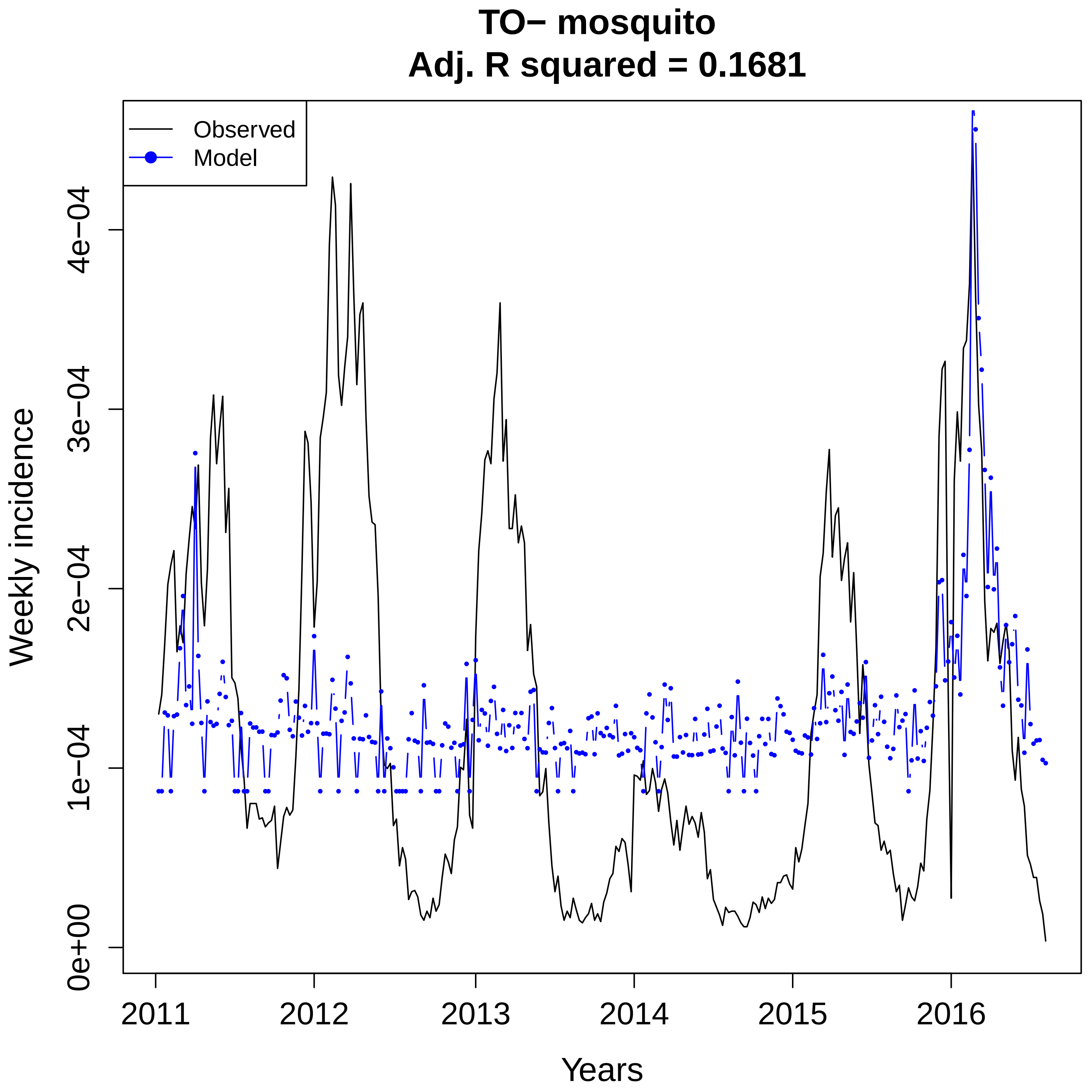
TO− mosquito
Adj. R squared = 0.1681
Observed
Model
Weekly incidence
0e+00
1e−04
2e−04
3e−04
4e−04
Years
2011
2012
2013
2014
2015
2016

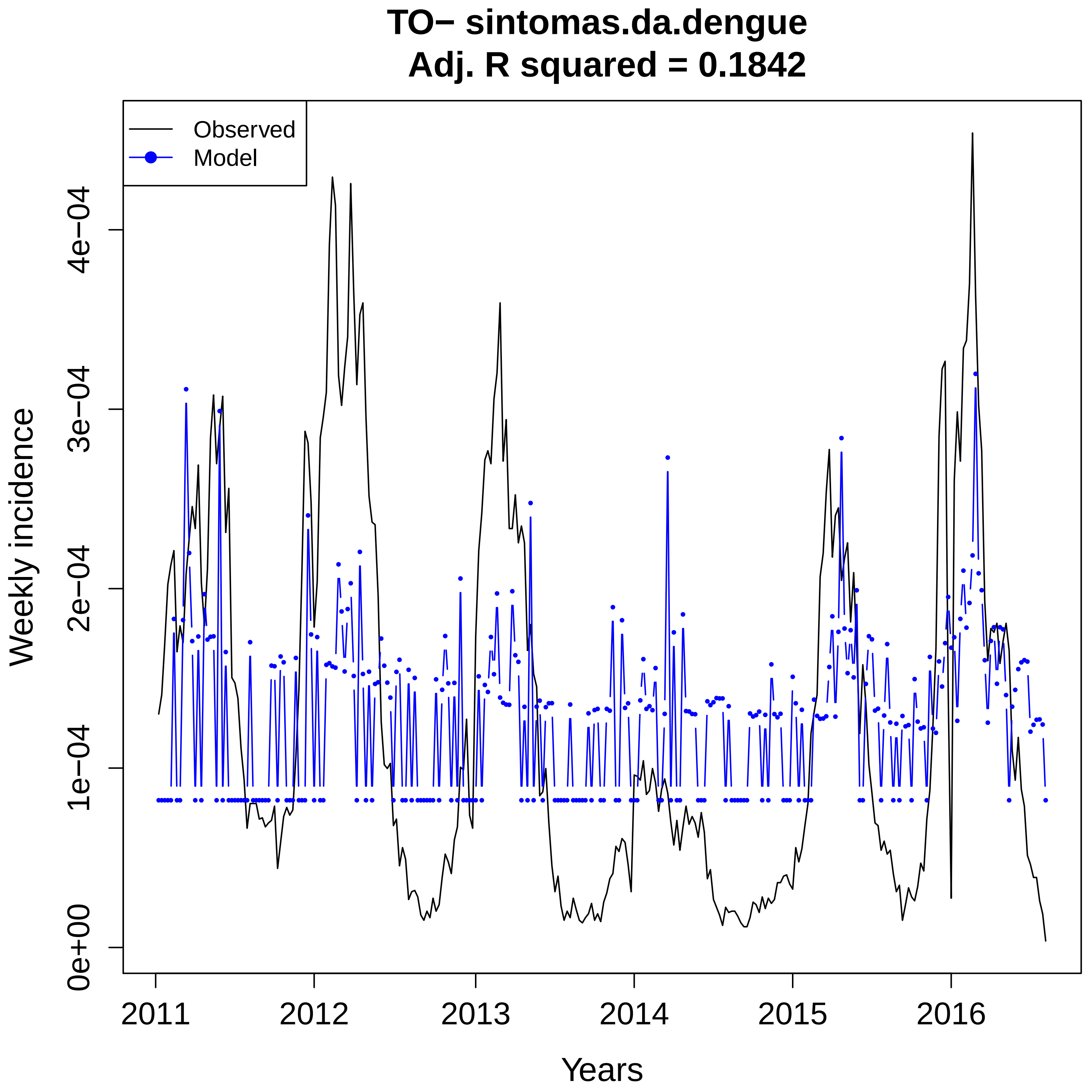
TO− sintomas.da.dengue
Adj. R squared = 0.1842
Observed
Model
Weekly incidence
0e+00
1e−04
2e−04
3e−04
4e−04
2011
2012
2013
2014
2015
2016
Years
