# Supplementary material for: Google Health Trends performance reflecting dengue incidence for the Brazilian states
Source: BMC Infect Dis. 2020 Mar 26;20:252. doi: 10.1186/s12879-020-04957-0 (PMC7104526; doi:10.1186/s12879-020-04957-0)
Supplement: Supplementary file 6 — Additional file 6. Correlation plot of the Internet access variables selected in the study. [file 12879_2020_4957_MOESM6_ESM.docx]

**Google Health Trends performance reflecting dengue incidence for the Brazilian states**

**Authors:** Daniel Romero-Alvarez, Nidhi Parikh, Dave Osthus, Kaitlyn Martinez, Nicholas Generous, Sara del Valle, Carrie A. Manore

**Additional file 6. Correlation plot between Internet variables.** V1: People above 10 years old using Internet in the last three months; V2: Logarithm of V1; V3: Number of people owing a mobile phone; V4: Logarithm V3; V5: Households using Internet in the last three months; V6: Logarithm V5; V7: Number of households with computers; V8: Logarithm of V7.
