# Supplementary material for: Google Health Trends performance reflecting dengue incidence for the Brazilian states
Source: BMC Infect Dis. 2020 Mar 26;20:252. doi: 10.1186/s12879-020-04957-0 (PMC7104526; doi:10.1186/s12879-020-04957-0)
Supplement: Supplementary file 8 — Additional file 8. Results of the principal component analysis of 474 predictors. [file 12879_2020_4957_MOESM8_ESM.docx]

**Google Health Trends performance reflecting dengue incidence for the Brazilian states**

**Authors:** Daniel Romero-Alvarez, Nidhi Parikh, Dave Osthus, Kaitlyn Martinez, Nicholas Generous, Sara del Valle, Carrie A. Manore

**Additional file 8:** Principal Component Analysis (PCA) on 474 demographic predictors. The six first PCs recovered 0.912 of the variance.

| **Components selected** | **Estimates** | **Adj. R squared** |
| --- | --- | --- |
| PC1  PC2  PC3  PC4  PC5  PC6 | 0.009915  -0.010542  -0.007275  0.009705  -0.012579  -0.027384 | 0.6547 |
