# Supplementary material for: Google Health Trends performance reflecting dengue incidence for the Brazilian states
Source: BMC Infect Dis. 2020 Mar 26;20:252. doi: 10.1186/s12879-020-04957-0 (PMC7104526; doi:10.1186/s12879-020-04957-0)
Supplement: Supplementary file 9 — Additional file 9. Variables selected by applying different variable selection routines and definitions of each of the selected predictors. [file 12879_2020_4957_MOESM9_ESM.docx]

**Google Health Trends performance reflecting dengue incidence for the Brazilian states**

**Authors:** Daniel Romero-Alvarez, Nidhi Parikh, Dave Osthus, Kaitlyn Martinez, Nicholas Generous, Sara del Valle, Carrie A. Manore

**Additional file 9. Variable selection methods.**

From the 474 demographic variables, we used the following framework to select those most informative on when GHT might be useful. We explored 25 variables at a time (i.e., 27 states against 25 predictors) by means of (A) multiple linear regression models with uncorrelated predictors selected with a Pearson correlation threshold above 0.7, (B) a stepwise forward-backward selection among those uncorrelated to develop a model with the most informative variables, (C) using a direct stepwise forward-backward selection over the complete dataset, (D) using an elastic net regression with an L1 normalization (i.e., Least Absolute Shrinkage and Selection Operator or Lasso) in a leave-one-out cross validation fashion, and (E) using a principal component analysis (PCA) and selecting those components recovering more than 90% of the variance. For stepwise approaches we selected the best model as the one with the lowest Akaike Information Criterion (AIC) and for Lasso regression, we selected models with the least lambda parameter. In all cases, we recorded the adjusted R squared statistic, the most informative predictors, and compared the predicted R squared against the observed between GHT and dengue cases.

Brazilian states: AC: Acré, AL: Alagoas, AP: Amapá, AM: Amazonas, BA: Bahia, CE: Ceará, DF (arrow): Distrito Federal, ES: Espírito Santo, GO: Goiás, MA: Maranhão, MT: Mato Grosso, MS: Mato Grosso do Sul, MG: Minas Gerais, PA: Pará, PB: Paraiba, PR: Paraná, PE: Pernambuco, PI: Piauí, RJ: Rio de Janeiro, RN: Rio Grande do Norte, RS: Rio Grande do Sul, RO: Rondônia, RR: Roraima, SC: Santa Catarina, SP: São Paulo, SE: Sergipe, TO: Tocantins.

| **Variable selection method** | **Variables selected** | **Official Brazilian header** | **Estimates** | **Adj. R squared** |
| --- | --- | --- | --- | --- |
| Pearson’ s correlation | 17 | T_ATRASO_1_BASICO  T_FLBAS  CPR  P_COM  P_EXTR  P_SIUP  TRABPUB  pesoRUR  SOBRE60 (log)  T_FBBAS (log)  T_FREQ15A17(log)  T_FREQ25A29 (log)  T_FREQ5A6 (log)  P_CONSTR (log)  P_TRANSF (log)  T_DES1517 (log)  T_LIXO (log) | 0.039626  0.074833  0.007734  0.016514  -0.007059  0.029574  0.137518  0.067067  -0.038295  -0.045841  -0.051367  0.042681  0.035409  -0.011125  0.326080  0.119230  -0.171322 | 0.3882 |
| Stepwise multiple linear regression on uncorrelated variables | 5 | P_TRANSF (log)  T_LIXO (log)  T_DES1517 (log)  TRABPUB  T_FREQ5A6 (log) | 0.29728  -0.15386  0.10098  0.08204  0.05479 | 0.6704 |
| Stepwise multiple linear regression | 9 | T_FBBAS  T_FREQSUPER1517  T_DES1014  T_LIXO  T_FORA4A5  P_TRANSF (log)  P_SIUP (log)  T_FORA6A14 (log)  PESO1113 (log) | 0.02194  0.04875  0.05661  -0.09344  -0.01028  0.13507  0.01537  -0.02215  0.08825 | 0.5945 |
| Lasso regression | 14 | T_ATRASO_0_FUND  PREN60  RIND  CPR  P_COM  P_CONSTR  P_EXTR  TRABPUB  T_LIXO  T_OCUPDESLOC_1  peso1517  pesoRUR  T_ENV (log)  T_MED25M (log) | 0.11220  0.04844  -0.08752  -0.00763  0.03887  -0.07082  0.03036  0.01190  -0.04997  0.11949  -0.04929  0.06953  0.21991  0.08407 | 0.5196 |
| Principal component analysis (PCA) | 0.91185 of the variance recovered by PCs 1 to 6 | PC1  PC2  PC3  PC4  PC5  PC6 | 0.009915  -0.010542  -0.007275  0.009705  -0.012579  -0.027384 | 0.6547 |

**Pearson’s correlation (n = 17, Adj. R squared = 0.3882)**

**Stepwise multiple linear regressions on uncorrelated variables (n = 5, Adj. R squared = 0.6704)**

**Stepwise multiple linear regression (n = 9, Adj. R squared = 0.5945)**

**Lasso regression (n = 14, Adj. R squared = 0.5196)**

**Principal Component Analysis (n = 6, Adj. R Squared = 0.6547)**
